# Efficacy and safety of peanut epicutaneous immunotherapy in patients with atopic comorbidities

**DOI:** 10.1016/j.jacig.2022.07.009

**Published:** 2022-09-22

**Authors:** Carla M. Davis, Lars Lange, Kirsten Beyer, David M. Fleischer, Lara Ford, Gordon Sussman, Roxanne C. Oriel, Jacqueline A. Pongracic, Wayne Shreffler, Katharine J. Bee, Dianne E. Campbell, Todd D. Green, Romain Lambert, Aurélie Peillon, Philippe Bégin

**Affiliations:** a Department of Pediatrics, Immunology, Allergy and Retrovirology Section, Baylor College of Medicine, Houston, Tex; b Department of Pediatrics, St. Marien Hospital Bonn, Bonn, Germany; c Department of Pediatric Pneumology, Immunology and Intensive Care Medicine, Charité Universitatsmedizin Berlin, Berlin, Germany; d Children’s Hospital Colorado, University of Colorado Denver School of Medicine, Aurora, Colo; e Department of Allergy and Immunology, The Children’s Hospital at Westmead, Sydney, Australia; f Discipline of Child and Adolescent Health, University of Sydney School of Medicine, Sydney, Australia; g Division of Clinical Immunology and Allergy, Department of Medicine, University of Toronto, Toronto, Ontario, Canada; h Gordon Sussman Clinical Research, Toronto, Ontario, Canada; i Department of Pediatrics, Division of Allergy & Immunology, Icahn School of Medicine at Mount Sinai, New York, NY; j Ann & Robert H. Lurie Children’s Hospital of Chicago, Chicago, Ill; k Food Allergy Center, Departments of Pediatrics and Medicine, Massachusetts General Hospital, Boston, Mass; l DBV Technologies SA, Montrouge, France; m UPMC Children’s Hospital of Pittsburgh and University of Pittsburgh School of Medicine, Pittsburgh, Pa; n Oral Immunotherapy Clinic, CHU Sainte-Justine, Montreal, Quebec, Canada

**Keywords:** Epicutaneous immunotherapy, immunotherapy, Viaskin Peanut, desensitization, peanut allergy, food allergy, concomitant atopic conditions, children

## Abstract

**Background:**

Co-occurring atopic conditions are common in children with peanut allergy. As such, it is important to examine the safety and efficacy of epicutaneous immunotherapy with Viaskin Peanut 250 μg patch (VP250) in peanut-allergic children with these conditions.

**Objective:**

We sought to compare efficacy and safety of VP250 versus placebo in peanut-allergic children with/without ongoing atopic conditions at baseline, including asthma, atopic dermatitis/eczema, or concomitant food allergy.

**Methods:**

A subgroup analysis of peanut-allergic children aged 4 to 11 years enrolled in PEPITES (12 months) and REALISE (6 months) randomized, placebo-controlled, phase 3 trials was conducted. The efficacy outcome measure was the difference in prespecified responder rate between placebo and VP250 groups at month 12 based on eliciting dose of peanut protein using double-blind, placebo-controlled food challenge in PEPITES. Safety profiles were evaluated by baseline concomitant disease subgroup in all randomized subjects who received 1 or more dose of the study drug in PEPITES and REALISE pooled data.

**Results:**

Responder rates were significantly (*P* < .05, all comparisons) greater with VP250 compared with placebo treatment regardless of whether subjects had other atopic conditions. Safety and tolerability profiles were generally similar across subgroups, with no new safety concerns detected. A trend for both higher responder rates and rates of local reactions was observed in subjects with baseline atopic dermatitis versus those without. In subjects with concomitant food allergy at baseline, higher rates of treatment-emergent adverse events, but not study discontinuations or overall rates of anaphylaxis, were observed.

**Conclusions:**

The results support the safety and efficacy of VP250 for treating peanut-allergic children with or without concomitant atopic conditions.

Children with peanut allergy have high rates of concomitant allergic disorders such as asthma, atopic dermatitis (AD), and other food allergies, with rates substantially higher than in the general pediatric population.^1^, ^2^, ^3^, ^4^

Until recently, the only strategies for managing peanut allergy were allergen avoidance and emergency treatment of allergic reactions due to accidental peanut ingestion.^2^ Although these remain the standard of care for peanut allergy management, various types of allergen-specific immunotherapies are being evaluated for the treatment of peanut and other food allergies, with a proprietary form of peanut oral immunotherapy recently being approved for use by the US Food and Drug Administration and the European Commission.^5^^,^^6^ Epicutaneous immunotherapy with investigational Viaskin Peanut (DBV712) 250 μg represents a novel approach to the treatment of peanut allergy. Unlike oral immunotherapy, Viaskin Peanut 250 μg delivers microgram, rather than milligram, amounts of peanut protein (∼1/1000 of a peanut) through the skin, which may help to avoid potential risks associated with systemic exposure to peanut proteins during other forms of immunotherapy.^7^^,^^8^ The efficacy and safety of Viaskin Peanut 250 μg has been investigated in peanut-allergic individuals in several phase 2 and 3 controlled clinical trials.^9^, ^10^, ^11^, ^12^ The phase 3 PEPITES (NCT02636699) trial of peanut-allergic children aged 4 to 11 years demonstrated that Viaskin Peanut 250 μg was well tolerated and resulted in a statistically significant difference (*P* < .001) in treatment response rate compared with placebo after 12 months.^11^ The 6-month interim results of the phase 3 REALISE trial (NCT02916446) confirmed that Viaskin Peanut 250 μg is well tolerated in peanut-allergic children overall.^11^^,^^13^

Given the substantial rates of other atopic disorders in peanut-allergic children, it is important to examine whether these conditions influence the safety or efficacy profiles of Viaskin Peanut 250 μg. Together, the PEPITES and REALISE studies afforded an opportunity to evaluate this in subgroups of peanut-allergic children with comorbid asthma, AD, and/or multiple food allergies.

## Methods

### Study design and participants

This analysis evaluated data from peanut-allergic subjects aged 4 to 11 years who participated in the PEPITES and REALISE phase 3 trials, which have been described previously.^11^^,^^13^^,^^14^ Protocols and statistical analysis plans for PEPITES and REALISE are available in this article’s Online Repository at www.jaci-global.org. Briefly, PEPITES was a randomized, double-blind, placebo-controlled study of the safety and efficacy of Viaskin Peanut 250 μg in peanut-allergic children (see Fig E1, *A*, in this article’s Online Repository available at www.jaci-global.org).^11^ Inclusion criteria included an eliciting dose (ED) to peanut protein at baseline of less than or equal to 300 mg, more than 0.7 kU_A_/L peanut-specific IgE, and greater than or equal to 8 mm longest skin prick test (SPT) wheal diameter to peanut (≥6 mm SPT wheal diameter for children aged 4-5 years).

REALISE was a 36-month study including a 6-month randomized, double-blind, placebo-controlled period to assess the safety of Viaskin Peanut 250 μg. The REALISE study was designed to mirror real-world practice and did not require an oral food challenge as an enrollment criterion or as a primary outcome measure (Fig E1, *B*), consistent with anticipated use in clinical practice.^13^ Inclusion criteria included a well-documented medical history of IgE-mediated reactions after peanut ingestion that led to emergency department visit or a physician consultation, a peanut-specific IgE level greater than or equal to 14 kU_A_/L, and positive peanut SPT result with the longest wheal diameter greater than or equal to 8 mm. Key eligibility criteria for these studies are compared in Table E1 in this article’s Online Repository available at www.jaci-global.org. Note that patients with severe/unstable or uncontrolled asthma, as defined by the National Asthma Education and Prevention Program or Global INitiative for Asthma guidelines,^14^^,^^15^ were excluded from both studies. For analysis of safety, data from all eligible subjects in PEPITES (12-month randomized controlled trial) and REALISE (6-month randomized controlled trial) were pooled into the phase 3 placebo-controlled (P3PC) data set.

These studies were conducted in accordance with the International Council for Harmonisation Good Clinical Practice, the ethical principles of the Declaration of Helsinki, and local legal requirements. Parents or legal guardians of all participants provided signed written informed consent, and children aged at least 7 years provided their assent to participate or as required by local regulations.

### Ongoing concomitant disease cohorts

Subgroups included in this analysis were children with ongoing asthma, AD, or any concomitant food allergies (CFAs) as assessed by site principal investigator at baseline. Medical history was reported by system organ class and preferred term, using the latest available version of the Medical Dictionary for Regulatory Activities (MedDRA versions 20.0/20.1). Baseline history of an ongoing asthma diagnosis was assessed by investigators and defined on the basis of modified Asthma/Bronchospasm Standardized MedDRA queries version 20.1 using all narrow terms as well as the broad term “wheezing.” AD was by investigator assessment, and severity was assessed using the Scoring Atopic Dermatitis (SCORAD) index. History of food allergy other than to peanut was collected at baseline as part of the complete medical history. For this analysis, ongoing food allergy, other than peanut allergy, was considered the relevant variable, not a past history of food allergy to which the subject was tolerant at baseline study entry.

### Interventions

In each study, subjects were randomly assigned to receive either the Viaskin Peanut 250 μg patch or an identical-appearing placebo patch lacking peanut protein (details of randomization and treatment are available in this article’s Online Repository at www.jaci-global.org).^11^ Patches were applied daily, beginning with a 6-hour duration for the first week, increasing to 12 hours for the second week, and then for 24 hours (±4 hours) a day for 12 months in PEPITES and for 6 months in the double-blind period of REALISE.^11^^,^^13^

### Efficacy outcome—PEPITES

Efficacy data were derived from the PEPITES study only, because double-blind, placebo-controlled food challenges (DBPCFCs) were not required for REALISE. The primary efficacy outcome was the percentage of responders at month 12 in the active group compared with the placebo group, based on EDs at month-12 DBPCFC of at least 300 mg of peanut protein for the low-ED group (baseline ED ≤ 10 mg) or at least 1000 mg of peanut protein for the high-ED group (baseline ED > 10 mg).^11^

### Safety outcomes—PEPITES and REALISE

Treatment-emergent adverse events (TEAEs) were assessed in all randomized subjects who received at least 1 dose of study medication in the pooled data from PEPITES and REALISE. TEAEs were investigator-assessed by seriousness, severity (mild, moderate, or severe), and relationship to treatment (related, probably, possible, unlikely, or not related). TEAEs leading to permanent and temporary discontinuation were also reported. The most frequent TEAEs were also tabulated by subgroup and treatment assignment. In addition, TEAEs relevant to the specific baseline concomitant atopic conditions were assessed. Changes from baseline in the SCORAD^16^ index were assessed at month 3 and month 6 in the pooled data. Respiratory TEAEs in subjects with or without asthma at baseline, including asthma, cough, wheezing, dyspnea, and bronchospasm, were also assessed. FEV_1_ was also assessed and measured on a standardized calibrated spirometer for subjects 6 years or older following the American Thoracic Society guidelines.

### Statistical methods

Determination of baseline medical history and ongoing concomitant disease status was prespecified. For efficacy subgroup analyses, asthma and AD subgroups were prespecified and CFA was *post hoc*. All safety outcomes by concomitant disease subgroups were *post hoc*. Categorical data were summarized according to number of subjects or observations with nonmissing data, frequency counts, and percentages. Continuous data were summarized according to number of subjects or observations with nonmissing data using descriptive statistics such as mean, SD, median, first and third quartiles, or range (minimum-maximum).

For *post hoc* efficacy analyses from PEPITES, response rates for each subgroup were calculated using Wilson 95% CI; the difference between Viaskin Peanut 250 μg and placebo response rates was calculated with the Newcombe 2-sided 95% CI. *P* values from Fisher exact test are provided. Subjects with missing food challenge values at month 12 were counted as nonresponders in this analysis. Differences between groups across baseline concomitant disease status were assessed using the Fisher exact test. Subgroups by treatment interactions were assessed via a joint test from a logistic regression, with subgroup, treatment, and Subgroup by Treatment interaction as covariates. Multiple *post hoc* comparisons are described between subgroups and safety outcomes, but no adjustment was made for multiple comparisons. Comparison of baseline peanut-specific IgE, IgG4, and SPT between subjects with/without asthma, AD, and CFA was performed using nonparametric Kruskal-Wallis test. Analyses were performed using SAS version 9.4, SAS, Toronto.

## Results

### Subject disposition

**Fig 1:**
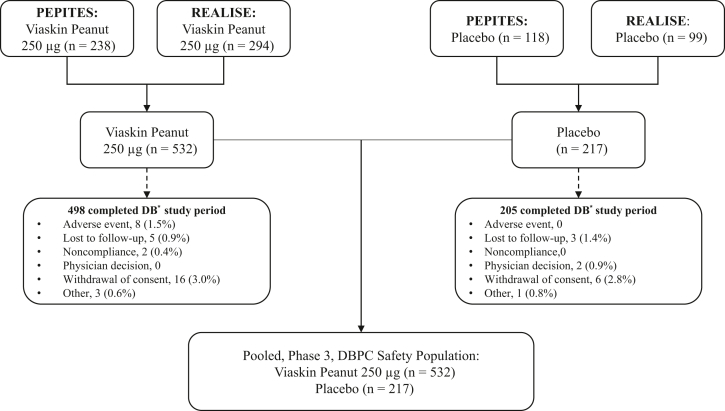
Subject disposition: Pooled, randomized, double-blind, placebo-controlled phase 3 studies. *DB*, Double-blind; *DBPC*, double-blind, placebo-controlled. ∗The DB period was 12 months in PEPITES and 6 months in REALISE.

The pooled P3PC (PEPITES plus REALISE) population consisted of a total of 749 subjects; 532 subjects treated with Viaskin Peanut 250 μg and 217 with placebo (Fig 1). A total of 238 subjects in PEPITES and 294 in REALISE received Viaskin Peanut 250 μg, with 118 subjects in PEPITES and 99 in REALISE receiving placebo. The disposition of subjects in each of these individual studies is shown in Figs E2 and E3 in this article’s Online Repository available at www.jaci-global.org.

### Baseline demographic and subject characteristics—P3PC pool

Demographic and subject baseline characteristics by treatment group and baseline asthma, AD, and/or CFA are presented in Table I. In the P3PC pool, rates of asthma, AD, and CFA were similar between subjects who received Viaskin Peanut 250 μg and placebo, with 45.7% and 42.9% having asthma, 45.7% and 47.5% having AD, and 56.2% and 55.8% having CFA at baseline, respectively. Subjects with asthma and CFA had higher baseline peanut-specific IgE (*P* < .0001 and *P* = .006, respectively) and peanut SPT (*P* = .014 and *P* = .05, respectively) than those without asthma or CFA. Subjects with CFA also had higher baseline peanut IgG4 than those without CFA (*P* < .001). No such patterns were observed between subjects with/without baseline AD, and no other trends were observed between groups based on distribution of age, sex, or ethnicity.

**Table I:** Baseline demographic characteristics (pooled PEPITES and REALISE) in patients with or without asthma, AD, or CFAs

Category	With asthma		Without asthma		With AD		Without AD		With CFA		Without CFA	
	Viaskin Peanut 250 μg (n = 243)	Placebo (n = 93)	Viaskin Peanut 250 μg (n = 289)	Placebo (n = 124)	Viaskin Peanut 250 μg (n = 243)	Placebo (n = 103)	Viaskin Peanut 250 μg (n = 289)	Placebo (n = 114)	Viaskin Peanut 250 μg (n = 299)	Placebo (n = 121)	Viaskin Peanut 250 μg (n = 233)	Placebo (n = 96)
Sex: male, n (%)	145 (59.7)	61 (65.6)	168 (58.1)	73 (59.9)	143 (58.8)	62 (60.2)	170 (58.8)	72 (63.2)	185 (61.9)	74 (61.2)	128 (54.9)	60 (62.5)
Age (y), mean ± SD	7.3 ± 2.2	7.3 ± 2.4	7.2 ± 2.1	7.2 ± 2.2	7.0 ± 2.2	7.2 ± 2.3	7.5 ± 2.1	7.3 ± 2.3	7.3 ± 2.2	7.4 ± 2.3	7.2 ± 2.2	7 ± 2.3
Race/ethnicity, n (%)												
White	187 (77.0)	71 (76.3)	233 (80.6)	96 (77.4)	181 (74.5)	79 (76.7)	239 (82.7)	88 (77.2)	230 (76.9)	87 (71.9)	190 (81.5)	80 (83.3)
Black/African American	9 (3.7)	2 (2.2)	0	2 (1.6)	6 (2.5)	3 (2.9)	3 (1.0)	1 (0.9)	6 (2.0)	2 (1.7)	3 (1.3)	2 (2.1)
Asian	20 (8.2)	12 (12.9)	29 (10.0)	15 (12.1)	30 (12.3)	14 (13.6)	19 (6.6)	13 (11.4)	31 (10.4)	20 (16.5)	18 (7.7)	7 (7.3)
Other	19 (7.8)	8 (8.6)	24 (8.3)	9 (7.3)	18 (7.4)	7 (6.8)	25 (8.7)	10 (8.8)	26 (8.7)	10 (8.3)	17 (7.3)	7 (7.3)
Not collected	8 (3.3)	0	3 (1.0)	2 (1.6)	8 (3.3)	0	3 (1.0)	2 (1.8)	6 (2.0)	2 (1.7)	5 (2.1)	0
Peanut-specific IgE (kU/L), median (Q1, Q3)	112.6 (52.7, 268.0)	151.0 (66.9, 330.0)	73.9 (25.9, 207.0)	82.2 (32.1, 231.5)	85.5 (37.1, 221.0)	109.0 (31.9, 261.0)	87.7 (36.6, 246.0)	92.7 (41.9, 275.0)	95.3 (38.9, 274.0)	141.0 (43.6, 334.0)	76.5 (33.1, 198.0)	84.2 (37.5, 210.0)
IgG4 (mg/L), median (Q1, Q3)	0.8 (0.3, 1.5)	0.9 (0.4, 1.6)	0.7 (0.3, 1.3)	0.6 (0.3, 1.5)	0.7 (0.3, 1.5)	0.9 (0.4, 1.6)	0.7 (0.3, 1.4)	0.6 (0.2, 1.5)	0.8 (0.4, 1.6)	1.0 (0.4, 2.0)	0.6 (0.2, 1.2)	0.5 (0.2, 1.3)
Baseline SPT wheal diameter (mm), median (range)	11.5 (6.0- 43.0)	11.5 (6.0- 33.5)	11.0 (5.0- 29.0)	11.0 (6.0- 25.0)	11.0 (6.0- 31.0)	12.0 (7.0- 27.5)	11.0 (5.0- 43.0)	10.5 (6.0- 33.5)	11.5 (5.0- 43.0)	11.5 (6.0- 33.5)	11.0 (6.0- 35.0)	11.0 (6.5- 25.5)

Percentages are based on the number of subjects with nonmissing values for each group.

*Q1*, First quartile; *Q3*, third quartile.

### Efficacy by baseline asthma, AD, and CFA—PEPITES

**Fig 2:**
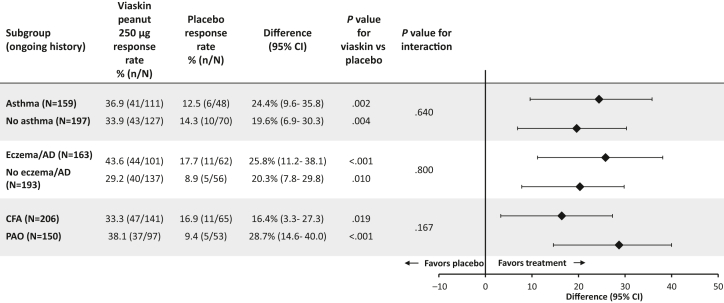
Responder rates by treatment arm and concomitant medical condition subgroup. *PAO*, Peanut allergy only. Response rates for each subgroup were calculated using Wilson 95% CI; the difference between Viaskin Peanut 250 μg and placebo response rates was calculated with the Newcombe 2-sided 95% CI. *P* value from Fisher exact test is presented. Subjects with missing food challenge values at month 12 were counted as nonresponders in this analysis.

In the PEPITES population, the proportion of subjects who reached the primary end point of ED response (as defined in methods) at month-12 DBPCFC differed significantly between the Viaskin Peanut 250 μg and placebo treatment arms regardless of whether or not the subjects had asthma, AD, or CFA at baseline (Fig 2). Responder rates in subjects treated with Viaskin Peanut 250 μg with and without asthma were 36.9% and 33.9%, respectively, and were significantly higher than with placebo (12.5% [*P* = .002] and 14.3% [*P* = .004]); responder rates did not differ significantly by baseline asthma status (interaction *P* = .640). Similar results were observed in subjects with AD, with responder rates in subjects with and without AD treated with Viaskin Peanut 250 μg of 43.6% and 29.2%, respectively, and were higher than those in corresponding placebo arms (25.8% [*P* < .001] and 20.3% [*P* < .01]) and did not differ by baseline AD status (interaction *P* = .80). Likewise, responder rates in subjects with and without CFA treated with Viaskin Peanut 250 μg were 33.3% and 38.1%, respectively, compared with 16.9% (*P* = .019) and 9.4% (*P* < .001) in the placebo arm; responder rates did not differ significantly by baseline CFA status (interaction *P* = .167).

When responder rates were examined by the subpopulation who had all 3 comorbidities compared with subjects who had 0, 1, or 2 comorbidities, the responder rates were 37.2% and 34.9%, respectively, with a *P* value for interaction of .51 (see Fig E4 in this article’s Online Repository available at www.jaci-global.org).

### Safety

#### TEAEs by treatment group and concomitant atopic condition—P3PC pool

Overall TEAEs by treatment group and in subjects with and without asthma, AD, and/or CFA are presented in Table II. Most subjects across all groups experienced at least 1 TEAE. Most TEAEs were mild or moderate, and the incidence of severe TEAEs was low. Across all 3 key concomitant atopic disease subgroups, there was a greater number of treatment-related TEAEs in the Viaskin Peanut 250 μg group (n = 230 [43.2%]) than in the placebo group (n = 53 [24.4%]).

**Table II:** TEAEs by treatment group and with or without asthma, AD, or CFAs—P3PC pool

Category	With asthma		Without asthma		With AD		Without AD		With CFA		Without CFA	
	Viaskin Peanut 250 μg (n = 243)	Placebo (n = 93)	Viaskin Peanut 250 μg (n = 289)	Placebo (n = 124)	Viaskin Peanut 250 μg (n = 243)	Placebo (n = 103)	Viaskin Peanut 250 μg (n = 289)	Placebo (n = 114)	Viaskin Peanut 250 μg (n = 299)	Placebo (n = 121)	Viaskin Peanut 250 μg (n = 233)	Placebo (n = 96)
Any TEAE, n (%)	226 (93.0)	82 (88.2)	263 (91.0)	106 (85.5)	224 (92.2)	88 (85.4)	265 (91.7)	100 (87.7)	281 (94.0)	108 (89.3)	208 (89.3)	80 (83.3)
Mild	214 (88.1)	78 (83.9)	249 (86.2)	96 (77.4)	217 (89.3)	83 (80.6)	246 (85.1)	91 (79.8)	266 (89.0)	102 (85.1)	197 (84.5)	71 (74.0)
Moderate	113 (46.5)	40 (43.0)	126 (43.6)	38 (30.6)	111 (45.7)	39 (37.9)	128 (44.3)	39 (34.2)	151 (50.5)	41 (33.9)	88 (37.8)	37 (38.5)
Severe	8 (3.3)	1 (1.1)	10 (3.5)	2 (1.6)	5 (2.1)	1 (1.0)	13 (4.5)	2 (1.8)	13 (4.3)	0	5 (2.1)	3 (3.1)
Serious	3 (1.2)	5 (5.4)	6 (2.1)	2 (1.6)	2 (0.8)	3 (2.9)	7 (2.4)	4 (3.5)	4 (1.3)	4 (3.3)	5 (2.1)	3 (3.1)
Treatment-related∗	96 (39.5)	24 (25.8)	134 (46.4)	29 (23.4)	113 (46.5)	34 (33.0)	117 (40.5)	19 (16.7)	139 (46.5)	31 (25.6)	91 (39.1)	22 (22.9)
Serious	2 (0.8)	0	2 (0.7)	0	1 (0.4)	0	3 (1.0)	0	2 (0.7)	0	3 (1.3)	0
TEAEs leading to permanent discontinuation	2 (0.8)	0	6 (2.1)	0	4 (1.6)	0	4 (1.4)	0	5 (1.7)	0	3 (1.3)	0
TEAEs leading to temporary discontinuation	34 (14.0)	10 (10.8)	40 (13.8)	8 (6.5)	32 (13.2)	8 (7.8)	42 (14.5)	10 (8.8)	49 (16.4)	9 (7.4)	25 (10.7)	9 (9.4)
Treatment-related local TEAEs	80 (32.9)	20 (21.5)	111 (38.4)	21 (16.9)	94 (38.7)	26 (25.2)	97 (33.6)	15 (13.2)	115 (38.5)	21 (17.4)	76 (32.6)	20 (20.8)
Anaphylactic reactions	10 (4.1)	3 (3.2)	19 (6.6)	2 (1.6)	11 (4.5)	3 (2.9)	18 (6.2)	2 (1.8)	18 (6)	3 (2.5)	11 (4.7)	2 (2.1)

∗ Considered related to study treatment when reported as possibly related, probably related, or related. Considered unrelated to investigational product when reported as unlikely related or unrelated.

Among subjects treated with Viaskin Peanut 250 μg, those with asthma did not have higher rates (or trends) of mild, moderate, severe, or serious TEAEs compared with those without asthma; nor did they have trends or higher rates of TEAEs leading to study discontinuation, TEAEs deemed treatment related, or anaphylaxis. Subjects with asthma receiving Viaskin Peanut 250 μg had a higher rate of asthma, cough, and wheeze reported than those without asthma (see Table E2 in this article’s Online Repository available at www.jaci-global.org), but they had similar rates as those with asthma who received placebo. In addition, no change was noted in pulmonary function as measured by FEV_1_ percent predicted over the study duration in any subgroup. Similar rates of local application-site reactions leading to topical corticosteroid use were reported in subjects with and without an ongoing history of eczema (25.1% and 22.1%, respectively).

In general, the presence or absence of an ongoing history of AD at baseline did not affect the TEAEs reported by category; the equivalent treatment group in each subgroup reported a similar frequency of TEAEs in each category and TEAEs leading to study discontinuation. Treatment-related TEAEs were higher in those with AD than in those without (46.5% vs 40.5%), with most of these being local application-site reactions. Regardless of the presence or absence of ongoing AD, no clinically relevant changes or trends from baseline were seen in any SCORAD parameters, with no median change from baseline in any subgroup at 3 or 6 months, and no difference between Viaskin Peanut 250 μg and placebo groups (see Table E3 in this article’s Online Repository available at www.jaci-global.org). In addition, in subjects with an ongoing history of AD, there was no indication of an increase in generalized AD disease flare with active treatment (reported in 36 [14.8%] subjects in the Viaskin Peanut 250 μg group vs 15 [14.6%] subjects in the placebo group).

By *post hoc* analysis, with Viaskin Peanut 250 μg treatment there was a trend for a greater proportion of subjects experiencing TEAEs in subjects with CFA (94.0% [95% CI, 90.7-96.2]) compared with those without (89.3% [95% CI, 84.6-92.6]). This difference was seen for overall TEAEs (as above), mild, moderate, and severe TEAEs, treatment-related TEAEs, and TEAEs leading to temporary treatment discontinuation. No trends for differences were observed between those with and without CFA with regard to rates of serious TEAEs, permanent discontinuations attributed to Viaskin Peanut 250 μg, or anaphylaxis (as reported by investigators).

Rates of TEAEs in the subpopulation of subjects with all 3 comorbidities, without all 3 comorbidities, and by each pair of comorbidities are presented in Table E4 in this article’s Online Repository available at www.jaci-global.org. Overall, rates of TEAEs, including anaphylactic reactions and treatment-related TEAEs, were similar in these comparisons. In the subpopulation of subjects who had all 3 comorbidities compared with those with 0, 1, or 2 comorbidities (Table E4, *A*), treatment-related TEAEs were 38.6% versus 35.4%, with adverse events leading to permanent and temporary discontinuation at 2.3% versus 1.4% and 15.9% versus 13.5%, respectively.

## Discussion

Unmet treatment needs in children with peanut allergy are considerable and potentially complicated by the presence of concomitant atopic conditions. A substantial number of subjects in this analysis had concomitant atopic conditions at baseline, consistent with prevalence rates reported in the published literature.^1^, ^2^, ^3^, ^4^ These rates underscore the importance of assessing the safety and efficacy of epicutaneous immunotherapy with Viaskin Peanut 250 μg in subgroups with other atopic conditions.

This subgroup analysis of 2 phase 3 studies, PEPITES and REALISE, demonstrated equivalent safety, tolerability, and efficacy of Viaskin Peanut 250 μg in subjects with peanut allergy regardless of an ongoing history of asthma, AD, or CFA at baseline. Efficacy based on responder rates significantly favored Viaskin Peanut 250 μg over placebo irrespective of the presence or absence of a concomitant atopic condition. Safety was generally similar regardless of concomitant atopic conditions and was in line with results from the primary analyses.

Not unexpectedly, a trend for higher rates of local reactions (predominately mild and moderate in severity) was observed in subjects with baseline AD compared with those subjects without. Importantly, no overall flare of AD was observed with therapy. Similarly, there was no difference between rates of respiratory-based TEAEs in subjects with asthma at baseline who received Viaskin Peanut 250 μg compared with placebo, suggesting that treatment did not contribute to these TEAEs. Overall, these safety and efficacy findings are similar to those of the primary results from PEPITES and REALISE^11^^,^^13^ and allow for appropriate anticipatory guidance/education for subjects with AD related to local application-site reactions.

We also observed a trend of higher rates of all TEAEs in children with CFA at baseline, although not in study discontinuations or overall rates of anaphylaxis. Because children with CFA are more likely to also have concomitant AD, this may contribute to the observed trend for higher rates of local reactions in this subgroup. For example, within the pooled population of children treated with Viaskin Peanut 250 μg, 51.5% (n = 154 of 299) of those with an ongoing history of a food allergy other than peanut had a history of ongoing AD compared with 38.2% (n = 89 of 233) of those without an ongoing history of an additional food allergy. Proportions were similar in the pooled placebo population: 55.4% (n = 67 of 121) versus 37.5% (n = 36 of 96).

Interestingly, a recent multivariate analysis of PEPITES results demonstrated that higher SCORAD at baseline was the fourth most highly predictive variable of having a higher ED at month 12.^17^ Although this current analysis reported a numerical difference in responder rate in subjects treated with Viaskin Peanut 250 μg with ongoing AD at baseline versus those without (43.6% vs 29.2%), the treatment effect versus placebo was similar in the 2 groups and the interaction *P* value was nonsignificant (*P* = .8).

As with any study, certain limitations should be considered when interpreting the data. In this study, although ongoing concomitant atopic conditions were prespecified at baseline and captured in the medical history, analyses of efficacy and safety by these subgroups were *post hoc*. Multiple *post hoc* comparisons are described between subgroups and safety outcomes, but because no adjustment was made for type 1 error, some trends noted may be by chance. Subjects may have more than 1 of these baseline atopic disorders of interest, and so the groups are not mutually exclusive. Although there were also differences in study design between PEPITES and REALISE, most were minor. Children with severe or unstable asthma were excluded from participation in both studies because of concerns related to the obligatory DBPCFC in PEPITES and the voluntary DBPCFC during REALISE, limiting the conclusion(s) that can be drawn regarding the safety of Viaskin Peanut 250 μg in that asthma subgroup. PEPITES and REALISE also differed in the duration of the double-blind placebo-controlled period at 12 months in PEPITES and 6 months in REALISE. Nevertheless, the 6-month period appears to have been sufficient to characterize safety and tolerability.

Taken together, the results reported herein support the safety, efficacy, and utility of Viaskin Peanut 250 μg for treating peanut-allergic children with or without concomitant atopic conditions, such as asthma, AD, or multiple food allergies. In children who have AD and/or multiple food allergies, it may be prudent to highlight the possibility of local application-site reactions (given the observed trends reported here) during the initial treatment phase. As previously reported, asthma exacerbations were not more frequent in subjects receiving placebo versus treatment, and no special precautions appear to be required for children with stable, nonsevere asthma.^11^ As new treatment options for peanut allergy continue to emerge, it will be important to assess the impact of commonly occurring concomitant atopic conditions on treatment effect in this patient population. The results described here suggest that the ongoing presence of asthma, AD, and other food allergies does not impact the efficacy or safety profile of Viaskin Peanut 250 μg in children with peanut allergy.

**Clinical implications**

The results suggest that the ongoing presence of co-occurring atopic conditions does not impact the efficacy or safety profile of Viaskin Peanut 250 μg in children with peanut allergy.

## Supplementary data

Supplement 3

Supplementary Tables E1-E3

Supplementary Table E4

Fig E1

Fig E2

Fig E3

Fig E4
