# Supplementary material for: Efficacy and safety of peanut epicutaneous immunotherapy in patients with atopic comorbidities
Source: J Allergy Clin Immunol Glob. 2022 Sep 22;2(1):69–75. doi: 10.1016/j.jacig.2022.07.009 (PMC10509968; doi:10.1016/j.jacig.2022.07.009)
Supplement: Supplement 1a [file mmc1.pdf]

A DOUBLE-BLIND, PLACEBO-CONTROLLED, RANDOMIZED PHASE III PIVOTAL TRIAL TO ASSESS THE EFFICACY AND SAFETY OF PEANUT EPICUTANEOUS IMMUNOTHERAPY WITH VIASKIN® PEANUT IN PEANUT-ALLERGIC CHILDREN (PEPITES STUDY)

**Sponsor:**	DBV TECHNOLOGIES S.A.<br>Green Square – Bâtiment D<br>80/84, rue des Meuniers<br>92220 Bagneux, France<br>Tel: + 33 1 55 42 78 78<br>Fax:+ 33 1 43 26 10 83
**Clinical Research Organization:**	PAREXEL International (IRL) Limited (“PAREXEL”)<br>70 Sir John Rogerson’s Quay<br>Dublin 2<br>Ireland
**Sponsor Protocol No.:**	**PEPITES**
**IND No.:**	14366
**EudraCT No.:**	2015-002461-37
**Study Drug Name:**	Viaskin® Peanut (DBV712)
**Development Phase:**	III (Pivotal)
**Versions and Dates of Protocol:**	Protocol Version 1.0, 20 Jul 2015<br>Protocol Version 2.0, 09 Dec 2015 (including Protocol Amendment 1)

The study will be conducted according to the protocol and in compliance with Good Clinical Practice (GCP), with the Declaration of Helsinki and with other applicable regulatory requirements.

This document contains confidential information of DBV Technologies.
Do not copy or distribute without written permission from the Sponsor.
CONFIDENTIAL

SIGNATURE PAGE

Declaration of Sponsor or Responsible Medical Officer

Title: A Double-blind, Placebo-controlled, Randomized Phase III Pivotal Trial to Assess the Efficacy and Safety of Peanut Epicutaneous Immunotherapy with Viaskin® Peanut in Peanut-allergic children (PEPITES Study)

This study protocol was subjected to critical review. The information it contains is consistent with current knowledge of the risks and benefits of the investigational product, as well as with the moral, ethical, and scientific principles governing clinical research as set out in the Declaration of Helsinki, [2013] (APPENDIX 1), and the guidelines on Good Clinical Practice.

[redacted]

Declaration of the Investigator

Title: A Double-blind, Placebo-controlled, Randomized Phase III Pivotal Trial to Assess the Efficacy and Safety of Peanut Epicutaneous Immunotherapy with Viaskin® Peanut in Peanut-allergic Children (PEPITES Study)

I have read and understood and agree to abide by all the conditions and instructions contained in this protocol.

All documentation for this study that is supplied to me and that has not been previously published will be kept in the strictest confidence. This documentation includes this study protocol, Investigator's Brochure (IB), electronic case report form (CRF), and other scientific data.

The study will not be commenced without the prior written approval of a properly constituted Institutional Review Board (IRB) or Independent Ethics Committee (IEC). No changes will be made to the study protocol without the prior written approval of the Sponsor and the IRB or IEC, except where necessary to eliminate an immediate hazard to the subjects.

Responsible Investigator of the local study center

____________________________________ ____________________
Signature Date

____
Name (block letters)

____
Title (block letters)

____
Institution (block letters)

____
Phone number

PROTOCOL SYNOPSIS

**Title**	A Double-blind, Placebo-controlled, Randomized Phase III Pivotal Trial to Assess the Efficacy and Safety of Peanut Epicutaneous Immunotherapy with Viaskin® Peanut in Peanut-allergic Children (PEPITES Study)
**Sponsor Study No.**	PEPITES
**Phase**	III (Pivotal)
**Sponsor**	DBV Technologies
**Study Centers**	This is a multicenter study to be conducted in Australia, Europe and North America. It is planned that approximately 28 to 40 sites from 4 to 7 countries will participate.
**Objective**	To assess the efficacy and safety of Viaskin® Peanut to induce desensitization to peanut in peanut-allergic subjects 4 through 11 years of age after a 12-month treatment period by EPicutaneous ImmunoTherapy (EPIT).
**Design**	This is a 12-month, Phase III, double-blind, placebo-controlled, randomized study to assess the efficacy and safety of Viaskin® Peanut, dosed at 250 µg peanut protein (per patch) in peanut-allergic children from 4 through 11 years of age.<br>The overall maximum study duration for each subject is approximately 61 weeks (6-week screening period, 12-month treatment period and 2-week follow-up period).<br>During the maximum 6-week screening period, subjects will undergo a first screening visit and an entry double-blind, placebo-controlled food challenge (DBPCFC) to peanut to confirm their allergy and their entry peanut eliciting dose (ED). The starting dose of the challenge will be 1 mg peanut protein and will escalate up to a highest dose of 300 mg peanut protein. Subjects who react at or below the dose of 300 mg peanut protein are considered eligible. Randomization of eligible subjects will occur in a 2:1 ratio to Viaskin® Peanut dosed at 250 µg peanut protein (active treatment) or placebo. Subjects will be stratified at randomization by their entry/screening DBPCFC ED in 1 of the following 2 strata and by study center:<br>• Stratum 1: children with a screening ED of 1 mg, 3 mg or 10 mg;<br>• Stratum 2: children with a screening ED of 30 mg, 100 mg or 300 mg.<br>The randomization scheme will ensure that the ratio of active treatment to placebo is maintained in each stratum.<br>Subjects randomized in the study and consenting for the genetic analysis on a voluntary basis will be assessed for mutations in the filaggrin gene. Subjects' participation for this genetic analysis will be optional.<br>Subjects will apply a Viaskin® patch containing either peanut protein or placebo daily for a period of 12 months. At Month 12, a post-treatment DBPCFC to peanut will be performed, with a starting dose of 1 mg peanut protein with escalation up to a highest dose of 2,000 mg peanut protein. This evaluation will help determine the primary efficacy endpoint of this pivotal study.<br>Subjects will undergo other efficacy parameter assessments at Months 3, 6 and 12, including immunological changes in peanut-specific immunoglobulin E (IgE) and immunoglobulin G4 subtype (IgG4) and skin prick tests (SPTs).<br>Key assessments of global safety will be performed at each study visit by the Investigators, including skin observation of the patch areas of application (inter-scapular area of the back), spirometry, peak expiratory flow (PEF) measurements, vital signs, physical examinations and clinical laboratory

	assessments. Atopic dermatitis will also be assessed at baseline and at Months 3, 6 and 12 using the SCORAD (Scoring atopic dermatitis), for which specific training will be provided for better use and accurate assessment.<br>In between visits, the severity of local skin reactions will be assessed on a daily basis by the subjects (parents/guardians) in their diary for 6 months. Any other adverse events (AEs), local skin reactions occurring after the first 6 months of treatment and any concomitant medications will also be reported in the diary by the subjects and this will be reviewed by the site medical staff at each patient visit.<br>In addition, the adhesion of the patch to the skin, especially the maintenance of the occlusion of the patch, will be assessed by the subjects' parents/guardians for 28 days of treatment (whenever possible, these should be consecutive days). This assessment will be conducted during a period between Month 3 and Month 9. On the same days, photographs of the patches attached to the skin will be made by the subjects' parents/guardians as instructed. The review of these photos will allow the Investigators to control and ensure that the parents' assessments of the patch adhesion were satisfactory. The trained site staff will also assess the patch adhesion of all subjects at each subject visit.<br>After completion of the PEPITES study, all subjects, including the placebo subjects, will be offered the opportunity to participate in an open-label extension study to receive Viaskin® Peanut 250 µg for 24 additional months of treatment. Subjects who will decide to roll-over into the extension study will have their last PEPITES study visit at Visit 11 and they will start the extension study at that visit. Subjects who decide not to roll-over into the extension study will continue their visit schedule up to Visit 12.
**Treatment**	The study drug, Viaskin® Peanut, is an epicutaneous delivery system (Viaskin® patch) containing a dry deposit of a formulation of peanut protein extract. The drug substance is an unmodified, lyophilized peanut extract produced from the extraction and freeze drying of defatted peanut flour of biological origin, the peanut seed from the Virginia variety of *Arachis hypogaea*.<br>The Viaskin® patch must be applied on the skin for 24 hours (± 4 hours of allowance) every day, which means a new patch will be applied every 24 hours on the inter-scapular area of the back of the subjects. To increase the safety of the Viaskin® patch at the initiation of treatment, the duration of application of the Viaskin® patch will be progressively increased as follows:<br>• During the first week (from Day 1 through Day 7), the patches will be applied for 6 hours every day;<br>• During the second week (from Day 8 through Day 14), the patches will be applied for 12 hours every day;<br>• From the third week onwards (Day 15), the patches will be applied for the entire 24 hours daily.
**Number of Patients**	It is planned to screen approximately 470 subjects to achieve 330 subjects randomized in a 2:1 ratio (220 subjects in the Viaskin® Peanut group and 110 subjects in the placebo group) in this study. Assuming that the drop-out rate might be up to 15%, this will allow ensuring that approximately 280 subjects complete the study.<br>Throughout the screening and randomization process, and to guarantee an adequate and sufficient representation of subjects of 4, 5 and 11 years of age (the youngest and the oldest of the age range), a minimum of 25 subjects of each of these 3 ages must be randomized. Each of these ages will thus represent a minimum of 7.5% of the overall number of randomized subjects.

	Two subgroups based on subjects' age are also defined: the 4-5 year-old and the 6-11 year-old subjects. It is planned that a minimum number of 75 children of either 4 or 5 years of age must be randomized. The 4 to 5 year-old subgroup will thus represent a minimum of 22.7% of the overall number of randomized subjects.
**Population**	Subjects will be enrolled in this study only if they meet, among others, the following inclusion criteria: Male or female children aged 4 through 11 years at Visit 1; physician-diagnosed peanut allergy or children with a well-documented medical history of IgE-mediated reactions after ingestion of peanut and currently following a strict peanut-free diet.<br><br>Subjects will not be enrolled, if they, among other exclusion criteria, have a history of severe anaphylaxis to peanut with any of the following symptoms: hypotension, hypoxia, neurological compromise (collapse, loss of consciousness or incontinence); if they have a severe reaction during the entry/screening DBPCFC (defined as need for intubation, hypotension persisting after epinephrine administration, and/or the need for ≥3 doses of epinephrine), or if they have an objective IgE-mediated reaction to the placebo formula during the entry/screening DBPCFC.<br><br>All subjects will continue with their usual peanut-free diet and label reading of food products to avoid as much as possible any accidental peanut consumption for the duration of the study.
**Criteria for Evaluation of Efficacy**	**Primary efficacy endpoint:**<br>The primary efficacy endpoint is the percentage of treatment responders at Month 12 in the active Viaskin® Peanut 250 µg group compared to the placebo group in the overall population. A subject is defined as a treatment responder if:<br>• The initial ED was >10 mg peanut protein and the ED is ≥1,000 mg peanut protein at the post-treatment DBPCFC at Month 12, or;<br>• The initial ED was ≤10 mg and the ED is ≥300 mg peanut protein at the post-treatment DBPCFC at Month 12.<br>**Secondary efficacy endpoints:**<br>• Percentage of treatment responders at Month 12 in the active Viaskin® Peanut 250 µg group compared to the placebo group in each of the 2 screening ED strata.<br>• Percentage of treatment responders at Month 12 in the active Viaskin® Peanut 250 µg group compared to the placebo group in each of the2 age subgroups: 4 to 5 years-old; 6 to 11 years-old.<br>• Mean and median cumulative reactive dose of peanut protein and change from baseline at Month 12 in the active Viaskin® Peanut 250 µg group versus the placebo group, overall as well as in each of the 2 screening ED strata;<br>• Mean and median ED of peanut protein and change from baseline at Month 12 in the active Viaskin® Peanut 250 µg group versus the placebo group, overall as well as in each of the 2 screening ED strata;<br>• Percentage of subjects responsive (those showing objective symptoms leading to DBPCFC stop) to a cumulative dose ≥1,444 mg peanut protein at the post-treatment DBPCFC at Month 12 in the active Viaskin® Peanut 250 µg group versus the placebo group, overall as well as in each of the 2 screening ED strata;<br>• Percentage of subjects unresponsive (those showing no objective symptoms leading to DBPCFC stop) to a cumulative dose ≥1,444 mg peanut protein at the post-treatment DBPCFC at

Month 12 in the active Viaskin® Peanut 250 µg group versus the placebo group, overall as well as in each of the 2 screening ED strata;

- Percentage of subjects unresponsive (those showing no objective symptoms leading to DBPCFC stop) to the highest dose of peanut protein, which is the percentage of subjects who pass the post-treatment DBPCFC at Month 12 in the active Viaskin® Peanut 250 µg group versus the placebo group, overall as well as in each of the 2 screening ED strata.

Other efficacy endpoints:

- Change from baseline in peanut-specific IgE and IgG4 at months 3, 6 and 12 in the active Viaskin® Peanut 250 µg group versus the placebo group, overall as well as in each of the 2 screening ED strata;
- Change from baseline in peanut skin prick testing maximum average wheal diameters at months 3, 6 and 12 in the active Viaskin® Peanut 250 µg group versus the placebo group, in the overall population;
- Description of the quality of life questionnaires (Food Allergy Quality of Life Questionnaire [FAQLQ]/Food Allergy Independent Measure [FAIM]) and change from baseline in FAQLQ score at Month 12 in the overall population (for those countries where the translated and validated questionnaires are available and used).

Criteria for Evaluation of Study Drug Safety

The following safety criteria will be evaluated:

- AEs and treatment-emergent adverse events (TEAEs) by System Organ Class (SOC) and Preferred Terms (PTs);
- TEAEs by maximum severity and relatedness to Viaskin® Peanut 250 µg;
- Incidence, duration and maximum severity of local cutaneous Viaskin® Peanut 250 µg-induced AEs as assessed by the subject;
- Severity of local cutaneous Viaskin® Peanut 250 µg-induced AEs as assessed by the Investigator;
- Adverse events of special interest (AESI) including grade 4 local cutaneous reactions and systemic allergic AEs considered related to Viaskin® Peanut 250 µg;
- Serious adverse events (SAEs) by SOC and PTs, and relatedness to Viaskin® Peanut 250 µg;
- Laboratory data, physical examinations and vital signs;
- Spirometry results or PEF results.

The above criteria will be studied in the overall Safety population, as well as for the age ranges 4 to 5 years, 6 to 8 years and 9 to 11 years, and for each of the screening ED strata.

Criteria for Evaluation of Study Procedure Safety

- Objective symptoms elicited during the entry/screening DBPCFC and post-treatment DBPCFC at Month 12 by severity;
- Change in severity of objective symptoms elicited during the DBPCFC from baseline to Month 12 in the active Viaskin® Peanut 250 µg group versus the placebo group;
- SAEs elicited during the entry/screening DBPCFC and post-treatment DBPCFC at Month 12.

A Data and Safety Monitoring Board (DSMB) composed of experts in food allergy and in the methodology of clinical studies will review study safety data at specific intervals during the study and on an *ad hoc* basis. A specific DSMB meeting will be held when the first 15 subjects 4 to 5 years of age have been randomized and treated for at least 4 weeks (that is, have

	completed the Month 1 visit). This is to assess very early in the study that the safety of Viaskin® Peanut is acceptable in the youngest subjects.
**Exploratory endpoints**	• Change from baseline in IgE and IgG4 specific to peanut protein components at months 3, 6 and 12 in the active Viaskin® Peanut 250 µg group versus the placebo group;<br>• Enumeration and characterization of reactions triggered by accidental consumption of peanut during the study;<br>• Analysis of "risk-taking behavior" of subjects (voluntary peanut consumption) during the study;<br>• Epigenetic modifications of the promoters of some specific genes;<br>• Safety sub-analysis in subjects with mutations in the filaggrin gene versus wild type subjects;<br>• Sensitization status to some other allergies and their evolution over the study period;<br>• SCORAD evolution over time.

Statistical Methods

Analysis sets:

Safety population:

The Safety population will be comprised of all subjects who are randomized and have received at least 1 dose of the study drug. This population will be used to assess comparative safety information. In case the wrong study drug is dispensed, the subject will be analyzed according to the study drug received for the longest period of time.

Intent-to-treat (ITT) population:

The ITT population will be comprised of all subjects who are randomized. This population will be used to assess comparative efficacy information.

Full analysis set (FAS):

The FAS will be comprised of all subjects who are randomized and have performed at least the peanut challenge of the second DBPCFC at Month 12.

Per-protocol (PP) population:

The PP population will include all subjects from the ITT population who do not have major deviations from the protocol that may affect the primary efficacy endpoint (for instance, subjects who have not gone through the second DBPCFCs at Month 12, subjects with a global treatment compliance below 80% etc.). The PP population will be used to perform confirmatory analyses of the primary and secondary efficacy evaluations.

Statistical methods:

Categorical variables will be summarized using the number of observations and percentages. The denominator for percentages will be the number of subjects in the population with data available unless otherwise stated. Continuous variables will be summarized using descriptive statistics (number of observations [n], mean, standard deviation, minimum, first quartile [Q1], median, third quartile [Q3], and maximum).

Primary efficacy analysis:

The primary efficacy endpoint is the percentage of treatment responders at Month 12 in the active Viaskin® Peanut 250 µg group compared to the placebo group.

The null hypothesis is that the percentage of treatment responders in the active Viaskin® Peanut 250 µg group is the same as that in the placebo group (which means the parameter estimate associated to the treatment group is equal to zero).

The primary efficacy analysis will be performed on the ITT population (that is on all randomized subjects), using last observation carried forward (LOCF) imputation (which means that subjects with missing DBPCFC value at Month 12 will be considered as non-responders). An exact logistic regression will be used to compare the percentage of treatment responders at Month 12 in the active Viaskin® Peanut 250 µg group versus the placebo group, adjusting for screening ED stratum and including the treatment group as fixed effect. A 2-sided, 1% significance level will be used to test the null hypothesis. The corresponding p-value, as well as the number and percentage (95% confidence interval [CI]) of responders, will be presented by treatment group. The difference between active treatment and placebo response rates and the corresponding 95% CI will be presented. Clinical relevance of treatment effect will be evaluated based on the lower bound of the 95% CI being higher or equal to 15%. This 15% difference is intended to exhibit a clear robustness of the treatment effect.

Relative risks of achieving response in the active group compared to the placebo group will be assessed using a log-binomial regression with the screening ED stratum and treatment group as covariates and will be presented together with associated the 95% CI.

The study will be considered positive, if the p-value from the exact logistic regression is significant (p<0.01) and the lower bound of the 95% CI of the difference between active treatment and placebo response rates is higher or equal to 15%.

Sensitivity analyses of primary efficacy endpoint:

The primary efficacy endpoint analysis will be repeated on the FAS and PP population. Besides, a sensitivity analysis with the region (Australia/Europe/North America) and the interaction between regions and treatment group as covariate will be performed. In addition, sensitivity analyses of the primary efficacy endpoint will be conducted on the overall ITT population using multiple imputation and worst-case imputation instead of LOCF imputation to assess the robustness of the primary efficacy analyses with regard to handling of missing data.

Secondary efficacy analysis:

<u>Comparison of the percentage of treatment responders in each screening ED stratum</u>:

Within each screening ED stratum, the treatment group comparison is conducted on the ITT population at a 2-sided 5% significance level using LOCF imputation and the exact logistic model described in the above section. The corresponding p-value, as well as the number and percentage of responders (95% CI), will be presented by treatment group. The difference between active treatment and placebo response rates and the corresponding 95% CI will be presented. The clinical relevance of the treatment effect in each stratum will be evaluated based on the lower bound of the 95% CI strictly higher than 0%. The relative risk of achieving a response in the active treatment group compared to the placebo group within each stratum will be assessed using the log-binomial model described above and will be presented together with the associated 95% CI.

This analysis will be repeated on the FAS and PP population. Besides, a sensitivity analysis with the region (Australia/Europe/North America) and the interaction between region and treatment group as covariate will be performed.

<u>Comparison of the percentage of treatment responders in each of the 2 age subgroups:</u>

Within each age subgroup (4 to 5 years-old; 6 to 11 years-old), the treatment group comparison is conducted on the ITT population at a 2-sided 5% significance level using LOCF imputation and the exact logistic model described above. The corresponding p-value, as well as the number and percentage of responders (95% CI), will be presented by treatment group. The difference between active treatment and placebo response rates and the corresponding 95% CI will be presented. The relative risk of achieving a response in the active treatment group compared to the placebo group within each subgroup will be assessed using the log-binomial model described above and will be presented together with the associated 95% CI.

This analysis will be repeated on the FAS and PP population.

<u>Cumulative reactive dose and Eliciting dose at Month 12</u>:

The peanut protein cumulative reactive dose at Month 12 and the peanut protein ED at Month 12 will be summarized descriptively by treatment group (for the whole population and for each screening ED stratum), for the ITT population using LOCF imputation, as well as for the FAS and PP population.

In addition, the peanut protein cumulative reactive dose and the ED in each treatment group at Month 12 will be compared using an analysis of covariance (ANCOVA) model. The ANCOVA model will include the treatment group, baseline value, screening ED stratum and region

(Australia/Europe/North America) as covariates. Results will be log-transformed if needed. The peanut protein cumulative reactive dose and the ED will be evaluated for the ITT population using LOCF imputation, as well as for the FAS and PP population, overall and for each screening ED stratum, at a 2-sided 5% significance level.

Subjects responsive/unresponsive to a cumulative dose ≥1,444 mg peanut protein and subjects passing the DBPCFC at Month 12:

The percentage of subjects responsive to a cumulative dose ≥1,444 mg peanut protein at Month 12, the percentage of subjects unresponsive to a cumulative dose ≥1,444 mg peanut protein at Month 12, and the percentage of subjects passing the DBPCFC at Month 12 will be summarized descriptively by treatment group for the whole population and for each screening ED stratum, for the ITT population using LOCF imputation, as well as for the FAS and PP population.

In addition, the percentage of subjects responsive to a cumulative dose ≥1,444 mg peanut protein at Month 12, the percentage of subjects unresponsive to a cumulative dose ≥1,444 mg peanut protein at Month 12 and the percentage of subjects passing the DBPCFC in each treatment group at Month 12 will be compared using an exact logistic regression (adjusting for screening ED stratum and including the treatment group as fixed effect) for the overall ITT population using LOCF imputation, as well as for the FAS and PP population, at a 2-sided 5% significance level.

Other efficacy analyses:

Immunological markers:

The change in peanut-specific IgE and IgG4 from baseline to Months 3, 6 and 12 will be tabulated by treatment group, in the overall ITT population and for each screening ED stratum, using observed data. In addition, the peanut-specific IgE and IgG4 values in each treatment group at Month 12 will be compared, in the overall ITT population and for each screening ED stratum, using an ANCOVA model. The ANCOVA model will include the treatment group, baseline value, screening ED stratum and region (Australia/Europe/North America) as covariates.

Skin prick test:

The change in skin prick test results from baseline to Months 3, 6 and 12 will be tabulated by treatment group in the overall ITT using observed data. In addition, the skin prick test results in each treatment group at Month 12 will be compared, in the overall ITT population, using an ANCOVA model (same model as described above).

Quality of life:

The change in the Quality of Life score between baseline and Month 12 will be summarized in the overall ITT population using observed data.

Multiplicity issues:

In order to handle the multiple comparisons versus placebo, the overall type-I error will be controlled by the use of a hierarchical inferential approach, which means a pre-defined hierarchical order will be followed for the primary and secondary efficacy endpoints. No further adjustments will be made for the other efficacy endpoints for which p-values will be provided for descriptive purpose only.

Sample size calculations:

The sample size is calculated based on the following assumptions:

- A 40% response rate for the active Viaskin® Peanut 250 µg group and 10% response rate for the placebo group in the overall population, with a 2-sided type-I error rate α set to 1%;
- A lower bound of the 95% CI of the difference between active

treatment and placebo response rates ≥15% in the overall population and >0% in each stratum;

- A 60% response rate for the active Viaskin® Peanut 250 µg group and 10% response rate for the placebo group in Stratum 1, with a 2-sided type-I error rate α set to 5%;
- A 30% response rate for the active Viaskin® Peanut 250 µg group and 10% response rate for the placebo group in Stratum 2, with a 2-sided type-I error rate α set to 5% and a power (1-β) set to 85%;
- The following expected distribution of the screening ED strata:
  - 1/3 of children having a screening ED from 1 mg to 10 mg;
  - 2/3 of children having a screening ED from 30 mg to 300 mg.
- A ratio 2:1 to maximize the number of subjects treated in the active Viaskin® Peanut 250 µg group.

Therefore, the sample size calculated for Stratum 2 is 185 subjects (approximately 123 subjects in the active Viaskin® Peanut 250 µg group and 62 subjects in the placebo group). Hence, if 185 children with a screening ED from 30 mg to 300 mg are required in the study, then, according to the expected distribution of the screening ED strata, approximately 95 children having a screening ED from 1 mg to 10 mg should be randomized, resulting in a total of 280 subjects evaluable for the primary efficacy endpoint in the analysis.

This number of subjects leads to a power of 99% in the overall population and in the Stratum 1.

This is summarized in the following Table:

Summary of Sample Size Calculations

**Population**	**alpha**	**Power**	**Sample size (N=N1+N2) Expected Response Rate**			**Lower bound of the 95% CI of the difference**
			Viaskin® Peanut 250 µg	Placebo	Total	
Screening ED Stratum 1	5%	99%	N1=63 60%	N2=32 10%	N=95	>0%
Screening ED Stratum 2	5%	85%	N1=123 30%	N2=62 10%	N=185	>0%
Overall population	1%	99%	N1=186 40%	N2=94 10%	N=280	≥15%

Abbreviations: CI = Confidence interval; ED = Eliciting dose; N = Number of subjects.

To allow for a potential premature withdrawal rate of about 15%, the study will randomize a total of 330 subjects: 220 subjects will be randomized to receive Viaskin® Peanut 250 µg and 110 subjects to placebo. Among the 330 subjects randomized, a minimum of 25 subjects will be randomized for each of the following ages: 4 years, 5 years and 11 years, respectively. Besides, a minimum number of 75 children of either 4 or 5 years of age must be randomized.

The distribution per ED stratum should lead to approximately 110 subjects with a screening ED from 1 mg to 10 mg and approximately 220 subjects with a screening ED from 30 mg to 300 mg, provided that the same distribution as obtained in the Phase IIb VIPES study is reproduced. If this expected distribution is not fully respected during the recruitment in the

PEPITES study, a minimum of 50 subjects would need to be included in Stratum 1 in order to guarantee a power of ≥90% in this stratum and at least 220 subjects would need to be included in Stratum 2 in order to guarantee a power of ≥85% in this stratum.

From experience, the screen failure rate could be as high as 30%; hence, up to 470 peanut allergic subjects might need to be screened.

LIST OF STUDY PERSONNEL

**Sponsor**	Wence AGBOTOUNOU, PhD, MBA<br>Chief Clinical Trial Officer<br>DBV Technologies S.A.<br>Green Square – Bâtiment D<br>80/84, rue des Meuniers<br>92220 Bagneux, France<br>Tel: + 33 1 55 42 78 74<br>E-mail: wence.agbotounou@dbv-technologies.com
	Laurent MARTIN, PharmD<br>Director, Regulatory Affairs & Quality<br>DBV Technologies S.A. (same address as above)<br>Tel: + 33 1 55 42 78 94<br>E-mail: laurent.martin@dbv-technologies.com
	Claude THEBAULT, MD<br>Medical Director<br>DBV Technologies S.A. (same address as above)<br>Tel: + 33 1 84 16 29 07<br>E-mail: claude.thebault@dbv-technologies.com
	Aurélie PEILLON<br>Biostatistician<br>DBV Technologies S.A. (same address as above)<br>Tel: + 33 1 84 16 29 44<br>E-mail: aurelie.peillon@dbv-technologies.com
**Contract Research Organization**	PAREXEL International (IRL) Limited (“PAREXEL”)<br>70 Sir John Rogerson’s Quay<br>Dublin 2<br>Ireland
**Central Laboratory**	Q^2 Solutions Limited<br>The Alba Campus<br>Rosebank, Livingston<br>EH54 7EG<br>Scotland

**Drug Manufacturing Company**	AMATSI<br>17 Parc des Vautes<br>F-34980 Saint Gely du Fesc (Montpellier)<br>France<br>Tel: + 33 4 99 58 38 60<br>FAX: + 33 4 99 58 38 61
**Drug Supply/Packaging Company**	CREAPHARM<br>Z.A. Air-Space<br>Avenue de Magudas<br>CS 2007<br>33187 Le Haillan (Bordeaux)<br>France<br>E-mail: dbv-technologies@creapharm.com

TABLE OF CONTENTS

PROTOCOL SYNOPSIS **4**

LIST OF STUDY PERSONNEL **14**

LIST OF ABBREVIATIONS AND DEFINITIONS OF TERMS **22**

1 INTRODUCTION **24**

1.1 Background **24**
1.1.1 Summary of Findings from Non-clinical and Clinical Studies 25
1.1.1.1 Non-clinical Studies *25*
1.1.1.2 Clinical Studies *26*
1.2 Rationale **35**
1.3 Risk-Benefit Assessment **35**

2 STUDY OBJECTIVES **37**

3 OVERALL DESIGN AND PLAN OF THE STUDY **38**

3.1 Overview **38**
3.2 Criteria for Evaluation of the Study **40**
3.2.1 Efficacy Criteria 40
3.2.1.1 Primary Efficacy Endpoint *40*
3.2.1.2 Secondary Efficacy Endpoint *40*
3.2.1.3 Other Efficacy Endpoints *41*
3.2.2 Safety Criteria 41
3.2.3 Exploratory Criteria 42
3.3 Justification of the Study Design **43**

4 STUDY POPULATION **44**

4.1 Inclusion Criteria **44**
4.2 Exclusion Criteria **45**
4.3 Patient Withdrawal and Replacement **47**
4.3.1 Criteria for Withdrawal from Study Treatment and Study 47
4.3.2 Study Stopping Rules 48
4.3.3 Replacement of Withdrawn Subjects 48
4.3.4 Data Collection and Follow-up after Withdrawal 48
4.4 Planned Sample Size and Number of Study Centers **48**
4.5 Patient Identification and Randomization **49**
4.5.1 Patient Identification 49
4.5.2 Randomization Scheme 49
4.5.3 Allocation/Randomization of Patients to Treatment 49

5 STUDY DRUG **50**

5.1 Identity **50**
5.2 Administration **50**
5.2.1 Adjustment of Viaskin® Patch Application in Case of Local or Systemic Reactions 52
5.2.2 Safety Precaution Information 53

5.3 Packaging, Labeling and Storage 53
5.4 Blinding and Breaking the Blind 54
5.5 Drug Accountability 55
5.6 Compliance 55
5.7 Viaskin® Patch Adhesion 55
5.8 Prior and Concomitant Medication 56
5.8.1 Permitted Concomitant Medication 56
5.8.2 Prohibited Prior and Concomitant Medication 57

6 VARIABLES AND METHODS OF ASSESSMENT 58

6.1 Efficacy Variables 58
6.1.1 Response to Treatment – DBPCFC to Peanut 58
6.1.1.1 Preparation of Peanut and Placebo Formulas 59
6.1.1.2 Time Interval and Doses 59
6.1.1.3 Entry/screening DBPCFC to Peanut 59
6.1.1.4 Post-treatment DBPCFC to Peanut at Month 12 62
6.2 Safety Variables 63
6.2.1 Adverse Events 63
6.2.1.1 Collection of Adverse Events 63
6.2.1.2 Definitions 63
6.2.1.3 Assessment of Adverse Events 63
6.2.1.4 Recording Adverse Events 67
6.2.1.5 Reporting Serious Adverse Events 67
6.2.1.6 Follow-up of Adverse Events 68
6.2.1.7 Pregnancy 68
6.2.1.8 Treatment of Overdose of Study Medication 68
6.2.2 Laboratory Variables 69
6.2.3 Vital Signs 69
6.2.4 Physical Examinations 70
6.2.5 Spirometry Test 70
6.2.6 Peak Expiratory Flow 70
6.2.7 Subject Diaries 71
6.2.8 Skin Reaction and Photography 71
6.2.9 Viaskin®Patch Adhesion 72
6.3 Demographics and Baseline Characteristics 73
6.3.1 Patient Demography 74
6.3.2 Disease History and Medical History 74
6.3.3 Prior and Concomitant Medications 74
6.4 Exploratory Variables 74
6.4.1 Immunological Markers 74
6.4.2 Skin Prick Test 74
6.4.3 Food Allergy Quality of Life Questionnaire /Food Allergy Independent Measure 75
6.4.4 Accidental Consumption of Peanuts 75
6.4.5 Epigenetic analyses 75
6.4.6 Genetic Screening 75
6.4.7 Scoring Atopic Dermatitis 76

7 STUDY CONDUCT ... 77

7.1 Schedule of Procedures ... 77

7.2 Procedures by Visit ... 82

7.2.1 Visit 1, Screening (Day -42) ... 82

7.2.2 Visit 2, Screening (through Day -2) ... 82

7.2.3 Visit 3, Screening (within 1 week of Visit 2 and through Day -1) ... 83

7.2.4 Visit 4 (Day 1), First Day of Treatment ... 84

7.2.5 Phone Contacts (Day 4±2 days and Day 22±2 days), Treatment Period ... 85

7.2.6 Visit 5 (Day 8±3 days), Visit 6 (Month 1±3 days) and Visit 9 (Month 9±7 days), Treatment Period ... 85

7.2.7 Phone Contacts (Month 2±3 days, Month 4.5±7 days, Month 7.5±7 days), Treatment Period ... 86

7.2.8 Visit 7 (Month 3±7 days) and Visit 8 (Month 6±7 days), Treatment Period ... 86

7.2.9 Visit 10 (Month 12±7 days), Treatment Period ... 87

7.2.10 Visit 11 (≤1 week after Visit 10), End of Treatment Period ... 89

7.2.11 Visit 12 (2 weeks after Visit 11), End of Study Period ... 90

7.2.12 Early Termination Visit ... 90

7.2.13 Unscheduled Visit ... 90

8 STATISTICAL METHODS ... 92

8.1 Study Patients ... 92

8.1.1 Disposition of Patients ... 92

8.1.2 Protocol Deviations ... 92

8.1.3 Analysis Sets ... 92

8.1.3.1 Safety Population ... 92

8.1.3.2 Intent-to-treat Population ... 92

8.1.3.3 Full analysis set ... 93

8.1.3.4 Per-protocol Population ... 93

8.2 General Considerations ... 93

8.2.1 Statistical Methods ... 93

8.2.2 Analysis and Data Conventions ... 93

8.2.2.1 Definition of Baseline ... 93

8.2.2.2 Visit Windows ... 93

8.2.2.3 Unscheduled Assessments ... 93

8.2.2.4 Missing Data Conventions ... 94

8.2.2.5 Pooling of Centers ... 94

8.3 Demographics, Disease and Medical Histories, Baseline Characteristics, and Concomitant Medications ... 95

8.4 Treatment Compliance and Exposure ... 95

8.5 Efficacy Analyses ... 95

8.5.1 Primary Efficacy Analysis ... 95

8.5.1.1 Hypothesis to be tested ... 95

8.5.1.2 Statistical Methods ... 96

8.5.1.3 Sensitivity Analyses ... 96

8.5.2 Secondary Efficacy Analyses 96
8.5.2.1 Percentage of treatment responders in each screening ED stratum 96
8.5.2.2 Percentage of treatment responders in each age subgroup....97
8.5.2.3 Mean and Median Cumulative Reactive/Eliciting Doses 97
8.5.2.4 Responsive/Unresponsive Subjects, Subjects Passing DBPCFC....97
8.5.3 Pre-defined Hierarchical Order for the Analysis of Efficacy Endpoints 98
8.5.4 Other Efficacy Analyses 99
8.6 Safety Analyses 99
8.6.1 Adverse Events 99
8.6.2 Laboratory Assessments 100
8.6.3 Vital Signs....100
8.6.4 Physical Examination....101
8.6.5 Spirometry and Peak Expiratory Flow Results....101
8.6.6 Subject Diaries....101
8.6.7 Skin Reactions 102
8.6.8 Symptomatic Reactions During the DBPCFC....102
8.7 Exploratory Analyses....102
8.8 Patch adhesion....103
8.9 Interim Analyses....103
8.10 Determination of Sample Size 103

9 ETHICAL, LEGAL, AND ADMINISTRATIVE ASPECTS 106

9.1 Data Quality Assurance....106
9.1.1 Database Management and Quality Control....106
9.2 Case Report Forms and Source Documentation....106
9.2.1 Data Collection 107
9.3 Access to Source Data 107
9.3.1 Routine Monitoring....107
9.3.2 Inspections and Auditing Procedures....107
9.4 Data Processing 108
9.5 Archiving Study Records....108
9.6 Good Clinical Practice 109
9.7 Informed Consent....109
9.8 Protocol Approval and Amendment....110
9.9 Data and Safety Monitoring Board 110
9.10 Duration of the Study....111
9.11 Premature Termination of the Study 111
9.12 Confidentiality....111
9.13 Contractual and Financial Details....112
9.14 Liability and Insurance....112
9.15 Publication Policy....112
9.16 Study Center File Management....112
9.17 Clinical Study Report 113

10 REFERENCE LIST **114**

APPENDICES **117**

APPENDIX 1 .... **118**

APPENDIX 2 .... **125**

APPENDIX 3 .... **127**

APPENDIX 4 .... **128**

APPENDIX 5 .... **129**

APPENDIX 6 .... **130**

APPENDIX 7 .... **132**

APPENDIX 8 .... **142**

Tables in Text

Table 1: Incidence of Dosing Reactions Per Age Group in the CoFAR6 Study 30
Table 2: Summary of Occurrence of Dosing Symptoms Per Applied Dose and Per Age Group in the CoFAR6 Study 30
Table 3: Summary of Viaskin® Peanut-related Adverse Events in the VIPES Study in Children (6- to 11-Years-Old) 32
Table 4: Time of Occurrence of Viaskin® Peanut-related Treatment-Emergent Adverse Events in the VIPES Study in Children (6- to 11-Years Old) 33
Table 5: Safety Data of Viaskin® Peanut 250 µg According to Age Group in the VIPES Study in Children (6- to 11-Years-Old) 33
Table 6: Skin Reaction Grading System 72
Table 7: Schedule of Procedures 78
Table 8: Pre-defined Hierarchical Order for Analysis of Efficacy Endpoints 98
Table 9: Criteria to assess Clinically Relevant Abnormalities in Vital Signs 101
Table 10: Summary of Sample Size Calculation 104

Figures in Text

Figure 1: Study Design 40
Figure 2: Schematic Representation of Viaskin® Patch Application on the Back of the Subjects 51

List of Appendices

Appendix 1 Declaration of Helsinki

Appendix 2 Dosages of Inhaled Corticosteroids

Appendix 3 Activity of Corticosteroids

Appendix 4 Short-acting and Long-acting Antihistamines based on Terminal Elimination Half-Lives

Appendix 5 Anaphylaxis Staging System

Appendix 6 Oral Food Challenge Symptom Score Sheet

Appendix 7 FAQLQ/FAIM Questionnaires

Appendix 8 SCORAD

LIST OF ABBREVIATIONS AND DEFINITIONS OF TERMS

AE	Adverse Event
AESI	Adverse Event of Special Interest
ANCOVA	ANalysis of COVAriance
APC	Antigen-Presenting Cells
AP-HP	Assistance Publique-Hôpitaux de Paris.
ATC	Anatomical Therapeutic Chemical (Classification System)
ATS	American Thoracic Society
CI	Confidence Interval
CoFAR	Consortium of Food Allergy Research
CSR	Clinical Study Report
CTCAE	Common Terminology Criteria for Adverse Events
DBPCFC	Double-Blind, Placebo-Controlled Food Challenge
DSMB	Data and Safety Monitoring Board
EAACI	European Academy of Allergy and Clinical Immunology
e-CRF	Electronic Case Report Form
ED	Eliciting Dose
EDC	Electronic Data Capture
EPIT	EPicutaneous ImmunoTherapy
FAQLQ/FAIM	Food Allergy Quality of Life Questionnaire/Food Allergy Independent Measure
FAS	Full Analysis Set
FEV_1	Forced Expiratory Volume in 1 second
FDA	Food and Drug Administration
GA^2LEN	Global Allergy and Asthma European Network
GCP	Good Clinical Practice
IB	Investigator's Brochure
ICF	Informed Consent Form
ICH	International Conference on Harmonization
IEC	Independent Ethics Committee
IgE, IgG, IgG4	Immunoglobulin E, Immunoglobulin G, Immunoglobulin G4 subtype
IRB	Institutional Review Board

ITT	Intent-To-Treat
IV	Intravenous
IWRS	Interactive Web Response System
LEAP	Learning Early About Peanut Allergy
LOCF	Last Observation Carried Forward
MedDRA	Medical Dictionary for Regulatory Activities
NIH	National Institute of Health
OFC	Oral Food Challenge
OIT	Oral ImmunoTherapy
PEF	Peak Expiratory Flow
PP	Per-Protocol
PT	Preferred Term
SAE	Serious Adverse Event
SAP	Statistical Analysis Plan
SCORAD	SCORing Atopic Dermatitis
SLIT	SubLingual ImmunoTherapy
SOC	System Organ Class
SPT	Skin Prick Test
TEAE	Treatment-Emergent Adverse Event
US(A)	United States (of America)
WHO	World Health Organization

1 INTRODUCTION

1.1 Background

Peanut allergy is the most common cause of fatal food allergy reactions (1,2). An estimated 1% of the population of the United States of America (USA), over 3 million people, is allergic to peanuts or tree nuts (3). The prevalence of peanut allergy in children has been increasing over the last 2 decades, as indicated by surveys conducted in the USA and United Kingdom: the rate of peanut allergy in children doubled within a range of 5 to 6 years (4,5) and more than tripled between 1997 and 2008 with a prevalence of 0.4% in 1997, 0.8% in 2002 and 1.4% in 2008 (3). Studies indicate that peanut allergy might resolve in about 20% of cases (6,7,8,9), but may recur in some desensitized individuals, making this allergy a life-long affliction in the vast majority of cases.

A recent randomized study of peanut consumption in infants at high risk of developing peanut allergy, the Learning Early About Peanut Allergy (LEAP) study, has evaluated the strategies of peanut consumption or avoidance at very early ages, between 4 and 11 months of age (10). In the subset of infants at high risk to develop peanut allergy, which were infants with eczema, positive egg skin prick test (SPT), negative or slightly positive peanut SPT, the results of this prospective study demonstrated that early (during infancy) peanut consumption might be preferable to avoidance of peanut consumption for preventing the occurrence of peanut allergy in infants at high risk.

Peanut allergy falls within the Immunoglobulin E (IgE)-mediated category of food allergies, with immediate reactions triggered by circulating allergen-specific IgE upon exposure to the allergen (11). IgE-mediated allergic reactions to foods have a rapid onset, usually within a few minutes following exposure to the allergen. IgE-mediated allergic reactions to peanut provoke characteristic responses in the skin, gastrointestinal tract, upper and lower respiratory tract, and cardiovascular system (12). IgE-mediated reactions to food may also trigger generalized reactions, that is anaphylaxis, a severe, potentially fatal, systemic allergic reaction that occurs suddenly after contact with an allergy-causing substance (13).

The complete mechanism of IgE-mediated food allergy remains unknown. However, it is thought that the development of an IgE-mediated response to an allergen is the result of a series of molecular and cellular interactions involving Antigen-Presenting Cells (APCs), T cells and B cells (14,15). Upon re-exposure to the sensitizing food, the allergen crosslinks the specific IgEs bound on mast cells and basophils, triggering release of vasoactive and inflammatory mediators such as histamine, leukotrienes, prostaglandins, and platelet-activating factor. The massive release of these mediators induces immediate allergic systemic symptoms, including anaphylaxis.

There are no approved treatments available for peanut allergy (16). Currently, the only therapeutic option available for peanut-allergic subjects is strict avoidance. However, since peanut is a ubiquitous ingredient in many foods, strict avoidance is difficult to achieve, and accidental ingestion of peanut by peanut-allergic subjects may result in severe reactions and fatal outcomes (17).

The only available countermeasure in case of severe systemic and/or life-threatening reactions/anaphylaxis to peanuts is injectable epinephrine as recommended by the World

Allergy Organization (18). Epinephrine remains a rescue therapeutic agent and is not designed for a routine use.

Various non-specific and food allergen-specific treatment approaches have been under evaluation. Non-specific approaches to food allergy include the use of monoclonal anti-IgE antibodies, which might increase the Eliciting Dose (ED) threshold for the food allergen (19,20). Food allergen-specific approaches in clinical development include Oral ImmunoTherapy (OIT), SubLingual ImmunoTherapy (SLIT), and EPicutaneous ImmunoTherapy (EPIT) (21). Food-specific approaches may be advantageous as they target the specific foods that cause the severe IgE-mediated anaphylactic reactions (22). Studies on SLIT and OIT have demonstrated some encouraging efficacy results (clinical desensitization), including beneficial immunologic changes (23,24,25,26). Oral immunotherapy has shown evidence for inducing desensitization in most subjects, with immunologic changes over time (25,26,27). These advances are, however, hampered by the significant risk of side effects and occurrence of eosinophilic esophagitis in the context of OIT (28,29,30,31,32). Sublingual immunotherapy for peanut allergy has demonstrated evidence of clinical success, with some subjects showing signs of desensitization with a more satisfactory side effect profile compared to OIT and with significant immunologic changes noted during the first year of therapy (24). Despite the evident interest of clinicians to further evaluate these treatment procedures, OIT and SLIT will not likely be applicable across all ages and risk categories of peanut-allergic children and adults. Alternative immunotherapeutic approaches for peanut allergy are therefore needed, with a clinically meaningful benefit.

The study drug, Viaskin® Peanut (or DBV712), consists of an epicutaneous delivery system (Viaskin® patch) containing a dry deposit of a formulation of peanut protein extract. The peanut protein allergens are deposited on the backing of an occlusive chamber by electrospraying a liquid formulation of the peanut protein extract. The drug substance is an unmodified, lyophilized peanut extract produced from the extraction and freeze drying of defatted peanut flour, derived from the peanut seed, *Arachis hypogaea*. Further details can be found in the IB (33), which contains comprehensive information on the study drug.

The PEPITES study is a 12-month, Phase III, double-blind, placebo-controlled, randomized study to assess the efficacy and safety of Viaskin® Peanut, dosed at 250 µg peanut protein (per patch) in peanut-allergic children from 4 through 11 years of age. The objective of this study is to assess the efficacy and safety of Viaskin® Peanut 250 µg peanut protein to induce desensitization to peanut in peanut-allergic children 4 through 11 years of age after a 12-month treatment by EPIT.

1.1.1 Summary of Findings from Non-clinical and Clinical Studies

Further details on the studies summarized below can be found in the IB (33).

1.1.1.1 Non-clinical Studies

DBV Technologies has conducted a number of non-clinical studies supporting the clinical development of Viaskin® Peanut. These include ISO 10993-compliant biocompatibility studies performed for the device component independent of the drug component, which is the Viaskin® patch, and non-clinical studies performed with the combined product, which includes *in vitro* pharmacokinetic/absorption studies, *in vivo*

pharmacology (efficacy) studies in a mouse model of peanut allergy, and Good Laboratory Practice-compliant toxicology studies in the rabbit and in a guinea pig model of peanut allergy.

1.1.1.2 Clinical Studies

1.1.1.2.1 Study PEP01.09: Phase Ib Safety (Completed)

This randomized, double-blind, placebo-controlled Phase Ib study was conducted to assess the safety and tolerability of Viaskin® Peanut administered epicutaneously to adults (≥18 years of age), adolescents (12 to 17 years of age), and children (6 to 11 years of age) with peanut allergy. A total of 100 subjects, 70 non-severe (without any history of severe anaphylactic reactions) and 30 severe (with a history of severe anaphylactic reactions), were randomized and treated with repeated doses of Viaskin® Peanut or placebo for 2 weeks. Eighty subjects received Viaskin® Peanut at the doses of 20 µg, 100 µg, 250 µg, or 500 µg of peanut protein per patch, and 20 subjects received placebo. Subjects applied 1 patch on the skin every 24 hours or every 48 hours. The patches were applied on the back for the children and on 1upper arm for the adolescents and adults.

Overall, Viaskin® Peanut was safe and well tolerated. No serious adverse events (SAEs) were reported in this study. The most commonly reported adverse events (AEs) were site pruritus, site erythema, site edema, or site urticaria. Viaskin® Peanut triggered more local reactions than placebo. Severe local AEs were reported in 11% of Viaskin® Peanut subjects; these mainly included pruritus (10% of subjects) and were generally transient. In such cases, the subject could remove the patch and allow for the AEs to subside. The maximal tolerated dose was established at 500 µg of peanut protein in adults (severe and non-severe), 500 µg of peanut protein in adolescents (non-severe), and 250 µg of peanut protein in children (non-severe).

1.1.1.2.2 ARACHILD Study: Phase II Pilot Efficacy and Safety Study (Completed)

This pilot double-blind, placebo-controlled Phase II study was sponsored by the largest French public hospital organization, Assistance Publique-Hôpitaux de Paris (AP-HP). The primary objective of this study was to demonstrate the efficacy of EPIT with Viaskin® Peanut to desensitize children 5 to 17 years of age with a documented peanut allergy. A double-blind, placebo-controlled food challenge (DBPCFC) was conducted at study entry and subjects reacting below a cumulative dose of 300 mg peanut protein were eligible. A total of 54 subjects were randomized at a 1:1 ratio to receive either Viaskin® Peanut 100 µg or placebo for a period of 6 months of blinded treatment. This was first followed by a 12-month open-label treatment period with Viaskin® Peanut 100 µg; the children who were in the placebo group during the first 6 months then crossed-over to also receive Viaskin® Peanut 100 µg for the remaining 12 months, up to Month 18. An amendment to the protocol further extended the open-label treatment period to an additional 18 months for subjects randomized to receive active Viaskin® Peanut treatment from the beginning, and to an additional 24 months for subjects randomized to the placebo group. A total of 30 subjects accepted to extend their study participation under this amendment.

The response rate in the ARACHILD study was defined as the percentage of subjects who reached a cumulative reactive dose of 1,000 mg peanut protein after EPIT treatment

or subjects able to multiply by 10 their cumulative reactive dose after treatment as compared to baseline.

This study is the first proof of concept pilot study to demonstrate safety and efficacy of EPIT using Viaskin® Peanut at a unique dose of 100 µg peanut protein. After 6 months of treatment during the blinded period, there was no statistical difference between the 2 treatment groups in the overall population (7.4% response in the active group versus 7.7% in the placebo group). However, after 12 months and 18 months of treatment, the overall response rate of the active group increased to 20% and 40%, respectively. Surprisingly, there were no responders among the 12- to 17-year-old adolescents. All responders were children (15) from 5 to 11 years of age. The response rate in these children was 5/15 (33.3%) and 10/15 (66.7%), respectively, at Month 12 and Month 18. Also, in the children (5- to 11-years-old) who received Viaskin® Peanut, the starting mean cumulative reactive dose at baseline was 23.14±29.31 mg which had progressed to reach 357.66±542.95 mg after 18 months.

In this study, there were 20 SAEs (18 cases). Five were considered related to study procedure, occurring during the DBPCFC: 3 anaphylactic reactions, 1 cutaneous and digestive anaphylaxis, and 1 bronchospasm. All of these events resolved within 24 hours. Four SAEs were considered possibly related to study drug by the Investigators: 1 herpetic gingivostomatitis, 1 pilonidal abscess on a probable pilonidal cyst, 1 recurrence of this pilonidal cyst, and 1 anaphylactic reaction after eating a “kebab” sandwich. In the Viaskin® Peanut group, there was 1 doubtful event of eye pruritus by contact after patch removal in 1 adolescent and 1 edema of the upper lip after patch application in 1 child.

Viaskin® Peanut 100 µg demonstrated an overall satisfactory safety profile. During the double-blind period of the study, 11.5% severe AEs occurred in the placebo group versus 7.1% in the Viaskin® Peanut group with mostly skin disorders (none severe), gastrointestinal disorders (2 severe AEs of abdominal pain in the Viaskin® Peanut group), and respiratory disorders (none severe) as compared to placebo. The safety profile of Viaskin® Peanut was compatible with a daily, long term application.

1.1.1.2.3 VIPES Study: Phase IIb Efficacy and Safety Study (Completed)

This Phase IIb, double-blind, placebo-controlled study assessed the safety and efficacy of EPIT with Viaskin® Peanut in 221 peanut-allergic subjects (113 children, 73 adolescents and 35 adults) who were randomized to receive Viaskin® Peanut 50 µg, 100 µg, 250 µg, or placebo for 12 months.

With regard to efficacy, the primary efficacy endpoint was met. The treatment response rate in the whole population at Month 12 showed a statistically significant higher responder rate in the Viaskin® Peanut 250 µg group with 28 (50.0%) responders compared to 14 (25.0%) responders in the placebo group (p-value = 0.0108). The study population was composed of 2 age strata: children (6 to 11 years of age) and adolescents and adults (12 years and above). Fifteen (53.6%) children (6- to 11-years-old) were responders in the Viaskin® Peanut 250 µg group at Month 12 compared to 6 (19.4%) children in the placebo group (p-value = 0.0076). For the main secondary efficacy endpoints analyzed, there was a better response with the highest dose of Viaskin® Peanut (250 µg) than with the lower doses (50 µg and 100 µg) compared to placebo. The results for the children (6- to 11-years-old) were very favorable in all analyses, as differences

compared to placebo were generally even more pronounced with better statistical significance than the results seen in the whole population. In the adolescent and adult age strata, even though Viaskin® Peanut 250 µg showed higher effects for all endpoints analyzed, these were not statistically significant.

Generally, in terms of safety, Viaskin® Peanut patches at 50 µg, 100 µg or 250 µg peanut protein were well tolerated. The most common and most frequent AEs reported by the subjects were local cutaneous reactions at the sites of the patch application in more than 90% of the subjects treated with any dose of Viaskin® Peanut, versus 50% of the subjects in placebo. These local reactions were generally of mild or moderate severity, and resulted in withdrawal from treatment in only 2/221 (0.9%) of subjects.

There were no clinical differences for Viaskin® Peanut-related AEs between the 3 active Viaskin® Peanut groups. There were clear differences between the 3 active Viaskin® Peanut groups and the placebo group for these local cutaneous AEs (pruritus/itching, erythema/redness, edema/swelling) at sites of patch application as auto-evaluated on a daily basis by the subjects over the first 3 months of EPIT and as assessed by the Investigators over the 12 months of EPIT. One case of grade 4 local skin reaction (on a scale of 0 = negative to 4 = erythema, vesicles) of erythema with 2-3 vesicles was reported by 1 subject without resulting in subject withdrawal. At all time-points, a higher percentage of subjects in any of the 3 active Viaskin® Peanut groups was assessed as having skin reactions of higher severity than the subjects in the placebo group. Over the course of the study, skin reactions of grades 1 to 3 appeared during the first month of treatment as the duration of the patch application gradually increased to 24 hours daily. For 50% of the subjects, the local skin reactions lasted less than 2 months and in the other half of subjects the local skin reactions mostly declined over time up to Month 9 or Month 12. Generalized itching after patch removal occurred in 1 child in the Viaskin® Peanut 250 µg group.

There were no SAEs related to Viaskin® Peanut in the VIPES study. Of the 20 SAEs that occurred, 14 SAEs were related to the study procedure of DBPCFC, with subjects reporting anaphylactic reactions that required prolonged hospitalization overnight.

1.1.1.2.4 OLFUS-VIPES Study: Open-label Follow-up Study of the VIPES Study (Ongoing)

This study evaluates the long-term efficacy and safety of Viaskin® Peanut in children, adolescents and adults. Subjects previously randomized in the VIPES study and who completed the study were offered to receive the Viaskin® Peanut 250 µg treatment for 24 additional months. From the 207 subjects who completed the VIPES study up to Month 12, 171 (83%) have rolled over into the OLFUS-VIPES study. As of 31 May 2015, 20 subjects (11.7%) have withdrawn from the study and 151 subjects were still under treatment. Eight SAEs were reported, none of which were related to Viaskin® Peanut and none of which were related to the conduct of the food challenges.

1.1.1.2.5 CoFAR6 Study: Phase II Efficacy and Safety Study (Ongoing)

This is a randomized, double-blind, placebo-controlled, Phase II study in children, adolescents and adults sponsored by the National Institute of Health (NIH) and conducted in the USA by the Consortium of Food Allergy Research (CoFAR). The primary objective of this study is to assess the safety and efficacy of EPIT with Viaskin®

Peanut in peanut-allergic subjects. In parallel, immunological effects using a set of immune-focused mechanistic studies as well as cellular modifications induced by EPIT are studied. A total of 75 subjects aged 4 to 25 years (including 10 children aged 4 to 5 years) have been randomized in this study to be treated with either Viaskin® Peanut 100 µg or 250 µg or placebo (ratio 1:1:1). After their first year of blinded treatment in the CoFAR6 study, subjects enter the open-label phase of the study and receive treatment with the 250 µg dose for either 18 additional months for those under active treatment during the blinded phase of the study or for 30 months for those who were under placebo. As of 13 May 2015, an estimated minimum of 60 subjects completed the double-blind period and all subjects rolled over to receive the active treatment with Viaskin® Peanut 250 µg for a total of 30 months.

Six randomized subjects (2 children, 3 adolescents and 1 adult) have withdrawn prematurely from the study or discontinued dosing; 1 adolescent due to local skin reactions (experience of several days of grade 3 skin reactions with erythema extending beyond the patch application site and one grade 4 reaction, i.e. erythema with vesicles), 1 adolescent due to an unrelated SAE (syncopal episodes) and 4 subjects due to other circumstances. The 6-year-old child withdrew after 1 week of patch treatment because of expressed anxiety surrounding future food challenges associated to the protocol combined to a strong aversion to the peanut taste, and the 8-year-old child withdrew even before any patch treatment because of unforeseen family circumstances. No drop-out occurred in the children 4 to 5 years of age.

Three SAEs were reported (1 child and 2 adolescents), none had a causal relationship with the study product. No SAEs were reported in the population of 4- to 5-year-old children. No subject has died while on study.

In this study, skin reactions at the site of patch application were graded on a scale from grade 0 (negative according to clinical assessment; normal skin, no reaction, according to subject assessment) to grade 4 (erythema, vesicles, according to clinical assessment; redness with blisters, according to subject assessment). The symptoms outside the patch site (in a separate area from the patch site, not those that extended beyond the patch site) included assessment of skin reactions, gastrointestinal reactions and respiratory reactions which were scored in severity as mild, moderate or severe.

In January 2014, grading of skin reactions at site of patch application showed that out of the first 366 doses administered, there were 56% with no reactions, 23.3% grade 1, 18.6% grade 2 and 1.9% grade 3 reactions. One dose resulted in the appearance of vesicles at site of patch application which resulted in the subject's permanent discontinuation from treatment and from the study. Also, there were doses (≤5%) resulting in reactions beyond the size of the patch or distant from the patch site, including hives, redness, and pruritus. Of note, 1 of these doses resulting in reactions beyond the patch was an urticaria lesion which extended largely and covered the upper right quartile of the subject's back.

Safety data gathered for 4- to 5-year old subjects in the CoFAR6 study:

A specific safety assessment for the 4- to 5-year-old subjects was made available for the study period up to 25 May 2014. The incidence of dosing reactions for all subjects by age category is presented in Table 1 and the occurrence of dosing symptoms per applied doses is summarized in Table 2. Six subjects, all in the ≥6-years-old age group, who did

not have any dosing data as of the database closure on 25 May 2014, were not included in the analysis.

Table 1: Incidence of Dosing Reactions Per Age Group in the CoFAR6 Study

Type of Dosing Reaction	4 to <6 years	≥6 years
Subjects with no dosing reaction (%)	10.0	12.2
Subjects with patch site reaction only (%)	40.0	67.3
Subjects with patch site and non-patch site reaction (%)	50.0	20.4
Subjects with grade 3 patch site reaction (%)	20.0	8.2
Subjects with grade 4 patch site reaction (%)	0.0	2.0

Table 2: Summary of Occurrence of Dosing Symptoms Per Applied Dose and Per Age Group in the CoFAR6 Study

Dosing Symptoms	4 to <6 years	≥6 years
Total doses reported (n)	1,028	2,751
Any symptoms (%)	70.4	59.7
Patch site reactions (%)	70.2	59.5
Grade 2 patch site reactions (%)	26.0	15.4
Grade 3 patch site reactions (%)	0.3	0.2
Grade 4 patch site reactions (%)	0.0	0.04
Reactions extending beyond the patch site (%)	6.9	6.1
Grade 2 reactions extending beyond the patch site (%)	0.2	1.8
Grade 3 reactions extending beyond the patch site (%)	0.1	0.0
Non-patch site reactions (%)	1.2	0.8
Non-patch site reactions with mild symptoms (%)	1.0	0.7
Non-patch site reaction with moderate symptoms (%)	0.1	0.0
Symptoms lasting >8 hours (%)	64.3	38.5
Treatment was administered (%)	23.4	23.4
Treated with topical steroids (%)	16.6	12.2
Treated with oral antihistamines (%)	5.4	5.5

There were no non-patch site reactions with severe symptoms and there were no doses that resulted in treatment with epinephrine. A total of 87 doses were not taken (13 doses in the 4- to <6-years-old group, and 74 doses in the ≥6-years-old group).

In conclusion, as of 13 May 2015, no drop-outs and no SAEs occurred in the 10 children 4 to 5 years of age, all randomized for more than 12 months in the CoFAR6 study. The comparative safety assessment available for the study period up to 25 May 2014 showed no specific safety concerns in this study population compared to ≥6-year-old subjects. A similar percentage of subjects in the age group 4 to 5 years and the age group ≥6 years reported dosing reactions (90% versus 87.8%, respectively). Even though more 4- to 5 year- old children experienced non-patch site reactions (50% versus 20.4%), none of these reactions were severe. Occurrence of patch site reactions per applied doses was slightly higher in the age group 4 to 5 years (70.2% versus 59.5%) but these were limited to local reactions up to grade 3 (only 1 adolescent subject had a severe grade 4 local

reaction leading to study discontinuation). Furthermore, similar medications were administered across age groups to treat these AEs.

1.1.1.2.6 Clinically Relevant Adverse Events Related to Viaskin® Peanut

Safety data are summarized in tabular format in the IB for all Phase I to II trials conducted so far with Viaskin® Peanut (33). To date, Viaskin® Peanut, regardless of the dose or the age of the subjects, was investigated in 4 randomized controlled trials and in 1 open-label follow-up study (see Section 1.1.1.2.1 to Section 1.1.1.2.5) in more than 400 subjects with peanut allergy.

Safety data of the VIPES study with 113 Viaskin® Peanut-treated children are available. The AEs considered related to Viaskin® Peanut by the Investigators are summarized in Table 3.

Table 3: Summary of Viaskin® Peanut-related Adverse Events in the VIPES Study in Children (6- to 11-Years-Old)

MedDRA SOC	AEs – PTs
**Eyes disorders**	Eye swelling (common)<br>Eyelid oedema (common)<br>Conjunctivitis (uncommon)<br>Eye pruritus (uncommon)
**Gastrointestinal disorders**	Abdominal pain upper (common)<br>Constipation (uncommon)<br>Diarrhea (uncommon)
**General disorders and administration site conditions**	Application site erythema (common)<br>Application site pruritus (common)<br>Application site swelling (common)<br>Application site dermatitis (common)<br>Application site papules (common)<br>Application site rash (common)<br>Application site eczema (uncommon)<br>Application site discoloration (uncommon)<br>Application site erosion (uncommon)<br>Application site irritation (uncommon)<br>Application site oedema (uncommon)<br>Application site reaction (uncommon)<br>Application site bleeding (uncommon)<br>Application site dryness (uncommon)<br>Application site exfoliations (uncommon)<br>Application site haematoma (uncommon)<br>Application site vesicles (uncommon)
**Immune system disorders**	Food allergy (uncommon)
**Infections and infestations**	Ear infections (uncommon)
**Respiratory, thoracic and mediastinal disorders**	Asthma (uncommon)<br>Rhinitis allergic (uncommon)
**Skin and subcutaneous tissue disorders**	Urticaria (uncommon)<br>Eczema (uncommon)<br>Pruritus (uncommon)<br>Rash papular (uncommon)<br>Rash (uncommon)<br>Erythema (uncommon)<br>Dermatitis (uncommon)<br>Pruritus generalised (uncommon)<br>Rash pruritic (uncommon)<br>Skin irritation (uncommon)<br>Skin lesion (uncommon)<br>Urticaria contact (uncommon)

Abbreviations: AE = Adverse event, MedDRA = Medical Dictionary for Regulatory Activities, SOC = System organ class, PT = Preferred term.
Note: The expected frequencies in brackets are defined as: common (≥1/100, <1/10), uncommon (≥1/1,000, <1/100).

The experience from the VIPES study with Viaskin® Peanut 250 µg used to treat children showed that the most frequently observed local skin AEs to be expected are application site erythema, application site pruritus, application site swelling, application site dermatitis, and application site papules. In approximately 35% of the subjects these local skin reactions may be severe during 1 day or more. The majority of the local skin reactions developed within the first 2 months of therapy. They generally decreased in severity over time and resolved without sequelae and without treatment discontinuation.

In the children (6- to 11-years-old), time of occurrence of treatment-emergent AEs (TEAEs) considered related to Viaskin® Peanut was distributed as indicated in Table 4 with 70% of events appearing during the first 6 months.

Table 4: Time of Occurrence of Viaskin® Peanut-related Treatment-Emergent Adverse Events in the VIPES Study in Children (6- to 11-Years Old)

Time TEAE occurred				
**Time frame**	**Frequency**	**Percent**	**Cumulative Frequency**	**Cumulative Percent**
<Day 90	127	37.03	127	37.03
≥Day 90 to <Day 180	114	33.24	241	70.26
≥Day 180 to <Day 270	62	18.08	303	88.34
≥Day 270	40	11.66	343	100.00

Abbreviations: TEAE = Treatment-emergent adverse event.

The safety profile of the Viaskin® Peanut 250 µg group in the VIPES study according to the age range of children (6- to 7-years-old, 8- to 9-years-old and 10- to 11-years-old) and total is shown in Table 5.

Table 5: Safety Data of Viaskin® Peanut 250 µg According to Age Group in the VIPES Study in Children (6- to 11-Years-Old)

	**6 to 7 years N=7**	**8 to 9 years N=13**	**10-11 years N=8**	**Total (N=28)**
**Data from Subject Diary Proportion of Days*, Mean (SD)**				
Itching				
Grade 2-3	28.4 (32.7)	16.2 (22.3)	26.3 (26.5)	22.1 (25.9)
Grade 3	6.5 (14.4)	1.6 (2.7)	12.2 (23.4)	5.9 (14.5)
Redness				
Grade 2-3	35.1 (37.3)	16.9 (18.7)	31.5 (29.1)	25.6 (27.5)
Grade 3	10.5 (18.8)	0.4 (0.6)	4.4 (7.3)	4.1 (10.5)
Swelling				
Grade 2-3	27.6 (37.3)	12.1 (14.8)	29.0 (33.1)	20.8 (27.5)
Grade 3	12.7 (22.1)	0.4 (0.7)	8.8 (14.9)	5.9 (14.0)
**Investigator Skin Observation, n (%)**				
Month 1, prior to patch application				
Grade 0	3 (42.9)	5 (38.5)	5 (62.5)	13 (46.4)
Grade 1	1 (14.3)	2 (15.4)	1 (12.5)	4 (14.3)

	6 to 7 years N=7	8 to 9 years N=13	10-11 years N=8	Total (N=28)
Grade 2	1 (14.3)	3 (23.1)	2 (25.0)	6 (21.4)
Grade 3	2 (28.6)	3 (23.1)	0 (0.0)	5 (17.9)
Month 3, prior to patch application				
Grade 0	2 (33.3)	4 (30.8)	2 (28.6)	8 (30.8)
Grade 1	2 (33.3)	2 (15.4)	1 (14.3)	5 (19.2)
Grade 2	0 (0.0)	5 (38.5)	3 (42.9)	8 (30.8)
Grade 3	2 (33.3)	2 (15.4)	1 (14.3)	5 (19.2)
Missing	1	0	1	2
**TEAEs Considered Related, n (%)**				
Any TEAE	7 (100)	12 (92.3)	8 (100)	27 (96.4)
Eyes disorders	1 (14.3)	0 (0.0)	0 (0.0)	1 (3.6)
Gastrointestinal disorders	3 (42.9)	0 (0.0)	0 (0.0)	3 (10.7)
General disorders and administration site conditions	6 (85.7)	12 (92.3)	8 (100)	26 (92.9)
Skin and subcutaneous tissue disorders	3 (42.9)	6 (46.2)	1 (12.5)	10 (35.7)
**TEAEs considered related by maximum severity, n (%)**				
Any TEAE				
Mild	4 (57.1)	6 (46.2)	4 (50.0)	14 (50.0)
Moderate	2 (28.6)	6 (46.2)	4 (50.0)	12 (42.9)
Severe	1 (14.3)	0 (0.0)	0 (0.0)	1 (3.6)
General disorders and administration site conditions				
Mild	4 (57.1)	7 (53.8)	4 (50.0)	15 (53.6)
Moderate	1 (14.3)	5 (38.5)	4 (50.0)	10 (35.7)
Severe	1 (14.3)	0 (0.0)	0 (0.0)	1 (3.6)
Skin and subcutaneous tissue disorders				
Mild	1 (14.3)	3 (23.1)	0 (0.0)	4 (14.3)
Moderate	1 (14.3)	3 (23.1)	1 (12.5)	5 (17.9)
Severe	1 (14.3)	0 (0.0)	0 (0.0)	1 (3.6)

Abbreviations: n = Number of subjects; SD = Standard deviation; TEAE = Treatment-emergent adverse event.
*During the 90 first days of study treatment.

With regards to the incidence of subjects with itching, redness or swelling by grade, assessed by the subjects over the first 3 months of treatment, there was no clinically significant difference in the Viaskin® Peanut 250 µg group between the children 6- to 7-year-old and the 10- to 11-year-old, suggesting that the younger children and the older children would react alike locally. Even though it seemed that subjects 8- to 9-year-old reported less frequent local cutaneous reactions

Regarding the Investigator skin observation assessments, the pooled incidences of severity of Grade 2 and 3 were similar in the 3 age ranges at the Month 3, with a slight trend towards less reactions for the 6-7 year old subjects.

The incidence of TEAEs by System Organ Class (SOC) considered treatment related were not clinically significantly different between the 3 age ranges, except for the SOC gastrointestinal disorders, with few more cases in the group of 6 to 7-year-old children.

The data presented above support an overall conclusion that no safety concerns have been raised in association with the use of Viaskin® Peanut 250 µg in children, regardless of age. This is also confirmed by the fact that no children withdrew due to AEs.

1.2 Rationale

Viaskin® Peanut is a ready-to-use and easy-to-administer form of allergen immunotherapy, particularly adapted to the pediatric population. It is intended to induce clinical desensitization/tolerization to peanut in subjects allergic to peanut through interaction with the local APCs such as the epidermic Langerhans and dendritic cells. By utilizing the epicutaneous route of administration, Viaskin® Peanut is able to initiate these immunomodulatory processes while minimizing the potential safety concerns associated with systemic exposure to peanut allergenic proteins.

In the 12-month, Phase IIb VIPES study, among the 3 doses tested (50 µg, 100 µg and 250 µg), the highest dose of 250 µg Viaskin® Peanut displayed a strong efficacy with the highest magnitude of effect in children with a good safety profile as described in Section 1.1.1.2.3. Based on these findings, Viaskin® Peanut at 250 µg used for EPIT for a duration of 12 months is considered to be the optimal and suitable dose for treating children with peanut allergy. The dose of 250 µg was thus chosen to investigate the efficacy of Viaskin® Peanut in children aged 4 through 11 years for this pivotal Phase III study.

1.3 Risk-Benefit Assessment

The primary safety concern for any allergen specific immunotherapy is related to the risk of inducing systemic, severe or life-threatening allergic reactions. Viaskin® Peanut applied epicutaneously was developed in this sense, which is to dramatically reduce the risk of these severe systemic reactions by applying the peanut allergens on the skin and reaching the immune system through the cutaneous Langerhans and dendritic cells. Safety information from the completed Phase Ib PEP01.09 study, the Phase II ARACHILD study and the Phase IIb VIPES study, as well as available safety information from the ongoing studies OLFUS-VIPES and CoFAR6, have demonstrated a good safety profile for Viaskin® Peanut 250 µg in children as of 4 years of age and above. The expected local skin reactions triggered by Viaskin® Peanut at the site of patch application, which are pruritus, erythema, edema, and urticaria, are in the majority of cases mild or moderate and managed and controlled satisfactorily by the subjects with topical medications containing corticosteroids. As a consequence, a very good compliance was shown in the studies conducted so far.

Furthermore, especially in the Phase IIb VIPES study, Viaskin® Peanut 250 µg has shown a statistically significant magnitude of effect with up to 53.6% of children responding positively to the treatment versus 19.4% for placebo, as per the dose-finding study primary endpoint. In a post-hoc analysis with a higher stringent criterion for the treatment benefit (the same stringency is used for the primary efficacy endpoint in this Phase III pivotal PEPITES study), the magnitude of effect of Viaskin® Peanut 250 µg

was even higher (46.5% response rate in the active treatment group versus 6.5% response rate for placebo).

Overall, the available information gathered from several clinical studies conducted in children with Viaskin® Peanut for several years suggest that Viaskin® Peanut 250 µg dose presents a favorable benefit-risk ratio.

2 STUDY OBJECTIVES

The objective of this study is to assess the efficacy and safety of Viaskin® Peanut to induce desensitization to peanut in peanut-allergic subjects 4 through 11 years of age after a 12-month treatment period by EPIT.

3 OVERALL DESIGN AND PLAN OF THE STUDY

3.1 Overview

This is a 12-month, Phase III, double-blind, placebo-controlled, randomized study to assess the efficacy and safety of Viaskin® Peanut, dosed at 250 µg peanut protein (per patch) in peanut-allergic male and female children from 4 through 11 years of age.

During the maximum 6-week screening period, subjects will undergo an entry/screening DBPCFC to peanut to confirm their allergy to peanut and to determine their entry or screening peanut ED. The starting dose of the challenge will be 1 mg peanut protein and will escalate up to a highest dose of 300 mg peanut protein. Subjects who react at or below the dose of 300 mg peanut protein are considered eligible. Randomization of eligible subjects will occur in a 2:1 ratio to Viaskin® Peanut dosed at 250 µg peanut protein or placebo. An Interactive Web Response System (IWRS) will be used to ensure that the randomization and the allocation of treatments to subjects during the 12-month treatment period are performed in a blinded manner. Detailed information on study assessments and procedures is provided in Section 6.

Subjects randomized in the study and consenting for the genetic analysis will be evaluated for mutations in the filaggrin gene.

Subjects will apply a Viaskin® patch containing either peanut or placebo daily for a period of 12 months. At Month 12, a post-treatment DBPCFC to peanut will be performed, with a starting dose of 1 mg peanut protein with escalation up to a highest dose of 2,000 mg peanut protein. This evaluation will help determine the primary efficacy endpoint of this pivotal study.

The follow-up period will comprise a maximum of 3 weeks.

The overall maximum study duration for each subject is approximately 61 weeks (6-week screening period, 12-month treatment period and 2-week follow-up period). During this period, subjects will attend a total of 12 study visits, including 3 visits during the screening period, 8 visits during the treatment period, and 1 visit at the end of the follow-up period. In addition, 5 telephone contacts will be made during the treatment period.

After completion of the 12-month blinded study, all subjects, including the placebo subjects, will be offered the opportunity to participate in an open-label extension study to receive an additional 24 months of treatment with active Viaskin® Peanut.

It is planned to screen approximately 470 subjects in this study to achieve 330 subjects randomized (220 subjects in the Viaskin® Peanut group and 110 subjects in the placebo group) and 280 subjects evaluable for the primary efficacy endpoint (185 subjects in the Viaskin® Peanut group and 95 subjects in the placebo group).

Throughout the screening and randomization process, and to guarantee an adequate and sufficient representation of subjects of 4, 5 and 11 years of age (the youngest and the oldest of the age range), a minimum of 20 subjects of each of these 3 ages must be randomized. Each of these ages will thus represent a minimum of 7.5% of the overall number of randomized subjects. Besides, a minimum number of 75 children of either 4 or 5 years of age must be randomized. The 4 to 5 years old subgroup will thus represent 22.7% of the overall number of randomized subjects.

Subjects will be stratified at randomization by their entry/screening DBPCFC ED in 1 of the 2 strata defined below and by study center. The entry/screening DBPCFC ED strata are defined as follows:

- Stratum 1: Children with a screening ED of 1 mg, 3 mg or 10 mg;
- Stratum 2: Children with a screening ED of 30 mg, 100 mg or 300 mg.

The randomization scheme will ensure that the ratio of active treatment to placebo is maintained in each stratum. No randomization ratio will be fixed for the 2 screening ED strata; however, according to the Phase IIb VIPES study data, the following distribution could be expected:

- 1/3 of children with a screening ED from 1 mg to 10 mg;
- 2/3 of children with a screening ED from 30 mg to 300 mg.

The study will be conducted at approximately 28 to 40 centers in 4 to 7 countries in Australia, Europe and North America with Investigators and staff who are trained and experienced in the diagnosis and management of peanut allergy and anaphylaxis, and equipped and capable of performing a DBPCFC in children.

Criteria for subject withdrawal and study stopping rules are described in Section 4.3.

A Data and Safety Monitoring Board (DSMB) composed of experts in food allergy and in the methodology of clinical studies will review study safety data at specific intervals during the study and on an *ad hoc* basis. A specific DSMB meeting will be held when the first 15 subjects 4 to 5 years of age have been randomized and treated for at least 4 weeks (that is, have completed the Month 1 visit) (see Section 9.9).

No interim analyses are planned for this study.

The Schedule of Procedures is shown in Table 7 and the study design is presented in Figure 1.

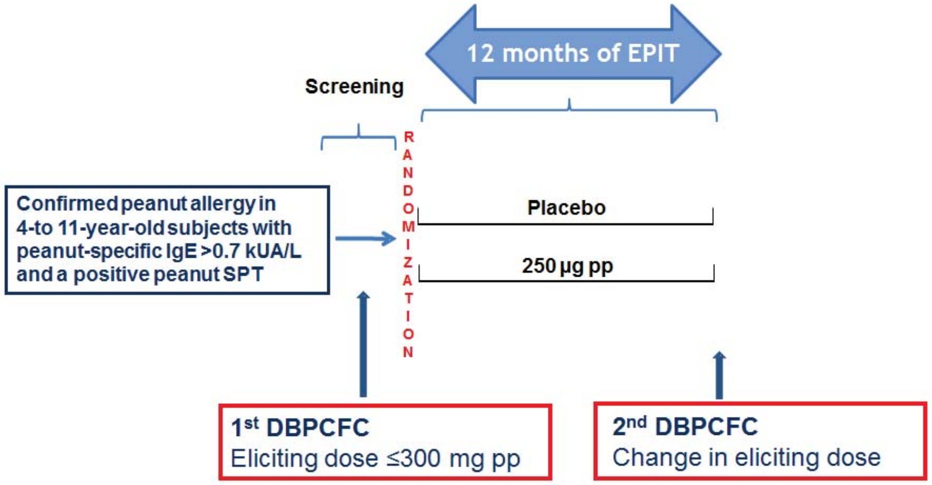

Abbreviations: DBPCFC = Double-blind, placebo-controlled food challenge; EPIT = Epicutaneous immunotherapy; IgE = Immunoglobulin E; pp = peanut protein; SPT = Skin prick test.

Figure 1: Study Design

3.2 Criteria for Evaluation of the Study

3.2.1 Efficacy Criteria

3.2.1.1 Primary Efficacy Endpoint

The primary efficacy endpoint is the percentage of treatment responders at Month 12 in the active Viaskin® Peanut 250 µg group compared to the placebo group in the overall population. A subject is defined as a treatment responder if:

- The initial ED was >10 mg peanut protein and the ED is ≥1,000 mg peanut protein at the post-treatment DBPCFC at Month 12; or
- The initial ED was ≤10 mg peanut protein and the ED is ≥300 mg peanut protein at the post-treatment DBPCFC at Month 12.

3.2.1.2 Secondary Efficacy Endpoint

The following secondary efficacy endpoints will be analyzed:

- The percentage of treatment responders at Month 12 in the active Viaskin® Peanut 250 µg group compared to the placebo group in each of the 2 screening ED strata;
- The percentage of treatment responders at Month 12 in the active Viaskin® Peanut 250 µg group compared to the placebo group in each of the 2 age subgroups (4 to 5 years-old; 6 to 11 years-old);
- The mean and median cumulative reactive dose of peanut protein and change from baseline at Month 12 in the active Viaskin® Peanut 250 µg group versus the placebo group, overall and for each screening ED stratum;

- The mean and median ED of peanut protein and change from baseline at Month 12 in the active Viaskin® Peanut 250 µg group versus the placebo group, overall as well as in each of the 2 screening ED strata;
- The percentage of subjects responsive (those showing objective symptoms leading to DBPCFC stop) to a cumulative dose ≥1,444 mg peanut protein at the post-treatment DBPCFC at Month 12 in the active Viaskin® Peanut 250 µg group versus the placebo group, overall as well as in each of the 2 screening ED strata;
- The percentage of subjects unresponsive (those showing no objective symptoms leading to DBPCFC stop) to a cumulative dose ≥1,444 mg peanut protein at the post-treatment DBPCFC at Month 12 in the active Viaskin® Peanut 250 µg group versus the placebo group, overall as well as in each of the 2 screening ED strata;
- The percentage of subjects unresponsive (those showing no objective symptoms leading to DBPCFC stop) to the highest dose of peanut protein, which is the percentage of subjects who pass the post-treatment DBPCFC at Month 12 in the active Viaskin® Peanut 250 µg group versus the placebo group, overall as well as in each of the 2 screening ED strata.

3.2.1.3 Other Efficacy Endpoints

- The change from baseline in peanut-specific IgE and immunoglobulin G4 subtype (IgG4) at months 3, 6 and 12 in the active Viaskin® Peanut 250 µg group versus the placebo group, overall as well as in each of the 2 screening ED strata;
- The change from baseline in peanut skin prick testing maximum average wheal diameters at months 3, 6 and 12 in the active Viaskin® Peanut 250 µg group versus the placebo group, in the overall population;
- Description of the quality of life questionnaires (Food Allergy Quality of Life Questionnaire [FAQLQ]/Food Allergy Independent Measure [FAIM]) and change from baseline in FAQLQ score at Month 12 in the overall population (for those countries where the translated and validated questionnaires are available and used).

Details of the assessment of each of the efficacy endpoints are provided in Section 6.1 and details of the corresponding statistical analysis are provided in Section 8.5.

3.2.2 Safety Criteria

The following study drug safety criteria will be evaluated:

- AEs and treatment-emergent adverse events (TEAEs) by System Organ Class (SOC) and Preferred Terms (PTs);
- TEAEs by maximum severity and relatedness to Viaskin® Peanut 250 µg;
- Incidence, duration and maximum severity of local cutaneous Viaskin® Peanut 250 µg-induced AEs as assessed by the subject;
- Severity of local cutaneous Viaskin® Peanut 250 µg-induced AEs as assessed by the Investigator;

- Adverse events of special interest (AESI) including grade 4 local cutaneous reactions and systemic allergic AEs considered related to Viaskin® Peanut 250 µg;
- Serious adverse events (SAEs) by SOC and PTs, and relatedness to Viaskin® Peanut 250 µg;
- Laboratory data, physical examinations and vital signs;
- Spirometry results or Peak Expiratory Flow (PEF) results.

The above criteria will be studied in the overall Safety population, as well as for each of the age ranges 4 to 5 years, 6 to 8 years and 9 to 11 years, and for each of the screening ED strata.

Besides, the following study procedure safety criteria will be evaluated:

- Objective symptoms elicited during the entry/screening DBPCFC and post-treatment DBPCFC at Month 12 by severity;
- Change in severity of objective symptoms elicited during the DBPCFC from baseline to Month 12 in the active Viaskin® Peanut 250 µg group versus the placebo group;
- SAEs elicited during the entry/screening DBPCFC and post-treatment DBPCFC at Month 12.

Details of the assessment of each of the safety criteria are provided in Section 6.2 and details of the corresponding statistical analysis are provided in Section 8.6.

3.2.3 Exploratory Criteria

The following exploratory criteria will be evaluated:

- The change from baseline in IgE and IgG4 specific to peanut protein components at months 3, 6 and 12 in the active Viaskin® Peanut 250 µg group versus the placebo group;
- Enumeration and characterization of reactions triggered by accidental consumption of peanut during the study;
- Analysis of “risk-taking behavior” of subjects (voluntary peanut consumption) during the study;
- Epigenetic modifications of the promoters of some specific genes;
- Safety sub-analysis in subjects with mutations in the filaggrin gene versus wild type subjects;
- Sensitization status to some other allergies and their evolution over the study period;
- Scoring atopic dermatitis (SCORAD) evolution over time.

Details of the assessment of each of the exploratory criteria are provided in Section 6.4 and details of the corresponding statistical analysis are provided in Section 8.7.

3.3 Justification of the Study Design

This study is designed to assess the efficacy and safety of Viaskin® Peanut 250 µg in children aged 4 through 11 years compared to placebo.

The study design follows the International Conference on Harmonization (ICH) guideline on general considerations for clinical studies (34).

Subject randomization and double-blinding with regard to treatment with Viaskin® Peanut and placebo were chosen in order to prevent bias in treatment allocation and in the assessment of both safety and efficacy. The use of a placebo group in this study is justified to obtain reliable scientific evidence for the evaluation of this new medicinal product.

Peanut allergy is of increasing prevalence in children (3,4,5). It is thus anticipated that the selected pediatric population of peanut allergic subjects will draw a direct benefit from this study.

The DBPCFC is the “gold standard” to diagnose food allergy (35). Since it carries a risk of inducing potentially severe allergic reactions, subjects will be appropriately selected for the challenge, based on their clinical history and peanut-specific IgE test results.

Subjects participating in this study must have a positive DBPCFC at ≤300 mg of peanut protein. The ED of peanut protein, that is the dose of peanut protein administered to subjects during the DBPCFC procedure which causes an objective allergic reaction leading to stopping the challenge, is capped to 300 mg, with the starting dose of 1 mg. Although the average amount of peanut consumed in an accidental exposure has not been accurately quantified, it is generally believed to be no more than 1 to 2 peanut kernels, or the equivalent of approximately 300 to 600 mg of peanut protein (36,37,38). Thus, this study will enroll subjects who would react at or below 300 mg peanut protein with the objective of inducing desensitization in order to increase significantly the subjects’ threshold levels of peanut.

The selected protein dose of 250 µg for treating peanut-allergic children in this Phase III PEPITES study is based on the safety and efficacy results for children 6 to11 years of age in the 12-month Phase IIb VIPES study, but also on the safety information from the CoFAR6 study for subjects 4 to 5 years of age (see Sections 1.1.1.2.3 and 1.1.1.2.5).

All subjects will continue with their usual peanut-free diet and label reading to avoid as much as possible any accidental peanut consumption during the duration of the study.

The primary endpoint in this study will be assessed at Month 12 based on positive results of previous clinical studies showing evidence of desensitization with peanut EPIT after 12 months of treatment.

4 STUDY POPULATION

The study population will consist of children with peanut allergy. Study participation will require consent from a legally authorized representative. Subjects must meet all the inclusion criteria and none of the exclusion criteria.

All subjects will continue with their usual peanut-free diet and label reading of food products to avoid as much as possible any accidental peanut consumption for the duration of the study.

4.1 Inclusion Criteria

Subjects will be enrolled in this study only if they meet all of the following criteria:

1. Male or female children aged 4 through 11 years at Visit 1;
2. Physician-diagnosis of peanut allergy or children with a well-documented medical history of IgE-mediated symptoms after ingestion of peanut and currently following a strict peanut-free diet, but without a physician diagnosis;
3. Signed informed consent of parents/guardians of the child and child's assent (for children ≥7 years of age or as per the country-specific regulations);
4. Negative urine pregnancy test for female subjects of childbearing potential. Female subjects of childbearing potential must agree and commit to use effective medical methods of contraception for the entire duration of their participation in the study. Sexual abstinence will be accepted as an effective method of contraception;
5. Peanut-specific IgE level (ImmunoCAP system) >0.7 kU/L;
6. Positive peanut SPT with a largest wheal diameter:
   a. ≥6 mm for children 4 through 5 years of age at Visit 1,
   b. ≥8 mm for children 6 years and above at Visit 1;
7. Positive DBPCFC at ≤300 mg peanut protein: the ED of peanut protein during the entry/screening DBPCFC is capped to 300 mg, as in subjects must have objective IgE-mediated symptoms to peanut leading to stopping the challenge at ≤300 mg peanut protein;
8. Ability to perform spirometry in accordance with the American Thoracic Society (ATS) guidelines 2007 (39) for subjects ≥6 years of age. Ability to perform peak expiratory flow (PEF) measurements for subjects ≥5 years of age. Subjects from 6 to 8 years of age who have documented inability to adequately perform spirometry can perform the PEF measurement instead. Subjects 4 years of age can be enrolled if they had no clinical features of moderate or severe persistent asthma[1] within 1 year prior to visit 1.

[1] National Heart, Lung, and Blood Institute. Expert panel report 3: guidelines for the diagnosis and management of asthma - summary report, 2007. Available online at: http://www.nhlbi.nih.gov/guidelines/asthma/asthsumm.pdf. Accessed July 06, 2015.

9. Subject and/or parents/guardians willing to comply with all study requirements during the subject's participation in the study.

4.2 Exclusion Criteria

Subjects will be enrolled in this study only if they meet none of the following criteria:

1. History of severe anaphylaxis to peanut with any of the following symptoms: hypotension, hypoxia, neurological compromise (collapse, loss of consciousness or incontinence);
2. Pregnancy or lactation;
3. Severe reaction during the entry/screening DBPCFC, defined as need for intubation, hypotension persisting after epinephrine administration, and/or the need for ≥3 doses of epinephrine;
4. Objective IgE-mediated symptoms to the placebo formula during the entry/screening DBPCFC and leading to stopping the challenge;
5. Viral upper respiratory infection or gastroenteritis within 7 days of a food challenge (challenge must be rescheduled to occur at least 7 days after recovery);
6. Any clinically significant abnormality identified at the time of screening such as major infectious diseases (for example chicken pox, measles) which in the judgment of the Investigator may preclude safe participation or strict compliance with the protocol procedures (subjects can be considered for the study after recovery from these diseases);
7. Generalized dermatologic disease (for example active atopic dermatitis, uncontrolled generalized active eczema, ichthyosis vulgaris) extending widely on the skin and especially on the back with no intact zones to apply the Viaskin® patches;
8. Symptomatic seasonal allergies that may interfere with the conduct of a DBPCFC. Subjects should be screened at a time when such allergies are asymptomatic (for example outside of the culprit season);
9. Known hypersensitivity to any of the Viaskin® patch components (except peanut), including the adhesive film;
10. Known hypersensitivity to any component of the food challenge formula (except peanut);
11. Inability to discontinue short-acting antihistamines or long-acting antihistamines for the minimum wash-out periods as specified in the table in APPENDIX 4) prior to the skin prick testings or the food challenges;
12. Spirometry forced expiratory volume in 1 second (FEV_1) <80% of the predicted value at screening (visit 1) for subjects ≥6 years of age and able to perform the spirometry, or PEF <80% of predicted value at screening (visit 1) for subjects performing only the PEF measurements.
13. Diagnosis of asthma that fulfills any of the following criteria:

a. Uncontrolled persistent asthma as defined by National Asthma Education and Prevention Program Asthma guidelines 2007 (40) or by Global Initiative for Asthma guidelines 2015 (41),

b. Asthma treated with either a high daily dose of inhaled corticosteroid or with a combination therapy of a medium daily dose of inhaled corticosteroid with a long-acting inhaled β2-agonist or with a combination therapy of a high daily dose of inhaled corticosteroid with a long-acting inhaled β2-agonist (a list of daily dosages of inhaled corticosteroid is provided in APPENDIX 2). Asthmatic subjects treated with a medium daily dose of inhaled corticosteroids are eligible. Intermittent asthmatic subjects who require intermittent use of inhaled corticosteroids for rescue are also eligible,

c. Two or more systemic corticosteroid courses for asthma in the past year or 1 oral corticosteroid course for asthma within 3 months prior to Visit 1, or during screening period (unless used to treat symptoms triggered by the DBPCFC),

d. Prior intubation/mechanical ventilation for asthma within 1 year prior to Visit 1, or during screening;

14. Receiving β-blocking agents, angiotensin-converting enzyme inhibitors, angiotensin-receptor blockers, calcium channel blockers or tricyclic antidepressant therapy;
15. Received anti-tumor necrosis factor drugs or anti-IgE drugs (such as omalizumab) or any biologic immunomodulatory therapy within 1 year prior to Visit 1, during screening period or during study participation;
16. Use of systemic long-acting corticosteroids within 12 weeks prior to Visit 1 and/or use of systemic short-acting corticosteroids within 4 weeks prior to Visit 1 or during screening (unless used for DBPCFC symptoms) (see APPENDIX 3);
17. Prior or concomitant history of any immunotherapy to any food allergy (for example EPIT, OIT, SLIT, or specific oral tolerance induction);
18. Receiving or planning to receive any aeroallergen immunotherapy during their participation in the study. Aeroallergen immunotherapy must be discontinued at the time of Visit 1;
19. Subject or parents/guardians of subjects with obvious excessive anxiety and unlikely to cope with the conditions of a food challenge;
20. Past or currently active disease(s) which, in the opinion of the Investigator or the Sponsor, may affect the subject's participation in this study or place the subject at increased risk during participation in the study, including but not limited to eosinophilic gastrointestinal disorders, autoimmune disorders, immunodeficiency, malignancy, uncontrolled diseases (for example hypertension, psychiatric illness, cardiac disease), or other disorders (for example liver, gastrointestinal, kidney, cardiovascular, pulmonary disease, or blood disorders);
21. Any disorder in which epinephrine is contraindicated such as coronary artery disease, uncontrolled hypertension, or serious ventricular arrhythmias;

22. Subjects unable to follow the protocol requirements;
23. Current participation in another clinical trial or participation in another clinical trial in the last 3 months prior to visit 1;
24. Diagnosis of mast cell disorders including mastocytosis or urticaria pigmentosa as well as hereditary or idiopathic angioedema;
25. Subjects receiving cyclosporine or other immunosuppressive agents within 1 year prior to Visit 1, or during the screening period or during study participation. Topical calcineurin inhibitors are permitted;
26. Subjects with severe psychiatric, psychological or neurological disorders;
27. Subjects being in any relationship or dependency with the sponsor and/or the investigator or the study staff.

4.3 Patient Withdrawal and Replacement

4.3.1 Criteria for Withdrawal from Study Treatment and Study

Subjects may withdraw from the study including follow-up at any time without penalty and for any reason without prejudice to their future medical care.

Subjects must be withdrawn from the **study treatment** under the following circumstances:

- Pregnancy (see Section 6.2.1.7);
- Severe “maculo-papular rash” or severe “dermatitis” failed to be controlled by adequate corrective treatments (including topical corticosteroids) and in spite of several study treatment interruptions;
- Severe anaphylaxis (or stage 3 anaphylaxis) (see APPENDIX 5) related to Viaskin® Peanut 250 µg patch application;
- More than 1 epinephrine injection for an AE related to Viaskin® Peanut 250 µg patch application (and not occurring during the DBPCFC).

If subjects are to withdraw due to pregnancy, the procedures that should be followed are described in Section 6.2.1.7.

Subjects may be required to withdraw from the **study treatment or study** after discussion with the Sponsor and/or Investigator for the following reasons:

- AE(s);
- At the discretion of the Investigator, if she/he decides that it is in the subject’s best interest to be withdrawn from the study;
- The subject is unwilling to continue in the study (consent withdrawal);
- Lack of compliance with protocol requirements and procedures;
- The Sponsor, Regulatory Authorities, or Independent Ethics Committees (IECs)/Institutional Review Boards (IRBs) for any reason, stop the study;

- The subject fails to return to the clinic for scheduled visits and does not respond to telephone or written attempts at contact (lost to follow-up);
- Premature termination of the entire study as described in Section 9.11.

In all cases, the primary reason for withdrawal, must be recorded in the e-CRF.

4.3.2 Study Stopping Rules

Study enrollment will be suspended pending an expedited safety review by the independent DSMB if any of the following occur:

1. Any death related to Viaskin® patch dosing;
2. More than one case of severe anaphylaxis (or stage 3 anaphylaxis) (see APPENDIX 5) related to Viaskin® Peanut 250 µg patch application (not occurring during the DBPCFC);
3. More than 3 subjects requiring more than 1 injection of epinephrine for an AE related to the Viaskin® Peanut 250 µg patch application (and not occurring during the DBPCFC).

Upon safety review, 1 of the following outcomes will be determined:

- Accrual to the study may continue without modification;
- Accrual to the study may continue with modifications as prescribed by the DSMB;
- Accrual to the study should be discontinued.

4.3.3 Replacement of Withdrawn Subjects

Subjects who withdraw prematurely after having been randomized and having received at least 1 dose of the study drug will not be replaced.

4.3.4 Data Collection and Follow-up after Withdrawal

If a subject is prematurely withdrawn from the study drug for any reason before completing all study visits, the Investigator must make every effort to perform the evaluations described for the Early Termination Visit (see Section 7.2.12). The Investigator must furthermore complete all appropriate subject diary and e-CRF pages, providing the date and explanation for the subject’s withdrawal/discontinuation. When indicated, the Investigator must arrange for appropriate follow-up and/or alternative medical care of the discontinued subject.

If the subject fails to attend a scheduled End of Study Visit, there will be at least 2 attempts to contact the subject’s parents/guardians via telephone and written communication. If these receive no reply, the subject will be considered as lost to follow-up.

4.4 Planned Sample Size and Number of Study Centers

It is planned to randomize 330 subjects at 28 to 40 centers in 4 to 7 countries for this study. See Section 8.10 for a discussion of sample size.

4.5 Patient Identification and Randomization

4.5.1 Patient Identification

At screening, each subject will receive a unique, 4-digit, screening number. Screened subjects who drop out of the study before randomization will retain their screening number. The screening number for each subject will be a combination of the 2-digit site number plus the 2-digit number assigned to the subject according to her/his chronological order of screening at that site. The screening number will be used as the subject identifier throughout the study.

4.5.2 Randomization Scheme

An IWRS will randomize subjects and assign the appropriate treatment number or kit number. Subjects will be randomized on a 2:1 basis to Viaskin® Peanut 250 µg or placebo.

Randomization will be stratified by center and by screening ED and managed centrally. The randomization scheme will ensure that the ratio of active treatment to placebo is maintained in Stratum 1 (children with a screening ED from 1 mg to 10 mg) and Stratum 2 (children with a screening ED from 30 mg to 300 mg).

No randomization ratio will be fixed for the 2 screening ED strata; however, according to the Phase IIb VIPES study, the following distribution could be expected:

- 1/3 of children with a screening ED from 1 mg to 10 mg;
- 2/3 of children with a screening ED from 30 mg to 300 mg.

The randomization codes will be maintained by IWRS.

Blinding and breaking the blind procedures are described in Section 5.4.

4.5.3 Allocation/Randomization of Patients to Treatment

Randomization of subjects to treatment will occur at Visit 4 after all screening procedures have been performed and eligibility for the study confirmed. Each randomized subject will be assigned by the IWRS a kit number based on a pre-defined algorithm/pre-defined randomization list.

5 STUDY DRUG

5.1 Identity

The study drug, Viaskin® Peanut, is a cutaneous patch (Viaskin® patch) containing a dry deposit of a formulation of peanut protein extract. The Viaskin patch is a 34 mm-side long square-shaped patch with a rounded condensation chamber of 18 mm-inner diameter. The peanut allergens are deposited on the backing of the patch chamber by electrospraying a liquid peanut protein formulation, which dries instantly. The outer adhesive part of the condensation chamber is composed of a small band of adhesive foam to stick to the skin. The Viaskin® patch is identical to the ones used in prior clinical studies, but now also includes a hypoallergenic adhesive film that will help prevent the Viaskin® patch from coming off inadvertently.

The drug substance is an unmodified, lyophilized peanut extract produced from the extraction and freeze drying of defatted peanut flour. The drug substance contains the biologically active ingredients, the peanut proteins. This drug substance derives from a natural source material of biologic origin, the peanut seed from the Virginia variety of *Arachis hypogaea* and the extract contains all peanut proteins.

The other components of the study drug are inactive excipients: ethanol, surfactant (Polyoxyl 20 oleyl ether), and buffering agents (trometamol and histidine).

Study subjects will be administered either Viaskin® Peanut at 250 µg peanut protein (active) or Viaskin® patch with placebo, according to randomization. The placebo treatment will consist of a similar formulation, but will be devoid of peanut protein.

Both, Viaskin® Peanut 250 µg and Viaskin® placebo will be manufactured by AMATSI, Montpellier, France and labeled, packaged and released for clinical use by CREAPHARM, Bordeaux, France, in accordance with the requirements of Good Manufacturing Practices.

5.2 Administration

Subjects will be randomized to receive either active Viaskin® Peanut 250 µg or placebo, in a 2:1 ratio (active versus placebo).

During the 12-month blinded treatment period, except for the first 2 weeks (see below), the Viaskin® patch will be applied on the skin for 24 hours (± 4 hours of allowance) every day and renewed on a daily basis, 1 new patch per day.

The location of patch application is the inter-scapular area of the back of the subjects. There will be 6 zones for applying the patch, 3 on each side of the spine (see Figure 2). The first Viaskin® patch will be applied on zone 1, the second on zone 2 (after removal of the first patch), and so forth, until all 6 zones have been used. After zone 6, dosing restarts with zone 1 and continues sequentially, as described.

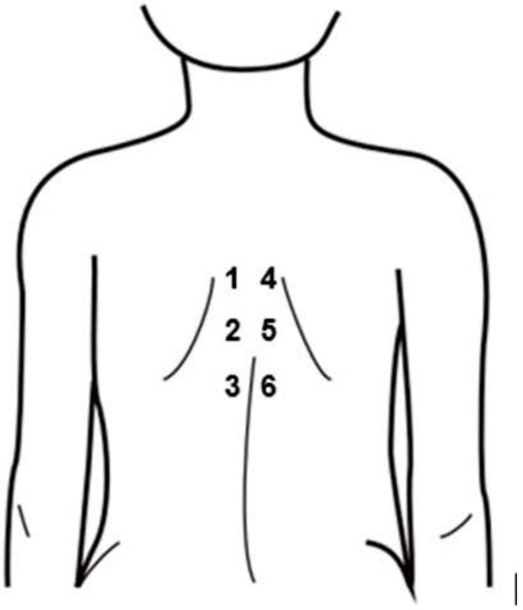

Figure 2: Schematic Representation of Viaskin® Patch Application on the Back of the Subjects

To better ensure the safety of the Viaskin® patch at the initiation of treatment, the duration of application of the Viaskin® patch will be progressively increased as follows:

- During the first week (from Day 1 through Day 7), the patches will be applied for 6 hours every day;
- During the second week (from Day 8 through Day 14), the patches will be applied for 12 hours every day;
- From the third week onwards (Day 15), the patches will be applied for the entire 24 hours.

If possible, the subjects should take advantage of their shower/bath time to change the patch. The previous Viaskin® patch should be removed just before the shower/bath, and the new Viaskin® patch should be applied a few minutes after the shower/bath and after drying the skin. Application of the Viaskin® patch at a similar time for each daily application (morning or evening) is recommended. If the subject does not bathe or shower daily or at the same time daily, it is recommended that the zone to which the patch will be applied be cleaned with a moist disposable napkin or tissue and dried prior to application.

A Viaskin® patch must not be re-applied. In case the Viaskin® patch comes off, it should be immediately discarded.

A new patch can be applied the same day to replace the previous patch that came off only if that patch came off within 2 hours after being applied. However, no new patch should be applied the same day if the previous patch came off more than 2 hours after being applied. In that case, a new patch will be applied once 24 hours have passed since the previous patch (the one that came off) was applied. After the Viaskin® patch comes off,

or after removing a Viaskin® patch to apply a new one, it is recommended that the subject's parents/guardians wipes the zone with a moist disposable napkin or a moist disposable tissue and then washes her/his hands to prevent accidental manual transmission of peanut protein.

After the first application of the Viaskin® patch at the study site, all subjects will be observed for 3 hours before being discharged in order to check and grade any reactions under or around the patch.

Because the study includes children aged 4 through 5 years, additional safety measures have been adopted and generalized to all ages. On Day 4 and on Day 22, specific phone contacts will be made to the parents/guardians to assess the safety of patch applications on the back. If required, the subjects may come to the site sooner than planned to be seen and evaluated by the Investigator.

Of note, the recommended duration of daily Viaskin® patch application is 24 hours of application per day. However, any daily duration of Viaskin® patch application of 24 hours ± 4 hours will be allowed.

5.2.1 Adjustment of Viaskin® Patch Application in Case of Local or Systemic Reactions

When subjects are unable to apply the Viaskin® patch for the recommended durations as described above because of local intense or severe reactions, under or adjacent to the patch site, the patch should be removed immediately, the site of application wiped with a moist disposable tissue and a topical corticosteroid medication might be topically applied to treat the reaction. The parents/guardians should take a photo of the back of the subject to document how intense and extended the local reactions are. In the specific case of local intense or severe reactions, it is mandatory that the next Viaskin® patch is applied only the next day on the next zone; no other Viaskin® patch must be applied the same day. In case of re-appearance of the local intense or severe reactions after application of the next patch the following day, the patch must again be removed. The next Viaskin® patch should be applied only the next day on the next zone. And the same process is repeated every day after patch application in case of local intense or severe reactions.

As a consequence, the daily duration of Viaskin® patch application should be adjusted/reduced as necessary, and subjects may need more than the 14 days previously described before they can apply and tolerate the Viaskin® patch for the full 24 hours daily.

In case of any suspected systemic reactions related to patch application (including cutaneous reactions distant from the sites of patch application), the same safety precautions should be followed: the Viaskin® patch is removed immediately, the site of application wiped with a moist disposable tissue and a treatment can be given to treat the reaction: antihistamines or topical corticosteroids with additional oral corticosteroids or similar anti-allergic drugs, as deemed necessary by the Investigator. The next Viaskin® patch should be applied the next day on the next zone. As a consequence, the daily duration of patch application should be adjusted/reduced as necessary, and subjects may need more time than initially defined before they can apply the Viaskin® patch for the full 24 hours daily.

The subject's parents/guardians will be instructed to contact the Investigator in case of intense or severe local reactions lasting for more than 1 day or any unexpected reactions during the treatment period, in particular in case of appearance of vesicles under the Viaskin® patch or close to the Viaskin® patch site application. It is then recommended that the parents/guardian take a photo of the back of the subject to document these intense or severe reactions.

5.2.2 Safety Precaution Information

A leaflet with safety precautions for using the Viaskin® patch and instructions to follow in case of any safety issue will be given to each subject. This safety leaflet will specify at least the following information:

1. Instructions and procedures to apply the patch safely and correctly in the back of the subject;
2. Necessity to call the investigative site staff in case of intense or severe local reactions lasting for more than 1 day or any unexpected reactions during the treatment period, in particular any appearance of vesicles/blisters under the patch or close to the area of patch application and a recommendation to take photos of the site of application to document such a situation;
3. Necessity to call the investigative site staff in case of occurrence of chicken pox or measles. At the same time, interruption of patch application until recovery;
4. In case of active eczema extending on the back of the child: stop applying the patch until recovery from the active eczema on the back;
5. Anaphylaxis Emergency Action Plan in case of a suspected anaphylactic reaction and how to administer the epinephrine auto-injector to rapidly treat the reaction;
6. Subject stopping rules (subject are consequently withdrawn):
    a. Severe "maculo-papular rash" or severe "dermatitis" at sites of patch application not controlled by topical corticosteroids,
    b. Severe anaphylaxis related to Viaskin® Peanut 250 µg patch application confirmed by an expedited safety review.
    c. More than 1 epinephrine injection in relation to Viaskin® Peanut 250 µg patch application (and not occurring during DBPCFC).

5.3 Packaging, Labeling and Storage

The study drug (active and placebo) will be packaged and labeled by CREAPHARM (Bordeaux, France) in accordance with applicable local regulatory requirements. One Viaskin® patch will be placed per pouch and each pouch will be labeled. The labeled pouches will be placed in labeled treatment boxes to be dispensed to subjects at each visit, with enough quantity of Viaskin® patches to cover the period between 2 consecutive visits.

The labeled and packaged study drug must be stored in accordance with the Sponsor's instructions (in the monitored refrigerator between 2°C and 8°C [35.6°F to 46.4°F]. Shipments from depots to clinical sites will be performed at refrigerated temperature between 2°C to 8°C [35.6°F to 46.4°F] with a temperature monitoring device. Storage at

ambient temperature for short and/or intermittent periods of time, including transportation from the clinical site to the subject's home, is permitted.

Upon receipt of a shipment request via IWRS, the study drug will be shipped to the clinical site. The site pharmacist or any other staff member designated for this task will receive and store the study drug until the time of dispensing it to the Investigators. Until dispensed by the Investigator to the subjects, the study drug will be stored in a securely locked area, accessible to authorized personnel only. At the end of the study, or at times designated by the Sponsor, and after complete accountability, the site pharmacist or the designated person will be responsible for preparing the return of the unused study drug to the study drug distributor. Destruction of study drug on-site should be avoided. However, in some cases, and upon authorization from the Sponsor, destroying the unused study drug on-site, with provision of a corresponding certificate of destruction, may be allowed.

5.4 Blinding and Breaking the Blind

The study will be performed in a double-blind manner. Active and placebo Viaskin® patches will be supplied in identical pouches and will be similar in physical appearance, thereby enabling double-blind conditions.

The treatment codes will be held according to the IWRS. Further instructions for emergency code break will be provided in a separate IWRS Manual.

The study blind should not be broken except in a medical emergency (where knowledge of the study drug received would affect the treatment of the emergency) or due to a regulatory requirement (for example for suspected unexpected serious adverse reactions). The blind will only be broken at the discretion of the Investigator, and if possible, following discussion on a case-by-case basis with the Sponsor/Medical Monitor. All calls resulting in an unblinding event will be recorded and reported by the IWRS to the Medical Monitor and the Sponsor.

In case of IWRS failure, a backup system is operated 24 hours every day of the year by the Sponsor enabling unblinding of treatment by calling the following universal toll free DBV Technologies numbers:

Germany and Ireland:	00 800 0800 3456
Canada and USA:	(1) 844 299 0837
Australia:	0011 800 0800 3456

If the blind is broken, the date, time, and reason must be recorded in the subject's source documents, in the e-CRF and any associated AE must be reported.

If an Investigator, site personnel performing assessments, or subject (parents/guardians), is unblinded, the subject must be listed as major protocol deviation and will be withdrawn from the study.

Serious unexpected suspected adverse reactions, which are subject to expedited reporting, will be unblinded by the Sponsor before submission to the Regulatory Authorities.

The overall randomization code will be broken only for reporting purposes. This will occur once all final clinical data have been entered into the database and all data queries have been resolved, the e-CRF has been signed and locked, and the assignment of subjects to the analysis sets has been completed.

5.5 Drug Accountability

The Investigator is responsible for maintaining accurate study drug accountability records throughout the study. Each site will have to complete a site study drug accountability log and an individual study drug accountability log for each subject. These records should include the amounts and dates that study drug supplies were received on-site, dispensed to the subject, returned by the subject, and returned to study drug distributor (or destroyed on-site, if applicable).

Each dispensing of study drug will be documented in the e-CRF via IWRS.

5.6 Compliance

It is the Investigators' responsibility to ensure that subjects (parents/guardians) are correctly instructed on how to store and administer the study drug. Records of study drug used and intervals between visits will be kept during the study. Drug accountability will be monitored by PAREXEL's site monitor during site monitoring visits and at the completion of the study. Subjects (parents/guardians) will be asked to return their unused study drug (boxes) when they come back for their study visits. All unused study drug (boxes) should be returned at the end of the study to the study drug distributor. The study drug should be dispensed by the Investigator, or by a qualified individual under the Investigator's supervision. An up-to-date treatment inventory/dispensing record must be maintained (see Section 5.5).

At each visit, prior to dispensing the study drug, previously dispensed study drug will be retrieved by the Investigator and compliance assessed. A global compliance of ≥80% over the whole treatment period is sought. Global compliance is defined as the total number of patches applied in the treatment period versus the number of days in that period of time. Compliance at each visit is to be calculated taking into account the total number of patches applied since the last visit versus the number of days in that period of time. Subjects exhibiting poor compliance (below 80%) at any specific time point during the treatment period should be counseled on the importance of good compliance to the study dosing regimen.

Subjects who are persistently non-compliant will be withdrawn from the study.

5.7 Viaskin® Patch Adhesion

An evaluation of the Viaskin® patch adhesion will be conducted. The assessment of the patch adhesion to the skin will consist of a 28-day specific analysis to occur between Month 3 and Month 6 (whenever possible, these should be consecutive days). This assessment period can be extended up to Month 9 in case it has not been completed between Month 3 and Month 6 (see Section 6.2.9).

5.8 Prior and Concomitant Medication

Any medication the subject takes other than the study drug, including herbal and other non-traditional remedies, is considered as concomitant medication. Any medication used in the last 6 months or being administered at the time of screening is considered as prior medication.

At screening, subjects will be asked what medications they have been taking during the last 6 months. At each subsequent study visit, subjects will be asked what concomitant medications they are currently taking. All concomitant and prior medications must be recorded in the e-CRF.

The following information must be recorded in the e-CRF for each prior and concomitant medication: generic name, route of administration, start date, stop date, dosage, total daily dose, and indication. Any changes in the dosage or regimen of a concomitant medication must be recorded in the e-CRF.

Of note, prior or concomitant medication prescribed to a subject but not actually taken or administered should not be reported in the e-CRF, such as the epinephrine auto-injector prescribed for use in case of anaphylaxis but never injected intramuscularly.

5.8.1 Permitted Concomitant Medication

Application of a topical corticosteroid to treat any local condition (eczematous lesions, pruritus, edema, etc.) is allowed and should be recorded as concomitant medication. For local reactions to the Viaskin® patch, a topical medication (ointment, gel, cream) with 1% hydrocortisone or equivalent will be distributed to each randomized subject at discharge on Day 1. In case the 1% hydrocortisone topical medication is not sufficient to treat the local reaction, a topical medication containing a more potent corticosteroid can be prescribed and locally applied.

Oral antihistamines or oral corticosteroids are allowed to treat conditions determined as being allergic reactions and should be recorded as concomitant medications. These treatments should be limited in duration and stopped as soon as the condition has resolved. The Investigator will determine the best choice of treatment, the dose and the regimen according to the subject's age, and the type and the degree of severity of the reactions. Cetirizine is recommended as the oral antihistamine of choice.

Intramuscularly injectable epinephrine (EpiPen® or Twinject® or AnaPen®, or any other trade name available at the right dosage in the different countries) (auto-injector) will be distributed to each subject at discharge on Day 1 to be used in case of symptoms of anaphylaxis. The Investigator will explain to the subject/parents/guardians when and how to (self) inject the epinephrine according to the Anaphylaxis Emergency Action Plan which will also be given to the subject. The intramuscularly injectable epinephrine will be replaced if used or if it expires. Any use of injectable epinephrine should be recorded as a concomitant medication.

All other treatments prescribed by the Investigator or any other physician to treat any conditions are also permitted. Medications that are not noted in the Section 5.8.2 are also permitted.

5.8.2 Prohibited Prior and Concomitant Medication

Prohibited prior and concomitant medications as outlined in the exclusion criteria (Section 4.2) are the following:

- Short-acting antihistamines or long-acting antihistamines taken must be washed out for the minimum period of time specified in the table in APPENDIX 4 prior to the skin prick testing or to the food challenges;
- Treatment with a high daily dose of inhaled corticosteroid or treatment with a combination therapy of a medium daily dose of inhaled corticosteroid with a long-acting inhaled β2-agonist or treatment with a combination therapy of a high daily dose of inhaled corticosteroid with a long-acting inhaled β2-agonist (a list of dosages of inhaled corticosteroids is provided in APPENDIX 2.
- Two or more systemic corticosteroid courses for asthma taken in the year prior to Visit 1 or 1 oral corticosteroid course for asthma taken within 3 months prior to Visit 1, or during screening period (unless used to treat symptoms triggered by the DBPCFC);
- β-blocking agents, angiotensin-converting enzyme inhibitors, angiotensin-receptor blockers, calcium channel blockers or tricyclic antidepressant therapy, during the screening period or during study participation;
- Anti-tumor necrosis factor drugs or anti-IgE drugs (such as omalizumab) or any biologic immunomodulatory therapy taken within 1 year prior to Visit 1, during screening period or during study participation;
- Cyclosporine or other immunosuppressive agents used within 1 year prior to Visit 1, during the screening period or during the study participation. Topical calcineurin inhibitors are permitted,
- Systemic long-acting corticosteroids used within 12 weeks prior to Visit 1 and/or systemic short-acting corticosteroids used within 4 weeks prior to Visit 1 or during screening (unless used for DBPCFC symptoms);
- Any prior or concomitant immunotherapy administered to any food (for example EPIT or OIT or SLIT or specific oral tolerance induction);
- Any aeroallergen immunotherapy administered during study participation.

6 VARIABLES AND METHODS OF ASSESSMENT

The efficacy, safety and exploratory endpoints are listed in Section 3.2. The Schedule of Procedures is provided in Table 7.

6.1 Efficacy Variables

6.1.1 Response to Treatment – DBPCFC to Peanut

The DBPCFC to peanut will be performed at study entry and post-treatment at Month 12 in order to assess the primary efficacy endpoint (percentage of treatment responders in the active Viaskin® Peanut 250 µg group compared to the placebo group), the main secondary efficacy endpoint (percentage of treatment responders in the active Viaskin® Peanut 250 µg group compared to the placebo group in each screening ED stratum), as well as the other secondary efficacy endpoints (mean and median cumulative reactive/ED of peanut protein and change from baseline at Month 12; percentage of subjects responsive/unresponsive to a cumulative dose ≥1,444 mg peanut protein at Month 12; percentage of subjects unresponsive to the highest cumulative dose of peanut protein at Month 12).

The DBPCFC is the gold standard to diagnose and assess food allergy. The challenges will occur over 2 days; the subject will be gradually fed increasing amounts of standardized blinded oral formulas containing either peanut protein during one of the two days of the challenge, or without any peanut protein during the other day of the challenge, or vice et versa. Challenges must take place under direct medical supervision in a hospital/clinic setting with resuscitation equipment and emergency medications and staff immediately available. An IV line can be established prior to the challenge when judged necessary by the Investigator. In that case, a local anesthetic cream can be used for establishing the IV line. If a site prefers to have the subjects come to the hospital the day before the conduct of the DBPCFC, to get them ready for the following day, this is allowed and will not be considered an SAE.

Subjects should wash out any antihistamines for the minimum period of time specified in the table in APPENDIX 4) prior to performing the DBPCFC.

Subjects will not be allowed to use long-acting β2-agonists within 36 hours prior to the DBPCFC. Subjects who received more than a 3-day course of systemic corticosteroids within 4 weeks of the DBPCFC should have the DBPCFC delayed to allow for 4 weeks of corticosteroid wash-out. If a subject has a reaction during the first day of the DBPCFC that requires treatment with systemic corticosteroids on the first day, the subject has to wait at least 3 days before performing the second day of the DBPCFC.

The subject should have a light breakfast and may drink water at home at least 2 hours before starting the DBPCFC at the site. During the conduct of the challenge, no food other than the challenge formulas should be consumed by the subject. Drinking a sip of water to help swallow the formulas during the challenge, if necessary, is allowed.

After the last dose of the challenge formula is administered, the medical staff should wait at least 1 hour before feeding the subject with any other food and/or water. This first feeding should be light.

A detailed and study-specific Manual of Procedures for the conduct of the DBPCFC will be provided to the Investigators, the site staff and the unblinded study-trained person responsible for reconstituting the formulas. When needed, updated versions will be made available. An outline of the procedures is specified below.

6.1.1.1 Preparation of Peanut and Placebo Formulas

Standardized formulas, centrally produced by AMATSI, Montpellier, France, and packed and released by CREAPHARM, Bordeaux, France, will be distributed to all participating centers for the DBPCFC. One of the formulas will be peanut-free (placebo) and the other one will contain peanut protein. An unblinded person identified at each site by the investigator and specifically trained to reconstitute the formulas in the study and not involved in the challenge itself will be responsible for the preparation, adequate labeling and blinding of the pots containing the prepared/hydrated formulas. The blinded pots will then be handed to the medical team to perform the challenge.

6.1.1.2 Time Interval and Doses

The order of the formulas to be consumed during the first and the second day of the DBPCFC will be determined at random by the unblinded study-trained person preparing the formula using a specific randomization list provided to her/him for this purpose. Other members of the site staff (for example the Investigator, study coordinators, and study nurses), the subject/parents/guardians will remain blinded to the order of consumption until the end of the second day of the DBPCFC. Up to 7 days (1 week) between the 2 days of the DBPCFC will be permitted. The 2 days of the DBPCFC may be 2 consecutive days, but not the same day.

The challenge will consist on giving doses of peanut protein or placebo in gradually increasing doses at 30-minute intervals. This standard interval has been used safely in the past, but the Investigator may use clinical judgment to increase the intervals between doses if there is a concern that an objective reaction may be developing.

The starting dose is 1 mg of peanut protein for the entry/screening DBPCFC as well as the post-treatment DBPCFC; the maximum dose is 300 mg of peanut protein for the entry/screening DBPCFC and 2,000 mg for the post-treatment DBPCFC.

The peanut protein dose increments for the entry/screening DBPCFC are 1 mg, 3 mg, 10 mg, 30 mg, 100 mg, and 300 mg.

The entry/screening DBPCFC challenge is stopped at the 300 mg dose as a maximum, regardless of whether a reaction occurred before or not.

The peanut protein dose increments for the post-treatment DBPCFC are 1 mg, 3 mg, 10 mg, 30 mg, 100 mg, 300 mg, 1,000 mg and 2,000 mg.

6.1.1.3 Entry/screening DBPCFC to Peanut

Subjects who have not had an objective IgE-mediated reaction leading to stopping the DBPCFC at ≤300 mg peanut protein will be considered not allergic enough and thus ineligible for this study. Hence, the entry/screening DBPCFC is stopped after the 300 mg dose of placebo or peanut has been dispensed on both days, even if there have been no objective reactions.

Subjects will receive 1 formula (placebo or peanut) on the first day and the other formula (peanut or placebo) on the second day. All subjects will undergo both days of the DBPCFC. The second day of a challenge might not be performed if the subject reacted severely or seriously during the first day of the challenge, leaving no doubt about the nature of the formula administered, which is peanut (i.e. need for intubation, hypotension persisting after epinephrine administration, and/or the need for ≥3 doses of epinephrine). In this rare case and in this case only, the formula will be unblinded to confirm that the subject received the peanut formula.

At the end of the second day of challenge, the sequence of the 2 formulas given on the 2 days will be unblinded and revealed to the medical staff by the unblinded study-trained person responsible for reconstituting the formulas and the results of the challenge will be established following the decision making algorithm below:

1. The subject had objective IgE-mediated symptoms to the placebo formula at any dose of the challenge. She/he cannot be randomized in the study;
2. The subject had no objective IgE-mediated symptoms during the 2 days of the DBPCFC, neither to the placebo nor to the peanut formulas, even at the 300 mg dose of peanut protein. She/he cannot be randomized in the study;
3. The subject had no objective symptom when receiving placebo but had an objective symptom at 1 of the doses of the peanut formula consumed between 1 mg and 300 mg inclusive. She/he will be randomized in the study in the screening ED stratum that corresponds to the subject's ED.

Only **clear-cut OBJECTIVE immediate-type symptom(s) requiring treatment** will be considered as a reason to stop the challenge and to determine the eliciting dose (highest dose given during the challenge) as well as the Cumulative Reactive Dose (CRD) of peanut protein. **Subjective symptoms will be graded but shall not count to stop the challenge.**

The OFC Symptom Score Sheet in the protocol APPENDIX 6 will be used to score the severity of each symptom (classified under the 5 categories: Skin, Upper respiratory, Lower Respiratory, Gastrointestinal and Cardiovascular/Neurologic).

More specifically, the food challenge must be stopped if:

- There is ≥1-point rise in an objective symptom from 1 category in the OFC Symptom Score Sheet with the following exceptions:

  The followings require a >1-point rise to stop the challenge if present alone
  - Urticaria
  - Rash
  - Laryngeal (if explained and not persistent)

  The followings require a >2-point rise to stop the challenge if present alone:
  - Pruritus
  - Sneezing/Itching
  - Nasal Congestion
  - Rhinorrhea
  - Conjunctivitis

- There is ≥1-point rise in an objective symptom from ≥2 categories in the OFC Symptom Score Sheet with the following exceptions:

  The following exceptions require a ≥2 point rise in one of these symptoms plus a ≥1-point from another category:
  - Pruritus
  - Sneezing/Itching
  - Nasal Congestion
  - Rhinorrhea
  - Conjunctivitis

Conjunctivitis (erythema of the conjunctiva) is not mentioned in the OFC Symptom Score Sheet but is an objective symptom that could appear during the challenges and is pre-specified in the e-CRF. Please also report in the e-CRF with a grade when this occurs, together with any other symptoms that could occur during the challenge.

In case of symptoms that develop but do not meet stopping rules, consider allowing 30 additional minutes to see if the reactions subside or progress to meet stopping criteria. You can also repeat the dose or step up at your discretion.

Further details are provided in the study-specific Manual of Procedures for the DBPCFC that will be provided to the sites.

In case of subjective symptoms, for example mouth pruritus, throat pruritus, nausea, abdominal pains or any other subjective symptoms, the severity of which might signify that objective symptoms are developing according to the Investigator’s judgment, the time between the previous dose and the next dose might be extended to see how the subject’s symptoms evolve. The same dose of peanut protein can be repeated to check whether the subjective symptoms reappear or not and with what severity, or whether objective symptoms now appear. If objective symptoms meeting stopping rules appear, they should be treated and the food challenge will be stopped. The dose given that

triggers the objective symptoms is considered the ED. If only subjective symptoms persist even after the repeated dose, the next planned peanut protein dose is then given to the subject, and the challenge should continue until the appearance of clear objective symptoms meeting stopping rules, at which time the challenge will be stopped and the symptoms treated.

As a safety precaution, the objective symptoms signaling the end of the DBPCFC will be treated. The medication used will be left to the Investigator’s judgment.

The Investigator and medical staff will use their own clinical judgment for the most effective treatment to give to the subject considering her/his age, the type of the allergic reactions and their severity. Also refer to recommendations made by Sampson et al. for treating anaphylaxis (42).

Suggested treatments for the different objective symptoms are detailed in the study-specific Manual of Procedures for the DBPCFC provided to the sites.

Should epinephrine need to be administered, it should be injected intramuscularly in the anterolateral thigh using auto-injectors commercially available in each specific site/country. Intravenous epinephrine should NOT be considered at the investigative sites to treat the reactions.

Subjects will be kept under observation for an additional 3 hours after the ingestion of the last dose of the challenge formula. Based on the Investigator’s judgment, the observation period could be extended beyond the first 3 hours to ensure that all symptoms have subsided before the subject is discharged. For instance, an overnight stay may be considered necessary by the Investigator if the symptoms have not completely resolved within the 3 hours or if the symptoms have been severe or serious and require longer observation periods.

Complete information for all reactions will be reported first in source documents then in the e-CRF, along with doses given, symptoms observed and their highest grades, time of appearance of the symptoms and an Investigator’s assessment of the ED.

6.1.1.4 Post-treatment DBPCFC to Peanut at Month 12

The DBPCFC at Month 12 is conducted following exactly the same procedures as for the entry/screening DBPCFC. However, it is not stopped at the 300 mg dose. The challenge must instead be continued up to the last dose of 2,000 mg peanut protein or until objective reactions leading to stopping the DBPCFC occur.

Only **clear-cut OBJECTIVE immediate-type symptom(s) requiring treatment** will be considered as a permissible reason to stop the challenge and to determine the eliciting dose (highest dose given during the challenge) as well as the Cumulative Reactive Dose (CRD) of peanut protein. **Subjective symptoms will be graded but shall not count to stop the challenge.** The same instructions provided above to stop the challenge at the entry challenge will be followed to stop the challenge post-treatment.

During the duration of the study, DBPCFCs to other foods cannot be conducted for any randomized subject.

6.2 Safety Variables

6.2.1 Adverse Events

Coding of AEs will be performed as described in Section 9.4.

6.2.1.1 Collection of Adverse Events

It is the responsibility of the Investigator to collect all AEs (both serious and non-serious) derived by spontaneous, unsolicited reports of subjects, by observation and by routine open questionings, for example "How have you felt since I last saw you?".

6.2.1.2 Definitions

An AE is any untoward medical-occurrence that occurs in a patient or clinical investigation subject administered a pharmaceutical product, and which does not necessarily have to have a causal relationship with this treatment. An AE can therefore be any unfavorable and unintended sign (including abnormal laboratory finding), symptom, or disease temporally associated with the use of an investigational product, whether or not considered related to the product.

All AEs, including intercurrent illnesses, occurring during the study will be documented in the e-CRF. Concomitant illnesses, which existed before entry into the study, will not be considered AEs unless they worsen during the treatment period. All AEs, regardless of the source of identification (for example physical examination, laboratory assessment, electrocardiogram [ECG], reported by subject), must be documented.

Pre-existing conditions will be recorded in the e-CRF on the Medical History or appropriate page.

A TEAE will be defined as an AE that begins or that worsens in severity after at least 1 dose of study drug has been administered.

A pretreatment-emergent AE will be defined as an AE that begins or that worsens in severity after the first screening visit, but before the first dose of study drug has been administered.

AEs and TEAEs due to DBPCFC will also be recorded in the e-CRF but will be analyzed separately.

6.2.1.3 Assessment of Adverse Events

Each AE will be assessed by the Investigator with regard to the following categories.

6.2.1.3.1 Seriousness

An SAE is defined as any untoward medical occurrence that at any dose:

- Results in death;
- Is life-threatening; This means that the subject is at risk of death at the time of the event; it does not mean that the event hypothetically might have caused death if it were more severe;
- Requires hospitalization (overnight or longer) or prolongation of existing hospitalization;
- Results in persistent or significant disability or incapacity;

- Is a congenital anomaly or birth defect;
- Is an important medical event that may not be immediately life-threatening or result in death or hospitalization but that may jeopardize the subject or require intervention to prevent one of the above outcomes. Examples of such events are intensive treatment in an emergency room or at home for allergic bronchospasm; blood dyscrasias or convulsions that do not result in hospitalization; or development of drug dependency or drug abuse.

Medical and scientific judgment should be exercised in deciding whether a case is serious and whether expedited reporting is appropriate.

Events associated with hospitalization for the following will not be considered as an SAE:

1. Evaluation or treatment of a pre-existing and non-exacerbating condition as long as the condition is associated with the hospitalization:
    a. Existed prior to the subject’s entry into the study and has been recorded in the subject’s disease/medical history as documented in the e-CRF,
    b. Has not worsened in severity or frequency during the subject’s exposure to study drug,
    c. Has not required a change in treatment management during the subject’s exposure to the study drug;
2. Elective or pre-planned treatment of a pre-existing and non-exacerbating condition;
3. Early hospitalization of a subject the day prior to the day of DBPCFC in order to have the subject prepared in advance.

6.2.1.3.2 Severity

The severity of each AE must be assessed by the Investigator using 1 of the following categories, and recorded in the e-CRF:

- Mild: The AE was transient and easily tolerated by the subject;
- Moderate: The AE caused discomfort and interference with the subject's general condition;
- Severe: The AE caused considerable interference with the subject's general condition and may have been incapacitating.

6.2.1.3.3 Causality

The Investigator will assess the causality/relationship between the study drug and the AE and record that assessment in the source documents and in the e-CRF.

The most likely cause of an AE/SAE (for example disease under treatment, concomitant disease, concomitant medication, other) will be indicated in the e-CRF with details of the concomitant disease or medication or other cause.

The causal relationship of the AE to study drug will be described in terms of:

- Related: the AE:
    - Follows a clear temporal sequence from administration of the study drug.

- Has no other possible explanations, such as the subject's clinical state, environmental or toxic factors or other therapies administrated to the subject.
- Disappears or decreases on cessation or reduction in dose of the study drug.
- Follows a clear pattern of response to the study drug.
- Reappears or worsens upon rechallenge.

- Probable: the AE:
  - Follows a reasonable temporal sequence from administration of the study drug.
  - Could not be reasonably explained by the subject's clinical state, environmental or toxic factors or other therapies administrated to the subject.
  - Disappears or decreases on cessation or reduction in dose of the study drug.
  - Follows a known pattern of response to the study drug.
  - Reappears or worsens upon rechallenge.
- Possible: the AE:
  - Follows a reasonable temporal sequence from administration of the study drug.
  - Could be reasonably explained by the subject's clinical state, environmental or toxic factors or other therapies administrated to the subject.
  - Follows a known pattern of response to the study drug.
- Unlikely: the AE
  - Does not follow a reasonable temporal sequence from administration of the study drug.
  - Could be reasonably explained by the subject's clinical state, environmental or toxic factors or other therapies administrated to the subject.
  - Does not follow a known pattern of response to the study drug.
  - Does not reappear or worsen upon rechallenge.
- Not related:
  - The AE does not meet the above criteria.
  - There is sufficient information that the etiology of the AE is not related to the study drug.

The study conduct relatedness for SAEs will also be assessed and documented.

6.2.1.3.4 Local Skin Reactions

The incidence, duration and maximum severity of skin reactions induced by the study drug will be reported by the subject (parents/guardians) in the diary. Photos of these local reactions should be taken at home by the parents/guardians. Additionally, the Investigator will assess the severity of local skin reactions induced by the study drug at each site visit during the physical examination and photos should be taken during the site visits by the medical staff to document these local skin reactions (see Sections 6.2.4 and 6.2.8).

6.2.1.3.5 Adverse Events of Special Interest

Adverse events of special interest to Sponsor in this study include grade 4 local skin reactions seen under the patch while the patch is still applied on the skin or upon removal of the patch from the skin. Skin reactions will be examined at the time points specified in the Schedule of Procedures (Table 7) according to grading in Table 6 (Section 6.2.8). Specifically, the appearance of any vesicles or ulcerative skin lesions or any other significant skin lesion which could potentially lead to skin barrier disruption at sites of Viaskin® patch applications will be considered AESI. Subjects (parents/guardians) will be instructed to take a photograph in case of appearance of any vesicles or ulcerative skin lesions. In these rare specific cases, subjects should transiently discontinue patch application and return to the site for evaluation and treatment of the wounded area, as well as for the next patch application and adequate evaluation and treatment of the wounded zone.

Upon re-application of the new patch, the subject should remain at the site for 1 hour before being discharged. The site must contact the subject (parents/guardians) by phone the day after this visit to ensure that no additional local blisters or vesicles developed and to confirm that the treatment can continue normally.

Any occurrence of IgE-mediated systemic-type of symptoms distant from the patch application site and considered at least possibly related to the study drug will also be considered as AESI and analyzed specifically as such at the end of the study at the time of the final analysis of study results.

6.2.1.3.6 Symptoms during to the Double-blind, Placebo-controlled Food Challenge

The severity of the objective and subjective symptoms elicited during the entry and post-treatment DBPCFC will be assessed by the Investigator. These objective and subjective symptoms assessed by the Investigator will not be reported in the AE pages of the e-CRF. Complete information for all these symptoms will be reported in the DBPCFC pages of the e-CRF, along with all peanut protein doses given, the symptoms observed and their highest grades, time of appearance of the symptoms, the doses of epinephrine with an auto-injector given, the doses of corticosteroids and antihistamines given and the Investigator's assessment of the peanut ED as well as the peanut cumulative dose. If, as per the Investigator's judgment, the combination of all these symptoms resulted in an anaphylaxis requiring a prolonged (minimally overnight) hospitalization, then this will be considered an SAE. Any such SAE with its specific verbatim must be reported in the AE pages of the e-CRF.

6.2.1.4 Recording Adverse Events

Adverse event reporting will start at the signature of the informed consent form (ICF) and end after the 2-week follow-up period (End of Study V12 Visit). During the screening period (that is, from the time of ICF signature through Day-1), only SAEs will be recorded in the e-CRF; during the screening period, AEs that do not meet any seriousness criteria will be documented in the subject's source documents. Adverse events occurring after the end of the study should be reported to the Sponsor by the Investigator if the Investigator considers that there is a causal relationship with the study drug.

Adverse events still ongoing at the time of the End of Study Visit will be followed up for an additional 30 days, or until they resolve or stabilize, whichever comes first.

All AEs occurring on or after the day of the first dose of study drug, regardless of the relationship to the study drug, will be recorded in the e-CRF.

All AE reports in the e-CRF should contain the following information of the event: date and time of onset, date and time of resolution, severity, treatment required, relationship to study drug, action taken with the study drug, outcome, and whether the event is classified as serious or not.

6.2.1.5 Reporting Serious Adverse Events

All SAEs must be reported within 24 hours of the site becoming aware of the event by filling in the SAE pages in the e-CRF. In case of technical issues with the e-CRF, the reporting can be done by faxing a completed SAE Report Form to the PAREXEL safety center at the following FAX numbers:

- Centers in Europe and Australia dial the following FAX numbers:

+33 1 44 90 32 75 or<br>+33 1 44 90 35 34

- Centers in North America dial the following FAX number:

+1 781 434 5957

The minimum information required for an initial report is:

- Name of person sending the report (that is name and address of Investigator);
- Subject identification (screening number, NOT the subject's name);
- Protocol number;
- Description of the SAE including a comprehensive verbatim term;
- Causality assessment, if possible.

However, as far as possible all points in the SAE pages in the e-CRF (or on the SAE Report Form, in case of technical issues with the e-CRF) should be covered in the initial report. If an SAE occurs during the DBPCFC, the verbatim of the SAE must specify "during the DBPCFC" or "due to the DBPCFC" both on the SAE Report Form and in the SAE pages of the e-CRF.

6.2.1.6 Follow-up of Adverse Events

All AEs experienced by a subject, irrespective of the suspected causality, will be monitored until the AE has resolved, any abnormal laboratory values have returned to baseline or stabilized at a level acceptable to the Investigator and Medical Monitor, until there is a satisfactory explanation for the changes observed, until the subject is lost to follow-up, or until the subject has died.

6.2.1.7 Pregnancy

Pregnancy will be determined by evaluation of urine pregnancy tests. Subjects who are pregnant at screening are excluded from the study. Subjects who become pregnant during treatment must be discontinued from the study.

The Sponsor has a responsibility to monitor the outcome of pregnancies where there has been maternal exposure to the study drug.

Pregnancy alone is not regarded as an AE unless there is a suspicion that the study drug may have interfered with the effectiveness of a contraceptive medication.

Elective abortions without complications should not be handled as AEs, unless they were therapeutic abortions (see below). Hospitalization for normal delivery of a healthy newborn will not be considered a SAE.

All pregnancies must be reported by the Investigator to PAREXEL/Sponsor in the specific pregnancy pages in the e-CRF within 30 days after becoming aware of the pregnancy. The Investigator must follow up and document the course and the outcome of all pregnancies even if the subject was discontinued from the study or if the study has finished.

All outcomes of pregnancy must be reported by the Investigator to PAREXEL/Sponsor in the specific pregnancy pages in the e-CRF within 30 days after she/he has gained knowledge of the normal delivery or elective abortion.

Any SAE that occurs during pregnancy (including SAEs occurring after last administration of study drug) must be recorded in the SAE pages in the e-CRF (for example maternal serious complications, spontaneous or therapeutic abortion, ectopic pregnancy, stillbirth, neonatal death, congenital anomaly, or birth defect) and reported within 24 hours in accordance with the procedure for reporting SAEs.

6.2.1.8 Treatment of Overdose of Study Medication

Overdose is defined as the concomitant application of 2 Viaskin® patches or more on the skin of the subject, whatever the duration of the concomitance of the multiple patch application.

One patch applied the same day after the previous patch was removed or has fallen off is NOT an overdose.

There has been no experience of overdosing with Viaskin® patches so far. No specific treatment for overdosing is known. The first action will be to remove any additional patch from the skin, leaving only 1 patch on the skin Treatment given to a subject in case of overdosing should be symptomatic and supportive.

Any case of overdose, with or without associated AEs, must be reported to PAREXEL. Overdose will be reported in the SAE pages of the e-CRF (with Overdose as event term)

within 24 hours of learning of the overdose. In case of technical issues with the e-CRF, this can be done by faxing a completed SAE Report Form to the PAREXEL safety center. Any AEs associated with the overdose should be reported in the relevant AE/SAE sections in the e-CRF.

6.2.2 Laboratory Variables

Laboratory assessments will be performed by a central laboratory, as identified in the List of Study Personnel.

Venous blood samples will be taken for hematology and biochemistry testing.

The following laboratory variables will be determined in accordance with the Schedule of Procedures (Table 7):

- Hematology: hemoglobin, hematocrit, platelets, red blood cells, white blood cells with differential cell count;
- Biochemistry: alanine aminotransferase, aspartate aminotransferase, total bilirubin, total protein, blood urea nitrogen, creatinine;
- A urine pregnancy test will be performed at the site.

Details of the procedures to be followed for sample collection, storage, and shipment will be documented in the Laboratory Manual.

Clinically significant changes (abnormalities) in laboratory parameters, in the judgment of the Investigator, will be recorded as AEs and appropriate countermeasures taken.

In the event of unexplained abnormal laboratory test values of clinical significance, the tests should be repeated at a reasonable time point and followed up until they have returned to the normal range and/or an adequate explanation of the abnormality is found.

Additional and repeat laboratory safety testing outside the study may be performed at the discretion of the Investigator.

6.2.3 Vital Signs

The following vital signs will be assessed in accordance with the Schedule of Procedures (Table 7):

- Blood pressure (systolic and diastolic; mmHg),
- Heart rate (beats per minute),
- Respiration rate (breaths per minute).

Systolic blood pressure and diastolic blood pressure will be measured in sitting position on the same arm after the subject has been resting.

Heart rate will be recorded simultaneously with blood pressure measurements, followed by respiratory rate.

During the study, the measurement of vital signs may be repeated at the discretion of the Investigator for safety reasons. Clinically relevant abnormal findings will be reported as AEs.

Vital signs must be assessed before the DBPCFC. Additional assessments can be repeated during the DBPCFC procedure on both days at any time if judged necessary by the Investigator.

6.2.4 Physical Examinations

Physical examinations will be performed in accordance with the Schedule of Procedures (Table 7).

Physical examinations will be performed by a physician or a master level clinician qualified for performing physical examinations (Nurse Practitioners or physician assistants) and will include examination of the following:

- General appearance,
- Head, ears, eyes, nose and throat,
- Neck,
- Complete skin examination,
- Cardiovascular system,
- Respiratory system,
- Abdominal system,
- Nervous system,
- Body weight (kg),
- Height (cm).

Body weight will be measured without shoes, jacket, or diaper and per Schedule of Procedures and as frequently as necessary.

Height will be measured without shoes and per Schedule of Procedures and as frequently as necessary for this population of subjects in active growth, especially for the pulmonary function tests (spirometry, PEF measurements).

For each body system, an assessment of normal or abnormal will be recorded in the e-CRF at screening and the abnormality will be documented. Besides, the skin aspect will be graded for the sites of patch application (see Section 6.2.8).

Physical examinations must be performed before the DBPCFC. Additional assessments can be repeated during the DBPCFC procedure on both days at any time judged necessary by the Investigator.

Clinically significant changes (abnormalities) in physical examination findings, in the judgment of the Investigator, will be recorded as AEs and appropriate countermeasures taken.

6.2.5 Spirometry Test

For subjects ≥ 6 years of age (unless they have documented inability to adequately perform spirometry), FEV_1 will be measured on a standardized calibrated spirometer following the ATS guidelines. At least 3 acceptable FEV_1 maneuvers will be obtained and the highest of the 3 attempts will be recorded in the e-CRF.

6.2.6 Peak Expiratory Flow

For all the subjects ≥5 years of age, PEF will be measured on a peak flow meter. At least 3 acceptable measures will be obtained and the highest of the 3 attempts will be reported in the e-CRF. The PEF assessment must be performed at all visits. On the days where a

challenge is performed the PEF assessment must be performed just before the DBPCFC. This can be repeated during the DBPCFC if needed.

For subjects 4 years of age at Visit 1, PEF will not be requested.

6.2.7 Subject Diaries

Subject diaries will be provided to each subject to report specific information between visits, as follows. Subjects and/or their parents/guardians will be asked to record on a daily basis in their diaries the time of application and removal of each Viaskin® patch, along with the reason for any early removal (should that occur). They will also record the grade of any observed local skin reactions to the Viaskin® patch. Evaluation of local skin reactions (pruritus, erythema and edema) will be reported daily by the subjects (parents/guardians) in their diaries for6 months. Subjects (parents/guardians) will also be instructed to record in their diaries any other AEs, local skin reactions occurring after the 6 months of treatment and any concomitant medication or treatment taken for any type of AEs. The subject diary will be reviewed by the site medical staff at each patient visit.

In addition, the adhesion of the Viaskin® patch to the skin will be assessed by the parents/guardians for 28 days of treatment between Month 3 and Month 6 (whenever possible, these should be consecutive days) and the assessment noted in a specific diary. Assessment may be continued during Month 6 and Month 9 to complete the evaluations started but not completed between Month 3 and Month 6. Assessment may also be done between Month 6 and Month 9 in some circumstances, especially when not adequately performed between Month 3 and Month 6. A specific scoring system to assess the maintenance of the occlusion of the Viaskin® patches after 24 hours of application is described in Section 6.2.9. This scoring system will be explained to the parents/guardians to ensure good assessment of patch adhesion. If the patch has been removed deliberately prior to the 24 hours of application for safety reasons or for subject's personal reasons on a specific day, the adhesion score of that specific day should also be reported, even though this will not count as an evaluable day of assessment for the adhesion evaluation. More days of assessment would then be needed to compensate for such cases and ensure that 28 evaluable days have been recorded.

Subjects must bring their diary back to the Investigator at each visit, and the Investigator must check the diary for completeness and accuracy. It is the Investigators' responsibility to instruct the subjects and/or parents/guardians about the use of the diary, and to ensure that the diary is completed accurately. Any problems with completing the diary will be addressed with the subjects and/or parents/guardians. At each site visit, the completed pages of the diary will be collected and kept by the Investigators. The remaining blank pages of the diary will be given back to the subject at each visit before discharge. All diaries must be returned to the site at completion of the study, or if the subject discontinues.

6.2.8 Skin Reaction and Photography

Local skin reactions under the Viaskin® patch or on any of the previous sites of patch application will be graded as a whole at each visit according to the recommendations of the European Academy of Allergology and Clinical Immunology (EAACI) and the

Global Allergy and Asthma European Network (GA^2LEN) (43), and modified as follows:

Table 6: Skin Reaction Grading System

Skin Reaction	Grade if localized under the patch	Grade if extending beyond the patch
Negative	Grade 0	Grade 0
Only erythema, or erythema + infiltration	Grade 1A	Grade 1B
Erythema, few papules	Grade 2A	Grade 2B
Erythema, many or spreading papules	Grade 3A	Grade 3B
Erythema, vesicles	Grade 4A	Grade 4B

The Viaskin® patch is transparent and the degree of the local reactions under the Viaskin® patch can be easily seen through the patch. Grading of the local skin reactions on the back will start being recorded at Visit 4 right after the Viaskin® patch has been applied and while the subject is kept at site under observation and subsequently when subjects arrive at site for their further visits.

At Visit 4, observations should be recorded at 30 minutes, 1 hour, 2 hours, and 3 hours after the Viaskin® patch is applied (through the patch, without removing it).

Photographic records of the sites of application of the Viaskin® patch will be taken and filed in the subject's medical records or source documents. The subject's face will not, at any time, be captured in the photograph. Photography acquisition guidelines will be provided separately to the sites, so that photography process can be standardized as much as possible. All the photographs will be downloaded by the site staff onto a specific PEPITES website with a restrictive access to the site staff members, the Sponsor members and Sponsor representatives.

Photographic records will be made available to the DSMB members upon request.

6.2.9 Viaskin®Patch Adhesion

To ensure that each Viaskin® patch adheres well on the subject's skin for 24 hours and that occlusion of the condensation chamber is guaranteed over the 24 hours of application of each patch, the parents/guardians will be asked to take photographs and assess these parameters for 28 days between Month 3 and Month 6 (or up to Month 9 if necessary; whenever possible, these days of assessment should be as consecutive as possible).

The scoring system for adhesion and occlusion of Viaskin® patches is indicated as follows:

- 0=No lift off or detachment of the dressing edges and the occlusion chamber of the patch is intact.
- 1=Some edges of the dressing have lifted off the skin with no impact on the occlusion chamber of the patch which remains fully adherent to the skin.
- 2=The occlusion chamber of the patch is partially or totally detached.
- 3=The patch has fallen off the skin.

During those 28 days of evaluable assessment, photos and assessment of patch adhesion must be done. Even on those days when the patch has deliberately been removed

prematurely by the subject or parents/guardians, the photos and assessment should be made as recommended, even if the assessment might not be used in the analysis. That's why the assessments should extend beyond 28 days to ensure that 28 days of usable evaluations of 24 hours of application are recorded. All information relative to the patch adhesion should be collected in the corresponding diary.

Photographs and assessment of the adhesion of each patch will be made at 2 time points by the parents/guardians for 28 days:

- Immediately after the application of a patch to assess that the patch was applied as recommended and stuck well to the skin (including the edges of the dressing); and
- After 24 hours of application at time of removal of the patch.

At the time of each patch removal, the subjects (parents/guardians) will also be asked:

- To specify the suspected cause of adhesion issue (if any),
- To grade the easiness of removal of the patch (very easy/ easy / difficult / very difficult),
- To grade whether the removal of the patch was painful (very painful / painful / not painful),
- To specify whether the patch removal caused skin injury (yes / no).

Of note, 24 hours is the recommended duration of daily application. However, a duration of daily application of 24 ± 4 hours will be allowed.

To guarantee the reliability of the adhesion scoring done by the subject's parents/guardians, the Investigator will review the parents/guardians' assessment of the patch adhesion based on the photographs made by the parents/guardians during these 28 days.

If the parents/guardians did not or could not assess the patch adhesion with sufficient reliability according to the Investigator's judgment and/or if the parents/guardians did not or could not take photos for a total of 28 days between Month 3 and Month 6, the Investigator can disqualify all or part of the data collected for the adhesion assessment and will re-train the parents/guardians so that the assessment of the patch adhesion can be repeated between Month 6 and Month 9 either totally for 28 evaluable days or partially to compensate for the disqualified days of the first period of assessment.

The assessment of adhesion of the Viaskin® patch will also be made at each site visit by site staff trained specifically for this purpose. The duration of application of the assessed Viaskin® patch must be noted, photographs must be taken and the assessment by the site staff will use the same scoring system as above.

All the photographs will be downloaded by the site staff onto the PEPITES-specific dedicated website described in section 6.2.8.

6.3 Demographics and Baseline Characteristics

Demographics and baseline characteristics consist of those variables that are assessed only at screening/baseline.

6.3.1 Patient Demography

Patient demography consists of:

- Age at screening Visit 1,
- Race,
- Sex.

6.3.2 Disease History and Medical History

History and duration of peanut allergy with well-documented IgE-mediated symptoms after ingestion of peanut must be reported in the disease history form in the e-CRF.

The documentation of the complete medical history will include history and duration of any other allergies and current medical conditions, any allergic reactions (other than peanut IgE-mediated symptoms) in the previous 12 months, past or present cardiovascular, respiratory (including asthma), gastrointestinal, renal, hepatic, neurological, endocrine, lymphatic, hematologic, immunologic, dermatological (including atopy), psychiatric, developmental, and genitourinary disorders, drug and surgical history and any other diseases or disorders.

The disease and medical histories will be obtained by interviewing the subject’s parents/guardians or by inspecting her/his medical records.

For coding of disease/medical history, see Section 9.4.

6.3.3 Prior and Concomitant Medications

Previous and concomitant medications will be documented as described in Section 5.8.

6.4 Exploratory Variables

6.4.1 Immunological Markers

Venous blood samples will be drawn to assess the following immunological markers: peanut-specific IgE and IgG4 at the visits specified in the Schedule of Procedures (Table 7).

The following immunological markers will also be assessed at specific time points: IgE and IgG4 specific to peanut protein components, and IgE specific to cow’s milk, egg, house dust mites, and grass pollen.

Analysis of samples will be conducted by Q^2 Solutions Laboratory (Ex-Quintiles Laboratory) and the results at any time point after baseline will be blinded until the data are unblinded at the study end. Further details of the procedures to be followed for sample collection, storage, and shipment will be documented in the Laboratory Manual.

6.4.2 Skin Prick Test

Peanut extract plus negative saline control and positive histamine control will be used for skin prick testing. All materials (SoluPrick® solutions and ancillary materials, CE marked) will be provided free of charge centrally to all sites by the Sponsor, including a detailed procedure for conducting the SPTs.

The subjects should wash out any antihistamines for the minimum period of time (specified in the table in APPENDIX 4) prior to performing the test. Briefly, a skin Duotip II® is pressed through a small drop of the commercial extract of peanut or positive and negative controls into the epidermis of the volar surface or back of the forearm. The area will be measured for the maximum wheal diameter after 15 minutes. A tracing should be obtained by using a fine ballpoint pen. The tracing will be performed at the demarcation line for the wheal as the skin drops back to flush. Scotch or a clear transport tape should be used to lift the tracing; the tape tracing should be placed on paper and filed as part of the subject’s source documentation.

6.4.3 Food Allergy Quality of Life Questionnaire /Food Allergy Independent Measure

The FAQLQs are disease-specific health-related quality of life questionnaires for subjects with food allergy. They are considered reliable and valid instruments to measure the impact of food allergy on health-related quality of life (44). The FAIM questionnaires capture the subjects' expectation of something happening because of her/his food allergy (45).

At screening and at Month 12, subjects ≥8 years of age and all parents/guardians will complete the FAQLQ/FAIM questionnaires. Children ≥8 years of age will complete the Child Forms of the questionnaires (FAQLQ-CF, FAIM-CF) while the parents/guardians, regardless of the age of their child, will complete the Parent Forms (FAQLQ-PF, FAIM-PF).

The template questionnaires are provided in APPENDIX 7.

6.4.4 Accidental Consumption of Peanuts

Specific reactions triggered by accidental consumption of peanut and the conditions around that consumption will be collected. The related AEs will be classified and analyzed separately. Subjects (parents/guardians) will be asked to clarify whether any peanut consumption was accidental or not.

6.4.5 Epigenetic analyses

Venous blood samples will be drawn to assess the epigenetic modifications induced by the EPIT treatment with Viaskin® Peanut on the promoter regions of the genes coding for several specific proteins. The tests will be managed centrally and the results at any time point after baseline will be blinded until the data are unblinded at the study end. Detailed procedures to be followed for sample collection, storage and shipment will be documented in a specific Manual provided to the sites.

6.4.6 Genetic Screening

Samples of blood will be taken from volunteer subjects for genetic analysis of the possible mutations in the filaggrin gene. The tests will be conducted centrally. Detailed procedures to be followed for sample collection, storage and shipment will be documented in a specific Manual provided to the sites.

6.4.7 Scoring Atopic Dermatitis

The SCORAD, a scoring index of atopic dermatitis developed by the European Task Force on Atopic Dermatitis (46) will be assessed as specified in the Schedule of Procedures (Table 7).

Sections A and B must be assessed and completed by the investigator, while section C is assessed by the subject/subject's parents.

The SCORAD is provided in APPENDIX 8.

7 STUDY CONDUCT

7.1 Schedule of Procedures

The Schedule of Procedures is presented in Table 7.

Table 7: Schedule of Procedures

Study Assessments	Screening			Treatment Period													End of Study	Early Termination	Unscheduled Visit
	V1	V2	V3[1]	V4[1]	PC	V5	PC	V6	PC	V7	PC	V8	PC	V9	V10	V11	V12	ET	UV[2]
				D1	D4	D8	D22	M1	M2	M3	M4.5	M6	M7.5	M9	M12				
Time Window	D-42 (max) before V4	Any-time through D-2	Within 1 week of V2 and through D-1		±2 ds	±3 ds	±2 ds	±3 ds	±3 ds	±7 ds	±7 ds	±7 ds	±7 ds	±7 ds	±7 ds	Max 1 week after V10	V11 + 2 weeks		
Informed consent	X																		
Check eligibility (inclusion/exclusion criteria)	X[3]			X															
Disease/Medical history[4]	X																		
Parental medical history of atopy	X																		
Demographics	X																		
Physical examination[5]	X	X[6]	X[6]	X		X		X		X		X		X	X[6]	X[6]	X	X	X
Vital signs[7]	X	X[6]	X[6]	X		X		X		X		X		X	X[6]	X[6]	X	X	X
Spirometry (FEV_1)[8]	X									X		X		X	X			X	X
PEF	X	X[6]	X[6]	X		X		X		X		X		X	X[6]	X[6]		X	X
SCORAD	X									X		X			X				
SPT	X									X		X			X			X	
Immunological markers[9]	X									X		X			X			X	
Laboratory tests[10]	X									X		X			X			X	X
FAQLQ/FAIM[11]	X														X				
Filaggrin gene: informed consent - blood sampling										X[12]		X[12]			X[12]				
Pregnancy test	X														X			X	

Study Assessments	Screening			Treatment Period													End of Study	Early Termination	Unscheduled Visit
	V1	V2	V3[1]	V4[1]	PC	V5	PC	V6	PC	V7	PC	V8	PC	V9	V10	V11	V12	ET	UV[2]
				D1	D4	D8	D22	M1	M2	M3	M4.5	M6	M7.5	M9	M12				
**Time Window**	D-42 (max) before V4	Any-time through D-2	Within 1 week of V2 and through D-1		±2 ds	±3 ds	±2 ds	±3 ds	±3 ds	±7 ds	±7 ds	±7 ds	±7 ds	±7 ds	±7 ds	Max 1 week after V10	V11 + 2 weeks		
Epigenetic analyses	X									X		X			X				
DBPCFC		X	X												X	X			
**Randomization**				**X**															
Adverse events	X	X	X	X	X	X	X	X	X	X	X	X	X	X	X	X	X	X	X
Concomitant medications	X	X	X	X	X	X	X	X	X	X	X	X	X	X	X	X	X	X	X
Check for any accidental peanut consumption					X	X	X	X	X	X	X	X	X	X	X	X	X	X	X
Subject diary (dispense/check)				X	X	X	X	X		X		X		X	X	X	X	X	X
Dispense subject safety leaflet and subject identification card				X															
Dispense study drug to the subject				X		X		X		X		X		X	X[15]				X
Apply 1 Viaskin® patch at site				X															
Check the used/unused study drug dispensed to the subject						X		X		X		X		X	X	X		X	X
Check skin reaction under the patch and grade it[13]				X		X		X		X		X		X	X	X		X	X

Study Assessments	Screening			Treatment Period													End of Study	Early Termination	Unscheduled Visit
	V1	V2	V3[1]	V4[1]	PC	V5	PC	V6	PC	V7	PC	V8	PC	V9	V10	V11	V12	ET	UV[2]
				D1	D4	D8	D22	M1	M2	M3	M4.5	M6	M7.5	M9	M12				
**Time Window**	D-42 (max) before V4	Any-time through D-2	Within 1 week of V2 and through D-1		±2 ds	±3 ds	±2 ds	±3 ds	±3 ds	±7 ds	±7 ds	±7 ds	±7 ds	±7 ds	±7 ds	Max 1 week after V10	V11 + 2 weeks		
Photographs of the back where patches were applied				X		X		X		X		X		X	X	X		X	X
Patch adhesion: subject's assessment										X[14]	X[14]	X[14]	X[14]	X[14]					
Patch adhesion: Investigator's assessment				X		X		X		X		X		X	X	X			X
Dispense epinephrine auto-injector and anaphylaxis emergency action plan				X															
Review utilization of epinephrine auto-injector and anaphylaxis emergency action plan (when required)						X		X		X		X		X					
Time under observation before discharge		3 hrs	3 hrs	3 hrs											3 hrs	3 hrs			

Abbreviations: D = Day; DBPCFC = Double-blind, placebo-controlled food challenge; ds = days; ET = Early termination; FAQLQ/FAIM = Food Allergy Quality of Life Questionnaire/Food Allergy Independent Measure; FEV_1 = Forced expiratory volume in one second; hrs = hours; M = Month; PC = Phone contact; PEF = Peak expiratory flow; SCORAD = Scoring atopic dermatitis; SPT = Skin prick test; UV = Unscheduled Visit; V = Visit.

1 Visit 4 may take place on the same day as Visit 3, but this would result in a very long day for the subject (parents/guardians) as all Visit 4 procedures

would then have to take place that same day.

2 Unscheduled Visit in case of AEs, need for treatment re-supply, etc. Procedures will be performed as deemed necessary by the investigator.
3 Except for those that depend on the results of the immunological markers testing (peanut-specific IgE) and on the outcome of the entry/screening DBPCFC.
4 Including history of peanut allergy.
5 Including a complete skin examination, body weight and height.
6 These examinations are to be done before the DBPCFC. Additionally, they can be repeated during the DBPCFC procedure on both days anytime if judged necessary by the Investigator.
7 Blood pressure, heart rate and respiratory rate.
8 FEV_1 will be measured in subjects 6 years of age and older.
9 Peanut-specific IgE, peanut-specific IgG4, peanut specific IgE and peanut specific IgG4 to Ara h 1, Ara h 2, Ara h 3, Ara h 8 and Ara h 9. IgE specific to cow's milk, egg white, house dust mites, and grass pollen will be collected at Visit 1 and Visit 10 only and in case of an early termination visit.
10 Laboratory tests performed centrally. Hematology: hemoglobin, hematocrit, platelets, red blood cells, white blood cells with differential cell count. Biochemistry: aspartate aminotransferase, alanine aminotransferase, total bilirubin, total protein, blood urea nitrogen, creatinine.
11 For both FAQLQ and FAIM, subjects ≥8 years of age will use the Child Form of the FAQLQ/FAIM. All parents/guardians will use the Parental Form for children. The specific forms of FAQLQ and FAIM will be completed in the countries where they are available in local languages.
12 Signing the consent for the filaggrin genetic analysis can be done any time after the subject is effectively randomized in the study. However, collection of blood is recommended only once at any of the following visits: Visit 7, 8 or 10.
13 Check the reaction of the skin on the back of the subject and grade the severity of the local skin reactions. At Visit 4, grading is to be done before patch application and 30 min, 1 h, 2 h and 3 h after patch application.
14 The assessment of the patch adhesion to the skin will consist of a 28-day specific analysis to occur between Month 3 (Visit 7) and Month 6 (Visit 8). This assessment period can be extended up to Month 9 (Visit 9) in case it has not been completed or fully completed between Month 3 and Month 6.
15 The same treatment box dispensed at Visit 9 will be re-dispensed at Visit 10 (until Visit 11) after the compliance has been assessed.

7.2 Procedures by Visit

Visits should occur within the time windows indicated in the Schedule of Procedures Table 7. All times should be recorded using the 24-hour clock (for example 23:20, not 11:20 pm).

7.2.1 Visit 1, Screening (Day -42)

The first screening Visit (Visit 1) will take place within 42 days prior to the start of the treatment period. Therefore, the duration of the screening period could be less than 42 days.

The following assessments will be performed at this visit:

- Written informed consent;
- Check inclusion/exclusion criteria, except for those criteria that depend on the results of the peanut-specific IgE assessment and on the outcome of the entry/screening DBPCFC;
- Disease/Medical history (see Section 6.3.2);
- Parental (father and/or mother) medical history of atopy (any allergies, asthma conditions, eczema/atopic dermatitis);
- Demographics (see Section 0);
- Physical examination (including a complete skin examination; see Section 6.2.4);
- Vital signs (see Section 6.2.3);
- Spirometry (FEV_1) (see Section 6.2.5);
- PEF (see Section 6.2.6);
- SCORAD (see Section 6.4.7);
- SPT (see Section 6.4.2);
- Immunological markers (see Section 6.4.1);
- Laboratory tests (see Section 6.2.2); if applicable, subjects with abnormal laboratory assessments due to a concomitant transient disease (flu, viral illness, etc.) can repeat their laboratory assessments or be rescheduled for laboratory assessment at the discretion of the Investigator;
- Urine pregnancy test for female subjects of childbearing potential;
- FAQLQ/FAIM (see Section 6.4.3);
- Collect blood sample for epigenetic analyses (see Section 6.4.5);
- AEs recording (including volunteered or solicited AEs and AESI);
- Concomitant medications (see Section 5.8);
- Schedule Visit 2.

7.2.2 Visit 2, Screening (through Day -2)

Visit 2 corresponds to the first day of the entry/screening DBPCFC. This visit can take place any time through Day -2, as soon as the peanut-specific IgE results for the subject

are obtained by the site from the central laboratory. If the subject fulfills all inclusion criteria including the peanut-specific IgE value (>0.7 kU/L), then Visit 2 may proceed.

The following assessments will be performed at this visit:

- Physical examination (including a complete skin examination; to take place before DBPCFC; see Section 6.2.4);
- Vital signs (to take place before DBPCFC; see Section 6.2.3);
- PEF (to take place before DBPCFC; see Section 6.2.6);
- Entry/screening DBPCFC (first day) (see Section 6.1.1)
- AEs recording (including volunteered or solicited AEs, AESIs, and recording of AEs/allergies in response to the DBPCFC);
- Concomitant medications (including any medications given to treat allergic symptoms; see Section 5.8);
- Schedule Visit 3.

Physical examinations, assessment of vital signs and PEF measurements can be repeated during the DBPCFC as deemed necessary by the Investigator.

The subject may be discharged after a minimum observation period of 3 hours after the last dose of the challenge formula ingested. The observation period can be extended beyond 3 hours if deemed necessary by the Investigator.

7.2.3 Visit 3, Screening (within 1 week of Visit 2 and through Day -1)

Visit 3 corresponds to the second day of the entry/screening DBPCFC. It takes place within a week following Visit 2 (the first day of the entry/screening DBPCFC). Visit 3 could take place 1 day prior to Visit 4.

The following assessments will be performed at this visit:

- Physical examination (including a complete skin examination; to take place before DBPCFC; see Section 6.2.4);
- Vital signs (to take place before DBPCFC; see Section 6.2.3);
- PEF (to take place before DBPCFC; see Section 6.2.6);
- Entry/screening DBPCFC (second day) (see Section 6.1.1.3)
- AEs recording (including volunteered or solicited AEs, AESIs and recording of AEs/allergies in response to the DBPCFC);
- Concomitant medications (including any medications given to treat allergic symptoms; see Section 5.8);
- Schedule Visit 4.

Physical examinations, assessment of vital signs and PEF measurements can be repeated during the DBPCFC as deemed necessary by the Investigator.

The subject may be discharged after a minimum observation period of 3 hours after the last dose of the challenge formula ingested. The observation period can be extended beyond 3 hours if deemed necessary by the Investigator.

7.2.4 Visit 4 (Day 1), First Day of Treatment

All screening assessments must be completed and subject eligibility must be checked before entry into the double-blind treatment period; thereafter, dates of all study visits will be scheduled relative to the date of Visit 4. Visit 4 will take place at a maximum of 42 days after Visit 1 and might occur even closer to Visit 1 provided that Visit 2 and Visit 3 have been performed in between. Ideally, it will take place 1 day after Visit 3 and it may take place up to several weeks after Visit 3, respecting the maximum interval of 42 days after Visit 1. Visit 4 may take place the same day as Visit 3 for those Investigators willing to combine the 2 visits, under the condition that all procedures of both visits and their specific durations are respected.

The following assessments will be performed at this visit:

- Physical examination (including a complete skin examination; see Section 6.2.4);
- Vital signs (see Section 6.2.3);
- PEF (see Section 6.2.6);
- Confirm eligibility of subject and perform randomization;
- AEs recording (including volunteered or solicited AEs and AESI);
- Concomitant medications (see Section 5.8);
- Dispense first box of study drug (instruct subject/parents/guardians on the proper application, timing and storage);
- Apply the first Viaskin® patch to the subject and stick the tear-off part of the pouch label in the appropriate section of the subject diary.
- Keep under observation on-site for 3 hours after the patch has been applied;
- Check skin reaction under patch and grade severity (see Table 6, Section 6.2.8);
- Without removing the patch, take a photograph of the site of application of the Viaskin® patch after the 3 hours of application, load it onto the PEPITES website (described in section 6.2.8) and keep it in the subject's medical records or source documents;
- Patch adhesion Investigator's assessment: Investigator assesses the adherence of the patch to the skin as well as the occlusion of the patch chamber just after first application and after 3 hours prior to discharge;
- Dispense subject diary (instruct subject/parents/guardians on the use); see Section 6.2.7);
- Dispense the camera to the parents/guardians and instruct them when and how to use it;
- Dispense the subject safety leaflet and subject identification card;
- Dispense an auto-injector of epinephrine and explain in detail the anaphylaxis emergency action plan to the subject/parents/guardians before discharge;
- Dispense 1% hydrocortisone ointment;
- Schedule the Day 4 phone contact and Visit 5.

After the first application of the Viaskin® patch at the study site, all subjects will be observed for 3 hours before being discharged with the patch still applied to the skin and

the reactions under or around the patch will be checked and graded before discharge. The subject/parents/guardians will be instructed to remove the patch after a total of 6 hours of application, which is approximately 3 hours after discharge.

7.2.5 Phone Contacts (Day 4±2 days and Day 22±2 days), Treatment Period

At Day 4 after the patch has been applied 6 hours daily for 3 days, and at Day 22 (Week 3 of treatment), 1 week after the patch has been applied for 24 hours daily, specific phone contacts will be made to the parents/guardians to assess the safety of patch applications on the back. During the phone contacts, the site staff could ask to be provided with photos of the back of the subject to assess the local skin reactions. If required, the subjects may be seen earlier by the Investigator.

The following assessments will be performed during the phone contact:

- AEs recording (including volunteered or solicited AEs and AESI);
- Concomitant medications (see Section 5.8);
- Check for any accidental peanut consumption (see Section 6.4.4);
- Check with the subject (parents/guardians) to ensure that the diary has been completed accurately and that the subject/parents/guardians is/are comfortable with using the diary;
- Remind of Visit 5 and Visit 6, respectively.

7.2.6 Visit 5 (Day 8±3 days), Visit 6 (Month 1±3 days) and Visit 9 (Month 9±7 days), Treatment Period

The following assessments will be performed at these visits:

- Physical examination (including a complete skin examination; see Section 6.2.4);
- Vital signs (see Section 6.2.3);
- Spirometry (FEV_1), only for Visit 9 (see Section 6.2.5);
- PEF (see Section 6.2.6);
- AEs recording (including volunteered or solicited AEs and AESI);
- Concomitant medications (see Section 5.8);
- Check for any accidental peanut consumption (see Section 6.4.4);
- Check that the subject diary has been completed accurately and ensure that the subject/parents/guardians is/are comfortable with using the diary;
- Dispense subject diary (re-instruct subject/parents/guardians on the use, if necessary; see Section 6.2.7);
- Collect the treatment box dispensed at the previous visit, check the unused medication and assess medication compliance;
- Dispense a new box of study drug (re-instruct subject/parents/guardians on the proper application, timing and storage, if necessary);
- Patch adhesion Investigator's assessment : with the patch still applied, the Investigator assesses the adherence of the patch to the skin as well as the occlusion of the patch chamber;

- Check skin reactions under or around the current patch (do not remove patch) and all other zones of application and grade severity of the skin reactions (see Table 6, Section 6.2.8);
- Take a photograph of the sites of application of the Viaskin® patch, load it onto the PEPITES website (described in section 6.2.8) and keep it in the subject's medical records or source documents;
- Review the use of the auto-injector of epinephrine and the anaphylaxis emergency action plan;
- Schedule next phone contact and Visit 6, Visit 7, and Visit 10 respectively.

There is no requirement for subject observation before discharge at Visits 5, 6 and 9.

7.2.7 Phone Contacts (Month 2±3 days, Month 4.5±7 days, Month 7.5±7 days), Treatment Period

The parents/guardians of the subject will be called to assess the subject's condition since the last Visit.

The following assessments will be performed during the phone contact:

- AEs recording (including volunteered or solicited AEs and AESI);
- Concomitant medications (see Section 5.8);
- Check for any accidental or peanut consumption (see Section 6.4.4);
- Remind of Visit 7, Visit 8, and Visit 9 respectively.

7.2.8 Visit 7 (Month 3±7 days) and Visit 8 (Month 6±7 days), Treatment Period

The following assessments will be performed at these visits:

- Physical examination (including a complete skin examination; see Section 6.2.4);
- Vital signs (see Section 6.2.3);
- Spirometry (FEV_1) (see Section 6.2.5);
- PEF (see Section 6.2.6);
- SCORAD (see Section 6.4.7);
- SPT (see Section 6.4.2);
- Immunological markers (see Section 6.4.1);
- Laboratory tests (see Section 6.2.2);
- Written informed consent for filaggrin genetic screening (may be obtained at any visit after randomization, but must occur before blood sampling)
- Blood sampling for filaggrin genetic screening (after written informed consent has been obtained; only 1 sample is to be collected at Visit 7, 8 or 10; see Section 6.4.6);
- Collect blood samples for epigenetic analyses (see Section 6.4.5);
- AEs recording (including volunteered or solicited AEs and AESI);
- Concomitant medications (see Section 5.8);
- Check for any accidental peanut consumption (see Section 6.4.4);

- Check that the subject diary has been completed accurately and ensure that the subject/parents/guardians is/are comfortable with using the diary;
- Dispense subject diary (re-instruct subject/parents/guardians on the use, if necessary; see Section 6.2.7);
- Collect the treatment box dispensed at the previous visit, check the unused medication and assess medication compliance;
- Dispense a new box of study drug (re-instruct subject/parents/guardians on the proper application, timing and storage, if necessary);
- Patch adhesion Investigator's assessment: with the patch still applied, the Investigator assesses the adherence of the patch to the skin as well as the occlusion of the patch chamber;
- Check skin reactions under or around the current patch (do not remove patch) and all other zones of application and grade severity of the skin reactions (see Table 6, Section 6.2.8);
- Take a photograph of the sites of application of the Viaskin® patch, load it onto the PEPITES website (described in section 6.2.8) and keep it in the subject's medical records or source documents;
- Review the use of the auto-injector of epinephrine and the anaphylaxis emergency action plan;
- Schedule next phone contact and Visit 8 and Visit 9, respectively.
- Patch adhesion Subject's assessment: at Visit 7 (Month 3), the subject (parents/guardians) will be asked to assess the patch adhesion for 28 days between Visit 7 and Visit 8 (Month 6). The specific diary for assessing the patch adhesion should be dispensed and Investigators should instruct subject/parents/guardians on its use. The site staff will instruct the parents/guardians how to take the photographs and how to perform the assessment based on the grading system in Section 6.2.9.

  The patch adhesion diary and all pictures taken will be collected by the site staff at Visit 8 (Month 6) (and up until Visit 9, if necessary). The Investigator will check whether the subject (parents/guardians) has provided all photographs and evaluations and will review the parents/guardians' assessments based on the same photographs, to control and ensure that the parents' assessments of the patch adhesion were satisfactory.

There is no requirement for subject observation before discharge at Visits 7 and 8.

7.2.9 Visit 10 (Month 12±7 days), Treatment Period

Visit 10 corresponds to the first day of the post-treatment DBPCFC.

The following assessments will be performed at this visit:

- Physical examination (including a complete skin examination; to take place before DBPCFC; see Section 6.2.4);
- Vital signs (to take place before DBPCFC; see Section 6.2.3);
- Spirometry (FEV_1) (see Section 6.2.5);

- PEF (to take place before DBPCFC; see Section 6.2.6);
- SCORAD (see Section 6.4.7);
- SPT (see Section 6.4.2);
- Immunological markers (see Section 6.4.1);
- Laboratory tests (see Section 6.2.2);
- FAQLQ/FAIM (see Section 6.4.3);
- Written informed consent for filaggrin genetic screening, if not already done (this is the last visit with the possibility for signing this consent);
- Blood sampling for filaggrin genetic screening, if not already done at Visit 7 or 8; see Section 6.4.6);
- Urine pregnancy test for female subjects of childbearing potential;
- Collect blood sample for epigenetic analyses (see Section 6.4.5);
- Post-treatment DBPCFC (first day) (see Section 6.1.1.4)
- AEs recording (including volunteered or solicited AEs, AESIs, and recording of AEs/allergies in response to the DBPCFC);
- Concomitant medications (including any medications given to treat allergic symptoms) (see Section 5.8);
- Check for any accidental peanut consumption (see Section 6.4.4);
- Check that the subject diary has been completed accurately and ensure that the subject/parents/guardians is/are comfortable with using the diary;
- Dispense subject diary (re-instruct subject/parents/guardians on the use, if necessary; see Section 6.2.7);
- Collect the treatment box dispensed at the previous visit, check the unused medication and assess medication compliance;
- Re-dispense the same treatment box to the subject for application until the 2nd day of the challenge (re-instruct subject/parents/guardians on the proper application, timing and storage, if necessary);
- Patch adhesion Investigator's assessment: with the patch still applied, the Investigator assesses the adherence of the patch to the skin as well as the occlusion of the patch chamber;
- Check skin reactions of all zones of application and grade the overall severity of the skin reactions (see Table 6, Section 6.2.8);
- Take a photograph of the sites of application of the Viaskin® patch, load it onto the PEPITES website (described in section 6.2.8) and keep it in the subject's medical records or source documents;
- Schedule Visit 11.

Physical examinations, assessment of vital signs and PEF measurements can be repeated during the DBPCFC as deemed necessary by the Investigator.

The subject may be discharged after a minimum observation period of 3 hours after the last dose of the challenge formula is ingested. The observation period can be extended beyond 3 hours if deemed necessary by the Investigator.

7.2.10 Visit 11 (≤1 week after Visit 10), End of Treatment Period

Visit 11 corresponds to the end of the 12-month treatment period. Patch application ends at this visit. The patch worn by the subject when she/he arrives to this visit is removed and there will be no further patches applied beyond Visit 11.

Visit 11 corresponds to the second day of the post-treatment DBPCFC after 12 months of treatment. It takes place within a week following the first day of the post-treatment DBPCFC.

The following assessments will be performed at this visit:

- Physical examination (including a complete skin examination; to take place before DBPCFC; see Section 6.2.4);
- Vital signs (to take place before DBPCFC; see Section 6.2.3);
- PEF (to take place before DBPCFC; see Section 6.2.6);
- Post-treatment DBPCFC (second day) (see Section 6.1.1)
- AEs recording (including volunteered or solicited AEs, AESIs, and recording of AEs/allergies in response to the DBPCFC);
- Concomitant medications (including any medications given to treat allergic symptoms) (see Section 5.8);
- Check for any accidental peanut consumption (see Section 6.4.4);
- Check that the subject diary has been completed accurately and ensure that the subject/parents/guardians is/are comfortable with using the diary;
- Dispense subject diary (re-instruct subjects/parents/guardians on the use, if necessary; see Section 6.2.7);
- Collect the treatment box dispensed at the visit 10 (Month 12 visit), check the unused medication and assess medication compliance;
- Patch adhesion Investigator's assessment: with the patch still applied, the Investigator assesses the adherence of the patch to the skin as well as the occlusion of the patch chamber;
- Check skin reactions of all zones of application and grade the overall severity of the skin reactions (see Table 6, Section 6.2.8);
- Take a photograph of the sites of application of the Viaskin® patch, load it onto the PEPITES website (described in section 6.2.8) and keep it in the subject's medical records or source documents;
- Schedule Visit 12.

Physical examinations, assessment of vital signs and PEF measurements can be repeated during the DBPCFC as deemed necessary by the Investigator.

The subject may be discharged after a minimum observation period of 3 hours after the last dose of the challenge formula ingested. The observation period can be extended beyond 3 hours if deemed necessary by the Investigator.

7.2.11 Visit 12 (2 weeks after Visit 11), End of Study Period

The following assessments will be performed at this visit:

- Physical examination (including a complete skin examination; see Section 6.2.4);
- Vital signs (see Section 6.2.3);
- AEs recording (including volunteered or solicited AEs and AESI);
- Check for any accidental peanut consumption (see Section 6.4.4);
- Concomitant medications (see Section 5.8);
- Check subject diary.

7.2.12 Early Termination Visit

Subjects who discontinue early from the study should, if possible, have an Early Termination Visit. This visit should take place as soon as possible after the subject stops taking the study drug (see also Section 4.3).

The following assessments will be performed at these visits:

- Physical examination (including a complete skin examination; see Section 6.2.4);
- Vital signs (see Section 6.2.3);
- Spirometry (FEV_1) (see Section 6.2.5);
- PEF (see Section 6.2.6);
- SPT (see Section 6.4.2);
- Pregnancy Test
- Immunological markers (see Section 6.4.1);
- Laboratory tests (Section 6.2.2);
- AEs recording (including volunteered or solicited AEs and AESI);
- Concomitant medications (see Section 5.8);
- Check for any accidental peanut consumption (see Section 6.4.4);
- Check subject diary;
- Check unused medication and assess medication compliance;
- Check skin reactions and grade severity (see Table 6, Section 6.2.8);
- Take a photograph of the sites of application of the Viaskin® patch and keep it in the subject's medical records or source documents.

7.2.13 Unscheduled Visit

Subjects may come to the site for visits outside of the scheduled visits in case of AEs, Viaskin® patch adhesion problems, etc.

The following assessments will be performed at these visits, at the discretion of the Investigator:

- Physical examination (including a complete skin examination; see Section 6.2.4);
- Vital signs (see Section 6.2.3);
- Spirometry (FEV_1) (see Section 6.2.5);

- PEF (see Section 6.2.6);
- Laboratory tests (Section 6.2.2);
- AEs recording (including volunteered or solicited AEs and AESI);
- Concomitant medications (see Section 5.8);
- Check for any accidental peanut consumption (see Section 6.4.4);
- Check subject diary;
- Dispense a new box of study drug (re-instruct subject/parents/guardians on the proper application, timing and storage, if necessary);
- Check unused medication and assess medication compliance;
- Patch adhesion Investigator's assessment: with the patch still applied, the Investigator assesses the adherence of the patch to the skin as well as the occlusion of the patch chamber;
- Check skin reactions under or around the current patch (do not remove patch) and all other zones of application and grade severity of the skin reactions (see Table 6, Section 6.2.8);
- Take a photograph of the sites of application of the Viaskin® patch, load it onto the PEPITES website (described in section 6.2.8) and keep it in the subject's medical records or source documents.

8 STATISTICAL METHODS

The statistical considerations summarized in this section outline the plan for data analysis of this study.

A separate detailed statistical analysis plan (SAP) providing detailed methods for the analyses outlined below will be prepared before first subject inclusion and finalized before unblinding.

Any deviations from the planned analyses will be described and justified in the final integrated study report.

8.1 Study Patients

8.1.1 Disposition of Patients

Subject disposition will be summarized for the Intent-to-treat (ITT) population overall and by screening ED stratum. The number and percentage of subjects randomized, subjects in each study population (Safety, ITT, Full analysis set (FAS), Per-protocol [PP]), and subjects who completed the double-blind period, who discontinued the double-blind period, and the primary reason for discontinuation from the double-blind period will be tabulated by treatment group and overall.

An enrollment summary will be presented overall and by site, showing the first date of consent, and the last study visit exit date among enrolled subjects, duration (in days) which is calculated as last study visit exit date – first date of consent +1, number of subjects enrolled, randomized and completed. The number and percentage of subjects enrolled in total and by site will be summarized for each treatment group and overall.

8.1.2 Protocol Deviations

Deviations from the protocol including violations of inclusion/exclusion criteria will be assessed as “minor” or “major” in cooperation with the Sponsor prior to unblinding. Major deviations from the protocol that impact the primary efficacy evaluation will lead to the exclusion of a subject from the PP population.

8.1.3 Analysis Sets

All analysis populations will be identified and finalized in the SAP.

8.1.3.1 Safety Population

The Safety population will be comprised of all subjects who are randomized and have received at least 1 dose of study drug. This population will be used to assess comparative safety information. In case the wrong study drug is dispensed, the subject will be analyzed according to the study drug received for the longest period of time.

8.1.3.2 Intent-to-treat Population

The ITT population will be comprised of all subjects who are randomized. This population will be used to assess comparative efficacy information.

8.1.3.3 Full analysis set

The FAS will be comprised of all subjects who are randomized and have performed at least the peanut challenge of the second DBPCFC at Month 12.

8.1.3.4 Per-protocol Population

The PP population will include all subjects from the ITT population who do not have major deviations from the protocol that may affect the primary efficacy endpoint (for instance, subjects who have not gone through the second DBPCFCs at Month 12, subjects with a global treatment compliance below 80%, etc.). The deviations to consider will be listed more exhaustively in the SAP. The PP population will be used to perform confirmatory analyses of the primary and secondary efficacy evaluations.

8.2 General Considerations

8.2.1 Statistical Methods

The statistical analyses for the entire study as further outlined in the SAP will be included in the clinical study report (CSR) for this protocol. The SAP will give a detailed description of the summaries and analyses (primary and secondary) that will be performed and clearly describe when these analyses will take place. The SAP will be finalized shortly after the final protocol and well ahead of the database lock to preserve the integrity of the statistical analysis and study conclusions.

All pre-defined statistical analyses will be performed after the database is released for unblinding. Statistical analyses will be performed using SAS® Version 9.3 or higher (SAS Institute, Cary, NC, USA).

Categorical variables will be summarized using number of observations and percentages. The denominator for percentages will be the number of subjects in the population with data available unless otherwise stated. Continuous variables will be summarized using descriptive statistics (number of observations [n], mean, standard deviation, minimum, first quartile [Q1], median, third quartile [Q3], and maximum).

8.2.2 Analysis and Data Conventions

8.2.2.1 Definition of Baseline

The baseline assessment will be the latest, valid pre-dose assessment available.

8.2.2.2 Visit Windows

Assessments outside of protocol allowable windows will be taken into account in the analysis according to the visit in which the data are entered.

8.2.2.3 Unscheduled Assessments

Extra assessments (laboratory data or vital signs associated with non-protocol clinical visits or obtained in the course of investigating or managing AEs) will be included in listings, but not summaries. If more than 1 laboratory value is available for a given visit, the first valid observation will be used in summaries and all observations will be presented in listings. It is noted that invalid laboratory data may not be used (from

hemolyzed samples, mishandled samples, quantity not sufficient, or other conditions that would render values invalid).

8.2.2.4 Missing Data Conventions

Every attempt must be made by the Investigator to provide complete data.

Analyses of primary and secondary efficacy endpoints:

Analyses of primary and secondary efficacy endpoints will be based on the ITT population, with missing values imputed using last observation carried forward (LOCF) imputation (whereby the last value is considered as the value of threshold sensitivity at entry/screening DBPCFC). Because threshold sensitivity to peanut protein for all randomized subjects will be ≤300 mg (based on the exclusion criteria), subjects who discontinue prior to the post-treatment DBPCFC will be counted as non-responders in the efficacy analyses.

In addition, primary and secondary efficacy analyses will be repeated on the FAS.

Besides, the robustness of the primary and secondary efficacy analyses conclusions will be explored via sensitivity analyses on the PP population (thus excluding subjects who discontinue from the study prior to the post-treatment DBPCFC or those with a major protocol deviation that impacts the primary efficacy criteria).

Lastly, sensitivity analyses of the primary efficacy endpoint will be conducted on the overall ITT population using multiple imputation and worst-case imputation (which means subjects from the Viaskin® Peanut group who discontinue prior to the post-treatment DBPCFC will be counted as non-responders and subjects from the placebo group who discontinue prior to the post-treatment DBPCFC will be counted as responders) instead of the LOCF imputation to assess the robustness of the primary efficacy analyses with regards to handling of missing data.

Analyses of other efficacy, safety and exploratory criteria:

No imputation will be performed for other efficacy, safety and exploratory criteria (observed data will be used). Partial or missing safety data will be imputed according to the most conservative approach. Adverse events with missing or incomplete onset date will be considered as TEAEs unless there is evidence that the event occurred prior to the treatment period. Treatment-emergent AEs with missing relationship to the study drug will be considered as drug-related and TEAEs with missing severity will be considered as severe. Actual values will be presented in listings.

8.2.2.5 Pooling of Centers

Considering the expected small number of subjects to be randomized by center, the center effect will not be investigated. The region (Australia/Europe/North America) and the treatment-by-region factor might be integrated as factors in the model used for the analysis of the primary efficacy endpoint as well as treatment response analyses by ED stratum in sensitivity analyses.

8.3 Demographics, Disease and Medical Histories, Baseline Characteristics, and Concomitant Medications

Descriptive statistics will be produced for continuous demographic and baseline characteristics (including age, height, weight, BMI, FEV_1, PEF, and peanut-specific IgE) for each treatment group, by screening ED stratum and overall. The number and percent of subjects in each group of the categorical demographic and baseline characteristics (including race, medical history, and SPT) will be tabulated by treatment group and screening ED stratum, and overall.

Concomitant medications will be coded using the latest available version of the World Health Organization (WHO) Drug Dictionary. A summary of concomitant medications will be produced by preferred drug name and treatment group. All concomitant medications will be listed.

Medical history will be reported by SOC and PT and coded using the latest available version of the Medical Dictionary for Regulatory Activities (MedDRA) dictionary. Disease history will also be described.

All individual subject demographic and baseline characteristic data will be listed.

8.4 Treatment Compliance and Exposure

Treatment compliance (see Section 5.6) will be summarized for each treatment group by means of descriptive statistics (n, mean, SD, median, Q1, minimum, Q3, and maximum) and/or frequency tables (compliance < or ≥80%).

The total number of days of patch application during the whole study will be presented overall and by treatment group. Besides, the exposure duration by subject will be summarized, quantitatively and by category, overall and by treatment group. Exposure duration will be calculated regardless of temporary interruptions, as follows:

Date of last patch application – Date of first patch application + 1

The total dose of peanut protein received during the study (mg) will also be summarized overall and by treatment group.

8.5 Efficacy Analyses

8.5.1 Primary Efficacy Analysis

The primary efficacy endpoint in this study is the percentage of treatment responders in the active Viaskin® Peanut 250 µg group compared to the placebo group after 12 months of EPIT treatment.

8.5.1.1 Hypothesis to be tested

The null hypothesis is that the percentage of treatment responders in the active Viaskin® Peanut 250 µg group is the same as that in the placebo treatment group (which means the parameter estimate associated to the treatment group is equal to zero).

8.5.1.2 Statistical Methods

The primary efficacy analysis will be performed on the ITT population (that is on all randomized subjects), using LOCF imputation (which means that subjects with missing DBPCFC value at Month 12 will be considered as non-responders). An exact logistic regression will be used to compare the percentage of treatment responders at Month 12 in the active Viaskin® Peanut 250 µg group versus the placebo group, adjusting for screening ED stratum and including the treatment group as fixed effect. A 2-sided, 1% significance level will be used to test the null hypothesis. The corresponding p-value as well as the number and percentage (95% confidence intervals [CIs]) of responders will be presented by treatment group. The difference between active and placebo response rates and the corresponding 95% CI will be presented. The clinical relevance of the treatment effect will be evaluated based on the lower bound of the 95% CI being higher or equal to 15%. This 15% difference is intended to exhibit a clear robustness of the treatment effect.

Relative risks of achieving response in the active group compared to the placebo group will be assessed using a log-binomial regression with the screening ED stratum and treatment group as covariates and will be presented together with associated 95% CI.

The study will be considered positive, if the p-value from the exact logistic regression is significant ($p<0.01$) and the lower bound of the 95% CI of the difference between active treatment and placebo response rates is higher or equal to 15%.

8.5.1.3 Sensitivity Analyses

The above analysis will be repeated on the FAS and PP population. Sensitivity analyses will be conducted on the ITT population using multiple imputation and worst-case imputation instead of the LOCF imputation to assess the robustness of the primary efficacy analysis with regard to handling of missing data. In addition, a sensitivity analysis with the region (Australia/Europe/North America) and the interaction between region and treatment group as covariate will be performed.

8.5.2 Secondary Efficacy Analyses

8.5.2.1 Percentage of treatment responders in each screening ED stratum

The first secondary efficacy analysis is the comparison of the percentage of treatment responders at Month 12 in each screening ED stratum. Within each screening ED stratum, the treatment group comparison is conducted on the ITT population at a 2-sided 5% significance level, using LOCF imputation and the exact logistic model described in Section 8.5.1.2. The corresponding p-value as well as the number and percentage of responders (95% CI) will be presented by treatment group. The difference between active and placebo response rates and the corresponding 95% CI will be presented. The clinical relevance of the treatment effect in each screening ED stratum will be evaluated based on a lower bound of the 95% CI strictly higher than 0%. The relative risk of achieving a response in the active group compared to the placebo group within each screening ED stratum will be assessed using the log-binomial model described in Section 8.5.1.2 and will be presented together with the associated 95% CI.

The above analysis will be repeated on the FAS and PP population. Besides, a sensitivity analysis with the region (Australia/Europe/North America) and the interaction between region and treatment group as covariate will be performed.

8.5.2.2 Percentage of treatment responders in each age subgroup

The comparison of the percentage of treatment responders at Month 12 in the following age subgroups will be presented: 4 to 5 years-old; 6 to 11 years-old. Within each age subgroup, the treatment group comparison is conducted on the ITT population at a 2-sided 5% and 10% significance levels, using LOCF imputation and the exact logistic model described in Section 8.5.1.2. The corresponding p-value as well as the number and percentage of responders (95% CI) will be presented by treatment group. The difference between active and placebo response rates and the corresponding 95% CI will be presented. The relative risk of achieving a response in the active group compared to the placebo group within each subgroup will be assessed using the log-binomial model described in Section 8.5.1.2 and will be presented together with the associated 95% CI.

The above analysis will be repeated on the FAS and PP population.

8.5.2.3 Mean and Median Cumulative Reactive/Eliciting Doses

The peanut protein cumulative reactive dose at Month 12 and the peanut protein ED at Month 12 will be summarized descriptively by treatment group (for the whole population and for each screening ED stratum), for the ITT population using LOCF imputation, as well as for the FAS and PP population.

In addition, the peanut protein cumulative reactive dose and the ED in each treatment group at Month 12 will be compared using an analysis of covariance (ANCOVA) model. The ANCOVA model will include the treatment group, baseline value, screening ED stratum and region (Australia/Europe/North America) as covariates. Results will be log-transformed if needed. The peanut protein cumulative reactive dose and the ED will be evaluated for the ITT population using LOCF imputation, as well as for the FAS and PP population, overall and for each screening ED stratum, at a 2-sided 5% significance level.

8.5.2.4 Responsive/Unresponsive Subjects, Subjects Passing DBPCFC

The percentage of subjects responsive to a cumulative dose ≥1,444 mg peanut protein at Month 12, the percentage of subjects unresponsive to a cumulative dose ≥1,444 mg peanut protein at Month 12, and the percentage of subjects passing the DBPCFC at Month 12 will be summarized descriptively by treatment group, for the whole population and for each screening ED stratum, for the ITT population using LOCF imputation, as well as for the FAS and PP population.

In addition, the percentage of subjects responsive to a cumulative dose ≥1,444 mg peanut protein at Month 12, the percentage of subjects unresponsive to a cumulative dose ≥1,444 mg peanut protein at Month 12 and the percentage of subjects passing the challenge in each treatment group at Month 12 will be compared using an exact logistic regression (adjusting for screening ED stratum and including the treatment group as fixed effect) for the overall ITT population using LOCF imputation, as well as for the FAS and PP population, at a 2-sided 5% significance level.

8.5.3 Pre-defined Hierarchical Order for the Analysis of Efficacy Endpoints

In order to handle the multiple comparisons versus placebo, the overall type-I error will be controlled by the use of a hierarchical inferential approach. Statistical significance of the primary efficacy criterion in the overall population at the 1% alpha level is required before drawing inferential conclusion about the first secondary efficacy criterion (percentage of treatment responders in Stratum 1). Statistical significance of the first secondary efficacy endpoint at the 5% alpha level is required before drawing inferential conclusion about the next endpoint (percentage of treatment responders in Stratum 2). Inferential conclusion about successive secondary efficacy endpoints require statistical significance at the 5% alpha level of the previous one. Specifically, the pre-defined hierarchical order is as summarized in Table 8.

Table 8: Pre-defined Hierarchical Order for Analysis of Efficacy Endpoints

Order	Efficacy endpoints (at Month 12)	Population	Missing data imputation method	Alpha	Method
1	Percentage of treatment responders	ITT Overall	LOCF imputation	0.01	Exact logistic regression
2	Percentage of treatment responders	ITT Screening ED Stratum 1	LOCF imputation	0.05	Exact logistic regression
3	Percentage of treatment responders	ITT Screening ED Stratum 2	LOCF imputation	0.05	Exact logistic regression
4	Cumulative reactive dose	ITT Overall	LOCF imputation	0.05	ANCOVA
5	Peanut protein ED	ITT Overall	LOCF imputation	0.05	ANCOVA
6	Percentage of treatment responders	ITT Age subgroup 6 to 11 years-old	LOCF imputation	0.10	Exact logistic regression
7	Percentage of treatment responders	ITT Age subgroup 4 to 5 years-old	LOCF imputation	0.10	Exact logistic regression
8	Percentage of subjects responsive to a cumulative dose ≥1,444 mg peanut protein	ITT Overall	LOCF imputation	0.05	Exact logistic regression
9	Percentage of subjects unresponsive to a cumulative dose ≥1,444 mg peanut protein	ITT Overall	LOCF imputation	0.05	Exact logistic regression
10	Cumulative reactive dose	ITT Screening ED Stratum 1	LOCF imputation	0.05	ANCOVA
11	Peanut protein ED	ITT Screening ED Stratum 1	LOCF imputation	0.05	ANCOVA
12	Cumulative reactive dose	ITT Screening ED Stratum 2	LOCF imputation	0.05	ANCOVA
13	Peanut protein ED	ITT Screening ED Stratum 2	LOCF imputation	0.05	ANCOVA
14	Percentage of subjects passing the challenge	ITT Overall	LOCF imputation	0.05	Exact logistic regression

Abbreviations: ANCOVA = Analysis of covariance; ED = Eliciting dose; ITT = Intent-to-treat; LOCF = Last observation carried forward.

8.5.4 Other Efficacy Analyses

No further adjustments will be made for the other efficacy endpoints for which p-values will be provided for descriptive purpose only. The following efficacy analyses will be performed using observed data (no imputation will be done).

- Peanut-specific IgE and IgG4 analysis over time: Peanut-specific IgE and IgG4 levels at baseline and changes from baseline to Months 3, 6 and 12 will be summarized descriptively by treatment group on the whole ITT population and for each screening ED stratum, using observed data. Besides, the peanut-specific IgE and IgG4 results at Month 12 will be compared for the whole ITT population and for each screening ED stratum using an ANCOVA model on observed data. The ANCOVA model will include the treatment group, baseline value, screening ED stratum, and region (Australia/Europe/North America) as covariates. Results will be log-transformed if needed.
- Skin Prick Test analysis: The mean change from baseline at Months 3, 6 and 12 in maximum average wheal diameter will be evaluated for each treatment group on the whole ITT population using observed data. The SPT results at Month 12 will be compared for the whole ITT population using an ANCOVA model on observed data. The ANCOVA model will include the treatment group, baseline value, screening ED stratum, and region (Australia/Europe/North America) as covariates. Results will be log-transformed if needed.

A quality of life score from the FAQLQ/FAIM will be summarized at Month 12 and compared to baseline for both treatment groups for the overall ITT population, using observed data.

8.6 Safety Analyses

Safety endpoints will be evaluated for the Safety population (all subjects who received at least 1 dose of study drug).

For all safety analyses, data will be summarized for Viaskin® Peanut 250 µg versus placebo during the 12 months of treatment.

8.6.1 Adverse Events

Treatment-emergent AEs will be defined as any AEs, regardless of relationship to study drug, which occur during or after the initial Viaskin® patch application or any event already present that worsens in either severity or relationship to study drug following exposure to Viaskin® patches.

All AEs will be reported by SOC and PT as detailed in Section 9.4 and coded using the latest available version of the MedDRA dictionary.

An overall summary of TEAEs will be provided showing the number and percentage of subjects in each treatment group with any TEAEs, any potentially drug-related TEAEs, any severe TEAEs, any severe potentially drug-related TEAEs, any serious TEAEs, any TEAEs leading to discontinuation, and any TEAEs leading to death. The number of events will also be presented.

The number of AEs as well as the number and percentage of subjects who experienced at least 1 AE will be summarized by SOC, PT and treatment group. The incidence of the following events will be summarized:

- TEAEs (distinguished from symptoms/reactions elicited during the DBPCFCs): incidence, maximum severity and duration;
- Local skin tolerance at sites of Viaskin® patch application as assessed by the subjects (incidence, duration and severity) (see Section 8.6.6);
- Local skin tolerance at sites of Viaskin® patch application as assessed by the Investigator (severity) (see Section 8.6.7);
- SAEs and serious TEAEs and relatedness;
- Potentially drug-related TEAEs;
- Discontinuation due to TEAEs;
- AESI (including systemic allergic reactions considered related to Viaskin® Peanut and vesicles at patch application sites).

The safety parameters above will be analyzed in the overall Safety population, as well as for each of the age ranges 4 to 5 years, 6 to 8 years and 9 to 11 years, and for each of the screening ED strata.

All AEs will be listed.

The reactions appearing during a DBPCFC (as they are expressly provoked) will be differentiated from those AEs occurring outside of the DBPCFC. Objective and subjective symptoms/reactions elicited during the DBPCFCs in the different treatment groups (see Section 6.1.1 for full details) will be summarized separately (see Section 8.6.8).

8.6.2 Laboratory Assessments

Descriptive statistics will be calculated for clinical laboratory tests (hematology and biochemistry) performed at Visit 1 (Day -42), Visit 7 (Month 3), Visit 8 (Month 6) and Visit 10 (Month 12). Categorical variables will be summarized by frequency and percentages of subjects in corresponding categories.

Changes in laboratory data from baseline will also be presented.

In addition, summaries of laboratory values categorized based on Common Toxicity Criteria for Adverse Events (CTCAE) grade will also be presented.

Shift tables of test abnormalities will be generated to compare baseline values to the values collected at other time points.

Laboratory data will be analyzed overall and for the age ranges 4 to 5 years, 6 to 8 years and 9 to 11 years, as well as for each screening ED stratum and association with any age stratum will be presented.

All laboratory data will be listed. Listing of values that are out of normal range will be flagged in the data listings.

8.6.3 Vital Signs

Observed vital sign values and changes from baseline will be descriptively summarized by visit and treatment group. All vital signs data will be listed.

The analysis of vital signs will focus on the incidence of clinically relevant abnormalities. The number of subjects evaluated and the number and percentage of subjects with clinically relevant post-baseline abnormalities at each visit will be presented. The criteria for clinically relevant post-baseline abnormalities are shown in Table 9.

Table 9: Criteria to assess Clinically Relevant Abnormalities in Vital Signs

Vital Sign Criteria for Abnormalities	Criteria for Abnormalities
Pulse	>130 beats per minute or an increase from pre-dosing of >20 beats per minute, or<br><60 beats per minute or a decrease from pre-dosing of >20 beats per minute
Systolic blood pressure	>130 mmHg or an increase from pre-dosing of >40 mmHg, or<br><70 mmHg or a decrease from pre-dosing of >30 mmHg
Diastolic blood pressure	>85 mmHg or an increase from pre-dosing of >30 mmHg, or<br><45 mmHg or a decrease from pre-dosing of >20 mmHg

Vital signs will be analyzed overall and for the age ranges 4 to 5 years, 6 to 8 years and 9 to 11 years, as well as for each screening ED stratum and association with any age stratum will be presented.

8.6.4 Physical Examination

Physical examination data will be summarized by visit and treatment group and listed.

Changes in physical examination data from baseline will be presented. Physical examination data will be analyzed overall and for the age ranges 4 to 5 years, 6 to 8 years and 9 to 11 years, as well as for each screening ED stratum and association with any age stratum will be presented.

Skin reactions observed during the physical examinations will also be reported and the corresponding data will be tabulated separately (see Section 8.6.7).

8.6.5 Spirometry and Peak Expiratory Flow Results

Percent predicted values for FEV_1 and PEF and changes from baseline will be descriptively summarized separately by visit and treatment group. All FEV_1 and PEF data will be listed. Spirometry and PEF data will be analyzed overall and for the age ranges 4 to 5 years, 6 to 8 years and 9 to 11 years, as well as for each screening ED stratum and association with any age stratum will be presented.

8.6.6 Subject Diaries

Subject diary data will be summarized where appropriate and listed.

During the first 6 months, the number of days of itching, redness and swelling of Grade 1, 2, or 3 will be summarized separately by treatment group, age ranges (4 to 5 years, 6 to 8 years and 9 to 11 years), screening ED stratum, and overall.

The most severe grades of itching, redness and swelling, documented in the diary, will also be summarized. The maximum grade of local reactions reported (itching, redness or swelling) during the first 6 months will also be tabulated.

8.6.7 Skin Reactions

Viaskin® patch site examination data will be summarized where appropriate and listed.

Examination of the skin at the site of patch application will be graded by the Investigators on a scale of Grade 0 (negative) to Grade 4 (erythema, vesicles). Localization of the skin reaction (under the patch/beyond the patch) will also be collected. These results will be summarized using descriptive statistics and presented by visit and treatment group, regardless of the localization as well as for each type of localization. The worst grade reported will also be tabulated. The above descriptions will be presented for the overall Safety population as well as for the age ranges 4 to 5 years, 6 to 8 years and 9 to 11 years and for each screening ED stratum.

8.6.8 Symptomatic Reactions During the DBPCFC

The incidence of objective and subjective symptoms/reactions elicited during the DBPCFCs will be summarized. Besides, a severity score based on the grades of objective symptoms elicited during the DBPCFCs will be described at baseline and at Month 12. The change in the severity score from baseline will also be presented.

8.7 Exploratory Analyses

There will be no adjustments for multiplicity for any of the following exploratory analyses.

The following exploratory analyses will be done:

- Analysis of IgE and IgG4 specific to peanut protein components: Descriptive analysis will be conducted for IgE and IgG4 specific to peanut protein components for the overall ITT population, using observed data. Comparison between treatment groups will be conducted using the ANCOVA model described above;
- Frequency of accidental consumptions, conditions around the accidental consumptions, estimated quantity consumed at each occurrence, and associated reactions and severity of reactions. These AEs will be classified and analyzed separately and specifically;
- Risk-taking behaviors: frequency of deliberate consumption of peanut, conditions around the consumption, estimated quantity consumed at each occurrence and associated reactions with these consumptions. These AEs will be classified and analyzed separately and specifically for the overall ITT population, using observed data;
- Descriptive analyses of epigenetic modifications of some specific genes in both treatment groups for the overall ITT population, using observed data;
- Treatment-emergent AEs and local skin tolerance at sites of Viaskin® patch application will be described among subjects with mutations in the filaggrin gene versus subjects carrying the wild type gene;
- Descriptive analysis will be conducted for each of the other allergen-specific IgE analyzed (cow's milk, egg, house dust mites, grass pollen) and the change from baseline will be tabulated for the overall ITT population, using observed data;

- SCORAD change from baseline at Months 3, 6 and 12 will be presented for the overall ITT population using observed data.

8.8 Patch adhesion

A frequency table showing the number of patches evaluated by the subjects' parents/guardians in their diaries and the proportion of patches for each adhesion score (see Section 6.2.9) will be presented, immediately after patch application and at time of patch removal.

Percentage of patches assessed by the subjects' parents/guardians and having an adhesion score equal to 2 (which means the occlusion chamber of the patch is partially or totally detached) or 3 (which means the patch has fallen off the skin) will be tabulated and the time to removal for those patches will be described.

Patch adhesion will be considered as acceptable if more than 90% of the patches at time of target patch removal, i.e. at 24±4 hours, have an adhesion score ≤1 as assessed by the subjects' parents/guardians.

Descriptive analysis will be conducted on the suspected causes of adhesion issues (scratching / swimming / sweating / other) and on the potential presence of skin injury(ies) caused by patch removal (yes / no), as assessed by the subjects (parents/guardians). Proportion of patches for each grade of patch removal easiness (very easy / easy / difficult / very difficult) and for each grade of pain at patch removal (very painful / painful / not painful), as assessed by the subjects (parents/guardians), will be tabulated.

Finally, the proportion of patches for each adhesion score as assessed by the Investigator at each time point will be tabulated.

8.9 Interim Analyses

No interim analysis is planned for this study.

8.10 Determination of Sample Size

The objective of this study is to assess the efficacy and safety of Viaskin® Peanut to induce desensitization to peanut in peanut-allergic children after a 12-month treatment period of EPIT. This will be evaluated by assessing the percentage of treatment responders in the active Viaskin® Peanut 250 µg group compared to the placebo group.

The sample size is calculated based on the following assumptions:

- A 40% response rate for the active Viaskin® Peanut 250 µg group and 10% response rate for the placebo group in the overall population, with a 2-sided type-I error rate α set to 1%;
- A lower bound of the 95% CI of the difference between active treatment and placebo response rates ≥15% in the overall population and >0% in each stratum;
- A 60% response rate for the active Viaskin® Peanut 250 µg group and 10% response rate for the placebo group in Stratum 1, with a 2-sided type-I error rate α set to 5%;

- A 30% response rate for the active Viaskin® Peanut 250 µg group and 10% response rate for the placebo group in Stratum 2, with a 2-sided type-I error rate α set to 5% and a power (1-β) set to 85%;
- The following expected distribution of the screening ED strata:
  - 1/3 of children having a screening ED from 1 mg to 10 mg;
  - 2/3 of children having a screening ED from 30 mg to 300 mg.
- A ratio 2:1 to maximize the number of subjects treated in the active Viaskin® Peanut 250 µg group.

Therefore, the sample size calculated for Stratum 2 is 185 subjects (approximately 123 subjects in the active Viaskin® Peanut 250 µg group and 62 subjects in the placebo group. Hence, if 185 children with a screening ED from 30 mg to 300 mg are required in the study, then, according to the expected distribution of the screening ED strata, approximately 95 children having a screening ED from 1 mg to 10 mg should be randomized, resulting in a total of 280 subjects evaluable for the primary efficacy endpoint in the analysis.

This number of subjects leads to a power of 99% in the overall population and in the Stratum 1.

This is summarized in Table 10.

Table 10: Summary of Sample Size Calculation

Population	alpha	Power	Sample size (N=N1+N2) Expected Response Rate			Lower bound of the 95% CI of the difference
			Viaskin® Peanut 250 µg	Placebo	Total	
Screening ED Stratum 1	5%	99%	N1=63 60%	N2=32 10%	N=95	>0%
Screening ED Stratum 2	5%	85%	N1=123 30%	N2=62 10%	N=185	>0%
Overall population	1%	99%	N1=186 40%	N2=94 10%	N=280	≥15%

Abbreviations: CI = Confidence interval; ED = Eliciting dose; N = Number of subjects.

To allow for a potential premature withdrawal rate of about 15%, the study will randomize a total of 330 subjects: 220 subjects will be randomized to receive Viaskin® Peanut 250 µg and 110 subjects to placebo. Among the 330 subjects randomized, a minimum of 20 subjects will be randomized for each of the following ages: 4 years, 5 years and 11 years, respectively. Besides, a minimum number of 75 children of either 4 or 5 years of age must be randomized.

The distribution per ED stratum should lead to approximately 110 subjects with a screening ED from 1 mg to 10 mg and approximately 220 subjects with a screening ED from 30 mg to 300 mg, provided that the same distribution as obtained in the Phase IIb VIPES study is reproduced. If this expected distribution is not fully respected during the recruitment in the PEPITES study, a minimum of 50 subjects would need to be included in Stratum 1 in order to guarantee a power of ≥90% in this stratum and at least

220 subjects would need to be included in Stratum 2 in order to guarantee a power of ≥85% in this stratum.

From experience, the screen failure rate could be up to 30%; hence, up to 470 peanut allergic subjects might need to be screened.

9 ETHICAL, LEGAL, AND ADMINISTRATIVE ASPECTS

9.1 Data Quality Assurance

The Sponsor or Sponsor's designee will conduct a site visit or a site phone contact to verify the qualifications of each Investigator, inspect the site facilities, and inform the Investigator of responsibilities and the procedures for ensuring adequate and correct documentation.

The Investigator is required to prepare and maintain adequate and accurate case histories designed to record all observations and other data pertinent to the study for each study participant. All information recorded in the e-CRF for this study must be consistent with the subjects' source documentation (that is medical records).

9.1.1 Database Management and Quality Control

All data generated by the site personnel will be captured electronically at each study center using e-CRFs. Data from external sources (such as laboratory data) will be imported into the database. Once the e-CRF clinical data have been submitted to the central server at the independent data center, corrections to the data fields will be captured in an audit trail. The reason for change, the name of the person who performed the change, together with the time and date will be logged to provide an audit trail.

If additional corrections are needed, the responsible monitor or data manager or the Sponsor will raise a query in the Electronic Data Capture (EDC) application. The appropriate staff at the study site will answer queries sent to the Investigator. The name of the staff member responding to the query, and time and date stamp will be captured to provide an audit trail. Once all source data verification is complete and all queries are closed, the monitor will freeze the e-CRF page.

The specific procedures to be used for data entry and query resolution using the EDC system/e-CRF will be provided to study sites in a training manual. In addition, site personnel will receive training on the EDC system/e-CRF.

9.2 Case Report Forms and Source Documentation

All data obtained during this study should be entered in the e-CRFs promptly. All source documents from which e-CRF entries are derived should be placed in the subject's medical records. Measurements for which source documents are usually available include laboratory assessments, spirometry, PEF and immunological markers.

Data that will be entered directly into the e-CRF (those for which there is no prior written or electronic record of data) are considered to be source data.

The original e-CRF entries for each subject may be checked against source documents at the study site by PAREXEL's site monitor.

After review by PAREXEL's site monitor, completed e-CRF entries will be uploaded and forwarded to PAREXEL. Instances of missing or uninterpretable data will be discussed with the Investigator for resolution.

The specific procedures to be used for data entry and query resolution using the e-CRF will be provided to study sites in a training manual. In addition, site personnel will receive training on the e-CRF.

9.2.1 Data Collection

The Investigators (and appropriately authorized staff) will be given access to an online web–based EDC system which is 21 CFR Part 11 compliant. This system is specifically designed for the collection of the clinical data in electronic format. Access and rights to the EDC system will be carefully controlled and configured according to each individual's role throughout the study. In general, only the Investigator and authorized staff will be able to enter data and make corrections in the e-CRFs.

The e-CRF should be completed for each subject for whom the study-specific ICF was obtained and should reflect the latest observations on the subject participating in the study. Therefore, the e-CRFs are to be completed as soon as possible during or immediately after the subject's visit or assessment. The Investigator must verify that all data entries in the e-CRF are accurate and correct. If some assessments cannot be done, or if certain information is unavailable, not applicable or unknown, the Investigator should indicate this in the e-CRF.

Computerized data-check programs and manual checks will identify any clinical data discrepancies for resolution. Corresponding queries will be loaded into the system and the site will be informed about new issues to be resolved on-line. All discrepancies will be solved on–line directly by the Investigator or by authorized staff. Off-line edit checks will be done to examine relationships over time and across panels to facilitate quality data.

After completion, the Investigator will be required to electronically sign off the clinical data.

Information concerning study drug dispensation to the subject will be tracked in the e-CRF.

9.3 Access to Source Data

9.3.1 Routine Monitoring

During the study, a PAREXEL site monitor will make site visits to review protocol compliance, compare e-CRF entries and individual subject's medical records, assess drug accountability, and ensure that the study is being conducted according to ethical and pertinent regulatory requirements. The e-CRF entries will be verified with source documentation. The review of medical records will be performed in a manner to ensure that subject confidentiality is maintained.

Checking of the e-CRF entries for completeness and clarity, and cross-checking with source documents, will be required to monitor the progress of the study. Direct access to source data will be required for the monitoring activities.

9.3.2 Inspections and Auditing Procedures

The Sponsor or its representative may conduct audits at the investigative sites including, but not limited to, drug supply, presence of required documents, the informed consent process, and comparison of e-CRFs with source documents. All medical records (progress notes) must be available for audit. The Investigator agrees to participate in audits conducted at a convenient time in a reasonable manner.

Moreover, Regulatory Authorities of certain countries, IRBs and IECs may wish to carry out such source data checks and/or on-site audit inspections. Direct access to source data will be required for these inspections and audits; they will be carried out giving due consideration to data protection and medical confidentiality. The Investigator assures PAREXEL and the Sponsor of the necessary support at all times.

The purpose of an audit is to assess whether ethics, regulatory and quality requirements are fulfilled.

9.4 Data Processing

All data will be entered by site personnel into the e-CRF (as detailed in Section 9.2.1).

The data-review and data-handling document, to be developed during the initiation phase of the study, will include specifications for consistency and plausibility checks on data and will also include data-handling rules for obvious data errors. Query/correction sheets for unresolved queries will be sent to the study monitors for resolution with the Investigator. The database will be updated on the basis of signed corrections.

Previous and concomitant medications will be coded using the WHO Drug Reference List, which employs the Anatomical Therapeutical Chemical (ATC) classification system. Disease and Medical histories/current medical conditions and AEs will be coded using the MedDRA terminology.

Previous and concomitant diseases as well as AEs will be coded with MedDRA.

The versions of the coding dictionaries will be provided in the CSR.

9.5 Archiving Study Records

All data derived from the study will remain the property of the Sponsor. Records must be retained in accordance with the current ICH Guidelines on Good Clinical Practice (GCP). All essential study documents including records of subjects, source documents, e-CRFs and study drug inventory must be kept in a study-specific file.

US Food and Drug Administration (FDA) regulations (21 Code of Federal Regulations [CFR] 312.62[c]) require that records and documents pertaining to the conduct of this study and the distribution of investigational drug, including e-CRFs, consent forms, laboratory test results, and medical inventory records, must be retained by the Principal Investigator for 2 years after marketing application approval. If no application is filed, these records must be kept 2 years after the investigation is discontinued and the US FDA and the applicable national and local health authorities are notified. The Sponsor or their representative will notify the Principal Investigator of these events.

The Investigator will not dispose of any records relevant to this study without written permission from the Sponsor, and will provide the Sponsor the opportunity to collect such records. The Investigator shall notify the Sponsor in writing of their intent to destroy all such material. The Sponsor shall have 30 days to respond to the Investigator’s notice, and the Sponsor shall have a further opportunity to retain such materials at the Sponsor’s expense.

The Investigator shall take responsibility for maintaining adequate and accurate hard copy source documents of all observations and data generated during this study. Such documentation is subject to inspection by the Sponsor, its representatives and Regulatory

Authorities. If an Investigator moves, withdraws from an investigation or retires the responsibility for maintaining the records may be transferred to another person who will accept responsibility. Notice of transfer must be made to and agreed by the Sponsor.

9.6 Good Clinical Practice

The procedures set out in this study protocol are designed to ensure that the Sponsor and Investigator abide by the principles of the ICH-GCP Guidelines, and of the Declaration of Helsinki (APPENDIX 1). The study also will be carried out in keeping with local legal requirements.

9.7 Informed Consent

The Investigator is responsible for and will obtain a signed ICF from each subject's parents/guardians, before each subject is admitted to the study, in accordance with the ICH-GCP Guidelines, the Declaration of Helsinki, and applicable regulatory requirements.

This consent form must be dated and retained by the Investigator as part of the study records. The Investigator will not undertake any investigation specifically required only for the clinical study until valid consent has been obtained. The terms of the consent and when it was obtained must be documented in the subject's medical records and the e-CRF.

Subjects/parents/guardians will be informed of the nature of the study, its aim, its possible risks and restrictions, its duration, and the compensation that they might receive. The protocol will be explained during a meeting prior to study enrollment, and each subject/parents/guardians must be informed that participation in the study is voluntary and that the subject may withdraw from the study at any time. The parents/guardians should read the ICF before signing and dating it and a copy of the signed document should be given to the parents/guardians. No subject can enter the study before informed consent has been obtained from her/his parents/guardians. Subjects 7 to 11 years of age will sign an assent form specific to their ages, wherever that is required by local country laws. The parents/guardians of all subjects regardless of age must sign the ICF.

The explicit wish of a minor, who is capable of forming an opinion and assessing the study information, to refuse participation in or to be withdrawn from the study at any time will be respected by the Investigator.

If a protocol amendment is required, the ICF may need to be revised to reflect the changes to the protocol. If the consent form is revised, it must be reviewed and approved by the appropriate IEC/IRB, and signed by the parents/guardians of all subjects subsequently enrolled in the study as well as those currently enrolled in the study.

The Investigator should inform the subject's primary physician about participation in the clinical study wherever required.

A specific written informed consent for the screening of mutations in the filaggrin gene and for evaluating the epigenetic modifications on some specific genes will also be prepared, submitted and approved by the IRBs/IECs, and has to be signed by the subject's parents/guardians before the blood samples are drawn.

9.8 Protocol Approval and Amendment

Before the start of the study, the study protocol and/or other relevant documents will be reviewed and approved by the IEC/IRB/Regulatory Authorities, in accordance with local legal requirements. The Sponsor must ensure that all ethical and legal requirements have been met before the first subject is enrolled in the study.

This protocol is to be followed exactly. Any change to the protocol must be handled as a protocol amendment. Any potential amendment must be approved by the Sponsor. A written amendment must be submitted to the appropriate Regulatory Authorities and to the IRB/IECs assuming this responsibility. The Investigator must await IRB/IEC approval of protocol amendments before implementing the changes, except where necessary to eliminate apparent immediate hazard to subjects. In these cases, the IRB/IEC must be notified within 5 days of the change.

All amendments to the protocol must be approved in writing by both the appropriate Regulatory Authorities and the IRB/IEC, except for administrative amendments, which require notification but not written approval. Once approved, the protocol amendment will be distributed to all recipients of the original protocol, with instructions to append the amendment to the protocol. If, in the judgment of the local IRB/IEC, the Investigator and/or Sponsor, the protocol amendment alters the study design, procedures and/or increases the potential risk to the subject, the currently approved written ICF will require modifications. The modified ICF must also be reviewed and approved by the Sponsor, appropriate Regulatory Authorities, and the IRB/IEC. In such cases, repeat informed consent must be obtained from the enrolled subjects' parents/guardians before participation continues.

9.9 Data and Safety Monitoring Board

A DSMB composed of experts in food allergy and in the methodology of clinical studies will be established in due time for the first data review. This DSMB will be independent of the Sponsor and will review safety data from the study at specified intervals during the study and on an ad hoc basis as deemed necessary by the DSMB Chair person or when conveyed by the Sponsor. A specific DSMB meeting will be held when the first 15 subjects 4 to 5 years of age have been randomized and treated for at least 4 weeks (that is, have completed the Month 1 visit). This is to assess very early in the study that safety of Viaskin® Peanut in these younger patients is acceptable.

The DSMB members will review blinded data, but may have access to unblinded data as deemed necessary during the closed review meetings. During these review meetings, the DSMB will assess whether the nature, frequency, and severity of the AEs associated with the study treatment warrant any recommendations or corrective actions in the best interest of the subjects.

The roles, responsibilities, constitution, and operations of the DSMB will be described in the DSMB Charter, which will be reviewed and signed by each member before the first subject is randomized and treated.

9.10 Duration of the Study

For an individual subject, the maximum duration of study participation will be up to 61 weeks (including up to 6 weeks for screening, 12 months of treatment and up to 3 weeks for follow-up). The planned study duration is 24 months. The study will close when all subjects have completed the Final Follow-up visit.

9.11 Premature Termination of the Study

If the Investigator, the Sponsor, or the Medical Monitor becomes aware of conditions or events that suggest a possible hazard to subjects if the study continues, the study may be terminated after appropriate consultation between the relevant parties. The study may also be terminated early at the Sponsor's discretion in the absence of such a finding.

Conditions that may warrant termination include, but are not limited to:

- The discovery of an unexpected, significant, or unacceptable risk to the subjects enrolled in the study;
- Failure to enroll subjects at an acceptable rate;
- A decision on the part of the Sponsor to suspend or discontinue development of the drug;
- A decision from the Regulatory Authorities to suspend or discontinue the study.

In terminating the study, the Sponsor, PAREXEL and the Investigator will ensure that adequate consideration is given to the protection of the subjects' interests.

9.12 Confidentiality

All information obtained during the conduct of the study with respect to the subject's state of health will be regarded as confidential. For disclosure of any such information, an agreement will be obtained in writing. The Investigator must ensure that each subject's anonymity is maintained. On e-CRFs and other documents submitted to the Sponsor or PAREXEL, subjects must not be identified by name. Instead, subjects will only be known by the unique subject screening number allocated to them in order to ensure confidentiality on all study documentation. Subjects will retain this unique number throughout the study. The Investigator will keep a separate log of these codes.

In order to comply with government regulatory guidelines and to ensure subject safety, it may be necessary for the Sponsor and its representative, the PAREXEL personnel, the IEC/IRB, or Regulatory Authority to review subjects' medical records as they relate to this study. Only the subject's unique number in the e-CRFs will identify her/him, but their full names may be made known to a Regulatory Authority or other authorized government or health care officials, if necessary, and to personnel designated by the Sponsor.

Documents that are not for submission to the Sponsor or to PAREXEL (for example consent forms) will be maintained by the Investigator in strict confidence, except to the extent necessary to allow monitoring by the Sponsor and PAREXEL, and auditing by regulatory authorities. No documents identifying subjects by name will leave the investigative site and subject identity will remain confidential in all publications related to the study.

9.13 Contractual and Financial Details

The Investigator (and/or, as appropriate, the hospital administrative representative) and the Sponsor will sign a clinical study agreement prior to the start of the study, outlining overall Sponsor and Investigator responsibilities in relation to the study. The contract should describe whether costs for pharmacy, laboratory and other protocol-required services are being paid directly or indirectly.

Financial Disclosure Statements will need to be completed, as requested by FDA CFR 21 part 54.

9.14 Liability and Insurance

The Sponsor will take out reasonable third-party liability insurance cover in accordance with all local legal requirements. The civil liability of the Investigator, the persons instructed by her/him and the hospital, practice, or institute in which they are employed and the liability of the Sponsor with respect to financial loss due to personal injury and other damage that may arise as a result of the carrying out of this study are governed by the applicable law.

Deviations from the study protocol, especially the prescription of a dose other than that scheduled in the study protocol, other modes of administration, other indications, and longer treatment periods, are not permitted and shall not be covered by the statutory subject insurance scheme.

The Sponsor will arrange for subjects participating in this study to be insured against financial loss due to personal injury caused by the pharmaceutical products being tested or by medical steps taken in the course of the study.

9.15 Publication Policy

By signing the study protocol, the Investigator agrees with the use of results of the study for the purposes of national and international registration, publication and information for medical and pharmaceutical professionals. If necessary, Regulatory Authorities will be notified of the Investigator's name, address, qualifications and extent of involvement.

An Investigator shall not publish any data (poster, abstract, paper, etc.) without having consulted with the Sponsor in advance and having received a written approval for such a publication. Details are provided in a separate document.

9.16 Study Center File Management

The Investigator is responsible for assuring that the Study Center File is maintained. The Study Center File will contain, but will not be limited to, the information listed below:

1. IB;
2. Current, signed version of the protocol and any previous versions of the protocol;
3. Protocol amendments (if applicable);
4. Operations Manual (if applicable);
5. Current ICF (blank) and any previous versions of the ICF;
6. Curricula Vitae of Investigator(s) and sub-investigator(s) and photocopy of their respective license(s) where required by law; Original US FDA Form 1572 (for all

studies conducted under US Investigational New Drug [IND] regulations), signed by all Principal Investigators. The names of any sub-investigators must appear on this form. Investigators must also complete all regulatory documentation as required the ICH GCP and by local or national regulations;

7. Documentation of IRB/IEC approval of the protocol, the ICF, any protocol amendments, and any ICF revisions;
8. All correspondence between the Investigator, IRB/IEC, and the Sponsor/ PAREXEL relating to study conduct;
9. Laboratory Manual and certification(s);
10. Monitoring log;
11. Study drug accountability forms;
12. Signature list of all staff completing e-CRFs;
13. Signature list of all staff completing drug accountability summaries; and
14. Delegation log.

9.17 Clinical Study Report

A final CSR will be prepared according to the ICH E3 guideline on Structure and Contents of CSRs. A final CSR will be prepared regardless of whether the study is completed or prematurely terminated. The Sponsor will provide each Investigator with a copy of the final report or synopsis for retention.

10 REFERENCE LIST

1 Bock SA, Muñoz-Furlong A, Sampson HA. Further fatalities caused by anaphylactic reactions to food, 2001-2006. J Allergy Clin Immunol. 2007;119(4):1016-8.

2 Bock SA, Muñoz-Furlong A, Sampson HA. Fatalities due to anaphylactic reactions to foods. J Allergy Clin Immunol. 2001;107(1):191-3.

3 Sicherer SH, Muñoz-Furlong A, Godbold JH, Sampson HA. US prevalence of self-reported peanut, tree nut, and sesame allergy: 11-year follow-up. J Allergy Clin Immunol. 2010;125(6):1322-6.

4 Sicherer SH, Muñoz-Furlong A, Sampson HA. Prevalence of peanut and tree nut allergy in the United States determined by means of a random digit dial telephone survey: a 5-year follow-up study. J Allergy Clin Immunol. 2003;112(6):1203-7.

5 Grundy J, Matthews S, Bateman B, Dean T, Arshad SH. Rising prevalence of allergy to peanut in children: Data from 2 sequential cohorts. J Allergy Clin Immunol. 2002;110(5):784-9.

6 Peters RL, Allen KJ, Dharmage SC, Koplin JJ, Dang T, Tilbrook KP, et al.; HealthNuts study. Natural history of peanut allergy and predictors of resolution in the first 4 years of life: A population-based assessment. J Allergy Clin Immunol. 2015;135(5):1257-1266.

7 Ho MH, Wong WH, Heine RG, Hosking CS, Hill DJ, Allen KJ. Early clinical predictors of remission of peanut allergy in children. J Allergy Clin Immunol. 2008;121(3):731-6.

8 Fleischer DM, Conover-Walker MK, Christie L, Burks AW, Wood RA. The natural progression of peanut allergy: Resolution and the possibility of recurrence. J Allergy Clin Immunol. 2003;112(1):183-9.

9 Hourihane JO, Roberts SA, Warner JO. Resolution of peanut allergy: case-control study. BMJ 1998;316(7140):1271-1275.

10 Du Toit G, Roberts G, Sayre PH, Bahnson HT, Radulovic S, Santos AF, et al; LEAP Study Team. Randomized trial of peanut consumption in infants at risk for peanut allergy. N Engl J Med. 2015;372(9):803-13.

11 Eigenmann PA, Beyer K, Wesley Burks A, Lack G, Liacouras CA, Hourihane JO, et al. New visions for food allergy: an iPAC summary and future trends. Pediatr Allergy Immunol. 2008;Suppl 19:26-39.

12 Sampson HA. Food allergy--accurately identifying clinical reactivity. Allergy. 2005;60 Suppl 79:19-24.

13 Sampson HA, Muñoz-Furlong A, Campbell RL, Adkinson NF Jr, Bock SA, Branum A, et al. Second symposium on the definition and management of anaphylaxis: summary report—second National Institute of Allergy and Infectious Disease/Food Allergy and Anaphylaxis Network symposium. Ann Emerg Med. 2006;47(4):373-80.

14 Burks AW. Peanut allergy. Lancet. 2008;371(9623):1538-46.

15 Eigenmann PA. Mechanisms of food allergy. Pediatr Allergy Immunol. 2009;20(1):5-11.

16 Otsu K, Dreskin SC. Peanut allergy: an evolving clinical challenge. Discov Med. 2011;12(65):319-28.

17 Sicherer SH, Furlong TJ, Muñoz-Furlong A, Burks AW, Sampson HA. A voluntary registry for peanut and tree nut allergy: characteristics of the first 5149 registrants. J Allergy Clin Immunol. 2001;108(1):128-32.

18 Kemp SF, Lockey RF, Simons FE; World Allergy Organization ad hoc Committee on Epinephrine in Anaphylaxis. Epinephrine: the drug of choice for anaphylaxis-a statement of the world allergy organization. World Allergy Organ J. 2008;1(7 Suppl):S18-26.

19 Leung DY, Sampson HA, Yunginger JW, Burks AW Jr, Schneider LC, Wortel CH, et al.; Avon Longitudinal Study of Parents and Children Study Team. Effect of anti-IgE therapy in patients with peanut allergy. N Engl J Med. 2003;348(11):986-93.

20 Sampson HA, Leung DY, Burks AW, Lack G, Bahna SL, Jones SM, et al. A phase II, randomized, double-blind, parallel-group, placebo-controlled oral food challenge trial of Xolair (omalizumab) in peanut allergy. J Allergy Clin Immunol. 2011;127(5):1309-10.

21 Jones SM, Burks AW, Dupont C. State of the art on food allergen immunotherapy: oral, sublingual, and epicutaneous. J Allergy Clin Immunol. 2014;133(2):318-23.

22 Leung DY. Food allergy: are we getting closer to a cure? J Allergy Clin Immunol. 2011;127(3):555-7.

23 Anagnostou K, Islam S, King Y, Foley L, Pasea L, Bond S, et al. Assessing the efficacy of oral immunotherapy for the desensitisation of peanut allergy in children (STOP II): a phase 2 randomised controlled trial. Lancet. 2014;383(9925):1297-304.

24 Kim EH, Bird JA, Kulis M, Laubach S, Pons L, Shreffler W, et al. Sublingual immunotherapy for peanut allergy: clinical and immunologic evidence of desensitization. J Allergy Clin Immunol. 2011;127(3):640-6.e1.

25 Varshney P, Jones SM, Scurlock AM, Perry TT, Kemper A, Steele P, et al. A randomized controlled study of peanut oral immunotherapy: clinical desensitization and modulation of the allergic response. J Allergy Clin Immunol. 2011;127(3):654-60.

26 Jones SM, Pons L, Roberts JL, Scurlock AM, Perry TT, Kulis M, et al. Clinical efficacy and immune regulation with peanut oral immunotherapy. J Allergy Clin Immunol. 2009;124(2):292-300.

27 Blumchen K, Ulbricht H, Staden U, Dobberstein K, Beschorner J, de Oliveira LC, et al. Oral peanut immunotherapy in children with peanut anaphylaxis. J Allergy Clin Immunol. 2010;126(1):83-91.e1.

28 Vickery BP, Scurlock AM, Steele P, Kamilaris J, Hiegel AM, Carlisle SK, et al. Early and Persistent Gastrointestinal Side Effects Predict Withdrawal from Peanut Oral Immunotherapy (OIT). J Allergy Clin Immunol. 2011;127(2):AB26.

29 Wasserman RL, Factor JM, Baker JW, Mansfield LE, Katz Y, Hague AR, et al. Oral immunotherapy for peanut allergy: multipractice experience with epinephrine-treated reactions. J Allergy Clin Immunol Pract. 2014;2(1):91-6.

30 Wasserman RL, Sugerman RW, Mireku-Akomeah N, Gallucci AR, Pence DM, Long NA. Peanut Oral Immunotherapy (OIT) of Food Allergy (FA) Carries a Significant Risk of Eosinophilic Esophagitis (EoE). J Allergy Clin Immunol. 2011;127(2):AB28.

31 Hsieh FH. Oral food immunotherapy and iatrogenic eosinophilic esophagitis: an acceptable level of risk? Ann Allergy Asthma Immunol. 2014;113(6):581-2.

32 Lucendo AJ, Arias A, Tenias JM. Relation between eosinophilic esophagitis and oral immunotherapy for food allergy: a systematic review with meta-analysis. Ann Allergy Asthma Immunol. 2014;113(6):624-9.

33 Investigator's Brochure. DBV712. Edition Number 7, 18 Aug 2015.

34 International Conference on Harmonisation of Technical Requirements for Registration of Pharmaceuticals for Human Use. ICH Harmonised Tripartite Guideline. General Considerations for Clinical Trials – E8. Version dated 17 July 1997. Available from www.ich.org.

35 Niggemann B, Beyer K. Diagnosis of food allergy in children: toward a standardization of food challenge. J Pediatr Gastroenterol Nutr. 2007;45(4):399-404.

36 Kulis M, Vickery BP, Burks AW. Pioneering immunotherapy for food allergy: clinical outcomes and modulation of the immune response. Immunol Res. 2011;49(1-3):216-26.

37 Taylor SL, Moneret-Vautrin DA, Crevel RW, Sheffield D, Morisset M, Dumont P, et al. Threshold dose for peanut: Risk characterization based upon diagnostic oral challenge of a series of 286 peanut-allergic individuals. Food Chem Toxicol. 2010;48(3):814-9.

38 Kim EH, Bird JA, Kulis M, Laubach S, Pons L, Shreffler W, et al. Sublingual immunotherapy for peanut allergy: clinical and immunologic evidence of desensitization. J Allergy Clin Immunol. 2011;127(3):640-6.

39 Beydon N, Davis SD, Lombardi E, Allen JL, Arets HG, Aurora P, et al; American Thoracic Society/European Respiratory Society Working Group on Infant and Young Children Pulmonary Function Testing. An official American Thoracic Society/European Respiratory Society statement:

pulmonary function testing in preschool children. Am J Respir Crit Care Med. 2007;175(12):1304-45.

40 National Heart, Lung, and Blood Institute. National Asthma Education and Prevention Program Expert Panel Report 3: Guidelines for the Diagnosis and Management of Asthma. Bethesda, MD: National Institutes of Health; 2007.

41 Global Initiative for Asthma. Global Strategy for Asthma Management and Prevention. Available from www.ginasthma.org. Date last updated: April 2015.

42 Sampson HA, Muñoz-Furlong A, Campbell RL, Adkinson NF Jr, Bock SA, Branum A, et al. Second symposium on the definition and management of anaphylaxis: summary report—Second National Institute of Allergy and Infectious Disease/Food Allergy and Anaphylaxis Network symposium. J Allergy Clin Immunol. 2006;117(2):391-7.

43 Turjanmaa K, Darsow U, Niggemann B, Rancé F, Vanto T, Werfel T. EAACI/GA2LEN position paper: present status of the atopy patch test. Allergy. 2006;61(12):1377-84.

44 Flokstra-de Blok, B.M.J. Food Allergy Quality of Life Questionnaires (FAQLQ). In: Encyclopedia of Quality of Life and Well-Being Research. Michalos, A.C. (Editor). 2014, 2319-22.

45 van der Velde JL, Flokstra-de Blok BM, Vlieg-Boerstra BJ, Oude Elberink JN, DunnGalvin A, Hourihane JO, et al. Development, validity and reliability of the food allergy independent measure (FAIM). Allergy. 2010;65(5):630-5.

46 Kunz B, Oranje AP, Labreze L, Staldor JF, Ring J, Taieb A. Clinical validation and guidelines for the SCORAD Index: Consensus Report of the European Task Force on Atopic Dermatitis. Dermatology 1997;195(1):10-9.

APPENDICES

APPENDIX 1: Declaration of Helsinki

APPENDIX 2: Dosages of Inhaled Corticosteroids

APPENDIX 3: Activity of Corticosteroids

APPENDIX 4: Wash-out periods of Short-acting and Long-acting Antihistamines based on Terminal Elimination Half-Lives

APPENDIX 5: Anaphylaxis Staging System

APPENDIX 6: Oral Food Challenge Symptom Score Sheet for the PEPITES Study

APPENDIX 7: FAQLQ/FAIM Questionnaires

APPENDIX 8: SCORAD

APPENDIX 1

Declaration of Helsinki

WORLD MEDICAL ASSOCIATION DECLARATION OF HELSINKI
Ethical Principles for Medical Research Involving Human Subjects

Adopted by the 18th WMA General Assembly, Helsinki, Finland, June 1964 and amended by the:
29th WMA General Assembly, Tokyo, Japan, October 1975
35th WMA General Assembly, Venice, Italy, October 1983
41st WMA General Assembly, Hong Kong, September 1989
48th WMA General Assembly, Somerset West, Republic of South Africa, October 1996
52nd WMA General Assembly, Edinburgh, Scotland, October 2000
53rd WMA General Assembly, Washington DC, USA, October 2002 (Note of Clarification added)
55th WMA General Assembly, Tokyo, Japan, October 2004 (Note of Clarification added)
59th WMA General Assembly, Seoul, Republic of Korea, October 2008
64th WMA General Assembly, Fortaleza, Brazil, October 2013

Preamble

1. The World Medical Association (WMA) has developed the Declaration of Helsinki as a statement of ethical principles for medical research involving human subjects, including research on identifiable human material and data.

The Declaration is intended to be read as a whole and each of its constituent paragraphs should be applied with consideration of all other relevant paragraphs.

2. Consistent with the mandate of the WMA, the Declaration is addressed primarily to physicians. The WMA encourages others who are involved in medical research involving human subjects to adopt these principles.

General Principles

3. The Declaration of Geneva of the WMA binds the physician with the words, “The health of my patient will be my first consideration,” and the International Code of Medical Ethics declares that, “A physician shall act in the patient's best interest when providing medical care.”

4. It is the duty of the physician to promote and safeguard the health, well-being and rights of patients, including those who are involved in medical research. The physician's knowledge and conscience are dedicated to the fulfilment of this duty.

5. Medical progress is based on research that ultimately must include studies involving human subjects.

6. The primary purpose of medical research involving human subjects is to understand the causes, development and effects of diseases and improve preventive, diagnostic and therapeutic interventions (methods, procedures and treatments). Even the best proven interventions must be evaluated continually through research for their safety, effectiveness, efficiency, accessibility and quality.

7. Medical research is subject to ethical standards that promote and ensure respect for all human subjects and protect their health and rights.

8. While the primary purpose of medical research is to generate new knowledge, this goal can never take precedence over the rights and interests of individual research subjects.

9. It is the duty of physicians who are involved in medical research to protect the life, health, dignity, integrity, right to self-determination, privacy, and confidentiality of personal information of research subjects. The responsibility for the protection of research subjects must always rest with the physician or other health care professionals and never with the research subjects, even though they have given consent.

10. Physicians must consider the ethical, legal and regulatory norms and standards for research involving human subjects in their own countries as well as applicable international norms and standards. No national or international ethical, legal or regulatory requirement should reduce or eliminate any of the protections for research subjects set forth in this Declaration.

11. Medical research should be conducted in a manner that minimises possible harm to the environment.

12. Medical research involving human subjects must be conducted only by individuals with the appropriate ethics and scientific education, training and qualifications. Research on patients or healthy volunteers requires the supervision of a competent and appropriately qualified physician or other health care professional.

13. Groups that are underrepresented in medical research should be provided appropriate access to participation in research.

14. Physicians who combine medical research with medical care should involve their patients in research only to the extent that this is justified by its potential preventive, diagnostic or therapeutic value and if the physician has good reason to believe that participation in the research study will not adversely affect the health of the patients who serve as research subjects.

15. Appropriate compensation and treatment for subjects who are harmed as a result of participating in research must be ensured.

Risks, Burdens and Benefits

16. In medical practice and in medical research, most interventions involve risks and burdens.

Medical research involving human subjects may only be conducted if the importance of the objective outweighs the risks and burdens to the research subjects.

17. All medical research involving human subjects must be preceded by careful assessment of predictable risks and burdens to the individuals and groups involved in the research in comparison with foreseeable benefits to them and to other individuals or groups affected by the condition under investigation.

Measures to minimise the risks must be implemented. The risks must be continuously monitored, assessed and documented by the researcher.

18. Physicians may not be involved in a research study involving human subjects unless they are confident that the risks have been adequately assessed and can be satisfactorily managed.

When the risks are found to outweigh the potential benefits or when there is conclusive proof of definitive outcomes, physicians must assess whether to continue, modify or immediately stop the study.

Vulnerable Groups and Individuals

19. Some groups and individuals are particularly vulnerable and may have an increased likelihood of being wronged or of incurring additional harm.

All vulnerable groups and individuals should receive specifically considered protection.

20. Medical research with a vulnerable group is only justified if the research is responsive to the health needs or priorities of this group and the research cannot be carried out in a non-vulnerable group. In addition, this group should stand to benefit from the knowledge, practices or interventions that result from the research.

Scientific Requirements and Research Protocols

21. Medical research involving human subjects must conform to generally accepted scientific principles, be based on a thorough knowledge of the scientific literature, other relevant sources of information, and adequate laboratory and, as appropriate, animal experimentation. The welfare of animals used for research must be respected.

22. The design and performance of each research study involving human subjects must be clearly described and justified in a research protocol.

The protocol should contain a statement of the ethical considerations involved and should indicate how the principles in this Declaration have been addressed. The protocol should include information regarding funding, sponsors, institutional affiliations, potential conflicts of interest, incentives for subjects and information regarding provisions for treating and/or compensating subjects who are harmed as a consequence of participation in the research study.

In clinical trials, the protocol must also describe appropriate arrangements for post-trial provisions.

Research Ethics Committees

23. The research protocol must be submitted for consideration, comment, guidance and approval to the concerned research ethics committee before the study begins. This committee must be transparent in its functioning, must be independent of the researcher, the sponsor and any other undue influence and must be duly qualified. It must take into consideration the laws and regulations of the country or countries in which the research is to be performed as well as applicable international norms and standards but these must not be allowed to reduce or eliminate any of the protections for research subjects set forth in this Declaration.

The committee must have the right to monitor ongoing studies. The researcher must provide monitoring information to the committee, especially information about any serious adverse events. No amendment to the protocol may be made without consideration and approval by the committee. After the end of the study, the researchers must submit a final report to the committee containing a summary of the study's findings and conclusions.

Privacy and Confidentiality

24. Every precaution must be taken to protect the privacy of research subjects and the confidentiality of their personal information.

Informed Consent

25. Participation by individuals capable of giving informed consent as subjects in medical research must be voluntary. Although it may be appropriate to consult family members or community leaders, no individual capable of giving informed consent may be enrolled in a research study unless he or she freely agrees.

26. In medical research involving human subjects capable of giving informed consent, each potential subject must be adequately informed of the aims, methods, sources of funding, any possible conflicts of interest, institutional affiliations of the researcher, the anticipated benefits and potential risks of the study and the discomfort it may entail, post-study provisions and any other relevant aspects of the study. The potential subject must be informed of the right to refuse to participate in the study or to withdraw consent to participate at any time without reprisal. Special attention should be given to the specific information needs of individual potential subjects as well as to the methods used to deliver the information.

After ensuring that the potential subject has understood the information, the physician or another appropriately qualified individual must then seek the potential subject's freely-given informed consent, preferably in writing. If the consent cannot be expressed in writing, the non-written consent must be formally documented and witnessed.

All medical research subjects should be given the option of being informed about the general outcome and results of the study.

27. When seeking informed consent for participation in a research study the physician must be particularly cautious if the potential subject is in a dependent relationship with the physician or may consent under duress. In such situations the informed consent must be sought by an appropriately qualified individual who is completely independent of this relationship.

28. For a potential research subject who is incapable of giving informed consent, the physician must seek informed consent from the legally authorised representative. These individuals must not be included in a research study that has no likelihood of benefit for them unless it is intended to promote the health of the group represented by the potential subject, the research cannot instead be performed with persons capable of providing informed consent, and the research entails only minimal risk and minimal burden.

29. When a potential research subject who is deemed incapable of giving informed consent is able to give assent to decisions about participation in research, the physician must seek that assent in addition to the consent of the legally authorised representative. The potential subject's dissent should be respected.

30. Research involving subjects who are physically or mentally incapable of giving consent, for example, unconscious patients, may be done only if the physical or mental condition that prevents giving informed consent is a necessary characteristic of the research group. In such circumstances the physician must seek informed consent from the legally authorised representative. If no such representative is available and if the research cannot be delayed, the study may proceed without informed consent provided that the specific reasons for involving subjects with a condition that renders them unable to give informed consent have been stated in the research protocol and the study has been approved by a research ethics committee. Consent to remain in the research must be obtained as soon as possible from the subject or a legally authorised representative.

31. The physician must fully inform the patient which aspects of their care are related to the research. The refusal of a patient to participate in a study or the patient's decision to withdraw from the study must never adversely affect the patient-physician relationship.

32. For medical research using identifiable human material or data, such as research on material or data contained in biobanks or similar repositories, physicians must seek informed consent for its collection, storage and/or reuse. There may be exceptional situations where consent would be impossible or impracticable to obtain for such research. In such situations the research may be done only after consideration and approval of a research ethics committee.

Use of Placebo

33. The benefits, risks, burdens and effectiveness of a new intervention must be tested against those of the best proven intervention(s), except in the following circumstances:

Where no proven intervention exists, the use of placebo, or no intervention, is acceptable; or

Where for compelling and scientifically sound methodological reasons the use of any intervention less effective than the best proven one, the use of placebo, or no intervention is necessary to determine the efficacy or safety of an intervention

and the patients who receive any intervention less effective than the best proven one, placebo, or no intervention will not be subject to additional risks of serious or irreversible harm as a result of not receiving the best proven intervention.

Extreme care must be taken to avoid abuse of this option.

Post-Trial Provisions

34. In advance of a clinical trial, sponsors, researchers and host country governments should make provisions for post-trial access for all participants who still need an intervention identified

as beneficial in the trial. This information must also be disclosed to participants during the informed consent process.

Research Registration and Publication and Dissemination of Results

35. Every research study involving human subjects must be registered in a publicly accessible database before recruitment of the first subject.

36. Researchers, authors, sponsors, editors and publishers all have ethical obligations with regard to the publication and dissemination of the results of research. Researchers have a duty to make publicly available the results of their research on human subjects and are accountable for the completeness and accuracy of their reports. All parties should adhere to accepted guidelines for ethical reporting. Negative and inconclusive as well as positive results must be published or otherwise made publicly available. Sources of funding, institutional affiliations and conflicts of interest must be declared in the publication. Reports of research not in accordance with the principles of this Declaration should not be accepted for publication.

Unproven Interventions in Clinical Practice

37. In the treatment of an individual patient, where proven interventions do not exist or other known interventions have been ineffective, the physician, after seeking expert advice, with informed consent from the patient or a legally authorised representative, may use an unproven intervention if in the physician's judgement it offers hope of saving life, re-establishing health or alleviating suffering. This intervention should subsequently be made the object of research, designed to evaluate its safety and efficacy. In all cases, new information must be recorded and, where appropriate, made publicly available.

APPENDIX 2

Dosages of Inhaled Corticosteroids

Children ≤4 years of age	Low Daily Dose	Medium Daily Dose	High Daily Dose
**Beclomethasone HFA** 40 or 80 mcg/puff	NA	NA	NA
**Budesonide DPI** 90, 180, or 200 mcg/inhalation	NA	NA	NA
**Budesonide Inhaled** Inhalation suspension for nebulization	0.25-0.5 mg	>0.5-1.0 mg	>1.0 mg
**Flunisolide** 250 mcg/puff	NA	NA	NA
**Flunisolide HFA** 80 mcg/puff	NA	NA	NA
**Fluticasone HFA/MDI** 44, 110, or 220 mcg/puff	176 mcg	>176-352 mcg	>352 mcg
**DPI** 50, 100, or 250 mcg/inhalation	NA	NA	NA
**Mometasone DPI** 200 mcg/inhalation	NA	NA	NA
**Triamcinolone acetonide** 75 mcg/puff	NA	NA	NA

Children 5 to 11 years of age	Low Daily Dose	Medium Daily Dose	High Daily Dose
**Beclomethasone HFA** 40 or 80 mcg/puff	80–160 mcg	>160–320 mcg	>320 mcg
**Budesonide DPI** 90, 180, or 200 mcg/inhalation	180–400 mcg	>400–800 mcg	>800 mcg
**Budesonide Inhaled** Inhalation suspension for nebulization	0.5 mg	1.0 mg	2.0 mg
**Flunisolide** 250 mcg/puff	500–750 mcg	1,000–1,250 mcg	>1,250 mcg
**Flunisolide HFA** 80 mcg/puff	160 mcg	320 mcg	≥640 mcg
**Fluticasone HFA/MDI** 44, 110, or 220 mcg/puff	88–176 mcg	>176–352 mcg	>352 mcg
**DPI** 50, 100, or 250 mcg/inhalation	100–200 mcg	>200–400 mcg	>400 mcg
**Mometasone DPI** 200 mcg/inhalation	NA	NA	NA
**Triamcinolone acetonide** 75 mcg/puff	300–600 mcg	>600–900 mcg	>900 mcg

Abbreviations: DPI = Dry powder inhaler; HFA = Hydrofluoroalkane; MDI = Metered-dose inhaler; NA=Not approved and no data available for this age category.

**Adolescents ≥12 years of age and Adults**	**Low Daily Dose**	**Medium Daily Dose**	**High Daily Dose**
**Beclomethasone HFA**<br>40 or 80 mcg/puff	80–240 mcg	>240–480 mcg	>480 mcg
**Budesonide DPI**<br>90, 180, or 200 mcg/inhalation	180–600 mcg	>600–1,200 mcg	>1,200 mcg
**Budesonide Inhaled**<br>Inhalation suspension for nebulization	NA	NA	NA
**Flunisolide**<br>250 mcg/puff	500–1,000 mcg	>1,000–2,000 mcg	>2,000 mcg
**Flunisolide HFA**<br>80 mcg/puff	320 mcg	>320–640 mcg	>640 mcg
**Fluticasone HFA/MDI**<br>44, 110, or 220 mcg/puff	88–264 mcg	>264–440 mcg	>440 mcg
**DPI**<br>50, 100, or 250 mcg/inhalation	100–300 mcg	>300–500 mcg	>500 mcg
**Mometasone DPI** 200 mcg/inhalation	200 mcg	400 mcg	>400 mcg
**Triamcinolone acetonide** 75 mcg/puff	300–750 mcg	>750–1,500 mcg	>1,500 mcg

Abbreviations: DPI = Dry powder inhaler; HFA = Hydrofluoroalkane; MDI = Metered-dose inhaler; NA=Not approved and no data available for this age category.

Source: National Heart, Lung, and Blood Institute. Expert panel report 3: guidelines for the diagnosis and management of asthma—full report 2007. August 28, 2007. Available at: www.nhlbi.nih.gov/guidelines/asthma/asthgdln.pdf. Accessed July 06, 2015.

APPENDIX 3

Activity of Corticosteroids

CORTICOSTEROID	*ACTIVITY*	*RELATIVE POTENCY*	*EQUIVALENT DOSE (MG)*
**Dexamethasone**	**Long-acting**	**25**	**0.75**
**Prednisone**	**Intermediate-acting**	**4**	**5.0**
**Methylprednisolone**	**Intermediate -acting**	**5**	**4.0**
**Hydrocortisone**	**Short-acting**	**1.0**	**20.0**

Information from Drug facts and comparisons. St Louis: Facts and Comparisons, 1997:122-23.

APPENDIX 4

Wash-out periods for Short-acting and Long-acting Antihistamines based on Terminal Elimination Half-Lives

**Short-acting antihistamines**	Terminal elimination half-life (h)	Minimum days of wash-out prior to skin prick tests or oral food challenges (days)
Oral		
acrivastine[1]	2-4	1[3] or ideally 3
cetirizine[1]	6.2 - 10	Must be 5[4]
cimetidine[1]	1.4	1[3] or ideally 3
diphenhydramine[1]	2-8	1[3] or ideally 3
famotidine[1]	0.8-5.8	1[3] or ideally 3
fexofenadine[1]	14.4	3
hydroxyzine[1]	4.8-9.4	Must be 5[4]
levocetirizine[1]	6	3
nizatadine[1]	1-2	1[3] or ideally 3
mizolastine[2]	12.9	3
ranitidine[1]	2.5-3	1[3] or ideally 3
rupatadine[2]	13	3
Intranasal/Ophthalmic		
emedastine[1]	3-4	1[3] or ideally 3
epinastine[1]	12	3
olopatadine[1]	8-12	3

**Long-acting antihistamines**	Terminal elimination half-life (h)	Wash-out period prior to skin prick tests or oral food challenges (days)
Oral		
desloratadine[1]	27	7
ebastine[2]	19.3	5
chlorpheniramine[1]	6.3-23.1	6
ketotifen[1]	21	5
loratadine[1]	28	7
Intranasal/Ophthalmic		
azelastine[1]	22-25	6
ketotifen[1]	21	5
levocabastine[1]	35-40	7

[1] *Lexicomp Online®, In: UpToDate, Waltham, MA. (Accessed on November 17, 2015.)*

[2] Derived from: *Allergy: Principles and Practice* (Middleton, 7th Edition) in the form of Table 87.4 (authored by FER Simons and C Akdis).

[3] *If the antihistamine is used the day prior to an oral food challenge, a Skin Prick Test must be performed before the initiation of the challenge and the result must be positive, i.e. a genuine wheal with the histamine positive control must be obtained before the challenge is effectively initiated. If not, postpone the challenge.*

[4] *Wash-out period extended beyond 3 days based on actual clinical experience.*

APPENDIX 5

Anaphylaxis Staging System

Anaphylaxis is a generalized allergic reaction that is rapid in onset and may progress to death (42).

Staging System of Severity of Anaphylaxis	
**Stage**	**Defined By**
1. *Mild (skin & subcutaneous tissues, GI, &/or mild respiratory)*	Flushing, urticaria, periorbital or facial angioedema; mild dyspnea, wheeze or upper respiratory symptoms; mild abdominal pain and/or emesis
2. *Moderate* (mild symptoms + features suggesting moderate respiratory, cardiovascular or GI symptoms)	Marked dysphagia, hoarseness, and/or stridor; shortness of breath , wheezing & retractions; crampy abdominal pain, recurrent vomiting and/or diarrhea; and/or mild dizziness
3. *Severe* (hypoxia, hypotension, or neurological compromise)	Cyanosis or $SpO_2 \leq 92\%$ at any stage, hypotension, confusion, collapse, loss of consciousness; or incontinence

APPENDIX 6

Oral Food Challenge Symptom Score Sheet for the PEPITES Study (Displayed by Categories I to V)

I. SKIN

A. Erythematous Rash- % area involved (see diagram below): ______________________

B. Pruritus

- 0 = Absent
- 1 = Mild: occasional scratching
- 2 = Moderate: scratching continuously for >2 minutes at a time
- 3 = Severe: hard continuous scratching excoriations

C. Urticaria/Angioedema

- 0 = Absent
- 1 = Mild: <3 hives, or mild lip edema
- 2 = Moderate: <10 hives but >3, or significant lip or face edema
- 3 = Severe: generalized involvement

D. Rash

- 0 = Absent
- 1 = Mild: few areas of faint erythema
- 2 = Moderate: areas of erythema (>20% and <50%), macular and raised rash
- 3 = Severe: generalized marked erythema (>50%), extensive raised lesions (>25%)

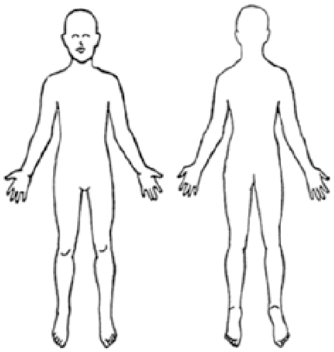

Head	4.5%
Neck	1%
Anterior trunk	18%
Posterior trunk	18%
Leg	18%
Arm	9%

II. UPPER RESPIRATORY

A. Sneezing/Itching

- 0 = Absent
- 1 = Mild: rare bursts
- 2 = Moderate: bursts <10, intermittent rubbing of nose / eyes / external ear canals
- 3 = Severe: continuous rubbing of nose / eyes, periocular swelling and/or long bursts of sneezing

B. Nasal Congestion

- 0 = Absent
- 1 = Mild: some hindrance to breathing

2 = Moderate: nostrils feel blocked, breathing through mouth most of time
3 = Severe: nostrils occluded

C. Rhinorrhea
0 = Absent
1 = Mild: occasional sniffling
2 = Moderate: frequent sniffling, requires tissues
3 = Severe: nose runs freely despite sniffling and tissues

D. Laryngeal
0 = Absent
1 = Mild: throat clearing, occasional cough
2 = Moderate: hoarseness, frequent dry cough
3 = Severe: inspiratory stridor

III. LOWER RESPIRATORY

A. Wheezing
0 = Absent
1 = Mild: expiratory wheezing to auscultation
2 = Moderate: dyspnea, inspiratory and expiratory wheezing
3 = Severe: dyspnea, use of accessory muscles, audible wheezing

IV. GASTROINTESTINAL

A. Subjective Complaints
0 = Absent
1 = Mild: itchy mouth/throat, c/o nausea, abdominal pain, no change in activity
2 = Moderate: frequent c/o nausea or abdominal pain, decreased activity
3 = Severe: patient in bed; crying, notably distressed

B. Objective Complaints
0 = Absent
1 = Mild: 1 episode of emesis or diarrhea
2 = Moderate: 2-3 episodes of emesis or diarrhea or 1 of each
3 = Severe: >3 episodes of emesis or diarrhea or 2 of each

V. CARDIOVASCULAR/NEUROLOGIC
0 = Normal: heart rate or BP for age/baseline
1 = Mild: color change, subjective response (weak, dizzy), or tachycardia, mental status change, mild hypotension (weak rapid pulse and/or 10-20% drop in blood pressure from baseline)
2 = Moderate: drop in blood pressure >20% from baseline, significant change in mental status, light-headedness, feeling of “pending doom”
3 = Severe: cardiovascular collapse, signs of impaired circulation, unconsciousness, bradycardia, cardiac arrest.

APPENDIX 7

FAQLQ/FAIM Questionnaires

English – Child version

FAQLQ-CF

Food Allergy Quality of Life Questionnaire – Child Form (8-12 years)

EuroPrevall

To cite this questionnaire:
Flokstra-de Blok BMJ, DunnGalvin A, Vlieg-Boerstra BJ, Oude Elberink JNG, Duiverman EJ, Hourihane JO, Dubois AEJ. Development and validation of a self-administered Food Allergy Quality of Life Questionnaire for children. Clin Exp Allergy 2009 Jan;39(1):127-137.

The questions are about the influence of your food allergy on your quality of life. It is important that you fill in the answers yourself. You may ask your parents for help, but they are not allowed to tell you which answer to give. Answer every question by putting an 'x' in the proper box. You may choose from the following answers.

not | barely | a little bit | fairly | quite | very | extremely

	**How *troublesome* do you find it, because of your food allergy, that you ...**							
1	must always watch what you eat?	☐	☐	☐	☐	☐	☐	☐
2	can eat fewer things?	☐	☐	☐	☐	☐	☐	☐
3	are limited in buying things you like?	☐	☐	☐	☐	☐	☐	☐
4	have to read labels?	☐	☐	☐	☐	☐	☐	☐
5	have to refuse food when you do things with others?	☐	☐	☐	☐	☐	☐	☐
6	can less easily stay for a meal with someone?	☐	☐	☐	☐	☐	☐	☐
7	can taste or try fewer things when eating out?	☐	☐	☐	☐	☐	☐	☐
8	have to tell beforehand about what you are not allowed to eat when eating out?	☐	☐	☐	☐	☐	☐	☐
9	have to check yourself whether you can eat something when eating out?	☐	☐	☐	☐	☐	☐	☐
10	hesitate eating certain foods when you don't know if it is safe?	☐	☐	☐	☐	☐	☐	☐
11	must watch out when touching certain foods?	☐	☐	☐	☐	☐	☐	☐
12	don't get anything when someone is giving treats at school?	☐	☐	☐	☐	☐	☐	☐

☺ not	☺ barely	☺ a little bit	☺ fairly	☹ quite	☹ very	☹ extremely

	**How *troublesome* is it, because of your food allergy, …**	☺	☺	☺	☺	☹	☹	☹
13	that the ingredients of a food change?	☐	☐	☐	☐	☐	☐	☐
14	that the label states: "May contain (traces of)…."?	☐	☐	☐	☐	☐	☐	☐
15	that you have to explain to people around you that you have a food allergy?	☐	☐	☐	☐	☐	☐	☐
16	that people around you forget that you have a food allergy?	☐	☐	☐	☐	☐	☐	☐
17	that others can eat the food you are allergic to when you do things with other people?	☐	☐	☐	☐	☐	☐	☐
18	that you don't know how things taste which you can't eat?	☐	☐	☐	☐	☐	☐	☐

	**How *frightened* are you because of your food allergy …**	☺	☺	☺	☺	☹	☹	☹
19	of an allergic reaction?	☐	☐	☐	☐	☐	☐	☐
20	of eating the wrong food by accident?	☐	☐	☐	☐	☐	☐	☐
21	to eat something you have never eaten before?	☐	☐	☐	☐	☐	☐	☐

	**Answer the following questions:**	☺	☺	☺	☺	☹	☹	☹
22	How *concerned* are you that you will never get rid of your food allergy?	☐	☐	☐	☐	☐	☐	☐
23	How *disappointed* are you when people don't take your food allergy into account?	☐	☐	☐	☐	☐	☐	☐
24	How *disappointed* do you feel because you have a food allergy?	☐	☐	☐	☐	☐	☐	☐

The following four questions are about the chance that you think you have of something happening to you because of your food allergy. Choose one of the answers. This is followed by two more questions about your food allergy. Answer every question by putting an 'x' in the box next to the proper answer.

0	1	2	3	4	5	6
never (0% chance)	very small chance	small chance	fair chance	big chance	very big chance	always (100% chance)

	How big do you think the chance is that you …	0	1	2	3	4	5	6
1	will accidentally eat something to which you are allergic?	☐	☐	☐	☐	☐	☐	☐
2	will have a severe reaction if you accidentally eat something to which you are allergic?	☐	☐	☐	☐	☐	☐	☐
3	will die if you accidentally eat something to which you are allergic?	☐	☐	☐	☐	☐	☐	☐
4	can <u>not</u> do the right things for your allergic reaction should you accidentally eat something to which you are allergic?	☐	☐	☐	☐	☐	☐	☐

**5. How many foods are you unable to eat because of your food allergy?**	**6.** Everyone does things with other people, such as;<br>- playing with friends,<br>- going to a birthday party,<br>- visiting,<br>- staying over with someone for a meal or eating out.<br>**How much does your food allergy affect things you do with others?**
☐ almost none	☐ so little I don't actually notice it
☐ very few	☐ very little
☐ a few	☐ little
☐ some	☐ moderately
☐ many	☐ a good deal
☐ very many	☐ a great deal
☐ almost all	☐ a very great deal

English – Parent version

FAQLQ-PF

Food Allergy Quality of Life Questionnaire – Parent Form (0-12 years)

To cite this questionnaire:
DunnGalvin A, Flokstra-de Blok BMJ, Burks AW, Dubois AEJ, Hourihane JO. Food allergy QoL questionnaire for children aged 0-12 years: content, construct, and cross-cultural validity. Clin Exp Allergy 2008 Jun;38(6):977-986.

Food Allergy Quality of Life Questionnaire-Parent Form (FAQoL-PF)

Children aged 0-12 years

Instructions to Parents

- The following are scenarios that parents have told us affect children's quality of life because of food allergy.
- Please indicate how much of an impact each scenario has on **your child's quality of life** by placing a tick or an x in one of the boxes numbered 0-6.

Response Options

0 = not at all
1= a little bit
2 = slightly
3 = moderately
4 = quite a bit
5 = very much
6 = extremely

All information given is completely confidential.
This questionnaire will only be identified by a code number.

- If your child is aged **0 to 3 years,** please answer **Section A.**
- If your child is aged **4 to 6 years,** please answer **Section A & Section B.**
- If your child is aged **7 years and over,** please answer **Section A, Section B & Section C.**

SECTION A

Not at all → Extremely

**Because of food allergy, my child feels……………**		0	1	2	3	4	5	6
1	Anxious about food	☐	☐	☐	☐	☐	☐	☐
2	Different from other children	☐	☐	☐	☐	☐	☐	☐
3	Frustrated by dietary restrictions	☐	☐	☐	☐	☐	☐	☐
4	Afraid to try unfamiliar foods	☐	☐	☐	☐	☐	☐	☐
5	Concerned that I am worried that he/she will have a reaction to food	☐	☐	☐	☐	☐	☐	☐

Not at all → Extremely

**Because of food allergy, my child……………**		0	1	2	3	4	5	6
6	Experiences physical distress	☐	☐	☐	☐	☐	☐	☐
7	Experiences emotional distress	☐	☐	☐	☐	☐	☐	☐
8	Has a lack of variety in his her diet	☐	☐	☐	☐	☐	☐	☐

Not at all → Extremely

**Because of food allergy, my child has been negatively affected by……….**		0	1	2	3	4	5	6
9	Receiving more attention more attention than other children of his/her age	☐	☐	☐	☐	☐	☐	☐
10	Having to grow up more quickly than other children of his/her age	☐	☐	☐	☐	☐	☐	☐
11	His/her environment being more restricted than other children of his/her age	☐	☐	☐	☐	☐	☐	☐

Not at all → Extremely

**Because of food allergy, my child's social environment is restricted because of limitations on……..**		0	1	2	3	4	5	6
12	Restaurants we can safely go to as a family	☐	☐	☐	☐	☐	☐	☐
13	Holiday destinations we can safely go to as a family	☐	☐	☐	☐	☐	☐	☐

Not at all → Extremely

**Because of food allergy, my child's ability to take part has been limited……..**		0	1	2	3	4	5	6
14	In social activities in other people's houses (*sleepovers, parties, playtime*)	☐	☐	☐	☐	☐	☐	☐

- If your child is aged **0 to 3 years,** please now go to **Section D.**
- If your child is aged **4 to 12 years,** please now answer **Section B.**

SECTION B

Not at all → Extremely

Because of food allergy, my child's ability to take part has been limited……..		0	1	2	3	4	5	6
15	In preschool/school events involving food (*class parties/treats/lunchtime*)	☐	☐	☐	☐	☐	☐	☐

Not at all → Extremely

Because of food allergy, my child feels……………		0	1	2	3	4	5	6
16	Anxious when going to new places	☐	☐	☐	☐	☐	☐	☐
17	Concerned that he/she must always be cautious about food	☐	☐	☐	☐	☐	☐	☐
18	'Left out' in activities involving food	☐	☐	☐	☐	☐	☐	☐
19	Upset that family social outings (*eating out, celebrations, days out*) have been limited by food allergy	☐	☐	☐	☐	☐	☐	☐
20	Anxious about accidentally eating an ingredient to which he/she is allergic	☐	☐	☐	☐	☐	☐	☐
21	Anxious when eating with unfamiliar adults/children	☐	☐	☐	☐	☐	☐	☐
22	Frustrated by social restrictions	☐	☐	☐	☐	☐	☐	☐

Not at all → Extremely

Because of food allergy, my child……………		0	1	2	3	4	5	6
23	Is more anxious in general than other children of his/her age	☐	☐	☐	☐	☐	☐	☐
24	Is more cautious in general than other children of his/her age	☐	☐	☐	☐	☐	☐	☐
25	Is not as confident as other children of his/her age in social situations	☐	☐	☐	☐	☐	☐	☐
26	Wishes his/her food allergy would go away	☐	☐	☐	☐	☐	☐	☐

- If your child is aged **6 years and under**, please now go to **Section D.**
- If your child is aged **7 years and older**, please answer **Section C.**

SECTION C

Not at all → Extremely

Because of food allergy, my child feels……………		0	1	2	3	4	5	6
27	Worried about his/her future(opportunities, relationships)	☐	☐	☐	☐	☐	☐	☐
28	That many people do not understand the serious nature of food allergy	☐	☐	☐	☐	☐	☐	☐
29	Concerned by poor labelling on food products	☐	☐	☐	☐	☐	☐	☐
30	That food allergy limits his/her life in general	☐	☐	☐	☐	☐	☐	☐

SECTION D.

Please answer the following questions with reference to the 6-point scale on the right

0 = extremely unlikely
1 = very unlikely
2 = somewhat unlikely
3 = likely
4 = quite likely
5 = very likely
6 = extremely likely

Q1. What chance **do you think** your child has of ….?

	Question	6-point Scale						
		0	1	2	3	4	5	6
1	……accidentally ingesting the food to which they are allergic ?							
2	……having a severe reaction if food is accidentally ingested ?							
3	……dying from his/her food allergy following ingestion in the future ?							
4	……effectively treating him/herself, or receiving effective treatment from others (including Epipen administration), if he/she accidentally ingests a food to which he/she is allergic ?							

Q2. What chance **does your child think** he/she has of ……?

	Question	6-point Scale						
		0	1	2	3	4	5	6
1	……accidentally ingesting the food to which they are allergic ?							
2	……having a severe reaction if food is accidentally ingested ?							
3	……dying from his/her food allergy following ingestion in the future ?							
4	……effectively treating him/herself, or receiving effective treatment from others (including Epipen administration), if he/she accidentally ingests a food to which he/she is allergic ?							

APPENDIX 8

SCORAD

SCORAD
EUROPEAN TASK FORCE ON ATOPIC DERMATITIS

Last Name | First Name

Date of Birth: DD/MM/YY

Date of Visit

INSTITUTION

PHYSICIAN

Topical Steroid used:
Potency(brand name)
Amount / Month (G)
Number of flares / Month

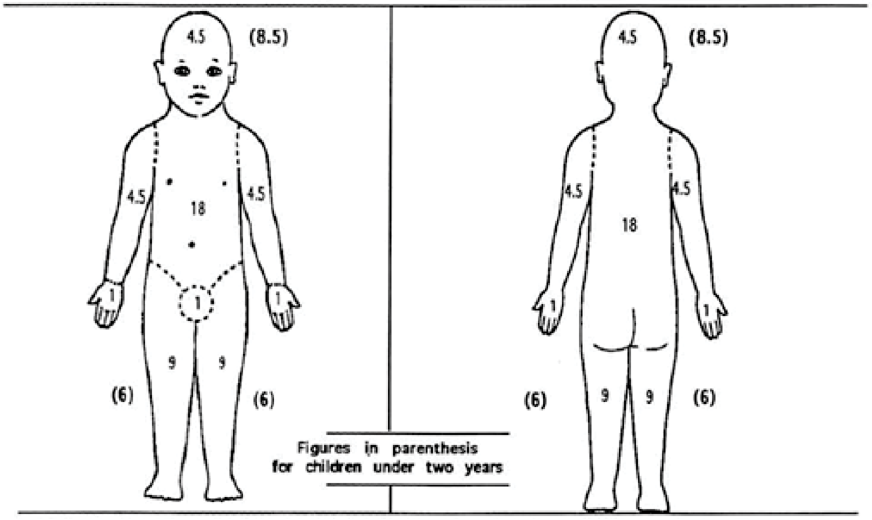

A: EXTENT Please indicate the area involved

B: INTENSITY

CRITERIA	INTENSITY
Erythema/darkening	
Edema/papulation	
Oozing/crust	
Excoriation	
Lichenification/prurigo	
Dryness*	

MEANS OF CALCULATION
INTENSITY ITEMS
(average representative area)
0= absence
1= mild
2= moderate
3= severe

* Dryness is evaluated on uninvolved areas

C: SUBJECTIVE SYMPTOMS PRURITUS+SLEEP LOSS

Objective SCORAD
A/5+7B/2 /83

SCORAD
A/5+7B/2+C /103

Visual analog scale (average for the last 3 days or nights)
PRURITUS (0 to 10)
SLEEP LOSS (0 to 10)
0 10

TREATMENT:

REMARKS:

10

Validation of SCORAD Index Dermatology 1997;195:10–19 17

Source: Kunz B, Oranje AP, Labrèze L, Stalder JF, Ring J, Taïeb A. Clinical validation and guidelines for the SCORAD index: consensus report of the European Task Force on Atopic Dermatitis. Dermatology. 1997;195(1):10-9.
