# Supplementary material for: Efficacy and safety of peanut epicutaneous immunotherapy in patients with atopic comorbidities
Source: J Allergy Clin Immunol Glob. 2022 Sep 22;2(1):69–75. doi: 10.1016/j.jacig.2022.07.009 (PMC10509968; doi:10.1016/j.jacig.2022.07.009)

PAREXEL International

DBV TECHNOLOGIES S.A.

PEPITES

A Double-blind, Placebo-controlled, Randomized Phase III Pivotal Trial to Assess the Efficacy and Safety of Peanut Epicutaneous Immunotherapy with Viaskin® Peanut in Peanut-allergic children (PEPITES Study)

Statistical Analysis Plan V4.0 – 04 October, 2017

PAREXEL Project Number: 223218

SPONSOR SIGNATURE PAGE

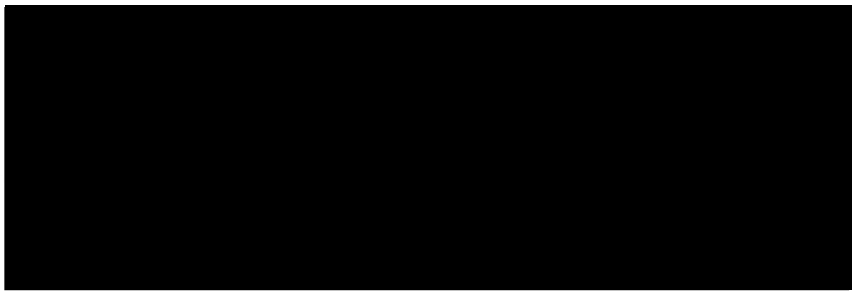

PAREXEL SIGNATURE PAGE

Signatures below confirm that the review process has been completed in accordance with SOP-GDO-WW-019.

This document has been approved and signed electronically on the final page by the following:

	**Signatory**
**Author**	Patrice BACQUET<br>Project Role: Biostatistics Lead
	**Signatory**
**Reviewer**	Vincent HOUE<br>Project Role: Biostatistics Reviewer
	Antje SIMON<br>Project Role: Primary Statistical Programmer
	Beate GERSTBREIN<br>Project Role: MWS Reviewer
	Claire VANEENWYK<br>Project Role: GMS Reviewer

TABLE OF CONTENTS

1 INTRODUCTION 10
2 STUDY OBJECTIVES 10
3 INVESTIGATIONAL PLAN 10
3.1 Overall Study Design and Plan 10
3.2 Efficacy and Safety Endpoints 17
4 STATISTICAL METHODS 19
4.1 Data Quality Assurance 19
4.2 General Considerations 20
4.3 Study Subjects 22
4.3.1 Disposition of Subjects 22
4.3.2 Protocol Deviations 23
4.4 Analysis Populations 24
4.4.1 Screened Population 24
4.4.2 Intent-to-treat Population 24
4.4.3 Full analysis set 24
4.4.4 Per-protocol Population 24
4.4.5 Safety Population 25
4.5 Demographics and Baseline characteristics (including medical history, parental atopic history and disease history) 25
4.5.1 Demographic variables 25
4.5.2 Baseline Characteristics 26
4.5.3 Medical history 27
4.5.4 Parental atopic medical history 27
4.5.5 Disease history 28
4.6 Previous and concomitant medications 28
4.7 Study duration, treatment exposure and compliance 30
4.8 Efficacy Evaluation 32
4.8.1 Analysis and Data Conventions 32
4.8.1.1 Multi-center Studies 32
4.8.1.2 Handling of Dropouts or Missing Data 32
4.8.1.3 Multiple Comparisons/Multiplicity 33
4.8.1.4 Interim Analyses 35
4.8.1.5 Examination of Subgroups 35
4.8.2 Primary Efficacy Analysis 36
4.8.3 Secondary Efficacy Analyses 38
4.8.3.1 Percentage of treatment responders in each screening ED subgroup 38
4.8.3.2 Percentage of treatment responders in each age group (4-5 and 6-11 years of age) 39
4.8.3.3 Cumulative reactive dose of peanut protein and change from Baseline at Month 12 40
4.8.3.4 Eliciting dose of peanut protein and change from Baseline at Month 12 42

4.8.3.5 Percentage of subjects *responsive* (those showing objective symptoms leading to DBPCFC stop) to a cumulative dose ≥1,444 mg peanut protein at the Month 12 DBPCFC 43
4.8.3.6 Percentage of subjects *unresponsive* (those showing no objective symptoms leading to DBPCFC stop) to a cumulative dose ≥1,444 mg peanut protein at the Month 12 DBPCFC 44
4.8.3.7 Percentage of subjects *unresponsive* (those showing no objective symptoms leading to DBPCFC stop) to the highest dose of peanut protein which is the percentage of subjects who pass the Month 12 DBPCFC .. 44
4.8.4 Other Efficacy Analyses 44
4.8.4.1 Peanut-specific IgE and IgG4 over time 44
4.8.4.2 Skin prick test mean wheal diameters over time and at Month 12 .. 45
4.8.4.3 Descriptive analysis of the quality of life questionnaires (Food Allergy Quality of Life Questionnaire [FAQLQ]/Food Allergy Independent Measure [FAIM]) and change from Baseline at Month 12.46
4.9 Study Drug Safety Evaluation 48
4.9.1 Adverse Events 49
4.9.2 Deaths 53
4.9.3 Local skin reactions as collected in the diary 53
4.9.4 Local skin reactions as graded by the investigator at each visit 54
4.9.5 Adverse event of special interest (AESI) 54
4.9.6 Clinical Laboratory Evaluation 55
4.9.7 Vital Signs 56
4.9.8 Physical Examination 57
4.9.9 Spirometry and Peak Expiratory Flow Results 58
4.9.10 Symptomatic Reactions during the double-blind placebo controlled food challenge (DBPCFC) 58
4.9.11 Data and Safety Monitoring Board 59
4.10 Exploratory Analyses 60
4.10.1 Treatment responders at Month 12 according to demographic and medical history subgroups 60
4.10.2 Analysis over time of IgE and IgG4 specific to peanut components 60
4.10.3 Enumeration and characterization of reactions triggered by accidental consumption of peanut during the study and analysis of Risk taking behavior of subjects (voluntary consumption of peanut) 61
4.10.4 Epigenetic modifications of the promoters of specific genes 61
4.10.5 Safety sub-analysis in subjects with mutations in the filaggrin gene versus wild type subjects 62
4.10.6 Sensitization status to other allergens and their evolution over the study period 62
4.10.7 SCORAD evolution over time 62
4.11 Patch adhesion 63
4.12 Determination of Sample Size 65
4.13 Changes in the Conduct of the Study or Analysis planned in protocol 66
5 REFERENCES 69
6 APPENDICES 70

6.1 Appendix 1: Major Protocol deviations 70
6.2 Appendix 2: SAS® Code lines 71
Appendix 2.1: Statistical analysis methods and procedures used for the analysis of the primary efficacy endpoint. 71
Appendix 2.2: Strategy of analysis of the primary efficacy endpoint using multiple imputation of missing data 73
Appendix 2.3: Statistical analysis methods and procedures used for the analysis of the cumulative reactive dose of peanut protein at month 12. 76
Appendix 2.4: Statistical analysis methods and procedures used for the analysis over time of peanut-specific IgE and IgG4. 77
6.3 Appendix 3: Common Terminology Criteria for Adverse Events (CTCAE) grades 78
6.4 Appendix 4: List of Post-Text Tables, figures, listings, and Supportive SAS output appendices 78
6.5 Appendix 5: Methodology of identification of systemic allergic adverse events 79

LIST OF ABBREVIATIONS

AE / AESI	Adverse event / Adverse event of special interest
ANCOVA	Analysis of covariance
ATC	Anatomical Therapeutic Chemical (Classification System)
BOCF	Baseline Observation Carried Forward
BSA	Body Surface Area
CI	Confidence interval
CTCAE	Common Terminology Criteria for Adverse Events
CTMS	Clinical trials Management System
D	Day
DBPCFC	Double-blind, placebo-controlled food challenge
DSMB	Data and Safety Monitoring Board
eCRF	Electronic case report form
ED	Eliciting Dose
EPIT	EPicutaneous ImmunoTherapy
ET	Early Termination
FAQLQ	Food Allergy Quality of Life Questionnaire
FAIM	Food Allergy Independent Measure
FAS	Full analysis set
FEV_1	Forced expiratory volume in one second
ICF	Informed consent form
IgE, IgG, IgG4	Immunoglobulin E, immunoglobulin G, immunoglobulin G4 subtype
IQR	Interquartile range
IP	Investigational product
ITT	Intent-to-treat
IWRS	Interactive Web Response System
M	Month
mBOCF	Modified BOCF
MedDRA	Medical Dictionary for Regulatory Activities
PC	Phone Contact
PEF	Peak Expiratory Flow

PP	Per-protocol
PT	Preferred term
PDV	Protocol deviation
Q1, Q3	First, third quartile
SAE	Serious adverse event
SAP	Statistical analysis plan
SCORAD	Scoring atopic dermatitis
SE	Standard Error
SOC	System organ class
SPT	Skin prick test
TEAE	Treatment-emergent adverse event
TFL	Tables, Figures and listings
US(A)	United States (of America)
UV	Unscheduled visit
V	Visit
WHO	World Health Organization

Figures and tables

Figure 1: Study Design 12

Table 1: Schedule of Procedures 13
Table 2: Pre-defined Hierarchical Order for Analysis of Efficacy Endpoints 34
Table 3: Criteria to Determine Clinically Relevant Abnormalities in Vital Signs 57

1 INTRODUCTION

This Statistical Analysis Plan (SAP) describes the statistical methods to be used for the reporting and analyses of data collected under the DBV Technologies Phase III protocol PEPITES[1].

This SAP is based upon the following study documents:

- Study Protocol, Version Final (July 20, 2015),
- Amended protocol, Version 2.0 (December 9, 2015)
- Electronic Case Report Form (eCRF), Version 4.0 (June 23, 2016)
- Q^2 Central Lab Data Transfer Agreement, Version 4.0 (January 09, 2017)
- SDTM annotated CRF, Version 1.3 (October 07, 2016)
- SDTM Data Transfer Mapping Specifications, Version 1.3 (January 17, 2017)

2 STUDY OBJECTIVES

The objective of this study is to assess the efficacy and the safety of Viaskin® Peanut to induce desensitization to peanut in peanut-allergic subjects 4 through 11 years of age after a 12-month treatment period by EPIT (Epicutaneous immunotherapy).

3 INVESTIGATIONAL PLAN

3.1 Overall Study Design and Plan

This is a 12-month, Phase III, double-blind, placebo-controlled, randomized study to assess the efficacy and safety of Viaskin® Peanut, dosed at 250 µg peanut protein (per patch) in peanut-allergic children from 4 through 11 years of age.
The overall maximum study duration for each subject is approximately 61 weeks (6-week screening period, 12-month treatment period and 2-week follow-up period).
During the maximum 6-week screening period, subjects will undergo a first screening visit and an entry double-blind, placebo-controlled food challenge (DBPCFC) to peanut to confirm their allergy and their entry peanut eliciting dose (ED). The starting dose of the challenge will be 1 mg peanut protein and will escalate up to a highest dose of 300 mg peanut protein. Subjects who react at or below the dose of 300 mg peanut protein are considered eligible. Randomization of eligible subjects will occur in a 2:1 ratio to Viaskin® Peanut dosed at 250 µg peanut protein (active treatment) or placebo. Subjects will be stratified at randomization by their entry/screening DBPCFC ED in one of the following two strata:

[1] PEPITES: A Double-blind, Placebo-controlled, Randomized Phase III Pivotal Trial to Assess the Efficacy and Safety of Peanut Epicutaneous Immunotherapy with Viaskin® Peanut in Peanut-allergic Children

- Stratum 1: children with a screening ED of 1 mg, 3 mg or 10 mg;
- Stratum 2: children with a screening ED of 30 mg, 100 mg or 300 mg.

The randomization scheme will ensure that the ratio of active treatment to placebo is maintained in each stratum.
Subjects randomized in the study and consenting for the genetic analysis on a voluntary basis will be assessed for mutations in the filaggrin gene. Subjects' participation for this genetic analysis will be optional.
Subjects will apply a Viaskin® patch containing either peanut protein or placebo daily for a period of 12 months. At Month 12, a second DBPCFC to peanut will be performed, with a starting dose of 1 mg peanut protein with escalation up to a highest dose of 2,000 mg peanut protein. This evaluation will help determine the primary efficacy endpoint of this pivotal study.

Subjects will undergo other efficacy parameter assessments at Months 3, 6 and 12, including immunological changes in peanut-specific immunoglobulin E (IgE), peanut-specific immunoglobulin G4 (IgG4) and skin prick tests (SPTs).
Key assessments of global safety will be performed at each study visit by the Investigators, including skin observation of the patch areas of application (inter-scapular area of the back), spirometry, peak expiratory flow (PEF) measurements, vital signs, physical examinations and clinical laboratory assessments. Atopic dermatitis will also be assessed at Baseline and at Months 3, 6 and 12 using the SCORAD (Scoring atopic dermatitis), for which specific training will be provided for better use and accurate assessment.

In between visits, the severity of local skin reactions will be assessed on a daily basis by the subjects (parents/guardians) in their diary for 6 months. Any other adverse events (AEs), local skin reactions occurring after the first 6 months of treatment and any concomitant medications will also be reported in the diary by the subjects and this will be reviewed by the site medical staff at each patient visit. The 3 pre-specified solicited symptoms graded daily by the subjects in their diaries, itching, redness and swelling at the patch skin location, will not be reported by the Investigators in the adverse events form of the e-CRF, except if these symptoms are part of another concomitant disease or if they result in the subject's study discontinuation or consider as a serious adverse event.

In addition, the adhesion of the patch to the skin, especially the maintenance of the occlusion of the patch, will be assessed by the subjects' parents/guardians for 28 days of treatment (whenever possible, these should be consecutive days). This assessment will be conducted during a period between Month 3 and Month 9. On the same days, photographs of the patches attached to the skin will be made by the subjects' parents/guardians as instructed. The review of these photos will allow the Investigators to control and ensure that the parents' assessments of the patch adhesion were satisfactory. The trained site staff will also assess the patch adhesion of all subjects at each subject visit (without removing the patch).

After completion of the PEPITES study, all eligible subjects, including the placebo subjects, will be offered the opportunity to participate in an open-label extension study (PEOPLE[2]) to receive Viaskin® Peanut 250 µg for 24 additional months of treatment. Subjects who will decide to roll-over into the extension study will have their last PEPITES study visit at Visit 11 and they will start the extension study at that visit. Subjects who decide not to roll-over into the extension study will continue their visit schedule up to Visit 12.
The study will be conducted at approximately 28 to 40 centers in 4 to 7 countries in Australia, Europe and North America with Investigators and staff who are trained and experienced in the diagnosis and management of peanut allergy and anaphylaxis, and equipped and capable of performing a DBPCFC in children.

The study design is presented below in Figure 1.

Figure 1: Study Design

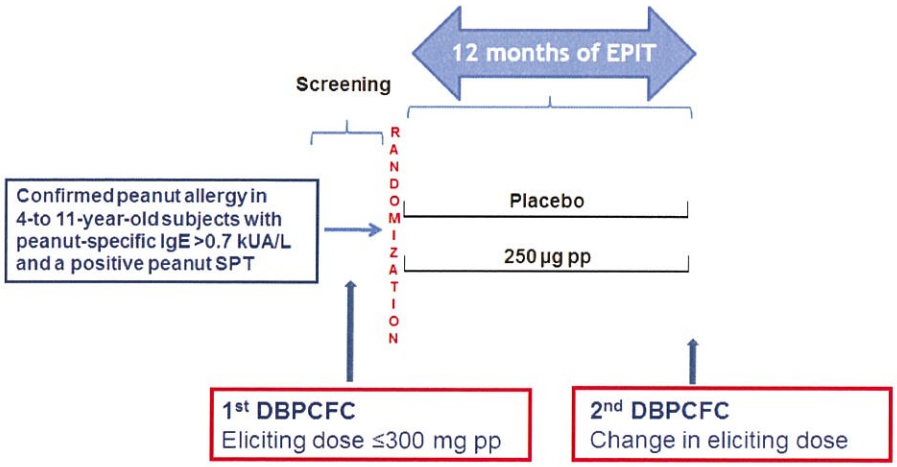

Abbreviations: DBPCFC = Double-blind, placebo-controlled food challenge; EPIT = Epicutaneous immunotherapy; IgE = Immunoglobulin E; pp = peanut protein; SPT = Skin prick test.

The schedule of procedures is presented in table 1.

[2] Open-label Follow-up Study of the PEPITES Study to Evaluate the Long-term Efficacy and Safety of Viaskin® Peanut

Table 1: Schedule of Procedures

Study Assessments	Screening			Treatment Period												End of Study		Early Termination	Unscheduled Visit
	V1	V2	V3[1]	V4[1]	PC	V5	PC	V6	PC	V7	PC	V8	PC	V9	V10	V11	V12	ET	UV[2]
	D-42			D1	D4	D8	D22	M1	M2	M3	M4.5	M6	M7.5	M9	M12				
Informed consent	X																		
Eligibility (inclusion/exclusion criteria)	X[3]			X															
Medical history[4]	X																		
Parental medical history of atopy	X																		
Demographics	X																		
Physical examination[5]	X	X[6]	X[6]	X		X		X		X		X		X	X[6]	X[6]	X	X	X
Vital signs[7]	X	X[6]	X[6]	X		X		X		X		X		X	X[6]	X[6]	X	X	X
Spirometry (FEV_1)[8]	X									X		X		X	X			X	X
PEF	X	X[6]	X[6]	X		X		X		X		X		X	X[6]	X[6]		X	X
SCORAD	X									X		X			X				
Skin Prick Test	X									X		X			X			X	
Immunological markers[9]	X									X		X			X			X	
Laboratory tests[10]	X									X		X			X			X	X
FAQLQ/FAIM[11]	X														X				
Filaggrin gene: informed consent - blood sampling										X[12]		X[12]			X[12]				
Pregnancy test	X														X			X	
Epigenetic analyses	X									X		X			X				
DBPCFC		X	X												X	X			
**Randomization**				X															
Adverse events	X	X	X	X	X	X	X	X	X	X	X	X	X	X	X	X	X	X	X
Concomitant medications	X	X	X	X	X	X	X	X	X	X	X	X	X	X	X	X	X	X	X

Study Assessments	Screening			Treatment Period												End of Study		Early Termination	Unscheduled Visit
	V1	V2	V3[1]	V4[1]	PC	V5	PC	V6	PC	V7	PC	V8	PC	V9	V10	V11	V12	ET	UV[2]
	D-42			D1	D4	D8	D22	M1	M2	M3	M4.5	M6	M7.5	M9	M12				
Check for accidental peanut consumption					X	X	X	X	X	X	X	X	X	X	X	X	X	X	X
Subject diary (dispense/check)				X	X	X	X	X		X		X		X	X	X	X	X	X
Dispense subject safety leaflet and subject identification card				X															
Study drug dispensation				X		X		X		X		X		X	X[15]				
Apply 1 Viaskin® patch at site				X															
Check the used/unused study drug dispensed						X		X		X		X		X	X	X		X	X
Check skin reaction under the patch and grade it[13]				X		X		X		X		X		X	X	X		X	X
Photographs of the back where patches were applied				X		X		X		X		X		X	X	X		X	X
Patch adhesion:<br>- subject's assessment										X[14]	X[14]	X[14]	X[14]	X[14]					
Patch adhesion:<br>- Investigator's assessment				X		X		X		X		X		X	X	X			
Dispense epinephrine auto-injector and anaphylaxis emergency action plan				X															
Review utilization of epinephrine auto-injector						X		X		X		X		X					

Study Assessments	Screening			Treatment Period												End of Study		Early Termination	Unscheduled Visit
	V1	V2	V3[1]	V4[1]	PC	V5	PC	V6	PC	V7	PC	V8	PC	V9	V10	V11	V12	ET	UV[2]
	D-42			D1	D4	D8	D22	M1	M2	M3	M4.5	M6	M7.5	M9	M12				
and anaphylaxis emergency action plan (when required)																			
Hours under observation before discharge		3	3	3											3	3			

Abbreviations: D = Day; DBPCFC = Double-blind, placebo-controlled food challenge; ET = Early termination; FAQLQ/FAIM = Food Allergy Quality of Life Questionnaire/Food Allergy Independent Measure; FEV_1 = Forced expiratory volume in one second; M = Month; PC = Phone contact; PEF = Peak expiratory flow; SCORAD = Scoring atopic dermatitis; SPT = Skin prick test; UV = Unscheduled Visit; V = Visit.

1 Visit 3 within 2 weeks of V2 and through D-1. Visit 4 may take place on the same day as Visit 3, but this would result in a very long day for the subject (parents/guardians) as all Visit 4 procedures would then have to take place that same day.

2 Unscheduled Visit in case of AEs, need for treatment re-supply, etc. Procedures will be performed as deemed necessary by the investigator.

3 Except for those that depend on the results of the immunological markers testing (peanut-specific IgE) and on the outcome of the entry/screening DBPCFC.

4 Including history of peanut allergy.

5 Including a complete skin examination, body weight and height.

6 These examinations are to be done before the DBPCFC. Additionally, they can be repeated during the DBPCFC procedure on both days anytime if judged necessary by the Investigator.

7 Blood pressure, heart rate and respiratory rate.

8 FEV1 will be measured in subjects 6 years of age and older.

9 Peanut-specific IgE, peanut-specific IgG4, peanut specific IgE and peanut specific IgG4 to Ara h 1, Ara h 2, Ara h 3, Ara h 8 and Ara h 9. IgE specific to cow's milk, egg white, house dust mites, and grass pollen will be collected at Visit 1 and Visit 10 only and in case of an early termination visit.

10 Laboratory tests performed centrally. Hematology: hemoglobin, hematocrit, platelets, red blood cells, white blood cells with differential cell count. Biochemistry: aspartate aminotransferase, alanine aminotransferase, total bilirubin, total protein, blood urea nitrogen, creatinine.

11 For both FAQLQ and FAIM, subjects ≥8 years of age will use the Child Form of the FAQLQ/FAIM. All parents/guardians will use the Parental Form for children. The specific forms of FAQLQ and FAIM will be completed in the countries where they are available in local languages.

12 Signing the consent for the filaggrin genetic analysis can be done any time after the subject is effectively randomized in the study. However, collection of blood is recommended only once at any of the following visits: Visit 7, 8 or 10.

13 Check the reaction of the skin on the back of the subject and grade the severity of the local skin reactions. At Visit 4, grading is to be done before patch

application and 30 min, 1 h, 2 h and 3 h after patch application.

14 The assessment of the patch adhesion to the skin will consist of a 28-day specific analysis to occur between Month 3 (Visit 7) and Month 6 (Visit 8). This assessment period can be extended up to Month 9 (Visit 9) in case it has not been completed or fully completed between Month 3 and Month 6.

15 The same treatment box dispensed at visit 9 will be re-dispensed at visit 10 (until visit 11) after the compliance has been assessed

Time windows:

Visit	Time windows
V1	D-42 max before V4
V2	Anytime through D-2
V3	Within 1 weeks of V2 and through D-1
PC-D4, PC-D22	+/- 2 days
V5 (D8) , V6 (M1), PC-M2	+/- 3 days
V7 to V10 (M3,6 ,9,12 ,PC M4.5, M7.5)	+/- 7 days
V11	Maximum 1 week after V10
V12	V11 + 2 weeks

3.2 Efficacy and Safety Endpoints

The DBPCFC to peanut will be performed at study entry and at Month 12 in order to assess the primary efficacy endpoint and the secondary efficacy endpoints.

Primary efficacy endpoint:

The primary efficacy endpoint is the difference between percentage of treatment responders in the active Viaskin® Peanut 250 µg group compared to the placebo group after 12 months of EPIT treatment in the overall population. A subject is defined as a treatment responder if:

- The initial ED was >10 mg peanut protein and the ED is ≥1,000 mg peanut protein at the Month 12 DBPCFC, or;
- The initial ED was ≤10 mg and the ED is ≥300 mg peanut protein at the Month 12 DBPCFC.

To ensure the validity of this endpoint, the actual screening ED value entered in the eCRF will be used to define the initial ED. This will account for any randomization stratum error. Additionally, actual screening ED value entered in the eCRF will be used to define screening ED subgroups (screening ED≤10 mg / screening ED>10 mg). Alternatively, the screening ED value used for the randomization (that may include randomization stratum errors), is designated hereunder as "Screening ED stratum".

Secondary efficacy endpoints:

The following secondary efficacy endpoints will be analysed.

- Percentage of treatment responders at Month 12 in the active Viaskin® Peanut 250 µg group compared to the placebo group in each of the 2 screening ED subgroups.
- Percentage of treatment responders at Month 12 in the active Viaskin® Peanut 250 µg group compared to the placebo group in each of the 2 age subgroups: 4 to 5 years-old; 6 to 11 years-old.
- Mean and median cumulative reactive dose of peanut protein and change from Baseline at Month 12 in the active Viaskin® Peanut 250 µg group versus the placebo group in the overall population, and for each screening ED subgroup.
- Mean and median ED of peanut protein and change from Baseline at Month 12 in the active Viaskin® Peanut 250 µg group versus the placebo group in the overall population, and for each screening ED subgroup.
- Percentage of subjects responsive (those showing objective symptoms leading to DBPCFC stop) to a cumulative dose ≥1,444 mg peanut protein at the Month 12 DBPCFC in the active Viaskin® Peanut 250 µg group versus the placebo group in the overall population, and for each screening ED subgroup.
- Percentage of subjects unresponsive (those showing no objective symptoms leading to DBPCFC stop) to a cumulative dose ≥1,444 mg peanut protein at the

Month 12 DBPCFC in the active Viaskin® Peanut 250 µg group versus the placebo group in the overall population, and for each screening ED subgroup.

- Percentage of subjects unresponsive (those showing no objective symptoms leading to DBPCFC stop) to the highest dose of peanut protein, which is the percentage of subjects who pass the Month 12 DBPCFC in the active Viaskin® Peanut 250 µg group versus the placebo group in the overall population, and for each screening ED subgroup.

Other efficacy endpoints:

The following variables will also be analysed:

- Change from baseline in peanut-specific IgE and IgG4 at months 3, 6 and 12 in the active Viaskin® Peanut 250 µg group versus the placebo group in the overall population, and for each screening ED subgroup;
- Change from baseline in peanut skin prick testing average wheal diameters at months 3, 6 and 12 in the active Viaskin® Peanut 250 µg group versus the placebo group in the overall population, and for each screening ED subgroup;
- Quality of life scores (Food Allergy Quality of Life Questionnaire [FAQLQ]/Food Allergy Independent Measure [FAIM]) at Baseline and Month 12 and change from baseline in FAQLQ score at Month 12 in the overall population.

Criteria for Evaluation of Study Drug Safety

The following safety criteria will be evaluated:

- AEs and treatment-emergent adverse events (TEAEs) by System Organ Class (SOC) and Preferred Terms (PTs);
- TEAEs by maximum severity and relatedness to Viaskin® Peanut 250 µg;
- Incidence, duration and maximum severity of local cutaneous Viaskin® Peanut 250 µg-induced AEs as assessed by the subject;
- Severity of local cutaneous Viaskin® Peanut 250 µg-induced AEs as assessed by the Investigator;
- Adverse events of special interest (AESI) including grade 4 local cutaneous reactions and systemic allergic AEs considered related to Viaskin® Peanut 250 µg;
- Serious adverse events (SAEs) by SOC and PTs, and relatedness to Viaskin® Peanut 250 µg (SAEs due to DBPCFC will be presented separately);
- Laboratory data, physical examinations and vital signs;
- Spirometry results and PEF results.

The above criteria will be studied overall and by treatment group, in the Safety population, as well as for the age ranges 4 to 5 years, 6 to 8 years and 9 to 11 years, and for each screening ED subgroup.

Criteria for Evaluation of Study Procedure Safety

- Objective symptoms elicited during the entry/screening DBPCFC and Month 12 DBPCFC by severity;
- Change in severity of objective symptoms elicited during the DBPCFC from Baseline to Month 12 in the active Viaskin® Peanut 250 µg group versus the placebo group;
- SAEs elicited during the entry/screening DBPCFC and Month 12 DBPCFC.

The above criteria will be studied overall and by treatment group, in the Safety population, as well as for the age ranges 4 to 5 years, 6 to 8 years and 9 to 11 years, and for each of the screening ED subgroup.

Exploratory criteria

- Change from Baseline in IgE and IgG4 specific to peanut protein components at Month 3, 6 and 12 in the active Viaskin® Peanut 250 µg group versus the placebo group;
- Enumeration and characterization of reactions triggered by accidental/voluntary consumption of peanut during the study and analysis of risk-taking behaviour;
- Epigenetic modifications of the promoters of specific genes (but this will not be available at the time of database lock);
- Safety sub-analysis in subjects with mutations in the filaggrin gene versus wild type subjects;
- Sensitization status to some other allergies and their evolution over the study period;
- SCORAD evolution over time.

4 STATISTICAL METHODS

4.1 Data Quality Assurance

All tables, figures and data listings to be included in the report will be independently checked for consistency and integrity in accordance with standard PAREXEL procedures as follows:

PAREXEL Biostatistics and Statistical Programming seeks to ensure the quality of the results provided for the study in the form of Tables, Figures and Listings (TFL), and the derived datasets used in their creation, through the following processes:

- Derived datasets will be independently reprogrammed by a second programmer. The separate datasets produced by the two programmers must match 100%.
- All Tables will be independently reprogrammed by a second programmer for numeric results and must match 100%.
- Statisticians will be involved in the process of programming and validating tables that include inferential statistical results.

- Figures will be checked for consistency against corresponding tables and listings, or independently reprogrammed if there are no corresponding tables or listings.
- Listings will be checked for consistency against corresponding tables, figures, and derived datasets.
- The entire set of TFL will be checked for completeness and consistency prior to its delivery to the Sponsor by the Lead Statistical Programmer, the Lead Biostatistician, and a senior level statistician, or above, who is not a member of the project team.
- The validation process will be repeated any time TFL are redelivered using different data. Execution of this validation process will be documented through the study "Program Status Tracker" (created to track all programs for analysis variables, tables, listings and figures) that will be provided to the Sponsor at study conclusion.

4.2 General Considerations

The following conventions will be used when presenting summary statistics and analyzing continuous study data:

- Continuous data will be summarized in terms of number of subjects or observations with non-missing data (n), mean, standard deviation (SD), first quartile (Q1), median, third quartile (Q3), minimum (min) and maximum (max), unless otherwise stated.
- For data not normally distributed, a $\log_{10}$ transformation will be implemented before analysis.
- Compared to the number of decimals recorded for the raw data in the database, the statistics will be reported with the following number of decimals:
  - Minimum and maximum: same number
  - Mean, median, Q1, Q3: one extra decimal
  - Standard deviation: two extra decimals.
- For any parameter at a specific visit:
  - Change from Baseline will be calculated as the value of that parameter at that visit minus the Baseline value of that parameter
  - Relative change from Baseline will be calculated as the value of that parameter at that visit minus the Baseline value of that parameter divided by the Baseline value of that parameter
- For laboratory parameters, values below a detection limit or above a specific value (e.g. <0.35 kU/L or >100 kU/L) will be considered as equal to that limit/value (e.g. =0.35 or 100) in the statistical analyses (except in listing where the reported value will be presented).
  In case of change of detection limit during the course of the study (e.g. from <0.35 kU/L to <0.1 kU/L), all values lower than the highest limit value will be imputed to this threshold (e.g. all values lower than 0.35 kU/L will be considered as equal to 0.35 kU/L). This conservative approach will ensure that changes from Baseline are consistent.

The following conventions will be used when presenting summary statistics for categorical study data:

- Categorical data will be summarized in terms of the number of subjects or observations providing non-missing data at the relevant time point (n), frequency counts and percentages. Any planned collapsing of categories will be detailed in the SAP text and the data displays.
- Percentages will be presented to one decimal place. Percentages will not be presented for zero counts. Percentages will be calculated using n as the denominator. If sample sizes are small, the data displays will show the percentages, but any textual report will describe frequencies only.
- Change from Baseline will be summarized using shift tables where appropriate.

The following conventions will be considered regarding study assessments and time points:

- Date and time of the first patch application will be reported in the eCRF. The baseline value is the last measurement before date and time of first patch application, in other words, Visit 4 data are taken into account for the Baseline, unless the date/time clearly indicates that the value is after the patch application. For physical exam, even if the time is not reported, V4 will be taken into account as Baseline.
- The 'end of study' assessment is defined as the last available post-treatment assessment.
- "Treatment Day" will be calculated relative to date of first patch application (Treatment day 1 = Date of first patch).
- Assessments collected at unscheduled visits will not be included in summary tables but only listed.
- Assessments outside of protocol allowable windows will be taken into account in the analysis according to the visit in which the data are entered.
- Early termination assessments will be analyzed as having occurred at the next scheduled assessment.

The following conventions will be considered regarding confidence p-values and confidence intervals (CI):

- P-values greater than or equal to 0.001, in general, will be presented to three decimal places. P-values less than 0.001 will be presented as "<0.001".
- Confidence intervals will be presented to one more decimal place than the raw data.

The following conventions will be applied on diary data dates, for each incomplete patch application or removal date:

- Incomplete application date:
    - During the first 14 days of patch application: imputed as removal dates (if removal date is complete).
    - Beyond the first 14 days: imputed as removal date -1 (if removal date is complete).

- Incomplete removal date:
  - During the first 14 days of patch application: imputed as application date (if application date is complete).
  - Beyond the first 14 days: imputed as application date +1 (if application date is complete).

No imputations will be performed on time of application/removal unless explicitly described for specific analyses.

Titles for tables and listings produced will specify the type of data reported as well as the population concerned (in parentheses).
For table and/ or listings duplicated for each of the screening ED subgroup, the identification of subgroup will be mentioned left justified below the header in the core of the table as:

- Screening ED subgroup 1: Children with a screening ED of 1 mg, 3 mg or 10 mg;

Or

- Screening ED subgroup 2: Children with a screening ED of 30 mg, 100 mg or 300 mg.

Listings will generally include the following identification variables: subject identifier, sex and age, screening ED subgroup, treatment group.
Unless specified, complete dates will be reported in listings using a DDMMMYYYY format (i.e.: 01JAN2015) and time using a 24h clock-time (i.e.: 20:35).
Partial dates will be reported as MMMYYYY or YYYY.
All report outputs will be produced using SAS® version 9.3 or a later version in a secure and validated environment. Each report output will be provided to the Sponsor in a Microsoft Word document (one document per output). All report outputs will be also pooled in one single Microsoft Word document.

4.3 Study Subjects

4.3.1 Disposition of Subjects

A clear accounting of the disposition of all subjects who enter the study will be provided, from screening to study completion.
Subject disposition will be summarized for all subjects, overall and by screening ED subgroup.
Reason for non-randomization will be listed for screened subjects who failed to be randomized.
The number of screened subjects will be provided.
The number and percentage of randomized subjects, who completed the study and those who discontinued the study together with the primary reason for discontinuation from the study will be tabulated overall and by treatment group.

All the above percentages will be expressed based on the number of subjects in the ITT population.
The number and percentage of subjects randomized in total, by region (Australia / Europe / North America), by country and by site will be summarized overall and for each treatment group.
Additionally, an enrollment summary will be presented overall, by site, country and region showing the number of subjects screened, randomized and completing the study and among randomized subjects: the first date of consent, the last study visit date, the study duration (in days - calculated as last study visit exit date – first date of consent for first screened subject +1).
Study disposition and termination details will be listed for each subject. Listings will also be created to show the study analysis set classifications and randomization assignments.

4.3.2 Protocol Deviations

Deviations from the protocol including violations of inclusion/exclusion criteria will be assessed as "minor" or "major" in cooperation with the Sponsor prior to unblinding.
Major protocol deviations (PDV) are defined as those deviations from the protocol likely to have an impact on the perceived efficacy and/or safety of the study treatment. The impact of major PDV on the efficacy and/or safety results will be investigated by assessing the robustness of the study results and conclusions to the choice of analysis population (see Section 4.4), both including and excluding data potentially affected by major PDV.
Observable PDV data will be entered into PAREXEL'S Clinical Trials Management System (CTMS). Some PDV will be identified via programmed edit checks and listings.
The study team and the Sponsor will conduct on-going reviews of the PDV data from the CTMS and the PDV listings and the resulting set of evaluable subjects throughout the study, adjusting the PDV criteria (major/minor PDV) as appropriate.
The evaluable subject sets must be finalized prior to database lock.
PDV will help to define the subjects to be excluded from the PP population.
The two following violation criteria (as Mentioned in Appendix 1: Major Protocol deviations) have been pre-defined to be excluded subjects from the PP analysis:

- Subjects who do not perform the DBPCFC at 12 months of EPIT treatment,
- Subjects with global treatment compliance below 80%.

Listings will be created to aid the review of data and identification of possible PDV.

The number and percentage of subjects with major PDV will be summarized by type of deviation, overall and by treatment group for all randomized subjects. All PDV data will be listed.

Major PDV and any action to be taken regarding the exclusion of subjects or affected data from specific analyses are specified in Appendix 1: Major Protocol deviations.

4.4 Analysis Populations

After database lock, analysis population outputs will be produced and will be sent to DBV Technologies for review. These outputs will first be blindly reviewed and discussed during the Blinded Data Review meeting and it will be decided which subjects and/or subject data will be excluded from certain analyses. Decisions made regarding the exclusion of subjects and/or subject data from analyses will be made prior to blind breaking and will be documented and approved by DBV Technologies in the Blinded Data Review minutes.

A summary of the number and percentage of subjects included in each analysis population described below (Intention to treat population [ITT], Safety set, Full analysis set [FAS], Per-protocol population [PP]) will be provided overall and by treatment group.

A by-subject listing of analysis populations (based on screened subjects) will be provided ordered by treatment group and will include: treatment group, subject identifier, and inclusion/exclusion flag for each population and reason for exclusion from each population.

The populations described in the following paragraphs will be considered.

4.4.1 Screened Population

The screened population consists of subjects whose parents/guardians have signed informed consent.

4.4.2 Intent-to-treat Population

The ITT population will be comprised of all subjects who are randomized. This population will be used to assess comparative efficacy information. Subjects will be analyzed according to the treatment they have been randomized to.

4.4.3 Full analysis set

The FAS will be comprised of all ITT subjects who are randomized and have performed at least the peanut challenge of the second DBPCFC at Month 12 (M12). Subjects will be analyzed according to the treatment they have been randomized to.

4.4.4 Per-protocol Population

A PP population will be used to perform sensitivity analyses of the primary and secondary evaluations. This PP population will include all subjects from the ITT population who do not have major deviations from the protocol that may affect the primary (and secondary) efficacy endpoints. The deviations to consider will be listed more in detail in Appendix 1: Major Protocol deviations of this SAP and will be reviewed

during the Blinded Data Review Meeting. This population will be analyzed according to study treatment that was actually received by the subjects. In case the wrong study drug is taken, the subject will be analyzed according to the study drug received for the longest period of time. In the event that a subject is stratified incorrectly (i.e. the screening ED recorded in the IWRS differs from the actual one), actual screening ED value (Screening ED subgroup) will be used rather than 'randomized stratum'.

4.4.5 Safety Population

The safety population will be comprised of all subjects who are randomized and have received at least one dose of study drug. This population will be used to assess comparative safety information. In case the wrong study drug is dispensed, the subject will be analyzed according to the study drug received for the longest period of time. In the event that a subject is stratified incorrectly, 'actual' screening ED value will be used rather than 'randomized stratum'.
A subject is assumed to have received at least one dose of study drug:

- If the date/time of first dose is completed in the eCRF
- Or if at least one patch adhesion scoring is completed (in case the date of first dose is missing)

4.5 Demographics and Baseline characteristics (including medical history, parental atopic history and disease history)

Demographic, Baseline characteristics (including medical history, parental atopic history and disease history) will be summarized by treatment group and overall for the Safety population (globally and by screening ED subgroup) as well as for the ITT population and for the FAS. If the Safety population is identical to the ITT population, tables will not be duplicated and ITT population will be used.
Balance between treatment groups at Baseline on the Safety population (globally and by screening ED subgroup) will be assessed using statistical tests on a selection of key variables, identified in the below sections.
Statistical tests used will be:

- a Student's t–test for continuous variables anticipated to have a normal distribution
- a Mann-Whitney test for continuous variables anticipated to have a non-normal distribution
- a χ^2 test for categorical variables (Fisher's exact test if one or more theoretical frequency is ≤ 5).

All subject demographic characteristics and Baseline characteristics will be listed.

4.5.1 Demographic variables

Demographic variables include:

- Age (years) populated by the IWRS system,

- Age in classes: 4-5 and 6-11, split as 6-8 and 9-11 years old (respectively 4 to 6 (excluded), 6 to 12 (excluded), 6 to 9 (excluded) and 9 to 12 (excluded)),
- Sex,
- Race/Ethnic origin as collected in the eCRF (Caucasian, Black, Hispanic, Asian, Other),
- Race using the following mapping: White (e-CRF value = "Caucasian"), Black or African American (e-CRF value = "Black"), Asian (e-CRF value =" Asian"), Other (e-CRF value = "Hispanic" or "other")

When the date of birth is partial (only year available or year and month), the earliest possible date will be considered (e.g. first of January or first day of the month). Comparability at Baseline will be assessed using statistical tests on age, age by category and sex.

4.5.2 Baseline Characteristics

Baseline characteristics include:

- Body weight (kg) [weight (in kg)] = weight (in lb.) / 2.204623]
- Height (cm) [height (in cm) = 2.54 × height (in inches)]
- Body surface area (m^2): $0.007184 \times (\text{height(cm)}^{0.725}) \times (\text{weight(kg)}^{0.425})$,
- Body mass index (kg/m^2): weight / (height(m)×height(m)),
- Spirometry: FEV1 value (L), FEV1 Percent Predicted (%), (also to be reported by age class 4-5, 6-8 and 9-11 years)
- Peak expiratory Flow (PEF): PEF value (L/min), PEF percent predicted (%), (also to be reported by age class 4-5, 6-8 and 9-11 years),
- Immunological markers: peanut-specific IgE (kU/L), peanut-specific IgG4 (mg/L),
- Skin Prick Test (SPT): Mean and longest wheal diameter for undiluted Peanut extract (mm) (Longest wheal diameter will be also reported for the following age classes: 4-5 and 6-11)
- Medical history (see Section 4.5.3)
- Parental atopic medical history (see Section 4.5.4)
- Disease history (see Section 4.5.5)
- Screening ED subgroup and screening ED randomization stratum
- Filaggrin mutation group: subjects with mutation in the filaggrin gene (Heterozygous / Homozygous) versus wild type subjects.
    - A filaggrin mutation is defined as a positive result ("heterozygous", "homozygous" or "indeterminate") on at least one of the following gene loci: R501X, 2282Del4, R2447X, S3247X, 3702delG.
        - Heterozygous is defined as at least 1 mutation in a single gene locus, or more than 1 mutation in different gene loci (at least one gene with "heterozygous" or "indeterminate" result, no gene with "homozygous" result)
        - Homozygous is defined as 2 identical mutations at the same locus (at least one gene with "homozygous" result)

- Wild type subjects will be identified as "Negative detection" (no positive results on all five genes)

- Comparability at Baseline will be assessed using statistical tests on Body mass index, Immunological markers, skin Prick Test, filaggrin mutation group, Screening ED subgroup and screening ED randomization stratum.

4.5.3 Medical history

Medical history covers the collections of information on past or current conditions (apart from peanut allergy) along with start and end dates including:

- Any allergy other than peanut
  - PT terms which contains "ALLERG" (excluding LLT "Peanut allergy") and PT terms which contains "HYPERSENSITIVITY" (excluding LLT "Peanut allergy")
- Other atopic conditions (Exhaustiveness of the list of terms specified below will be reviewed during the data review meeting):
  - Asthma
    - SMQ Asthma/bronchospasm MedDRA Version 19.1 from narrow terms: List of PT terms: Asthma, asthma exercise induced, asthma late onset, asthmatic crisis, bronchospasm, bronchial hyperreactivity, infantile asthma, status asthmaticus, wheezing
  - Eczema/Atopic Dermatitis
    - List of PT terms: dermatitis atopic, dermatitis Allergic, Eczema, Application site eczema, Eczematous dermatitis, dermatitis contact.
  - Allergic Rhinitis
    - List of PT terms: Rhinitis Allergic, seasonal allergy, Rhinitis perennial, conjunctivitis allergic
- Past or current medical conditions:
  - Cardiovascular, respiratory (including asthma), gastrointestinal, renal, hepatic, neurological, endocrine, lymphatic, hematologic, immunologic, dermatological (including atopy), psychiatric, developmental, and genitourinary disorders, drug and surgical history and any other diseases or disorders.

They will be coded using the latest available version of the Medical Dictionary for Regulatory Activities (MedDRA) dictionary and will be reported by SOC and PT. The number and percentage of subjects with at least one medical history term will also be provided

Comparability at Baseline will be assessed using statistical tests on subjects with/without asthma, with/without eczema/atopic dermatitis, with/without allergic rhinitis and with/without any other allergy than peanut.

4.5.4 Parental atopic medical history

Parental (father only, mother only, or both parents) atopic medical history covers: asthma, seasonal allergies, perennial allergies, food allergies, eczema/atopic dermatitis and other allergic diseases. The number and percentages of subjects with at least one parental atopic medical history will also be provided.
In case a subject's parent is not the biological parent, no parental medical history is collected about this parent.

4.5.5 Disease history

Disease history covers the collection of information about the peanut allergy of the subject and will be reported using the following characteristics:

- Age at peanut allergy diagnosis (in years, calculated as Date of diagnosis – Date of birth divided by 365.25),
- Time since peanut allergy diagnosis (in years, calculated as Date of Informed consent - Date of diagnosis divided by 365.25),
- Category of the physician who made the diagnosis,
- Main reason having led to the diagnosis (reaction after ingestion, parental/sibling history of atopy, other risk factors)
- Diagnosis criteria (Allergic reaction(s) following peanut consumption, Positive SPT to peanut, Positive titer of peanut-sIgE, Positive Double-Blind Placebo-Controlled FC to peanut, Positive Single-Blind Placebo-Controlled FC to peanut, Positive Open FC to peanut)
- Most recent results of peanut allergy diagnostic tests performed,
- Description of allergic reactions:
    - Number of allergic reactions after ingestion of peanut,
    - Number of allergic reactions after ingestion of peanut in the previous 12 months,
    - Time since last reaction after ingestion of peanut (in years, calculated as Date of Informed consent - Date of last reaction divided by 365.25). When the date of last reaction is partial (only year available or year and month), the latest possible date will be assumed (e.g. 31st of December or last day of the month).
    - Allergic reaction after ingestion of peanut after the age of 2 years old

When the date of diagnosis is partial (only year available or year and month), the latest date between the date of birth and the earliest possible date of diagnosis (e.g. first of January or first day of the month) will be considered.

4.6 Previous and concomitant medications

All medications taken before the study entry, at study entry and during the study will be recorded and coded using the latest available version of the World Health Organization (WHO) Drug Dictionary.
Medications will be classified either as "Prior only" (P), "Both Prior and Concomitant" (PC) or "Concomitant only" (C).

This classification will be made by comparing the study medication start and stop dates with the date of first application of study medication (or V4 date if date of first patch application is not completed).
Medications starting after the completion/withdrawal date will be listed but will not be classified or summarized.
"Prior only" (P) medications will be:

- Medications that were taken within 6 months prior to the date of first application of study medication and that stopped before the first application of study medication,
- Medications with partial start/stop date where there is clear evidence to suggest that the medication stopped within 6 months prior to the date of first dose of study medication.

"Both prior and concomitant" (PC) medications will be:

- Medications starting before the date of first application of study medication and stops on or after the date of first application of study medication,
- Medications with partial start/stop date where there is clear evidence to suggest that the medication started prior to the date of first dose of study medication (no evidence for the stop date).

"Concomitant only" (C) medications will be:

- Medications with a start date on or after the date of first dose of study medication,
- Medications with partial start/stop date where there is clear evidence to suggest that the medication started after the date of the first dose of study medication,
- Medications with partial or complete stop date where there is clear evidence to suggest that the medication stopped after the date of the first dose of study medication (no evidence for the start date or V4 date if date of first application is not completed),
- All other medications with partial start/stop date where there is no clear evidence to suggest that the medication started or stopped before or after the date of the first dose of study medication.

For prior and/or concomitant medications, number and percent of subjects overall and per ATC class (level 3) and preferred drug name will be calculated. The following summaries will be presented:

- Prior medications (other than the ones taken during the food challenge at Baseline), prior only medications (P) will be taken into account;
- Concomitant medications (other than the ones taken for the food challenge at M12 and the ones taken for voluntary or accidental consumption of peanut), both prior and concomitant (PC) and concomitant (C) medications will be taken into account;
- Medications taken for food challenges, by time point;
- Concomitant medications taken due to accidental/voluntary consumption of peanut.

Number of antihistamines and number of epinephrine intakes during food challenges will also be tabulated separately (for the Peanut and Placebo days of DBPCFC separately).
Non-drug therapies and surgical procedures are collected and will be coded using the latest available version of the MedDRA Dictionary. They will be also presented by SOC and preferred term and listed.

Prior and concomitant medications as well as Non-drug therapies and surgical procedures will be summarized by treatment group and overall for the safety population.

All prior and concomitant medications, non-drug therapies and surgical procedures will be listed.

4.7 Study duration, treatment exposure and compliance

The overall study duration for each subject will be approximately 61 weeks (6-week screening period, 12-month treatment period and 2-week follow-up period).
Study duration (days) will be calculated as:

- Last study visit date - Date of informed consent +1.

During the 12-month blinded treatment period, the Viaskin® patch must be applied on the skin for 24 hours every day, except during the initiation of treatment, where the duration of application of the Viaskin® patch will be progressively increased as follows:

- During the first week (from Day 1 through Day 7), the patches will be applied for 6 hours every day,
- During the second week (from Day 8 through Day 14), the patches will be applied for 12 hours every day,
- From the third week onwards (Day 15), the patches will be applied for the entire 24 hours daily.

Treatment exposure will be based on:

- The exposure duration (in days, regardless of treatment interruption), calculated for the following periods:
  - Over the whole study period as: Date of last patch application – Date of first patch application +1,
  - At each study visit (Day 8, Month 1, 3, 6, 9, 12) as: Date of last patch application before the visit – Date of first patch application at the preceding visit+1.
- The total dose of peanut protein (in mg) received via the patch during the study, calculated for the following periods:
  - Over the whole study period as: Exposure duration (in days) * Actual treatment dosage,
  - At each study visits, as: Exposure duration at the visit (in days) * Actual treatment dosage.

The compliance (%) determined for the following periods:

- Over the whole study period as:

$$100\left(\frac{Number\ of\ patches\ dispensed - Number\ of\ patches\ returned}{Exposure\ duration\ in\ days}\right)$$

- At each study visit (Day 8, Month 1, 3, 6, 9, 12) as:

$$100\left(\frac{Nb\ of\ patches\ dispensed(visit\ n-1) - Nb\ of\ patches\ returned(visit\ n)}{Date\ of\ (visit\ n) - Date\ of\ (visit\ n-1)(1)}\right)$$

(1) Date of current visit – Date of previous visit+1 for Day 8

Compliances exceeding 100% will be set to 100%.
Global compliance of at least 80% over the treatment period is sought.

Additionally, the average daily application duration (in hours) will be summarized descriptively using the subject diary data for the following periods:

- D1 to D7,
- D8 to D14,
- D15 to M3 (D90),
- M3 (D91) to M6 (D185),
- M6 (D186) to M9 (D275),
- M9 (D276) to M12 (end of treatment),
- D15 to M12

If more than one patch is applied the same day, the daily duration considered is the cumulative application duration of these patches.
In case patches are sequentially applied on the same day: if the end date/time of first patch is not documented, the application date/time of the next patch will be used for imputation.

The percentage of days with no patch application, per subject diary reported data, will also be tabulated, overall and for each of the above periods.

A patch will be considered as "applied" if the date and time of application is documented in the diary.
The start and/or end date/time of patch application used to calculate the duration of patch application are extracted from the diary.
Inconsistencies in the diaries are not queried and only self-evident corrections according to the Data Validation specifications will be made; therefore, inconsistent date/time of patch application or patch removal may remain in the data. In case where calculation of patch application duration leads to a negative value or duration cannot be calculated (missing date or time of removal or invalid dates or times) then:

- The duration of patch application will be considered as 'unknown' and not used in the calculation of the average duration of patch application
- For any other calculation based on the number of days with patch application, this day will be considered as a day with application.

These data will be reported as:

- Descriptive statistics for study duration, treatment exposure duration, total dose of protein and compliance (overall and by visit - except for study duration -),
- Number and percent per category of overall exposure (in days, categories are: 1-7, 8-14, 15-30, 31-90, 91-185, 186-275, 276-365, >365), compliance in classes (<80%, ≥80%).

Study duration, treatment exposure and compliance will be summarized for the Safety population in tables that will be produced for each treatment group and overall (except for peanut protein dose that will only be described for the Viaskin Peanut 250 µg arm), globally and by screening ED subgroup.
All data including documentation of treatment interruptions will be listed by treatment group.

4.8 Efficacy Evaluation

4.8.1 Analysis and Data Conventions

The primary efficacy endpoint in this study is the difference between percentage of treatment responders in the active Viaskin® Peanut 250 µg group compared to the placebo group after 12 months of EPIT treatment.

4.8.1.1 Multi-center Studies

Considering the expected small number of subjects to be randomized by center, the center effect will not be investigated. Nevertheless, the region (Australia/ Europe/ North America) effect will be investigated on the ITT population for the primary efficacy endpoint.

4.8.1.2 Handling of Dropouts or Missing Data

Primary and Secondary Analyses
Analyses of the efficacy endpoints (primary and secondary) will be based on the ITT population. In the event of missing data imputation rules will be incorporated in respective analytical details.

Cumulative Reactive Dose (CRD)
The cumulative reactive dose of peanut (CRD) will be calculated as follow

- If the ED reported by the investigator in the eCRF is missing, then the CRD is missing
- If the ED reported by the investigator in the eCRF is not missing then the CRD is calculated as the sum of all doses given, including also the repeated and partial doses; doses greater than the ED are not included in the calculation. Example: For a particular patient if the set of recorded doses is $\{1, 3, 10, 30, 100, 300, 300, 1000\}$, and recorded ED = 300mg, then $CRD = 1 + 3 + 10 + 30 + 100 + 2\times300 = 744mg$.

No imputation will be performed for other efficacy, safety and exploratory parameters. In those instances observed data will be used, excepted for safety data where partial or missing data will be imputed according to the most conservative approach (See Section 4.9.1: Adverse Events).

Actual values will be presented in listings.

4.8.1.3 Multiple Comparisons/Multiplicity

In order to handle the multiple comparisons versus placebo, the overall type-I error will be controlled at a level of 5% (2-sided) by the use of a hierarchical inferential approach. The primary analysis in the overall population must be positive according to the clinical relevance criterion (described in section 4.8.2) before drawing inferential conclusions about the secondary comparisons as defined by the hierarchical order. The first secondary efficacy analysis (difference in treatment response in Screening ED subgroup 2) must meet its success criterion before drawing an inferential conclusion about the next secondary comparison (difference in treatment response in Screening ED subgroup 1). Inferential conclusions about the next successive secondary comparisons require meeting the success criterion of the previous comparison. The pre-defined hierarchical order and success criteria are summarized in Table 2.

Table 2: Pre-defined Hierarchical Order for Analysis of Efficacy Endpoints

Order	Efficacy endpoints (at M12)	Population or sub-group	Success criterion	Method / SAS® Procedure
1	Difference in percentages of treatment responders	ITT Overall	95% CI lower bound ≥15%	2-sided Newcombe 95% CI (SAS® FREQ procedure with RISKDIFF option)
2	Difference in percentages of treatment responders	ITT Screening ED subgroup 2	95% CI lower bound >0%	2-sided Newcombe 95% CI (SAS® FREQ procedure with RISKDIFF option)
3	Difference in percentages of treatment responders	ITT Screening ED subgroup 1	95% CI lower bound >0%	2-sided Newcombe 95% CI (SAS® FREQ procedure with RISKDIFF option)
4	Cumulative reactive dose	ITT Overall	p ≤0.05	ANCOVA
5	Peanut protein ED	ITT Overall	p ≤0.05	ANCOVA
6	Difference in percentages of treatment responders	ITT Age group 6-11 years of age	95% CI lower bound >0%	2-sided Newcombe 95% CI (SAS® FREQ procedure with RISKDIFF option)
7	Difference in percentages of treatment responders	ITT Age group 4-5 years of age	95% CI lower bound >0%	2-sided Newcombe 95% CI (SAS® FREQ procedure with RISKDIFF option)
8	Difference in percentage of subjects responsive to a cumulative dose ≥1,444 mg peanut protein	ITT Overall	95% CI lower bound >0%	2-sided Newcombe 95% CI (SAS® FREQ procedure with RISKDIFF option)
9	Difference in percentage of subjects unresponsive to a cumulative dose ≥1,444 mg peanut protein	ITT Overall	95% CI lower bound >0%	2-sided Newcombe 95% CI (SAS® FREQ procedure with RISKDIFF option)
10	Cumulative reactive dose	ITT Screening ED subgroup 2	p ≤0.05	ANCOVA

Order	Efficacy endpoints (at M12)	Population or sub-group	Success criterion	Method / SAS® Procedure
11	Peanut protein ED	ITT Screening ED subgroup 2	$p \leq 0.05$	ANCOVA
12	Cumulative reactive dose	ITT Screening ED subgroup 1	$p \leq 0.05$	ANCOVA
13	Peanut protein ED	ITT Screening ED subgroup 1	$p \leq 0.05$	ANCOVA
14	Difference in percentage of subjects passing the challenge (percentage of subjects unresponsive to the highest dose of peanut protein)	ITT Overall	95% CI lower bound >0%	2-sided Newcombe 95% CI (SAS® FREQ procedure with RISKDIFF option)

Abbreviations: CI = Confidence Interval; p = p-value; ANCOVA = Analysis of covariance; ED = Eliciting dose; ITT = Intent-to-treat.

4.8.1.4 Interim Analyses

No interim analyses are planned. Hence no adjustments for multiplicity are required.

4.8.1.5 Examination of Subgroups

The treatment effect for the primary efficacy variable will be examined as secondary efficacy analyses in each of the subgroup defined according to the screening ED.
Sample size has been calculated to demonstrate a treatment difference at the level of 5% in each of the screening ED stratum based on a lower bound of the 2-sided Newcombe 95% CI of the difference between active treatment and placebo response rates >0%:

- At a power of 99% in stratum 1, with an expected response rate of 60% in the active treatment vs. 10% for placebo, both estimated using missing=failure method
- At a power of 95% in stratum 2, with an expected response rate of 30% in the active treatment vs. 10% for placebo, both estimated using missing=failure method.

To handle cases of randomization stratum error (i.e. the stratum recorded in the IWRS was not consistent with the actual screening ED value), screening ED subgroups were considered for secondary analyses using the actual screening ED value (i.e. subjects will be considered in the screening ED subgroup corresponding to the actual screening ED value).

The treatment effect for the primary efficacy variable will also be examined as secondary

efficacy analyses in each of the following age groups: 4-5 year of age, 6-11 year of age (respectively [4-6[, [6-12[).

Response rates by treatment groups and treatment effect will also be provided descriptively, as exploratory analysis, for each region and on the following subgroups:

- Gender (Male/Female)
- Race (White/Black or African American/Asian/Other)
- Subjects with / without history of asthma
- Subjects with / without history of allergy other than peanut
- Subjects with / without history of atopic dermatitis or eczema.

Analysis will be performed only on subgroups containing at least 15 subjects.
According to the sample size of each subgroup (if N<50), exact 2-sided 95% CIs will be provided instead of 2-sided Newcombe 95% CIs for the difference in response rates. In the presence of sparse data (i.e. at least one of the percentages is zero) the following will not be presented:

- within-treatment confidence intervals around response rate (of 0%),
- between-treatment comparative p-value.

A forest plot will be provided summarizing the primary efficacy variable in the above subgroups.

No other subgroup analysis will be performed.

4.8.2 Primary Efficacy Analysis

The primary efficacy analysis is based on an endpoint that is derived from the Eliciting dose (ED).
The eliciting dose, i.e. dose at which objective symptoms leading to ending the DBPCFC occurred, is documented by the investigator in the eCRF.
In the following two cases, the ED at the M12 DBPCFC is not documented in the eCRF by the investigator:

- If the M12 DBPCFC is stopped without any objective symptoms leading to ending the DBPCFC (stopping rules) or
- if the subject took the 2,000mg dose without any objective symptom leading to ending the DBPCFC.

In those cases, the last dose given at M12 DBPCFC will be considered for ED value.
Partially ingested dose:
If the last dose given is only partially ingested by the subject prior to the DBPCFC stop, then the ED reported by the investigator is:

- the previous dose given if the quantity of the last dose given actually ingested by the subject is ≤ to the previous dose ingested;
- the last dose given if the quantity of the last dose given actually ingested by the subject is > than the previous dose ingested.

Main analysis

The primary efficacy endpoint is the difference between percentage of treatment responders in the active Viaskin® Peanut 250 µg group compared to the placebo group after 12 months of EPIT treatment. A subject is defined as a treatment responder if:

- The initial ED was ≤10 mg peanut protein (Screening ED subgroup 1) and the ED is ≥300 mg peanut protein at the Month 12 DBPCFC; or
- The initial ED was >10 mg peanut protein (Screening ED subgroup 2) and the ED is ≥1,000 mg peanut protein at the Month 12 DBPCFC.

The primary efficacy analysis will be performed on the ITT population, using missing=failure imputation method (which means that subjects with missing DBPCFC peanut eliciting dose value at M12 will be considered as non-responders).
The primary measure of treatment effect will be the difference in response rates between active and placebo treatment groups. The primary analysis will apply a Wald test at a 2-sided 5% significance level to evaluate a null hypothesis of no difference, and the corresponding 2-sided Newcombe 95% confidence interval (CI) for the difference in response rates will be calculated. The clinical relevance criterion for the primary analysis will be defined by a ≥15% lower confidence bound, and this condition will determine whether the primary objective has been successfully met.
The Wald test p-value will be calculated using the SAS® GENMOD procedure, with DIST=binomial and LINK=identity option of the MODEL statement.

The 2-sided Wilson 95% CIs of individual response rate will be presented overall and by treatment group using the SAS® FREQ procedure, with the RISKDIFF option.

All methods described above will be performed using SAS® procedures described in Appendix 2: SAS® Code lines

The study will be considered positive if the lower bound of the 95% CI of the difference between active treatment and placebo response rates is higher or equal to 15%. This 15% difference is intended to exhibit a clear robustness of the treatment effect.

Sensitivity analyses

Several sensitivity analyses of the primary efficacy variable will be performed by running a primary efficacy analysis either on different analysis sets, or with alternative imputation methods for missing data or using other or additional covariates.
The sensitivity analyses that will be performed are the following:

Sensitivity to analysis set:

i. On the FAS, with no imputation for missing data (subjects with missing M12 peanut challenge are excluded from the FAS)
ii. On the PP population, with no imputation for missing data (subjects with missing M12 peanut challenge are excluded from the PP population)

Sensitivity to handling of missing data:

iii. On the ITT population, using multiple imputation method.
Specifically, the probability distribution that imputes the missing response will rely on placebo subjects with an available response and so the missing data would mimic placebo response. Imputations will be performed using SAS® MI procedure generating 1000 sets of imputed data. Seed used for imputation will be specified (details on the implementation of multiple imputations are given in Appendix 2.2).

iv. On the ITT population, using worst case imputation for missing data (i.e. subjects with missing M12 peanut challenge in the placebo group to be considered as responders whereas subjects in the Viaskin® group considered as non-responders)

Sensitivity to adjustment on covariates:

v. On the ITT population, using missing=failure imputation for missing data, Cochran-Mantel-Haenszel (CMH) method with Screening ED randomization stratum (Screening ED > 10 mg/Screening ED ≤ 10 mg) as stratification variable to compute a Newcombe 95% CI for the strata-adjusted difference in response rates.

vi. On the ITT population, using missing=failure imputation for missing data, Cochran-Mantel-Haenszel (CMH) method with region as stratification variable to compute a Newcombe 95% CI for the region-adjusted difference in response rates.

vii. On the ITT population, using missing=failure imputation for missing data, Cochran-Mantel-Haenszel (CMH) method with age group (4-5 years of age/ 6-11 years of age) as stratification variable to compute a Newcombe 95% CI for the age group-adjusted difference in response rates.

Regarding the sensitivity analysis (iii) on the ITT population using the multiple imputation method [4] for missing data, the strategy used is described in

Appendix 2.2: Strategy of analysis of the primary efficacy endpoint using multiple imputation of missing data.

In case of inconsistency between the primary analysis results and sensitivity analysis results, additional analyses will be performed to explore the reasons.

All data (raw and derived) related to the primary efficacy analysis and its sensitivity analyses will be tabulated in by-subject listings.

4.8.3 Secondary Efficacy Analyses

4.8.3.1 Percentage of treatment responders in each screening ED subgroup

The two first secondary efficacy analyses are the comparisons of the percentage of treatment responders in each screening ED subgroup, in the ITT population.
These analyses will be repeated as sensitivity analyses:

i. On the FAS,
ii. On the PP population

No imputation for missing data will be performed since subjects with missing M12 peanut challenge are excluded from these two populations.

The same analyses methods as the ones performed for the primary efficacy analysis will be used. The clinical relevance of the difference in response rates in each screening ED subgroup will be evaluated based on the lower bound of the 2-sided Newcombe 95% CI > 0%. The p-value based on the Wald test will be presented. In the case of sparse data (at least one of the percentages is zero), no individual response rate confidence intervals and no p-value will be presented.

According to the sample size of each screening ED subgroup (if N<50), a Fisher's exact test will be performed instead of the Wald test, in which case exact 2-sided 95% CIs will be provided for the difference of response rate.

With respect to the hierarchical procedure described in Table 2, inference of a treatment effect in ED subgroup 2 will be contingent on ruling out a <15% difference in response rates in the overall population (the primary analysis), and inference of ED subgroup 1 will be contingent on ruling out a <0% difference in response rates in ED subgroup 2.

4.8.3.2 Percentage of treatment responders in each age group (4-5 and 6-11 years of age)

The difference in percentage of treatment responders will also be evaluated in each age group, in the ITT subpopulations.

These analyses will be repeated:

i. On the FAS,
ii. On the PP population.

The same analyses methods as the ones performed for the primary efficacy analysis will be used. The clinical relevance of the treatment effect in each age group will be evaluated based on the lower bound of the 2-sided Newcombe 95% CI > 0%. The p-value based on the Wald test will be presented. In the case of sparse data (at least one of the percentages is zero), no individual response rate confidence intervals and no p-value will be presented.

According to the sample size of each subgroup (if N<50), a Fisher's exact test will be performed instead of the Wald test, in which case exact 2-sided 95% CIs will be provided for the difference of response rate.

With respect to the hierarchical procedure described in Table 2, the success criterion for the comparison of treatment responders between treatments in the 6-11 years of age group (ITT) [Number 6 in the pre-defined hierarchical order of the efficacy analyses] will be claimed if the Newcombe 95% lower CI bound is ≥0 and the results for the previous efficacy comparisons in the hierarchical order met their success criteria, and the same would apply for the next comparison in the order evaluating the 4-5 years of age group (ITT) [Number 7 in the pre-defined hierarchical order of the efficacy analyses].

4.8.3.3 Cumulative reactive dose of peanut protein and change from Baseline at Month 12

The following variables will be tabulated by treatment group using descriptive statistics:

- Peanut protein cumulative reactive dose (CRD) at Baseline and at M12
- Change in Peanut protein cumulative reactive dose at M12 since Baseline

The above statistics will be tabulated overall and for each screening ED subgroup:

- On the ITT population (using modified Baseline Observation Carried Forward [mBOCF] method to impute missing data at Month 12),

mBOCF for CRD is defined as follow:
- If the DBPCFC is stopped without the objective symptoms leading to ending the DBPCFC (stopping rules) or if the subject took the 2,000mg dose without any objective symptom leading to ending the DBPCFC, the CRD value at M12 is considered as the maximum between:
    - the sum of all doses given at Month 12, including also the repeated and partial doses AND
    - the CRD value at screening
- For the subjects who did not undergo the M12 DBPCFC, CRD at screening is carried forward at M12.

- On the FAS and on the PP population (using observed data)

- If the M12 DBPCFC is stopped without any objective symptoms leading to ending the DBPCFC (stopping rules) or if the subject took the 2,000mg dose without any objective symptom leading to ending the DBPCFC, the CRD at Month 12 is considered as the sum of all doses given at Month 12, including also the repeated and partial doses.

The peanut protein cumulative reactive dose in each treatment group at M12 will be compared using an analysis of covariance (ANCOVA) model. The ANCOVA model will include the treatment group, adjusted for the Baseline cumulative reactive dose value. This comparison will be performed in the overall ITT population using mBOCF imputation [Number 4 in the pre-defined hierarchical order of the efficacy analyses], as well as for each screening ED subgroup [Number 10 & 12 in the pre-defined hierarchical order of the efficacy analyses].

Additionally, this analysis will be repeated on the FAS and the PP population, using observed data, overall and for each screening ED subgroup.

Finally, the M12 peanut protein cumulative reactive dose comparison will also be performed for each age group (4-5 and 6-11 years of age):

- On the overall ITT population (using mBOCF imputation),
- On the FAS (using observed data),
- On the PP population (using observed data).

The ANCOVA will be performed through a mixed procedure. A sample of the SAS® code to be used can be found in Appendix 2.3: Statistical analysis methods and procedures used for the analysis of the cumulative reactive dose of peanut protein at month 12.

The following statistics will be presented:

- The Least square means (LS Means) by treatment group: mean for the variable obtained by the model adjusted on the covariates
- The difference between the LS Means and the associated confidence interval
- The effect size: calculated as the absolute difference in the LS means between the treatment groups divided by pooled standard deviation
- The p-value from the hypothesis test of no difference between the treatment groups based on type III sum of squares.

The treatment effect will be estimated using the difference between the treatment LS means (adjusted means). The treatment difference will be evaluated at the 5% level of significance.

The assumption of normality underlying the statistical model will be tested using the Shapiro-Wilk test. A p-value less than 0.05 will be taken to indicate evidence of non-normality. In addition, a normal probability plot of the studentized residuals will be used to investigate the normality assumption.

The assumption of homogeneity of variance of the treatment groups will be tested using Levene's test. A p-value less than 0.05 will be taken to indicate evidence of heterogeneity of variance.

If the assumptions of normality and/or homogeneity of variance underlying the statistical model are violated, log_{10}-transformation of the data will be considered instead. In that case, the following statistics will be presented:

- The back transformed Least square means: the Geometric Least Square Means by treatment group
- The back transformed difference between the LS Means and the associated confidence interval: the geometric LS Means ratio
- The effect size
- The p-value from the hypothesis test of no difference between the treatment groups based on type III sum of squares.

If the assumptions still do not hold for log_{10}-transformed data, in addition to the analysis of the log_{10}-transformed data, a sensitivity analysis will be performed using an appropriate non-parametric test procedure. The treatment effect will be estimated using Hodges-Lehmann estimate of the difference in medians. The corresponding 95% confidence interval and the p-value from the hypothesis test of no difference between the treatment groups (Wilcoxon rank-sum test) will be presented. This p-value will not be adjusted for multiplicity and provided for descriptive purpose only.

4.8.3.4 Eliciting dose of peanut protein and change from Baseline at Month 12

The following variables will be tabulated by treatment group using descriptive statistics:

- Peanut protein ED dose at Baseline and at M12
- Change in Peanut protein ED dose at M12 since Baseline.

The above statistics will be tabulated overall and for each screening ED subgroup:

- On the ITT population (using mBOCF imputations for missing values at Month 12),

mBOCF for ED is defined as follow:
- If the DBPCFC is stopped without any of the objective symptoms leading to ending the DBPCFC (stopping rules) or if the subject took the 2,000mg dose without any objective symptom leading to ending the DBPCFC, the ED value at M12 is considered as the maximum between:
    - the last dose given at M12 DBPCFC (if the last dose given at M12 DBPCFC is only partially ingested by the subject, then the rules as defined under partially ingested dose in section 4.8.2 will apply) AND
    - the ED value at screening

- For the subjects who did not undergo the M12 DBPCFC, ED value at screening is carried forward at M12.

- On the FAS and on the PP population (using observed data).

- If the M12 DBPCFC is stopped without any of the objective symptoms leading to ending the DBPCFC (stopping rules) or if the subject took the 2,000mg dose without any objective symptom leading to ending the DBPCFC, the last dose given at M12 DBPCFC will be considered for ED value. If the last dose given at M12 DBPCFC is only partially ingested by the subject, then the rules as defined under partially ingested dose in section 4.8.2 will apply.

The peanut protein ED in each treatment group at Month 12 will be compared using the same ANCOVA model as described in Section 4.8.3.3, but adjusted on the Baseline ED [Number 5, 11 and 13 in the pre-defined hierarchical order of the efficacy analyses].
Finally, the M12 peanut protein ED comparison will also be performed for each age group (4-5 and 6-11 years of age):

- On the overall ITT population (using MBOCF imputation),
- On the FAS,
- On the PP population.

4.8.3.5 Percentage of subjects *responsive* (those showing objective symptoms leading to DBPCFC stop) to a cumulative dose ≥1,444 mg peanut protein at the Month 12 DBPCFC

A subject is said to be *responsive* (showing objective symptoms leading to DBPCFC stop) to a cumulative dose of ≥ 1,444 mg of peanut protein at Month 12 if CRD ≥1,444 mg (as recalculated in section 4.8.1.2).

The number and percentage of subjects responsive to a cumulative dose ≥1,444 mg peanut protein at the Month 12 DBPCFC will be tabulated overall and for each screening ED subgroup on the ITT population, as well as on the FAS and PP population.
The percentage of subjects responsive to a cumulative dose ≥1,444 mg peanut protein at M12 will also be tabulated for each age group (4-5 and 6-11 years of age), on the overall ITT, FAS and PP populations.

The percentage of subjects responsive to a cumulative dose ≥1,444 mg peanut protein at M12 in the overall ITT population, using "Missing=failure" imputation, will be compared using the difference in treatment rates between the active treatment and placebo and the corresponding 2-sided Newcombe 95% CI [Number 8 in the pre-defined hierarchical order of the efficacy analyses]. The success criterion for this endpoint will be evaluated based on the lower bound of the 2-sided Wald 95% CI > 0%. Additionally, a p-value based on the Wald test will be presented. In the case of sparse data (at least one of the

percentages is zero), no individual response rate confidence intervals and no p-value will be presented.
The same SAS® procedures as for the analysis of primary efficacy endpoint will be run.
According to the sample size of each subgroup (if N <50), a Fisher's exact test will be performed instead of the Wald test, in which case exact 2-sided 95% CIs will be provided.

This analysis will be repeated on the FAS and PP population.

4.8.3.6 Percentage of subjects *unresponsive* (those showing no objective symptoms leading to DBPCFC stop) to a cumulative dose ≥1,444 mg peanut protein at the Month 12 DBPCFC

A subject is said to be *unresponsive* to a cumulative dose of ≥ 1,444 mg of peanut protein at Month 12 if:

- the sum of all doses given at Month 12, including repeated and partial doses is ≥ 1,444 mg; AND
- showing no objective symptoms which would trigger stopping of the DBPCFC (ED value entered by the investigator is missing).

The number and percentage of subjects *unresponsive* to a cumulative dose ≥1,444 mg peanut protein at the Month 12 DBPCFC will be reported and analyzed the same way as the percentage of subjects responsive to a cumulative dose ≥1,444 mg peanut protein at the Month 12 DBPCFC.

The analysis run on the ITT using "Missing=failure" imputation is the analysis [number 9 in the pre-defined hierarchical order of the efficacy analyses].

4.8.3.7 Percentage of subjects *unresponsive* (those showing no objective symptoms leading to DBPCFC stop) to the highest dose of peanut protein which is the percentage of subjects who pass the Month 12 DBPCFC

Subjects *unresponsive* to the highest dose of peanut protein are defined as subjects:

- for which the sum of all doses given at Month 12, including repeated and partial doses is ≥ 3,444 mg; AND
- showing no objective symptoms which would trigger stopping of the DBPCFC (ED value entered by the investigator is missing).

The analysis of this variable will be performed the same way as for the two preceding variables. The analysis run on the ITT using "Missing=failure" imputation is the analysis number 14 in the pre-defined hierarchical order of the efficacy analyses.

4.8.4 Other Efficacy Analyses

No imputation for missing data is used for Other Efficacy variables, i.e. only observed data are presented, except for laboratory results reported as below the lower detection limit (or reported above a specific value) which will be imputed as described in Section 4.2.

4.8.4.1 Peanut-specific IgE and IgG4 over time

Descriptive analysis of peanut-specific IgE and IgG4 over time including actual values, absolute and relative changes from Baseline at Month 3, Month 6 and Month 12 will be provided for the overall ITT population and by screening ED subgroup.
Repeated-measures ANCOVA models will be built to compare the mean absolute change from Baseline value in peanut-specific IgE and IgG4 using all time points evaluated up to M12 in the active Viaskin® Peanut 250 µg group versus the placebo group on the ITT population using observed data. The possibility of running the model on log_{10}-transformed data will be investigated. The repeated-measures ANCOVA model will be run globally and for each screening ED subgroup. Treatment group, treatment-by-time point interaction, Baseline value and screening ED subgroup (only for the model run on the global ITT population) will be included in the model.
The SAS® code used is listed in Appendix 2.4: Statistical analysis methods and procedures used for the analysis over time of peanut-specific IgE and IgG4.
Statistics reported will include: Adjusted Least Square (LS) Mean and standard error of the absolute change from Baseline at each time point (Month 3, 6 and 12), together with the LS mean difference versus Placebo, the associated 95% confidence interval and the corresponding p-values at each time point. P-value for treatment effect all time points taken together will also be provided.
Mean (±SD) and median relative (+/- IQR) change from Baseline in IgE and IgG4 will be presented graphically, by treatment group, for the overall ITT population as well as for each screening ED subgroup.
The log_{10} transformation of the ratio between peanut specific IgG4 and peanut specific IgE, calculated as:

$$log10\left(\frac{Peanut\ specific\ IgG4(mg/L)\times 1000}{Peanut\ specific\ IgE\ (kU/L)\times 2.4}\right)$$

will be tabulated for the ITT population at each time point:

- by treatment group and overall;
- for each screening ED subgroup;
- for the 2 age subgroups: 4 to 5 years-old; 6 to 11 years-old;
- for treatment responders vs. non responders (primary endpoint), by treatment group and overall.

For patients treated with Viaskin® 250 µg:

- The association between change in cumulative reactive dose and the log_{10} of the ratio will be evaluated through an ANCOVA (SAS® MIXED procedure) with cumulative reactive dose as the outcome and the log_{10} ratio as the single factor.

- The association between treatment response and the $\log_{10}$ of the ratio will be evaluated through a generalized linear model (SAS® GENMOD procedure) with treatment response as the outcome and the $\log_{10}$ ratio as the single factor.

4.8.4.2 Skin prick test mean wheal diameters over time and at Month 12

Descriptive analysis of SPT mean wheal diameter over time including actual values and absolute change from Baseline at Month 3, Month 6 and Month 12 will be provided for the overall ITT population and by screening ED subgroup.
The mean wheal diameter at M12 in each treatment group will be compared using an analysis of covariance (ANCOVA) model on the ITT population on observed data. The ANCOVA model will be run globally and for each screening ED subgroup. The model will include the treatment group, adjusted for the Baseline value and the screening ED subgroup (only for the model run on the global ITT population) as covariates.
The process followed for the analysis and the SAS® code used will be the same as the one described in 4.8.3.3.

4.8.4.3 Descriptive analysis of the quality of life questionnaires (Food Allergy Quality of Life Questionnaire [FAQLQ]/Food Allergy Independent Measure [FAIM]) and change from Baseline at Month 12.

Food Allergy Quality of Life Questionnaires (FAQLQ)

The FAQLQs (Child Form [CF] and Parent Form [PF]) are disease-specific health-related quality of life questionnaires for subjects with food allergy.

At screening and at M12, the following questionnaires will be completed (for countries where translated and validated questionnaires are available and used):
By subjects, for subjects ≥8 years of age (at screening):

- FAQLQ-CF: 24 items,

By parents for all subjects:

- FAQLQ-PF: 26 items for subjects aged 4 to 6; 30 items for subjects age 7 and older (at screening),

Age at screening will be used to define which items will be applicable during the study for each subject. Items not applicable will be not used for analysis. The sub-scores rely on different numbers of items according to the age-groups (4-6 and 7-12 years old).

FAQLQ questionnaires will be summarized on specific scores calculated by main domains of quality of life. FAQLQ-CF will be analyzed according to the 4 following domains [1]:

- EI: Emotional impact (item no: 19-24),
- AA: Allergen avoidance (item no: 4, 6-10, 15),

- RAE: Risk of accidental exposure (item no: 11, 13-14, 16-17),
- DR: Dietary restriction (item no: 1-3, 5, 12, 18).

FAQLQ-PF will be analyzed according to the 3 following domains [2]:

- EI: Emotional impact (item no: 2, 6-7, 9-11, 23-28, 30),
- FA: Food-related anxiety (item no: 1, 4-5, 16-17, 20-21, 29),
- SDL: Social and Dietary limitations (item no: 3, 8, 12-15, 18-19, 22).

The following instructions from the reference website (www.faqlq.com) will be used for the derivations of (sub-) scores:

1) Each question of the FAQLQ is answered on a 7-point scale (0 to 6) and should be recoded 1 to 7. (Note: FAQLQ-CF items are coded on a scale of 1-7 in the CRF and in SDTM)

2) The total FAQLQ-scores (except for FAQLQ-PF for which the total score is equal to the sum of the three-scores score divided by 3) and all sub-scores are calculated by dividing the sum of completed items by the number of completed items

3) If > 20% of items in any (sub-)domain are missing then the respective (sub-)score is set to missing

Baseline and Month 12 values for each item of the FAQLQ questionnaires will be summarized overall and by treatment group, in the ITT population.
Baseline, Month 12 and change from Baseline of the the FAQLQ scores (global score, scores by domain) will be summarized overall and by treatment group, in the ITT population.
The scores range from no problem/impairment to maximal problem/impairment. A negative score in the changes from baseline means impairment.

Additionally, an ANCOVA model will be built to test the change from Baseline in FAQLQ scores between treatment groups. The model will include the treatment group, adjusted for the Baseline value and the screening ED subgroup as covariates.

Baseline, Month 12 and change from Baseline of the FAQLQ questionnaires (global score, scores by domain as well as values for each item of the questionnaires) will also be summarized using descriptive statistics by response status (Responder/Non responder), in the ITT population.

Finally, the change in FAQLQ scores according to the change in cumulative reactive dose will be assessed graphically (for the global score and each domain specific score).

FAQLQ data will be listed.

Food Allergy Independent Measure (FAIM)

The FAIM questionnaires capture the subject's expectation of something happening because of her/his food allergy.

Each item of the FAIM is answered on a 7-point scale (0 to 6) and should be recoded 1 to 7.

Total FAIM scores will be calculated by dividing the sum of completed items by the number of completed items. Total FAIM scores range from 1 "low perceived disease severity" to 7 "high perceived disease severity".
Calculation of total FAIM scores will be done only when 80% or more of the items are completed.
Some specific items as mentioned below have to be reversed:

- FAIM-CF: Child Form (8-12 years)
  This questionnaire consists of 6 items. The mean score will be performed if at least 5 items are completed.

- FAIM-PF: Parent Form (Children aged 0-12 years)
  There are 2 sections given in the questionnaire each with the same items :
  - one from the perspective of the parents (What chance do you think your child has of.....?)
  - one reflecting the thoughts of the child (What chance does your child think he/she has of.....?).

Both mean scores of the parent's form ("Parent's thoughts" and "Child's thoughts") are calculated as the mean of the single items, only if none of the items is missing. In both scores item 4 needs to be reverse scored.

Baseline and Month 12 values for each item of the FAIM questionnaires will be summarized overall and by treatment group, in the ITT population.
Baseline, Month 12 and change from Baseline of the FAIM questionnaires (global score, scores by domain) will be summarized overall and by treatment group, in the ITT population.
Baseline, Month 12 and change from Baseline of the FAIM questionnaires (total score, as well as values at each item of the questionnaires) will also be summarized using descriptive statistics by response status (Responder/Non responder), in the ITT population.

FAIM data will be listed.

4.9 Study Drug Safety Evaluation

The safety analysis will be run on the safety population, successively on the following set of subjects:

- For all subjects
- For the subjects within the age range 4 to 5 years: 4 to 6 years old (6 years excluded)
- For the subjects within the age range 6 to 8 years: 6 to 9 years old (9 excluded)
- For the subjects within the age range 9 to 11 years: 9 to 12 years old (12 excluded)
- For each ED screening subgroup

Some safety sub-analyses will be also run on subjects with mutations in the filaggrin gene versus subjects carrying the wild type gene.
Results will be presented by treatment group and overall.
Unless specified no missing data will be replaced/imputed (see Section 4.9.1).
Symptoms due to DBPCFC will be reported in specific pages of the eCRF and not in the AE form, unless the reaction is serious. SAEs due to DBPCFC will be presented separately from AEs (see Section 4.9.10) and will be excluded from all tables and listings summarizing AEs/TEAEs.

4.9.1 Adverse Events

Treatment-emergent AEs will be defined as any AEs, regardless of relationship to study drug reported during or after the initial Viaskin® patch application, i.e.:

- New event, or
- Condition already present that worsens in either severity or relationship to study drug following exposure to Viaskin® patches.

AEs occurring after the end of the study (Visit 11 or Visit 12) will be recorded only if the investigator considers that there is a causal relationship with the study drug and as such, will be considered also as TEAEs.
Pre-treatment emergent AE will be defined as an AE that begins before the first administration of study drug and that does not worsen in severity after the first administration of study drug. During the screening period (from the ICF signature to Day-1), only SAEs are recorded in the database (AEs are only documented in the source documents).
Where dates are missing or partially missing, adverse events will be assumed to be treatment-emergent, unless there is clear evidence (through comparison of partial dates) to suggest that the adverse event started prior to the first dose of study treatment.
More specifically:

- Start date of AE missing: Assumed to be date of first treatment dose.
- Start day and month of AE missing: Assumed to be date of first treatment dose (if same year for both dates), January 1st otherwise.
- Start day of AE missing: Assumed to be date of first treatment dose (if same month and year for both dates), first day of the month otherwise.
- End date for non-ongoing AE missing: Assumed to date of last treatment dose
- End day and month for non-ongoing AE missing: Assumed to be date of last treatment dose (if same year for both dates), December 31st otherwise.

- End day for non-ongoing AE missing: Assumed to be date of last treatment dose (if same month and year for both dates), last day of the month otherwise.

TEAEs with missing severity will be considered as "severe"; TEAEs with missing relationship to the study drug will be considered as "drug-related".
TEAEs reported as related, probably related, or possibly related to the study drug will be considered as related to IP.
Viaskin Peanut-induced Local TEAEs are defined as TEAEs considered as related to IP with a High Level Term equal to "Application and instillation site reactions" and will be flagged in the analysis datasets.
For each summary presented by SOC and PT, the SOC "General disorders and administration site conditions" will be tabulated globally as well as split in 2 subsections:

- General disorders (High Level Term not equal to "Application and instillation site reactions"),
- Administration site conditions (High Level Term equal to "Application and instillation site reactions").

All AEs will be coded using the latest available version of the MedDRA dictionary.
Symptoms related to DBPCFC are reported in tables separately from other AEs.
AEs related to accidental peanut consumption are reported with other AEs.
An overall overview table of AEs (to be distinguished from symptoms/reactions elicited during the DBPCFCs) will be provided showing the number of subjects, the percentage of subjects and the number of events, overall and for each treatment group, for the following categories of AEs:

- Any AE,
- Any serious AEs,
- AEs leading to study treatment discontinuation,
- AEs leading to death,
- Any mild AEs,
- Any moderate AEs,
- Any severe AEs.

Additionally, an overall overview table of TEAEs will be provided showing the number of subjects, the percentage of subjects and the number of events for the following categories of TEAEs:

- Any TEAE,
- Any serious TEAEs,
- Any TEAEs considered related to IP:
  - Any TEAE reported as related,
  - Any TEAE reported as probably related,
  - Any TEAE reported as possibly related,
- Any TEAEs considered unrelated to IP:
  - Any TEAE reported as unlikely related,
  - Any TEAE reported as unrelated,
- Any Serious TEAEs considered related to investigational product (IP),
- Any TEAEs leading to permanent study treatment discontinuation,
- Any TEAEs leading to temporary study treatment discontinuation,
- Any TEAEs leading to death,

- Any mild TEAEs,
- Any moderate TEAEs,
- Any severe TEAEs,
- Any severe TEAEs considered related to IP (reported as related, probably or possibly related),
- Any Viaskin Peanut-induced Local TEAE,
- Any severe Viaskin Peanut-induced Local TEAE,
- Any systemic allergic TEAE considered related to IP (reported as related, probably or possibly related)
- Any TEAE leading to an epinephrine intake
    - Considered related to IP
    - Considered unrelated to IP.

For TEAEs, TEAEs considered related, SAEs, serious TEAEs, serious TEAEs considered related, TEAEs leading to permanent study treatment discontinuation, TEAEs leading to temporary study treatment discontinuation, Viaskin Peanut-induced Local TEAEs and TEAEs leading to death, detailed tables will be created showing the number of subjects who experienced at least one TEAE, the corresponding percentage of subjects and the number of events by SOC and PT.
Adverse events will be ordered by decreasing frequency on the total population by SOC and by PT within the SOC.

Additional tables will be created to display the most frequent TEAEs (by preferred term), occurring in at least 5% of the subjects in any of the treatment groups. Most frequent TEAEs will also be determined on sub-populations defined by class of age and the corresponding table will be displayed.

Additionally, tables showing the number and percentage of subjects who experienced at least one TEAE and the number of events by SOC and PT, summarizing TEAEs leading to:
- Epinephrine use:
    - Standard medication name that contains "EPINEPHRINE"
- Systemic or inhaled corticosteroid use,
    - ATC code starting with "H02"
    - ATC code starting with "A07EA", "C05", "R01" and (ROUTE in "ORAL" "RECTAL" "RESPIRATORY (INHALATION)") or (ROUTE=OTHER with other, specify that contains ("IV" "INTRAVENOUS"))
- Topical corticosteroid use,
    - ATC code starting with "D07"
    - ATC code starting with "A07EA", "C05", "R01" and ROUTE = Topical

will be presented (exhaustiveness of the above selections, especially regarding the route that can be reported as "Other" with a text field specification, will be checked during the blind data review meeting).

These TEAEs will be identified according to the 2 conditions below:

- In the AE form: TEAEs for which a medication is administered ("Were there any medication administered for this adverse event? answered "Yes")
- In the Concomitant medication form for the medication considered: corresponding AE numbers documented in the field "Number of adverse events for which the medication was taken"

For TEAEs and TEAEs considered related to IP, additional tables will be provided showing number of subjects and percentage of subjects:

- By maximum severity (severe, moderate and mild): Subjects will be counted once per SOC and once per PT at the worst severity. If the severity is missing, the worst severity will be assumed.
- By maximum duration. For these tables TEAEs will be presented by SOC, PT within each SOC and maximum duration within each PT (using the classes for duration in days: 1-7, 8-15, 16-30, 31-60, 61-90, above 91). For AEs ongoing at the end of the study, duration will be imputed based on the date of subject end of study. For AEs not ongoing but with missing end date, duration will be imputed based on date of last study treatment.

Additionally, for TEAEs and TEAEs considered related to IP, tables showing number of subjects, percentage of subjects and number of events by severity will be provided. Subjects will be counted once per SOC, per PT and per severity. For these specific tables, if the severity is missing, no hypothesis will be assumed to replace the missing severity.

Adverse event summaries will be ordered in terms of decreasing frequency for SOC, and PT within SOC, in the Viaskin® treatment group, and then similarly by decreasing frequency in the placebo group, and then alphabetically for SOC, and PT within SOC.

The proportions of patches during the one-year exposure period that led to mild, moderate or severe TEAE considered related to IP will be summarized on the Safety population (overall, by screening ED subgroup and by class of age). For this analysis, the worst ongoing severity will be considered for each day of exposure and the following definitions will be used:

- % of patches leading to severe TEAE will be estimated as =

$$100\left(\frac{Number\ of\ days\ with\ ongoing\ severe\ TEAE}{Exposure\ duration}\right)$$

- % of patches leading to moderate TEAE will be estimated as =

$$100\left(\frac{Number\ of\ days\ with\ ongoing\ moderate\ (1)\ TEAE}{Exposure\ duration}\right)$$

(1) without concomitant event of severe intensity

- % of patches leading to mild TEAE will be estimated as =

$$100\left(\frac{Number\ of\ days\ with\ ongoing\ mild\ (2)\ TEAE}{Exposure\ duration}\right)$$

(2) without concomitant event of moderate or severe intensity

The proportion of patches during the one-year exposure period that led to Viaskin® Peanut 250 µg-induced AEs will also be summarized the same way as above.

A by-subject listing of all adverse events will be provided. This listing will be presented by treatment group and will include: treatment group, subject identifier, age, sex, adverse event (SOC, PT, and verbatim term), date/time of onset (and corresponding study day), date/time of resolution (and corresponding study day), duration, severity, treatment required, relationship to study drug, relationship to an accidental peanut consumption and relationship to DBPCFC, action taken with the study drug, outcome, and whether the event is classified as serious or not with the corresponding criteria.
Pre-treatment emergent adverse events will be listed separately the same way as the treatment emergent adverse events.
TEAE leading to an epinephrine intake will also be listed.
Finally, SAEs will also be listed the same way with an additional flag for treatment emergent SAEs.

4.9.2 Deaths

As detailed in the preceding section, a detailed table will be created showing the number of subjects who experienced one SAE leading to Death, the corresponding percentage of subjects and the number of events grouped by SOC and PT.
All deaths will be listed in a summary table.

4.9.3 Local skin reactions as collected in the diary

Number and percentage of subjects with evaluable diary data will be presented.
Local skin reactions at sites of Viaskin® application as assessed by the patient and documented in the diary during the first 6 months of the study treatment will be summarized for itching, redness, swelling, and any local reaction (itching, redness and swelling) using:

- The number and percentage of subjects by maximum severity grade reported (1, 2 or 3) among subjects with evaluable diary data and among subjects with a reaction (grade ≥ 1)
- The number and proportion of days with a reaction reported (based on days with patches graded by type of reaction or for at least one reaction between itching, redness and swelling when reported on any local reaction) among subjects diary data,
- The proportion of days scored by severity grade (worst severity grade per day is considered).
  All proportions will be calculated as

$$\left(\frac{Number\ of\ days\ with\ a\ reaction\ reported}{Number\ of\ days\ with\ patches\ graded}\right)$$

Proportion of days with no assessment of local skin reaction out the number of patches entered in the diary will be provided.

The number of days with a reaction reported and number of days with patches graded will be calculated based on removal dates.

The periods considered for the reporting will be:

- The overall first 6 months (D1 to D180),
- Month 1 (D1 to D30),
- Month 2 (D31 to D60),
- Month 3 (D61 to D90),
- Month 4 (D91 to D120),
- Month 5 (D121 to D150),
- Month 6 (D151 to D180)

The above analysis will be repeated for subjects with filaggrin gene mutations versus wild type subjects.
All local skin reactions reported by the subject will be listed.

4.9.4 Local skin reactions as graded by the investigator at each visit

Local skin reactions at sites of Viaskin® application are graded by the investigator at each visit. More specifically at Visit 4, this evaluation is performed prior to the application and 30 minutes, 1 hour, 2 hours and 3 hours after the administration. These graded reactions will be reported in summary tables showing the number and percentage of subjects:

- By grade (by localization and regardless of the localization) by study visit
- By most severe grade (by localization and regardless of the localization) for the overall treatment period.

The above analysis will be repeated for subjects with filaggrin gene mutations versus wild type subjects.
All local skin reactions as graded by the investigator will also be listed.

4.9.5 Adverse event of special interest (AESI)

Treatment emergent adverse events of special interest (AESI), as defined in the protocol, are:

- Treatment emergent grade 4 local cutaneous reactions or any significant skin lesion which could potentially lead to skin barrier disruption at sites of patch application
  (identified with AETERM which contains "GRADE 4" (or "GRADE IV") and High Level Term equal to "Application and instillation site reactions"),

- Any occurrence of IgE-mediated systemic-type of symptoms distant from the patch application site and considered at least possibly related to the study drug. These systemic allergic TEAEs of interest will be identified through the algorithm of the Anaphylactic Reaction Standardized MedDRA queries (SMQ) (methodology described in the Appendix 5).

All AESI data will be listed.

An overall overview table of systemic allergic TEAEs cases will also be provided showing the number of subjects, the percentage of subjects and the number of cases for the following categories of systemic AESI, using the definition of the protocol (related only) and in addition showing any systemic allergic reaction considered as unrelated to IP:

- Any systemic allergic TEAE cases considered related to IP
  - Reported as Related,
  - Reported as Probably related,
  - Reported as Possibly related,
- Any systemic allergic TEAE cases considered unrelated to IP:
  - Reported as Unlikely related,
  - Reported as Unrelated

All systemic allergic TEAEs cases will be listed.

They will be reported in summary tables showing the number and percentage of subjects affected and the number of underlying AE events by SOC and PT, overall and for subjects with filaggrin gene mutations versus wild type subjects. Summary tables will be split for systemic allergic TEAE cases considered related, systemic allergic TEAE cases considered unrelated and treatment emergent local cutaneous reactions.
AESI will be ordered by decreasing frequency on the total population by SOC and by PT within the SOC.

4.9.6 Clinical Laboratory Evaluation

The following laboratory evaluations will be performed at V1 (D-42), V7 (M3), V8 (M6), V10 (M12), Early termination visit, unscheduled visit (in case of AEs, etc.):

- Hematology: hemoglobin, hematocrit, platelets, red blood cells, white blood cells (neutrophils, lymphocytes, monocytes, eosinophils, basophils in value and %);
- Biochemistry: alanine aminotransferase, aspartate aminotransferase, total bilirubin, total protein, blood urea nitrogen, creatinine;

A urine pregnancy test is performed for female subjects of childbearing potential at site at Baseline and M12.
Clinically significant changes (abnormalities) in laboratory parameters, in the judgment of the Investigator, will be recorded as AEs and appropriate countermeasures taken.
In the event of unexplained abnormal laboratory test values of clinical significance, the tests should be repeated at a reasonable time point and followed up until they have returned to the normal range and/or an adequate explanation of the abnormality is found.
Those repeated assessments performed outside scheduled visits will appear in listings only.

The following results will be tabulated for hematology and biochemistry tests on the safety population:

- Descriptive statistics for each test result at Baseline (V1 /Day -42), Visit 7 (M3), Visit 8 (M6) and Visit 10 (M12).
- Descriptive statistics for changes from Baseline (V1/D-42) for each test result at Visit 7 (M3), Visit 8 (M6) and Visit 10 (M12).
- Common Toxicity Criteria for Adverse Events (CTCAE version 4.03) grade at each time point and shift from Baseline (D-42) for each test result at Visit 7 (M3), Visit 8 (M6) and Visit 10 (M12). CTCAE grades for hematology and biochemistry measures will be derived according to Appendix 3: Common Terminology Criteria for Adverse Events (CTCAE) grades.
- Shift of test abnormalities (Low, Within normal range, High) between Baseline (V1/Day-42) and Visit 7 (M3), Visit 8 (M6) and Visit 10 (M12).

The above descriptions will be repeated on each age range (4 to 5 years, 6 to 8 years, and 9 to 11 years), and each of the screening ED subgroup.

All laboratory data will be listed.
Values that are out of normal range will be flagged in the data listings.
Flags used are:

- Low (below normal ranges), based on the following original flags from the laboratory reports:
    - PL, L2 ("Panic Low")
    - TL, L1 ("Telephone Low")
    - L ("Low")
- High (above normal ranges), based on the following original flags from the laboratory reports:
    - H ("High")
    - TH, H1 ("Telephone High")
    - PH, H2 ("Panic High")

When summarized in tables, all "High" flags will be merged together ("Panic High", "Telephone High"," High"); the same for the low flags as well.
In listings, "High" and "low" flags will be reported.
Pregnancy test data will be reported only as listings.
Listings of all laboratory data will be provided by treatment group, and will include subject identifier, age, sex, weight and visit. Laboratory reference ranges will also be listed.

4.9.7 Vital Signs

The following vital signs will be recorded, before each DBPCFC challenges and at V1 (D-42), V4 (D1), V5 (D8), V6 (M1), V7 (M3), V8 (M6), V10 (M12), End of study, Early termination visit and at any unscheduled visit:

- Systolic blood pressure (in mmHg),
- Diastolic blood pressure (in mmHg),

- Heart rate (beats/minute),
- Respiratory rate (breaths per minute).

The following results will be tabulated for all vital signs:

- Summary statistics of values by visit and changes from Baseline (V4 or V3 –if missing at V4-)
- Number and percentage of subjects with vital signs abnormalities at D-42, Baseline (D1), D8, M1, 3, 6 and 12 for heart rate, systolic blood pressure and diastolic blood pressure classified using the criteria defined below.

Table 3: Criteria to Determine Clinically Relevant Abnormalities in Vital Signs

Vital Sign Criteria for Abnormalities	Criteria for Abnormalities (any of the following situation)
Heart rate	• value <60 beats/min,<br>• value >130 beats/min,<br>• an increase from pre-dosing of >20 beats/min, or<br>• a decrease from pre-dosing of >20 beats/min
Systolic blood pressure	• value <70 mmHg<br>• value >130 mmHg,<br>• an increase from pre-dosing of > 40 mmHg, or<br>• a decrease from pre-dosing of >30 mmHg
Diastolic blood pressure	• value <45 mmHg<br>• value >85 mmHg,<br>• an increase from pre-dosing of >30 mmHg, or<br>• a decrease from pre-dosing of >20 mmHg

Vital signs data and clinically relevant abnormalities in vital signs will also be listed.

4.9.8 Physical Examination

A Physical examination will be performed, before each DBPCFC challenges and at V1 (D-42), V4 (D1), V5 (D8), V6 (M1), V7 (M3), V8 (M6), V9 (M9), V10 (M12), End of study, Early termination visit and at any unscheduled visit at the discretion of the investigator.

Physical examination will include the measure of the weight (kg) and height (cm) and the examination of the following:

- General appearance,
- Head and Neck,
- Ears, nose and throat,
- Eyes,
- Complete skin examination,
- Cardiovascular system,
- Respiratory system,

- Abdominal system,
- Nervous system,
- Other system(s).

The following results will be tabulated:

- Summary statistic for weight, height BMI and BSA by visit and change from Baseline (D1),
- Number and percentage of subjects with abnormal (vs. normal / not done) physical examination by visit for each of the system examined.

Physical examination data will also be listed.

4.9.9 Spirometry and Peak Expiratory Flow Results

For subjects aged 6 years or older (at the date of the visit), spirometry (FEV_1) will be performed at V1 (D-42), V7 (M3), V8 (M6), V9 (M9), V10 (M12), Early Termination Visit and at any unscheduled visit at the discretion of the investigator.
PEF will be performed before each DBPCFC challenge and at V1 (D-42), V4 (Baseline D1), V5 (D8), V6 (M1), V7 (M3), V8 (M6), V9 (M9), V10 (M12), Early termination visit and at any unscheduled visit at the discretion of the investigator.

The following results will be tabulated:

- Summary statistics for FEV1 value (L) and FEV1 percent predicted by visit, and change of value from Baseline (D-42) by visit,
- Summary statistics for PEF value (L) and PEF percent predicted by visit, and change of value from Baseline (D1) by visit.

All FEV_1 and PEF data will be listed.

4.9.10 Symptomatic Reactions during the double-blind placebo controlled food challenge (DBPCFC)

The objective symptoms collected during the DBPCFC are the following:

- Skin:
  - Erythematous rash (and % of rash area concerned)
  - Pruritus
  - Urticaria/ angioedema
  - Rash
- Upper respiratory:
  - Sneezing/ itching
  - Nasal congestion
  - Rhinorrhea
  - Laryngeal

- Lower respiratory:
  - Wheezing
- Gastrointestinal:
  - Diarrhea
  - Vomiting
- Cardiovascular
- Eyes:
  - Conjunctivitis
- Any other objective symptoms (not reported, mild, moderate, severe)

The subjective symptoms collected are:

- Itchy mouth
- Itchy throat
- Nausea
- Abdominal pain
- Any other subjective symptoms (not reported, mild, moderate, severe).

With the exception of erythematous rash (recorded as Yes/No), each symptom is graded as: 0=" absent", 1=" mild", 2=" moderate" or 3=" severe". For erythematous rash, the % area involved is collected. The grading corresponding to the area involved is captured in the grading of the "Rash" symptom. No further step is required.

A total symptom score for each subject will be calculated as the sum of all severity score for all objective symptoms (for the Peanut formula only, excluding erythematous rash and subjective complaints). This will be based on the sum of each of the following symptom being graded for severity as 0, 1, 2 or 3 (respectively absent, mild, moderate and severe):
Pruritus, Urticaria/ angioedema, Rash, Sneezing/ itching, Nasal congestion, Rhinorrhea, Laryngeal, Wheezing, Diarrhea, Vomiting, Cardiovascular, Conjunctivitis, Any other objective symptoms (one score for each of the other objective symptoms, 0 if no other objective symptom is recorded or if the answer to the question "Did the subject experienced any objective symptoms" is "No").

The reactions appearing during a DBPCFC (as they are expressly provoked) will be differentiated from those AEs occurring outside of the DBPCFC. They will be summarized group showing:

- The number and percentage of subjects with objective symptoms (by severity) at Baseline and at M12 (actual value) (overall and by type of symptom)
- The number and percentage of subjects with subjective symptoms (by severity) at Baseline and at M12 (overall and by type of symptom)
- Summary statistics on the Severity score at Baseline, at M12 (actual value) and the change from Baseline to M12 (using observed values) (excluding erythematous rash and subjective complaints).

All SAEs elicited during the entry/screening DBPCFC and Month 12 DBPCFC will be tabulated by SOC and PT and will also be listed.

4.9.11 Data and Safety Monitoring Board

A Data and Safety Monitoring Board (DSMB) composed of experts in food allergy and in the methodology of clinical studies will review study safety data at specific intervals during the study and on an *ad hoc* basis. A specific DSMB meeting will be held when the first 15 subjects 4 to 5 years of age have been randomized and treated for at least 4 weeks (that is, have completed the Month 1 visit). This is to assess very early in the study that the safety of Viaskin® Peanut is acceptable in the youngest subjects. A specific SAP for DSMB meeting was prepared in addition to the present document.

4.10 Exploratory Analyses

There will be no adjustments for multiplicity for any of the following exploratory analyses.
Results will be presented by treatment group.

4.10.1 Treatment responders at Month 12 according to demographic and medical history subgroups

Response rates by treatment groups and treatment effect will be provided on the following subgroups:

- Region (North America, Europe, Australia)
- Gender (Male/Female)
- Race (White/Black or African American/Asian/Other)
- Subjects with / without history of asthma
- Subjects with / without history of allergy other than peanut
- Subjects with / without history of atopic dermatitis or eczema.

Analysis will be performed only on subgroups containing at least 15 subjects.
According to the sample size of each subgroup, exact 2-sided 95% CIs would be provided instead of a 2-sided Wald 95% CIs.
A forest plot will be provided summarizing the primary efficacy variable in the above subgroups.

4.10.2 Analysis over time of IgE and IgG4 specific to peanut components

IgE and IgG4 specific to peanut components will include at least Ara h 1, Ara h 2, Ara h 3, Ara h 8 and Ara h 9.
The following variables will be tabulated, on the overall ITT population, by treatment group using descriptive statistics:

- IgE and IgG4 specific to peanut components at Baseline, M3, M6 and M12
- Change from Baseline in IgE and IgG4 specific to peanut components at M3, M6 and M12
- Relative change from Baseline in IgE and IgG4 specific to peanut components at M3, M6 and M12 calculated as:
    - (Value at Month M minus Value at Baseline) / Value at Baseline

Repeated-measures ANCOVA models will be built to compare the mean absolute change from Baseline value IgE and IgG4 specific to peanut components using all time points evaluated up to M12 in the active Viaskin® Peanut 250 µg group versus the placebo group on the ITT population using observed data. The default variance/ covariance structure is "unstructured".
The analysis performed will be the same as the one described in 4.8.4.1.

4.10.3 Enumeration and characterization of reactions triggered by accidental consumption of peanut during the study and analysis of Risk taking behavior of subjects (voluntary consumption of peanut)

The following results will be reported on the Safety population for each treatment group overall and by screening ED subgroup:

- Number and percentage of subjects with any (at least one) peanut consumption and number of consumptions (in classes: 1, 2,..)
- Number and percentage of subjects with any (at least one) confirmed accidental consumption of peanut and number of accidental consumptions (in classes: 1, 2,..)
- Number and percentage of subjects with any (at least one) not confirmed accidental consumption of peanut (assumed to be voluntary) and number of voluntary consumptions (in classes: 1, 2,..)
- Number and percentage of subjects with induced allergic reaction (overall and by type of symptom as classified by SOC and PT) (separately for confirmed and not confirmed accidental consumption)
- Number and percentage of subjects with treatment taken following accidental peanut consumption (overall and by type of treatment as classified by ATC class (level 3) and preferred drug name)

The above descriptions will be repeated on each age range (4 to 5 years, 6 to 8 years, and 9 to 11 years).
All data related to accidental/voluntary peanut consumption will be listed. These data include: confirmation (or not) of accidental consumption, food consumed, estimated quantity consumed at each occurrence, and associated reactions and severity of reactions. The cumulative reactive dose the subject reached at screening DBPCFC will also be presented in the listing.

All SAEs (allergic reactions) caused by consumption of peanut will be listed.

4.10.4 Epigenetic modifications of the promoters of specific genes

Descriptive analyses of epigenetic modifications of specific genes in both treatment groups will be performed using actual values, change from baseline and relative change from baseline, for the overall ITT population, using observed data. Please Note that Epigenetic data will not be available at time of database lock.

4.10.5 Safety sub-analysis in subjects with mutations in the filaggrin gene versus wild type subjects

The following safety sub-analyses (on the safety population) will be performed for the subjects with mutations in the filaggrin gene versus subjects carrying the wild type gene:

- Analysis of treatment-emergent adverse events: overview table of TEAE, frequency table of TEAEs, TEAEs considered related to IP and AESI by SOC and PT (as described in Section 4.9.1),
- Analysis of local skin reactions collected in the diary (as described in Section 4.9.3),
- Analysis of local skin reactions as graded by the investigator (as described in Section 4.9.4),
- Sensitization status to some other allergens (as described in Section 4.10.6),
- SCORAD evolution over time (as described in Section 4.10.7).

4.10.6 Sensitization status to other allergens and their evolution over the study period

Observed data on allergen specific IgE to cow milk, egg white, pteronyssinus (house dust mites), and Timothy (grass pollen) will be reported using descriptive statistics on:

- Value at Baseline and M12,
- Change and relative change from Baseline at M12.

Shift tables presenting number and percentage of subjects with a value $\geq$ or $<$ 0.7 kU/L at baseline and post-baseline assessments will be provided.
Additionally, shift tables will be presented using the categories (quartiles) as defined below:

- Minimum value to Q1
- Q1 (excluded) to median
- Median (excluded) to Q3
- Q3 (excluded) to maximum value.

A "missing" category will be added at M12 only. Results will be expressed on subjects with a non-missing value at Baseline.
This will be reported for the ITT population and repeated for subjects with filaggrin gene mutations versus wild type subjects.

4.10.7 SCORAD evolution over time

SCORAD total score will be collected at Baseline, M3, M6 and M12.
SCORAD total score will be imputed as 0 if the subject answer "No" to the question: *"Did subject have any areas of atopic dermatitis ?"*Summary statistics will be presented for:

- Value at Baseline, M3, M6 and M12 (actual values),
- Change from Baseline at M3, M6 and M12 (actual values).

This will be reported for the ITT population and repeated for subjects with filaggrin gene mutations versus wild type subjects.

4.11 Patch adhesion

The Viaskin® patch adhesion will be evaluated during 28 days between M3 and M6 (whenever possible, these should be consecutive days - this assessment period can be extended up to M9 in case it has not been completed between M3 and M6).
The scoring system for adhesion and occlusion of Viaskin® patches (assessed at patch application and at patch removal) is indicated as follows:

- 0 = no lift off or detachment of the dressing edges and the occlusion chamber of the patch is intact.
- 1 = some edges of the dressing have lifted off the skin with no impact on the occlusion chamber of the patch which remains fully adherent to the skin.
- 2 = the occlusion chamber of the patch is partially or totally detached.
- 3 = the patch has fallen off the skin.

Even on those days when the patch has deliberately been removed prematurely by the subject or parents/guardians, the photos and assessment should be made as recommended, even if the assessment will not be used in the analysis. That is why the assessments may extend beyond 28 days to ensure that 28 days of usable evaluations of 24 hours of application are recorded.
Assessment of the adhesion of each patch will be made at 2 time points by the parents/guardians for 28 days:

- Immediately after the application of a patch to assess that the patch was applied as recommended and adhered well to the skin (including the edges of the dressing); and
- At the end of the 24-hours application period at time of removal of the patch.

At the time of each patch removal, the subjects (parents/guardians) will also be asked:

- To specify the status at removal of the patch (removal at 24±4 hours, deliberate removal before 24±4 hours, accidental removal),
- To specify the suspected cause of adhesion issue (if any: scratching, swimming, sweating…),
- To grade the ease of patch removal (very easy/ easy / difficult / very difficult),
- To grade whether the removal of the patch was painful (very painful / painful / slightly painful / not painful),

- To specify whether the patch removal caused skin injury (yes / no).

To guarantee the reliability of the adhesion scoring done by the subject's parents/guardians, the Investigator will review the parents/guardians' assessment of the patch adhesion based on the photographs made by the parents/guardians during these 28 days.
If the parents/guardians did not or could not assess the patch adhesion with sufficient reliability according to the Investigator's judgment and/or if the parents/guardians did not or could not take photos for a total of 28 days during M3 and M6, the Investigator can disqualify all or part of the data collected for the adhesion assessment and will re-train the parents/guardians so that the assessment of the patch adhesion can be repeated between M6 and M9 either totally for 28 evaluable days or partially to compensate for the disqualified days of the first period of assessment.
The assessment of adhesion of the Viaskin® patch will also be made at each site visit by site staff trained specifically for this purpose. The duration of application of the assessed Viaskin® patch must be noted, photographs must be taken and the assessment by the site staff will use the same scoring system as above.
A Per-Protocol Adhesion Set will include all evaluations except patches removed by the investigator (intentionally removed early, for example due to intolerable irritation) (i.e. : the investigator answered "No" to the question "do you confirm that the subject's evaluation is valid for the adhesion analysis?") . A listing of evaluations which will not be included in the PP Adhesion set will be provided.
The following tabulations will be provided:

- The number and percentage of patches evaluated overall and by adhesion grade, at time of application and at time of removal,
- The number and percentage of patches evaluated with an adhesion grade 2 or 3, at time of removal,
- The number and percentage of subjects with at least one patch evaluated with an adhesion grade 2 or 3, at time of removal,
- The number and percentage of patches by subject evaluated with an adhesion grade 2 or 3, at time of removal,
- The duration of application for patches with an adhesion grade 2 or 3 and for patches with an adhesion grade of 3,
- The suspected reason for adhesion issue (scratching / swimming / sweating / other/ unknown),
- The presence of skin injury due to removal (yes / no),
- The ease of removal (number and percentage of patches evaluated by category - very easy / easy / difficult / very difficult),
- The pain at removal (number and percentage of patches evaluated by category - very painful / painful / slightly painful / not painful).

Patch adhesion will be considered as acceptable if more than 90% of the patches at the expected time of patch removal, i.e. at 24±4 hours after patch application, have an adhesion score ≤1 as assessed by the subjects' parents/guardians. In other words, adhesion of the patch will be considered as acceptable if less than 10% of the patches are

evaluated with an adhesion grade 2 or 3 at time of removal, during the period of adhesion, for the overall population.
In addition, the population of subjects with more than 10% of their patches evaluated as a grade 2 or 3 will be studied and suspected reason(s) for adhesion issues for the concerned subjects will be tabulated. Response rate (primary efficacy variable) by treatment group and overall will also be presented descriptively on this population

The use of hypoallergenic adhesive dressing (e.g. Tegaderm®) to prevent possible patch adhesion issues was authorized on Dec 2, 2016 (at the time the last subject reached 4 months of treatment) and is collected in the eCRF (investigator assessment at study visits). Following a comment from the FDA, this decision was cancelled on Feb 2, 2017. As some subjects may have already used a hypoallergenic adhesive dressing, the number and percentage of subjects using at least one hypoallergenic adhesive dressing will be evaluated on the following periods:

- Between Month 3 (V7) and Month 6 (V8),
- Between Month 6 (V8) and Month 9 (V9),
- Between Month 9 (V9) and Month 12 (V10),
- Whole study period.

The quantity of dressing used (absolute and relative – compared to the total number of patches applied collected in the diary) with a hypoallergenic adhesive dressing used will also be tabulated.

For the adhesion assessment performed by the investigator at each visit, the following description will be tabulated:

- Number of subjects and percentage of subjects for each adhesion score at each time point.

4.12 Determination of Sample Size

The sample size is calculated based on the following assumptions:

- A 40% response rate for the active Viaskin® Peanut 250 µg group and 10% response rate for the placebo group, both estimated under "Missing=failure" method (based on a maximum drop-out rate of 15%) in the overall population;
- A lower bound of the 95% CI of the difference between active treatment and placebo response rates ≥15% in the overall population and >0% in each screening ED stratum;
- A 60% response rate for the active Viaskin® Peanut 250 µg group and 10% response rate for the placebo group in screening ED Stratum 1, both estimated under "Missing=failure" method (based on a maximum drop-out rate of 15%);
- A 30% response rate for the active Viaskin® Peanut 250 µg group and 10% response rate for the placebo group in screening ED Stratum 2, both estimated under "Missing=failure" method (based on a maximum drop-out rate of 15%);
- The following expected distribution of the screening ED strata:
  - 1/3 of children having a screening ED from 1 mg to 10 mg;

- 2/3 of children having a screening ED from 30 mg to 300 mg.

- A ratio 2:1 to maximize the number of subjects treated in the active Viaskin® Peanut 250 µg group.

Therefore, the overall sample size calculated is approximately 330 subjects evaluable for the primary efficacy endpoint in the analysis (220 subjects in the active Viaskin® Peanut 250 µg group and 110 subjects in the placebo group). Hence, according to the expected distribution of the screening ED strata, approximately 110 children having a screening ED from 1 mg to 10 mg and 220 children having a screening ED from 30 mg to 300 mg should be randomized.
This number of subjects leads to a power of >90% in the overall population, >99% in the Stratum 1 and >95% in the Stratum 2.
This is summarized in the following Table.

Summary of Sample Size Calculations

Population	Lower bound of the 95% CI of the difference	Power	Sample size (N=N1+N2) Expected Response Rate		
			Viaskin® Peanut 250 µg	Placebo	Total
Overall population	≥15%	90%	N1=220 40%	N2=110 10%	N=330
Screening ED Stratum 1	>0%	99%	N1=73 60%	N2=37 10%	N=110
Screening ED Stratum 2	>0%	95%	N1=146 30%	N2=74 10%	N=220

Abbreviations: CI = Confidence interval; ED = Eliciting dose; N = Number of subjects.

Among the 330 subjects randomized, a minimum of 25 subjects should be randomized for each of the following ages: 4 years, 5 years and 11 years, respectively. Additionally, a minimum number of 75 children of either 4 or 5 years of age should be randomized.
The distribution per ED stratum should lead to approximately 110 subjects with a screening ED from 1 mg to 10 mg and approximately 220 subjects with a screening ED from 30 mg to 300 mg, provided that the same distribution as obtained in the Phase IIb VIPES study is reproduced. If this expected distribution is not fully respected during the recruitment in the PEPITES study, a minimum of 50 subjects would need to be included in Stratum 1 in order to guarantee a power of >95% in this stratum and at least 220 subjects would need to be included in Stratum 2 in order to guarantee a power of >95% in this stratum.
From experience, the screen failure rate could be as high as 30%; hence, up to 470 peanut allergic subjects might need to be screened.

4.13 Changes in the Conduct of the Study or Analysis planned in protocol

- To handle cases of randomization stratum error (i.e. the stratum recorded in the IWRS differs from the actual one documented in the eCRF), screening ED

subgroups were considered for secondary analyses using actual screening ED values (i.e. subjects will be considered in the screening ED subgroup corresponding to their actual screening ED value).

- Following FDA comment,, the primary measure of treatment effect will be the difference in response rates between active and placebo treatment groups. The primary analysis will apply a Wald test at a 2-sided 5% significance level to evaluate a null hypothesis of no difference, and a 2-sided Newcombe 95% confidence interval (CI) for the difference in response rates will be calculated. The clinical relevance criterion for the primary analysis will be defined by a ≥15% lower confidence bound of the Newcombe CI, and this condition will determine whether the primary objective has been successfully met, as recommended by FDA.
  The Wald test p-value will be calculated using the SAS® GENMOD procedure, with DIST=binomial and LINK=identity option of the MODEL statement.
  The 2-sided Wilson 95% CIs of individual response rate will be presented by treatment group using the SAS® FREQ procedure.

- Per protocol, descriptions by age group (4-5, 6-11 years of age) are planned for treatment response rate. Descriptions by age group (4-5, 6-11 years of age) will also be provided for Cumulative reactive dose, Percentage of subjects responsive/unresponsive to a cumulative dose of ≥1,444 mg peanut protein and Percentage of subjects unresponsive to the highest cumulative dose of peanut protein.

- IgE and IgG4 values at M12 were planned to be analyzed using an ANCOVA model. IgE and IgG4 results will be analyzed using a repeated-measures ANCOVA model taking into account all time points up to M12 to compare the mean absolute change from Baseline in the Viaskin 250 µg group vs Placebo.

- SPT description will be performed in the overall population, as well as for each screening ED subgroup.

- FAQLQ questionnaires (Child form and Parent form) will be analyzed using a global score (as described in the protocol) and, additionally, by domain and by item.

- The region effect will be explored for primary efficacy endpoint only.
  As sensitivity analysis of primary analysis:
  - On the ITT population, using missing=failure imputation for missing data, Cochran-Mantel-Haenszel (CMH) method with region as stratification variable to compute a Newcombe 95% CI for the region-adjusted difference in response rates.

  Response rates by treatment groups and treatment effect will also be provided descriptively, as exploratory analysis, for each region.

Exploration of region effect for secondary analyses was removed.

- The proportion of days with mild, moderate or severe TEAE occurring during the exposure period will be studied. This additional safety description will be performed on the Safety population.

- Description of risk-taking behavior will be performed on the Safety population (instead of the ITT population).

- The randomization was not stratified by site.

- Methodologies to impute missing data at Month 12 for primary analysis and secondary analysis were revised to address the cases where:
  - the DBPCFC is stopped without the objective symptoms leading to ending the DBPCFC (stopping rules) OR
  - the subject took the 2,000mg dose without any objective symptom leading to ending the DBPCFC.

5 REFERENCES

[1] Flokstra-de Blok BM, DunnGalvin A, Vlieg-Boerstra BJ et al. Development and validation of a self-administered Food Allergy Quality of Life Questionnaire for children. Clin Exp Allergy 2009; 39(1):127-137.

[2] DunnGalvin A, de BlokFlokstra BM, Burks AW, Dubois AE, Hourihane JO. Food allergy QoL questionnaire for children aged 0-12 years: content, construct, and cross-cultural validity. Clin Exp Allergy 2008;38(6):977-986.

[3] O'Kelly M, Ratitch B. Combining Analysis Results from Multiply Imputed Categorical Data. PharmaSUG 2013 - Paper SP03

[4] Little RJ, D'Agostino R, Cohen ML, Dickersin K, Emerson SS, Farrar JT, et al. The prevention and treatment of missing data in clinical trials. N Engl J Med. 2012 Oct 4;367(14):1355-60

[5] Yeonhee K, Seunghyun W. Adjusted proportion difference and confidence interval in stratified randomized trials. PharmaSUG 2013 - Paper SP04

6 APPENDICES

6.1 Appendix 1: Major Protocol deviations

This table provides a sample of attitute taken regarding inclusion or exclusion of subjects with major protocol deviations in the analysis sets. Categories of major protocol deviations will be discussed and finalised during blind data review meetings.

**Major Protocol Deviation**	**Action to be Taken For Analysis**
Subject did not take any study medication	Exclude from all population except ITT
Subject took incorrect study medication	Analyze 'As-randomized' for ITT Analysis. Analyze 'As-treated' for Safety Analysis. Exclude from PP population
Subject failed to meet study inclusion criteria but was entered into the study.	Exclude from PP population if it could have an impact of the Primary efficacy criteria
Subject met study exclusion criteria but was entered into the study.	Exclude from PP population if it could have an impact of the Primary efficacy criteria
Subject met study withdrawal criteria but was not withdrawn.	Exclude from PP population if it could have an impact of the Primary efficacy criteria
Subject non-compliant with study medication regimen (<80% compliant)	Exclude from PP population
Subject non-compliant with study assessment schedule:	
Subjects who do not perform the DBPCFC at 12 months	Exclude from PP population and Full analysis sets.
Visit not within a pre-specified window after baseline visit.	Exclude from PP population if it could have an impact of the Primary efficacy criteria
Subject took prohibited concomitant medication	Exclude from PP population if it could have an impact of the Primary efficacy criteria
Subject is stratified incorrectly	'Actual stratum' will be used to define screening ED subgroup

6.2 Appendix 2: SAS® Code lines

The SAS code lines provided in Appendices 2 are provisional and can be used as a guidance. Further updates to this code lines might be necessary.

Appendix 2.1: Statistical analysis methods and procedures used for the analysis of the primary efficacy endpoint.

The following notations in lower case will be used in the SAS® code lines provided below:

resp	=	Binary variable for treatment response
trt	=	Binary variable for study treatment
sed	=	Binary variable for screening ED stratum
reg	=	Region

Calculating the response rates in each two group and getting the corresponding 2-sided 95% Wilson CIs.
Calculating the difference between active and placebo response rates and getting the corresponding 2-sided 95% Newcombe CI.

```
PROC FREQ;
TABLES RESP /ALPHA=0.05 BINOMIAL(WILSON);
BY TRT ;
run;
PROC FREQ;
   TABLES trt*resp/ ALPHA=0.025 RISKDIFF(SUP MARGIN=0.15
METHOD=NEWCOMBE);
RUN;
```

- Calculating the p-value of the Wald test for the difference between active treatment and placebo response rates

```
PROC GENMOD;
   CLASS trt;
   MODEL resp = trt / DIST=BIN LINK=IDENTITY;
RUN;
```

Sensitivity analyses:

- Cochran-Mantel-Haenszel (CMH) method with Screening ED randomization stratum (Screening ED > 10 mg/Screening ED ≤ 10 mg) as stratification variable to compute a Newcombe 95% CI for the strata-adjusted difference in response rates [5].

```
%stratCI (data=, treatment= trt, strata=sed, response= resp, level=0.05);
```

- Cochran-Mantel-Haenszel (CMH) method with region as stratification variable to compute a Newcombe 95% CI for the region-adjusted difference in response rates [5].

```
%stratCI (data=, treatment= trt, strata=reg, response= resp, level=0.05);
```

Appendix 2.2: Strategy of analysis of the primary efficacy endpoint using multiple imputation of missing data

The following notations in minor case will be used in the SAS® code lines provided below:

resp	=	Binary Variable for treatment response
trt	=	Binary variable for study treatment
sed	=	Binary variable for screening ED stratum

The following steps will be performed along with the specified SAS code [3]:

- Step 1: Running a Multiple imputation of the response rate on 1000 datasets using the SAS® MI procedure. The seed used will be specified in the table output and in the table footnote (process will be rerun using an alternative seed to check for the stability of the results – corresponding results will not be provided in the table outputs but archived as a statistical appendix). A monotone logistic model on the Baseline screening ED stratum will be used to perform the imputations. The multiple imputations will be performed by keeping in the procedure placebo subjects with non-missing data along with any subject with missing data. Thus the probability distribution that imputes the missing response will rely on placebo subjects with a response and so the missing data would mimic placebo response

```
PROC MI NIMPUTE=1000 OUT=<imputed data> SEED=<seed
number=223218> ;
CLASS resp sed;
MONOTONE LOGISTIC (resp=sed/DETAILS) ;
VAR sed resp ; RUN ;
```

- Step 2: Adding to the imputed dataset with 1000 replications for each subject (identified by the variable _IMPUTATION_), a thousand times the subjects under the active treatment that had no missing value for treatment response (subjects that were excluded from the preceding MI procedure)

- Step 3: Calculation of the rate of responders for each treatment group for each imputed dataset using SAS® FREQ procedure and creating a dataset containing estimated response rate and their standard error in each treatment group.

```
PROC FREQ DATA=<imputed data as obtained from step 2>;
   BY _IMPUTATION_ trt ;
   TABLES resp / BINOMIAL(WILSON);
   ODS OUTPUT BINOMIALPROP=< response rate> ;
DATA <response rate and SE>;
MERGE < response rate> (WHERE=(LABEL1="Proportion")
                KEEP=_IMPUTATION_ trt NVALUE1 LABEL1
                RENAME=(NVALUE1=p))
        <exact response rate> (WHERE=(LABEL1="ASE")
                KEEP=_IMPUTATION_ trt NVALUE1 LABEL1
                RENAME=(NVALUE1=se));
BY _IMPUTATION_ trt ;
```

```
RUN;
PROC SORT; BY trt _IMPUTATION_;
RUN;
```

Note:
p = Variable for response rate
se = Variable for standard error for response rate

- Step 4: Calculation of the combined estimate of the response rate in each treatment group using dataset created in step 3.

```
DATA <response rate difference and SE>;
MERGE <response rate and SE> (WHERE=(trt=2) RENAME=(p=P2 se=SE1))
      <response rate and SE> (WHERE=(trt=1) RENAME=(p=P1 se=SE2));
  BY _IMPUTATION_;
  RUN;
PROC MIANALYZE DATA=<response rate and SE from step 3>;
MODELEFFECTS p1 p2;
STDERR se1 se2;
ODS OUTPUT PARAMETERESTIMATES=<combined estimates of response rate>;
RUN;
```

- Step 5: Calculation of the Wilson confidence interval of the combined estimate of the response rate in each treatment group using dataset created in step 3.
- Calculation of the combined estimate of the difference in response rate between the two treatment groups and the Newcombe confidence interval using dataset created in step 3.

```
DATA <combined estimates of response rate Wilson IC trt1> (WHERE=(parm=p1));
SET <combined estimates of response rate>;
quartile_norm=quantile("normal",0.975);
Wilson=1
L1= (Estimate+quartile_norm*quartile_norm/(2*n1)-quartile_norm*sqrt((Estimate*(1-
Estimate)+(quartile_norm*quartile_norm)/(4* n1))/ n1))
/(1+(quartile_norm*quartile_norm)/ n1);
U1=(Estimate+quartile_norm*quartile_norm/(2*n1)+quartile_norm*sqrt((Estimate*(1-
Estimate)+(quartile_norm*quartile_norm)/(4* n1))/ n1))
/(1+(quartile_norm*quartile_norm)/ n1);
RUN;

DATA <combined estimates of response rate Wilson IC trt2> (WHERE=(parm=p2));
SET <combined estimates of response rate>;
quartile_norm=quantile("normal",0.975);
Wilson=1
L2= (Estimate+quartile_norm*quartile_norm/(2*n2)-quartile_norm*sqrt((Estimate*(1-
Estimate)+(quartile_norm*quartile_norm)/(4* n2))/ n2))
/(1+(quartile_norm*quartile_norm)/ n2);
U2= (Estimate+quartile_norm*quartile_norm/(2* n2)+quartile_norm*sqrt((Estimate*(1-
Estimate)+(quartile_norm*quartile_norm)/(4* n2))/ n2))
/(1+(quartile_norm*quartile_norm)/ n2);
RUN;
```

```
DATA <combined estimates of difference in response rate Newcombe IC>
MERGE < combined estimates of response rate Wilson IC trt1 > < combined estimates of
response rate Wilson IC trt2 >
by Wilson;
p_d =p1-p2;
dL=(p1-p2)-sqrt((p1-L1)*(p1-L1)+(U2-p2)*(U2-p2));
dU=(p1-p2)+sqrt((U1-p1)*(U1-p1)+(p2-L2)*(P2-L2));
RUN;
```

Note:

p1	=	Variable for estimated response rate
se1	=	Variable for standard error of estimated response rate
p2	=	Variable for estimated response rate
se2	=	Variable for standard error of estimated response rate
n1	=	N for treatment 1
n2	=	N for treatment 2
p_d	=	Variable name fort estimated difference in response rate between treatment
dL	=	Variable for the lower bound of Newcombe confidence interval
dU	=	Variable for the upper bound of Newcombe confidence interval

Appendix 2.3: Statistical analysis methods and procedures used for the analysis of the cumulative reactive dose of peanut protein at month 12.

The following notations in minor case will be used in the SAS® code lines provided below:

Crd0(12)	=	Variable for Peanut protein cumulative reactive dose at Baseline (M12)
trt	=	Binary variable for study treatment

- The peanut protein cumulative reactive dose in each treatment group at M12 will be compared using an analysis of covariance (ANCOVA) model. The ANCOVA model will include the treatment group, adjusted for the Baseline value.

```
PROC MIXED ;
CLASS trt;
MODEL crd12=trt crd0 / DDFM=KR ;
LSMEANS trt / CL DIFF OM ;
ODS OUTPUT LSMEANS=<least square means>
DIFFS=<least square means differences> ;
RUN ;
```

Appendix 2.4: Statistical analysis methods and procedures used for the analysis over time of peanut-specific IgE and IgG4.

The following notations in minor case will be used in the SAS® code lines provided below:

trt	=	Binary variable for study treatment
x0	=	Peanut specific IgE (IgG4) at Baseline
visit	=	Visit variable<br>1=M3, 2=M6, 3=M12
chx	=	Absolute change in Peanut specific IgE (IgG4 or skin prick test value) at:<br>M3 (for visit=1)<br>M6 (for visit=2)<br>M12 (for visit=3)
sed	=	Binary variable for screening ED subgroup
Id	=	Subject Identification

- The analysis of peanut-specific IgE and IgG4 over time will be performed using a repeated-measure analysis of covariance (ANCOVA). The ANCOVA model will include the treatment group, adjusted for the Baseline value and the screening ED subgroup. The input data set will have one observation by visit (up to 3 by subject).

```
PROC MIXED ;
CLASS visit id trt sed;
MODEL chx=trt x0 sed trt*visit visit;
REPEATED visit / type=UN SUBJECT=id;
LSMEANS visit trt visit * trt  / PDIFF=ALL CL;
RUN
```

6.3 Appendix 3: Common Terminology Criteria for Adverse Events (CTCAE) grades

Haematology	G1	G2	G3	G4
Red blood cells (x10e12 /L)	No grading according to CTCAE			
Hematocrit (V/V)	No grading according to CTCAE			
Hemoglobin (g/L) - low	[100;LLN[	[80;100[	<80	
Hemoglobin (g/L) - high	]ULN;ULN+20]	]ULN+20;ULN+40]	>ULN+40	
Platelets (x10e9 /L) - low	[75;LLN [	[50;75[	[25;50[	<25
Leucocytes (x10e9 /L) - low	[3;LLN[	[2;3[	[1;2[	<1
Neutrophils (x10e9 /L) - low	[1.5;LLN[	[1;1.5[	[0.5;1[	<0.5
Eosinophils (x10e9 /L)	No grading according to CTCAE			
Basophils (x10e9 /L)	No grading according to CTCAE			
Lymphocytes (x10e9 /L) - low	[0.8;LLN[	[0.5;0.8[	[0.2;0.5[	<0.2
Monocytes (x10e9 /L)	No grading according to CTCAE			

Biochemistry	G1	G2	G3	G4
BUN/urea (mmol/L)	No grading according to CTCAE			
Total protein (g/L)	No grading according to CTCAE			
Total bilirubin (µmol/L) – high	]ULN; ULN*1.5]	]ULN*1.5; ULN*3]	]ULN*3;ULN*10]	>ULN*10
AST (U/L) - high	]ULN; ULN*3]	]ULN*3; ULN*5]	]ULN*5;ULN*20]	>ULN*20
ALT (U/L) - high	]ULN; ULN*3]	]ULN*3; ULN*5]	]ULN*5;ULN*20]	>ULN*20
Creatinine (µmol/L) - high	]ULN; ULN*1.5]	]ULN*1.5; ULN*3]	]ULN*3;ULN*6]	>ULN*6

6.4 Appendix 4: List of Post-Text Tables, figures, listings, and Supportive SAS output appendices

Will be provided in a separate document.

6.5 Appendix 5: Methodology of identification of systemic allergic adverse events

This methodology will be based on the MedDRA SMQ "Anaphylactic reaction" (MedDRA version in use at the time of study results analysis) as described in the "Introductory Guide for Standardised MedDRA Queries (SMQs) Version 20.0.

The retrieval will be performed in the SMQ anaphylactic reaction using an algorithmic approach which combines a number of anaphylactic reaction symptoms in order to increase specificity. A case must include either:

- A narrow term or a term from Category A;
- A term from Category B - (Upper Airway/Respiratory) AND a term from Category C - (Angioedema/Urticaria/Pruritus/Flush);
- A term from Category D - (Cardiovascular/Hypotension) AND [a term from Category B - (Upper Airway/Respiratory) OR a term from Category C - (Angioedema/Urticaria/ Pruritus/Flush)]

The events will be considered as concomitant for the algorithm output if they appear on the same day +/- 1 day.

- Related AESI will be determined if at least on term of any association in the algorithm is "related".
- Unrelated AESI will be determined if all terms of any association in the algorithm are not "related".
- Where dates are missing or partially missing, adverse events are not considered for possible association."

Document ID: 384676549 - Name at Publication: PEPITES_SAP_201701004_4.0_signed.pdf
This document contains a total of 80 pages including any signature page/s.

PAREXEL International Electronic Signature Page

This page is the manifestation of the electronic signature(s) used in compliance with PAREXEL International's electronic signature policies and procedures and in compliance with applicable regulations.

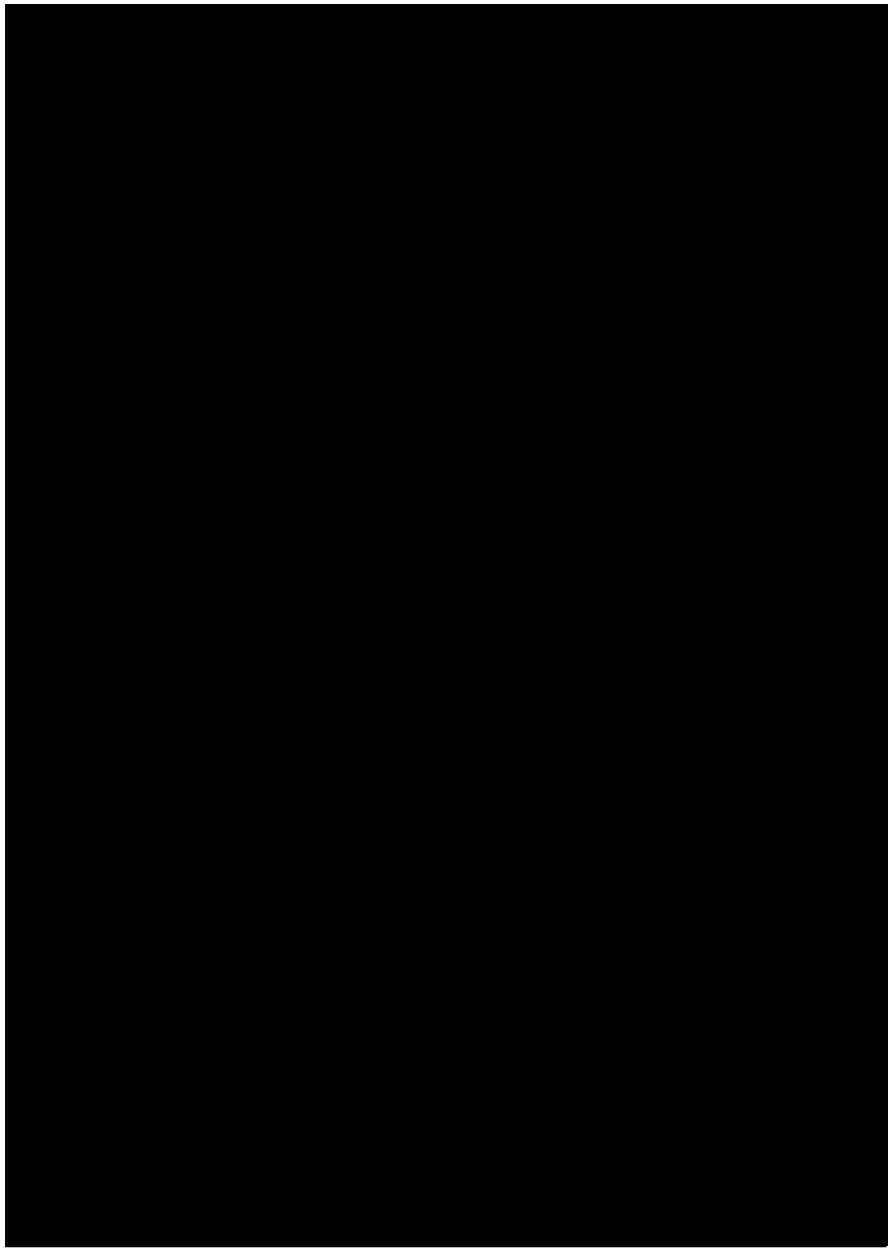

Supplement: Supplement 1b [file mmc2.pdf]
