# Supplementary material for: Efficacy and safety of peanut epicutaneous immunotherapy in patients with atopic comorbidities
Source: J Allergy Clin Immunol Glob. 2022 Sep 22;2(1):69–75. doi: 10.1016/j.jacig.2022.07.009 (PMC10509968; doi:10.1016/j.jacig.2022.07.009)
Supplement: Supplement 2a [file mmc3.pdf]

LONG-TERM ASSESSMENT of SAFETY and THERAPEUTIC BENEFIT of VIASKIN® PEANUT EPICUTANEOUS TREATMENT in PEANUT-ALLERGIC CHILDREN: A 6-MONTH RANDOMIZED, DOUBLE-BLIND, PLACEBO-CONTROLLED PHASE III STUDY FOLLOWED by AN OPEN LABEL ACTIVE TREATMENT (REALISE STUDY)

**Sponsor:**	DBV TECHNOLOGIES S.A.<br>177-181 avenue Pierre Brossolette<br>92120 – Montrouge<br>France<br>Tel: + 33 1 55 42 78 78<br>Fax:+ 33 1 43 26 10 83
**Clinical Research Organization:**	PAREXEL International (IRL) Limited<br>70 Sir John Rogerson's Quay<br>Dublin 2<br>Ireland
**Sponsor Protocol No.:**	**REALISE**<br>(**REAL LI**fe Use and **S**afety of **E**PIT)
**IND No.:**	14366
**Study Drug Name:**	Viaskin® Peanut (DBV712)
**Development Phase:**	III
**Versions and Dates of Protocol:**	Protocol Version 4.0, 9 October 2017 (including Protocol Amendment 3 – 9 October 2017)

The study will be conducted according to the protocol and in compliance with Good Clinical Practice (GCP), with the Declaration of Helsinki and with other applicable regulatory requirements.

This document contains confidential information of DBV Technologies.
Do not copy or distribute without written permission from the Sponsor.
CONFIDENTIAL

SIGNATURE PAGE

Declaration of Sponsor or Responsible Medical Officer

Title: Long-term assessment of safety and therapeutic benefit of VIASKIN® peanut epicutaneous treatment in peanut-allergic children: A 6-month randomized, double-blind, placebo-controlled, phase III study followed by an open label active treatment (REALISE study)

This study protocol was subject to critical review. The information it contains is consistent with current knowledge of the risks and benefits of the investigational product, as well as with the moral, ethical, and scientific principles governing clinical research as set out in the Declaration of Helsinki, [2013] (APPENDIX 1)*, and the guidelines on Good Clinical Practice.*

[redacted]

[redacted]
Date

Declaration of the Investigator

Title: Long-term assessment of safety and therapeutic benefit of VIASKIN® peanut epicutaneous treatment in peanut-allergic children: A 6-month randomized, double-blind, placebo-controlled, phase III study followed by an open label active treatment (REALISE study)

I have read and understood and agree to abide by all the conditions and instructions contained in this protocol.

All documentation for this study that is supplied to me and that has not been previously published will be kept in the strictest confidence. This documentation includes this study protocol, Investigator's Brochure (IB), electronic case report form (e-CRF), and other scientific data.

The study will not be commenced without the prior written approval of a properly constituted Institutional Review Board (IRB) or Independent Ethics Committee (IEC). No changes will be made to the study protocol without the prior written approval of the Sponsor and the IRB or IEC, except where necessary to eliminate an immediate hazard to the subjects.

Responsible Investigator of the local study center

__________________________________ ____________________

Signature Date

____

Name (block letters)

____

Title (block letters)

____

Institution (block letters)

____

Phone number

PROTOCOL SYNOPSIS

**Title**	Long-term assessment of safety and therapeutic benefit of Viaskin® Peanut epicutaneous treatment in peanut-allergic children: A 6-month randomized, double-blind, placebo-controlled, phase III study followed by an open label active treatment (REALISE study)
**Sponsor Study No.**	REALISE
**Phase**	III
**Sponsor**	DBV Technologies SA
**Study Centers**	This is a multicenter, randomized, double-blind study to be conducted in North America (US and Canada). It is planned that approximately 28 to 40 sites could participate.
**Objectives**	• To characterize the safety of Viaskin® Peanut 250 µg in peanut-allergic children of 4-11 years of age over a 36-month treatment period<br>• To explore the therapeutic benefit of a long-term treatment (up to 36 months) with Viaskin® Peanut 250 µg in peanut-allergic children.
**Design**	This is a 36-month study to assess the safety of Viaskin® Peanut, dosed at 250 µg peanut protein (per patch), in peanut-allergic children from 4 to 11 years of age.<br>The study is designed in 2 periods:<br>- A randomized, double-blind, placebo-controlled design for the first 6 months of treatment,<br>- Followed by an open-label, single arm active treatment design with Viaskin® Peanut 250 µg. The duration of this open label period will depend on the initial treatment group of the subjects. After the initial 6-month blinded period, subjects initially randomized in the active Viaskin® Peanut 250 µg group will continue their active treatment for another 30 months. Subjects initially randomized in the placebo group will cross-over to receive the active treatment Viaskin® Peanut 250 µg for 36 months.<br>After selection, the subjects will be randomized with a ratio of 3:1 (active *versus* placebo). The randomization will be stratified by center and age group (children 4 and 5 years of age in a stratum and children 6-11 years in a second stratum).<br>The overall maximum total study duration for each subject ranges from 3 to 3.5 years:<br>- A 2-week screening period,<br>- Subjects initially in the active arm: 36 months of treatment,<br>- Subjects initially in the placebo arm: 42 months of treatment (6 months under placebo + 36 months of active treatment),<br>- A 2-week follow-up period after the end of the treatment.<br>The subjects selected for the study must have a physician-diagnosis of peanut allergy based on a well-documented medical history of IgE-mediated reactions after ingestion of peanut i.e. reactions having led to an emergency department visit or a physician consultation and currently following a strict peanut-free diet. Once selected, the subject will perform several procedures and must at least fulfill the two here mentioned criteria to be eligible for treatment: a peanut Skin Prick Test (SPT) with a largest wheal diameter ≥8 mm <u>and</u> peanut-specific IgE ≥14 kU/L.<br>Key assessments of safety parameters will include adverse events (AEs), skin observation of the patch areas of application (inter-scapular area of the back), spirometry, Peak Expiratory Flow (PEF) measurements, vital signs, physical examinations and laboratory assessments. Atopic dermatitis will also be assessed using the Scoring atopic dermatitis (SCORAD).

Laboratory parameters will include peanut-specific immunoglobulin E (IgE), peanut component-specific IgE, peanut-specific immunoglobulin G4 subtype (IgG4), peanut component-specific IgG4. Skin prick tests will also be performed.

During the whole study duration, any AE will be spontaneously reported by the subjects in a dedicated section in the diaries, as well as any used concomitant medications.

- During the first 6 months, 3 pre-specified symptoms (itching, redness, swelling) will be reported on a daily basis in the diaries. These 3 pre-specified solicited symptoms will not be reported by the Investigator in the AE pages of the electronic case report form (e-CRF), except if these symptoms are part of another concomitant disease or if these symptoms are leading to the subject's study discontinuation or serious AEs. Any other local skin reactions or any other type of adverse events will be spontaneously reported in the diaries and will be reported by the investigators in the AE e-CRF form.
- After month 6, any AE, including the 3 above symptoms, other local skin reactions or any other type of AE, will be spontaneously reported in the dedicated section of the diaries. These AEs will be reported by the Investigator in the e-CRF.

The diaries will be systematically reviewed by the site medical staff at each subject's visit.

Considering the length of this study, and for ensuring the subject's medical management, the investigators may need to perform peanut-food challenges during the course of the study, based on their own medical judgment.

A maximum of 2 open peanut-food challenges for the same subject is recommended during the treatment course up to the 36 months of active treatment. Performing open food challenge(s) in the REALISE study is optional and is left to the investigator's decision. However, no food challenge can occur before the subject has received 12 months of active treatment with Viaskin® Peanut 250 µg.

If the decision is made by the investigators to perform a peanut-food challenge, its performance must comply with the standardized methodology defined in the protocol APPENDIX 6. A standardized peanut food challenge formula and material as well as the Manual of Procedures will be provided to all sites, and must be used for conducting the open peanut food challenge.

If 2 peanut challenges are performed for the same subject within the treatment course, the second challenge can occur only after at least 12 additional months of active treatment have been administered to that subject.

When performed, the results of all food challenge(s) will be documented and reported in the e-CRF.

All subjects will remain on a peanut-free diet for the duration of the study. The re-introduction or not of peanut into the subject's diet at the end of their participation in the study will be left to the Investigator's decision. The Investigator's decision will be collected in the eCRF.

Treatment

The study drug, Viaskin® Peanut 250 µg, is an epicutaneous delivery system (Viaskin® patch) containing a solid deposit of a formulation of peanut protein extract. The drug substance is an unmodified, lyophilized peanut extract produced from the extraction and freeze drying of defatted peanut flour of biological origin, the peanut seed from the Virginia variety of *Arachis hypogaea*.

The placebo Viaskin® patches are similar to the Viaskin® Peanut 250 µg, except that they do not contain peanut protein extract.

The Viaskin® patch must be applied on the intact skin for 24 hours every day, which means that a new patch will be applied every 24 hours (± 4 hours) on the inter-scapular area of the back of the subjects.

To increase the safety of the Viaskin® patch at the initiation of treatment, the application duration of the Viaskin® patch will be progressively increased to a duration of 24 hours daily over a 15-day graduated dosing period as follows:

- During the first week (from Day 1 through Day 7), the patches will be applied for 6 hours (± 1 hour) every day;
- During the second week (from Day 8 through Day 14), the patches will be applied for 12 hours (± 2 hours) every day;
- From the third week onwards (Day 15), the patches will be applied for the entire 24 hours (± 4 hours) daily.

At the start of the study, all subjects, whatever their groups of treatment, will follow the progressive increase of daily duration of patch application described above.

From the month 6 Visit, all the subjects will receive the active treatment Viaskin® Peanut 250 µg in an open label manner. However, the randomization code will not be unblinded at that time. To ensure that the placebo subjects switching to receive the active treatment will start their treatment safely, all subjects will repeat the progressive increase of daily duration of the Viaskin® Peanut 250 µg patches application as at the start of the study.

All the subjects will then be treated with Viaskin® Peanut 250 µg for either 30 or 36 additional months, for subjects randomized in the active or placebo groups respectively. The randomization code will be unblinded and the treatment groups assigned during the first 6 months will only be communicated to the investigators and the subjects after the 6-month database lock.

Number of Subjects

It is planned to randomize 335 subjects in this study. Based on a randomization ratio 3:1, it is anticipated to have approximately 250 subjects in the active arm and 85 subjects in the placebo arm. Based on an anticipated screen failure rate of 30%, it is anticipated that approximately 480 subjects will need to be screened.

Assuming a drop-out rate of 15% per year, this will ensure that approximately 284 subjects will complete the first year of treatment, 242 subjects will complete the study up to the end of year-2 and 205 subjects will complete the study up to the end of year-3.

To ensure an adequate and sufficient representation of the youngest subjects 4 and 5 years of age, a minimum of 90 subjects of these ages must be randomized in the active arm. With the randomization ratio 3:1, this means that a minimum of 120 subjects (36%) 4-5 years of age must be randomized. Hence, the study will also be stratified by age.

The two strata of the study will comprise:

- Stratum 1 composed of a minimum of 120 subjects 4-5 years of age;
- Stratum 2 composed of approximately 215 subjects 6-11 years of age.

Population

Subjects will be enrolled in this study only if they meet, among others, the following <u>key inclusion criteria</u>:

- Male or female children aged 4 through 11 years at Visit 1;
- Physician-diagnosis of peanut allergy based on a well-documented medical history of IgE-mediated reactions after ingestion of peanut, i.e. allergic reaction(s), having led to an emergency department visit or a physician consultation,
- A peanut SPT with a wheal largest diameter ≥8 mm;

- A specific-peanut IgE ≥14 kU/L;
- Subjects following a strict peanut-free diet.

Peanut allergic subjects presenting a medical history of severe anaphylaxis to peanut will be considered for this study. Severe anaphylaxis is defined by the Grade 3 of the Anaphylaxis Staging System (APPENDIX 5) including:

- Severe hypoxia, hypotension (more than 20% drop in blood pressure) or neurological compromise, or
- Cyanosis or SpO2 ≤ 92% at any stage, confusion, cardiovascular collapse, loss of consciousness, incontinence, bradychardia, cardiac arrest.

Primary Criteria: Evaluation of Study Drug Safety

The following safety criteria will be evaluated:

- AEs and Treatment-Emergent Adverse Events (TEAEs) by System Organ Class (SOC) and Preferred Terms (PTs);
- TEAEs by maximum severity, duration and relatedness to treatment patch, placebo or active;
- TEAEs leading to discontinuation;
- Incidence, duration and maximum severity of local cutaneous Viaskin® patch-induced reactions as assessed by the subject;
- Severity of local cutaneous Viaskin® patch-induced AEs as assessed by the Investigator;
- Adverse Events of Special Interest (AESI) including Grade 4 local cutaneous reactions and systemic allergic AEs considered related to Viaskin® patch;
- Serious Adverse Events (SAEs) by SOC and PTs, and relatedness to Viaskin® patch;
- Laboratory data, physical examinations and vital signs;
- Spirometry results or PEF results;
- Safety sub-analysis in subjects with mutations in the filaggrin gene *versus* wild type subjects.

During the first 6 months, corresponding to the blinded period of treatment, all safety parameters will be presented by treatment group, i.e. Viaskin® Peanut 250 µg *versus* Placebo.

After 6 months, subjects in the placebo arm will cross-over to the active, Viaskin® Peanut 250 µg treatment during the open-label follow-up period.

The safety parameters will be first studied separately in two Safety sub-Population groups, i.e. the Safety sub-Population without history of Severe Anaphylaxis (SPwoSA) and the Safety sub-Population with history of Severe Anaphylaxis (SPwSA). If no difference in local tolerability and safety is observed in these 2 sub-population groups (as determined by the Data and Safety Monitoring Board, DSMB), then they will be pooled in an overall Safety population and the analysis will be repeated. The results for the age ranges 4 to 5 years, 6 to 8 years and 9 to 11 years will be presented.

The DSMB, composed of independent experts in food allergy and in the methodology of clinical studies, will review the study safety data at specific intervals during the study or on an *ad hoc* basis.

Exploratory Criteria:

The following exploratory criteria will be evaluated:

- Peanut-specific IgE and IgG4 over time;
- Peanut component-specific IgE and IgG4 over time;
- Peanut SPT mean wheal diameters over time;

- Description of the quality of life questionnaires (Food Allergy Quality of Life Questionnaire [FAQLQ]/Food Allergy Independent Measure [FAIM]) and change from baseline in FAQLQ scores (global score and score by domain) over time;
- Description of reactions triggered by accidental consumption of peanut during the study and analysis of "risk-taking behavior" of subjects (voluntary peanut consumption) during the study;
- Epigenetic modifications of the promoters of specific genes;
- Sensitization status to other allergens: evolution of their specific IgE over the study period;
- SCORAD evolution over time;
- Eliciting and cumulative doses reported for any peanut-food challenge(s) performed during the study;
- Symptoms reported during peanut-food challenge(s) performed during the study.

Statistical Methods

Analysis sets:

Randomized Population:

The Randomized population will be comprised of all subjects who are randomized in the study.

Safety population:

The overall Safety population will be comprised of all subjects who are randomized and have used at least 1 patch of the study drug.

Peanut allergic subjects presenting a medical history of severe anaphylaxis to peanut will be specifically identified among the Safety population, resulting in the following 2 sub-populations:

- Safety Sub-Population of subjects **Without** a history of severe anaphylaxis to peanut (SPwoSA),
- Safety Sub-Population of subjects **With** a history of severe anaphylaxis to peanut (SPwSA).

If the safety profile of these two sub-populations are similar, both sub-populations will be merged and safety analyses will be repeated on the overall Safety population.

Per-protocol (PP) population:

The PP population will include all subjects from the Safety population who do not have major deviations from the protocol that may affect the safety evaluation. The PP population will be used to perform confirmatory analyses. In case the 2 Safety sub-populations are not equivalent, PP sub-populations will be defined as follows:

- PP sub-population **Without** a history of severe anaphylaxis to peanut (PPwoSA),
- PP sub-population **With** a history of severe anaphylaxis to peanut (PPwSA).

Statistical methods:

Categorical variables will be summarized using the number of observations and percentages. The denominator for percentages will be the number of subjects in the population with data available unless otherwise stated. Continuous variables will be summarized using descriptive statistics (number of observations [n], mean, standard deviation, minimum, first quartile [Q1], median, third quartile [Q3], and maximum).

Safety endpoints will first be evaluated on the Safety Sub-Population of subjects without a history of severe anaphylaxis to peanut (SPwoSA) and then on the Safety Sub-Population of subjects with history of Severe Anaphylaxis (SPwSA).

Safety analyses will be performed when all subjects reach Visit 6 (6 months of blinded treatment on either Viaskin® Peanut 250 µg or Placebo).

Further Safety analyses will also be performed:

- After all subjects have received 12 months of treatment, i.e. all subjects have reached Visit 9,
- After all subjects have received 12 months of active treatment, i.e. subjects from the initial placebo group have reached Visit 11,
- After all subjects have received 24 months of active treatment, i.e. subjects from the initial placebo group have reached Visit 13,
- After all subjects have received 36 months of active treatment, i.e. subjects from the initial placebo group have reached Visit 15P.

Below are the rescheduled visits based on the duration of the active treatment received:

Time point:	D1	M1	M3	M6	M9	M12	M18	M24	M30	M36
Initial group: active	V2	V4	V5	V6	V8	V9	V11	V12	V13	V14
Initial group: placebo	V6	V7	V8	V9	V10	V11	V12	V13	V14	V15P

All assessments until Month 6 will be presented by treatment group and tables will display "Viaskin® 250 µg" group *versus* "Placebo" group. Assessments from Month 6 to the end of study will be analyzed using the rescheduling visits described above and tables will show the "Viaskin® 250 µg" group, the "Rescheduled Placebo" group, and both groups pooled together.

Safety analysis:

Adverse Events

TEAEs will be defined as any AEs, regardless of relationship to study drug, which occur during or after the initial Viaskin® patch application or any event already present that worsens in either severity or relationship to Viaskin® Peanut 250 µg or placebo.

All AEs will be reported by SOC and PT and coded using the latest available version of the MedDRA dictionary.

An overall summary of TEAEs will be provided showing the number and percentage of subjects with any TEAEs, any potentially drug-related TEAEs, any severe TEAEs, any severe potentially drug-related TEAEs, any serious TEAEs, any serious potentially drug-related TEAEs, any TEAEs leading to study treatment discontinuation, and any TEAEs leading to death. The number of events will also be presented.

The number of TEAEs, as well as the number and percentage of subjects who experienced at least one TEAE will be summarized by SOC and PT.

The incidence of the following events will also be summarized:

- TEAEs by maximum severity, by duration and by relatedness to Viaskin® patch;
- Local skin reactions at sites of Viaskin® patch application as assessed by the subjects (incidence, duration and severity, 0 to 3 grading scale);
- Local skin reactions at sites of Viaskin® patch application as assessed by the Investigator (severity, 0 to 4 grading scale at each time point);
- SAEs, serious TEAEs and serious potentially drug-related TEAEs;
- Potentially drug-related TEAEs;
- TEAEs leading to Study Treatment discontinuation;
- AESI (including systemic allergic reactions considered related to Viaskin® Patch and Grade 4 local cutaneous reactions at patch application sites).

The above criteria will be studied in the Safety Sub-population of subjects without a history of severe anaphylaxis to peanut and will be also studied in the Safety Sub-population of subjects with a history of severe anaphylaxis to peanut (if less than 15

are included in this sub-population, listings will be edited). If the 2 sub-populations are similar safety-wise (as determined by the DSMB), safety endpoints will be repeated in the overall Safety population. Safety analyses will be repeated for each of the age ranges 4 to 5 years, 6 to 8 years and 9 to 11 years. A safety sub-analysis will be performed in subjects with mutations in the filaggrin gene *versus* wild type subjects.

All AEs will be listed.

The AEs induced by any food challenge performed during the study for the subject medical management, because they are expressly provoked, will be differentiated from those AEs occurring outside of challenges. Objective and subjective symptoms/reactions elicited during challenges will be summarized separately:

- Symptoms elicited during a peanut-food challenge by severity;
- SAEs elicited during a peanut-food challenge.

Laboratory Assessments

Descriptive statistics will be calculated for all laboratory tests (hematology and biochemistry) performed at Visit 1 (-2 or -1 w), Visit 5 (Month 3), Visit 6 (Month 6), Visit 8 (Month 9), Visit 9 (Month 12), Visit 11 (Month 18), Visit 12 (Month 24), Visit 13 (Month 30), Visit 14 (Month 36) for all subjects and Visit 15P (Month 42) for subjects initially in the placebo group. Categorical variables will be summarized by frequency and percentages of subjects in corresponding categories.

Changes in laboratory data from baseline will also be presented.

In addition, summaries of laboratory values categorized based on Common Toxicity Criteria for Adverse Events (CTCAE) grade will also be presented.

Shift tables of test abnormalities will be generated to compare baseline values to the values collected at other time points.

Laboratory data will be analyzed overall and for the age ranges 4 to 5 years, 6 to 8 years and 9 to 11 years.

All laboratory data will be listed. Listing of values that are out of normal ranges will be flagged in the data listings.

Vital Signs

Observed vital signs values and changes from baseline will be descriptively summarized by visit. All vital signs data will be listed.

The analysis of vital signs will focus on the incidence of clinically relevant abnormalities. The number of subjects evaluated and the number and percentage of subjects with clinically relevant post-baseline abnormalities at each visit will be presented.

Vital signs will be analyzed overall and for the age ranges 4 to 5 years, 6 to 8 years and 9 to 11 years.

Physical Examination

Physical examination data will be summarized and listed by visit.

Changes in physical examination data from baseline will be presented. Physical examination data will be analyzed overall and for the age ranges 4 to 5 years, 6 to 8 years and 9 to 11 years.

Skin reactions observed during the physical examinations will also be reported and the corresponding data will be tabulated.

Spirometry and Peak Expiratory Flow Results

Percent predicted values for Forced expiratory volume in 1 second (FEV1) and PEF and changes from baseline will be descriptively summarized separately by visit. All FEV1 and PEF data will be listed. The performed spirometry and PEF data (depending on the children's ages) will be analyzed overall and for the age ranges 5 years (PEF only), 6 to 8 years and 9 to 11 years (PEF and spirometry).

Subject Diaries

Local skin reactions (itching, redness, swelling) induced by Viaskin® will be reported daily by the subject during the first 6 months of the study. These local skin reactions will be listed and summarized using the number of days of itching, redness and swelling of Grade 1, 2, or 3 overall and by age ranges (4 to 5 years, 6 to 8 years and 9 to 11 years).

The most severe grades of itching, redness and swelling, documented in the diary, will also be summarized. The maximum grade of local reactions reported (itching, redness or swelling) during the first 6 months will also be tabulated. Finally, the percentage of doses during the first 6 months with any local reaction (itching, redness or swelling) reported by the subject will be estimated and summarized by severity, for each treatment group.

Skin Reactions

Viaskin® patch site examinations assessed by the investigator will be summarized where appropriate and listed.

Examination of the skin at the sites of patch application will be graded by the Investigators on a scale of Grade 0 (negative) to Grade 4 (erythema, vesicles). Localization of the skin reactions (under the patch/beyond the patch) will also be collected. These results will be summarized using descriptive statistics and presented by visit, regardless of the localization as well as for each type of localization. The worst grade reported will also be tabulated.

Sample size calculations:

The sample size of this study is defined to be 335 randomized subjects with 250 subjects to receive Viaskin® Peanut in the initial double-blind period of the study. This number of subjects enables to detect events with an annual rate ≥0.024 after the 6-month double-blind period and will increase the overall Viaskin® Peanut safety database size to a number of subject-years that would allow us to detect events with an annual rate ≥0.0055. After the 6 Month visit, the placebo arm will cross-over to the active treatment arm, and will initiate a 36-month Viaskin® Peanut active treatment.

Assuming a drop-out rate of 15% per year, this will ensure that approximately 284 subjects complete the first year of follow-up, 242 subjects complete up to end of year 2 and 205 subjects complete up to the end of year 3.

Among the 335 subjects randomized, a minimum of 90 subjects 4-5 years will be randomized in the active Viaskin® Peanut 250 µg group, with the randomization ratio 3:1, this means that a minimum of 120 subjects (36% of the Randomized population) 4-5 years of age will be randomized.

Based on the previous experience and the inclusion/exclusion criteria, the screen failure rate could be up to 30%; hence, 480 peanut-allergic subjects might need to be screened.

Exploratory analyses:

The following exploratory endpoints will be analyzed using observed data:

- Change from baseline in peanut-specific IgE and IgG4 at Month 3, 6, 9, 12, 18, 24, 30, 36 for all subjects and Month 42 for subjects initially in the placebo group,
- Change from baseline in peanut component-specific IgE and IgG4 at Month 3, 6, 9, 12, 18, 24, 30, 36 for all subjects and Month 42 for subjects initially in the placebo group,
- Change from baseline in peanut skin prick testing mean wheal diameters at Month 3, 6, 9, 12, 18, 24, 30, 36 for all subjects and Month 42 for subjects initially in the placebo group,
- Description of the quality of life questionnaires (Food Allergy Quality of Life Questionnaire [FAQLQ]/Food Allergy Independent Measure [FAIM]). Global FAQLQ score as well as FAQLQ score by domain will be derived and

change from baseline will be presented at Month 6, 12, 18, 24, 30, 36 for all subjects and Month 42 for subjects initially in the placebo group.

- Description of reactions triggered by accidental consumption of peanut during the study and description of risk-taking behaviors using the frequency of deliberate ingestion of peanut;
- Epigenetic modifications of the promoters of specific genes;
- Sensitization status to some other allergies and their evolution over the study period;
- SCORAD evolution over time.

The open food challenge(s) performed upon the investigator's decision after 12 months of active treatment with Viaskin® Peanut 250 µg will be reported in the e-CRF.

When performed, the following key data of the open peanut food challenge will be reported and described:

- Peanut Eliciting Dose (ED),
- Peanut Cumulative Reactive Dose (CRD),
- Change in CRD from the last historical CRD obtained from a peanut-food challenge performed before entering the study when available to the CRD from the peanut food challenges performed at any time points during the study between 12 months and 36 months of active treatment,
- Percentage of subjects reaching a CRD ≥1,000 mg peanut protein at any time point after 12 months of active treatment onwards.

Table 1: Schedule of Procedures

ALL SUBJECTS (ACTIVE AND PLACEBO ARMS) – FIRST 6 MONTHS BLINDED PERIOD									
**Study Assessments**	**Screening**	**Double-Blind Treatment Period (from V1 to V6)**							
**Visit tags – PC (Phone Call)**	**V1**	**V2**	**PC**	**V3**	**PC**	**V4**	**PC**	**V5**	**PC**
**Duration in study**	**Screen**	**D1**	**D4**	**D8**	**D22**	**M1**	**M2**	**M3**	**M4.5**
**Time Windows**		within 2 w. of V1	±2 d	±3 d	±2 d	±3 d	±3 d	±7 d	±7 d
Informed consent	X								
Medical history/Peanut allergy or positive Peanut FC documentation[1] / Family atopy history	X								
Check eligibility (inclusion/exclusion criteria)	X	X							
Demographics	X								
Physical examination [2]	X	X		X		X		X	
Vital signs [3]	X	X		X		X		X	
Spirometry (FEV_1) [4]	X								
PEF [5]	X	X		X		X		X	
SCORAD	X							X	
FAQLQ/FAIM [6]	X								
SPT (Skin Prick Test)	X							X	
Immunological markers [7]	X							X	
Laboratory tests [8]	X							X	
Filaggrin gene [9] *(Optional)*								X[7]	
Urine pregnancy test	X								
Epigenetic analyses	X							X	
Treatment Initiation		X							
Adverse events		X	X	X	X	X	X	X	X
Concomitant medications	X	X	X	X	X	X	X	X	X
Check for any accidental peanut consumption			X	X	X	X	X	X	X
Subject diary (dispense/check)		X	X	X	X	X	X	X	X
Dispense subject safety leaflet and subject identification card		X							
Apply Viaskin® patch at site		X							

ALL SUBJECTS (ACTIVE AND PLACEBO ARMS) – FIRST 6 MONTHS BLINDED PERIOD									
**Study Assessments**	**Screening**	**Double-Blind Treatment Period (from V1 to V6)**							
**Visit tags – PC (Phone Call)**	**V1**	**V2**	**PC**	**V3**	**PC**	**V4**	**PC**	**V5**	**PC**
**Duration in study**	**Screen**	**D1**	**D4**	**D8**	**D22**	**M1**	**M2**	**M3**	**M4.5**
**Time Windows**		within 2 w. of V1	±2 d	±3 d	±2 d	±3 d	±3 d	±7 d	±7 d
Time under observation before discharge		3 hrs							
Check skin reactions under the patch and grading [10]		X		X		X		X	
Dispense epinephrine auto-injector and anaphylaxis emergency action plan / 1% hydrocortisone ointment		X							
Review Epinephrine auto-injector use and subject safety precaution information leaflet, including anaphylaxis emergency action plan				X		X		X	
Assessment of used/unused study drug dispensed to the subject and of medication compliance				X		X		X	
Dispense study drug to the subject (Viaskin® Peanut 250 µg or Viaskin® placebo)		X		X		X		X[11]	

Abbreviations: D = Day; d = days; ET = Early termination; FAQLQ/FAIM = Food Allergy Quality of Life Questionnaire/Food Allergy Independent Measure; FEV_1 = Forced expiratory volume in one second; hrs = hours; M = Month; PC = Phone contact; PEF = Peak expiratory flow; SCORAD = Scoring atopic dermatitis; SPT = Skin prick test; UV = Unscheduled Visit; V = Visit;

1. Including history of peanut allergy and peanut FC previously performed, including date, procedures, doses used, challenge material and content in peanut protein, Eliciting Dose and symptoms.
2. Including a systematic complete skin examination, other physical examination as required,
3. Blood pressure, heart rate and respiratory rate.
4. FEV1 will be measured for subjects ≥6 years of age (unless they have documented inability to adequately perform spirometry).
5. PEF will be measured for all the subjects ≥5 years of age.
6. For both FAQLQ and FAIM, subjects ≥8 years of age will use the Child Form of the FAQLQ/FAIM. All parents/guardians will use the Parental Form. The FAQLQ and FAIM forms will be completed.
7. Peanut-specific IgE, peanut-specific IgG4, peanut-specific-component IgE and peanut-specific-component IgG4 to Ara h 1, Ara h 2, Ara h 3, Ara h 8 and Ara h 9. IgE specific to cow's milk, to egg white, to house dust mites, and to grass pollen will be tested at screening, month 3, 6, 9, 12, 18, 24, 30, 36 for all subjects and Month 42 for subjects initially in the placebo group.
8. Laboratory tests performed centrally. Hematology: hemoglobin, hematocrit, platelets, red blood cells, white blood cells with differential cell count. Biochemistry: aspartate aminotransferase, alanine aminotransferase, total bilirubin, blood urea nitrogen, creatinine, total protein.
9. Signing the consent for the filaggrin genetic analysis can be done any time after the subject is included in the study. However, collection of blood is done only once at Visit 5 or 6.
10. Check the reactions of the skin on the back of the subject and grade the severity of the local skin reactions. At Visit 2, grading is to be done before patch application and at 30 min, 1 h, 2 h and 3 h after patch application.

11. At Visit 5, the study drug (Viaskin® Peanut 250µg or Viaskin® placebo) is dispensed for a duration of 3 months.

	ALL SUBJECTS – 6 to 42 MONTHS OPEN LABEL TREATMENT PERIOD																		
**Study Assessments**	**Open Label Active Treatment Period**															**End of Study**	**Early Term**	**Unsched Visit[12]**
**Visit tags**	**V6**	**PC**	**V7**	**PC**	**V8**	**V9**	**V10**	**V11**	**PC**	**V12**	**PC**	**V13**	**PC**	**V14**	**PC**	**V15P**	**EoSV**	**ET**	**UV**
**Duration in study**	**M6**	**M6.5**	**M7**	**M8**	**M9**	**M12**	**M15**	**M18**	**M21**	**M24**	**M27**	**M30**	**M33**	**M36**	**M39**	**M42**			
**Time Windows**	**±7 d**	**±2 d**	**±3 d**	**±3 d**	**±7 d**	**±7 d**	**±7 d**	**±14 d**	**±14 d**	**±14 d**	**±14 d**	**±14 d**	**±14 d**	**±14 d**	**±14 d**	**±14 d**	**≤ 2 w after V14 or V15P**		
**Active Arm Active treatment duration**	M6	M6.5	M7	M8	M9	M12	M15	M18	M21	M24	M27	M30	M33	M36	-	-			
**Placebo Arm Active treatment duration**	D1	D15	M1	M2	M3	M6	M9	M12	M15	M18	M21	M24	M27	M30	M33	M36			
Physical examination [1]	X		X		X	X	X	X		X		X		X		XP	X	X	X
Vital signs [2]	X		X		X	X	X	X		X		X		X		XP	X	X	X
Spirometry (FEV_1) [3]	X					X		X		X		X		X		XP		X	X
PEF [4]	X		X		X	X		X		X		X		X		XP		X	X
SCORAD	X				X	X		X		X		X		X		XP		X	
FAQLQ/FAIM [5]	X					X		X		X		X		X		XP		X	
SPT (Skin Prick Test)	X				X	X		X		X		X		X		XP		X	
Immunological markers [6]	X				X	X		X		X		X		X		XP		X	
Laboratory tests [7]	X				X	X		X		X		X		X		XP		X	X
Filaggrin gene [8] *(Optional)*	X																		
Urine pregnancy test *(optional at other visits)*	X					X				X				X		XP			
Epigenetic analyses	X				X	X		X		X		X		X		XP			
Adverse events	X	X	X	X	X	X	X	X	X	X	X	X	X	X	XP	XP	X	X	X
Concomitant medications	X	X	X	X	X	X	X	X	X	X	X	X	X	X	XP	XP	X	X	X
Check for any accidental peanut consumption	X	X	X	X	X	X	X	X	X	X	X	X	X	X	XP	XP	X	X	X
Subject diary (dispense/check)	X	X	X	X	X	X	X	X	X	X	X	X	X	X	XP	XP	X	X	X
Apply Viaskin® patch at site	X																		

	**ALL SUBJECTS – 6 to 42 MONTHS OPEN LABEL TREATMENT PERIOD**																		
**Study Assessments**	**Open Label Active Treatment Period**																**End of Study**	**Early Term**	**Unsched Visit[12]**
**Visit tags**	**V6**	**PC**	**V7**	**PC**	**V8**	**V9**	**V10**	**V11**	**PC**	**V12**	**PC**	**V13**	**PC**	**V14**	**PC**	**V15P**	**EoSV**	**ET**	**UV**
**Duration in study**	**M6**	**M6.5**	**M7**	**M8**	**M9**	**M12**	**M15**	**M18**	**M21**	**M24**	**M27**	**M30**	**M33**	**M36**	**M39**	**M42**			
**Time Windows**	**±7 d**	**±2 d**	**±3 d**	**±3 d**	**±7 d**	**±7 d**	**±7 d**	**±14 d**	**±14 d**	**±14 d**	**±14 d**	**±14 d**	**±14 d**	**±14 d**	**±14 d**	**±14 d**	**≤ 2 w after V14 or V15P**		
**Active Arm Active treatment duration**	M6	M6.5	M7	M8	M9	M12	M15	M18	M21	M24	M27	M30	M33	M36	-	-			
**Placebo Arm Active treatment duration**	D1	D15	M1	M2	M3	M6	M9	M12	M15	M18	M21	M24	M27	M30	M33	M36			
Time under observation before discharge	3 hrs																		
Check skin reactions under the patch and grading [9]	X		X		X	X	X	X		X		X		X		XP		X	X
Review Epinephrine auto-injector use and subject safety precaution information leaflet, including anaphylaxis emergency action plan	X		X		X	X	X	X		X		X		X		XP		X	X
Assessment used/unused study drug dispensed to the subject	X		X		X	X	X	X		X		X		X		XP		X	X
Peanut Food Challenge (optional) [10]						X[11]		X		X		X		X		XP			
Dispense study drug to the subject (Viaskin® Peanut 250 µg)	X		X		X	X	X	X		X		X		XP					

Abbreviations: D = Day; d = days; ET = Early termination; FAQLQ/FAIM = Food Allergy Quality of Life Questionnaire/Food Allergy Independent Measure; FEV_1 = Forced expiratory volume in one second; hrs = hours; M = Month; PC = Phone contact; PEF = Peak expiratory flow; SCORAD = Scoring atopic dermatitis; SPT = Skin prick test; UV = Unscheduled Visit; V = Visit; XP = Procedure or test only conducted with the subjects initially randomized in the Placebo arm for the first 6 months,

1. Including a systematic complete skin examination, other physical examination as required,
2. Blood pressure, heart rate and respiratory rate.

3. FEV1 will be measured for subjects ≥6 years of age (unless they have documented inability to adequately perform spirometry).
4. PEF will be measured for all the subjects ≥5 years of age.
5. For both FAQLQ and FAIM, subjects ≥8 years of age will use the Child Form of the FAQLQ/FAIM. All parents/guardians will use the Parental Form. The FAQLQ and FAIM forms will be completed.
6. Peanut-specific IgE, peanut-specific IgG4, peanut-specific-component IgE and peanut-specific-component IgG4 to Ara h 1, Ara h 2, Ara h 3, Ara h 8 and Ara h 9. IgE specific to cow's milk, to egg white, to house dust mites, and to grass pollen will be tested at screening, month 3, 6, 9, 12, 18, 24, 30, 36 for all subjects and Month 42 for subjects initially in the placebo group.
7. Laboratory tests performed centrally. Hematology: hemoglobin, hematocrit, platelets, red blood cells, white blood cells with differential cell count. Biochemistry: aspartate aminotransferase, alanine aminotransferase, total bilirubin, blood urea nitrogen, creatinine, total protein.
8. Signing the consent for the filaggrin genetic analysis can be done any time after the subject is included in the study. However, collection of blood is done only once at Visit 5 or 6.
9. Check the reactions of the skin on the back of the subject and grade the severity of the local skin reactions. At Visit 6, grading is to be done before patch application and 30 min, 1 h, 2 h and 3 h after patch application.
10. A maximum of 2 open peanut food challenges can be performed during the course of the study. The first food challenge should be performed after a minimum of 12 months of active treatment. The second peanut challenges can be performed for the same subject only after at least 12 additional months of active treatment have been administered to that subject.
11. Peanut challenge might be conducted at this visit only for the subjects initially randomized in the active arm.
12. Procedures during the unscheduled visits will be performed as deemed necessary by the investigator.

Table 2: Study Schematic Diagram

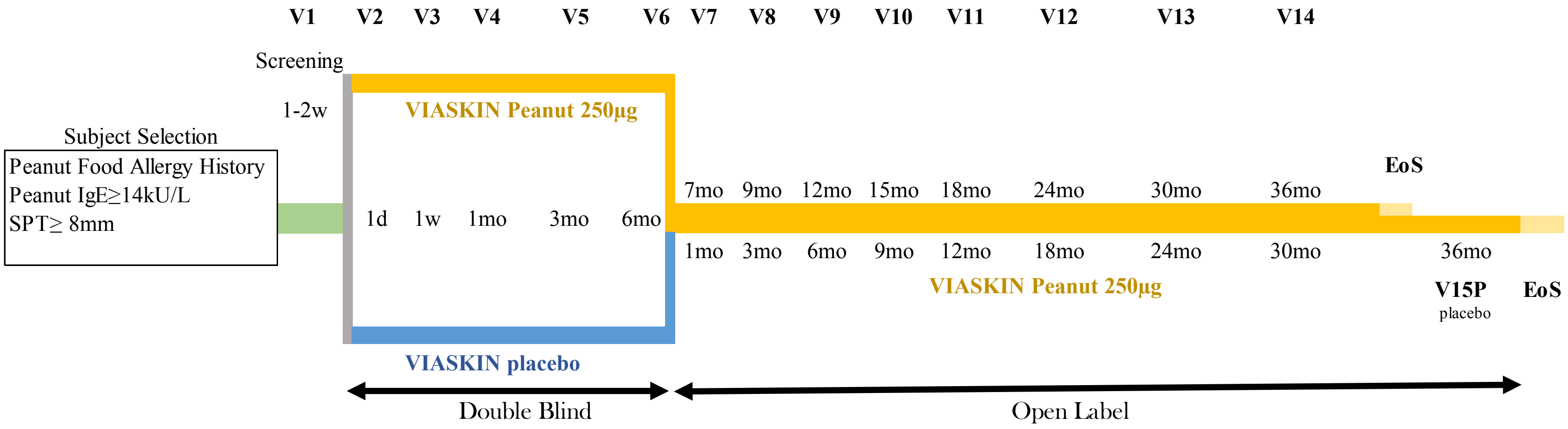

LIST OF STUDY PERSONNEL

Sponsor

Lucia SEPTIEN VELEZ, MD
Chief Medical Officer
DBV Technologies S.A.
177-181 avenue Pierre Brossolette
92120 – Montrouge
France
Tel: + 33 1 84 86 11 98
E-mail: *lucia.septien-velez@dbv-technologies.com*

Wence AGBOTOUNOU, PhD, MBA
Chief Clinical Trial Officer, SVP
DBV Technologies S.A. (same address as above)
Tel: + 33 1 55 42 78 74
E-mail: *wence.agbotounou@dbv-technologies.com*

Claude THEBAULT, MD
VP Biostatistics & Pharmacovigilance
DBV Technologies S.A. (same address as above)
Tel: + 33 1 84 16 29 07
E-mail: *claude.thebault@dbv-technologies.com*

Aurélie PEILLON
Senior Manager, Biostatistician
DBV Technologies S.A. (same address as above)
Tel: + 33 1 84 16 29 44
E-mail: *aurelie.peillon@dbv-technologies.com*

Medical Monitor:
Michel ROUX, MD
VP Medical Operations
DBV Technologies S.A. (same address as above)
Tel: + 33 1 84 86 11 34
E-mail: *michel.roux@dbv-technologies.com*

Contract Research Organization

PAREXEL International (IRL) Limited (“PAREXEL”)
70 Sir John Rogerson’s Quay
Dublin 2
Ireland

**Central Laboratory**	Q^2 Solutions Limited<br>The Alba Campus<br>Rosebank, Livingston<br>EH54 7EG<br>Scotland
**Drug Manufacturing Company**	AMATSI<br>17 Parc des Vautes<br>F-34980 Saint Gely du Fesc (Montpellier)<br>France
**Drug Supply/Packaging Company**	CREAPHARM<br>Z.A. Air-Space<br>Avenue de Magudas<br>CS 2007<br>33187 Le Haillan (Bordeaux)<br>France
**Culprit food of accidental food allergy laboratory**	University of Nebraska-Lincoln<br>Dept of Food Science & Technology<br>FARRP<br>Rm 276 Food Innovation Center<br>1901 North 21 Street<br>Lincoln, Nebraska<br>USA

TABLE OF CONTENTS

PROTOCOL SYNOPSIS 4
LIST OF STUDY PERSONNEL 20
TABLE OF CONTENTS 22
LIST OF ABBREVIATIONS AND DEFINITIONS OF TERMS 27
1. INTRODUCTION 29
1.1. Background 29
1.2. Summary of Findings from Non-clinical and Clinical Studies 30
1.2.1. Non-clinical Studies 30
1.2.2. Clinical Studies 31
1.2.2.1. Study PEP01.09: Phase Ib Safety (Completed) 31
1.2.2.2. ARACHILD Study: Phase II Pilot Efficacy and Safety Study (Completed) 31
1.2.2.3. VIPES Study: Phase IIb Efficacy and Safety Study (Completed) 32
1.2.2.4. OLFUS-VIPES Study: Open-label Follow-up Study of the VIPES Study (Ongoing) 33
1.2.2.5. CoFAR6 Study: Phase II Efficacy and Safety Study (Ongoing) 33
1.2.2.6. PEPITES Study: Phase III Pivotal Efficacy and Safety Study (Ongoing) 36
1.2.3. Clinically Relevant Adverse Events Related to Viaskin® Peanut 36
1.3. Rationale 40
1.4. Risk-Benefit Assessment 40
2. STUDY OBJECTIVES 41
3. OVERALL DESIGN AND PLAN OF THE STUDY 41
3.1. Overview 41
3.2. Study Schematic Diagram 43
3.3. Criteria for Evaluation of the Study 43
3.3.1. Safety Criteria 43
3.3.2. Exploratory Criteria 44
3.4. Justification of the Study Design 45
4. STUDY POPULATION 47
4.1. Inclusion Criteria 47
4.2. Exclusion Criteria 47
4.3. Food Challenge Documentation 49
4.4. Subject Withdrawal and Replacement 49
4.4.1. Criteria for Withdrawal from Study Treatment and Study 49
4.4.2. Study Stopping Rules 50
4.4.3. Replacement of Withdrawn Subjects 50
4.4.4. Data Collection and Follow-up after Withdrawal 51
4.5. Planned Sample Size and Number of Study Centers 51
4.6. Subject Identification and Randomization 51
4.6.1. Subject Identification 51
4.6.2. Randomization Scheme 51
4.6.3. Allocation of Treatment to Subjects 52
5. STUDY DRUG 52

5.1. Identity 52
5.2. Administration 52
5.2.1. Adjustment of Viaskin® Patch Application in Case of Local or Systemic Reactions 54
5.2.2. Safety Precaution Information 55
5.3. Packaging, Labeling and Storage 56
5.4. Blinding and Breaking the Blind 56
5.5. Drug Accountability 57
5.6. Compliance 57
5.7. Prior and Concomitant Medications 58
5.7.1. Permitted Concomitant Medications 58
5.7.2. Prohibited Prior and Concomitant Medications 59
6. VARIABLES AND METHODS OF ASSESSMENT 60
6.1. Demographics and Baseline Characteristics 60
6.1.1. Subjects Demography 60
6.1.2. Disease History and Medical History 60
6.1.3. Prior and Concomitant Medications 60
6.2. Safety Variables 60
6.2.1. Adverse Events 60
6.2.1.1. Collection of Adverse Events 61
6.2.1.2. Definitions 61
6.2.1.3. Assessment of Adverse Events 61
6.2.1.4. Seriousness 61
6.2.1.5. Severity 62
6.2.1.6. Causality 62
6.2.1.7. Local Skin Reactions 63
6.2.1.8. Adverse Events of Special Interest 63
6.2.1.9. Symptoms during Food Challenge(s) performance 64
6.2.1.10. Recording Adverse Events 64
6.2.1.11. Reporting Serious Adverse Events 64
6.2.1.12. Follow-up of Adverse Events 65
6.2.2. Pregnancy Test 65
6.2.3. Treatment of Overdose of Study Medication 66
6.2.4. Laboratory Variables 66
6.2.5. Vital Signs 66
6.2.6. Physical Examinations 67
6.2.7. Spirometry Test 67
6.2.8. Peak Expiratory Flow 68
6.2.9. Subject Diaries 68
6.2.10. Skin Reaction and Photography 69
6.3. Exploratory Variables 70
6.3.1. Immunological Markers 70
6.3.2. Skin Prick Test 70
6.3.3. Food Allergy Quality of Life Questionnaire /Food Allergy Independent Measure 71
6.3.4. Accidental Consumption of Peanut-containing Food 71
6.3.5. Epigenetic analyses 72
6.3.6. Filaggrin Optional Genetic Testing 72
6.3.7. Scoring of Atopic Dermatitis 72
6.3.8. Peanut Food Challenge 72
7. STUDY CONDUCT 73

7.1. Schedule of Procedures ... 73
7.2. Procedures by Visit ... 80
7.2.1. Visit 1, Screening ... 80
7.2.2. Visit 2 (Day 1), First Day of Treatment – Double-Blind Period ... 80
7.2.3. Phone Contacts (Day 4, Day 22, Month 2, Month 4.5) – Double-Blind Period ... 81
7.2.4. Visit 3 (Day 8) and Visit 4 (Month 1) – Double-Blind Period ... 82
7.2.5. Visit 5 (Month 3) – Double-Blind Period ... 82
7.2.6. Visit 6 – Open Label Viaskin® Peanut 250 µg for All Subjects ... 83
7.2.7. Visit 7 (Month 7), Visit 10 (Month 15) - Open Label Period ... 85
7.2.8. Phone Contacts (Month 6.5, Month 8, Month 21, Month 27, Month 33, Month 39 - subjects initially in placebo arm) – Open Label Period ... 85
7.2.9. Visit 8 (Month 9), Visit 9 (Month 12), Visit 11 (Month 18), Visit 12 (Month 24), Visit 13 (Month 30), - Open Label Period ... 86
7.2.10. Visit 14 (Month 36), Visit 15P (Month 42- only for subjects initially in placebo arm) - End of Open Label Period ... 87
7.2.11. End of Study Visit (EoS) ... 88
7.2.12. Early Termination Visit ... 88
7.2.13. Unscheduled Visit ... 89
8. STATISTICAL METHODS ... 89
8.1. Study Subjects ... 89
8.1.1. Disposition of Subjects ... 89
8.1.2. Protocol Deviations ... 90
8.1.3. Analysis Sets ... 90
8.1.4. Randomized Population ... 90
8.1.5. Safety Population ... 90
8.1.6. Safety Sub-Population – Subjects without a history of severe anaphylaxis to peanut ... 90
8.1.7. Safety Sub-Population – Subjects with a history of severe anaphylaxis to peanut ... 90
8.1.8. Per-protocol Population ... 91
8.2. General Considerations ... 91
8.2.1. Statistical Methods ... 91
8.2.2. Analysis and Data Conventions ... 91
8.2.3. Definition of Baseline ... 92
8.2.4. Visit Windows ... 92
8.2.5. Unscheduled Assessments ... 92
8.2.6. Missing Data Conventions ... 92
8.3. Demographics, Disease and Medical History, Baseline Characteristics, and Concomitant Medications ... 93
8.4. Treatment Compliance and Exposure ... 93
8.4.1. Safety Analyses ... 93
8.4.2. Adverse Events ... 94
8.4.3. Laboratory Assessments ... 95
8.4.4. Vital Signs ... 95
8.4.5. Physical Examination ... 95
8.4.6. Spirometry and Peak Expiratory Flow Results ... 96
8.4.7. Subject Diaries ... 96
8.4.8. Skin Reactions ... 96
8.5. Exploratory Analyses ... 96
8.5.1. Immunological markers ... 96
8.5.2. Skin Prick Test ... 97

8.5.3. Food Allergy Quality of Life Questionnaire /Food Allergy Independent Measure 97
8.5.4. Accidental Consumption of Peanut-containing Food 97
8.5.5. Epigenetic analyses 97
8.5.6. Genetic Screening 97
8.5.7. Scoring Atopic Dermatitis 97
8.5.8. Peanut Food Challenge 97
8.6. Interim Analyses 98
8.7. Determination of Sample Size 98
9. ETHICAL, LEGAL, AND ADMINISTRATIVE ASPECTS 99
9.1. Data Quality Assurance 99
9.1.1. Database Management and Quality Control 99
9.2. Case Report Forms and Source Documentation 99
9.2.1. Data Collection 100
9.3. Access to Source Data 100
9.3.1. Routine Monitoring 100
9.3.2. Inspections and Auditing Procedures 101
9.4. Data Processing 101
9.5. Archiving Study Records 101
9.6. Good Clinical Practice 102
9.7. Informed Consent 102
9.8. Protocol Approval and Amendment 103
9.9. Data and Safety Monitoring Board 103
9.10. Duration of the Study 104
9.11. Premature Termination of the Study 104
9.12. Confidentiality 104
9.13. Contractual and Financial Details 105
9.14. Liability and Insurance 105
9.15. Publication Policy 105
9.16. Critical Documents 106
9.17. Clinical Study Report 106
10. REFERENCES 107
11. APPENDICES 110
APPENDIX 1 111
APPENDIX 2 117
APPENDIX 3 119
APPENDIX 4 120
APPENDIX 5 121
APPENDIX 6 122
APPENDIX 7 130
APPENDIX 8 140

Tables in Text

Table 1: Schedule of Procedures....13
Table 2: Study Schematic Diagram 19
Table 3: Incidence of Dosing Reactions per Age Group in the CoFAR6 Study 35
Table 4: Summary of Occurrence of Dosing Symptoms per Applied Dose and per Age Group in the CoFAR6 Study....35
Table 5: Summary of Viaskin® Peanut-related Adverse Events in the VIPES Study in Children (6 to 11 years)....37
Table 6: Time of Occurrence of Viaskin® Peanut-related Treatment-Emergent Adverse Events in the VIPES Study in Children (6 to 11 years) 38
Table 7: Safety of Viaskin® Peanut 250 µg According to Age Group in the VIPES Study in Children (6 to 11 years)....38
Table 8: Skin Reaction Grading System....69
Table 9: Schedule of Procedures....74
Table 10 Criteria of Potentially Clinically Relevant Abnormalities in Vital Signs....95

Figures in Text

Figure 1: Schematic Representation of Viaskin® Patch Application on the Back of the Subjects 53

List of Appendices

APPENDIX 1	Declaration of Helsinki
APPENDIX 2	Dosages of Inhaled Corticosteroids
APPENDIX 3	Activity of Corticosteroids
APPENDIX 4	Wash-out periods for Short-acting and Long-acting Antihistamines based on Terminal Elimination Half-Lives
APPENDIX 5	Anaphylaxis Staging System
APPENDIX 6	Proposed Oral Food Challenge Procedure and Symptom Score Sheets
APPENDIX 7	FAQLQ/FAIM Questionnaires
APPENDIX 8	SCORAD

LIST OF ABBREVIATIONS AND DEFINITIONS OF TERMS

AE	Adverse Event
AESI	Adverse Event of Special Interest
ANCOVA	ANalysis of COVAriance
APC	Antigen-Presenting Cells
AP-HP	Assistance Publique-Hôpitaux de Paris.
ATC	Anatomical Therapeutic Chemical (Classification System)
ATS	American Thoracic Society
CI	Confidence Interval
CoFAR	Consortium of Food Allergy Research
CRD	Cumulative Reactive Dose
CSR	Clinical Study Report
CTCAE	Common Terminology Criteria for Adverse Events
DBPCFC	Double-Blind, Placebo-Controlled Food Challenge
DSMB	Data and Safety Monitoring Board
EAACI	European Academy of Allergy and Clinical Immunology
e-CRF	Electronic Case Report Form
ED	Eliciting Dose
EDC	Electronic Data Capture
EPIT	EPicutaneous ImmunoTherapy
FAQLQ/FAIM	Food Allergy Quality of Life Questionnaire/Food Allergy Independent Measure
FAS	Full Analysis Set
FEV_1	Forced Expiratory Volume in 1 second
FDA	Food and Drug Administration
GA^2LEN	Global Allergy and Asthma European Network
GCP	Good Clinical Practice
IB	Investigator's Brochure
ICF	Informed Consent Form
ICH	International Conference on Harmonization
IEC	Independent Ethics Committee
IgE, IgG, IgG4	Immunoglobulin E, Immunoglobulin G, Immunoglobulin G4 subtype
IRB	Institutional Review Board
ITT	Intent-To-Treat
IV	Intravenous
IWRS	Interactive Web Response System
LEAP	Learning Early About Peanut Allergy
LOCF	Last Observation Carried Forward
MedDRA	Medical Dictionary for Regulatory Activities
NIH	National Institute of Health
OFC	Oral Food Challenge
OIT	Oral ImmunoTherapy
PEF	Peak Expiratory Flow
PP	Per-Protocol
PT	Preferred Term
SAE	Serious Adverse Event

SAP	Statistical Analysis Plan
SCORAD	SCORing Atopic Dermatitis
SLIT	SubLingual ImmunoTherapy
SOC	System Organ Class
SPT	Skin Prick Test
TEAE	Treatment-Emergent Adverse Event
US(A)	United States (of America)
WHO	World Health Organization

1. INTRODUCTION

1.1. Background

Peanut allergy is the most common cause of fatal food allergic reactions [1,2]. An estimated 1% of the population of the United States of America (USA), over 4 million people, are allergic to peanuts or tree nuts [3]. The prevalence of peanut allergy in children has been increasing over the last 2 decades, as indicated by surveys conducted in the USA and United Kingdom: the rate of peanut allergy in children doubled within a range of 5 to 6 years [4,5] and more than tripled between 1997 and 2008 with a prevalence of 0.4% in 1997, 0.8% in 2002 and 1.4% in 2008 [3]. Studies indicate that peanut allergy might resolve in about 20% of young children [6–9], but may recur in some desensitized individuals, making this allergy a life-long affliction in the vast majority of cases.

A recent randomized study of peanut consumption in infants at high risk of developing peanut allergy, the Learning Early About Peanut Allergy (LEAP) study, has evaluated the strategies of peanut consumption or avoidance at very early ages, between 4 and 11 months of age [10]. In the subset of infants at high risk to develop peanut allergy, which were infants with eczema, positive egg skin prick test (SPT), or negative or slightly positive peanut SPT, the results of this prospective study demonstrated that early (during infancy) peanut consumption might be preferable to avoidance of peanut consumption for preventing the occurrence of peanut allergy in infants at high risk.

Peanut allergy falls within the Immunoglobulin E (IgE)-mediated category of food allergies, with immediate reactions triggered by circulating allergen-specific IgE upon exposure to the allergen [11]. IgE-mediated allergic reactions to foods have a rapid onset, usually within a few minutes following exposure to the allergen. IgE-mediated allergic reactions to peanut provoke characteristic responses in the skin, gastrointestinal tract, upper and lower respiratory tract, and cardiovascular system [12]. IgE-mediated reactions to food may also trigger generalized reactions, that is anaphylaxis, a severe, potentially fatal systemic allergic reaction that occurs suddenly after contact with an allergy-causing substance [13].

The complete mechanism of IgE-mediated food allergy remains unknown. However, it is thought that the development of an IgE-mediated response to an allergen is the result of a series of molecular and cellular interactions involving Antigen-Presenting Cells (APCs), T cells and B cells [14,15]. Upon re-exposure to the sensitizing food, the allergen crosslinks specific IgE molecules bound to surface receptors on mast cells and basophils, triggering release of vasoactive and inflammatory mediators such as histamine, leukotrienes, prostaglandins, and platelet-activating factor. The massive release of these mediators induces immediate allergic systemic symptoms, including anaphylaxis.

There are no approved treatments available for peanut allergy [16]. Currently, the only therapeutic option available for peanut-allergic subjects is strict avoidance. However, since peanut is a ubiquitous ingredient in many foods, strict avoidance is difficult to achieve, and accidental ingestion of peanut by peanut-allergic subjects may result in severe reactions and fatal outcomes [17].

The only available countermeasure in case of severe systemic and/or life-threatening reactions/anaphylaxis to peanuts is injectable epinephrine as recommended by the World Allergy

Organization [18]. Epinephrine remains a rescue therapeutic agent and is not designed for a routine use.

Various non-specific and food allergen-specific treatment approaches have been under evaluation. Non-specific approaches to food allergy include the use of monoclonal anti-IgE antibodies, which might increase the Eliciting Dose (ED) threshold for the food allergen [19,20]. Food allergen-specific approaches in clinical development include Oral ImmunoTherapy (OIT), SubLingual ImmunoTherapy (SLIT), and EPicutaneous ImmunoTherapy (EPIT) [21]. Food-specific approaches may be advantageous as they target the specific foods that cause the severe IgE-mediated anaphylactic reactions [22]. Studies on SLIT and OIT have demonstrated some encouraging efficacy results (clinical desensitization), including beneficial immunologic changes [23–26]. Oral immunotherapy has shown evidence for inducing desensitization in most subjects, with immunologic changes over time [25–27]. These advances are, however, hampered by the significant risk of side effects and occurrence of eosinophilic esophagitis in the context of OIT [28–32]. Sublingual immunotherapy for peanut allergy has demonstrated evidence of clinical success, with some subjects showing signs of desensitization with a more satisfactory side effect profile compared to OIT and with significant immunologic changes noted during the first year of therapy [24]. Despite the evident interest of clinicians to further evaluate these treatment procedures, OIT and SLIT will not likely be applicable across all ages and risk categories of peanut-allergic children and adults. Alternative immunotherapeutic approaches for peanut allergy are therefore needed, with a clinically meaningful benefit.

The study drug, Viaskin® Peanut (or DBV712), consists of an epicutaneous delivery system (Viaskin® patch) containing a dry deposit of a formulation of peanut protein extract. The peanut protein allergens are deposited on the backing of an occlusive chamber by electrospraying a liquid formulation of the peanut protein extract. The drug substance is an unmodified, lyophilized peanut extract produced from the extraction and freeze drying of defatted peanut flour, derived from the peanut seed, *Arachis hypogaea*. Further details can be found in the IB [33], which contains comprehensive information on the study drug.

1.2. Summary of Findings from Non-clinical and Clinical Studies

Further details on the studies summarized below can be found in the IB [33].

1.2.1. Non-clinical Studies

DBV Technologies has conducted a number of non-clinical studies supporting the clinical development of Viaskin® Peanut. These include ISO 10993-compliant biocompatibility studies performed for the device component independent of the drug component, which is the Viaskin® patch, and non-clinical studies performed with the combined product, which includes *in vitro* pharmacokinetic/absorption studies, *in vivo* pharmacology (efficacy) studies in a mouse model of peanut allergy, and Good Laboratory Practice-compliant toxicology studies in the rabbit and in a guinea pig model of peanut allergy.

1.2.2. Clinical Studies

1.2.2.1. Study PEP01.09: Phase Ib Safety (Completed)

This randomized, double-blind, placebo-controlled Phase Ib study was conducted to assess the safety and tolerability of Viaskin® Peanut administered epicutaneously to adults (≥18 years of age), adolescents (12 to 17 years of age), and children (6 to 11 years of age) with peanut allergy. A total of 100 subjects, 70 non-severe (without any history of severe anaphylactic reactions) and 30 severe (with a history of severe anaphylactic reactions), were randomized and treated with repeated doses of Viaskin® Peanut or placebo for 2 weeks. Eighty subjects received Viaskin® Peanut at the doses of 20 µg, 100 µg, 250 µg, or 500 µg of peanut protein per patch, and 20 subjects received placebo. Subjects applied 1 patch on the skin every 24 hours or every 48 hours. The patches were applied on the back for the children and on upper arm for the adolescents and adults. Overall, Viaskin® Peanut was safe and well tolerated. No serious adverse events (SAEs) were reported in this study. The most commonly reported adverse events (AEs) were site pruritus, site erythema, site edema, or site urticaria. Viaskin® Peanut triggered more local reactions than placebo. Severe local AEs were reported in 11% of Viaskin® Peanut subjects; these mainly included pruritus (10% of subjects) and were generally transient. In such cases, the subject could remove the patch and allow for the AEs to subside. The maximal tolerated dose was established at 500 µg of peanut protein in adults (severe and non-severe), 500 µg of peanut protein in adolescents (non-severe), and 250 µg of peanut protein in children (non-severe) [34].

1.2.2.2. ARACHILD Study: Phase II Pilot Efficacy and Safety Study (Completed)

This pilot double-blind, placebo-controlled Phase II study was sponsored by the largest French public hospital organization, Assistance Publique-Hôpitaux de Paris (AP-HP). The primary objective of this study was to demonstrate the efficacy of EPIT with Viaskin® Peanut to desensitize children 5 to 17 years of age with a documented peanut allergy. A double-blind, placebo-controlled food challenge (DBPCFC) was conducted at study entry and subjects reacting below a cumulative dose of 300 mg peanut protein were eligible. A total of 54 subjects were randomized at a 1:1 ratio to receive either Viaskin® Peanut 100 µg or placebo for a period of 6 months of blinded treatment. This was first followed by a 12-month open-label treatment period with Viaskin® Peanut 100 µg; the children who were in the placebo group during the first 6 months then crossed-over to also receive Viaskin® Peanut 100 µg for the remaining 12 months, up to Month 18. An amendment to the protocol further extended the open-label treatment period to an additional 18 months for subjects randomized to receive active Viaskin® Peanut treatment from the beginning, and to an additional 24 months for subjects randomized to the placebo group. A total of 30 subjects accepted to extend their study participation under this amendment.

The response rate in the ARACHILD study was defined as the percentage of subjects who reached a cumulative reactive dose of 1,000 mg peanut protein after EPIT treatment or subjects able to multiply by 10 their cumulative reactive dose after treatment as compared to baseline.

This study is the first proof of concept pilot study to demonstrate safety and efficacy of EPIT using Viaskin® Peanut at a unique dose of 100 µg peanut protein. After 6 months of treatment during the blinded period, there was no statistical difference between the 2 treatment groups in the overall population (7.4% response in the active group *versus* 7.7% in the placebo group). However, after

12 months and 18 months of treatment, the overall response rate of the active group increased to 20% and 40%, respectively. Surprisingly, there were no responders among the 12- to 17-year-old adolescents. All responders were children (15) from 5 to 11 years of age. The response rate in these children was 5/15 (33.3%) and 10/15 (66.7%), respectively, at Month 12 and Month 18. Also, in the children (5 to 11 years) who received Viaskin® Peanut, the starting mean cumulative reactive dose at baseline was 23.14±29.31 mg which had progressed to reach 357.66±542.95 mg after 18 months.

In this study, there were 20 SAEs (18 cases). Five were considered related to study procedure, occurring during the DBPCFC: 3 anaphylactic reactions, 1 cutaneous and digestive anaphylaxis, and 1 episode of bronchospasm. All of these events resolved within 24 hours. Four SAEs were considered possibly related to study drug by the Investigators: 1 herpetic gingivostomatitis, 1 pilonidal abscess on a probable pilonidal cyst, 1 recurrence of this pilonidal cyst, and 1 anaphylactic reaction after eating a "kebab" sandwich. In the Viaskin® Peanut group, there was 1 doubtful event of eye pruritus by contact after patch removal in 1 adolescent and 1 edema of the upper lip after patch application in 1 child.

Viaskin® Peanut 100 µg demonstrated an overall satisfactory safety profile. During the double-blind period of the study, 11.5% severe AEs occurred in the placebo group *versus* 7.1% in the Viaskin® Peanut group with mostly skin disorders (none severe), gastrointestinal disorders (2 severe AEs of abdominal pain in the Viaskin® Peanut group), and respiratory disorders (none severe) as compared to placebo. The safety profile of Viaskin® Peanut was compatible with a daily, long-term application.

1.2.2.3. VIPES Study: Phase IIb Efficacy and Safety Study (Completed)

This Phase IIb, double-blind, placebo-controlled study assessed the safety and efficacy of EPIT with Viaskin® Peanut in 221 peanut-allergic subjects (113 children, 73 adolescents and 35 adults) who were randomized to receive Viaskin® Peanut 50 µg, 100 µg, 250 µg, or placebo for 12 months.

With regard to efficacy, the primary efficacy endpoint was met. The treatment response rate in the whole population at Month 12 showed a statistically significant higher responder rate in the Viaskin® Peanut 250 µg group with 28 (50.0%) responders compared to 14 (25.0%) responders in the placebo group (p-value = 0.0108). The study population was composed of 2 age strata: children (6 to 11 years of age) and adolescents and adults (12 years and above). Fifteen (53.6%) children (6 to 11 years) were responders in the Viaskin® Peanut 250 µg group at Month 12 compared to 6 (19.4%) children in the placebo group (p-value = 0.0076). For the main secondary efficacy endpoints analyzed, there was a better response with the highest dose of Viaskin® Peanut (250 µg) than with the lower doses (50 µg and 100 µg) compared to placebo. The results for the children (6 to 11 years) were very favorable in all analyses, as differences compared to placebo were generally even more pronounced with better statistical significance than the results seen in the whole population. In the adolescent and adult age strata, even though Viaskin® Peanut 250 µg showed higher effects for all endpoints analyzed, these were not statistically significant.

Generally, in terms of safety, Viaskin® Peanut patches at 50 µg, 100 µg or 250 µg peanut protein were well tolerated. The most common and most frequent AEs reported by the subjects were local cutaneous reactions at the sites of the patch application in more than 90% of the subjects treated with any dose of Viaskin® Peanut *versus* 50% of the subjects in placebo. These local reactions

were generally of mild or moderate severity, and resulted in withdrawal from treatment in only 2/221 (0.9%) of subjects.

There were no clinical differences for Viaskin® Peanut-related AEs between the 3 active Viaskin® Peanut groups. There were clear differences between the 3 active Viaskin® Peanut groups and the placebo group for these local cutaneous AEs (itching, redness, swelling) at sites of patch application as self-evaluated on a daily basis by the subjects over the first 3 months of EPIT and as assessed by the Investigators over the 12 months of EPIT. One case of Grade 4 local skin reaction (on a scale of 0 = negative to 4 = erythema, vesicles) of erythema with 2-3 vesicles was reported by 1 subject without resulting in subject withdrawal. At all time-points, a higher percentage of subjects in any of the 3 active Viaskin® Peanut groups was assessed as having skin reactions of higher severity than the subjects in the placebo group. Over the course of the study, skin reactions of Grades 1 to 3 appeared during the first month of treatment as the duration of the patch application gradually increased to 24 hours daily. For 50% of the subjects, the local skin reactions lasted less than 2 months and in the other half of subjects the local skin reactions mostly declined over time up to Month 9 or Month 12. Generalized itching after patch removal occurred in 1 child in the Viaskin® Peanut 250 µg group.

There were no SAEs related to Viaskin® Peanut in the VIPES study. Of the 20 SAEs that occurred, 14 SAEs were related to the study procedure of DBPCFC, with subjects reporting anaphylactic reactions that required prolonged hospitalization overnight.

1.2.2.4. OLFUS-VIPES Study: Open-label Follow-up Study of the VIPES Study (Ongoing)

This study evaluates the long-term efficacy and safety of Viaskin® Peanut in children, adolescents and adults. Subjects previously randomized in the VIPES study and who completed the study were offered to receive the Viaskin® Peanut 250 µg treatment for 24 additional months. From the 207 subjects who completed the VIPES study up to Month 12, 171 (83%) have rolled over into the OLFUS-VIPES study. Of the 171 subjects, 44, 41, 38 and 48 subjects were in the VIPES initial treatment groups of 50 µg, 100 µg and 250 µg Viaskin® Peanut and placebo, respectively. The interim analysis results at Month 12 (data collected until and including the DBPCFC performed at 12 months of treatment in the OLFUS-VIPES study for the overall population) showed that majority of subjects (149 [87.1%]) reached Month 12, with only 22 (12.9%) subjects who discontinued from the study before Month 12. The main reason for withdrawal was the unwillingness of the subject to complete the study, followed by lost of follow-up and AE (2 [1.2%] subjects in total: 50 µg: 1 [2.3%] subject and 100 µg: 1 [2.4%] subject). The results of the interim analysis showed that increasing the duration of study treatment was beneficial in this study population. Globally there is no safety-specific concerns so far in the OLFUS-VIPES study.

1.2.2.5. CoFAR6 Study: Phase II Efficacy and Safety Study (Ongoing)

This is a randomized, double-blind, placebo-controlled, Phase II study in children, adolescents and adults sponsored by the National Institute of Health (NIH) and conducted in the USA by the Consortium of Food Allergy Research (CoFAR). The primary objective of this study is to assess the safety and efficacy of EPIT with Viaskin® Peanut in peanut-allergic subjects. In parallel, immunological effects using a set of immune-focused mechanistic studies as well as cellular

modifications induced by EPIT are studied. A total of 75 subjects aged 4 to 25 years (including 10 children aged 4 to 5 years) have been randomized in this study to be treated with either Viaskin® Peanut 100 µg or 250 µg or placebo (ratio 1:1:1). After their first year of blinded treatment in the CoFAR6 study, subjects enter the open-label phase of the study and receive treatment with the 250 µg dose for either 18 additional months for those under active treatment during the blinded phase of the study or for 30 months for those who were under placebo. As of 13 May 2015, an estimated minimum of 60 subjects completed the double-blind period and all subjects rolled over to receive the active treatment with Viaskin® Peanut 250 µg for a total of 30 months.

As of mid-May 2016, 75 subjects were randomized, with 69 subjects having received active treatment: 20 placebo crossover subjects, 24 100 µg peanut subjects, and 25 250 µg peanut subjects. Thirteen randomized subjects (5 children, 7 adolescents and 1 adult) have withdrawn from the study or discontinued dosing. A 6-year-old child withdrew after 1 week of patch treatment because of expressed anxiety surrounding future food challenges associated to the protocol combined to a strong aversion to the peanut taste, and a 8-year-old child withdrew even before any patch application because of unforeseen family circumstances. One adolescent withdrew due to local skin dosing reactions (experience of several days of Grade 3 skin reactions with erythema extending beyond the patch application site and one Grade 4 reaction, i.e. erythema with vesicles), 1 adolescent due to an unrelated SAE (syncopal episodes), 2 for non-compliance or lack of adherence and the 7 remaining subjects due to other circumstances.

Three SAEs were reported (1 child and 2 adolescents), none had a causal relationship with the study product. No SAEs were reported in the population of 4- to 5-year-old children. No subject has died while on study.

In this study, skin reactions at the site of patch application were graded on a scale from Grade 0 (negative according to clinical assessment; normal skin, no reaction, according to subject assessment) to Grade 4 (erythema, vesicles, according to clinical assessment; redness with blisters, according to subject assessment). The symptoms outside the patch site (in a separate area from the patch site, not those that extended beyond the patch site) included assessment of skin reactions, gastrointestinal reactions and respiratory reactions which were scored in severity as mild, moderate or severe.

In January 2014, grading of skin reactions at site of patch application showed that out of the first 366 doses administered, there were 56% with no reactions, 23.3% Grade 1, 18.6% Grade 2 and 1.9% Grade 3 reactions. One dose resulted in the appearance of vesicles at the site of the patch application, which resulted in the subject's permanent discontinuation from treatment and from the study. Also, there were doses (≤5%) resulting in reactions beyond the size of the patch or distant from the patch site, including hives, redness, and pruritus. Of note, 1 of these doses resulting in reactions beyond the patch was an urticarial lesion that extended broadly and covered the upper right quartile of the subject's back.

Safety data gathered for 4- to 5-year old subjects in the CoFAR6 study:

A specific safety assessment for the 4- to 5-year-old subjects was made available for the study period up to 25 May 2014. The incidence of dosing reactions for all subjects by age category is presented in Table 3 and the occurrence of dosing symptoms per applied doses is summarized in

Table 4. Six subjects, all in the ≥6-year-old age group, who did not have any dosing data as of the database closure on 25 May 2014, were not included in the analysis.

Table 3: Incidence of Dosing Reactions per Age Group in the CoFAR6 Study

Type of Dosing Reaction	4 to <6 years	≥6 years
Subjects with no dosing reaction (%)	10.0	12.2
Subjects with patch site reaction only (%)	40.0	67.3
Subjects with patch site and non-patch site reaction (%)	50.0	20.4
Subjects with Grade 3 patch site reaction (%)	20.0	8.2
Subjects with Grade 4 patch site reaction (%)	0.0	2.0

Table 4: Summary of Occurrence of Dosing Symptoms per Applied Dose and per Age Group in the CoFAR6 Study

Dosing Symptoms	4 to <6 years	≥6 years
Total doses reported (n)	1,028	2,751
Any symptoms (%)	70.4	59.7
Patch site reactions (%)	70.2	59.5
• Grade 2 patch site reactions (%)	26.0	15.4
• Grade 3 patch site reactions (%)	0.3	0.2
• Grade 4 patch site reactions (%)	0.0	0.04
Reactions extending beyond the patch site (%)	6.9	6.1
• Grade 2 reactions extending beyond the patch site (%)	0.2	1.8
• Grade 3 reactions extending beyond the patch site (%)	0.1	0.0
Non-patch site reactions (%)	1.2	0.8
• Non-patch site reactions with mild symptoms (%)	1.0	0.7
• Non-patch site reaction with moderate symptoms (%)	0.1	0.0
Symptoms lasting >8 hours (%)	64.3	38.5
Treatment was administered (%)	23.4	23.4
Treated with topical steroids (%)	16.6	12.2
Treated with oral antihistamines (%)	5.4	5.5

There were no non-patch site reactions with severe symptoms and there were no doses that resulted in treatment with epinephrine. A total of 87 doses were not taken (13 doses in the 4- to <6-year-old group, and 74 doses in the ≥6-year-old group).

In conclusion, as of 13 May 2015, no drop-outs and no SAEs occurred in the 10 children 4 to 5 years of age, all randomized for more than 12 months in the CoFAR6 study. The comparative safety assessment available for the study period up to 25 May 2014 showed no specific safety concerns in this study population compared to ≥6-year-old subjects. A similar percentage of subjects in the age group 4 to 5 years and the age group ≥6 years reported dosing reactions (90% *versus* 87.8%, respectively). Even though more 4- to 5-year-old children experienced non-patch site reactions (50% *versus* 20.4%), none of these reactions were severe. Occurrence of patch site reactions per applied doses was slightly higher in the age group 4 to 5 years (70.2% *versus* 59.5%) but these were limited to local reactions up to Grade 3 (only 1 adolescent subject had a severe Grade 4 local reaction leading to study discontinuation). Furthermore, similar medications were administered across age groups to treat these AEs.

1.2.2.6. PEPITES Study: Phase III Pivotal Efficacy and Safety Study (Ongoing)

The PEPITES study is a 12-month, Phase III, pivotal, double-blind, placebo-controlled, randomized study to assess the efficacy and safety of Viaskin® Peanut, dosed at 250 µg peanut protein (per patch) in peanut-allergic children from 4 through 11 years of age after a 12-month treatment by EPIT. More than 330 subjects are planned to be randomized in a 2:1 ratio in 31 centers from 5 countries.

The study is ongoing.

1.2.3. Clinically Relevant Adverse Events Related to Viaskin® Peanut

Safety data are summarized in tabular format in the IB for all Phase I to II trials conducted so far with Viaskin® Peanut [33]. To date, Viaskin® Peanut, regardless of the dose or the age of the subjects, was investigated in 4 randomized controlled trials and in 1 open-label follow-up study (see Section 1.2.2.1 to Section 1.2.2.5) in more than 400 subjects with peanut allergy.

Safety data of the VIPES study with 113 Viaskin® Peanut-treated children are available. The AEs considered related to Viaskin® Peanut by the Investigators are summarized in Table 5.

Table 5: Summary of Viaskin® Peanut-related Adverse Events in the VIPES Study in Children (6 to 11 years)

MedDRA System Organ Class (SOC)	Adverse Events – Preferred Terms
Blood and lymphatic system disorders	Lymphadenopathy
Cardiac disorders.	/
Congenital, familial and genetic disorders	/
Ear and labyrinth disorders	/
Endocrine disorders	/
Eye disorders	Conjunctivitis, Conjunctivitis allergic, Eye allergy, Eye oedema, Eye pruritus, Eye swelling, Eyelid oedema, Lacrimation increased, Orbital oedema
Gastrointestinal disorders	Abdominal distention, Abdominal pain, Abdominal pain upper, Constipation, Diarrhoea, Lip oedema, Nausea, Vomiting
General disorders and administration site conditions *	Asthenia, Application site (Appl. Site) anesthesia, Appl. site bleeding, Appl. site dermatitis, Appl. site discharge, Appl. site discolouration, Appl. site dryness, Appl. site eczema, Appl. site erosion, Appl. site erythema, Appl. site exfoliation, Appl. site itching, Appl. site haematoma, Appl. site irritation, Appl. site oedema, Appl. site pain, Appl. site papules, Appl. site paraesthesia, Appl. site pruritus, Appl. site rash, Appl. site reaction, Appl. site redness, Appl. site scar, Appl. site swelling, Appl. site urticaria, Appl. site vesicles, Chest discomfort, Inflammation, Influenza-like illness, Pyrexia
Hepatobiliary disorders	/
Immune system disorders	Anaphylactic reaction, Food allergy, Hypersensitivity
Infections and infestations	Bronchitis, ear infection, Gastroenteritis, Rhinitis, Tracheitis
Injury, poisoning and procedural complications	/
Investigations	/
Metabolism and nutrition disorders	/
Musculoskeletal and connective tissue disorders	Musculoskeletal stiffness
Neoplasms benign, malignant and unspecified	/
Nervous system disorders	Headache, Migraine, Paresthesia mucosal, Presyncope, Sleep disturbances
Pregnancy, puerperium and perinatal conditions	/
Psychiatric disorders	Middle insomnia, Mood altered
Renal and urinary disorders	/
Reproductive system and breast disorders	/
Respiratory, thoracic & mediastinal disorders	Asthma, Asthma crisis, Cough, Dyspnoea, Epistaxis, Nasal congestion, Rales, Rhinitis, Rhinitis allergic, Rhinorrhea, Sneezing, Throat irritation
Skin and subcutaneous tissue disorders *	Acne, Blister, Dermatitis, Dermatitis atopic, Dry skin, Eczema, Erythema, Heat rash, Papule, Pruritus, Pruritus generalized, Rash, Rash papular, Rash pruritic, Skin disorder, Skin irritation, Skin lesion, Urticaria, Urticaria contact
Social circumstances	/
Surgical and medical procedures	/
Vascular disorders	Pallor

* Frequently occurring (≥ 10%) otherwise < 5%.

The experience from the VIPES study with Viaskin® Peanut 250 µg in children showed that the most frequently observed local skin AEs are application site erythema, application site pruritus, application site swelling, application site dermatitis, and application site papules. In approximately 35% of the subjects, these local skin reactions may be severe for 1 day or more. The majority of the local skin reactions developed within the first 2 months of therapy. They generally decreased in severity over time and resolved without sequelae and without treatment discontinuation.

In the children (6 to 11 years), time of occurrence of treatment-emergent AEs (TEAEs) considered related to Viaskin® Peanut was distributed as indicated in Table with 70% of events appearing during the first 6 months.

Table 6: Time of Occurrence of Viaskin® Peanut-related Treatment-Emergent Adverse Events in the VIPES Study in Children (6 to 11 years)

Time TEAE occurred				
Time frame	Frequency	Percent	Cumulative Frequency	Cumulative Percent
<Day 90	127	37.03	127	37.03
≥Day 90 to <Day 180	114	33.24	241	70.26
≥Day 180 to <Day 270	62	18.08	303	88.34
≥Day 270	40	11.66	343	100.00

Abbreviations: TEAE = Treatment-emergent adverse event.

The safety profile of the Viaskin® Peanut 250 µg group in the VIPES study according to the age range of children (6 to 7 years, 8 to 9 years and 10 to 11 years) and total is shown in Table 7.

Table 7: Safety of Viaskin® Peanut 250 µg According to Age Group in the VIPES Study in Children (6 to 11 years)

	6 to 7 years N=7	8 to 9 years N=13	10-11 years N=8	Total (N=28)
**Data from Subject Diary Proportion of Days*, Mean (SD)**				
Itching				
Grade 2-3	28.4 (32.7)	16.2 (22.3)	26.3 (26.5)	22.1 (25.9)
Grade 3	6.5 (14.4)	1.6 (2.7)	12.2 (23.4)	5.9 (14.5)
Redness				
Grade 2-3	35.1 (37.3)	16.9 (18.7)	31.5 (29.1)	25.6 (27.5)
Grade 3	10.5 (18.8)	0.4 (0.6)	4.4 (7.3)	4.1 (10.5)
Swelling				
Grade 2-3	27.6 (37.3)	12.1 (14.8)	29.0 (33.1)	20.8 (27.5)
Grade 3	12.7 (22.1)	0.4 (0.7)	8.8 (14.9)	5.9 (14.0)
**Investigator Skin Observation, n (%)**				
Month 1, prior to patch application				
Grade 0	3 (42.9)	5 (38.5)	5 (62.5)	13 (46.4)
Grade 1	1 (14.3)	2 (15.4)	1 (12.5)	4 (14.3)
Grade 2	1 (14.3)	3 (23.1)	2 (25.0)	6 (21.4)
Grade 3	2 (28.6)	3 (23.1)	0 (0.0)	5 (17.9)
Month 3, prior to patch application				
Grade 0	2 (33.3)	4 (30.8)	2 (28.6)	8 (30.8)
Grade 1	2 (33.3)	2 (15.4)	1 (14.3)	5 (19.2)

	**6 to 7 years N=7**	**8 to 9 years N=13**	**10-11 years N=8**	**Total (N=28)**
Grade 2	0 (0.0)	5 (38.5)	3 (42.9)	8 (30.8)
Grade 3	2 (33.3)	2 (15.4)	1 (14.3)	5 (19.2)
Missing	1	0	1	2
**TEAEs Considered Related, n (%)**				
Any TEAE	7 (100)	12 (92.3)	8 (100)	27 (96.4)
Eyes disorders	1 (14.3)	0 (0.0)	0 (0.0)	1 (3.6)
Gastrointestinal disorders	3 (42.9)	0 (0.0)	0 (0.0)	3 (10.7)
General disorders and administration site conditions	6 (85.7)	12 (92.3)	8 (100)	26 (92.9)
Skin and subcutaneous tissue disorders	3 (42.9)	6 (46.2)	1 (12.5)	10 (35.7)
**TEAEs considered related by maximum severity, n (%)**				
Any TEAE				
Mild	4 (57.1)	6 (46.2)	4 (50.0)	14 (50.0)
Moderate	2 (28.6)	6 (46.2)	4 (50.0)	12 (42.9)
Severe	1 (14.3)	0 (0.0)	0 (0.0)	1 (3.6)
General disorders and administration site conditions				
Mild	4 (57.1)	7 (53.8)	4 (50.0)	15 (53.6)
Moderate	1 (14.3)	5 (38.5)	4 (50.0)	10 (35.7)
Severe	1 (14.3)	0 (0.0)	0 (0.0)	1 (3.6)
Skin and subcutaneous tissue disorders				
Mild	1 (14.3)	3 (23.1)	0 (0.0)	4 (14.3)
Moderate	1 (14.3)	3 (23.1)	1 (12.5)	5 (17.9)
Severe	1 (14.3)	0 (0.0)	0 (0.0)	1 (3.6)

Abbreviations: n = Number of subjects; SD = Standard deviation; TEAE = Treatment-emergent adverse event.
*During the 90 first days of study treatment.

With regards to the incidence of subjects with itching, redness or swelling by grade, assessed by the subjects over the first 3 months of treatment, there was no clinically significant difference in the Viaskin® Peanut 250 µg group between the 6- to 7-year-old children and the 10- to 11-year-old children, suggesting that the younger children and the older children would react alike locally. Even though it seemed that 8- to 9-year-old subjects reported less frequent local cutaneous reactions

Regarding the Investigator skin observation assessments, the pooled incidences of severity of Grade 2 and 3 were similar in the 3 age ranges at the Month 3, with a slight trend towards less reactions for the 6- to 7-year-old subjects.

The incidence of TEAEs by System Organ Class (SOC) considered treatment related were not clinically significantly different between the 3 age ranges, except for the SOC gastrointestinal disorders, with few more cases in the group of 6- to 7-year-old children.

The data presented above support an overall conclusion that no safety concerns have been raised in association with the use of Viaskin® Peanut 250 µg in children, regardless of age. This is also confirmed by the fact that no children withdrew due to AEs.

1.3. Rationale

Viaskin® Peanut is a ready-to-use and easy-to-administer form of allergen immunotherapy called Epicutaneous Immunotherapy (EPIT), particularly adapted to the pediatric population. It is intended to induce clinical desensitization/tolerance to peanut in subjects allergic to peanut through interaction with the local APCs such as the epidermal Langerhans and dendritic cells. By utilizing the epicutaneous route of administration, Viaskin® Peanut is able to initiate these immunomodulatory processes while minimizing the potential safety concerns associated with systemic exposure to peanut allergenic proteins.

In the 12-month, Phase IIb VIPES study, among the 3 doses tested (50 µg, 100 µg and 250 µg), the highest dose of 250 µg Viaskin® Peanut displayed a strong efficacy with the highest magnitude of effect in children with a good safety profile as described in Section 1.2.2.3. Based on these findings, Viaskin® Peanut at 250 µg used for EPIT for a duration of 12 months is considered to be the optimal and suitable dose for treating children with peanut allergy. The dose of 250 µg was thus the dose of Viaskin® Peanut selected for children aged 4 through 11 years for the ongoing pivotal Phase III study (PEPITES study). The same dose of 250 µg Viaskin® Peanut is used in the current study which was designed in the same age range subjects from 4-11 years for addressing several objectives:

- Increase the short and long-term safety database of the Viaskin® Peanut at the 250 µg dose in children from 4-11 years of age over a 36-month treatment period;
- Explore the therapeutic benefit of Viaskin® Peanut 250 µg in children over a 36-month treatment period.
- To gain experience about the use of Viaskin® Peanut 250 µg in the usual conditions of medical practices.

1.4. Risk-Benefit Assessment

The primary safety concern for any allergen specific immunotherapy is related to the risk of inducing systemic, severe or life-threatening allergic reactions. Viaskin® Peanut applied epicutaneously was developed with the objective to dramatically reduce the risk of these severe systemic reactions by applying the peanut allergens on the skin and reaching the immune system through the cutaneous Langerhans and dendritic cells. Safety information from the completed Phase Ib PEP01.09 study, the Phase II ARACHILD study and the Phase IIb VIPES study, as well as available safety information from the ongoing studies OLFUS-VIPES and CoFAR6, have demonstrated a good safety profile for Viaskin® Peanut 250 µg in children as of 4 years of age and above. The expected local skin reactions triggered by Viaskin® Peanut at the site of patch application, mainly pruritus, erythema, edema, and urticaria, are in the majority of cases of mild or moderate intensity and managed and controlled satisfactorily by the subjects with topical medications containing corticosteroids. As a consequence, a very good compliance above 92% was observed in the studies conducted so far.

Furthermore, especially in the dose-finding Phase IIb VIPES study, Viaskin® Peanut 250 µg has shown a statistically significant treatment effect with up to 53.6% of children responding positively to the treatment *versus* 19.4% for placebo, and the primary endpoint was met. In a post-hoc analysis with a more stringent criterion for the treatment benefit (subjects to reach ≥300 mg peanut

protein if their initial peanut eliciting dose was 1-10 mg peanut protein; subjects to reach ≥1000 mg peanut protein if their initial peanut eliciting dose was 30-300 mg peanut protein), the magnitude of effect of Viaskin® Peanut 250 µg was even higher (46.5% response rate in the active treatment group *versus* 6.5% response rate for placebo).

Overall, the available information gathered from several clinical studies conducted in children with Viaskin® Peanut suggest that Viaskin® Peanut 250 µg presents a favorable benefit-risk ratio.

2. STUDY OBJECTIVES

The objectives of this study are to assess the safety of Viaskin® Peanut 250 µg in peanut-allergic subjects 4 through 11 years of age over a 36-month treatment period, to explore the treatment benefit over a 36-month treatment period and to gain experience about the use of Viaskin® Peanut 250 µg in the usual conditions of medical practices (real life).

3. OVERALL DESIGN AND PLAN OF THE STUDY

3.1. Overview

This is a randomized 36-month, Phase III, safety study to assess the long-term safety of Viaskin® Peanut, dosed at 250 µg peanut protein (per patch) in peanut-allergic children from 4 through 11 years of age.

The study design is in 2 main parts:

- A randomized, double-blind, placebo-controlled design for the first 6 months of treatment,
- Followed by an open-label, single arm active treatment design with Viaskin® Peanut 250 µg.

After selection, the subjects will be randomized with a ratio of 3:1, in either the active arm or placebo arm. The randomization will be stratified by center and age group (children 4 and 5 years of age in a stratum and children 6-11 years in a second stratum).

The duration of the open label period will depend on the initial treatment group of the subjects. After the initial 6-month double-blind period, subjects initially randomized in the active Viaskin® Peanut 250 µg group will continue their active treatment for 30 months. Subjects initially randomized in the placebo group will cross-over to receive the active treatment Viaskin® Peanut 250 µg for 36 months. However, the randomization code will not be unblinded at that time. To ensure that the placebo subjects switching to receive the active treatment will start their treatment safely, all subjects will repeat the progressive increase of daily duration of the Viaskin® Peanut 250 µg patches application, as at the start of the study. The randomization code will be unblinded and the treatment groups assigned during the first 6 months will only be communicated to the investigators and the subjects after the 6-month database lock.

The overall maximum total study duration for each subject ranges from 3 to 3.5 years:

- A 2-week screening period,
- Subjects initially in the active arm: 36 months of treatment,
- Subjects initially in the placebo arm: 42 months of treatment (6 months under placebo + 36 months of active treatment),

- A 2-week follow-up period after the end of the treatment.

The subjects selected for the study must have a physician-diagnosis of peanut allergy based on the presence of a well-documented medical history of IgE-mediated reactions after ingestion of peanut i.e. allergic reaction(s) having led to an emergency department visit or a physician consultation and currently following a strict peanut-free diet.

Once selected, the subject will perform several procedures and must at least fulfill the two following criteria to be eligible for treatment: a peanut SPT with a largest wheal diameter ≥8 mm and peanut-specific IgE ≥14 kU/L.

Subjects will apply daily on an intact skin a Viaskin® patch containing 250 µg peanut protein for a period of 36 months.

Key assessments of safety will be performed at each study visit by the Investigators, including skin observation of the patch areas of application (inter-scapular area of the back), spirometry, peak expiratory flow (PEF) measurements, vital signs, physical examinations and laboratory assessments. Atopic dermatitis will also be assessed using the Scoring atopic dermatitis (SCORAD), for which specific training will be provided for better use and accurate assessment.

Laboratory parameters will include peanut-specific immunoglobulin E (IgE), peanut component-specific IgE, peanut-specific immunoglobulin G4 (IgG4), peanut component-specific IgG4. Skin prick tests (SPTs) will also be performed.

During the whole study duration, any AE will be spontaneously reported by the subjects in the dedicated section in the diaries as well as any used concomitant medications.

- During the first 6 months, 3 pre-specified symptoms (itching, redness, swelling) will be reported on a daily basis in the diaries. These 3 pre-specified solicited symptoms will not be reported in the AE pages of the electronic case report from (e-CRF), except if these symptoms are part of another concomitant disease or if these symptoms are leading to the subject's study discontinuation or serious AEs. Any other local skin reactions or any other type of adverse events will be spontaneously reported in the diaries and will be reported by the investigators in the AE e-CRF form.
- After month 6, any AE, including the 3 above symptoms, other local skin reactions or any other type of AE will be spontaneously reported in the dedicated section of the diaries. These AEs will be reported by the investigators in the e-CRF.

The diaries will be systematically reviewed by the site medical staff at each subject's visit.

Considering the length of this study and for ensuring the subject's medical management, the investigators may need to perform peanut-food challenge during the course of the study.

A maximum of 2 open peanut-food challenges for the same subject is recommended during the subject treatment course up to the 36 months of active treatment. Performing open food challenge(s) in the REALISE study is optional and is left to the investigator's decision. However, no food challenge can occur before the subject has received 12 months of active treatment with Viaskin® Peanut 250 µg. If 2 peanut challenges are performed for the same subject within the treatment course, the second challenge can occur only after at least 12 additional months of active treatment have been administered to that subject.

If the decision is made by the investigators to perform a peanut-food challenge, its performance must comply with the standardized methodology defined in the protocol APPENDIX 6. A standardized peanut food challenge formula and material as well as the Manual of Procedures will be provided to all sites, and must be used for conducting the open peanut food challenge.
All food challenges results will be reported in the e-CRF

Detailed information on study assessments and procedures are provided in Section 6.

All subjects will remain on a peanut-free diet for the duration of the study. The re-introduction or not of peanut into the subject's diet at the end of their participation in the study will be left to the Investigator's decision. The Investigator's decision will be collected in the eCRF.

3.2. Study Schematic Diagram

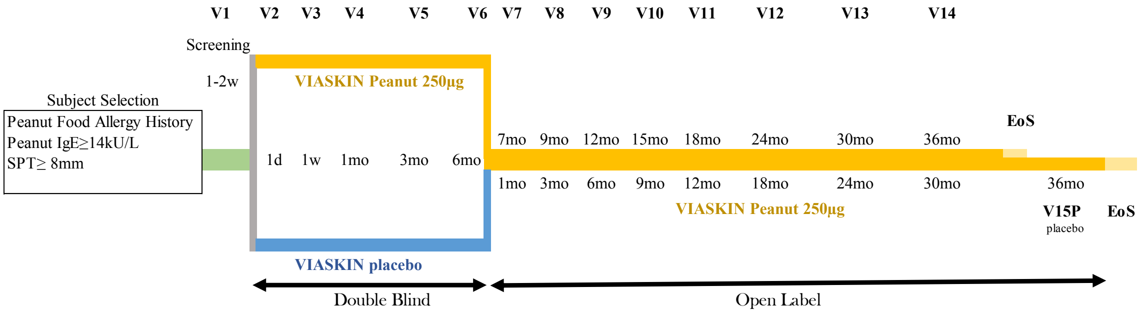

3.3. Criteria for Evaluation of the Study

3.3.1. Safety Criteria

The following study drug safety criteria will be evaluated:

- AEs and Treatment-Emergent Adverse Events (TEAEs) by SOC and Preferred Terms (PTs);
- TEAEs by maximum severity, duration and relatedness to treatment patch, placebo or active;
- TEAEs leading to discontinuation;
- Incidence, duration and maximum severity of local cutaneous Viaskin® patch-induced AEs as assessed by the subject;
- Severity of local cutaneous Viaskin® patch-induced AEs as assessed by the Investigator;
- Adverse Events of Special Interest (AESI) including Grade 4 local cutaneous reactions and systemic allergic AEs considered related to Viaskin® patch;
- SAEs by SOC and PTs and SAEs relatedness to Viaskin® patch;
- Laboratory data, physical examinations and vital signs;
- Spirometry results and PEF results;
- Safety sub-analysis in subjects with mutations in the filaggrin gene *versus* wild type subjects.

During the first 6 months corresponding to the blinded period of treatment, all safety parameters will be presented by treatment group, Viaskin® Peanut 250µg *versus* Placebo. After 6 months and until the end of the study, the placebo treatment group assessments time tags will be rescheduled to consider the actual active treatment duration with Viaskin® Peanut 250 µg, up to 36 months of treatment. Viaskin® Peanut 250 µg and the rescheduled placebo groups will be presented separately and pooled.

These parameters will be first studied separately in two Safety sub-Population groups, i.e. the Safety sub-Population **Without** history of Severe Anaphylaxis (SPwoSA) and the Safety sub-Population **With** history of Severe Anaphylaxis (SPwSA). If no major safety differences are observed (as determined by the Data and Safety Monitoring Board, DSMB), both sub-populations will be pooled and safety analyses will be repeated on the overall Safety population. If the safety profile of the two sub-populations is not comparable, these two sub-populations will be analyzed separately.

The results for the age ranges 4 to 5 years, 6 to 8 years and 9 to 11 years will be presented.

The DSMB, composed of independent experts in food allergy, will review the study safety data at specific intervals during the study and on an *ad hoc* basis.
When performed during the study for the subject medical management, the AEs induced by a peanut-food challenge will be differentiated, as they are expressly provoked, from those AEs occurring outside of a challenge. Objective and subjective symptoms/reactions elicited during a challenge will be summarized separately:

- Symptoms elicited during a peanut-food challenge by severity;
- SAEs elicited during a peanut-food challenge.

Details of the assessment of each of the safety criteria are provided in Section 6.1.

3.3.2. Exploratory Criteria

The following exploratory criteria will be evaluated:

- Change from baseline in peanut-specific IgE and IgG4 at Month 3, 6, 9, 12, 18, 24, 30, 36 for all subjects and Month 42 for subjects initially in the placebo group;
- Change from baseline in peanut component-specific IgE and IgG4 at Month 3, 6, 9, 12, 18, 24, 30, 36 for all subjects and Month 42 for subjects initially in the placebo group;
- Change from baseline in peanut skin prick testing average wheal diameters at Month 3, 6, 9, 12, 18, 24, 30, 36 for all subjects and Month 42 for subjects initially in the placebo group;
- Description of the quality of life questionnaires (Food Allergy Quality of Life Questionnaire [FAQLQ]/Food Allergy Independent Measure [FAIM]) and change from baseline in FAQLQ scores (global score and score by domain) at Month 6, 12, 18, 24, 30, 36 for all subjects and Month 42 for subjects initially in the placebo group;
- Description of accidental consumptions of peanut during the study and analysis of "risk-taking behavior" of subjects (voluntary peanut consumption);
- Epigenetic modifications of the promoters of specific genes;
- Sensitization status to some other allergies and their evolution over the study period;
- SCORAD evolution over time;

- Peanut eliciting doses and peanut cumulative doses reported for any peanut-food challenge performed during the study;
- Symptoms reported during peanut-food challenges, if performed during the study.

For the subjects who will have food challenge(s) performed during the study, the following criteria will be described:

- Peanut Eliciting Dose (ED),
- Peanut Cumulative Reactive Dose (CRD),
- Change in CRD from the last historical CRD obtained from a peanut-food challenge performed before entering the study when available to the CRD from the peanut food challenges performed at any time points during the study,
- Percentage of subjects reaching a CRD ≥1,000 mg peanut protein at any time point after 12 months of active treatment onwards.

Details of the assessment of each of the exploratory criteria are provided in Section 6.3.

3.4. Justification of the Study Design

This study will assess the safety and will explore the treatment benefit of Viaskin® Peanut 250 µg in children aged 4 to 11 years.

The study is designed in 2 periods:

- A randomized, double-blind, placebo-controlled design for the first 6 months of treatment,
- Followed by an open-label, single arm active treatment design with Viaskin® Peanut 250 µg.

The duration of this open label period will depend on the initial treatment group of the subjects. After the initial 6-month double-blind period, subjects initially randomize in the active Viaskin® Peanut 250 µg group will continue their active treatment for an additional 30 months. Subjects initially randomize in the placebo group will cross-over to receive the active treatment Viaskin® Peanut 250 µg for 36 months.

The randomization code will not be unblinded until all subjects will reach Month 6 visit.

All the subjects will repeat the progressive increase of daily duration of application of the Viaskin® Peanut 250 µg patches as described at the start of the study.

The treatment assigned during the 6 first months will only be communicated to the investigators after the 6-month database lock. Then the investigators will communicate the information on the initial treatment group to each subjects.

After selection, the subjects will be randomized with a ratio of 3:1 (active versus placebo).

Peanut allergy is a potentially serious condition and of increasing prevalence in children (3-5). It is thus anticipated that the selected pediatric peanut-allergic subjects will have a direct therapeutic benefit from this study.

This study design was selected to ensure two main objectives:

- To minimize any possible subjects and investigators reporting bias, by ensuring the first 6 months evaluations to be performed in double-blind manner,
- To balance the constraints linked to the placebo control design with the individual benefit that participants and their parents may expect from a long-term participation of 3.5 years in a clinical study.

Therefore, a cross-over design of the placebo arm to the active treatment after the initial 6 months of study was selected. At 6-month all the placebo subjects visit will receive the active Viaskin® Peanut patch treatment for the remaining duration of the study, i.e. ensuring 3 years (36 months) of active treatment to all subjects
The duration of the double-blind period is justified by the higher incidence of adverse events observed in previous clinical studies during the first 3 months following the initiation of the Viaskin® Peanut patch treatment, which lowers after. The initial double-blind 6-months evaluation period should be long enough to ensure that the safety and tolerability related to the treatment initiation is fully captured with double-blind assessments, ensuring unbiased subjects and investigators study procedure assessments. Additionally, long-term placebo treatment to the food-allergic subjects appears to be highly impracticable and ethically questionable.
A ratio 3:1 (active: placebo) allows a size of the placebo control population considered appropriate to serve as control group for the most common adverse events reported with the Viaskin® Peanut use, i.e. administration site conditions and skin disorders with reported incidences of approximately 55% and 20% respectively, as reported in the VIPES study for the 3 active tested doses. This ratio 3:1, favoring the active arm, makes also the study more attractive for the subjects and their families.
This ratio will yield the enrolment of approximately 85 subjects in the placebo arm on top of the 250 subjects to be randomized in the Viaskin® Peanut 250 µg arm.

Subjects participating in this study will be selected on criteria used in usual medical practices for peanut food allergy diagnosis. However more stringent positivity levels than commonly accepted are required for the purpose of this phase III study.
The diagnosis is based on:

- a physician-diagnosed peanut allergy based on a well-documented medical history of IgE-mediated reactions after ingestion of peanut, having led to an emergency department admission or a physician consultation, and currently following a strict peanut-free diet,
- a peanut SPT with the largest wheal diameter ≥8 mm and
- specific-peanut IgE ≥14 kU/L.

These combined criteria widely used in routine medical practices are able to ensure a very high specificity in the peanut food allergy diagnosis, estimated as greater than 95%.
As a consequence, not having to perform a food challenge in the REALISE study will allow the inclusion of the subjects with a history of severe anaphylaxis to peanut. So far, because of the high risk to repeat their severe /life threatening anaphylaxis, these subjects have been excluded from efficacy studies in food allergy immunotherapies including the Viaskin® Peanut clinical studies, despite the potential high individual therapeutic benefit expected in this specific population.
Their participation and the evaluation of Viaskin® Peanut 250 µg safety in this particularly severe sub-population will strengthen and expand the safety knowledge of Viaskin® Peanut.
The selected dose of Viaskin® Peanut 250 µg for treating peanut-allergic children in the REALISE clinical study is based on the safety and efficacy results for 6 to11 years old children in the 12-month Phase IIb VIPES study, but also on the safety information from the CoFAR6 study in subjects 4 to 5 years of age (see Sections 1.2.2.3 and 1.2.2.5). This dose was also tested in a Phase I study on subjects with non-severe and severe peanut-food allergy (Adult subjects with a positive

SPT (wheal diameter ≥8 mm), sIgE >0.7 kU/L, with a history of allergy to peanuts with anaphylaxis of Grade 4 or 5, (in the Grading of Food-Induced Anaphylaxis).

The safety of the treatment with Viaskin® Peanut 250 µg in 4- to 11-year-old children will be evaluated at different time-points: at the end of the double-blind period (6 months), after 12 months of treatment and after 12, 24 and 36 months of active Viaskin® Peanut 250 µg treatment.

The long-term safety of Viaskin® Peanut will be regularly evaluated and reported.

This study will also assess the evidence of peanut desensitization after 12 to 36 months with active Viaskin® Peanut 250 µg EPIT treatment by the change in peanut-specific IgE and SPT testing.

4. STUDY POPULATION

The study population will consist of children with peanut allergy. Study participation will require a signed consent from a legally authorized representative. Subjects must meet all the inclusion criteria and none of the exclusion criteria.

4.1. Inclusion Criteria

Subjects will be enrolled in this study only if they meet all of the following criteria:

1. Male or female children aged 4-11 years at Visit 1;
2. Physician-diagnosed peanut allergy based on a well-documented medical history of IgE-mediated reactions after ingestion of peanut, i.e. allergic reaction(s), having led to an emergency department visit or a physician consultation,
3. Peanut-specific IgE level (ImmunoCAP system) ≥14 kU/L at Visit 1;
4. Positive peanut SPT with a largest wheal diameter: ≥8 mm at Visit 1;
5. Subject following a strict peanut-free diet;
6. Signed informed consent of parents/guardians of the child and child's assent (for children ≥7 years of age or as per the country-specific regulations);
7. Negative urine pregnancy test for female subjects of childbearing potential. Female subjects of childbearing potential must agree and commit to use effective medical methods of contraception for the entire duration of their participation in the study. Sexual abstinence will be accepted as an effective method of contraception;
8. Ability to perform spirometry in accordance with the American Thoracic Society (ATS) guidelines 2007 (1) for subjects ≥6 years of age. Ability to perform peak expiratory flow (PEF) measurements for subjects ≥5 years of age. Subjects from 6 to 8 years of age who have documented inability to adequately perform spirometry can be enrolled if they can perform the PEF measurement.

   Subjects 4 years of age can be enrolled if they had no clinical features of moderate or severe persistent asthma within 1 year prior to visit 1;
9. Subjects and/or parents/guardians willing to comply with all study requirements during the subject's participation in the study.

4.2. Exclusion Criteria

Subjects will be enrolled in this study only if they meet none of the following criteria:

1. Pregnancy or lactation;
2. Any clinically significant abnormality identified at the time of screening such as major infectious diseases (for example chicken pox, measles) which in the judgment of the Investigator may preclude safe participation or strict compliance with the protocol procedures (subjects can be considered for the study after recovery from these diseases);
3. Generalized dermatologic disease (for example active atopic dermatitis, uncontrolled generalized active eczema, ichthyosis vulgaris) extending widely on the skin and especially on the back with no intact zones to apply the Viaskin® patches;
4. Known hypersensitivity to any of the Viaskin® patch components (except peanut), including the adhesive film;
5. Inability to discontinue short-acting antihistamines or long-acting antihistamines for the minimum wash-out periods as specified in the table in annex, prior to the skin prick testing;
6. Spirometry forced expiratory volume in 1 second (FEV_1) <80% of the predicted value at screening (visit 1) for subjects able to perform the spirometry, or PEF <80% of predicted value at screening (visit 1) for subjects performing only the PEF measurements;
7. Diagnosis of asthma that fulfills any of the following criteria:
    a. Uncontrolled persistent asthma as defined by National Asthma Education and Prevention Program Asthma guidelines 2007 [35] or by Global Initiative for Asthma guidelines 2015 [36],
    b. Asthma treated with either
        - a high daily dose of inhaled corticosteroid or
        - with a combination therapy of a medium daily dose of inhaled corticosteroid with a long-acting inhaled β2-agonist or
        - with a combination therapy of a high daily dose of inhaled corticosteroid with a long-acting inhaled β2-agonist (a list of daily dosages of inhaled corticosteroid is provided in annex).

       Asthmatic subjects treated with a medium daily dose of inhaled corticosteroids are eligible. Intermittent asthmatic subjects who require intermittent use of inhaled corticosteroids for rescue are also eligible,
    c. Two or more systemic corticosteroid courses for asthma in the past year or 1 oral corticosteroid course for asthma within 3 months prior to Visit 1,
    d. Intubation/mechanical ventilation for asthma within 1 year prior to Visit 1;
8. Receiving β-blocking agents, angiotensin-converting enzyme inhibitors, angiotensin-receptor blockers, calcium channel blockers or tricyclic antidepressant therapy;
9. Received anti-tumor necrosis factor drugs or anti-IgE drugs (such as omalizumab) or any biologic immunomodulatory therapy within 1 year prior to Visit 1, or planned use during study participation;
10. Use of systemic long-acting corticosteroids within 12 weeks prior to Visit 1 and/or use of systemic short-acting corticosteroids within 4 weeks prior to Visit 1 (unless used for symptoms triggered by food challenges) (see annex);
11. Prior or concomitant history of any immunotherapy to any food allergy (for example EPIT, OIT, SLIT, or specific oral tolerance induction);
12. Receiving or planning to receive any other immunotherapy during their participation in the study. Any immunotherapy must be discontinued at the time of Visit 1;

13. Past or currently active disease(s) which, in the opinion of the Investigator or the Sponsor, may affect the subject's participation in this study or place the subject at increased risk during participation in the study, including but not limited to eosinophilic gastrointestinal disorders, autoimmune disorders, immunodeficiency, malignancy, uncontrolled diseases (for example hypertension, psychiatric illness, cardiac disease), or other disorders (for example liver, gastrointestinal, kidney, cardiovascular, pulmonary disease, or blood disorders);
14. Any disorder in which epinephrine is contraindicated such as coronary artery disease, uncontrolled hypertension, or serious ventricular arrhythmias;
15. Subjects unable to follow the protocol requirements;
16. Current participation in another clinical trial, or participation in another clinical trial in the last 3 months prior to Visit 1, or participation to any previous clinical trial involving the Viaskin® patch;
17. Diagnosis of mast cell disorders including mastocytosis or urticaria pigmentosa as well as hereditary or idiopathic angioedema;
18. Subjects receiving cyclosporine or other immunosuppressive agents within 1 year prior to Visit 1, or during the screening period or during study participation. Topical calcineurin inhibitors are permitted;
19. Subjects with severe psychiatric, psychological or neurological disorders;
20. Subjects being in any personal relationship or dependency with the sponsor and/or the investigator or the study staff. Family members of the sponsor, the investigator or the study staff cannot be part of the REALISE study.
21. Sibling of a subject already included in this REALISE study or in any other clinical trial involving Viaskin® Peanut, especially the PEPITES Study.

4.3. Food Challenge Documentation

For the subjects who will have food challenge(s) to peanut performed any time between 12 months and 36 months of active treatment as decided by the investigators, the following criteria will be described and reported:

- Peanut Eliciting Dose (ED),
- Peanut Cumulative Reactive Dose (CRD),
- Change in CRD from the last historical CRD obtained from a peanut-food challenge performed before entering the study when available to the CRD from the peanut-food challenges performed at any time points during the study,
- Type and severity of the allergic symptoms elicited.

The peanut food allergy information should be available in the subject's medical file and reported in the e-CRF.

4.4. Subject Withdrawal and Replacement

4.4.1. Criteria for Withdrawal from Study Treatment and Study

Subjects may withdraw from the study at any time without penalty and for any reason without prejudice to their future medical care.

Subjects **must be withdrawn** from the study treatment under the following circumstances:

- Pregnancy (see Section 6.2.2);
- Severe "maculo-papular rash" or severe "dermatitis" that cannot be controlled by adequate corrective treatments (including topical corticosteroids) and in spite of several study treatment interruptions;
- Severe anaphylaxis (or stage 3 anaphylaxis) (see APPENDIX 5) related to Viaskin® Peanut 250 µg patch application;
- More than 1 epinephrine injection for an AE related to Viaskin® Peanut 250 µg patch application (and not occurring during the food challenge).

If subjects are to withdraw due to pregnancy, the procedures that should be followed are described in Section 6.2.2.

Subjects **may be required to withdraw** from the study treatment or the study after discussion with the Sponsor and/or Investigator for the following reasons:

- AE(s);
- At the discretion of the Investigator, if she/he decides that it is in the subject's best interest to be withdrawn from the study;
- The subject is unwilling to continue in the study (consent withdrawal);
- Lack of compliance with protocol requirements, study treatment or procedures;
- The Sponsor, Regulatory Authorities, or Independent Ethics Committees (IECs)/Institutional Review Boards (IRBs) for any reason, stop the study;
- The subject fails to return to the clinic for scheduled visits and does not respond to telephone or written attempts at contact (lost to follow-up);
- Premature termination of the entire study as described in Section 9.11.

In all cases, the primary reason for withdrawal, must be recorded in the e-CRF.

4.4.2. Study Stopping Rules

Study will be suspended, pending an expedited safety review by the independent DSMB, if any of the following occur:

1. Any death related to Viaskin® patch application;
2. More than one case of severe anaphylaxis (or stage 3 anaphylaxis, APPENDIX 5) related to Viaskin® Peanut 250 µg patch application (not occurring during the food challenge);
3. More than 3 subjects requiring more than 1 injection of epinephrine in relation to the Viaskin® patch application (and not occurring during the food challenge);

Upon safety review, 1 of the following outcomes will be determined:

- Study may continue without modification;
- Study may continue with modifications as prescribed by the DSMB;
- Study should be discontinued.

4.4.3. Replacement of Withdrawn Subjects

Subjects who withdraw prematurely after having received at least 1 dose of the study drug will not be replaced.

4.4.4. Data Collection and Follow-up after Withdrawal

If a subject is prematurely withdrawn from the study for any reason before completing all study visits, the Investigator must make every effort to perform the evaluations described for the Early Termination Visit (see Section 7.2.12). The Investigator must furthermore complete all appropriate e-CRF pages, providing the date and explanation for the subject's withdrawal/discontinuation. When indicated, the Investigator must arrange for appropriate follow-up and/or alternative medical care of the discontinued subject.

If the subject fails to attend a scheduled End of Study Visit, there will be at least 2 documented attempts to contact the subject's parents/guardians via telephone and written communication. If these receive no reply, the subject will be considered as lost to follow-up.

4.5. Planned Sample Size and Number of Study Centers

It is planned to enroll 335 subjects at 28 to 40 centers in a minimum of 2 countries for this study. See Section 8.7 for a discussion on sample size.

4.6. Subject Identification and Randomization

4.6.1. Subject Identification

At screening, each subject will receive a unique, 4-digit, screening number. Subjects failing screening will retain their screening number. The screening number for each subject will be a combination of the 2-digit site number plus the 2-digit number assigned to the subject according to her/his chronological order of screening at that site. The screening number will be used as the subject identifier throughout the study.

4.6.2. Randomization Scheme

An IWRS will randomize subjects and assign treatment or kit numbers during the study.

In the first part of the study (6 months), subjects will be randomized by the IWRS to either Viaskin® Peanut 250 µg or placebo with a 3:1 ratio.

After the Month-6 visit, an open label Viaskin® Peanut 250 µg treatment will be assigned to all subjects by the IWRS without unblinding any subject.

The treatment assigned during the first 6 months will only be communicated to the investigators and the subjects after the 6-month database lock.

Randomization will be stratified by center and age group.

To ensure an adequate and sufficient representation of the youngest subjects 4 and 5 years of age in the final safety database, a minimum of 90 subjects of these ages must be randomized in the active arm. With the randomization ratio 3:1, this means that a minimum of 120 subjects (36% of the randomized population) 4-5 years of age will be randomized. Hence, the study will also be stratified by age.

The two strata of the study will comprise:

- Stratum 1 composed of a minimum of 120 subjects 4-5 years of age;
- Stratum 2 composed of approximately 215 subjects 6-11 years of age.

Blinding and decoding procedures are described in Section 5.4.

4.6.3. *Allocation of Treatment to Subjects*

Subjects will initiate Viaskin® patch treatment at Visit 2 after all screening procedures have been performed and eligibility of the subject for participation in the study is confirmed. Each subject will be assigned at each visit with the kit number assigned by the IWRS.

5. STUDY DRUG

5.1. Identity

The study drug, Viaskin® Peanut, is a cutaneous patch (Viaskin® patch) containing a solid deposit of a formulation of peanut protein extract. The Viaskin patch is a 34 mm-side long square-shaped patch with a rounded condensation chamber of 18 mm-inner diameter. The peanut allergens are deposited on the backing of the patch chamber by electrospraying a liquid peanut protein formulation, which dries instantly. The outer adhesive part of the condensation chamber is composed of a small band of adhesive foam to stick to the skin. The Viaskin® patch is identical to the ones used in the phase III clinical study (PEPITES) and includes a hypoallergenic adhesive film that improve the adhesion of the Viaskin® patch.

The drug substance is an unmodified, lyophilized peanut extract produced from the extraction and freeze drying of defatted peanut flour. The drug substance contains the biologically active ingredients, the peanut proteins. This drug substance derives from a natural source material of biologic origin, the peanut seed from the Virginia variety of *Arachis hypogaea* and the extract contains all peanut proteins.

The other components of the study drug are inactive excipients: ethanol, surfactant (Polyoxyl 20 oleyl ether), and buffering agents (trometamol and histidine).

Study subjects will be administered either Viaskin® Peanut at 250 µg peanut protein (active) or Viaskin® patch with placebo, according to randomization. The placebo treatment will consist of a similar formulation, but will be devoid of peanut protein.

Both, Viaskin® Peanut 250 µg and Viaskin® placebo will be manufactured by AMATSI, Montpellier, France and labeled, packaged and released for clinical use by CREAPHARM, Bordeaux, France, in accordance with the requirements of Good Manufacturing Practices. At the month 6 visit, all study subjects will receive Viaskin® Peanut at 250 µg peanut protein.

5.2. Administration

During the double-blind treatment period, subjects will be randomized to receive either active Viaskin® Peanut 250 µg or placebo, in a 3:1 ratio (active *versus* placebo).

During the active treatment period, except for the 2 weeks of progressive increase of the daily duration of application of the patch (see below), the Viaskin® patch will be applied on the skin for 24 hours (± 4 hours of allowance) every day and renewed on a daily basis, i.e. 1 new patch per day.

The location of patch application is the inter-scapular area of the back of the subjects. There will be 6 zones for applying the patch, 3 on each side of the spine (see Figure 1). The first Viaskin® patch will be applied on zone 1, the second on zone 2 (after removal of the first patch), and so

forth, until all 6 zones have been used. After zone 6, dosing restarts with zone 1 and continues sequentially, as described.

Figure 1: Schematic Representation of Viaskin® Patch Application on the Back of the Subjects

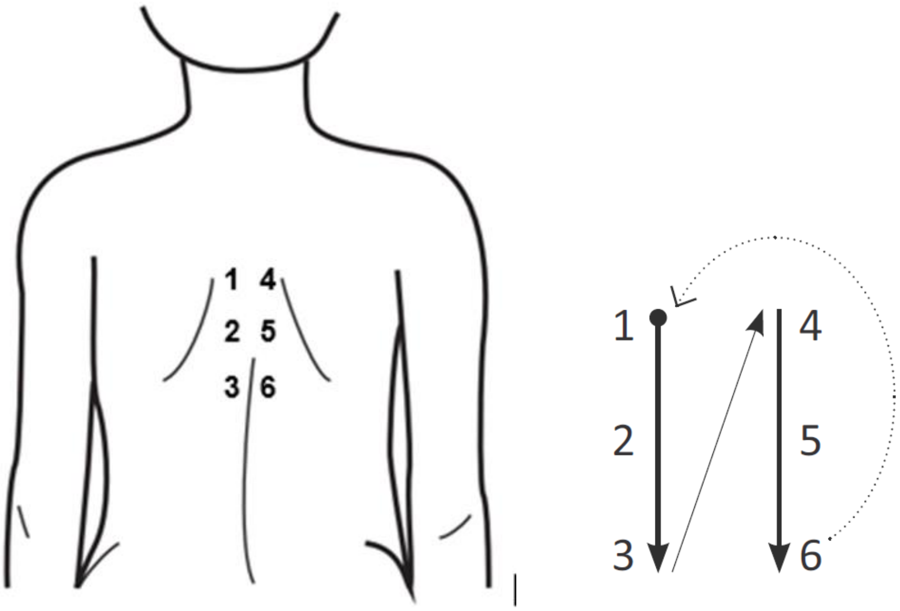

To ensure an enhanced local tolerance at the initiation of Viaskin® Peanut treatment, the duration of application of the Viaskin® patch will be progressively increased as follows:

- During the first week (from Day 1 through Day 7), the patches will be applied for 6 hours (± 1 hour) each day;
- During the second week (from Day 8 through Day 14), the patches will be applied for 12 hours (± 2 hours) each day;
- From the third week onwards (Day 15), the patches will be applied each day for the entire 24 hours (± 4 hours).

From the month 6 visit, all the subjects will receive the active treatment Viaskin® Peanut 250 µg in an open label manner. However, the randomization code will not be unblinded at that time. To ensure that the placebo subjects switching to receive the active treatment will start their treatment safely, all subjects will repeat the progressive increase of daily duration of the Viaskin® Peanut 250 µg patches application as at the start of the study. Some subjects may have a higher skin reactivity at the initiation of the treatment, leading to uncomfortable sensations or intense pruritus/itching. These local reactions usually decrease over time as far as the treatment continues. However, some subjects, may have difficulties in bearing these uncomfortable sensations or intense pruritus/itching. In these subjects, it is recommended to remove the patch as soon as the subject starts complaining, to clean and to wash the skin area and if necessary use topical corticosteroids. The next patch the following day will be applied in the next skin zone as scheduled. The duration of patch application could be more progressively increased, up to the final targeted

24 hours of patch application; therefore, the treatment initiation period could be longer than the 2 weeks proposed above.

If possible, the subjects should take advantage of their shower/bath time to change the patch. The previous Viaskin® patch should be removed just before the shower/bath, and the new Viaskin® patch should be applied a few minutes after the shower/bath and after carefully drying the skin. Application of the Viaskin® patch at a similar time for each daily application (morning or evening) is recommended. If the subject does not bathe daily or take shower at the same time daily, it is recommended that the zone to which the patch will be applied be cleaned with a moist disposable napkin or tissue and carefully dried prior to application.

In case a patch comes off, it should be immediately discarded. If a patch comes off within 2 hours after being applied, a new replacement patch can be applied the same day. If a patch comes off more than 2 hours after being applied, a replacement patch should not be applied the same day – a new patch will be applied the next day at the usual time.

In case a patch is worn more than the 24 + 4 hours, the recommendation is to apply a new patch until the next planned time of patch renewal. This new patch will consequently be applied less than 24h.

In case the child has experienced allergic reaction(s) after accidental ingestion of peanut-containing food, the new patch application(s) must be skipped until these allergic symptoms are resolved. After the Viaskin® patch comes off, or after removing a Viaskin® patch to apply a new one, it is important to wipe the zone with a moist disposable napkin or a moist disposable tissue and then to wash hands to prevent accidental manual transmission of peanut proteins. A Viaskin® patch already used must not be re-applied.

After the first application of the Viaskin® patch at the study site, all subjects will be monitored for 3 hours before being discharged in order to check and grade any reactions under or around the patch and a picture will be taken to document the application site whether there is a local reaction or not. The same procedure will be followed with all the subjects at the Visit 6 when the open label Viaskin® Peanut 250 μg patches will be assigned to all subjects.

Because the study includes children aged 4-5 years, additional safety measures have been adopted and generalized to all ages. On Day 4 and on Day 22, specific phone contacts will be made to the parents/guardians to assess the safety of patch applications on the back. If required, the subjects may need to come to the site sooner than planned to be seen and evaluated by the Investigator.

Of note, the recommended duration of daily Viaskin® patch application is 24 hours of application per day. However, any daily duration of Viaskin® patch application of 24 hours ±4 hours will be allowed.

5.2.1. Adjustment of Viaskin® Patch Application in Case of Local or Systemic Reactions

When subjects are unable to apply the Viaskin® patch for the recommended durations as described above because of local intense or severe reactions, under or adjacent to the patch site, the patch should be removed immediately, the site of application wiped with a moist disposable tissue and a topical corticosteroid medication might be topically applied to treat the local reactions. The parents/guardians should take a photo of the back of the subject to document how intense and extended the local reactions are. In the specific case of local intense or severe reactions, it is mandatory that the next Viaskin® patch is applied only the next day on the next zone; no other

Viaskin® patch must be applied the same day. In case of re-appearance of the local intense or severe reactions after application of the next patch the following day, the patch must again be removed. The next Viaskin® patch should be applied only the next day on the next zone. The same process is repeated every day after patch application in case of local intense or severe reactions.
As a consequence, the daily duration of Viaskin® patch application should be adjusted/reduced as necessary, and subjects may need more than the 14 days previously described before they can apply and tolerate the Viaskin® patch for the full 24 hours daily.
In case of any suspected systemic reactions related to patch application (including cutaneous reactions distant from the sites of patch application), the same safety precautions should be followed: the Viaskin® patch is removed immediately, the site of application wiped with a moist disposable tissue and a treatment can be given to treat the reaction: antihistamines or topical corticosteroids with additional oral corticosteroids or similar anti-allergic drugs, as deemed necessary by the Investigator. The next Viaskin® patch should be applied the following day on the next zone. As a consequence, the daily duration of patch application should be adjusted/reduced as necessary, and subjects may need more time than initially defined before they can apply the Viaskin® patch for the full 24 hours daily.

Appearance of any vesicles (grade 4) or ulcerative skin lesions or any other significant or unexpected skin lesion seen under the patch while the patch is still applied on the skin or upon removal of the patch and lasting more than 1 day which could potentially lead to skin barrier disruption at sites of Viaskin® patch applications will be cautiously managed and follow-up.
Children's parents or guardians will be instructed to take a photograph in case of appearance of any vesicles or ulcerative skin lesions. In these rare specific cases, subjects should transiently discontinue patch application. The subject's parents/guardians will be instructed to return to the site for evaluation and treatment of the wounded area, as well as for the next patch application and adequate evaluation and treatment of the wounded zone.
Upon re-application of the new patch, the subject should remain at the site for 1 hour before being discharged. The site must contact the subject's parents/guardians by phone the day after this visit to ensure that no additional local blisters or vesicles developed and to confirm that the treatment can continue normally.
In case the child has experienced allergic reaction(s) after accidental ingestion of peanut containing-food, the new patch application(s) must be skipped until these allergic symptoms are resolved.

5.2.2. Safety Precaution Information

A leaflet with general instructions for use, safety precautions for using the Viaskin® patch and instructions to follow in case of any safety issue will be given to each subject and his/her parents/guardians. This safety leaflet will specify at least the following information:

1. Instructions and procedures to apply the patch safely and correctly on the back of the subject;
2. Necessity to call the investigational site staff in case of intense or severe local reactions lasting for more than 1 day or any unexpected reactions during the treatment period, in particular any presence of vesicles/blisters under the patch or close to the area of patch

application. Instruction to take photos of the site of application will be made to the parents for documenting such a situation;

3. Necessity to call the investigational site staff in case of occurrence of chicken pox or measles. At the same time, the patch application should be discontinued until recovery;
4. In case of active eczema extending on the back of the child: stop applying the patch until recovery from the active eczema on the back;
5. In case of a suspected anaphylactic reaction and how to administer the epinephrine auto-injector to rapidly treat the reaction;
6. Subject stopping rules (subject are consequently withdrawn):
   a. Severe “maculo-papular rash” or severe “dermatitis” at sites of patch application not controlled by topical corticosteroids or adaptation of the application duration of the patches,
   b. Severe anaphylaxis (stage 3 anaphylaxis, see APPENDIX 5) related to Viaskin® patch application.
   c. More than 1 epinephrine injection for an AE related to Viaskin® patch application (not occurring during the food challenge).

5.3. Packaging, Labeling and Storage

The study drug (active and placebo) will be packaged and labeled by CREAPHARM (Bordeaux, France) in accordance with applicable local regulatory requirements. One Viaskin® patch will be placed per pouch and each pouch will be labeled. The labeled pouches will be placed in labeled treatment boxes to be dispensed to subjects at each visit, with enough quantity of Viaskin® patches to cover the period between 2 consecutive visits.

The labeled and packaged study drug must be stored in accordance with the Sponsor’s instructions (below 25°C [77°F] and should not be frozen). Shipments from depots to clinical sites will be performed at refrigerated temperature between 2°C to 8°C [36°F to 46°F] with a temperature monitoring device. Storage at ambient temperature for short and/or intermittent periods of time, including transportation from the clinical site to the subject’s home, is permitted.

Upon receipt of a shipment request, the study drug will be shipped to the clinical site. The site pharmacist or any other staff member designated for this task will receive and store the study drug until the time of dispensing it to the subject. The study drug will be stored in a securely locked area, accessible to authorized personnel only. At the end of the study, or at times designated by the Sponsor, and after complete accountability, the site pharmacist or the designated person will be responsible for preparing the return of the unused study drug to the study drug distributor. Destruction of study drug on-site should be avoided. However, in some cases, and upon authorization from the Sponsor, destroying the unused study drug on-site, with provision of a corresponding certificate of destruction, may be allowed.

5.4. Blinding and Breaking the Blind

During the first 6 months, the study will be performed in a double-blind manner. Active and placebo Viaskin® patches will be supplied in identical pouches and will be similar in physical appearance, thereby enabling double-blind conditions. The treatment codes will be held by the

IWRS. Further instructions for emergency code break will be provided in a separate IWRS User Guide.

The study blind should not be broken except in a medical emergency (where knowledge of the study drug received would affect the treatment of the emergency) or due to a regulatory requirement (for example for suspected unexpected serious adverse reactions). The blind will only be broken at the discretion of the Investigator, and if possible, following discussion on a case-by-case basis with the Sponsor/Medical Monitor. All calls resulting in an unblinding event will be recorded and reported by the IWRS to the Medical Monitor and the Sponsor.

In case of IWRS failure (both IWRS system and IWRS hotline unavailable), a third-line backup system is operated 24 hours every day of the year by the Sponsor enabling unblinding of treatment by calling the following universal toll free DBV Technologies numbers:

Canada and USA:...1 844 299 0837

If the blind is broken during the first 6 months, the date, time, and reason must be recorded in the subject's source documents, in the e-CRF and any associated AE must be reported.

If an Investigator, site personnel performing assessments, or subject (parents/guardians), is unblinded, the subject will perform the Month-6 visit and continue the open label part of the study.

Serious unexpected suspected adverse reactions occurring during the first 6-month double-blind period, which are subject to expedited reporting, will be unblinded before submission to the concerned Regulatory Authorities.

The overall randomization codes will only be opened and released after Month 6 database will be locked, and the 6-months safety results released.

After these first 6 months, the study will be conducted in an open label design. All subjects will receive Viaskin® Peanut 250 µg treatment, knowing that the former placebo subjects would have crossed-over to receive this active treatment at the end of the Month 6 visit.

5.5. Drug Accountability

The Investigator is responsible for maintaining accurate study drug accountability records throughout the study. Each site will have to complete a site study drug accountability log and an individual study drug accountability log for each subject. These records should include the amounts and dates that study drug supplies were received on-site, dispensed to the subject, returned by the subject, and returned to study drug distributor (or destroyed on-site, if applicable). Any discrepancy must be documented.

Each dispensing of study drug will be documented in the e-CRF and IWRS (data integration).

5.6. Compliance

It is the Investigators' responsibility to ensure that subjects (parents/guardians) are correctly instructed on how to store and administer the study drug. The study drug should be dispensed by the Investigator, or by a qualified individual under the Investigator's supervision. An up-to-date study drug accountability log must be maintained (see Section 5.5). Records of study drug used and intervals between visits will be kept during the study. Drug accountability will be monitored

by the monitor during site monitoring visits and at the completion of the study. Subjects (parents/guardians) will be asked to return empty boxes and their unused study drug (boxes containing remaining patches) when they come back for their study visits. All unused study drug should be returned by the end of the study to the study drug distributor.

At each visit, prior to dispensing the study drug, previously dispensed study drug will be retrieved by the Investigator, the compliance will be assessed and documented. A global compliance of ≥80% over the whole treatment period is sought. Global compliance is defined as the total number of patches applied in the treatment period *versus* the number of days in that period of time. Compliance at each visit is to be calculated taking into account the total number of patches applied since the last visit *versus* the number of days in that period of time. Subjects exhibiting poor compliance (below 80%) should be reminded of the importance of good compliance to the study dosing regimen.

Subjects who are persistently non-compliant with the study treatment will be withdrawn from the study (see Section 4.4).

5.7. Prior and Concomitant Medications

Any medication the subject takes other than the study drug, including herbal and other non-traditional remedies, is considered as concomitant medication. Any medication used in the last 6 months or being administered at the time of screening is considered as prior medication.

At screening, subjects will be asked what medications they have been taking for the last 6 months. At each subsequent study visit, subjects will be asked what concomitant medications they are currently taking. All concomitant and prior medications must be recorded in the e-CRF.

The following information must be recorded in the e-CRF for each prior and concomitant medication: generic name, route of administration, start date, stop date, dosage, total daily dose, and indication. Any changes in the dosage or regimen of a concomitant medication must be recorded in the e-CRF.

Of note, prior or concomitant medication prescribed to a subject but not actually taken or administered should not be reported in the e-CRF, such as the epinephrine auto-injector prescribed for use in case of anaphylaxis but never injected intramuscularly.

5.7.1. Permitted Concomitant Medications

Application of a topical corticosteroid and topical calcineurin inhibitors to treat any local condition (eczematous lesions, pruritus, edema, etc.) is allowed and should be recorded as concomitant medication. For local reactions to the Viaskin® patch, a topical medication (ointment, gel, cream) with 1% hydrocortisone or equivalent will be distributed to each included subject at discharge on Day 1. In case the 1% hydrocortisone topical medication is not sufficient to treat the local reaction, a topical medication containing a more potent corticosteroid can be prescribed and locally applied. Oral antihistamines or oral corticosteroids are allowed to treat conditions determined as being allergic reactions and should be recorded as concomitant medications. These treatments should be limited in duration and stopped as soon as the condition has resolved. The Investigator will determine the best choice of treatment, the dose and the regimen according to the subject's age,

and the type and the degree of severity of the reactions. Cetirizine is recommended as the oral antihistamine of choice.

Intramuscularly injectable epinephrine (any auto-injector trade name available at the right dosage in the different countries) will be distributed to each subject at discharge on Day 1 to be used in case of symptoms of anaphylaxis. The Investigator will explain to the subject/parents/guardians when and how to (self) inject the epinephrine according to the Anaphylaxis Emergency Action Plan which will also be given to the subject. The epinephrine auto-injector will be replaced if it is used or if it expires. Any used epinephrine auto-injector should be recorded as a concomitant medication.

All other treatments prescribed by the Investigator or any other physician to treat any conditions are permitted except those prohibited in the Section 5.7.2.

5.7.2. Prohibited Prior and Concomitant Medications

Prohibited prior and concomitant medications as outlined in the exclusion criteria (Section 5.7) are the following:

- Short-acting antihistamines or long-acting antihistamines taken must be washed out for the minimum period of time prior to the skin prick testing or food challenges as specified in the table in APPENDIX 4;
- Treatment with a high daily dose of inhaled corticosteroid or treatment with a combination therapy of a medium daily dose of inhaled corticosteroid with a long-acting inhaled β2-agonist or treatment with a combination therapy of a high daily dose of inhaled corticosteroid with a long-acting inhaled β2-agonist (a list of dosages of inhaled corticosteroids is provided APPENDIX 3). Two or more systemic corticosteroid courses for asthma taken in the year prior to Visit 1 or 1 oral corticosteroid course for asthma taken within 3 months prior to Visit 1, or during screening period (unless used to treat symptoms triggered by a food challenge);
- β-blocking agents, angiotensin-converting enzyme inhibitors, angiotensin-receptor blockers, calcium channel blockers or tricyclic antidepressant therapy, during the screening period or during study participation;
- Anti-tumor necrosis factor drugs or anti-IgE drugs (such as omalizumab) or any biologic immunomodulatory therapy taken within 1 year prior to Visit 1, during screening period or during study participation. Topical calcineurin inhibitors are permitted;
- Cyclosporine or other immunosuppressive agents used within 1 year prior to Visit 1, during the screening period or during the study participation.;
- Systemic long-acting corticosteroids used within 12 weeks prior to Visit 1 and/or systemic short-acting corticosteroids used within 4 weeks prior to Visit 1 or during screening (unless used for food challenge symptoms);
- Any prior or concomitant immunotherapy administered to any food (for example EPIT or OIT or SLIT or specific oral tolerance induction);
- Any aeroallergen immunotherapy administered during study participation.

6. VARIABLES AND METHODS OF ASSESSMENT

The safety and exploratory endpoints are listed in Section 3.3. The Schedule of Procedures is provided in Table 9.

6.1. Demographics and Baseline Characteristics

Demographics and baseline characteristics consist of those variables that are assessed only at screening/baseline.

6.1.1. Subjects Demography

Subject demography consists of:

- Age at screening Visit 1,
- Race/Ethnic origin,
- Sex.

6.1.2. Disease History and Medical History

The disease history will be exhaustively documented. Diagnosis of peanut allergy will be based on a well-documented medical history of IgE-mediated reactions after ingestion of peanut, i.e. allergy reaction(s), having led to an emergency department visit or a physician consultation with or without history of positive peanut-food challenge. These allergic event(s) should have occurred and been reported after 2 years of age. History of severe anaphylaxis to peanut should clearly be identified and described in the disease history. Previously performed allergy diagnosis tests (peanut specific IgE, SPT and food challenges), most recent allergic reaction(s) related to peanut consumption, severity and treatment and eviction diet, history and duration of any food allergies (other than peanut), or any other allergies, will be reported.

The documentation of the complete medical history will include the other current medical conditions, past or present cardiovascular, respiratory (including asthma), gastrointestinal, renal, hepatic, neurological, endocrine, lymphatic, hematologic, immunologic, dermatological (including atopy), psychiatric, developmental, and genitourinary disorders, drug and surgical history and any other diseases or disorders.

The disease and medical histories will be obtained by interviewing the subject's parents/guardians or by reviewing her/his medical records.

For coding of disease/medical history, see Section 9.4.

6.1.3. Prior and Concomitant Medications

Previous and concomitant medications will be documented as described in Section 5.7.

6.2. Safety Variables

6.2.1. Adverse Events

Coding of AEs will be performed as described in Section 9.4.

6.2.1.1. Collection of Adverse Events

It is the responsibility of the Investigator to collect all AEs (both serious and non-serious) derived by spontaneous, unsolicited reports of subjects, by observation and by routine open questionings, for example "How have you felt since I last saw you?".

6.2.1.2. Definitions

An AE is any untoward medical-occurrence that occurs in a subject or clinical investigation subject administered a pharmaceutical product, and which does not necessarily have to have a causal relationship with this treatment. An AE can therefore be any unfavorable and unintended sign (including abnormal laboratory finding), symptom, or disease temporally associated with the use of an investigational product, whether or not considered related to the product.

All AEs, including intercurrent illnesses, occurring during the study will be documented in the e-CRF. Concomitant illnesses, which existed before entry into the study, will not be considered AEs unless they worsen during the treatment period. All AEs, regardless of the source of identification (for example physical examination, laboratory assessment, electrocardiogram, reported by subject), must be documented.

Pre-existing conditions will be recorded in the e-CRF on the Medical History or appropriate page.

A TEAE will be defined as an AE that begins or that worsens in severity after at least 1 dose of study drug has been administered.

A pretreatment-emergent AE will be defined as an AE that begins or that worsens in severity after the first screening visit, but before the first dose of study drug has been administered.

In case of food challenge performance, the symptoms elicited by this challenge will also be recorded in the e-CRF but will be analyzed separately.

6.2.1.3. Assessment of Adverse Events

Each AE will be assessed by the Investigator with regard to the following categories.

6.2.1.4. Seriousness

An SAE is defined as any untoward medical occurrence that at any dose:

- Results in death;
- Is life-threatening; This means that the subject is at risk of death at the time of the event; it does not mean that the event hypothetically might have caused death if it were more severe;
- Requires hospitalization (overnight or longer) or prolongation of existing hospitalization;
- Results in persistent or significant disability or incapacity;
- Is a congenital anomaly or birth defect;
- Is an important medical event that may not be immediately life-threatening or result in death or hospitalization but that may jeopardize the subject or require intervention to prevent one of the above outcomes. Examples of such events are intensive treatment in an emergency room or at home for allergic bronchospasm; blood dyscrasias or convulsions that do not result in hospitalization; or development of drug dependency or drug abuse.

Medical and scientific judgment should be exercised in deciding whether a case is serious and whether expedited reporting is appropriate.

Events associated with hospitalization for the following will not be considered as an SAE:

1. Evaluation or treatment of a pre-existing and non-exacerbating condition as long as the condition is associated with the hospitalization:
    a. Existed prior to the subject's entry into the study and has been recorded in the subject's disease/medical history as documented in the e-CRF,
    b. Has not worsened in severity or frequency during the subject's exposure to study drug,
    c. Has not required a change in treatment management during the subject's exposure to the study drug;
2. Elective or pre-planned treatment of a pre-existing and non-exacerbating condition;
3. Hospitalization of a subject the day of or the day prior to the day of food challenge performance.

6.2.1.5. Severity

The severity of each AE must be assessed by the Investigator using 1 of the following categories, and recorded in the e-CRF:

- Mild: the AE was transient and easily tolerated by the subject;
- Moderate: the AE caused discomfort and interference with the subject's general condition;
- Severe: the AE caused considerable interference with the subject's general condition and may have been incapacitating.

6.2.1.6. Causality

The Investigator will assess the causality/relationship between the study drug and the AE and record that assessment in the source documents and in the e-CRF.

The most likely cause of an AE/SAE (for example disease under treatment, concomitant disease, concomitant medication, other) will be indicated in the e-CRF with details of the concomitant disease or medication or other cause.

The causal relationship of the AE to study drug will be described in terms of:

- Related, the AE:
    - follows a clear temporal sequence from administration of the study drug,
    - has no other possible explanations, such as the subject's clinical state, environmental or toxic factors or other therapies administrated to the subject,
    - disappears or decreases on cessation or reduction in dose of the study drug,
    - follows a clear pattern of response to the study drug,
    - reappears or worsens upon re-challenge.
- Probable, the AE:
    - follows a reasonable temporal sequence from administration of the study drug,
    - could not be reasonably explained by the subject's clinical state, environmental or toxic factors or other therapies administrated to the subject,
    - disappears or decreases on cessation or reduction in dose of the study drug,
    - follows a known pattern of response to the study drug,
    - reappears or worsens upon re-challenge.
- Possible, the AE:
    - follows a reasonable temporal sequence from administration of the study drug,

  - could be reasonably explained by the subject's clinical state, environmental or toxic factors or other therapies administrated to the subject,
  - follows a known pattern of response to the study drug.
- Unlikely, the AE;
  - does not follow a reasonable temporal sequence from administration of the study drug,
  - could be reasonably explained by the subject's clinical state, environmental or toxic factors or other therapies administrated to the subject,
  - does not follow a known pattern of response to the study drug,
  - does not reappear or worsen upon re-challenge.
- Not related:
  - the AE does not meet the above criteria,
  - there is sufficient information that the etiology of the AE is not related to the study drug.

The study conduct relatedness for SAEs will also be assessed and documented. Unlikely related AEs will be considered as not related to the study drug.

6.2.1.7. Local Skin Reactions

The incidence, duration and severity of skin reactions induced by the study drug will be reported by the subject (parents/guardians) in the diary. Photos of these local reactions should be taken at home by the parents/guardians. Additionally, the Investigator will assess the severity of local skin reactions induced by the study drug at each visit during the physical examination and photos should be taken during the site visits by the medical staff to document these local skin reactions, especially when skin reactions are graded 3 or 4, or led to subject treatment discontinuation or were noticeable in the opinion of the investigators (see Sections 6.2.6 and 6.2.10).

During the first 6 months, 3 pre-specified symptoms (itching, redness, swelling) will be reported on a daily basis in the diaries. These 3 pre-specified solicited symptoms will not be reported by the investigators in the AE pages of the e-CRF, except if these symptoms are part of another concomitant disease or if these symptoms are leading to the subject's study discontinuation or serious AEs.

After month 6, any AE, including the 3 above symptoms, other local skin reactions or any other type of AE will be spontaneously reported in the dedicated section of the diaries. These AEs will be reported by the investigators in the e-CRF.

6.2.1.8. Adverse Events of Special Interest

Adverse events of special interest (AESI) to Sponsor in this study include grade 4 local skin reactions seen under the patch while the patch is still applied on the skin or upon removal of the patch from the skin. Skin reactions will be examined at the time points specified in the Schedule of Procedures Table 9 according to grading in Table 8 (Section 6.2.10). Specifically, the appearance of any vesicles or ulcerative skin lesions or any other significant skin lesion which could potentially lead to skin barrier disruption at sites of Viaskin® patch applications will be considered AESI. In rare case of appearance of any vesicles or ulcerative skin lesions, subjects should transiently discontinue patch application and return to the site for evaluation and treatment

of the wounded area until resolution, as well as for the next patch application and adequate evaluation and treatment of the wounded zone.

Upon re-application of the new patch, the subject should remain at the site for 1 hour before being discharged. The site must contact the subject (parents/guardians) by phone the day after this visit to ensure that no additional local blisters or vesicles developed and to confirm that the treatment can continue normally.

Any occurrence of IgE-mediated systemic symptoms distant from the patch application site and considered at least possibly related to the study drug will also be considered as an AESI and specifically analyzed.

6.2.1.9. Symptoms during Food Challenge(s) performance

The severity of the objective and subjective symptoms elicited during food challenges that may be performed during the study for the subject medical management will be assessed by the Investigator. These objective and subjective symptoms will not be reported in the AE pages of the e-CRF. Complete information for all these symptoms will be reported in the FC pages of the e-CRF, along with all peanut doses given to the subject, the description of the observed symptoms and their highest severity grade(s), time of appearance of the symptoms, the doses of corticosteroids and antihistamines given or the doses of epinephrine given. The Investigator's assessment of the peanut ED as well as the peanut cumulative dose will be systematically reported. If, as per the Investigator's judgment, the combination of all these symptoms resulted in an anaphylaxis requiring a prolonged (minimally overnight) hospitalization, then this will be considered as an SAE. Any such SAE with its specific verbatim must be reported in the AE pages of the e-CRF.

6.2.1.10. Recording Adverse Events

Adverse event reporting will start at the signature of the informed consent form (ICF) and will end at the End of Study Visit (or Early Termination Visit). Adverse events occurring after the end of the study will only be reported to the Sponsor by the Investigator if the Investigator considers that there is a causal relationship with the study drug.

Adverse events still ongoing at the time of the End of Study Visit will be followed up for an additional 30 days, or until they resolve or stabilize, whichever comes first.

All AE reports in the e-CRF should contain the following information of the event: date and time of onset, date and time of resolution, severity, treatment required, relationship to study drug, action taken with the study drug, outcome, and whether the event is classified as serious or not.

6.2.1.11. Reporting Serious Adverse Events

All SAEs must be reported within 24 hours from the time the site becomes aware of the event by filling in the SAE pages in the e-CRF. In case of technical issues with the e-CRF, the reporting can be done by faxing a completed SAE Report Form to the PAREXEL safety center at the following FAX numbers:

- Centers in North America dial the following FAX number:
  +1 781 434 5957

The minimum information required for an initial report is:

- Name of person sending the report (that is name and address of Investigator);
- Subject identification (screening number, NOT the subject's name);
- Protocol number;
- Description of the SAE including a comprehensive verbatim term;
- Causality assessment, if possible.

However, as far as possible all information requested in the SAE pages in the e-CRF (or on the SAE Report Form, in case of technical issues with the e-CRF) should be covered in the initial report. If an SAE occurs during a FC, the verbatim of the SAE must specify "during the FC" or "due to the FC" both on the SAE Report Form and in the SAE pages of the e-CRF.

6.2.1.12. Follow-up of Adverse Events

All AEs experienced by a subject, irrespective of the suspected causality, will be monitored until the AE has resolved, any abnormal laboratory values have returned to baseline or stabilized at a level acceptable to the Investigator and Medical Monitor, until there is a satisfactory explanation for the changes observed, excepted when the subject is lost to follow-up, or the subject has died.

6.2.2. Pregnancy Test

Pregnancy will be determined by evaluation of urine pregnancy tests. Subjects who are pregnant at screening are excluded from the study. Subjects who become pregnant during treatment must be discontinued from the study. Pregnancy test will be performed at month 6, 12, 24, 36 for all subjects and at month 42 for subjects initially randomized in the placebo group and could be performed at any visit if a possible pregnancy is suspected.

The Sponsor has a responsibility to monitor the outcome of pregnancies where there has been maternal exposure to the study drug.

Pregnancy alone is not regarded as an AE unless there is a suspicion that the study drug may have interfered with the effectiveness of a contraceptive medication.

Elective abortions without complications should not be handled as AEs, unless they were therapeutic abortions (see below). Hospitalization for normal delivery of a healthy newborn will not be considered a SAE.

All pregnancies must be reported by the Investigator to PAREXEL/Sponsor in the specific pregnancy pages in the e-CRF within 24 hours after becoming aware of the pregnancy. The Investigator must follow up and document the course and the outcome of all pregnancies even if the subject was discontinued from the study or if the study has finished.

All outcomes of pregnancy must be reported by the Investigator to PAREXEL/Sponsor in the specific pregnancy pages in the e-CRF within 24 hours after she/he has gained knowledge of the normal delivery or elective abortion.

Any SAE that occurs during pregnancy (including SAEs occurring after last administration of study drug) must be recorded in the SAE pages in the e-CRF (for example maternal serious complications, spontaneous or therapeutic abortion, ectopic pregnancy, stillbirth, neonatal death, congenital anomaly, or birth defect) and reported within 24 hours in accordance with the procedure for reporting SAEs.

6.2.3. Treatment of Overdose of Study Medication

Overdose is defined as the concomitant application of 2 Viaskin® patches or more on the skin of the subject, whatever the duration of the concomitance of the multiple patch applications.
One patch applied the same day after the previous patch was removed or has fallen off is NOT an overdose.
There has been no experience of overdosing with Viaskin® patches so far. No specific treatment for overdosing is known. The first action will be to remove any additional patch from the skin, leaving only 1 patch on the skin. Treatment given to a subject in case of overdosing should be symptomatic and supportive.
Any case of overdose, with or without associated AEs, must be reported to PAREXEL. Overdose will be reported in the SAE pages of the e-CRF (with Overdose as event term) within 24 hours of learning of the overdose. In case of technical issues with the e-CRF, this can be done by faxing a completed SAE Report Form to the PAREXEL safety center. Any AEs associated with the overdose should be reported in the relevant AE/SAE sections in the e-CRF.

6.2.4. Laboratory Variables

Laboratory assessments will be performed by a central laboratory, as identified in the List of Study Personnel.

Venous blood samples will be taken for hematology and biochemistry testing.

The following laboratory variables will be determined in accordance with the Schedule of Procedures (Table 9):

- Hematology: hemoglobin, hematocrit, platelets, red blood cells, white blood cells with differential cell count;
- Biochemistry: alanine aminotransferase, aspartate aminotransferase, total bilirubin, total protein, blood urea nitrogen, creatinine;
- Urine pregnancy tests (for females of childbearing potential) will be performed at the site.

Details of the procedures to be followed for sample collection, storage, and shipment will be documented in the Laboratory Manual.
Clinically significant changes (abnormalities) in laboratory parameters, in the judgment of the Investigator, will be recorded as AEs and appropriate countermeasures taken.
In the event of unexplained abnormal laboratory test values of clinical significance, the tests should be repeated at a reasonable time point and followed up until they have returned to the normal range and/or an adequate explanation of the abnormality is found.
Additional and repeat laboratory safety testing outside the study may be performed at the discretion of the Investigator.

6.2.5. Vital Signs

The following vital signs will be assessed in accordance with the Schedule of Procedures (Table 9),

- Blood pressure (systolic and diastolic; mmHg),
- Heart rate (beats per minute),
- Respiration rate (breaths per minute).

Systolic blood pressure and diastolic blood pressure will be measured in sitting position on the same arm after the subject has been resting.
Heart rate will be recorded simultaneously with blood pressure measurements, followed by respiratory rate.
During the study, the measurement of vital signs may be repeated at the discretion of the Investigator for safety reasons. Clinically relevant abnormal findings will be reported as AEs.

6.2.6. Physical Examinations

Physical examinations will be performed in accordance with the Schedule of Procedures (Table 9). Physical examinations will be performed by a physician or a master level clinician qualified for performing physical examinations (Nurse Practitioners or physician assistants) and will include examination of the following:

- General appearance,
- Body weight (kg),
- Height (cm),
- Complete skin examination,
- Head, ears, eyes, nose and throat,
- Cardiovascular system,
- Respiratory system,
- Abdominal system,
- Nervous system,

Body weight will be measured without shoes, jacket, or diaper and per Schedule of Procedures and as frequently as necessary.
Height will be measured without shoes and per Schedule of Procedures and as frequently as necessary for this population of subjects in active growth. This also applies for the pulmonary function tests (spirometry, PEF measurements).
For each body system, the assessment is made as normal or abnormal and will be recorded in the e-CRF at screening and the abnormality will be documented. Besides, the skin aspect will be graded for the sites of patch application (see Section 6.2.10).
Clinically significant changes (abnormalities) in physical examination findings, in the judgment of the Investigator, will be recorded as AEs and appropriate countermeasures taken.

6.2.7. Spirometry Test

Forced expiratory flow in 1 second (FEV1) will be measured on a standardized calibrated spirometer following the ATS guidelines. At least 3 acceptable FEV1 maneuvers will be obtained and the highest of the 3 attempts will be recorded in the e-CRF. Subjects from 6 to 8 years of age who have documented inability to adequately perform spirometry can only perform the PEF measurement. Spirometry will be systematically performed at baseline and Month 6, 12, 18, 24, 30, 36 for all subjects and Month 42 for subjects initially in the placebo group. For children reaching 6 years old during the study, the spirometry will be performed after turning 6-year-old.

6.2.8. *Peak Expiratory Flow*

For all the subjects ≥5 years of age, PEF will be measured on a peak flow meter. At least 3 acceptable measures will be obtained and the highest of the 3 attempts will be reported in the e-CRF. The PEF assessments will be performed at Baseline, Day 1, Day 8, Month1, Month 3, Month 6, Month 7, 9, 12 ,18, 24, 30, 36 for all subjects and at Month 42 for subjects initially in the placebo group. For children reaching 5 years old during the study, the PEF will be performed after turning 5-year-old.

6.2.9. *Subject Diaries*

Subject diaries will be provided to each subject to report specific information between visits. Subjects and/or their parents/guardians will be asked to record on a daily basis in their diaries the time of application and removal of each Viaskin® patch, along with the reason for any early removal (should that occur). During the first 6 months, subjects (parents/guardians) will also be asked to daily record pre-specified local skin reactions (local itching, redness and swelling) and grade them (grading scale 0 to 3: none, mild, moderate or severe).

Definitions of the grading of each symptom are provided in the diary:

Grade Local	Itching Local	Redness Local	Swelling
1 = Mild	Itching easily bearable, painless, occasional scratching localized under the Viaskin patch areas.	Mild redness (pale pink) localized under the Viaskin patch areas.	Swelling localized under the Viaskin patch areas.
2 = Moderate	Itching with intermittent scratching, possibly up to 2 min each localized under the Viaskin patch areas.	Redness more intense (brighter pink to red), and/or redness extending beyond the Viaskin patch areas.	Swelling extending beyond the Viaskin patch areas.
3 = Severe	Spreading intense itching resulting in continuous scratching unbearable, interfering with daily activities. Requiring treatment.	Very intense redness/rash and/or redness largely extending beyond the Viaskin patch areas.	Large swelling area extending beyond the Viaskin patch areas.

Subjects (parents/guardians) will also be instructed to use the diaries as reminder tool and to report any other AEs or local skin reactions and any concomitant medication or treatment taken.

The subject diary will be reviewed by the site medical staff at each subject visit. During the first 6 months, 3 pre-specified symptoms (itching, redness, swelling) will be reported on a daily basis in the diaries. These 3 pre-specified solicited symptoms will not be reported by the investigators in the AE pages of the e-CRF, except if these symptoms are part of another concomitant disease or if these symptoms are leading to the subject's study discontinuation or serious AEs. Any other local skin reactions or any other type of adverse events will be spontaneously reported in the diaries and will be reported by the investigators in the AE e-CRF form. After month 6, any AE, including the 3 above symptoms, other local skin reactions or any other type of AE will be spontaneously reported in the dedicated section of the diaries. These AEs will be reported by the investigators in the e-CRF.

Subjects must bring their diaries back to the Investigator at each visit, and the Investigator must check the diary for completeness and accuracy. It is the Investigators' responsibility to instruct the subjects and/or parents/guardians about the use of the diary, and to ensure that the diary is accurately completed. Any problems with completing the diary will be addressed with the subjects and/or parents/guardians. At each site visit, the completed pages of the diary will be collected and kept by the Investigators. The remaining blank pages of the diary will be given back to the subject at each visit before discharge. All diaries must be returned to the site at completion of the study or if the subject discontinues.

6.2.10. Skin Reaction and Photography

Local skin reactions under the Viaskin® patch or on any of the previous sites of patch application will be graded as a whole at each visit according to the recommendations of the European Academy of Allergy and Clinical Immunology (EAACI) and the Global Allergy and Asthma European Network (GA^{2}LEN) [37] , and modified as follows:

Table 8: Skin Reaction Grading System

**Skin Reaction**	**Grade if localized under the patch**	**Grade if extending beyond the patch**
Negative	Grade 0	Grade 0
Only erythema, or erythema + infiltration	Grade 1A	Grade 1B
Erythema, few papules	Grade 2A	Grade 2B
Erythema, many or spreading papules	Grade 3A	Grade 3B
Erythema, vesicles	Grade 4A	Grade 4B

The Viaskin® patch is transparent and the degree of the local reactions under the Viaskin® patch can be easily seen through the patch. Grading of the local skin reactions on the back will start being recorded at Visit 2 right after the Viaskin® patch has been applied and while the subject is kept at site under observation and subsequently when subjects arrive at site for their further visits.

At Visit 2, observations should be recorded at 30 minutes, 1 hour, 2 hours, and 3 hours after the first Viaskin® patch is applied (through the patch, without removing it).
When skin reactions are graded 3 or 4, or lead to subject treatment discontinuation or are noticeable in the opinion of the investigators, photos should be taken during the visits for documentation.
At Visit 6 the same procedure will be followed with the application in the investigator's site of the first open label Viaskin® Peanut 250 µg patch. Subjects will be kept and observed again for 3 hours.
Photos of skin reactions may also be requested by the site staff during a phone contact with the subject's parents to document these reactions between 2 site visits.

Appearance of any vesicles (grade 4) or ulcerative skin lesions or any other significant skin lesion seen under the patch while the patch is still applied on the skin or upon removal of the patch which could potentially lead to skin barrier disruption at sites of Viaskin® patch applications will be cautiously managed and follow-up.

Children's parents or guardians will be instructed to take a photograph of the patch skin area at home in case of appearance of any severe skin reaction, including vesicles or ulcerative skin lesions. In these rare cases, subjects should transiently discontinue the patch applications and

return to the site for evaluation and treatment of the wounded area, as well as for the next patch application and adequate evaluation and treatment of the wounded zone.

Upon re-application of the new patch, the subject should remain at the site for 1 hour before being discharged. The application duration may be temporarily adapted if deemed necessary. The site must contact the subject's parents/guardians by phone the day after this visit to ensure that no additional local blisters or vesicles developed and to confirm that the treatment can continue normally.

Photographic records of the Viaskin® patch sites of application will be taken and filed in the subject's medical records or source documents. The subject's face will not, at any time, be captured in the photograph. Photography acquisition guidelines will be provided separately to the sites, so that photography process can be standardized as much as possible. The anonymized photographs will be available for documenting the skin reactions and will be downloaded by the site staff onto a specific website with a restrictive access to the site staff members, the Sponsor members and Sponsor representatives.
Photographic records will be made available to the DSMB members upon request.

6.3. Exploratory Variables

6.3.1. Immunological Markers

Venous blood samples will be drawn to assess the following immunological markers: peanut-specific IgE and IgG4 at the visits specified in the Schedule of Procedures (Table 9).
The following immunological markers will also be assessed at specific time points: IgE and IgG4 specific to peanut protein components, and IgE specific to cow's milk, egg, house dust mites, and grass pollen.
Analysis of samples will be conducted by Q2 Solutions Laboratory. The baseline results will be provided and made available to the site staff and Sponsor and representatives. The results during the initial blinded period of 6 months after baseline will be kept blinded for each subject until the Month 6 database will be locked and 6-month study report will be issued. After the communication of the 6 initial month assigned treatment to the investigators and the subjects, the subject's specific immunological marker results can be provided unblinded. Further details of the procedures to be followed for sample collection, storage, and shipment will be documented in the Laboratory Manual.

6.3.2. Skin Prick Test

Peanut extract plus negative saline control and positive histamine control will be used for skin prick testing. All materials (SoluPrick® solutions, Duotip II® picking device and ancillary materials, CE marked, ALK, Denmark) will be centrally provided, free of charge to all sites by the Sponsor, along with a detailed procedure for performing the SPTs.
The subjects should wash out any antihistamines for the minimum period of time (specified in the APPENDIX 4) prior to performing the test. Briefly, a skin Duotip II® is pressed through a small drop of the commercial extract of peanut or positive and negative controls into the epidermis of the volar surface or back of the forearm.

After 15 minutes the area will be measured by the longest wheal diameter (D1) and the longest perpendicular diameter (d2). The Average wheal diameter = (D1 + d2) / 2.
e.g.

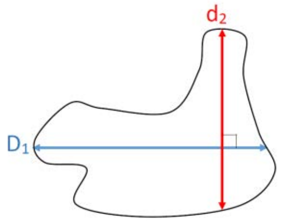

D1: size of the longest diameter in mm
d2: size of the longest perpendicular diameter to D1 in mm

A tracing should be obtained by using a fine ballpoint pen. The tracing will be performed at the demarcation line for the wheal as the skin drops back to flush. Scotch or a clear transparent tape should be used to lift the tracing; the tape tracing should be placed on the appropriate SPT Form and filed as part of the subject's source documentation.

.

6.3.3. *Food Allergy Quality of Life Questionnaire /Food Allergy Independent Measure*

The FAQLQs are disease-specific health-related quality of life questionnaires for subjects with food allergy. They are considered reliable and valid instruments to measure the impact of food allergy on health-related quality of life [38]. The FAIM questionnaires capture the subjects' expectation of something happening because of her/his food allergy [39].

At screening and at Month 6, 12, 18, 24, 30, 36 for all subjects and Month 42 for subjects initially in the placebo group, subjects ≥8 years of age and all parents/guardians will complete the FAQLQ/FAIM questionnaires. Children ≥8 years of age will complete the Child Forms of the questionnaires (FAQLQ-CF, FAIM-CF) while the parents/guardians, regardless of the age of their child, will complete the Parent Forms (FAQLQ-PF, FAIM-PF). The template questionnaires are provided in APPENDIX 7.

6.3.4. *Accidental Consumption of Peanut-containing Food*

Specific reactions triggered by accidental consumption of peanut-containing food and the conditions around that consumption will be collected with as much detailed information as possible, in particular with regard to the nature of the food and the quantity consumed. The subjects' parents/guardians will be asked to clarify whether any peanut consumption was accidental or not. The AEs induced by the consumption will be classified and analyzed separately.

As far as possible, a sample of the food having triggered the reactions will be collected and sent for analysis of its peanut content to a specialized laboratory (University of Nebraska, University of Nebraska-Lincoln; Dept of Food Science & Technology, FARRP, Nebraska USA). A specific procedure to organize the collection and the shipment of these samples will be provided to the centers and the subjects' parents/guardians.

6.3.5. Epigenetic analyses

Venous blood samples will be drawn to assess the epigenetic modifications induced by the EPIT treatment with Viaskin® Peanut on the promoter regions of the genes coding for several specific proteins. The tests will be managed centrally. Detailed procedures to be followed for sample collection, storage and shipment will be documented in a specific Manual provided to the sites.

6.3.6. Filaggrin Optional Genetic Testing

Samples of blood will be drawn from volunteer subjects for genetic analysis of the possible mutations in the filaggrin gene. The tests will be managed centrally. Detailed procedures to be followed for sample collection, storage and shipment will be documented in a specific Manual provided to the sites.

6.3.7. Scoring of Atopic Dermatitis

The SCORAD, a scoring index of atopic dermatitis developed by the European Task Force on Atopic Dermatitis [40] will be assessed be at screening, months 3, 6, 9, 12, 18, 24, 30, 36 for all subjects and 42 months for subjects initially in the placebo group, as specified in the Schedule of Procedures (Table 9).

Sections A and B must be assessed and completed by either the investigator, a physician or any specifically well-trained staff member, while section C is assessed by the subject/subject's parents. The SCORAD is provided in APPENDIX 8.

6.3.8. Peanut Food Challenge

Considering the length of this study, and for ensuring the subject's medical management, the investigators may need to (a) perform peanut-food challenge(s) during the course of the study, based on their own medical judgment.

A maximum of 2 open peanut-food challenges for the same subject is recommended during the subject treatment course up to the 36 months of active treatment. Performing open food challenge(s) in the REALISE study is optional and is left to the investigator's decision. However, no food challenge can occur before the subject has received 12 months of active treatment with Viaskin® Peanut 250 µg. If 2 peanut challenges are performed for the same subject within the treatment course, the second challenge can occur only after at least 12 additional months of active treatment have been administered to that subject.

If the decision is made by the investigators to perform a peanut-food challenge, its performance must comply with the standardized methodology defined in the protocol APPENDIX 6. A standardized peanut food challenge formula and material as well as the Manual of Procedures will be provided to all sites, and must be used for conducting the open peanut food challenge.

All food challenges results will be reported in the e-CRF. The following data should be reported when available:

- Peanut Eliciting Dose (ED),
- Peanut Cumulative Reactive Dose (CRD),
- Type and severity of the allergic symptoms elicited.

7. STUDY CONDUCT

7.1. Schedule of Procedures

The Schedule of Procedures is presented in Table 9.

Table 9: Schedule of Procedures

ALL SUBJECTS (ACTIVE AND PLACEBO ARMS) – FIRST 6 MONTHS BLINDED PERIOD									
**Study Assessments**	**Screening**	**Double-Blind Treatment Period (from V1 to V6)**							
**Visit tags – PC (Phone Call)**	**V1**	**V2**	**PC**	**V3**	**PC**	**V4**	**PC**	**V5**	**PC**
**Duration in study**	**Screen**	**D1**	**D4**	**D8**	**D22**	**M1**	**M2**	**M3**	**M4.5**
**Time Windows**		within 2 w. of V1	±2 d	±3 d	±2 d	±3 d	±3 d	±7 d	±7 d
Informed consent	X								
Medical history/Peanut allergy or positive Peanut FC documentation[1] / Family atopy history	X								
Check eligibility (inclusion/exclusion criteria)	X	X							
Demographics	X								
Physical examination [2]	X	X		X		X		X	
Vital signs [3]	X	X		X		X		X	
Spirometry (FEV_1) [4]	X								
PEF [5]	X	X		X		X		X	
SCORAD	X							X	
FAQLQ/FAIM [6]	X								
SPT (Skin Prick Test)	X							X	
Immunological markers [7]	X							X	
Laboratory tests [8]	X							X	
Filaggrin gene [9] *(Optional)*								X[7]	
Urine pregnancy test	X								
Epigenetic analyses	X							X	
Treatment Initiation		X							
Adverse events		X	X	X	X	X	X	X	X
Concomitant medications	X	X	X	X	X	X	X	X	X
Check for any accidental peanut consumption			X	X	X	X	X	X	X
Subject diary (dispense/check)		X	X	X	X	X	X	X	X
Dispense subject safety leaflet and subject identification card		X							
Apply Viaskin® patch at site		X							

ALL SUBJECTS (ACTIVE AND PLACEBO ARMS) – FIRST 6 MONTHS BLINDED PERIOD									
**Study Assessments**	**Screening**	**Double-Blind Treatment Period (from V1 to V6)**							
**Visit tags – PC (Phone Call)**	**V1**	**V2**	**PC**	**V3**	**PC**	**V4**	**PC**	**V5**	**PC**
**Duration in study**	**Screen**	**D1**	**D4**	**D8**	**D22**	**M1**	**M2**	**M3**	**M4.5**
**Time Windows**		within 2 w. of V1	±2 d	±3 d	±2 d	±3 d	±3 d	±7 d	±7 d
Time under observation before discharge		3 hrs							
Check skin reactions under the patch and grading [10]		X		X		X		X	
Dispense epinephrine auto-injector and anaphylaxis emergency action plan / 1% hydrocortisone ointment		X							
Review Epinephrine auto-injector use and subject safety precaution information leaflet, including anaphylaxis emergency action plan				X		X		X	
Assessment of used/unused study drug dispensed to the subject and of medication compliance				X		X		X	
Dispense study drug to the subject (Viaskin® Peanut 250 µg or Viaskin® placebo)		X		X		X		X[11]	

Abbreviations: D = Day; d = days; ET = Early termination; FAQLQ/FAIM = Food Allergy Quality of Life Questionnaire/Food Allergy Independent Measure; FEV_1 = Forced expiratory volume in one second; hrs = hours; M = Month; PC = Phone contact; PEF = Peak expiratory flow; SCORAD = Scoring atopic dermatitis; SPT = Skin prick test; UV = Unscheduled Visit; V = Visit;

1. Including history of peanut allergy and peanut FC previously performed, including date, procedures, doses used, challenge material and content in peanut protein, Eliciting Dose and symptoms.
2. Including a systematic complete skin examination, other physical examination as required,
3. Blood pressure, heart rate and respiratory rate.
4. FEV1 will be measured for subjects ≥6 years of age (unless they have documented inability to adequately perform spirometry).
5. PEF will be measured for all the subjects ≥5 years of age.
6. For both FAQLQ and FAIM, subjects ≥8 years of age will use the Child Form of the FAQLQ/FAIM. All parents/guardians will use the Parental Form. The FAQLQ and FAIM forms will be completed.
7. Peanut-specific IgE, peanut-specific IgG4, peanut-specific-component IgE and peanut-specific-component IgG4 to Ara h 1, Ara h 2, Ara h 3, Ara h 8 and Ara h 9. IgE specific to cow's milk, to egg white, to house dust mites, and to grass pollen will be tested at screening, month 3, 6, 9, 12, 18, 24, 30, 36 for all subjects and Month 42 for subjects initially in the placebo group.
8. Laboratory tests performed centrally. Hematology: hemoglobin, hematocrit, platelets, red blood cells, white blood cells with differential cell count. Biochemistry: aspartate aminotransferase, alanine aminotransferase, total bilirubin, blood urea nitrogen, creatinine, total protein.
9. Signing the consent for the filaggrin genetic analysis can be done any time after the subject is included in the study. However, collection of blood is done only once at Visit 5 or 6.
10. Check the reactions of the skin on the back of the subject and grade the severity of the local skin reactions. At Visit 2, grading is to be done before patch application and at 30 min, 1 h, 2 h and 3 h after patch application.

11. At Visit 5, the study drug (Viaskin® Peanut 250µg or Viaskin® placebo) is dispensed for a duration of 3 months.

	**ALL SUBJECTS – 6 to 42 MONTHS OPEN LABEL TREATMENT PERIOD**																		
**Study Assessments**	**Open Label Active Treatment Period**															**End of Study**	**Early Term**	**Unsched Visit**[12]
**Visit tags**	**V6**	**PC**	**V7**	**PC**	**V8**	**V9**	**V10**	**V11**	**PC**	**V12**	**PC**	**V13**	**PC**	**V14**	**PC**	**V15P**	**EoSV**	**ET**	**UV**
**Duration in study**	**M6**	**M6.5**	**M7**	**M8**	**M9**	**M12**	**M15**	**M18**	**M21**	**M24**	**M27**	**M30**	**M33**	**M36**	**M39**	**M42**			
**Time Windows**	**±7 d**	**±2 d**	**±3 d**	**±3 d**	**±7 d**	**±7 d**	**±7 d**	**±14 d**	**±14 d**	**±14 d**	**±14 d**	**±14 d**	**±14 d**	**±14 d**	**±14 d**	**±14 d**	**≤ 2 w after V14 or V15P**		
**Active Arm Active treatment duration**	M6	M6.5	M7	M8	M9	M12	M15	M18	M21	M24	M27	M30	M33	M36	-	-			
**Placebo Arm Active treatment duration**	D1	D15	M1	M2	M3	M6	M9	M12	M15	M18	M21	M24	M27	M30	M33	M36			
Physical examination [1]	X		X		X	X	X	X		X		X		X		XP	X	X	X
Vital signs [2]	X		X		X	X	X	X		X		X		X		XP	X	X	X
Spirometry (FEV$_1$) [3]	X					X		X		X		X		X		XP		X	X
PEF [4]	X		X		X	X		X		X		X		X		XP		X	X
SCORAD	X				X	X		X		X		X		X		XP		X	
FAQLQ/FAIM [5]	X					X		X		X		X		X		XP		X	
SPT (Skin Prick Test)	X				X	X		X		X		X		X		XP		X	
Immunological markers [6]	X				X	X		X		X		X		X		XP		X	
Laboratory tests [7]	X				X	X		X		X		X		X		XP		X	X
Filaggrin gene [8] *(Optional)*	X																		
Urine pregnancy test *(optional at other visits)*	X					X				X				X		XP			
Epigenetic analyses	X				X	X		X		X		X		X		XP			
Adverse events	X	X	X	X	X	X	X	X	X	X	X	X	X	X	XP	XP	X	X	X
Concomitant medications	X	X	X	X	X	X	X	X	X	X	X	X	X	X	XP	XP	X	X	X
Check for any accidental peanut consumption	X	X	X	X	X	X	X	X	X	X	X	X	X	X	XP	XP	X	X	X
Subject diary (dispense/check)	X	X	X	X	X	X	X	X	X	X	X	X	X	X	XP	XP	X	X	X
Apply Viaskin® patch at site	X																		

	ALL SUBJECTS – 6 to 42 MONTHS OPEN LABEL TREATMENT PERIOD																		
**Study Assessments**	**Open Label Active Treatment Period**															**End of Study**	**Early Term**	**Unsched Visit[12]**
**Visit tags**	**V6**	**PC**	**V7**	**PC**	**V8**	**V9**	**V10**	**V11**	**PC**	**V12**	**PC**	**V13**	**PC**	**V14**	**PC**	**V15P**	**EoSV**	**ET**	**UV**
**Duration in study**	**M6**	**M6.5**	**M7**	**M8**	**M9**	**M12**	**M15**	**M18**	**M21**	**M24**	**M27**	**M30**	**M33**	**M36**	**M39**	**M42**			
**Time Windows**	**±7 d**	**±2 d**	**±3 d**	**±3 d**	**±7 d**	**±7 d**	**±7 d**	**±14 d**	**±14 d**	**±14 d**	**±14 d**	**±14 d**	**±14 d**	**±14 d**	**±14 d**	**±14 d**	**≤ 2 w after V14 or V15P**		
**Active Arm Active treatment duration**	M6	M6.5	M7	M8	M9	M12	M15	M18	M21	M24	M27	M30	M33	M36	-	-			
**Placebo Arm Active treatment duration**	D1	D15	M1	M2	M3	M6	M9	M12	M15	M18	M21	M24	M27	M30	M33	M36			
Time under observation before discharge	3 hrs																		
Check skin reactions under the patch and grading [9]	X		X		X	X	X	X		X		X		X		XP		X	X
Review Epinephrine auto-injector use and subject safety precaution information leaflet, including anaphylaxis emergency action plan	X		X		X	X	X	X		X		X		X		XP		X	X
Assessment used/unused study drug dispensed to the subject	X		X		X	X	X	X		X		X		X		XP		X	X
Peanut Food Challenge (optional) [10]						X[11]		X		X		X		X		XP			
Dispense study drug to the subject (Viaskin® Peanut 250 µg)	X		X		X	X	X	X		X		X		XP					

Abbreviations: D = Day; d = days; ET = Early termination; FAQLQ/FAIM = Food Allergy Quality of Life Questionnaire/Food Allergy Independent Measure; FEV_1 = Forced expiratory volume in one second; hrs = hours; M = Month; PC = Phone contact; PEF = Peak expiratory flow; SCORAD = Scoring atopic dermatitis; SPT = Skin prick test; UV = Unscheduled Visit; V = Visit; XP = Procedure or test only conducted with the subjects initially randomized in the Placebo arm for the first 6 months,

1. Including a systematic complete skin examination, other physical examination as required,
2. Blood pressure, heart rate and respiratory rate.

3. FEV1 will be measured for subjects ≥6 years of age (unless they have documented inability to adequately perform spirometry).
4. PEF will be measured for all the subjects ≥5 years of age.
5. For both FAQLQ and FAIM, subjects ≥8 years of age will use the Child Form of the FAQLQ/FAIM. All parents/guardians will use the Parental Form. The FAQLQ and FAIM forms will be completed.
6. Peanut-specific IgE, peanut-specific IgG4, peanut-specific-component IgE and peanut-specific-component IgG4 to Ara h 1, Ara h 2, Ara h 3, Ara h 8 and Ara h 9. IgE specific to cow's milk, to egg white, to house dust mites, and to grass pollen will be tested at screening, month 3, 6, 9, 12, 18, 24, 30, 36 for all subjects and Month 42 for subjects initially in the placebo group.
7. Laboratory tests performed centrally. Hematology: hemoglobin, hematocrit, platelets, red blood cells, white blood cells with differential cell count. Biochemistry: aspartate aminotransferase, alanine aminotransferase, total bilirubin, blood urea nitrogen, creatinine, total protein.
8. Signing the consent for the filaggrin genetic analysis can be done any time after the subject is included in the study. However, collection of blood is done only once at Visit 5 or 6.
9. Check the reactions of the skin on the back of the subject and grade the severity of the local skin reactions. At Visit 6, grading is to be done before patch application and 30 min, 1 h, 2 h and 3 h after patch application.
10. A maximum of 2 open peanut food challenges can be performed during the course of the study. The first food challenge should be performed after a minimum of 12 months of active treatment. The 2nd peanut challenges can be performed for the same subject within the treatment course only after at least 12 additional months of active treatment have been administered to that subject.
11. Peanut challenge might be conducted at this visit only for the subjects initially randomized in the active arm.
12. Procedures during the unscheduled visits will be performed as deemed necessary by the investigator.

7.2. Procedures by Visit

Visits should occur within the time windows indicated in the Schedule of Procedures Table 9. All times should be recorded using the 24-hour clock (for example 23:20, not 11:20 pm).

7.2.1. Visit 1, Screening

The first screening Visit (Visit 1) will take place within 2 weeks prior to the start of the treatment period. Therefore, the duration of the screening period would be less than or equal to 14 days.

The following assessments will be performed at this visit:

- Signed informed consent;
- FAQLQ/FAIM (see Section 6.3.3);
- Family (father and/or mother/sibling) medical history of atopy (any allergies, asthma conditions, eczema/atopic dermatitis);
- Check inclusion/exclusion criteria, except for those criteria that depend on the results of the peanut-specific IgE assessment;
- Disease History/Medical history (see Section 6.1.2);
- Demographics (see Section 6.1.1);
- Physical examination (including a complete skin examination; (see Section 6.2.6);
- Vital signs (see Section 6.2.5);
- Spirometry (FEV_1) (see Section 6.2.7);
- PEF (see Section 6.2.8);
- SCORAD (see Section 6.3.7);
- SPT (see Section 6.3.2);
- Immunological markers (see Section 6.3.1);
- Laboratory tests (see Section 6.2.4); if applicable, subjects with abnormal laboratory assessments due to a concomitant transient disease (flu, viral illness, etc.) can repeat their laboratory assessments or be rescheduled for laboratory assessment at the discretion of the Investigator);
- Urine pregnancy test for female subjects of childbearing potential;
- Collect blood sample for epigenetic analyses (see Section 6.3.5);
- Concomitant medications (see Sections 5.7 and 6.1.3);
- Schedule Visit 2.

7.2.2. Visit 2 (Day 1), First Day of Treatment – Double-Blind Period

All screening assessments must be completed and subject eligibility must be checked before starting the treatment period. Visit 2 should take place at a maximum of two weeks after Visit 1. This visit can take place as soon as the peanut-specific IgE and the blood testing results for the subject are obtained by the site from the central laboratory. If the subject fulfills all inclusion criteria including the peanut-specific IgE value (≥14 kU/L), then Visit 2 may proceed.

The following assessments will be performed at this visit:

- Physical examination (including a complete skin examination (see Section 6.2.6);
- Vital signs (see Section 6.2.5);
- PEF (see Section 6.2.8);
- Confirm eligibility of subject;
- AEs recording (including volunteered or solicited AEs and AESI);
- Concomitant medications (see Sections 5.7 and 6.1.3);
- Dispense first box of study drug (instruct subject/parents/guardians on the proper application, timing and storage conditions);
- Apply the first Viaskin® patch to the subject and stick the tear-off part of the pouch label in the appropriate section of the subject diary.
- Keep under observation on-site for 3 hours after the patch has been applied;
- Check skin reaction under patch and grade severity. Take photos of the application site if required (see Section 6.2.10);
- Dispense subject diary (instruct subject/parents/guardians on the use); see Section 6.2.9);
- Dispense the subject safety precaution information leaflet and subject identification card;
- Dispense an auto-injector of epinephrine and explain in detail the anaphylaxis emergency action plan to the subject/parents/guardians before discharge;
- Dispense 1% hydrocortisone ointment;
- Schedule the Day 4 phone contact and Visit 3.

After the first application of the Viaskin® patch at the study site, all subjects will be observed for 3 hours before being discharged with the patch still applied to the skin and the reactions under or around the patch will be checked and graded before discharge. After a total of 6 hours of application, which is approximately 3 hours after discharge, the subject/parents/guardians will be instructed to remove the patch, and clean the application site.

7.2.3. Phone Contacts (Day 4, Day 22, Month 2, Month 4.5) – Double-Blind Period

The parents/guardians of the subject will be called between two consecutive visits, to assess the subject's condition since the previous Visit.

At Day 4 after the patch was applied during 6 hours daily for 3 days, and at Day 22 (Week 3 of treatment), 1 week after the patch was applied for 24 hours daily, specific phone contacts will be made to the parents/guardians to assess the safety/tolerability of patch applications on the back.

At Months 2 and 4.5 the phone contacts will be scheduled to ensure that the subject and his/her parents do not have any issue or specific questions, and that the diary is correctly filled up.

During the phone contacts, the site staff may ask the parents to take photos of the back of the subject to assess the local skin reactions. If required, the next visit may be scheduled earlier by the Investigator.

The following assessments will be performed during the phone contacts:

- AEs recording (including volunteered or solicited AEs and AESI);
- Concomitant medications (see Section 5.7 and 6.1.3)
- Check for any accidental peanut consumption (see Section 6.3.4);
- Check with the subject (parents/guardians) to ensure that the diary has been completed accurately and that the subject/parents/guardians is/are comfortable with using the diary;
- Remind them the next visit and to bring back their diary at next visit on site.

7.2.4. *Visit 3 (Day 8) and Visit 4 (Month 1) – Double-Blind Period*

The following simplified set of assessments will be performed at these visits:

- Physical examination (including a complete skin examination (see Section 6.2.6);
- Vital signs (see Section 6.2.5);
- PEF (see Section 6.2.8);
- AEs recording (including volunteered or solicited AEs and AESI);
- Concomitant medications (see Sections 5.7 and 6.1.3);
- Check for any accidental peanut consumption (see Section 6.3.4);
- Check that the subject diary has been completed accurately and ensure that the subject/parents/guardians is/are comfortable with using the diary; (re-instruct subject/parents/guardians on the use, if necessary; see Section 6.2.9);
- Give the diary back to the subject;
- Collect the treatment box dispensed at the previous visit, check the unused medication and assess medication compliance;
- Dispense a new box of study drug (re-instruct subject/parents/guardians on the proper application, timing and storage conditions, if necessary);
- Check skin reactions under or around the current patch (do not remove patch) and all other zones of application and grade severity of the skin reactions. Take photos of the application site if required (see Section 6.2.10);
- Review the use of the auto-injector of epinephrine and the anaphylaxis emergency action plan;
- Schedule next phone contacts and Visits.

7.2.5. *Visit 5 (Month 3) – Double-Blind Period*

The following assessments will be performed:

- Physical examination (including a complete skin examination (see Section 6.2.6);
- Vital signs (see Section 6.2.5);
- PEF (see Section 6.2.8). The test may be repeated more frequently for asthmatic subjects if necessary.
- SCORAD;
- SPT (see Section 6.3.2);
- Immunological markers (see Section 6.3.1);

- Laboratory tests (see Section 6.2.4);
- Written informed consent for filaggrin genetic testing (may be obtained at any visit after screening, but must occur before blood sampling)
- Blood sampling for filaggrin genetic testing (after written informed consent has been obtained; only 1 sample is to be collected at Visits 5 or 6; see Section 6.3.6);
- Collect blood samples for epigenetic analyses (see Section 6.3.6);
- AEs recording (including volunteered or solicited AEs and AESI);
- Concomitant medications (see Sections 5.7 and 6.1.3);
- Check for any accidental or peanut consumption (see Section 6.3.4);
- Check that the subject diary has been completed accurately and ensure that the subject/parents/guardians is/are comfortable with using the diary (re-instruct subject/parents/guardians on the use, if necessary; see Section 6.2.9);
- Give the diary back to the subject;
- Check skin reactions under or around the current patch (do not remove patch) and all other zones of application and grade severity of the skin reactions. Take photos of the application site if required (see Section 6.2.10);
- Review the use of the auto-injector of epinephrine and the anaphylaxis emergency action plan;
- Collect the treatment box dispensed at the previous visit, check the unused medication and assess medication compliance;
- Dispense a new box of study drug (re-instruct subject/parents/guardians on the proper application, timing and storage conditions, if necessary);
- Schedule next visit;

7.2.6. Visit 6 – Open Label Viaskin® Peanut 250 µg for All Subjects

This visit includes the complete set of assessments. The following assessments will be performed:

- Physical examination (including a complete skin examination (see Section 6.2.6);
- Vital signs (see Section 6.2.5);
- Spirometry (FEV_1, see Section 6.2.7) and/or PEF (see Section 6.2.8). These tests may be repeated more frequently for asthmatic subjects if necessary.
- SCORAD;
- FAQLQ/FAIM (see 6.3.3)
- SPT (see Section 6.3.2);
- Immunological markers (see Section 6.3.1);
- Laboratory tests (see Section 6.2.4);
- Blood sampling for filaggrin genetic testing (after written informed consent has been obtained; only 1 sample is to be collected at Visits 5 or 6; see Section 6.3.6);
- Written informed consent for filaggrin genetic testing (may be obtained at any visit after screening, but must occur before blood sampling);
- Pregnancy urine test,

- Collect blood samples for epigenetic analyses (see Section 6.3.6);
- AEs recording (including volunteered or solicited AEs and AESI);
- Concomitant medications (see Sections 5.7 and 6.1.3);
- Check for any accidental or peanut consumption (see Section 6.3.4);
- Check that the subject diary has been completed accurately and ensure that the subject/parents/guardians is/are comfortable with using the diary (re-instruct subject/parents/guardians on the use, if necessary; see Section 6.2.9);
- Collect the treatment box dispensed at the previous visit, check the unused medication and assess medication compliance;
- Check skin reactions under or around the current patch (do not remove patch) and all other zones of application and grade severity of the skin reactions. Take photos of the application site if required (see Section 6.2.10);
- Review the use of the auto-injector of epinephrine and the anaphylaxis emergency action plan;

At Visit 6, the IWRS will assign to all subjects the active Viaskin® Peanut 250 µg patches treatment in an open label manner and the following assessments and procedures will be performed:

- A 1-month box of peanut Viaskin® Peanut 250 µg patches will be allocated to the subjects. The subject/parents/guardians will be re-instruct on the proper application, timing and storage conditions.
- The active Viaskin® patch (first active patch application for the patients who were in the placebo group) will be applied to the subject at site, the tear-off part of the pouch label will be stuck in the appropriate section of the subject diary. Subject/parents/guardians must be re-instructed on the proper application, timing and storage of study drug, if necessary);
- The subject will be kept under observation on-site for 3 hours after the patch has been applied;
- Check skin reactions under patch and grade severity. Take photos of the application site if required (see Section 6.2.10);
- Dispense a new diary to the subject (instruct subject/parents/guardians on the use); see Section 6.2.9);

All subjects and their parents will be instructed to repeat the treatment initiation phase as it was conducted during the first 3 weeks of the study treatment. The treatment initiation with the open label Viaskin® Peanut 250µg will then be followed by all subjects and it consists of the progressive increase of the patches application duration, following the same protocol than at the study start:

  - 6-hours/day (± 1 hour) application for the 1st week,
  - 12-hours/day (± 2 hours) application for the 2nd week,
  - 24-hours/day (± 4 hours).application from the 3rd week and onward,

- The subject will be scheduled for the next Phone contact 15 days later and a visit 1 month later for ensuring the follow-up of the treatment period and check the safety of the active patch application.
- Schedule next phone contact and visit;

7.2.7. *Visit 7 (Month 7), Visit 10 (Month 15) - Open Label Period*

These visits will be conducted with all the subjects independently to the previously received treatment, the blind code of these treatment being not yet known.
The following assessments will be performed:

- Physical examination (including a complete skin examination, see Section 6.2.6);
- Vital signs (see Section 6.2.5);
- PEF (see Section 6.2.8); PEF will not be required at Visit 10.
- AEs recording (including volunteered or solicited AEs and AESI);
- Concomitant medications (see Sections 5.7 and 6.1.3);
- Check for any accidental peanut consumption (see Section 6.3.4);
- Check that the subject diary has been completed accurately and ensure that the subject/parents/guardians is/are comfortable with using the diary (re-instruct subject/parents/guardians on the use, if necessary; see Section 6.2.9);
- Give the diary back to the subject;
- Collect the treatment box dispensed at the previous visit, check the unused medication and assess medication compliance;
- Dispense a new box of study drug (re-instruct subject/parents/guardians on the proper application, timing and storage conditions, if necessary);
- Check skin reactions under or around the current patch (do not remove patch) and all other zones of application and grade severity of the skin reactions. Take photos of the application site if required (see Section 6.2.10);
- Review the use of the auto-injector of epinephrine and the anaphylaxis emergency action plan;
- Schedule next phone contact and next Visit.

7.2.8. *Phone Contacts (Month 6.5, Month 8, Month 21, Month 27, Month 33, Month 39 - subjects initially in placebo arm) – Open Label Period*

The parents/guardians of the subject will be called between two consecutive visits, except when the visits are performed within 3 months. These phone contacts could be repeated or be scheduled more frequently if a specific aspect of the treatment needs to be more closely monitored by the investigator's staff.
The phone contacts will be scheduled to ensure that the subject and his/her parents do not have any issues or specific questions, and that the diary is correctly filled out.
During the phone contacts, the site staff may ask the parents to take photos of the back of the subject to assess the local skin reactions. If required, the next visit may be scheduled earlier by the Investigator.

The following assessments will be performed during the phone contacts:

- AEs recording (including volunteered or solicited AEs and AESI);
- Concomitant medications (see Section 5.7 and 6.1.3)
- Check for any accidental peanut consumption (see Section 6.3.4);
- Check with the subject (parents/guardians) to ensure that the diary has been completed accurately and that the subject/parents/guardians is/are comfortable with using the diary;
- Remind them the next visit.

7.2.9. Visit 8 (Month 9), Visit 9 (Month 12), Visit 11 (Month 18), Visit 12 (Month 24), Visit 13 (Month 30), - Open Label Period

This visit includes the complete set of assessments. The following assessments will be performed:

- Physical examination (including a complete skin examination, see Section 6.2.6);
- Vital signs (see Section 6.2.5);
- Spirometry (FEV_1, see Section 6.2.7). Not required at Visit 8;
- PEF (see Section 6.2.8);
- SCORAD;
- FAQLQ/FAIM (see 6.3.3). Not required at Visit 8;
- SPT (see Section 6.3.2);
- Immunological markers (see Section 6.3.1);
- Laboratory tests (see Section 6.2.4);
- If required, perform the open peanut food challenge (at Visits Month 12, Month 18, Month 24 or Month 30).
- Pregnancy urine test. Not required at Visit 8, Visit 11 and Visit 13;
- Collect blood samples for epigenetic analyses (see Section 6.3.6);
- AEs recording (including volunteered or solicited AEs and AESI);
- Concomitant medications (see Sections 5.7 and 6.1.3);
- Check for any accidental or peanut consumption (see Section 6.3.4);
- Check that the subject diary has been completed accurately and ensure that the subject/parents/guardians is/are comfortable with using the diary (re-instruct subject/parents/guardians on the use, if necessary; see Section 6.2.9); at Visit 9, dispense a new diary to the subject (instruct subject/parents/guardians on the use); see Section 6.2.9);
- Collect the treatment box dispensed at the previous visit, check the unused medication and assess medication compliance;
- Check skin reactions under or around the current patch (do not remove patch) and all other zones of application and grade severity of the skin reactions. Take photos of the application site if required (see Section 6.2.10);
- Review the use of the auto-injector of epinephrine and the anaphylaxis emergency action plan;

- Dispense a new box of study drug (re-instruct subject/parents/guardians on the proper application, timing and storage conditions, if necessary);
- Schedule next phone contacts and next Visits.

7.2.10. Visit 14 (Month 36), Visit 15P (Month 42- only for subjects initially in placebo arm) - End of Open Label Period

The end of the 36-month treatment period will be reached at Visit 14 for initial active arm subjects and at Visit 15P for the initial placebo arm subjects.
For the subjects initially in the active group, the patch worn by the subject when she/he arrives to this visit is removed and there will be no further patches applied beyond this visit. Only the subjects initially in the placebo group will continue the study for further 6 months until the Visit 15P, at Month 42.
This visit includes the complete set of assessments. The following assessments will be performed:

- Physical examination (including a complete skin examination, see Section 6.2.6);
- Vital signs (see Section 6.2.5);
- Spirometry (FEV_1, see Section 6.2.7).
- PEF (see Section 6.2.8);
- SCORAD;
- FAQLQ/FAIM (see 6.3.3);
- SPT (see Section 6.3.2);
- Immunological markers (see Section 6.3.1);
- Laboratory tests (see Section 6.2.4);
- If required, perform the open peanut food challenge (at Visits Month 36, or Month 42).
- Pregnancy urine test;
- Collect blood samples for epigenetic analyses (see Section 6.3.6);
- AEs recording (including volunteered or solicited AEs and AESI);
- Concomitant medications (see Sections 5.7 and 6.1.3);
- Check for any accidental or peanut consumption (see Section 6.3.4);
- Check that the subject diary has been completed accurately and ensure that the subject/parents/guardians is/are comfortable with using the diary (re-instruct subject/parents/guardians on the use, if necessary; see Section 6.2.9);
- Collect the treatment box dispensed at the previous visit, check the unused medication and assess medication compliance;
- Check skin reactions under or around the current patch (do not remove patch) and all other zones of application and grade severity of the skin reactions. Take photos of the application site if required (see Section 6.2.10);
- Review the use of the auto-injector of epinephrine and the anaphylaxis emergency action plan;

- Dispense a new box of study drug for subjects initially randomized in the Placebo arm continuing the study up to Months 42 V15P. (re-instruct subject/parents/ guardians on the proper application, timing and storage conditions, if necessary). Not applicable for Visit 15P.

The final visit will be the EoS study conducted within the 2 following weeks.

7.2.11. End of Study Visit (EoS)

This visit will be conducted 2 weeks after the previous visit, either Visit 14 or Visit 15P, after the 36-month Viaskin® Peanut 250µg treatment is completed.
The following assessments will be performed at this visit:

- Physical examination (including a complete skin examination (see Section 6.2.6);
- Vital signs (see Section 6.2.5);
- AEs recording (including volunteered or solicited AEs and AESI);
- Concomitant medications (see 5.7 and 6.1.3);
- Check for any accidental or peanut consumption (see Section 6.3.4).
- Check subject diary;

7.2.12. Early Termination Visit

Subjects who discontinue early from the study should undergo an Early Termination Visit. This visit should take place as soon as possible after the subject stops taking the study drug (see also Section 4.4.4).

- Physical examination (including a complete skin examination (see Section 6.2.6);
- Vital signs (see Section 6.2.5);
- Spirometry (FEV_1) (see Section 6.2.7);
- PEF (see Section 6.2.8);
- SCORAD;
- FAQLQ/FAIM (see 6.3.3)
- SPT (see Section 6.3.2);
- Immunological markers (see Section 6.3.1);
- Laboratory tests (see Section 6.2.4);
- Collect blood samples for epigenetic analyses (see Section 6.3.6);
- AEs recording (including volunteered or solicited AEs and AESI);
- Concomitant medications (see Sections 5.7 and 6.1.3);
- Check for any accidental peanut consumption (see Section 6.3.4);
- Check that the subject diary has been completed accurately
- Check skin reactions under or around the patch (do not remove patch) and all other zones of application and grade severity of the skin reactions. Take photos of the application site if required (see Section 6.2.10).
- Review the use of the auto-injector of epinephrine,
- Check unused medication and assess medication compliance.

7.2.13. Unscheduled Visit

Subjects may come to the site for visits outside of the scheduled visits in case of AEs, or any other issue requiring the investigator's assistance, etc.

The following assessments will be performed at this visit, at the discretion of the Investigator:

- Physical examination (including a complete skin examination (see Section 6.2.6);
- Vital signs (see Section 6.2.5);
- Spirometry (FEV_1) (see Section 6.2.7);
- PEF (see Section 6.2.8);
- Laboratory tests (see Section 6.2.4);
- AEs recording (including volunteered or solicited AEs and AESI);
- Concomitant medications (see Sections 5.7 and 6.1.3);
- Check for any accidental or peanut consumption (see Section 6.3.4);
- Check subject diary;
- Check unused medication and assess medication compliance;
- Check skin reactions under or around the current patch (do not remove patch) and all other zones of application and grade severity of the skin reactions. Take photos of the application site if required (see Section 6.2.10).
- Review the use of the auto-injector of epinephrine and the anaphylaxis emergency action plan.

At the end of the visit, the investigator will decide of the next steps of the study, either continue the scheduled course of the study or to adapt is to the issue encountered (additional unscheduled visits, phone contacts…).

8. STATISTICAL METHODS

The statistical considerations summarized in this section outline the plan for data analysis of this study.

A separate detailed statistical analysis plan (SAP) providing detailed methods for the analyses outlined below will be prepared and finalized before the database lock.

Any deviations from the planned analyses will be described and justified in the final integrated study report.

8.1. Study Subjects

8.1.1. Disposition of Subjects

Subject disposition will be summarized for Safety population overall. The number and percentage of subjects screened, subjects in each study population (Randomized set, Safety population, Per-protocol [PP] population), and subjects who completed the treatment period, who discontinued the treatment period, and the primary reason for discontinuation from the treatment period will be tabulated. Number and percentage of peanut allergic subjects presenting a medical history of severe anaphylaxis to peanut in the Safety population will also be tabulated.

An enrollment summary will be presented overall and by site, showing the first date of consent, and the last study visit exit date among enrolled subjects, duration (in days) which is calculated as last study visit exit date – first date of consent +1, number of subjects enrolled, treated and completed. The number and percentage of subjects enrolled in total and by site will be summarized.

8.1.2. Protocol Deviations

Deviations from the protocol including violations of inclusion/exclusion criteria will be assessed as "minor" or "major" in cooperation with the Sponsor. Major deviations from the protocol that impact the safety evaluation will lead to the exclusion of a subject from the PP population.

8.1.3. Analysis Sets

All analysis populations will be identified and finalized in the SAP.

8.1.4. Randomized Population

The Randomized population will be comprised of all subjects who are randomized in the study.
This population will be used to describe subject disposition and baseline characteristics.

8.1.5. Safety Population

The Safety population will be comprised of all subjects from the Randomized set that have received at least 1 dose of study drug. In case the wrong study drug is dispensed, the subject will be analyzed according to the study drug actually received for the longest period of time. Peanut allergic subjects presenting a medical history of severe anaphylaxis after peanut consumption will be identified among the Safety population using the sub-populations described hereafter.

8.1.6. Safety Sub-Population – Subjects without a history of severe anaphylaxis to peanut

This sub-population will include all subjects from the Safety set who do not present a medical history of severe anaphylaxis to peanut (Food-induced anaphylaxis reaction of grade 3, ref. APPENDIX 5). This sub-population will first be used to describe the safety endpoints.

8.1.7. Safety Sub-Population – Subjects with a history of severe anaphylaxis to peanut

This sub-population will include all subjects from the Safety set presenting a medical history of severe anaphylaxis to peanut after either consumption foods containing peanut or after a peanut-food challenge. Severe anaphylaxis reactions are defined as specified in APPENDIX 5.
The safety of the Safety sub-population with a history of severe anaphylaxis to peanut will be studied separately using tables summarizing the main safety endpoints (TEAEs, skin reactions…). If less than 15 subjects are included in this sub-population, listings will be

edited rather than tables. This Safety sub-population will be compared to the Safety sub-population without a history of severe anaphylaxis to peanut.
If no major safety differences are observed as evaluated by the DSMB, both sub-populations will be pooled and safety analyses will be repeated on the overall Safety population. If the safety profile of the two sub-populations is not comparable, these two sub-populations will be analyzed separately.

8.1.8. Per-protocol Population

The PP population will include all subjects from the Safety population who do not have major deviations from the protocol that may affect the safety evaluation. The deviations to consider will be listed more exhaustively in the SAP. The PP population will be used to perform confirmatory analyses of the safety evaluation.
In case the 2 Safety sub-populations are not equivalent, PP sub-populations will be defined:

- PP sub-population without a history of severe anaphylaxis to peanut;
- PP sub-population with a history of severe anaphylaxis to peanut.

8.2. General Considerations

8.2.1. Statistical Methods

The statistical analyses for the entire study as further outlined in the SAP will be included in the clinical study report (CSR) for this protocol. The SAP will give a detailed description of the summaries and analyses that will be performed and will clearly describe when these analyses will take place. The SAP will be prepared shortly after the final protocol and well ahead of the database lock to preserve the integrity of the statistical analysis and study conclusions.
Statistical analyses will be performed using SAS® Version 9.3 or higher (SAS Institute, Cary, NC, USA).
Categorical variables will be summarized using number of observations and percentages. The denominator for percentages will be the number of subjects in the population with data available unless otherwise stated. Continuous variables will be summarized using descriptive statistics (number of observations [n], mean, standard deviation, minimum, first quartile [Q1], median, third quartile [Q3], and maximum).

8.2.2. Analysis and Data Conventions

The subjects randomized in the placebo arm who will cross-over to the active Viaskin® treatment after the 6-month double-blind period, will have their assessments rescheduled and merged to those of the active arm subjects, according to the duration of the active treatment received. The baseline will be re-defined in order to compare the first day under active treatment with the follow-ups beyond Month 6 for all subjects. The visits will be re-labelled for the subjects initially randomized in the placebo group as follows:

Time point:	D1	M1	M3	M6	M9	M12	M18	M24	M30	M36
Initial group: active	V2	V4	V5	V6	V8	V9	V11	V12	V13	V14
Initial group: placebo	V6	V7	V8	V9	V10	V11	V12	V13	V14	V15P

Safety endpoints prior to Month 6 will be analyzed by treatment group and tables will show "Viaskin® 250 µg" group *versus* "Placebo" group. Safety endpoints post Month 6 will be analyzed using the above rescheduling rules and tables will show the "Viaskin® 250 µg" group, the "Rescheduled Placebo" group, and both groups pooled together.

8.2.3. Definition of Baseline

For the first 6 months double-blind treatment period, the baseline assessment will be the last, valid pre-dose assessment available.
For the analyses using the rescheduling rules (see Section 8.2.2), the baseline assessment will be the last, valid pre-active dose assessment available.

8.2.4. Visit Windows

Assessments outside of protocol allowable windows will be taken into account in the analysis according to the visit in which the data are entered.

8.2.5. Unscheduled Assessments

Additional assessments (laboratory data or vital signs associated with non-protocol clinical visits or obtained in the course of investigating or managing AEs) will be included in listings, but not summaries (except for food challenge data). If more than 1 laboratory value is available for a given visit, the first valid observation will be used in summaries and all observations will be presented in listings. It is noted that invalid laboratory data may not be used (from hemolyzed samples, mishandled samples, quantity not sufficient, or other conditions that would render values invalid).

8.2.6. Missing Data Conventions

Best efforts should be made by the Investigator to provide complete data.
Analyses of safety endpoints:
Partial or missing safety data will be imputed according to the most conservative approach. Adverse events with missing or incomplete onset date will be considered as TEAEs unless there is evidence that the event occurred prior to the treatment period. Treatment-emergent AEs with missing relationship to the study drug will be considered as drug-related and TEAEs with missing severity will be considered as severe. Actual values will be presented in listings.
Analyses of Exploratory Endpoints:
No imputation will be performed and observed data will be used.

8.3. Demographics, Disease and Medical History, Baseline Characteristics, and Concomitant Medications

Descriptive statistics will be produced for continuous demographic and baseline characteristics (including age, height, weight, BMI, FEV_1, PEF, and peanut-specific IgE) for the whole Safety population. The number and percent of subjects in each modality of the categorical demographic and baseline characteristics (including race/ethnicity and SPT) will be presented.

Prior and concomitant medications will be coded using the latest available version of the World Health Organization (WHO) Drug Dictionary. Summaries of prior and concomitant medications will be produced by preferred drug name. All concomitant medications will be listed.

Medical history will be reported by SOC and PT and coded using the latest available version of the Medical Dictionary for Regulatory Activities (MedDRA) dictionary. Disease history will be described, including

- age at time of diagnosis,
- time from diagnosis to study enrolment,
- physician category who made the diagnosis,
- main reason having led to the diagnosis (reaction after ingestion, parental/sibling history of atopy, other risk factors)
- diagnosis criteria (SPT, IgE, positive food challenge, peanut allergic reaction)
- Most recent results of peanut allergy diagnostic tests performed,
- Description of allergic reactions (number of reactions per subject, number of reaction in the previous year…)

All individual subject demographic and baseline characteristic data will be listed.

8.4. Treatment Compliance and Exposure

Treatment compliance (see Section 5.6) will be summarized for the whole Safety population by means of descriptive statistics (n, mean, SD, median, Q1, minimum, Q3, and maximum) and/or frequency tables (compliance < or ≥80%).

The total number of days of patch application during the whole study will be presented on the Safety population. Besides, the exposure duration by subject will be summarized, quantitatively and by category. Exposure duration will be calculated regardless of temporary interruptions, as follows:

Date of last patch application – Date of first patch application + 1

The total dose of peanut protein received during the study (mg) will also be summarized.

8.4.1. Safety Analyses

Safety endpoints will first be evaluated for the Safety Sub-population without a history of severe anaphylaxis to peanut, and described on the Safety Sub-population with a history of severe anaphylaxis to peanut. If no major safety differences are observed (as determined by

the DSMB), both sub-populations will be merged and safety endpoints will be evaluated on the overall Safety population.

Safety endpoints prior to Month 6 will be analyzed by treatment group and tables will display "Viaskin® 250 µg" group *versus* "Placebo" group. Safety endpoints post Month 6 will be analyzed using the rescheduling rules described in Section 8.2.2 and tables will show the "Viaskin® 250 µg" group, the "Rescheduled Placebo" group, and both groups pooled together.

8.4.2. Adverse Events

Treatment-emergent AEs will be defined as any AEs, regardless of relationship to study drug, which occur during or after the initial Viaskin® patch application or any event already present that worsens in either severity or relationship to study drug following exposure to Viaskin® patches.

All AEs will be reported by SOC and PT as detailed in Section 9.4 and coded using the latest available version of the MedDRA dictionary.

An overall summary of TEAEs will be provided showing the number percentage of subjects with any TEAEs, any potentially drug-related TEAEs, any severe TEAEs, any severe potentially drug-related TEAEs, any serious TEAEs, any serious potentially drug-related TEAEs, any TEAEs leading to discontinuation, and any TEAEs leading to death. The number of events will also be presented.

The number of TEAEs as well as the number and percentage of subjects who experienced at least one TEAE will be summarized by SOC and PT. The incidence of the following events will be summarized:

- TEAEs (distinguished from symptoms/reactions elicited during the FCs): incidence, maximum severity, duration and relatedness to treatment patch, placebo or active;
- TEAEs leading to discontinuation;
- Incidence, duration and maximum severity local skin tolerance at sites of Viaskin® patch application as assessed by the subjects (see Section 8.4.7);
- Severity of local skin tolerance at sites of Viaskin® patch application as assessed by the Investigator (see Section 8.4.8);
- Adverse Events of Special Interest (AESI) (see 6.2.1.8);
- Serious Adverse Events (SAEs) by SOC and PTs, and relatedness to Viaskin® patch;
- Laboratory data, physical examinations and vital signs;
- Spirometry results or PEF results.

The percentage of doses with any reaction, mild patch site reaction, moderate patch site reaction and severe patch site reactions, based on subject's treatment exposure, will be estimated and summarized.

The safety parameters above will be analyzed overall and for each of the age ranges 4 to 5 years and 6 to 8 years and 9-11 years.

All AEs will be listed.

The reactions appearing during a food challenge (as they are expressly provoked) will be differentiated from those AEs occurring outside of the food challenge.

8.4.3. Laboratory Assessments

Descriptive statistics will be calculated for all laboratory tests (hematology and biochemistry)

- at Baseline and Months 3, 6, 9, 12, 18, 24, 30, 36 for all subjects and 42 only for subjects initially randomized in the placebo group.

Categorical variables will be summarized by frequency and percentages of subjects in corresponding categories.

Changes in laboratory data from baseline will also be presented.

In addition, summaries of laboratory values categorized based on Common Toxicity Criteria for Adverse Events (CTCAE) grade will also be presented.

Shift tables of test abnormalities will be generated to compare baseline values to the values collected at other time points.

Laboratory data will be analyzed overall and for the age ranges 4 to 5 years, 6 to 8 years and 9 to 11 years.

All laboratory data will be listed. Listing of values that are out of normal range will be flagged in the data listings.

8.4.4. Vital Signs

Observed vital sign values and changes from baseline will be descriptively summarized by visit. All vital signs data will be listed.

The analysis of vital signs will focus on the incidence of clinically relevant abnormalities. The number of subjects evaluated and the number and percentage of subjects with clinically relevant post-baseline abnormalities at each visit will be presented. The criteria for potentially clinically relevant post-baseline abnormalities are shown in Table 10.

Table 10 Criteria of Potentially Clinically Relevant Abnormalities in Vital Signs

Vital Sign Criteria for Abnormalities	Criteria for Abnormalities
Pulse	>130 beats per minute or an increase from pre-dosing of >20 beats per minute, or<br><60 beats per minute or a decrease from pre-dosing of >20 beats per minute
Systolic blood pressure	>130 mmHg or an increase from pre-dosing of >40 mmHg, or<br><70 mmHg or a decrease from pre-dosing of >30 mmHg
Diastolic blood pressure	>85 mmHg or an increase from pre-dosing of >30 mmHg, or<br><45 mmHg or a decrease from pre-dosing of >20 mmHg

Vital signs will be analyzed overall and for the age ranges 4 to 5 years, 6 to 8 years and 9 to 11 years.

8.4.5. Physical Examination

Physical examination data will be summarized by visit and listed.

Changes in physical examination data from baseline will be presented. Physical examination data will be analyzed overall and for the age ranges 4 to 5 years, 6 to 8 years and 9 to 11 years.

Skin reactions observed during the physical examinations will also be reported and the corresponding data will be tabulated separately (see Section 8.5.7).

8.4.6. Spirometry and Peak Expiratory Flow Results

Percent predicted values for FEV_1 and PEF and changes from baseline will be descriptively summarized separately by visit. All FEV_1 and PEF data will be listed. Spirometry and PEF data will be analyzed overall and for the age ranges 4 to 5 years, 6 to 8 years and 9 to 11 years.

8.4.7. Subject Diaries

Subject diary data will be summarized where appropriate and listed.
During the first 6 months of the double-blind period the number of days of itching, redness and swelling of Grade 1, 2, or 3 will be summarized for the overall and separately by age ranges (4 to 5 years, 6 to 8 years and 9 to 11 years).
The most severe grades of itching, redness and swelling, documented in the diary, will also be summarized. The maximum grade of local reactions reported (itching, redness or swelling) during the first 6 months will also be tabulated.
Finally, the percentage of doses during the first 6 months with any local reaction (itching, redness or swelling) reported by the subject will be estimated and summarized by severity, for each treatment group.

8.4.8. Skin Reactions

Viaskin® patch site examination data will be summarized where appropriate and listed.
Examination of the skin at the site of patch application will be graded by the Investigators on a scale of Grade 0 (negative) to Grade 4 (erythema, vesicles). Localization of the skin reactions (under the patch/beyond the patch) will also be collected. These results will be summarized using descriptive statistics and presented by visit, regardless of the localization as well as for each type of localization. The worst grade reported will also be tabulated. The above descriptions will be presented for the overall as well as for the age ranges 4 to 5 years, 6 to 8 years and 9 to 11 years.

8.5. Exploratory Analyses

8.5.1. Immunological markers

Actual values and change from baseline in peanut-specific IgE and IgG4 will be tabulated at each time point. Relative change defined as:
(Value at a specific time point – Baseline value) / Baseline value
will also be presented.
Besides, IgE and IgG4 specific to peanut protein components, and IgE specific to cow's milk, egg, house dust mites, and grass pollen will be summarized at each time point.

8.5.2. Skin Prick Test

Actual values and change from baseline in average wheal diameters of undiluted skin prick will be tabulated at each time point.

8.5.3. Food Allergy Quality of Life Questionnaire /Food Allergy Independent Measure

FAQLQ Quality of life scores (total score as well as score by domain) and changes from baseline will be derived and summarized at screening and after 6, 12, 18, 24, 30, 36 for all subjects and 42 only for subjects initially in the placebo group. FAQLQ data will also be listed.

Data collected from the FAIM questionnaires will help validating of the different forms of FAQLQs, and will be listed.

8.5.4. Accidental Consumption of Peanut-containing Food

Frequency of peanut-containing food consumption, conditions around the consumption: accidental consumption, estimated quantity consumed at each occurrence, and associated reactions and severity of reactions will be analyzed specifically. These AEs will be classified and analyzed separately and specifically.

Risk-taking behaviors will be assessed using the frequency of consumption of peanut reported as deliberate consumptions.

Relatedness to the subject's age category will also be studied.

8.5.5. Epigenetic analyses

Descriptive analyses of epigenetic modifications of specific genes, using observed data will be presented.

8.5.6. Genetic Screening

Treatment-emergent AEs and local skin tolerance at sites of Viaskin® patch application will be described among subjects with mutations in the filaggrin gene *versus* subjects carrying the wild type gene.

8.5.7. Scoring Atopic Dermatitis

SCORAD actual value and change from baseline at each time point will be presented using observed data.

8.5.8. Peanut Food Challenge

The food challenges performed at the initiative of the investigators during the course of the study will be described, such as:

- Peanut ED,
- Peanut CRD,
- Change in CRD from the last historical CRD obtained from a peanut-food challenge performed before entering the study when available to the CRD from the peanut food challenges performed at any time points during the study, Percentage of subjects

reaching a CRD ≥1,000 mg peanut protein at any time point after 12 months of active treatment onwards.

The above descriptions will be presented for food challenge(s) performed at any time during the period of active treatment with Viaskin® Peanut 250 µg, keeping in mind that no peanut food challenge can be conducted for a subject before his/her12 months of active Viaskin® Peanut 250 µg treatment. The following categories will be used:

- From the beginning of the active treatment to 10 months of treatment (food challenges should not occur before 12 months of treatment);
- From Month 10 to Month 18 of the active treatment;
- From Month 18 to Month 30 of the active treatment;
- From Month 30 to the end of the active treatment.

8.6. Interim Analyses

Interim analyses of the safety will be performed at scheduled time points, i.e. when all subjects will reach:

- 6 months of double-blind treatment (e.g. 6 months after the study start for all subjects);
- 12 months of treatment (e.g. 12 or 6 months of active treatment);
- 12 months of active treatment for all subjects (e.g. 12 months after the study start for the subjects randomized in active treatment arm and 18 months after the study start for the subjects initially randomized in placebo treatment arm),
- 24 months of active treatment for all subjects (e.g. 24 months after the study start for the subjects randomized in active treatment arm and 30 months after the study start for the subjects initially randomized in placebo treatment arm),
- 36 months of active treatment for all subjects (e.g. 36 months after the study start for the subjects randomized initially in active treatment arm and 42 months after the study start for the subjects randomized in placebo treatment arm),

Additional analyses may be required for specific request from the DSMB for other needs.

As no formal statistical analysis is planned, no adjustment for multiplicity is needed.

8.7. Determination of Sample Size

The sample size of this study, based on an expected exposure to Viaskin® Peanut 250 µg close to 600 subject-years in the safety database at the time of registration filing, is defined at 250 subjects randomized in the active treatment group. This number of subjects enable the detection events with an annual rate ≥0.024 after 6 months of follow-up in this study and will increase the overall Viaskin® Peanut 250 µg safety database size to a number of subject-years allowing the detection of events with annual incidence rates ≥0.0055.

It is planned to screen approximately 480 subjects to reach 335 subjects randomized in the treatment period of this study (anticipated 30% screen failure rate). Assuming a drop-out rate of 15% per year, this will ensure that approximately 284 subjects will complete the first year

of treatment, 242 subjects will complete the study up to the end of year 2 and 205 subjects will complete the study up to the end of year 3.
The subjects will be randomized at the start of the study with a 3:1 ratio, leading to approximately 250 subjects in the active arm and 85 subjects in the placebo arm.

Throughout the screening process and to ensure an adequate and sufficient representation of the youngest subjects from age range 4 to 6 years of age, a minimum of 90 subjects aged less than 6 years old will be enrolled in the active arm from the 120 total number of randomized subjects in this age range. This will represent a minimum of 36% of the overall number of subjects treated with active treatment at the end of the first 6 months.

9. ETHICAL, LEGAL, AND ADMINISTRATIVE ASPECTS

9.1. Data Quality Assurance

The Sponsor or Sponsor's designee will conduct a site visit or a site phone contact to verify the qualifications of each Investigator, inspect the site facilities, and inform the Investigator of responsibilities and the procedures for ensuring adequate and correct documentation.
The Investigator is required to prepare and maintain adequate and accurate case histories designed to record all observations and other data pertinent to the study for each study participant. All information recorded in the e-CRF for this study must be consistent with the subjects' source documentation (that is medical records).

9.1.1. Database Management and Quality Control

All data generated by the site personnel will be captured electronically at each study center using e-CRFs. Data from external sources (such as laboratory data) will be imported into the database. Once clinical data are entered and validated in the e-CRF, they are transmitted and recorded by the central server in the central database. Computerized edit-checks will be developed in addition to manual review to detect any discrepancies and to ensure consistency of the data. The appropriate staff at the study site will answer queries sent to the Investigator. The reason for changes, the name of the person who performed the changes, together with the time and date will be automatically recorded by the Electronic Data Capture (EDC). An electronic audit trail system will be used to track all data changes in the database subsequent to the first data entry.
If additional corrections are needed, the responsible monitor or data manager or the Sponsor will raise a query in the EDC application. Appropriate feedback to the study staff or other corrective measures will be undertaken if any missing data, inconsistent data, outlier data and potential protocol deviations are identified during routine remote data review.
Once all source data verification is complete and all queries are closed, the monitor will freeze the e-CRF page.

9.2. Case Report Forms and Source Documentation

All data obtained during this study should be entered in the e-CRFs promptly. All source documents from which e-CRF entries are derived should be placed in the subject's medical

records. Measurements for which source documents are usually available include laboratory assessments, spirometry, PEF and immunological markers.
Data that will be entered directly into the e-CRF (those for which there is no prior written or electronic record of data) are considered to be source data.
The original e-CRF entries for each subject may be checked against source documents at the study site by PAREXEL's site monitor.
After review by PAREXEL's site monitor, completed e-CRF entries will be uploaded and forwarded to PAREXEL. Instances of missing or uninterpretable data will be discussed with the Investigator for resolution.
The specific procedures to be used for data entry and query resolution using the e-CRF will be provided to study sites in a training manual. In addition, site personnel will receive training on the e-CRF.

9.2.1. Data Collection

The Investigators (and appropriately authorized staff) will be given access to an online web-based EDC system which is 21 CFR Part 11 compliant. This system is specifically designed for the collection of the clinical data in electronic format. Access and rights to the EDC system will be carefully controlled and configured according to each individual's role throughout the study. In general, only the Investigator and authorized staff will be able to enter data and make corrections in the e-CRFs.
The e-CRF should be completed for each subject for whom the study-specific Informed Consent Form (ICF) was obtained and should reflect the latest observations on the subject participating in the study. Therefore, the e-CRFs are to be completed as soon as possible during or immediately after the subject's visit or assessment. The Investigator must verify that all data entries in the e-CRF are accurate and correct. If some assessments cannot be done, or if certain information is unavailable, not applicable or unknown, the Investigator should indicate this in the e-CRF.
Computerized data-check programs and manual checks will identify any clinical data discrepancies for resolution. Corresponding queries will be loaded into the system and the site will be informed about new issues to be resolved on-line. All discrepancies will be solved on-line directly by the Investigator or by authorized staff. Off-line edit checks will be done to examine relationships over time and across panels to facilitate quality data.
After completion, the Investigator will be required to electronically sign off the clinical data. Information concerning study drug dispensation to the subject will be tracked in the e-CRF.

9.3. Access to Source Data

9.3.1. Routine Monitoring

During the study, a PAREXEL site monitor will conduct site visits to review protocol compliance, compare e-CRF entries and individual subject's medical records, assess drug accountability, and ensure that the study is being conducted according to ethical and pertinent regulatory requirements. The e-CRF entries will be verified with source documentation. The

review of medical records will be performed in a manner to ensure that subject confidentiality is maintained.

Checking of the e-CRF entries for completeness and clarity, and cross-checking with source documents, will be required to monitor the progress of the study. Direct access to source data will be required for the monitoring activities.

9.3.2. Inspections and Auditing Procedures

The Sponsor or its representative may conduct audits at the investigative sites including, but not limited to, drug supply, presence of required documents, the informed consent process, and comparison of e-CRFs with source documents. All medical records (progress notes) must be available for audit. The Investigator agrees to participate in audits conducted at a convenient time in a reasonable manner.

Moreover, local or foreign Regulatory Authorities of certain countries may wish to carry out such source data checks and/or on-site inspections. Direct access to source data will be required for these inspections; they will be carried out giving due consideration to data protection and medical confidentiality.

9.4. Data Processing

All data will be entered by site personnel into the e-CRF (as detailed in Section 9.2.1).

The data-review and data-handling document, to be developed during the initiation phase of the study, will include specifications for consistency and plausibility checks on data and will also include data-handling rules for obvious data errors. Query/correction sheets for unresolved queries will be sent to the study monitors for resolution with the Investigator. The database will be updated on the basis of signed corrections.

Previous and concomitant medications will be coded using the WHO Drug Reference List, which employs the Anatomical Therapeutical Chemical (ATC) classification system. Disease and Medical histories/current medical conditions and AEs will be coded using the MedDRA terminology.

The versions of the coding dictionaries will be provided in the CSR.

9.5. Archiving Study Records

All data derived from the study will remain the property of the Sponsor. Records must be retained in accordance with the current ICH Guidelines on Good Clinical Practice (GCP). All essential study documents including records of subjects, source documents, e-CRFs and study drug inventory must be kept in a study-specific file.

Trial documents should be retained until at least 2 years after the last approval of a marketing application in an ICH region and until there are no pending or planned marketing applications in an ICH region or at least 2 years have elapsed since the formal discontinuation of clinical development of the product.

For this study, the Investigator site files records must be retained by the Investigator at least 15 years after the completion of the trial, or longer if required by their national regulation.

Subjects' medical records must be kept for the maximum period permitted by the hospital, institution or private practice.

The Investigator will not discard any relevant records to this study without prior written permission from the Sponsor. The Investigator shall notify the Sponsor in writing of their intent to destroy all such materials. The Sponsor shall have 30 days to respond to the Investigator's notice, and the Sponsor shall have a further opportunity to retain such materials at the Sponsor's expense.

The Investigator shall take responsibility for maintaining adequate and accurate hard copy source documents of all observations and data generated during this study. Such documentation is subject to inspection by the Sponsor, its representatives and Regulatory Authorities. If an Investigator moves, withdraws from an investigation or retires the responsibility for maintaining the records may be transferred to another person who will accept responsibility. Notice of transfer must be made to and agreed by the Sponsor.

9.6. Good Clinical Practice

The procedures set out in this study protocol are designed to ensure that the Sponsor and Investigator abide by the principles of the ICH-GCP Guidelines, and of the Declaration of Helsinki (APPENDIX 1). The study also will be carried out in keeping with local legal requirements.

9.7. Informed Consent

The Investigator is responsible for and will obtain a signed ICF from each subject's parents/guardians, before each subject is admitted to the study, in accordance with the ICH-GCP Guidelines, the Declaration of Helsinki, and local applicable regulatory requirements.

This consent form must be dated, signed and retained by the Investigator as part of the study records. The Investigator will not undertake any investigation specifically required only for the clinical study until valid consent has been obtained. The terms of the consent and when it was obtained must be documented in the subject's medical records and the e-CRF.

Subjects/parents/guardians will be informed of the nature of the study, its aim, its possible risks and constraints, its expected benefits, its duration, and the compensation that they might receive. The protocol will be explained during a meeting prior to study enrollment, and each subject/parents/guardians must be informed that participation in the study is voluntary and that the subject may withdraw from the study at any time. The parents/guardians should read the ICF before signing and dating it and a copy of the signed document should be given to the parents/guardians. No subject can enter the study before informed consent has been obtained from her/his parents/guardians. Subjects 7 to 11 years of age will sign an assent form, specific to their ages, wherever required by local country laws. The parents/guardians of all subjects regardless of age must sign the ICF.

The explicit wish of a minor, who is capable of forming an opinion and assessing the study information, to refuse participation in or to be withdrawn from the study at any time will be respected by the Investigator.

If a protocol amendment is required, the ICF may need to be revised to reflect the changes to the protocol. If the consent form is revised, it must be reviewed and approved by the appropriate IEC/IRB, and signed by the parents/guardians of all subjects subsequently enrolled in the study as well as those currently enrolled in the study.

The Investigator should inform the subject's primary physician about participation in the clinical study wherever required.

A specific written informed consent for the screening of mutations in the filaggrin gene and for evaluating the epigenetic modifications on some specific genes will also be prepared, submitted and approved by the IRBs/IECs, and has to be signed by the subject's parents/guardians before the blood samples are drawn.

9.8. Protocol Approval and Amendment

Before the start of the study, the study protocol and/or other relevant documents will be reviewed and approved by the IEC/IRB/Regulatory Authorities, in accordance with local legal requirements. The Sponsor must ensure that all ethical and legal requirements have been met before the first subject is enrolled in the study.

This protocol is to be followed exactly. Any change to the protocol must be handled as a protocol amendment. Any potential amendment must be approved by the Sponsor. A written amendment must be submitted to the appropriate Regulatory Authorities and to the IRB/IECs assuming this responsibility. The Investigator must await IRB/IEC approval of substantial protocol amendments before implementing the changes, except where necessary to eliminate apparent immediate hazard to subjects.

All substantial amendments to the protocol must be approved in writing by both the appropriate Regulatory Authorities and the IRB/IEC, except for administrative amendments, which require notification but not written approval. Once approved, the protocol amendment will be distributed to all recipients of the original protocol, with instructions to append the amendment to the protocol. If, in the judgment of the local IRB/IEC, the Investigator and/or Sponsor, the protocol amendment alters the study design, procedures and/or increases the potential risk to the subject, the currently approved written ICF will require modifications. The modified ICF must also be reviewed and approved by the Sponsor, appropriate Regulatory Authorities, and the IRB/IEC. In such cases, repeat informed consent must be obtained from the enrolled subjects' parents/guardians before participation continues.

9.9. Data and Safety Monitoring Board

A DSMB composed of independent experts in food allergy and in the methodology of clinical studies will be established in due time for the first data review. This DSMB will be independent of the Sponsor and will review safety data from the study at specified intervals during the study and on an ad hoc basis as deemed necessary by the DSMB Chair person or when conveyed by the Sponsor.

During these review meetings, the DSMB will assess whether the nature, frequency, and severity of the AEs associated with the study treatment warrant any recommendations or corrective actions of the study conduct in the best interest of the subjects.

The DSMB will also evaluate at each analysis, if the Safety of the sub-Population with history of Severe Anaphylaxis is not different from the Safety sub-Population without history of Severe Anaphylaxis.
The roles, responsibilities, constitution, and operations of the DSMB will be described in the DSMB Charter, which will be reviewed and signed by each member before the first subject is enrolled and treated.

9.10. Duration of the Study

For an individual subject, the maximum duration of study participation will be up to 3.5 years: 2-week screening period, 36 months of treatment (active arm) to 42 months (6 months + 36 months for placebo arm) and 2-week follow-up period. The planned study duration is approximately 4 years.

9.11. Premature Termination of the Study

If the Investigator, the Sponsor, or the Medical Monitor becomes aware of conditions or events that suggest a possible hazard to subjects if the study continues, the study may be terminated after appropriate consultation between the relevant parties. The study may also be terminated early at the Sponsor's discretion in the absence of such a finding.
Conditions that may warrant termination include, but are not limited to:

- The discovery of an unexpected, significant, or unacceptable risk to the subjects enrolled in the study;
- Failure to enroll subjects at an acceptable rate;
- A decision on the part of the Sponsor to suspend or discontinue development of the drug;
- A decision from the Regulatory Authorities to suspend or discontinue the study.

In terminating the study, the Sponsor and the Investigator will ensure that adequate consideration is given to the protection of the subjects' interests.

9.12. Confidentiality

All information including the skin photos, obtained during the conduct of the study with respect to the subject's state of health will be regarded as confidential. For disclosure of any such information, an agreement will be obtained in writing. The Investigator must ensure that each subject's anonymity is maintained. On e-CRFs, the other documents and the skin photos transmitted to the Sponsor or PAREXEL, subjects must not be identified by name. Instead, subjects will only be known by the unique subject screening number allocated to them in order to ensure confidentiality on all study documentation. Subjects will retain this unique number throughout the study. The Investigator will keep a separate log of these codes.
In order to comply with government regulatory guidelines and to ensure subject safety, it may be necessary for the Sponsor and its representative, the IEC/IRB, or Regulatory Authority to review subjects' medical records as they relate to this study. Only the subject's unique number in the e-CRFs will identify her/him, but their full names may be made known

to a Regulatory Authority or other authorized government or health care officials, if necessary, and to personnel designated by the Sponsor.

Documents that are not for submission to the Sponsor or to PAREXEL (for example consent forms) will be maintained by the Investigator in strict confidence, except to the extent necessary to allow monitoring by the Sponsor and PAREXEL, and auditing by regulatory authorities. No documents identifying subjects by name will leave the investigative site and subject identity will remain confidential in all publications related to the study.

9.13. Contractual and Financial Details

The Investigator (and/or, as appropriate, the hospital administrative representative) and the Sponsor will sign a clinical study agreement prior to the start of the study, outlining overall Sponsor and Investigator responsibilities in relation to the study. The contract should describe whether costs for pharmacy, laboratory and other protocol-required services are being paid directly or indirectly.

Financial Disclosure Statements will need to be completed, as requested by FDA CFR 21 part 54.

9.14. Liability and Insurance

The Sponsor will take out reasonable third-party liability insurance coverage in accordance with all local legal requirements. The civil liability of the Investigator, the persons instructed by her/him and the hospital, practice, or institute in which they are employed and the liability of the Sponsor with respect to financial loss due to personal injury and other damage that may arise as a result of the carrying out of this study are governed by the applicable law.

Deviations from the study protocol, especially the prescription of a dose other than that scheduled in the study protocol, other modes of administration, other indications, and longer treatment periods, are not permitted and shall not be covered by the statutory subject insurance scheme.

The Sponsor will arrange for subjects participating in this study to be insured against financial loss due to personal injury caused by the pharmaceutical products being tested or by medical steps taken in the course of the study.

9.15. Publication Policy

By signing the study protocol, the Investigator agrees with the use of results of the study for the purposes of national and international registration, publication and information for medical and pharmaceutical professionals. If necessary, Regulatory Authorities will be notified of the Investigator's name, address, qualifications and extent of involvement.

An Investigator shall not publish any data (poster, abstract, paper, etc.) without having consulted with the Sponsor in advance and having received a written approval for such a publication. Details are provided in a separate document.

9.16. Critical Documents

Before the Investigator starts the trial, the following documents must be available to DBV Technologies:

- Regulatory approval;
- Curricula vitae of Investigator and Sub-investigator(s) (current, dated and signed);
- Favourable opinion from IRB/IEC clearly identifying the documents reviewed: the protocol, any substantial amendments, subject information/informed consent form and any other written information to be provided to the subject, subject recruitment procedures;
- List of IRB/IEC members/constitution;
- Signed and dated agreement on the final protocol;
- Signed and dated agreement on any substantial amendment(s), if applicable;
- Copy of IRB/IEC approved subject information/informed consent form/any other written information/advertisement;
- Signed receipt of IB by Investigator;
- Laboratory certification and normal ranges;
- Financial agreement(s);
- FDA financial disclosure form (as the trial is conducted under an Investigational New Drug Application (IND)).

9.17. Clinical Study Report

An interim CSR will be prepared after the 6- month interim analysis as well as the final CSR, according to the ICH E3 guideline on Structure and Contents of CSRs. A final CSR will be prepared regardless of whether the study is completed or prematurely terminated. The Sponsor will provide each Investigator with a copy of the final report or synopsis for retention.

10. REFERENCES

1. Bock SA, Muñoz-Furlong A, Sampson HA. Further fatalities caused by anaphylactic reactions to food, 2001-2006. J. Allergy Clin. Immunol. 2007;119:1016–8.

2. Bock SA, Muñoz-Furlong A, Sampson HA. Fatalities due to anaphylactic reactions to foods. J. Allergy Clin. Immunol. 2001;107:191–3.

3. Sicherer SH, Muñoz-Furlong A, Godbold JH, Sampson HA. US prevalence of self-reported peanut, tree nut, and sesame allergy:, 11-year follow-up. J. Allergy Clin. Immunol. 2010;125:1322–6.

4. Sicherer SH, Muñoz-Furlong A, Sampson HA. Prevalence of peanut and tree nut allergy in the United States determined by means of a random digit dial telephone survey:, a 5-year follow-up study. J. Allergy Clin. Immunol. 2003;112:1203–7.

5. Grundy J, Matthews SM, Bateman B, Dean TP, Arshad SH. Rising prevalence of allergy to peanut in children:, data from 2 sequential cohorts. J. Allergy Clin. Immunol. 2002;110:784–9.

6. Fleischer DM, Conover-Walker MK, Christie L, Burks AW, Wood RA. The natural progression of peanut allergy:, resolution and the possibility of recurrence. J. Allergy Clin. Immunol. 2003;112:183–9.

7. Ho MHK, Wong WHS, Heine RG, Hosking CS, Hill DJ, Allen KJ. Early clinical predictors of remission of peanut allergy in children. J. Allergy Clin. Immunol. 2008;121:731–6.

8. O'B Hourihane J, Roberts SA, Warner JO. Resolution of peanut allergy:, case-control study. BMJ 1998;316:1271–5.

9. Peters RL, Allen KJ, Dharmage SC, et al. Natural history of peanut allergy and predictors of resolution in the first 4 years of life:, a population-based assessment. J. Allergy Clin. Immunol. 2015;135:1257–66.

10. Du Toit G, Roberts GC, Sayre PH, et al. Randomized trial of peanut consumption in infants at risk for peanut allergy. N. Engl. J. Med. 2015;372:803–13.

11. Eigenmann PA, Beyer K, Burks AW, et al. New visions for food allergy:, an iPAC summary and future trends. Pediatr. Allergy Immunol. 2008;19 Suppl 19:26–39.

12. Sampson HA. Food allergy-accurately identifying clinical reactivity. Allergy 2005;60 Suppl 79:19–24.

13. Sampson HA, Muñoz-Furlong A, Campbell RL, et al. Second symposium on the definition and management of anaphylaxis: symposium, summary report-Second National Institute of Allergy and Infectious Disease/Food Allergy and Anaphylaxis Network. J. Allergy Clin. Immunol. 2006;117:391–7.

14. Burks AW. Peanut allergy. Lancet 2008;371:1538–46.

15. Eigenmann PA. Mechanisms of food allergy. Pediatr. Allergy Immunol. 2009;20:5–11.

16. Otsu K, Dreskin SC. Peanut allergy:, an evolving clinical challenge. Discov Med 2011;12:319–28.

17. Sicherer SH, Furlong TJ, Muñoz-Furlong A, Burks AW, Sampson HA. A voluntary registry for peanut and tree nut allergy:, characteristics of the first 5149 registrants. J. Allergy Clin. Immunol. 2001;108:128–32.

18. Kemp SF, Lockey RF, Simons FER. Epinephrine:, the drug of choice for anaphylaxis. A statement of the World Allergy Organization. Allergy 2008;63:1061–70.

19. Leung DYM, Sampson HA, Yunginger JW, et al. Effect of anti-IgE therapy in patients with peanut allergy. N. Engl. J. Med. 2003;348:986–93.

20. Sampson HA, Leung DYM, Burks AW, et al. A phase II, randomized, double blind, parallel group, placebo controlled oral food challenge trial of Xolair (omalizumab) in peanut allergy. J. Allergy Clin. Immunol. 2011;127:1309–10.

21. Jones SM, Burks AW, Dupont C. State of the art on food allergen immunotherapy:, oral, sublingual, and epicutaneous. J. Allergy Clin. Immunol. 2014;133:318–23.

22. Leung DYM. Food allergy:, are we getting closer to a cure? J. Allergy Clin. Immunol. 2011;127:555–7.

23. Anagnostou K, Islam S, King Y, et al. Assessing the efficacy of oral immunotherapy for the desensitisation of peanut allergy in children (STOP II):, a phase 2 randomised controlled trial. Lancet 2014;383:1297–304.

24. Kim EH, Bird JA, Kulis MD, et al. Sublingual immunotherapy for peanut allergy: clinical and immunologic evidence of desensitization. J. Allergy Clin. Immunol. 2011;127:640–6.

25. Varshney P, Jones SM, Scurlock AM, et al. A randomized controlled study of peanut oral immunotherapy (OIT):, clinical desensitization and modulation of the allergic response. J. Allergy Clin. Immunol. 2011;127:654–60.

26. Jones SM, Pons L, Roberts JL, et al. Clinical efficacy and immune regulation with peanut oral immunotherapy. J. Allergy Clin. Immunol. 2009;124:292-300, 300.

27. Blümchen K, Ulbricht H, Staden U, et al. Oral peanut immunotherapy in children with peanut anaphylaxis. J. Allergy Clin. Immunol. 2010;126:83–91.

28. Vickery BP, Scurlock AM, Steele PH, et al. Early and persistent gastrointestinal side effects predict withdrawal from peanut oral immunotherapy (OIT). J. Allergy Clin. Immunol. 2011;127:AB26.

29. Wasserman RL, Factor JM, Baker JW, et al. Oral immunotherapy for peanut allergy: multipractice experience with epinephrine-treated reactions. J. Allergy Clin. Immunol. Pract. 2014;2:91–6.

30. Wasserman RL, Sugerman RW, Mireku-Akomeah N, Gallucci AR, Pence DM, Long NA. Peanut oral immunotherapy (OIT) of food allergy (FA) carries a significant risk of eosinophilic esophagitis (EoE). J. Allergy Clin. Immunol. 2011;127:AB28.

31. Hsieh FH. Oral food immunotherapy and iatrogenic eosinophilic esophagitis: an acceptable level of risk? Ann. Allergy Asthma Immunol. 2014;113:581–2.

32. Lucendo AJ, Arias Á, Tenias JM. Relation between eosinophilic esophagitis and oral immunotherapy for food allergy:, a systematic review with meta-analysis. Ann. Allergy Asthma Immunol. 2014;113:624–9.

33. Investigator's Brochure Viaskin Peanut (DBV712). Edition Number 8. 2016 Aug 19.

34. Jones SM, Agbotounou WK, Fleischer DM, et al. Safety of epicutaneous immunotherapy for the treatment of peanut allergy: a phase 1 study using the Viaskin patch. J. Allergy Clin. Immunol. 2016;137:1258-1261.e10. (Accessed May 9, 2016).

35. Bethesda MD. Expert Panel Report 3: Guidelines for the Diagnosis and Management of Asthma, National Asthma Education and Prevention Program, Third

Expert Panel on the Diagnosis and Management of Asthma. Report No.: 07-4051. 2007 Aug.

36. Global Initiative for Asthma., Global Strategy for Asthma Management and Prevention. Available from http://ginasthma.org/gina-reports/. Date of last update: 2016.

37. Turjanmaa K, Darsow U, Niggemann B, Rancé F, Vanto T, Werfel T. EAACI/GA2LEN position paper:, present status of the atopy patch test. Allergy 2006;61:1377–84.

38. Goossens NJ, Flokstra-de Blok BMJ, van der Meulen GN, et al. Health-related quality of life in food-allergic adults from eight European countries. Ann. Allergy Asthma Immunol. 2014;113:63–8.

39. van der Velde JL, Flokstra-de Blok BMJ, Vlieg-Boerstra BJ, et al. Development, validity and reliability of the food allergy independent measure (FAIM). Allergy 2010;65:630–5.

40. Kunz B, Oranje AP, Labreze L, Stalder J-F, Ring J, Taïeb A. Clinical validation and guidelines for the SCORAD index: consensus report of the European Task Force on Atopic Dermatitis. Dermatology (Basel) 1997;195:10–9.

11. APPENDICES

**APPENDIX 1**	Declaration of Helsinki
**APPENDIX 2**	Dosages of Inhaled Corticosteroids
**APPENDIX 3**	Activity of Corticosteroids
**APPENDIX 4**	Wash-out periods for Short-acting and Long-acting Antihistamines based on Terminal Elimination Half-Lives
**APPENDIX 5**	Anaphylaxis Staging System
**APPENDIX 6**	Proposed Oral Food Challenge Procedure and Symptom Score Sheets
**APPENDIX 7**	FAQLQ/FAIM Questionnaires
**APPENDIX 8**	SCORAD

APPENDIX 1

Declaration of Helsinki

WORLD MEDICAL ASSOCIATION DECLARATION OF HELSINKI

Ethical Principles for Medical Research Involving Human Subjects

Adopted by the 18th WMA General Assembly, Helsinki, Finland, June 1964 and amended by the:

29th WMA General Assembly, Tokyo, Japan, October 1975
35th WMA General Assembly, Venice, Italy, October 1983
41st WMA General Assembly, Hong Kong, September 1989
48th WMA General Assembly, Somerset West, Republic of South Africa, October 1996
52nd WMA General Assembly, Edinburgh, Scotland, October 2000
53rd WMA General Assembly, Washington DC, USA, October 2002 (Note of Clarification added)
55th WMA General Assembly, Tokyo, Japan, October 2004 (Note of Clarification added)
59th WMA General Assembly, Seoul, Republic of Korea, October 2008
64th WMA General Assembly, Fortaleza, Brazil, October 2013

Preamble

1. The World Medical Association (WMA) has developed the Declaration of Helsinki as a statement of ethical principles for medical research involving human subjects, including research on identifiable human material and data.

The Declaration is intended to be read as a whole and each of its constituent paragraphs should be applied with consideration of all other relevant paragraphs.

2. Consistent with the mandate of the WMA, the Declaration is addressed primarily to physicians. The WMA encourages others who are involved in medical research involving human subjects to adopt these principles.

General Principles

3. The Declaration of Geneva of the WMA binds the physician with the words, “The health of my patient will be my first consideration,” and the International Code of Medical Ethics declares that, “A physician shall act in the patient's best interest when providing medical care.”

4. It is the duty of the physician to promote and safeguard the health, well-being and rights of patients, including those who are involved in medical research. The physician's knowledge and conscience are dedicated to the fulfilment of this duty.

5. Medical progress is based on research that ultimately must include studies involving human subjects.

6. The primary purpose of medical research involving human subjects is to understand the causes, development and effects of diseases and improve preventive, diagnostic and therapeutic interventions (methods, procedures and treatments). Even the best proven interventions must be evaluated continually through research for their safety, effectiveness, efficiency, accessibility and quality.

7. Medical research is subject to ethical standards that promote and ensure respect for all human subjects and protect their health and rights.

8. While the primary purpose of medical research is to generate new knowledge, this goal can never take precedence over the rights and interests of individual research subjects.

9. It is the duty of physicians who are involved in medical research to protect the life, health, dignity, integrity, right to self-determination, privacy, and confidentiality of personal information of research subjects. The responsibility for the protection of research subjects must always rest with the physician or other health care professionals and never with the research subjects, even though they have given consent.

10. Physicians must consider the ethical, legal and regulatory norms and standards for research involving human subjects in their own countries as well as applicable international norms and standards. No national or international ethical, legal or regulatory requirement should reduce or eliminate any of the protections for research subjects set forth in this Declaration.

11. Medical research should be conducted in a manner that minimises possible harm to the environment.

12. Medical research involving human subjects must be conducted only by individuals with the appropriate ethics and scientific education, training and qualifications. Research on patients or healthy volunteers requires the supervision of a competent and appropriately qualified physician or other health care professional.

13. Groups that are underrepresented in medical research should be provided appropriate access to participation in research.

14. Physicians who combine medical research with medical care should involve their patients in research only to the extent that this is justified by its potential preventive, diagnostic or therapeutic value and if the physician has good reason to believe that participation in the research study will not adversely affect the health of the patients who serve as research subjects.

15. Appropriate compensation and treatment for subjects who are harmed as a result of participating in research must be ensured.

Risks, Burdens and Benefits

16. In medical practice and in medical research, most interventions involve risks and burdens.

Medical research involving human subjects may only be conducted if the importance of the objective outweighs the risks and burdens to the research subjects.

17. All medical research involving human subjects must be preceded by careful assessment of predictable risks and burdens to the individuals and groups involved in the research in comparison with foreseeable benefits to them and to other individuals or groups affected by the condition under investigation.

Measures to minimise the risks must be implemented. The risks must be continuously monitored, assessed and documented by the researcher.

18. Physicians may not be involved in a research study involving human subjects unless they are confident that the risks have been adequately assessed and can be satisfactorily managed.

When the risks are found to outweigh the potential benefits or when there is conclusive proof of definitive outcomes, physicians must assess whether to continue, modify or immediately stop the study.

Vulnerable Groups and Individuals

19. Some groups and individuals are particularly vulnerable and may have an increased likelihood of being wronged or of incurring additional harm.

All vulnerable groups and individuals should receive specifically considered protection.

20. Medical research with a vulnerable group is only justified if the research is responsive to the health needs or priorities of this group and the research cannot be carried out in a non-vulnerable group. In addition, this group should stand to benefit from the knowledge, practices or interventions that result from the research.

Scientific Requirements and Research Protocols

21. Medical research involving human subjects must conform to generally accepted scientific principles, be based on a thorough knowledge of the scientific literature, other relevant sources of information, and adequate laboratory and, as appropriate, animal experimentation. The welfare of animals used for research must be respected.

22. The design and performance of each research study involving human subjects must be clearly described and justified in a research protocol.

The protocol should contain a statement of the ethical considerations involved and should indicate how the principles in this Declaration have been addressed. The protocol should include information regarding funding, sponsors, institutional affiliations, potential conflicts of interest, incentives for subjects and information regarding provisions for treating and/or compensating subjects who are harmed as a consequence of participation in the research study.

In clinical trials, the protocol must also describe appropriate arrangements for post-trial provisions.

Research Ethics Committees

23. The research protocol must be submitted for consideration, comment, guidance and approval to the concerned research ethics committee before the study begins. This committee must be transparent in its functioning, must be independent of the researcher, the sponsor and any other undue influence and must be duly qualified. It must take into consideration the laws and regulations of the country or countries in which the research is to be performed as well as applicable international norms and standards but these must not be allowed to reduce or eliminate any of the protections for research subjects set forth in this Declaration.

The committee must have the right to monitor ongoing studies. The researcher must provide monitoring information to the committee, especially information about any serious adverse events. No amendment to the protocol may be made without consideration and approval by the committee. After the end of the study, the researchers must submit a final report to the committee containing a summary of the study's findings and conclusions.

Privacy and Confidentiality

24. Every precaution must be taken to protect the privacy of research subjects and the confidentiality of their personal information.

Informed Consent

25. Participation by individuals capable of giving informed consent as subjects in medical research must be voluntary. Although it may be appropriate to consult family members or community leaders, no individual capable of giving informed consent may be enrolled in a research study unless he or she freely agrees.

26. In medical research involving human subjects capable of giving informed consent, each potential subject must be adequately informed of the aims, methods, sources of funding, any possible conflicts of interest, institutional affiliations of the researcher, the anticipated benefits and potential risks of the study and the discomfort it may entail, post-study provisions and any other relevant aspects of the study. The potential subject must be informed of the right to refuse to participate in the study or to withdraw consent to participate at any time without reprisal. Special attention should be given to the specific information needs of individual potential subjects as well as to the methods used to deliver the information.

After ensuring that the potential subject has understood the information, the physician or another appropriately qualified individual must then seek the potential subject's freely-given informed consent, preferably in writing. If the consent cannot be expressed in writing, the non-written consent must be formally documented and witnessed.

All medical research subjects should be given the option of being informed about the general outcome and results of the study.

27. When seeking informed consent for participation in a research study the physician must be particularly cautious if the potential subject is in a dependent relationship with the physician or may consent under duress. In such situations the informed consent must be sought by an appropriately qualified individual who is completely independent of this relationship.

28. For a potential research subject who is incapable of giving informed consent, the physician must seek informed consent from the legally authorised representative. These individuals must not be included in a research study that has no likelihood of benefit for them unless it is intended to promote the health of the group represented by the potential subject, the research cannot instead be performed with persons capable of providing informed consent, and the research entails only minimal risk and minimal burden.

29. When a potential research subject who is deemed incapable of giving informed consent is able to give assent to decisions about participation in research, the physician must seek that assent in addition to the consent of the legally authorised representative. The potential subject's dissent should be respected.

30. Research involving subjects who are physically or mentally incapable of giving consent, for example, unconscious patients, may be done only if the physical or mental condition that prevents giving informed consent is a necessary characteristic of the research group. In such circumstances the physician must seek informed consent from the legally authorised representative. If no such representative is available and if the

research cannot be delayed, the study may proceed without informed consent provided that the specific reasons for involving subjects with a condition that renders them unable to give informed consent have been stated in the research protocol and the study has been approved by a research ethics committee. Consent to remain in the research must be obtained as soon as possible from the subject or a legally authorised representative.

31. The physician must fully inform the patient which aspects of their care are related to the research. The refusal of a patient to participate in a study or the patient's decision to withdraw from the study must never adversely affect the patient-physician relationship.

32. For medical research using identifiable human material or data, such as research on material or data contained in biobanks or similar repositories, physicians must seek informed consent for its collection, storage and/or reuse. There may be exceptional situations where consent would be impossible or impracticable to obtain for such research. In such situations the research may be done only after consideration and approval of a research ethics committee.

Use of Placebo

33. The benefits, risks, burdens and effectiveness of a new intervention must be tested against those of the best proven intervention(s), except in the following circumstances:

Where no proven intervention exists, the use of placebo, or no intervention, is acceptable; or

Where for compelling and scientifically sound methodological reasons the use of any intervention less effective than the best proven one, the use of placebo, or no intervention is necessary to determine the efficacy or safety of an intervention

and the patients who receive any intervention less effective than the best proven one, placebo, or no intervention will not be subject to additional risks of serious or irreversible harm as a result of not receiving the best proven intervention.

Extreme care must be taken to avoid abuse of this option.

Post-Trial Provisions

34. In advance of a clinical trial, sponsors, researchers and host country governments should make provisions for post-trial access for all participants who still need an intervention identified as beneficial in the trial. This information must also be disclosed to participants during the informed consent process.

Research Registration and Publication and Dissemination of Results

35. Every research study involving human subjects must be registered in a publicly accessible database before recruitment of the first subject.

36. Researchers, authors, sponsors, editors and publishers all have ethical obligations with regard to the publication and dissemination of the results of research. Researchers have a duty to make publicly available the results of their research on human subjects and are accountable for the completeness and accuracy of their reports. All parties should adhere to accepted guidelines for ethical reporting. Negative and inconclusive as well as positive results must be published or otherwise made publicly available. Sources of funding, institutional affiliations and conflicts of interest must be

declared in the publication. Reports of research not in accordance with the principles of this Declaration should not be accepted for publication.

Unproven Interventions in Clinical Practice

37. In the treatment of an individual patient, where proven interventions do not exist or other known interventions have been ineffective, the physician, after seeking expert advice, with informed consent from the patient or a legally authorized representative, may use an unproven intervention if in the physician's judgement it offers hope of saving life, re-establishing health or alleviating suffering. This intervention should subsequently be made the object of research, designed to evaluate its safety and efficacy. In all cases, new information must be recorded and, where appropriate, made publicly available.

APPENDIX 2

Dosages of Inhaled Corticosteroids

Children ≤4 years of age	Low Daily Dose	Medium Daily Dose	High Daily Dose
**Beclomethasone HFA**<br>40 or 80 mcg/puff	NA	NA	NA
**Budesonide DPI**<br>90, 180, or 200 mcg/inhalation	NA	NA	NA
**Budesonide Inhaled**<br>Inhalation suspension for nebulization	0.25-0.5 mg	>0.5-1.0 mg	>1.0 mg
**Flunisolide**<br>250 mcg/puff	NA	NA	NA
**Flunisolide HFA**<br>80 mcg/puff	NA	NA	NA
**Fluticasone HFA/MDI**<br>44, 110, or 220 mcg/puff	176 mcg	>176-352 mcg	>352 mcg
**DPI**<br>50, 100, or 250 mcg/inhalation	NA	NA	NA
**Mometasone DPI** 200 mcg/inhalation	NA	NA	NA
**Triamcinolone acetonide**<br>75 mcg/puff	NA	NA	NA
**Children 5 to 11 years of age**	**Low Daily Dose**	**Medium Daily Dose**	**High Daily Dose**
**Beclomethasone HFA**<br>40 or 80 mcg/puff	80–160 mcg	>160–320 mcg	>320 mcg
**Budesonide DPI**<br>90, 180, or 200 mcg/inhalation	180–400 mcg	>400–800 mcg	>800 mcg
**Budesonide Inhaled**<br>Inhalation suspension for nebulization	0.5 mg	1.0 mg	2.0 mg
**Flunisolide**<br>250 mcg/puff	500–750 mcg	1,000–1,250 mcg	>1,250 mcg
**Flunisolide HFA**<br>80 mcg/puff	160 mcg	320 mcg	≥640 mcg
**Fluticasone HFA/MDI**<br>44, 110, or 220 mcg/puff	88–176 mcg	>176–352 mcg	>352 mcg
**DPI**<br>50, 100, or 250 mcg/inhalation	100–200 mcg	>200–400 mcg	>400 mcg
**Mometasone DPI** 200 mcg/inhalation	NA	NA	NA
**Triamcinolone acetonide**<br>75 mcg/puff	300–600 mcg	>600–900 mcg	>900 mcg

Abbreviations: DPI = Dry powder inhaler; HFA = Hydrofluoroalkane; MDI = Metered-dose inhaler; NA=Not approved and no data available for this age category.

**Adolescents ≥12 years of age and Adults**	**Low Daily Dose**	**Medium Daily Dose**	**High Daily Dose**
**Beclomethasone HFA**<br>40 or 80 mcg/puff	80–240 mcg	>240–480 mcg	>480 mcg
**Budesonide DPI**<br>90, 180, or 200 mcg/inhalation	180–600 mcg	>600–1,200 mcg	>1,200 mcg
**Budesonide Inhaled**<br>Inhalation suspension for nebulization	NA	NA	NA
**Flunisolide**<br>250 mcg/puff	500–1,000 mcg	>1,000–2,000 mcg	>2,000 mcg
**Flunisolide HFA**<br>80 mcg/puff	320 mcg	>320–640 mcg	>640 mcg
**Fluticasone HFA/MDI**<br>44, 110, or 220 mcg/puff	88–264 mcg	>264–440 mcg	>440 mcg
**DPI**<br>50, 100, or 250 mcg/inhalation	100–300 mcg	>300–500 mcg	>500 mcg
**Mometasone DPI**<br>200 mcg/inhalation	200 mcg	400 mcg	>400 mcg
**Triamcinolone acetonide**<br>75 mcg/puff	300–750 mcg	>750–1,500 mcg	>1,500 mcg

Abbreviations: DPI = Dry powder inhaler; HFA = Hydrofluoroalkane; MDI = Metered-dose inhaler; NA=Not approved and no data available for this age category.

Source: National Heart, Lung, and Blood Institute. Expert panel report 3: guidelines for the diagnosis and management of asthma—full report 2007. August 28, 2007. Available at: www.nhlbi.nih.gov/guidelines/asthma/asthgdln.pdf. Accessed July 06, 2015.

APPENDIX 3

Activity of Corticosteroids

Relative Potencies of Systemic Glucocorticoids			
CORTICOSTEROID	*ACTIVITY*	*RELATIVE POTENCY*	*EQUIVALENT DOSE (MG)*
Dexamethasone	Long-Acting	25	0.75
Prednisone	Intermediate-acting	4	5.0
Methylprednisolone	Intermediate-acting	5	4.0
Hydrocortisone	Short-Acting	1.0	20.0
Information from Drug facts and comparisons. St Louis: Facts and Comparisons, 1997:122-23.			

APPENDIX 4

Wash-out periods for Short-acting and Long-acting Antihistamines based on Terminal Elimination Half-Lives

**Short-acting antihistamines**	Terminal elimination half-life (h)	Minimum days of wash-out prior to skin prick tests or oral food challenges (days)
Oral		
acrivastine[1]	2-4	1[3] or ideally 3
cetirizine[1]	6.2 - 10	Must be 5[4]
cimetidine[1]	1.4	1[3] or ideally 3
diphenhydramine[1]	2-8	1[3] or ideally 3
famotidine[1]	0.8-5.8	1[3] or ideally 3
fexofenadine[1]	14.4	3
hydroxyzine[1]	4.8-9.4	Must be 5[4]
levocetirizine[1]	6	3
nizatadine[1]	1-2	1[3] or ideally 3
mizolastine[2]	12.9	3
ranitidine[1]	2.5-3	1[3] or ideally 3
rupatadine[2]	13	3
Intranasal/Ophthalmic		
emedastine[1]	3-4	1[3] or ideally 3
epinastine[1]	12	3
olopatadine[1]	8-12	3

**Long-acting antihistamines**	Terminal elimination half-life (h)	Wash-out period prior to skin prick tests or oral food challenges (days)
Oral		
desloratadine[1]	27	7
ebastine[2]	19.3	5
chlorpheniramine[1]	6.3-23.1	6
ketotifen[1]	21	5
loratadine[1]	28	7
Intranasal/Ophthalmic		
azelastine[1]	22-25	6
ketotifen[1]	21	5
levocabastine[1]	35-40	7

[1] *Lexicomp Online®, In: UpToDate, Waltham, MA. (Accessed on November 17, 2015.)*

[2] *Derived from: Allergy: Principles and Practice (Middleton, 7th Edition) in the form of Table 87.4 (authored by FER Simons and C Akdis).*

[3] *If the antihistamine is used the day prior to an oral food challenge, a Skin Prick Test must be performed before the initiation of the challenge and the result must be positive, i.e. a genuine wheal with the histamine positive control must be obtained before the challenge is effectively initiated. If not, postpone the challenge.*

[4] *Wash-out period extended beyond 3 days based on actual clinical experience.*

APPENDIX 5

Anaphylaxis Staging System
Anaphylaxis is a generalized allergic reaction that is rapid in onset and may progress to death (Adapted from [1]).

Staging System of Severity of Anaphylaxis	
**Stage**	**Defined by**
**1 Mild**<br>skin & subcutaneous tissues, GI, &/or mild respiratory	Flushing, urticaria, periorbital or facial angioeodema; mild dyspnea, wheeze or upper respiratory symptoms; mild abdominal pain and/or emesis
**2 Moderate**<br>mild symptoms + features suggesting moderate respiratory, cardiovascular or GI symptoms	Marked dysphagia, hoarseness, and/or stridor; shortness of breath, wheezing & retractions; crampy abdominal pain, recurrent vomiting and/or diarrhea; and/or mild dizziness
**3 Severe**<br>hypoxia,<br>hypotension (more than 20% drop in blood pressure) or<br>neurological compromise	Cyanosis or SpO2 ≤ 92% at any stage, confusion, cardiovascular collapse, loss of consciousness, incontinence, bradychardia, cardiac arrest.

[1] Sampson HA, Muñoz-Furlong A, Campbell RL, Adkinson NF Jr, Bock SA, Branum A, et al. Second symposium on the definition and management of anaphylaxis: summary report—Second National Institute of Allergy and Infectious Disease/Food Allergy and Anaphylaxis Network symposium. J Allergy Clin Immunol. 2006;117(2):391-7.

APPENDIX 6

Oral Food Challenge Procedure and Symptom Score Sheet

Oral Food Challenge Procedure

For ensuring the subject's medical management, the investigators may need to perform peanut-food challenge during the course of the study, based on their own medical judgment.

A maximum of 2 open peanut-food challenges for the same subject is recommended during the subject treatment course up to the 36 months of active treatment. Performing open food challenge(s) in the REALISE study is optional and is left to the investigator's decision. However, no food challenge can occur before the subject has received 12 months of active treatment with Viaskin® Peanut 250 µg. If 2 peanut challenges are performed for the same subject within the treatment course, the second challenge can occur only after at least 12 additional months of active treatment have been administered to that subject.

If the decision is made by the investigators to perform a peanut-food challenge, its performance must comply with the standardized methodology defined in the protocol APPENDIX 6. A standardized peanut food challenge formula and material as well as the Manual of Procedures will be provided to all sites, and must be used for conducting the open peanut food challenge.

All available food challenges results will be described, when available, such as:

- Eliciting Dose (ED),
- Cumulative Reactive Dose (CRD),
- Type and severity of the allergic symptoms that occur during the food challenge.

Challenges must take place under direct medical supervision in a hospital/clinic setting with resuscitation equipment and emergency medications and staff immediately available. An IV line can be established prior to the challenge when judged necessary by the Investigator. In that case, a local anesthetic cream can be used for establishing the IV line. If a site prefers to have the subjects admitted to the hospital the day before the conduct of the food challenge, to get them ready for the following day, this is allowed and will not be considered a serious adverse event (SAE).

Subjects should be off antihistamines depending of its half-life (refer to Appendix 4) prior to the food challenge.

Subjects will not be allowed to use long-acting β2-agonists within 36 hours prior to the food challenge. Subjects who received more than a 3-day course of systemic corticosteroids within 4 weeks of the food challenge should have the food challenge delayed to allow for a minimum of 4-week wash-out.

The subject should have a light breakfast and may drink water at home at least 2 hours before starting the food challenge at the site. During the conduct of the challenge, no food other than the challenge formulas should be consumed by the subject. Drinking a sip of water to help swallow the formula during the challenge, if necessary, is allowed. The subject will be gradually fed increasing amounts of standardized oral formulas containing peanut.

After the last dose of the challenge formula is administered, the medical staff should wait at least 1 hour before feeding the subject with any other food and/or water. This first feeding should be light.

Preparation of Peanut Formulas

Standardized peanut formulas centrally produced by AMATSI, Montpellier, France, and packed and released by CREAPHARM, Bordeaux, France, will be provided to the participating centers.
A detailed and study-specific Manual of Procedures for the conduct of the peanut food challenge will be provided to the Investigators, the site staff and the study-trained person responsible for reconstituting the formula.

Time Interval and Doses

The challenge will consist of giving to the subjects increasing doses of peanut protein at 30-minute intervals (± 5 min). This standard interval has been previously used safely, but the Investigator may use clinical judgment to increase the intervals between doses if there is a concern that an objective reaction may be developing.

The starting dose will be 1 mg of peanut protein, and the peanut protein dose increments should be: 1 mg, 3 mg, 10 mg, 30 mg, 100 mg, 300 mg, 1,000 mg, 2,000 mg and again another last dose of 2,000 mg to reach a cumulative dose of 5,444 mg peanut protein.

Only **clear-cut OBJECTIVE immediate-type symptom(s)** requiring treatment will be considered as a reason to stop the challenge and to determine the eliciting dose (highest dose given during the challenge) as well as the CRD of peanut protein. **Subjective symptoms will be graded but will not be considered in the stopping rules of the challenge.**

The Oral Food-Challenge (OFC) Symptom Score Sheet will be used to score the severity of each pre-specified objective and subjective symptoms. This scoring is classified under the 5 categories:

- I Skin, (Erythematous Rash, Pruritus, Urticaria/angioedema, Rash)
- II Upper respiratory, (Sneezing/Itching, Nasal Congestion, Rhinorrhea, Laryngeal)
- III Lower Respiratory, (Wheezing)
- IV Gastrointestinal, (Subjective Complains, Objective Complains)
- V Cardiovascular/Neurologic (hypotension/collapse/consciousness).

<u>**More specifically, the food challenge must be stopped if**</u>:

Only 1 out of 5 categories has increased its grading symptom score

**≥ 1-point rise**<br>**(i.e. mild, moderate or severe)**		**≥ 2-point rise**<br>**(i.e. moderate or severe)**		**3-point rise**<br>**(i.e. severe)**
o Laryngeal<br>o Wheezing<br>o Objective Gastrointestinal<br>o Cardiovascular/neurological	**OR**	o Urticaria<br>o Rash<br>o Laryngeal (short transient and not persistent discomfort)	**OR**	o Pruritus<br>o Sneezing /Itching<br>o Nasal Congestion<br>o Rhinorrhea<br>o Conjunctivitis

2 categories (or more) have increased their grading symptom scores

**≥ 1-point rise**<br>**(i.e. mild, moderate or severe)**		**≥ 2-point rise**<br>**(i.e. moderate or severe)**
o Urticaria<br>o Angioedema<br>o Rash<br>o Laryngeal<br>o Wheezing<br>o Objective Gastrointestinal<br>o Cardiovascular/neurological	**AND**	o Pruritus<br>o Sneezing/Itching<br>o Nasal Congestion<br>o Rhinorrhea<br>o Conjunctivitis

For emesis/vomiting, the symptoms may result from two different situations: vomiting/spitting-up during the feeding, resulting from subjective intolerance to the food formula or vomiting/emesis resulting from a gastrointestinal allergic reaction to the peanut allergen, usually delayed from the feeding. Only the second situation will be considered as an objective OFC symptom and will be considered for the stopping rules. In case of symptoms emergence not meeting the stopping rules, 30 additional minutes of observation should be considered to evaluate if the reactions are subsiding or progressing.

In case of doubt on the positivity of the response, the same dose could be repeated, but this situation should be avoided as much as possible, as the repetition of the same dose will increase the CRD without changing the level of the ED.

In case of subjective symptoms, for example mouth pruritus, throat pruritus, nausea, abdominal pains or any other subjective symptoms, the severity of which might signify that objective symptoms are developing according to the Investigator's judgment, the time between the previous dose and the next dose might be extended to see how the subject's symptoms evolve.

The dose that triggers objective symptoms requiring treatment as defined by the stopping rules will be considered as the ED.

As a safety precaution, the objective symptoms signaling the end of the food-challenge will be treated. The medication used will be left to the Investigator's judgment.

The Investigator and medical staff will use their own clinical judgment for the most effective treatment to give to the subject considering her/his age, the type of the allergic reactions and their severity. Also refer to recommendations made by Sampson et al. for treating anaphylaxis[13].

Should epinephrine need to be administered, it should be injected intramuscularly in the anterolateral thigh using auto-injectors commercially available in each specific site/country. Intravenous epinephrine should NOT be considered at the investigative sites to treat the reactions.

Subjects will be kept under observation for an additional 3 hours after the ingestion of the last dose of the challenge formula. Based on the Investigator's judgment, the observation period could be extended beyond the first 3 hours to ensure that all symptoms have subsided before the subject is discharged. For instance, an overnight stay may be considered necessary by the Investigator if the symptoms have not completely resolved within the 3 hours or if the symptoms have been severe or serious and require longer observation periods.

Complete information for all reactions will be reported first in source documents and then in the e-CRF, along with doses given, symptoms observed and their highest grades, time of appearance of the symptoms and an Investigator's assessment of the ED.

During the duration of the study, food-challenges to other foods cannot be conducted for any treated subject.

Oral Food Challenge Symptom Score Sheet

I. SKIN

	**Stopping rules: minimal # of grade increase**	
	**Alone**	**Associated**
**A. Pruritus**	**+3**	**+2**
0 = Absent		
1 = Mild: occasional scratching		
2 = Moderate: scratching continuously for >2 minutes at a time		
3 = Severe: hard continuous scratching excoriations		
**B. Urticaria/Angioedema**	**+2**	**+1**
0 = Absent		
1 = Mild: <3 hives, or mild lip edema		
2 = Moderate: <10 hives but >3, or significant lip or face edema		
3 = Severe: generalized involvement		
**C. Erythematous Rash**	**+2**	**+1**
0 = Absent		
1 = Mild: few areas of faint erythema		
2 = Moderate: areas of erythema (>20% and <50%), macular and raised rash		
3 = Severe: generalized marked erythema (>50%), extensive raised lesions (>25%)		
Erythematous Rash % area involved (see below):	/_____/____/%	

	5-year-old	10-year-old
Head	16%	12%
Neck	2%	2%
Anterior trunk	14%	14%
Posterior trunk	18%	18%
Leg (1)	15%	17%
Arm (1)	10%	10%

Adapted from Lund CC, Browder NC (1944) The estimation of areas of burns. Surg Gynecol Obstet 79: 352–8

II. UPPER RESPIRATORY/OCCULAR

	**Stopping rules: minimal # of grade increase**	
	**Alone**	**Associated**
**A. Sneezing/Itching**	**+3**	**+2**
0 = Absent		
1 = Mild: rare bursts		
2 = Moderate: bursts <10, intermittent rubbing of nose / eyes / external ear canals		

3 = Severe: continuous rubbing of nose / eyes, periocular swelling and/or long		
bursts of sneezing		
**B. Nasal Congestion**	**+3**	**+2**
0 = Absent		
1 = Mild: some hindrance to breathing		
2 = Moderate: nostrils feel blocked, breathing through mouth most of time		
3 = Severe: nostrils occluded		
**C. Rhinorrhea**	**+3**	**+2**
0 = Absent		
1 = Mild: occasional sniffling		
2 = Moderate: frequent sniffling, requires tissues		
3 = Severe: nose runs freely despite sniffling and tissues		
**D. Laryngeal**	**+1**	**+1**
0 = Absent		
1 = Mild: throat clearing, occasional cough		
2 = Moderate: hoarseness, frequent dry cough		
3 = Severe: inspiratory stridor		
**E. Conjunctivitis** (see example below)	**+3**	**+2**
0 = Absent		
1 = Mild:		
2 = Moderate:		
3 = Severe:		

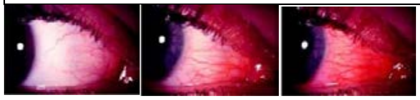

Grade 1 Grade 2 Grade 3

III. LOWER RESPIRATORY

	Stopping rules: minimal # of grade increase	
	**Alone**	**Associated**
**A. Wheezing**	**+1**	**+1**
0 = Absent		
1 = Mild: expiratory wheezing to auscultation		
2 = Moderate: dyspnea, inspiratory and expiratory wheezing		
3 = Severe: dyspnea, use of accessory muscles, audible wheezing		

IV. GASTROINTESTINAL

	Stopping rules: minimal # of grade increase	
	**Alone**	**Associated**
**A. Subjective Complaints**	**NA**	**NA**
0 = Absent		
1 = Mild: itchy mouth/throat, c/o nausea, abdominal pain, no change in activity		
2 = Moderate: frequent c/o nausea or abdominal pain, decreased activity		
3 = Severe: patient in bed; crying, notably distressed		
**B. Objective Complaints**	**+1**	**+1**
0 = Absent		
1 = Mild: 1 episode of emesis* or diarrhea		
2 = Moderate: 2-3 episodes of emesis* or diarrhea or 1 of each		
3 = Severe: >3 episodes of emesis* or diarrhea or 2 of each		

* Emesis to be differentiated from "spitting-up" due to food intolerance while feeding.

V. CARDIOVASCULAR/NEUROLOGIC

	Stopping rules: minimal # of grade increase	
	**Alone**	**Associated**
**Cardiovascular/neurologic**	**+1**	**+1**
0 = Normal: heart rate or BP for age/baseline		
1 = Mild: color change, subjective response (weak, dizzy), or tachycardia, mental status change, mild hypotension (weak rapid pulse and/or 10-20% drop in blood pressure from baseline)		
2 = Moderate: drop in blood pressure >20% from baseline, significant change in mental status, light-headedness, feeling of "pending doom"		
3 = Severe: cardiovascular collapse, signs of impaired circulation, unconsciousness, bradycardia, cardiac arrest.		

Reference: Sampson HA, Muñoz-Furlong A, Campbell RL, Adkinson NF Jr, Bock SA, Branum A, et al. Second symposium on the definition and management of anaphylaxis: summary report—Second National Institute of Allergy and Infectious Disease/Food Allergy and Anaphylaxis Network symposium. J Allergy Clin Immunol. 2006;117(2):391-7.

APPENDIX 7

FAQLQ/FAIM Questionnaires

English – Child version

FAQLQ-CF

Food Allergy Quality of Life Questionnaire – Child Form (8-12 years)

EuroPrevall

To cite this questionnaire:
Flokstra-de Blok BMJ, DunnGalvin A, Vlieg-Boerstra BJ, Oude Elberink JNG, Duiverman EJ, Hourihane JO, Dubois AEJ. Development and validation of a self-administered Food Allergy Quality of Life Questionnaire for children. Clin Exp Allergy 2009 Jan;39(1):127-137.

The questions are about the influence of your food allergy on your quality of life. It is important that you fill in the answers yourself. You may ask your parents for help, but they are not allowed to tell you which answer to give. Answer every question by putting an 'x' in the proper box. You may choose from the following answers.

not	barely	a little bit	fairly	quite	very	extremely

	**How *troublesome* do you find it, because of your food allergy, that you ...**							
1	must always watch what you eat?	☐	☐	☐	☐	☐	☐	☐
2	can eat fewer things?	☐	☐	☐	☐	☐	☐	☐
3	are limited in buying things you like?	☐	☐	☐	☐	☐	☐	☐
4	have to read labels?	☐	☐	☐	☐	☐	☐	☐
5	have to refuse food when you do things with others?	☐	☐	☐	☐	☐	☐	☐
6	can less easily stay for a meal with someone?	☐	☐	☐	☐	☐	☐	☐
7	can taste or try fewer things when eating out?	☐	☐	☐	☐	☐	☐	☐
8	have to tell beforehand about what you are not allowed to eat when eating out?	☐	☐	☐	☐	☐	☐	☐
9	have to check yourself whether you can eat something when eating out?	☐	☐	☐	☐	☐	☐	☐
10	hesitate eating certain foods when you don't know if it is safe?	☐	☐	☐	☐	☐	☐	☐
11	must watch out when touching certain foods?	☐	☐	☐	☐	☐	☐	☐
12	don't get anything when someone is giving treats at school?	☐	☐	☐	☐	☐	☐	☐

not	barely	a little bit	fairly	quite	very	extremely

	**How *troublesome* is it, because of your food allergy, ...**							
13	that the ingredients of a food change?	☐	☐	☐	☐	☐	☐	☐
14	that the label states: "May contain (traces of)....."?	☐	☐	☐	☐	☐	☐	☐
15	that you have to explain to people around you that you have a food allergy?	☐	☐	☐	☐	☐	☐	☐
16	that people around you forget that you have a food allergy?	☐	☐	☐	☐	☐	☐	☐
17	that others can eat the food you are allergic to when you do things with other people?	☐	☐	☐	☐	☐	☐	☐
18	that you don't know how things taste which you can't eat?	☐	☐	☐	☐	☐	☐	☐

	**How *frightened* are you because of your food allergy ...**							
19	of an allergic reaction?	☐	☐	☐	☐	☐	☐	☐
20	of eating the wrong food by accident?	☐	☐	☐	☐	☐	☐	☐
21	to eat something you have never eaten before?	☐	☐	☐	☐	☐	☐	☐

	**Answer the following questions:**							
22	How *concerned* are you that you will never get rid of your food allergy?	☐	☐	☐	☐	☐	☐	☐
23	How *disappointed* are you when people don't take your food allergy into account?	☐	☐	☐	☐	☐	☐	☐
24	How *disappointed* do you feel because you have a food allergy?	☐	☐	☐	☐	☐	☐	☐

The following four questions are about the chance that you think you have of something happening to you because of your food allergy. Choose one of the answers. This is followed by two more questions about your food allergy. Answer every question by putting an 'x' in the box next to the proper answer.

0	1	2	3	4	5	6
never (0% chance)	very small chance	small chance	fair chance	big chance	very big chance	always (100% chance)

	How big do you think the chance is that you ...	0	1	2	3	4	5	6
1	will accidentally eat something to which you are allergic?	☐	☐	☐	☐	☐	☐	☐
2	will have a severe reaction if you accidentally eat something to which you are allergic?	☐	☐	☐	☐	☐	☐	☐
3	will die if you accidentally eat something to which you are allergic?	☐	☐	☐	☐	☐	☐	☐
4	can <u>not</u> do the right things for your allergic reaction should you accidentally eat something to which you are allergic?	☐	☐	☐	☐	☐	☐	☐

**5. How many foods are you unable to eat because of your food allergy?**	**6.** Everyone does things with other people, such as;<br>- playing with friends,<br>- going to a birthday party,<br>- visiting,<br>- staying over with someone for a meal or eating out.<br>**How much does your food allergy affect things you do with others?**
☐ almost none	☐ so little I don't actually notice it
☐ very few	☐ very little
☐ a few	☐ little
☐ some	☐ moderately
☐ many	☐ a good deal
☐ very many	☐ a great deal
☐ almost all	☐ a very great deal

English – Parent version

FAQLQ-PF

Food Allergy Quality of Life Questionnaire – Parent Form (0-12 years)

To cite this questionnaire:

DunnGalvin A, Flokstra-de Blok BMJ, Burks AW, Dubois AEJ, Hourihane JO. Food allergy QoL questionnaire for children aged 0-12 years: content, construct, and cross-cultural validity. Clin Exp Allergy 2008 Jun;38(6):977-986.

Food Allergy Quality of Life Questionnaire-Parent Form (FAQoL-PF)

Children aged 0-12 years

Instructions to Parents

- The following are scenarios that parents have told us affect children's quality of life because of food allergy.
- Please indicate how much of an impact each scenario has on **your child's quality of life** by placing a tick or an x in one of the boxes numbered 0-6.

Response Options

0 = not at all
1= a little bit
2 = slightly
3 = moderately
4 = quite a bit
5 = very much
6 = extremely

All information given is completely confidential.
This questionnaire will only be identified by a code number.

- If your child is aged **0 to 3 years**, please answer **Section A.**
- If your child is aged **4 to 6 years**, please answer **Section A & Section B.**
- If your child is aged **7 years and over**, please answer **Section A, Section B & Section C.**

SECTION A

Not at all → Extremely

	**Because of food allergy, my child feels...............**	0	1	2	3	4	5	6
1	Anxious about food	☐	☐	☐	☐	☐	☐	☐
2	Different from other children	☐	☐	☐	☐	☐	☐	☐
3	Frustrated by dietary restrictions	☐	☐	☐	☐	☐	☐	☐
4	Afraid to try unfamiliar foods	☐	☐	☐	☐	☐	☐	☐
5	Concerned that I am worried that he/she will have a reaction to food	☐	☐	☐	☐	☐	☐	☐

Not at all → Extremely

	**Because of food allergy, my child...............**	0	1	2	3	4	5	6
6	Experiences physical distress	☐	☐	☐	☐	☐	☐	☐
7	Experiences emotional distress	☐	☐	☐	☐	☐	☐	☐
8	Has a lack of variety in his her diet	☐	☐	☐	☐	☐	☐	☐

Not at all → Extremely

	**Because of food allergy, my child has been negatively affected by..........**	0	1	2	3	4	5	6
9	Receiving more attention more attention than other children of his/her age	☐	☐	☐	☐	☐	☐	☐
10	Having to grow up more quickly than other children of his/her age	☐	☐	☐	☐	☐	☐	☐
11	His/her environment being more restricted than other children of his/her age	☐	☐	☐	☐	☐	☐	☐

Not at all → Extremely

	**Because of food allergy, my child's social environment is restricted because of limitations on........**	0	1	2	3	4	5	6
12	Restaurants we can safely go to as a family	☐	☐	☐	☐	☐	☐	☐
13	Holiday destinations we can safely go to as a family	☐	☐	☐	☐	☐	☐	☐

Not at all → Extremely

	**Because of food allergy, my child's ability to take part has been limited........**	0	1	2	3	4	5	6
14	In social activities in other people's houses (*sleepovers, parties, playtime*)	☐	☐	☐	☐	☐	☐	☐

- If your child is aged **0 to 3 years**, please now go to **Section D.**
- If your child is aged **4 to 12 years**, please now answer **Section B.**

SECTION B

Not at all → Extremely

	Because of food allergy, my child's ability to take part has been limited……..	0	1	2	3	4	5	6
15	In preschool/school events involving food (*class parties/treats/lunchtime*)	☐	☐	☐	☐	☐	☐	☐

Not at all → Extremely

	Because of food allergy, my child feels……………	0	1	2	3	4	5	6
16	Anxious when going to new places	☐	☐	☐	☐	☐	☐	☐
17	Concerned that he/she must always be cautious about food	☐	☐	☐	☐	☐	☐	☐
18	'Left out' in activities involving food	☐	☐	☐	☐	☐	☐	☐
19	Upset that family social outings (*eating out, celebrations, days out*) have been limited by food allergy	☐	☐	☐	☐	☐	☐	☐
20	Anxious about accidentally eating an ingredient to which he/she is allergic	☐	☐	☐	☐	☐	☐	☐
21	Anxious when eating with unfamiliar adults/children	☐	☐	☐	☐	☐	☐	☐
22	Frustrated by social restrictions	☐	☐	☐	☐	☐	☐	☐

Not at all → Extremely

	Because of food allergy, my child……………	0	1	2	3	4	5	6
23	Is more anxious in general than other children of his/her age	☐	☐	☐	☐	☐	☐	☐
24	Is more cautious in general than other children of his/her age	☐	☐	☐	☐	☐	☐	☐
25	Is not as confident as other children of his/her age in social situations	☐	☐	☐	☐	☐	☐	☐
26	Wishes his/her food allergy would go away	☐	☐	☐	☐	☐	☐	☐

- If your child is aged **6 years and under**, please now go to **Section D.**
- If your child is aged **7 years and older**, please answer **Section C.**

SECTION C

Not at all → Extremely

	Because of food allergy, my child feels……………	0	1	2	3	4	5	6
27	Worried about his/her future(opportunities, relationships)	☐	☐	☐	☐	☐	☐	☐
28	That many people do not understand the serious nature of food allergy	☐	☐	☐	☐	☐	☐	☐
29	Concerned by poor labelling on food products	☐	☐	☐	☐	☐	☐	☐
30	That food allergy limits his/her life in general	☐	☐	☐	☐	☐	☐	☐

SECTION D.

Please answer the following questions with reference to the 6-point scale on the right

0 = extremely unlikely
1 = very unlikely
2 = somewhat unlikely
3 = likely
4 = quite likely
5 = very likely
6 = extremely likely

Q1. What chance **do you think** your child has of ….?

	Question	6-point Scale						
		0	1	2	3	4	5	6
1	……accidentally ingesting the food to which they are allergic ?							
2	……having a severe reaction if food is accidentally ingested ?							
3	……dying from his/her food allergy following ingestion in the future ?							
4	……effectively treating him/herself, or receiving effective treatment from others (including Epipen administration), if he/she accidentally ingests a food to which he/she is allergic ?							

Q2. What chance **does your child think** he/she has of ……?

	Question	6-point Scale						
		0	1	2	3	4	5	6
1	……accidentally ingesting the food to which they are allergic ?							
2	……having a severe reaction if food is accidentally ingested ?							
3	……dying from his/her food allergy following ingestion in the future ?							
4	……effectively treating him/herself, or receiving effective treatment from others (including Epipen administration), if he/she accidentally ingests a food to which he/she is allergic ?							

APPENDIX 8

SCORAD

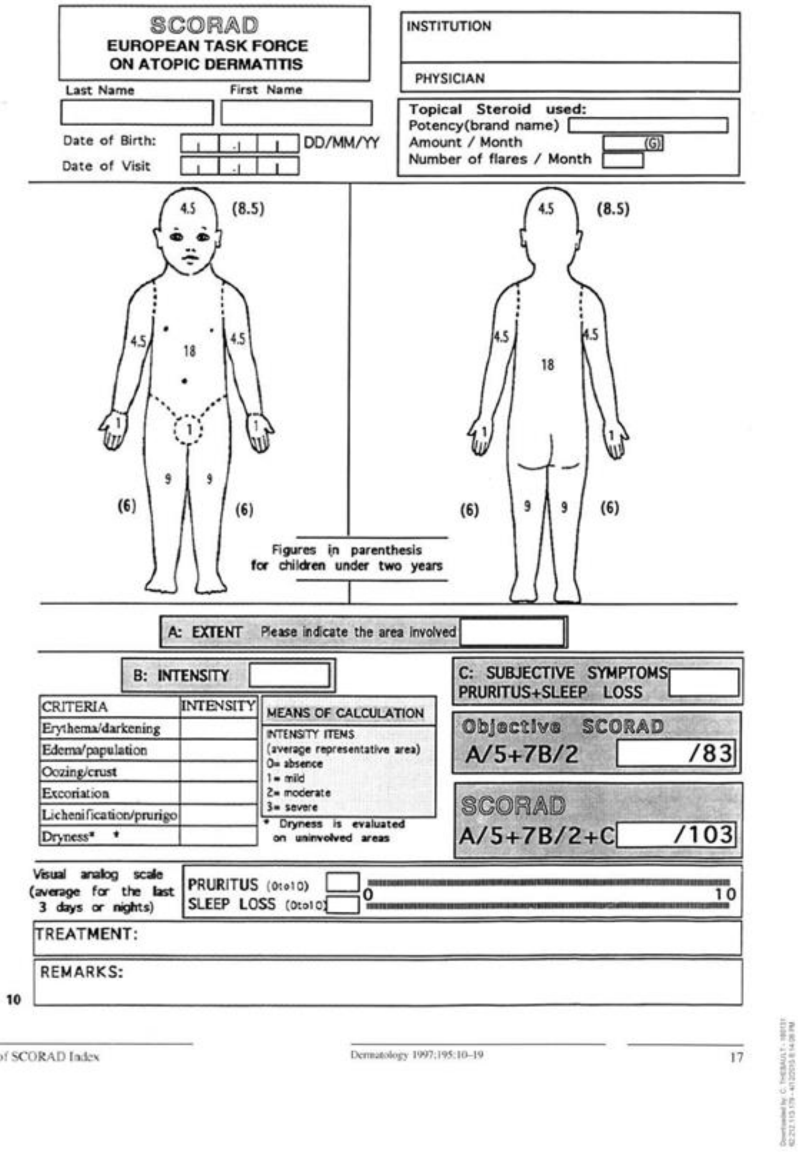

SCORAD
EUROPEAN TASK FORCE
ON ATOPIC DERMATITIS

Last Name First Name

Date of Birth: DD/MM/YY

Date of Visit

INSTITUTION

PHYSICIAN

Topical Steroid used:
Potency(brand name)
Amount / Month (G)
Number of flares / Month

Figures in parenthesis for children under two years

A: EXTENT Please indicate the area involved

B: INTENSITY

CRITERIA	INTENSITY
Erythema/darkening	
Edema/papulation	
Oozing/crust	
Excoriation	
Lichenification/prurigo	
Dryness*	

MEANS OF CALCULATION
INTENSITY ITEMS
(average representative area)
0= absence
1= mild
2= moderate
3= severe

* Dryness is evaluated on uninvolved areas

C: SUBJECTIVE SYMPTOMS
PRURITUS+SLEEP LOSS

Objective SCORAD
A/5+7B/2 /83

SCORAD
A/5+7B/2+C /103

Visual analog scale
(average for the last 3 days or nights)

PRURITUS (0 to 10) 0 10
SLEEP LOSS (0 to 10)

TREATMENT:

REMARKS:

10

Validation of SCORAD Index

Dermatology 1997;195:10–19

17

Source: Kunz B, Oranje AP, Labrèze L, Stalder JF, Ring J, Taïeb A. Clinical validation and guidelines for the SCORAD index: consensus report of the European Task Force on Atopic Dermatitis. Dermatology. 1997;195(1):10-9.
