# Supplementary material for: Efficacy and safety of peanut epicutaneous immunotherapy in patients with atopic comorbidities
Source: J Allergy Clin Immunol Glob. 2022 Sep 22;2(1):69–75. doi: 10.1016/j.jacig.2022.07.009 (PMC10509968; doi:10.1016/j.jacig.2022.07.009)
Supplement: Supplement 2b [file mmc4.pdf]

PAREXEL International

DBV TECHNOLOGIES S.A.

LONG-TERM ASSESSMENT of SAFETY and THERAPEUTIC BENEFIT of VIASKIN® PEANUT EPICUTANEOUS TREATMENT in PEANUT-ALLERGIC CHILDREN: A 6-MONTH RANDOMIZED, DOUBLE-BLIND, PLACEBO-CONTROLLED PHASE III STUDY FOLLOWED BY AN OPEN LABEL ACTIVE TREATMENT (REALISE STUDY) V712-302

Statistical Analysis Plan V2.0 – 12 October 2017

PAREXEL Project Number: 230235

SIGNATURE PAGE

Approved by:

[redacted]

PAREXEL SIGNATURE PAGE

Signatures below confirm that the review process has been completed in accordance with SOP-GDO-WW-019.

This document has been approved and signed electronically on the final page by the following:

	**Signatory**
**Author**	[redacted]
	**Signatory**
**Reviewer**	[redacted]
	[redacted]
	[redacted]
	[redacted]

TABLE OF CONTENTS

1. INTRODUCTION ... 9
2. STUDY OBJECTIVES ... 9
3. INVESTIGATIONAL PLAN ... 9
3.1 Overall Study Design and Plan ... 9
3.3 Efficacy and Safety Variables ... 17
4. STATISTICAL METHODS ... 19
4.1 Data Quality Assurance ... 19
4.2 General Considerations ... 19
4.2.1 Statistical methods ... 19
4.2.2 Type of analyses ... 22
4.2.3 Definition of Baseline ... 23
4.2.4 Treatment groups ... 23
4.2.5 Study assessments ... 23
4.2.6 Missing Data Conventions ... 25
4.3 Study Subjects ... 25
4.3.1 Disposition of Subjects ... 25
4.3.2 Protocol Deviations ... 26
4.4 Analysis Populations ... 26
4.4.1 Screened population ... 27
4.4.2 Randomized population ... 27
4.4.3 Safety Populations & Safety Sub-Populations ... 27
4.4.4 Per-protocol Population & sub-populations ... 28
4.4.5 Interim and Final Analyses ... 29
4.4.6 Data and Safety Monitoring Board ... 29
4.4.7 Examination of Subgroups ... 30
4.5 Demographics and Baseline characteristics ... 30
4.5.1 Demographic variables ... 30
4.5.2 Baseline Characteristics ... 31
4.5.3 Medical history ... 31
4.5.4 Parental atopic medical history ... 32
4.5.5 Disease history ... 32
4.6 Previous and concomitant medications ... 33
4.7 Study duration and study treatment characteristics ... 35
4.7.1 Study duration ... 35
4.7.2 Treatment exposure ... 36
4.7.3 Total dose of peanut protein ... 38
4.7.4 Compliance ... 38
4.8 Study Drug Safety Evaluation ... 39
4.8.1 Adverse Events ... 39

4.8.2 Adverse event of special interest (AESI) 44
4.8.3 Subject Diaries (Local skin reactions) 45
4.8.4 Skin reactions (as graded by the investigator) 46
4.8.5 Deaths 46
4.8.6 Clinical Laboratory Evaluation 46
4.8.7 Vital Signs 48
4.8.8 Physical Examination 49
4.8.9 Spirometry and Peak Expiratory Flow Results 49
4.9 Exploratory Analyses 51
4.9.1 Immunological markers 51
4.9.2 Skin Prick Test 51
4.9.3 Food Allergy Quality of Life Questionnaire /Food Allergy Independent Measure 52
4.9.4 Accidental Consumption of Peanuts 54
4.9.5 Epigenetic analyses 54
4.9.6 Genetic Screening 55
4.9.7 SCORAD (Scoring Atopic Dermatitis) 55
4.9.8 Peanut-Food Challenge 55
4.10 Determination of Sample Size 57
4.11 Changes in the Conduct of the Study or Analysis planned in the protocol 57
5. REFERENCES 58
6. APPENDICES 59
6.1 Appendix 1: Staging System of severity of Anaphylaxis 59
6.2 Appendix 2: Major Protocol deviations 60
6.3 Appendix 3: Common Terminology Criteria for Adverse Events (CTCAE) grades version 4.03 61
6.4 Appendix 4: List of Post-Text Tables, figures, listings, and Supportive SAS output appendices 61
6.5 Appendix 5: Methodology of identification of systemic allergic adverse events 62
6.6 Appendix 6: Definition of filaggrin null mutation group 62

LIST OF ABBREVIATIONS

AE / AESI	Adverse event / Adverse event of special interest
ATC	Anatomical Therapeutic Chemical (Classification System)
ATP	Active Treatment Period
BSA	Body Surface Area
CI	Confidence interval
CTCAE	Common Terminology Criteria for Adverse Events
CTMS	Clinical trials Management System
D	Day
DA	Day of Active Treatment
DBP	Double-Blind Period
DSMB	Data and Safety Monitoring Board
eCRF	Electronic case report form
ED	Eliciting Dose
EDC	Electronic data capture
EoS	End of Study
EPIT	EPicutaneous ImmunoTherapy
ET	Early Termination
FAQLQ	Food Allergy Quality of Life Questionnaire
FAIM	Food Allergy Independent Measure
FAS	Full analysis set
FEV_1	Forced expiratory volume in one second
ICF	Informed consent form
IgE, IgG, IgG4	Immunoglobulin E, immunoglobulin G, immunoglobulin G4 subtype
IQR	Interquartile range
IP	Investigational product
ITT	Intent-to-treat
IWRS	Interactive Web Response System
M	Month
MA	Month of Active Treatment

MedDRA	Medical Dictionary for Regulatory Activities
PC	Phone Contact
PEF	Peak Expiratory Flow
PFC	Peanut-Food Challenge
PP	Per-protocol
PT	Preferred term
PDV	Protocol deviation
Q1, Q3	First, third quartile
SAE	Serious adverse event
SAP	Statistical analysis plan
SCORAD	Scoring atopic dermatitis
SE	Standard Error
SOC	System organ class
SPwoSA	Safety Sub-population without history of Severe Anaphylaxis
SPwSA	Safety Sub-population with history of Severe Anaphylaxis
SPT	Skin prick test
TEAE	Treatment-emergent adverse event
TFL	Tables, Figures and listings
US(A)	United States (of America)
UV	Unscheduled visit
V	Visit
WHO	World Health Organization
VP 250	Viaskin® Peanut 250 µg

Figures and tables

Figure 1: Study Design 12

Table 1: Schedule of Procedures : First 6 months Blinded Period (Double-Blind Treatment period)..13
Table 2: Schedule of Procedures : Month 6 to Month 42 Open Label treatment Period (Open-label Active Treatment period)..15

1. INTRODUCTION

This Statistical Analysis Plan (SAP) describes the statistical methods to be used for the reporting and analyses of data collected under the DBV protocol REALISE[1].

This SAP is based upon the following study documents:

- Protocol, Version 3.0 (January 5, 2017)
- Electronic Case Report Form (eCRF), Version 2.0 (April 27, 2017)
- Data Transfer agreement, Version 1.0 (January 18, 2017)

2. STUDY OBJECTIVES

The objectives of this study are to assess the safety of Viaskin® Peanut in peanut-allergic subjects 4 through 11 years of age over a 36-month treatment period, to explore the treatment benefit over a 36-month treatment period and to gain experience about the use of Viaskin® Peanut 250 µg in the usual conditions of medical practices (real life).

3. INVESTIGATIONAL PLAN

3.1 Overall Study Design and Plan

This is a randomized 36-month, Phase III, safety study to assess the long-term safety of Viaskin® Peanut, dosed at 250 µg peanut protein (per patch) in peanut-allergic children from 4 through 11 years of age.

The study design is in 2 main parts:

- A randomized, double-blind, placebo-controlled design for the first 6 months of treatment,
- Followed by an open-label, single arm active treatment design with Viaskin® Peanut 250 µg.

After selection, the subjects will be randomized with a ratio of 3:1, in either the active arm or placebo arm. The randomization was planned to be stratified by center and age group (children 4 and 5 years of age in a stratum and children 6-11 years in a second stratum). The randomization was finally only stratified by age group.

[1] REALISE: **REAL LI**fe Use and **S**afety of **E**PIT –
LONG-TERM ASSESSMENT of SAFETY and THERAPEUTIC BENEFIT of VIASKIN® PEANUT EPICUTANEOUS TREATMENT in PEANUT-ALLERGIC CHILDREN: A 6-MONTH RANDOMIZED, DOUBLE-BLIND, PLACEBO-CONTROLLED PHASE III STUDY FOLLOWED by AN OPEN LABEL ACTIVE TREATMENT

The duration of the open label period will depend on the treatment received during the first 6 months:

- Subjects initially randomized in the active Viaskin® Peanut 250 µg group will continue their active treatment for 30 months.
- Subjects initially randomized in the placebo group will cross-over to receive the active treatment Viaskin® Peanut 250 µg for 36 months.

The randomization code will be unblinded and the treatment groups assigned during the first 6 months will only be communicated to the investigators and the subjects after the 6-month database lock (i.e.: when all patients achieved 6 months of treatment). To ensure that the placebo subjects switching to receive the active treatment will start their treatment safely, all subjects will repeat the progressive increase of daily duration of the Viaskin® Peanut 250 µg patches application, as at the start of the study.

The overall maximum total study duration for each subject ranges from 3 to 3.5 years:

- A 2-week screening period,
- Subjects initially in the active arm: 36 months of treatment,
- Subjects initially in the placebo arm: 42 months of treatment (6 months under placebo + 36 months of active treatment),
- A 2-week follow-up period after the end of the treatment.

The subjects selected for the study must have a physician-diagnosis of peanut allergy based on the presence of a well-documented medical history of IgE-mediated reactions after ingestion of peanut i.e. allergic reaction(s) having led to an emergency department visit or a physician consultation and currently following a strict peanut-free diet.

Once selected, the subject will perform several procedures and must at least fulfill the two following criteria to be eligible for treatment:

- A peanut Skin Prick Test (SPT) with a largest wheal diameter ≥8 mm and
- A peanut-specific IgE ≥14 kU/L.

Subjects will apply daily on an intact skin a Viaskin® patch containing 250 µg peanut protein for a period of 36 months.

Key assessments of safety will be performed at each study visit by the Investigators, including:

- Skin observation of the patch areas of application (inter-scapular area of the back),
- Spirometry (for subjects ≥6 years of age),
- Peak expiratory flow (PEF) measurements (for subjects ≥5 years of age),
- Vital signs,
- Physical examinations,
- Laboratory assessments,
- Atopic dermatitis using the SCORAD (Scoring atopic dermatitis).

Laboratory parameters will include:

- Peanut-specific immunoglobulin E (sIgE),
- Peanut component-sIgE,
- Peanut-specific immunoglobulin G4 subtype (sIgG4),

- Peanut component-sIgG4.

Skin prick tests (SPTs) will also be performed.

During the whole study duration, all concomitant medications will be reported in the eCRF. Any adverse event (AE) will be spontaneously reported in the dedicated section in the diaries and/or in the eCRF as follows:

During the first 6 months:

- Three pre-specified solicited symptoms (itching, redness, swelling) will not be reported in the AE pages of the e-CRF, except:
  - if these symptoms are part of another concomitant disease;
  - if these symptoms are leading to the subject's study discontinuation;
  - if these are serious AEs.
- Any other local skin reactions or any other type of AEs will be spontaneously reported in the diaries and will be reported by the investigators in the AE e-CRF form.

After month 6:

- Any AE, including the 3 above symptoms, other local skin reactions or any other type of AE will be spontaneously reported in the dedicated section of the diaries. These AEs will be reported by the investigators in the AE e-CRF form.

The diaries will be systematically reviewed by the site medical staff at each subject's visit.

Considering the length of this study and for ensuring the subject's medical management, the investigators may need to perform peanut-food challenge during the course of the study.

A maximum of 2 open peanut-containing food challenges for the same subject is recommended during the subject treatment course up to the 36 months of active treatment. Performing open food challenge(s) in the REALISE study is optional and is left to the investigator's decision. However, no food challenge can occur before the subject has received 12 months of active treatment with Viaskin® Peanut 250 µg. If 2 peanut challenges are performed for the same subject within the treatment course, the second challenge can occur only after at least 12 additional months of active treatment have been administered to that subject.

If the decision is made by the investigators to perform an open peanut-containing food challenge, its performance must comply with the standardized methodology defined in the protocol APPENDIX 6. A standardized peanut-food challenge formula and material as well as the Manual of Procedures will be provided to all sites, and must be used for conducting the open peanut-food challenge.

All food challenges results will be reported in the e-CRF.

Detailed information on study assessments and procedures are provided below.

All subjects will remain on a peanut-free diet for the duration of the study. The re-introduction or not of peanut into the subject's diet at the end of their participation in the study will be left to the Investigator's decision. The Investigator's decision will be collected in the eCRF.

3.2 Study Schematic Diagram

The study design is presented below in Figure 1.

Figure 1: Study Design

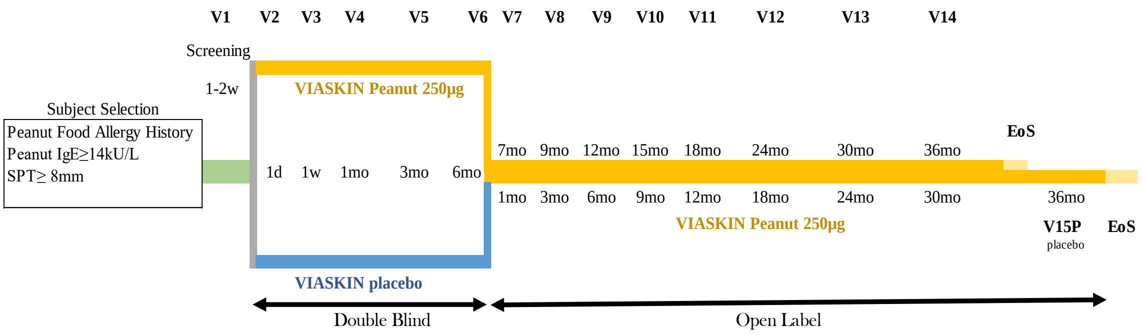

Abbreviations: EoS = End of study ; IgE = Immunoglobulin E; SPT = Skin Prick Test.

The schedule of procedures is presented in table 1.

Table 1: Schedule of Procedures: First 6 months Blinded Period (Double-Blind Treatment period)

**Study Assessments (Double-Blind Treatment period – V1 to V6)**	**V1 screening**	**V2 D1**	**V3 D8**	**V4 M1**	**V5 M3**	**V6 M6**	**☎ #**
Informed consent	X						
Medical history/Peanut allergy or positive PFC documentation[1] / Family atopy history	X						
Check eligibility (inclusion/exclusion criteria)	X	X					
Demographics	X						
Physical examination [2]	X	X	X	X	X	X	
Vital signs [3]	X	X	X	X	X	X	
Spirometry (FEV_1) [4]	X					X	
PEF [5]	X	X	X	X	X	X	
SCORAD	X				X	X	
FAQLQ/FAIM [6]	X					X	
SPT (Skin Prick Test)	X				X	X	
Immunological markers [7]	X				X	X	
Laboratory tests [8]	X				X	X	
Filaggrin gene [9] *(Optional)*					X	X	
Urine pregnancy test	X					X	
Epigenetic analyses	X				X	X	
Treatment Initiation		X					
Adverse events		X	X	X	X	X	X
Concomitant medications	X	X	X	X	X	X	X
Check for any accidental peanut consumption			X	X	X	X	X
Subject diary (dispense/check)		X	X	X	X	X	X
Dispense subject safety leaflet and subject identification card		X					
Apply Viaskin® patch at site		X				X	
Time under observation before discharge		3 hrs				X	
Check skin reactions under the patch and grading [10]		X	X	X	X	X	
Dispense epinephrine auto-injector and anaphylaxis emergency action plan / 1% Hydrocortisone ointment		X					
Review Epinephrine auto-injector use and subject safety precaution information leaflet, including anaphylaxis emergency action plan			X	X	X	X	
Assessment of used/unused study drug dispensed to the subject and of medication compliance			X	X	X	X	
Dispense study drug to the subject (Viaskin® Peanut 250 µg or Viaskin® placebo) [11]		X	X	X	X	X	

Abbreviations:

D = Day; d = days; ET = Early termination; FAQLQ/FAIM = Food Allergy Quality of Life Questionnaire/Food Allergy Independent Measure; FEV_1 = Forced expiratory volume in one second; hrs = hours; M = Month; PC = Phone contact; PEF = Peak expiratory flow; SCORAD = Scoring atopic dermatitis; SPT = Skin prick test; UV = Unscheduled Visit; V = Visit;

1. Including history of peanut allergy and peanut FC previously performed, including date, procedures, doses used, challenge material and content in peanut protein, Eliciting Dose and symptoms.
2. Including a systematic complete skin examination, other physical examination as required,
3. Blood pressure, heart rate and respiratory rate.
4. FEV1 will be measured for subjects ≥6 years of age (unless they have documented inability to adequately perform spirometry).
5. PEF will be measured for all the subjects ≥5 years of age.
6. For both FAQLQ and FAIM, subjects ≥8 years of age will use the Child Form of the FAQLQ/FAIM. All parents/guardians will use the Parental Form. The FAQLQ and FAIM forms will be completed.
7. Peanut-specific IgE, peanut-specific IgG4, peanut-specific-component IgE and peanut-specific-component IgG4 to Ara h 1, Ara h 2, Ara h 3, Ara h 8 and Ara h 9. IgE specific to cow's milk, to egg white, to house dust mites, and to grass pollen will be tested at screening, month 3, 6, 9, 12, 18, 24, 30, 36 for all subjects and Month 42 for subjects initially in the placebo group.
8. Laboratory tests performed centrally. Hematology: hemoglobin, hematocrit, platelets, red blood cells, white blood cells with differential cell count. Biochemistry: aspartate aminotransferase, alanine aminotransferase, total bilirubin, blood urea nitrogen, creatinine, total protein.
9. Signing the consent for the filaggrin genetic analysis can be done any time after the subject is included in the study. However, collection of blood is done only once at Visit 5 or 6.
10. Check the reactions of the skin on the back of the subject and grade the severity of the local skin reactions. At Visit 2, grading is to be done before patch application and at 30 min, 1 h, 2 h and 3 h after patch application.
11. At Visit 5, the study drug (Viaskin® Peanut 250µg or Viaskin® placebo) is dispensed for a duration of 3 months.

☎ Phone Contacts at D4 D22 M2 M4.5

Time windows:

Visit	Time windows
V2	within 2 weeks of V1
PC (D4), PC (D22)	+/- 2 days
V3 (D8), V4 (M1), PC (M2)	+/- 3 days
V5 (M3), PC (M4.5)	+/- 7 days

Table 2: Schedule of Procedures: Month 6 to Month 42 Open Label treatment Period

**Study assessments**	**V6**	**V7**	**V8**	**V9**	**V10**	**V11**	**V12**	**V13**	**V14**	**V15P**	**EoSV**	**ET**	**UV[12]**	**☎**
	**M6**	**M7**	**M9**	**M12**	**M15**	**M18**	**M24**	**M30**	**M36**	**M42**				**#**
**Active Arm Active treatment duration**	M6	M7	M9	M12	M15	M18	M24	M30	M36	-				
**Placebo Arm Active treatment duration**	D1	M1	M3	M6	M9	M12	M18	M24	M30	M36				
Physical examination [1]	X	X	X	X	X	X	X	X	X	XP	X	X	X	
Vital signs [2]	X	X	X	X	X	X	X	X	X	XP	X	X	X	
Spirometry (FEV_1) [3]	X			X		X	X	X	X	XP		X	X	
PEF [4]	X	X	X	X		X	X	X	X	XP		X	X	
SCORAD	X		X	X		X	X	X	X	XP		X		
FAQLQ/FAIM [5]	X			X		X	X	X	X	XP		X		
SPT (Skin Prick Test)	X		X	X		X	X	X	X	XP		X		
Immunological markers [6]	X		X	X		X	X	X	X	XP		X		
Laboratory tests [7]	X		X	X		X	X	X	X	XP		X	X	
Filaggrin gene [8] *(Optional)*	X													
Urine pregnancy test *(optional at other visits)*	X			X			X		X	XP				
Epigenetic analyses	X		X	X		X	X	X	X	XP				
Adverse events	X	X	X	X	X	X	X	X	X	XP	X	X	X	X
Concomitant medications	X	X	X	X	X	X	X	X	X	XP	X	X	X	X
Check for any accidental peanut consumption	X	X	X	X	X	X	X	X	X	XP	X	X	X	X
Subject diary (dispense/check)	X	X	X	X	X	X	X	X	X	XP	X	X	X	X
Apply Viaskin® patch at site	X													
Time under observation before discharge	3 hrs													
Check skin reactions under the patch and grading [9]	X	X	X	X	X	X	X	X	X	XP		X	X	
Review Epinephrine auto-injector use and subject safety precaution information leaflet, including anaphylaxis emergency action plan	X	X	X	X	X	X	X	X	X	XP		X	X	
Assessment used/unused study drug dispensed to the subject	X	X	X	X	X	X	X	X	X	XP		X	X	
Peanut-Food Challenge (optional) [10]				X[11]		X	X	X	X	XP				
Dispense study drug to the subject (Viaskin® Peanut 250 µg)	X	X	X	X	X	X	X	X	XP					

Abbreviations:
D = Day; EoSV= End of Study Visit; ET = Early termination; FAQLQ/FAIM = Food Allergy Quality of Life Questionnaire/Food Allergy Independent Measure; FEV_1 = Forced expiratory volume in one second; hrs = hours; M = Month; PC = Phone contact; PEF = Peak expiratory flow; SCORAD = Scoring atopic dermatitis; SPT = Skin prick test; UV = Unscheduled Visit; V = Visit; XP = Procedure or test for the subjects initially randomized in the Placebo arm for the 6 first months,

1. Including a systematic complete skin examination, other physical examination as required,
2. Blood pressure, heart rate and respiratory rate.
3. FEV1 will be measured for subjects ≥6 years of age (unless they have documented inability to adequately perform spirometry).
4. PEF will be measured for all the subjects ≥5 years of age.
5. For both FAQLQ and FAIM, subjects ≥8 years of age will use the Child Form of the FAQLQ/FAIM. All parents/guardians will use the Parental Form. The FAQLQ and FAIM forms will be completed.
6. Peanut-specific IgE, peanut-specific IgG4, peanut-specific-component IgE and peanut-specific-component IgG4 to Ara h 1, Ara h 2, Ara h 3, Ara h 8 and Ara h 9. IgE specific to cow's milk, to egg white, to house dust mites, and to grass pollen will be tested at screening, month 3, 6, 9, 12, 18, 24, 30, 36 for all subjects and Month 42 for subjects initially in the placebo group.
7. Laboratory tests performed centrally. Hematology: hemoglobin, hematocrit, platelets, red blood cells, white blood cells with differential cell count. Biochemistry: aspartate aminotransferase, alanine aminotransferase, total bilirubin, blood urea nitrogen, creatinine, total protein.
8. Signing the consent for the filaggrin genetic analysis can be done any time after the subject is included in the study. However, collection of blood is done only once at Visit 5 or 6.
9. Check the reactions of the skin on the back of the subject and grade the severity of the local skin reactions. At Visit 6, grading is to be done before patch application and 30 min, 1 h, 2 h and 3 h after patch application.
10. A maximum of 2 open peanut-food challenges can be performed during the course of the study. The first food challenge should be performed after a minimum of 12 months of active treatment. The 2nd peanut challenges can be performed for the same subject within the treatment course only after at least 12 additional months of active treatment have been administered to that subject.
11. Peanut challenge might be conducted at this visit only for the subjects initially randomized in the active arm.
12. Procedures during the unscheduled visits will be performed as deemed necessary by the investigator.

☎ Phone Contacts at M6.5 M8 M21 M27 M33 M39 (M39 for patients randomized in the Placebo arm for the 6 first months)

Time windows:

Visit	Time windows
V6 (M6) V8 (M9) V9 (M12) V10 (M15)	+/- 7 days
PC (M6.5)	+/- 2 days
V7 (M7) PC (M8)	+/- 3 days
V11 (M18) V12 (M24) V13 (M30) V14 (M36) V15P (M42) PC (M21, M27, M33, M39)	+/- 14 days
EoSV	≤ 2 weeks after V14 or V15P

3.3 Efficacy and Safety Variables

Criteria for Evaluation of Study Drug Safety

The following study drug safety criteria will be evaluated:

- AEs and treatment-emergent adverse events (TEAEs) by System Organ Class (SOC) and Preferred Terms (PTs);
- TEAEs by maximum severity, duration and relatedness to treatment patch, placebo or active;
- TEAEs leading to discontinuation;
- Incidence, duration and maximum severity of local cutaneous Viaskin® patch-induced AEs as assessed by the subject;
- Severity of local cutaneous Viaskin® patch-induced AEs as assessed by the Investigator;
- Adverse Events of Special Interest (AESI) including Grade 4 local cutaneous reactions and systemic allergic AEs considered related to Viaskin® patch;
- SAEs by SOC and PTs and SAEs relatedness to Viaskin® patch;
- Laboratory data, physical examinations and vital signs;
- Spirometry results and Peak Expiratory Flow (PEF) results;
- Safety sub-analysis in subjects with mutations in the filaggrin gene *versus* wild type subjects.

These parameters will be first studied separately in two Safety sub-Population groups, i.e. the Safety sub-Population Without history of Severe Anaphylaxis (SPwoSA) and the Safety sub- Population With history of Severe Anaphylaxis (SPwSA). If no major safety differences are observed (as determined by the Data and Safety Monitoring Board, during the second DSMB meeting), additional outputs pooling both sub-populations will also be produced. If the safety profile of the two sub-populations is not comparable, the table with the pooled sub-population will not be produced.

The results for the age ranges 4-5 years, 6-8 years and 9-11 years will be presented.

When performed during the study for the subject medical management, the AEs induced by a peanut-food challenge, as they are expressly provoked, will be differentiated from those AEs occurring outside of a challenge. Objective and subjective symptoms/reactions elicited during a challenge will be summarized separately:

- Symptoms elicited during a peanut-food challenge by severity;
- SAEs elicited during a peanut-food challenge.

Exploratory Criteria

The following exploratory criteria will be evaluated:

- Change from baseline at Month 3, 6, 9, 12, 18, 24, 30, 36 for all subjects and Month 42 for subjects initially in the placebo group:
    - Peanut-specific IgE;
    - Peanut-specific IgG4;
    - Peanut component-specific IgE
    - Peanut component-specific IgG4;
    - Peanut skin prick testing average wheal diameters;
- Description of the quality of life questionnaires (Food Allergy Quality of Life Questionnaire [FAQLQ]/Food Allergy Independent Measure [FAIM]);
- Change from baseline at Month 6, 12, 18, 24, 30, 36 for all subjects and Month 42 for subjects initially in the placebo group for:
    - FAQLQ global score
    - FAQLQ scores by domain
- Description of accidental consumptions of peanut during the study and analysis of “risk-taking behavior” of subjects (voluntary peanut consumption);
- Epigenetic modifications of the promoters of specific genes;
- Sensitization status to some other allergies and their evolution over the study period;
- Scoring atopic dermatitis (SCORAD) evolution over time;

For the subjects who will have open food challenge(s) performed during the study, the following criteria will be described:

- Symptoms reported during peanut-food challenges;
- Peanut Eliciting Dose (ED);
- Peanut Cumulative Reactive Dose (CRD);
- Change in CRD from the last historical CRD obtained from a peanut-food challenge performed before entering the study when available to the CRD from the peanut-food challenges performed at any time points during the study,
- Percentage of subjects reaching a CRD ≥1,000 mg peanut protein at any time point after 12 months of active treatment onwards

The above descriptions will be presented for food challenge(s) performed at any time during the period of active treatment with Viaskin® Peanut 250 µg, keeping in mind that peanut-food challenge should not be conducted for a subject before his/her 12 months of active Viaskin® Peanut 250 µg treatment.

4. STATISTICAL METHODS

4.1 Data Quality Assurance

All tables, figures and data listings to be included in the report will be independently checked for consistency and integrity in accordance with standard PAREXEL procedures as follows:

PAREXEL Biostatistics and Statistical Programming seeks to ensure the quality of the results provided for the study in the form of Tables, Figures and Listings (TFL), and the derived datasets used in their creation, through the following processes:

- Derived datasets will be independently reprogrammed by a second programmer. The separate datasets produced by the two programmers must match 100%.
- All Tables will be independently reprogrammed by a second programmer for numeric results and must match 100%.
- Statisticians will be involved in the process of programming and validating tables that include inferential statistical results.
- Figures will be checked for consistency against corresponding tables and listings, or independently reprogrammed if there are no corresponding tables or listings.
- Listings will be checked for consistency against corresponding tables, figures, and derived datasets.
- The entire set of TFL will be checked for completeness and consistency prior to its delivery to the sponsor by the Lead Statistical Programmer, the Lead Biostatistician, and a senior level statistician, or above, who is not a member of the project team.
- The validation process will be repeated any time TFL are redelivered using different data. Execution of this validation process will be documented through the study "Program Status Tracker" (created to track all programs for analysis variables, tables, listings and figures) that will be provided to the sponsor at study conclusion.

4.2 General Considerations

4.2.1 Statistical methods

The following conventions will be used when presenting summary statistics and analyzing continuous study data:

- Continuous data will be summarized in terms of number of subjects or observations with non-missing data (n), mean, standard deviation (SD), first quartile (Q1), median, third quartile (Q3), minimum (min) and maximum (max), unless otherwise stated.

- Compared to the number of decimals recorded for the raw data in the database, the statistics will be reported with the following number of decimals:
    - Minimum and maximum: same number
    - Mean, median, Q1, Q3: one extra decimal
    - Standard deviation: two extra decimals.
- For any parameter at a specific visit:
    - Change from Baseline will be calculated as the value of that parameter at that visit minus the Baseline value of that parameter
    - Relative change from Baseline (%) will be calculated as (the value of that parameter at that visit minus the Baseline value of that parameter) divided by the Baseline value of that parameter * 100
- For laboratory parameters for which a limit exists, values documented as below a limit or above a specific value (e.g. <0.35 kU/L or >100 kU/L) will be considered as equal to the limit/the specific value (e.g. = 0.35 or 100 kU/L) in the statistical analyses (except in listing where the reported value will be presented).
  In case of change of detection limit during the course of the study (e.g. from <0.35 kU/L to <0.1 kU/L), all values lower than the highest limit value will be imputed to this threshold (e.g. all values lower than 0.35 kU/L will be considered as equal to 0.35 kU/L). This conservative approach will ensure that changes from Baseline are consistent.

The following conventions will be used when presenting summary statistics for categorical study data:

- Categorical data will be summarized in terms of the number of subjects or observations providing non-missing data at the relevant time point (n), frequency counts and percentages. Any planned collapsing of categories will be detailed in the SAP text and the data displays.
- Percentages will be presented to one decimal place. Percentages will not be presented for zero counts. Percentages will be calculated using n (number of subjects with available data in the population) as the denominator. If sample sizes are small, the data displays will show the percentages, but any textual report will describe frequencies only.
- Change from Baseline will be summarized using shift tables where appropriate.

The following conventions will be considered regarding study assessments and time points:

- Date and time of the first patch application will be reported in the eCRF. The baseline value is the last measurement before date and time of first patch application (see section 4.2.3 Definition of Baseline).
- The ‘end of study’ assessment is defined as the last available post-treatment assessment.
- “Treatment Day” will be calculated relative to date of first patch application (Treatment day 1 = Date of first patch).

- Assessments collected at unscheduled visits will not be included in summary tables but only listed.
- Assessments outside of protocol allowable windows will be taken into account in the analysis according to the visit in which the data are entered.
- Early termination assessments will be analyzed as having occurred at the next scheduled assessment.

The following conventions will be considered regarding confidence p-values and confidence intervals (CI):

- P-values greater than or equal to 0.001, in general, will be presented to three decimal places. P-values less than 0.001 will be presented as "<0.001".
- Confidence intervals will be presented to one more decimal place than the raw data.

The following conventions will be applied on diary data dates, for each incomplete patch application or removal date:

- Incomplete application date:
    - During the first 14 days of patch application: imputed as removal dates (if removal date is complete).
    - Beyond the first 14 days: imputed as removal date -1 (if removal date is complete).
- Incomplete removal date:
    - During the first 14 days of patch application: imputed as application date (if application date is complete).
    - Beyond the first 14 days: imputed as application date +1 (if application date is complete).

No imputations will be performed on time of application/removal unless explicitly described for specific analyses.

Titles for tables and listings produced will specify the type of data reported and well as the population concerned (in parentheses).
For table and/ or listings duplicated by age class, the identification of the age class will be mentioned in the table before any display of the results.

Listings will generally include the following identification variables: subject identifier, sex and age, treatment group.
Unless specified, complete dates will be reported in listings using a DDMMMYYYY format (i.e.: 01JAN2015) and time using a 24h clock-time (i.e.: 20:35).
Partial dates will be reported as MMMYYYY or YYYY.

All report outputs will be produced using SAS® version 9.3 or a later version in a secure and validated environment. All report outputs will be provided to the Sponsor in a single Microsoft Word document.

4.2.2 Type of analyses

Without loss of generality, the following terminology will be uniformly used in this document:

- Placebo = patients randomized to placebo
- Viaskin Peanut 250 µg = patients who were randomized to Viaskin Peanut 250 µg group.
- Rescheduled = for analyses, all timepoints will be temporally matched (as described in Table 3 below). This is labelled as “rescheduled”.

Two types of analyses will be performed in the study:

- Analysis on the Double-Blind treatment Period (also called in this document “DBP analysis”). Such analyses cover the analysis of endpoints collected during the double-blind period of the study (i.e. up to Visit 6 at Month 6). The Safety Analysis during the DBP is considered as the primary analysis of the study.Analysis of endpoints occurring during Active Treatment Period (also called in this document “ATP analysis”).

For ATP analysis, the assessments will be compared according to timepoints redefined according to the duration of the active treatment received in both groups as described below.

Table 3: Definition of timepoints for the ATP analyses

Duration of Viaskin® Peanut (months)	Day/ Month of Active treatment (DA/MA)	Randomized Group	
		Viaskin® Peanut 250 µg	Placebo
Initiation	DA1	V2 (D1)	V6 (M6)
1	MA1	V4 (M1)	V7 (M7)
3	MA3	V5 (M3)	V8 (M9)
6	MA6	V6 (M6)	V9 (M12)
9	MA9	V8 (M9)	V10 (M15)
12	MA12	V9 (M12)	V11 (M18)
18	MA18	V11 (M18)	V12 (M24)
24	MA24	V12 (M24)	V13 (M30)
30	MA30	V13 (M30)	V14 (M36)
36	MA36	V14 (M36)	V15P (M42)

Safety endpoints prior to Month 6 will be analyzed by treatment groups and tables will show “VP 250” (for Viaskin® Peanut 250 µg) group *versus* “Placebo” group. Safety endpoints post Month 6 will be analyzed using the above rescheduling rules and tables will show the VP 250 group, the “Rescheduled Placebo” group, and both groups pooled together.

4.2.3 Definition of Baseline

For the DBP analyses, the baseline value will be the last valid pre-dose assessment available. In other words, V1 or V2 data will be taken into account for the Baseline, unless the date/time clearly indicates that the value is after the patch application.
For the ATP analyses, the “Rescheduled baseline” assessment will be the last valid pre-active dose assessment available (V1 or V2 for Viaskin Peanut® 250 µg arm / V6 for placebo arm, unless the date/time clearly indicates that the value is after the patch application of active treatment). Some assessments are only performed at V1. The “Rescheduled baseline” values for these assessments will be the value at V1.

For the DBP analysis, the baseline will be considered in the outputs.
For the ATP analysis, the rescheduled baseline will be considered in the outputs.

4.2.4 Treatment groups

For the DBP analyses, the treatment groups to be compared will be:

- ”VP 250”
- “Placebo”

For the ATP analyses, the treatment groups compared will be:

- ”VP 250” (Patients randomized to Viaskin® Peanut 250 µg in the double-blind period)
- “Rescheduled Placebo” (Patients randomized to Placebo in the double-blind period)

4.2.5 Study assessments

- Assessments for the DBP analysis are defined according to the visits from Visit 1 to Visit 6.
- Assessments for the ATP analyses are defined according to the duration of treatment with Viaskin® Peanut 250 µg from Day of Active Treatment 1 (DA1) to Month of Active Treatment 36 (MA36) (see 0).
- The ‘end of study’ assessment is defined as the last available post-treatment assessment.
- The ‘study day’ is calculated as:
    - For DBP analysis: Start/Stop Date of Event – Date of First Dose of investigational product + 1 for dates on or after first dose, or Start/Stop Date of Event – First Dose of investigational product for others.
    - For ATP analysis: Start/Stop Date of Event – Date of First Dose of <u>active</u> investigational product + 1
- Assessments collected at unscheduled visits will not be included in summary tables but only listed (except for food challenge data).

- Assessments outside of protocol allowable windows will be taken into account in the analysis according to the visit in which the data are entered.
- Early termination assessments will be analyzed as having occurred at the next scheduled assessment
- If more than one laboratory value is available for a given visit, the first valid observation will be used in summaries and all observations will be presented in listings. It is noted that invalid laboratory data may not be used (from hemolyzed samples, mishandled samples, quantity not sufficient, or other conditions that would render values invalid).

4.2.6 Missing Data Conventions

Best efforts should be made by the Investigator to provide complete data.
Analyses of safety endpoints:

- Partial or missing safety data will be imputed according to the most conservative approach. Adverse events with missing or incomplete onset date will be considered as Treatment-emergent AEs unless there is evidence that the event occurred prior to the treatment period. Treatment-emergent AEs with missing relationship to the study drug will be considered as drug-related and TEAEs with missing severity will be considered as severe. Actual values will be presented in listings.

Analyses of Exploratory Endpoints:

- No imputation will be performed and observed data will be used.

4.3 Study Subjects

4.3.1 Disposition of Subjects

A clear accounting of the disposition of all subjects who enter the study will be provided, from screening to study completion.
Subject disposition will be summarized for all subjects, overall and by treatment group.
Reason for non-randomization will be listed for screened subjects who failed to be randomized.
The number of screened subjects will be provided.
The number and percentage of the following categories of subjects will be tabulated overall and by treatment group on the safety population:

- Subjects who completed the double-blind period
- Subjects who discontinued the study during or at the end of the double-blind period
- Subjects who discontinued the study after the double-blind period
- Primary reason for study discontinuation
- Subjects who discontinued the study treatment during or at the end of the double-blind period
- Subjects who discontinued the study treatment after the double-blind period
- Primary reason for study treatment discontinuation.

Since safety of subjects will be also analyzed according to the presence or absence of a medical history of severe anaphylaxis to peanut (appendix 0), the number and percentage of subjects with and without history of severe anaphylaxis will also be tabulated (cf. section 3.3).

An enrollment summary will be presented overall, by country (USA, Canada) and by site showing the number of subjects screened, randomized, treated, completing the double-blind period and completing the study and among randomized subjects: the first date of

consent, the last study visit date, the study duration (in days - calculated as last study visit exit date – first date of consent +1).
Study disposition and termination details will be listed for each subject. Listings will also be created to show the study analysis population classifications and randomization assignments.

4.3.2 Protocol Deviations

Deviations from the protocol including violations of inclusion/exclusion criteria will be assessed as "minor" or "major" in cooperation with the Sponsor prior to unblinding.
The impact of major protocol deviations that affect the safety evaluation will be investigated by assessing the robustness of the study results on the Per-Protocol populations in which such subjects will be excluded.
Observable protocol deviations data will be entered into PAREXEL's Clinical Trials Management System (CTMS). Some protocol deviations will be programmed and edited in listings. The study team and the Sponsor will conduct on-going reviews of the protocol deviation data from the CTMS and the listings and the resulting set of evaluable subjects throughout the study, adjusting the deviation criteria (major/minor) as appropriate.
The classification of protocol deviations must be finalized prior to database lock.
Major protocol deviations and any action to be taken regarding the exclusion of subjects or affected data from specific analyses are specified in 6.2 Appendix 2: Major Protocol deviations.
The number and percentage of subjects with major protocol deviations will be summarized by type of deviation, overall and by treatment group for the randomized population. All protocol deviations will also be listed.

4.4 Analysis Populations

Upon database lock, analysis population outputs will be produced and will be sent to DBV for review. These outputs will be reviewed and discussed during the Blind Data Review Meeting (BDRM) and it will be decided which subjects and/or subject data will be excluded from certain analyses. Decisions made regarding the exclusion of subjects and/or subject data from analyses will be made prior to blind breaking and will be documented and approved by DBV and PAREXEL in the BDRM minutes.

A summary of the number of subjects included in each analysis population described below (Randomized population, Safety population and Per-Protocol population [PP]) will be provided overall and by treatment group. Percentage of subjects in the PP population will be calculated on the Randomized population.

A by-subject listing of analysis population (on screened subjects) will be provided ordered by treatment group and will include: treatment group, subject identifier, and inclusion/exclusion flag for each population and reason for exclusion from each population.

The populations described in the following paragraphs will be considered.

4.4.1 Screened population

The screened population consists of subjects whose parents/guardians have signed informed consent.

4.4.2 Randomized population

The randomized population consists of subjects who have been randomized into the study. This population will be used to describe subject disposition and baseline characteristics. Subjects will be analyzed according to the treatment they have been randomized to.

4.4.3 Safety Populations & Safety Sub-Populations

Two safety populations will be defined according to the two types of analyses:

- DBP Safety Population: will be comprised of all subjects who are randomized and have received at least one dose of study drug. In case the wrong study drug is dispensed, the subject will be analyzed according to the study drug received for the longest period of time during the DBP. This population will be used to assess the study drug safety for the DBP analyses.
- ATP Safety Population: will be comprised of all subjects who are randomized and have received at least one dose of active study drug. This population will be used to assess the study drug safety for the ATP analyses.

A subject is assumed to have received at least one dose of study drugif the date/time of first dose is completed or partially completed

Peanut allergic subjects presenting a medical history of severe anaphylaxis after peanut consumption (Food-induced anaphylaxis reaction of grade 3, see appendix 5 of protocol version 3.0) will be identified among the Safety population in order to define the two sub-populations described below:

- Safety Sub-Population 1: Subjects **without** history of severe anaphylaxis to peanut (SPwoSA).
  This sub-population will include all subjects from the Safety population who do not present a medical history of severe anaphylaxis to peanut.

- Safety Sub-Population 2: Subjects **with** a history of severe anaphylaxis to peanut (SPwSA).

This sub-population will include all subjects from the Safety population presenting a medical history of severe anaphylaxis to peanut.

Analysis tables will be produced for both the overall safety population and the two safety sub-populations (unless otherwise specified by the DSMB members during the second DSMB meeting).

However, the safety profile of the subjects will be compared between the two safety sub-populations 1 and 2:

- If no major safety differences are observed as evaluated by the DSMB, both sub-populations will be pooled and the primary safety analyses considered will be the ones conducted on the overall Safety population on the DBP analysis.
- If the safety profile of the two sub-populations is not comparable, the primary safety analysis considered will be the ones conducted on the two sub-populations separately on the DBP analysis.

For the ATP analysis, the two safety sub-populations will be pooled whatever the comparability of the safety profile. Therefore, the outputs will be produced on the overall Safety population only.

4.4.4 Per-protocol Population & sub-populations

The PP population will include all subjects from the Randomized population who do not have major deviations from the protocol that may affect the safety evaluation. The deviations to consider are listed in Appendix 0 and will be reviewed during the Blind Data Review Meeting. The PP population will be used to assess the robustness of the safety evaluation. This population will be analyzed according to study treatment that was actually received by the subjects during the DBP. In case the wrong study drug is dispensed, the subject will be analyzed according to the study drug received for the longest period of time.

In case the 2 Safety sub-populations defined above are not comparable (see 4.4.2), two PP sub-populations will be considered separately for the analysis:

- PP Sub-Population 1: Subjects from the PP population without a history of severe anaphylaxis to peanut.
- PP Sub-Population 2: Subjects from the PP population with a history of severe anaphylaxis to peanut.

4.4.5 Interim and Final Analyses

Interim analyses of the safety will be performed at the following scheduled timepoints:

- When all subjects reach 6 months of double-blind treatment. This analysis will be the "DBP analysis" (as described in section 4.2.2).
- When all subjects reach 12 months of treatment (i.e. 12 or 6 months of active treatment). This analysis will be an ATP analysis of the first 6 months of active treatment (Rescheduled Placebo vs. Viaskin® Peanut 250 µg)
- When all subjects reach 12 months of active treatment (i.e. 12 months after the study start for the subjects randomized in active treatment arm and 18 months after the study start for the subjects initially randomized in placebo treatment arm). This analysis will be an ATP analysis of the first 12 months of active treatment (Rescheduled Placebo vs. Viaskin® Peanut 250 µg)
- When all subjects reach 24 months of active treatment (i.e. 24 months after the study start for the subjects randomized in active treatment arm and 30 months after the study start for the subjects initially randomized in placebo treatment arm). This analysis will be an ATP analysis of the first 24 months of active treatment (Rescheduled Placebo vs. Viaskin® Peanut 250 µg)

The final analysis will be an ATP analysis of 36 months of active treatment (36 months after the study start for the subjects randomized in active treatment arm and 42 months after the study start for the subjects initially randomized in placebo treatment arm).

4.4.6 Data and Safety Monitoring Board

A DSMB composed of experts in food allergy and in the methodology of clinical studies will be established in due time for the first data review. This DSMB will be independent of the Sponsor and will review safety data from the study at specified intervals during the study and on an *ad hoc* basis as deemed necessary by the DSMB Chair person or when conveyed by the Sponsor.

During these review meetings, the DSMB will assess whether the nature, frequency, and severity of the AEs associated with the study treatment warrant any recommendations or corrective actions of the study conduct in the best interest of the subjects.

The DSMB will also evaluate at each analysis, if the Safety of the sub-Population **with** history of Severe Anaphylaxis (SPwSA) is not different from the Safety sub-Population **without** history of Severe Anaphylaxis (SPwoSA).

The roles, responsibilities, constitution, and operations of the DSMB will be described in the DSMB Charter, which will be reviewed and signed by each member.

Six DSMB data review meetings are scheduled. At each of these data review meetings, tables and listings will be produced.

4.4.7 Examination of Subgroups

In addition to the sub-population of patients defined according the presence or absence of any history of Severe Anaphylaxis to peanut, the safety analysis might be run on the following set of subjects:

- For all subjects
- For the subjects within the age range 4 to 5 years at visit 1: [4-6[years old
- For the subjects within the age range 6 to 8 years at visit 1: [6-9[years old
- For the subjects within the age range 9 to 11 years at visit 1: [9-12[years old
- For the subjects with a null mutation on the filaggrin gene (either heterozygous or homozygous mutation) vs subjects with the wild type filaggrin gene. (The data will provide with clear identification of whether the subject has a null mutation ("heterozygous" or "homozygous") or is wild type (no specified mutation). More details on filaggrin mutation are described in Appendix 6)

4.5 Demographics and Baseline characteristics

Demographic characteristics and Baseline characteristics will be summarized and listed for the safety population.
Balance between treatment groups at Baseline on the Safety population (globally and by Sub-population) will be assessed using statistical tests on a selection of key variables, identified in the below sections.
Statistical tests used will be:

- a Student's t–test for continuous variables anticipated to have a normal distribution
- a Mann-Whitney test for continuous variables anticipated to have a non-normal distribution
- a χ^2 test for categorical variables (Fisher's exact test if one or more theoretical frequency is ≤5).

4.5.1 Demographic variables

Demographic characteristics that will be reported in summary tables include:

- Sex
- Age (years) populated by the IWRS at visit 1,
- Age in classes
  - 4 to 5 (6 excluded)
  - 6 to 8 (9 excluded)
  - 9 to 11 (12 excluded)
- Race/Ethnicity,
- Filaggrin null mutation group (see Appendix 6):
  - Subjects with null mutation on the filaggrin gene

        - Heterozygous
        - Homozygous
    - Wild type subjects.

When the date of birth is partial (only year available or year and month), the earliest possible date will be considered (i.e. first of January or first day of the month).

Comparability at Baseline will be assessed using statistical tests on age, age in classes and sex.

4.5.2 Baseline Characteristics

Baseline characteristics that will be reported in summary tables include:

- Body weight (kg) [weight (in kg)] = weight (in lb.) / 2.204623]
- Height (cm) [height (in cm) = 2.54 * height (in inches)]
- Body mass index (kg/m²): weight / (height (in m) * height (in m)),
- Spirometry: FEV1 value (L), FEV1 Percent Predicted (%), (also to be reported by age class 6-8 and 9-11 years)
- Peak Expiratory Flow (PEF): PEF value (L/min), PEF percent predicted (%), (also to be reported by age class 5, 6-8 and 9-11 years),
- Immunological markers: peanut-specific IgE (kU/L), peanut-specific IgG4 (mg/L),
- Skin Prick Test (SPT): Mean and longest wheal diameter for undiluted Peanut extract (mm)
- Medical history (see 4.5.3 Medical history)
- Disease history (see 4.5.5 Disease history)
- Parental atopic medical history (see 4.5.4 Parental atopic medical history)

For ATP analysis, for all the above parameters (except Disease history, Parental atopic Medical history and Filaggrin mutation group) the value at rescheduled baseline will be presented.

4.5.3 Medical history

The documentation of the complete medical history will include the other current medical conditions, past or present cardiovascular, respiratory (including asthma), gastrointestinal, renal, hepatic, neurological, endocrine, lymphatic, hematologic, immunologic, dermatological (including atopy), psychiatric, developmental, and genitourinary disorders, drug and surgical history and any other diseases or disorders.

Medical history will be reported by SOC and PT and coded using the latest available version of the Medical Dictionary for Regulatory Activities (MedDRA) dictionary.

Medical history covers the collections, at D1 baseline, of information on past (within the previous 12 months) or current conditions (apart from peanut allergy) along with start and end dates including:

- Any allergy other than peanut
    - ▪ PT terms which contains “ALLERG” (excluding LLT “Peanut allergy”) and PT terms which contains “HYPERSENSITIVITY” (excluding LLT “Peanut allergy”)
- Other atopic conditions (Exhaustiveness of the list of terms specified below will be reviewed during the data review meetings):
    - o Asthma
        - ▪ SMQ Asthma/bronchospasm latest MedDRA Version from narrow terms: List of PT terms: Asthma, asthma exercise induced, asthma late onset, asthmatic crisis, bronchospasm, bronchial hyperreactivity, infantile asthma, status asthmaticus, wheezing
    - o Eczema/Atopic Dermatitis
        - ▪ List of PT terms: dermatitis atopic, dermatitis Allergic, Eczema, Application site eczema, Eczematous dermatitis, dermatitis contact.
    - o Allergic Rhinitis
        - ▪ List of PT terms: Rhinitis Allergic, seasonal allergy, Rhinitis perennial, conjunctivitis allergic.
- Past or current medical conditions:
    - o Cardiovascular, respiratory (including asthma), gastrointestinal, renal, hepatic, neurological, endocrine, lymphatic, hematologic, immunologic, dermatological (including atopy), psychiatric, developmental, and genitourinary disorders, drug and surgical history and any other diseases or disorders.

They will be coded using the latest available version of the Medical Dictionary for Regulatory Activities (MedDRA) and will be reported by SOC and PT. The number and percentage of subjects with at least one medical history term will be also provided

4.5.4 Parental atopic medical history

Parental (father only, mother only or both parents) atopic medical history covers: asthma, seasonal allergies, perennial allergies, food allergies, eczema/atopic dermatitis and other allergic diseases. The number and percentages of subjects with at least one parental atopic medical history will be also provided.

4.5.5 Disease history

Disease history covers the collection of information about the peanut allergy of the subject and its diagnosis. The following characteristics will be reported in summary tables:

- Age at peanut allergy diagnosis (in years, calculated as Date of diagnosis – Date of birth +1 divided by 365.25),
- Time since peanut allergy diagnosis (in years, calculated as Date of Informed consent - Date of diagnosis +1 divided by 365.25),
- Category of the physician who made the diagnosis (Pediatrician or Pediatrician allergist, Private practice allergist, Public hospital allergist, Emergency department/room, Other)
- Reason for diagnosis (Reaction after contact with or ingestion of food containing peanut, Parental history of atopy/allergy, Sibling(s) history of atopy/allergy, Child with high risk factors of peanut allergy)
- Risk factors of peanut allergy (Atopic dermatitis, Cow’s milk allergy, Egg allergy, Any other food allergy, Any Respiratory allergy, Drug allergy, Other atopic disease)
- Diagnosis criteria (Allergic reaction(s) following peanut consumption, Positive SPT to peanut, Positive titer of peanut-specific IgE, Positive Double Blind Placebo Controlled Food Challenge to peanut, Positive Single Blind Placebo Controlled Food Challenge to peanut, Positive Open Food Challenge to peanut)
- Most recent results of peanut allergy diagnostic tests performed prior to the study visit,
- Allergic reactions related to peanut consumption prior to the study (per subject):
    - Number of reactions related to peanut consumption in the past,
    - Number of reactions after the age of 2.
    - Number of reactions after ingestion of peanut in the previous 12 months,
        - Time since last reaction after ingestion of peanut (in years, calculated as Date of Informed consent - Date of last reaction +1). When the date of last reaction is partial (only year available or year and month), the latest possible date will be assumed (e.g. 31st of December or last day of the month). In case, the imputed date is after to the inform consent date, the inform consent date will be considered as the imputation date.

When the date of diagnosis is partial (only year available or year and month), the latest date between the date of birth and the earliest possible date of diagnosis (i.e. first of January or first day of the month) will be considered.

4.6 Previous and concomitant medications

Prior (used in the last 6 months prior to screening) and concomitant medications will be coded using the latest available version of the World Health Organization (WHO) Drug Dictionary. Summaries of prior and concomitant medications will be produced by preferred drug name.

All medications taken before the study entry, at study entry and during the study will be collected and coded using the latest available version of the World Health Organization (WHO) Drug Dictionary.

Medications will be flagged to identify:

- Prior medications
- Concomitant medications of the DBP
- Concomitant medications of the ATP

This classification will be made by comparing the study medication start and stop dates with:

- For the DBP:
  - the date of first application of any study medication
  - the day before the first application of the open-label period.
- For the ATP:
  - the date of first application of Viaskin® Peanut 250 µg
  - the date of study completion/withdrawal.

Medications starting after the completion/withdrawal date will be listed but will not be classified or summarized.

Prior medications are medications:

- starting and/or stopping within 6 months before the date of first application of study medication of the DBP,
- with partial stop date where there is a clear evidence to suggest that it stopped within 6 months prior to the date of first application of study medication of the DBP,
- with partial start date where there is a clear evidence to suggest that it started within 6 months prior to the date of first application of study medication of the DBP.
- Starting before the 6 months prior the date of first application of study medication of the DBP and still ongoing.

Concomitant medications of the DBP are medications:

- starting or stopping during the DBP,
- with partial start date where there is a clear evidence to suggest that the medication started during the DBP,
- with partial stop date where there is a clear evidence to suggest that the medication stopped during the DBP,
- with partial start/stop date where there is a clear evidence to suggest that the medication started prior to the DBP (no evidence for the stop date),
- with partial start/stop date where there is <u>no</u> clear evidence to suggest that the medication started or stopped during the DBP.

Concomitant medications of the ATP are defined the same way as for the DBP but using dates of the ATP as a reference.

For prior and/or concomitant medications, number and percent of subjects overall and per ATC class (level 3) and preferred drug name will be calculated. The following summaries will be presented:

1. Prior medications;

2. Concomitant medications (other than the ones taken for voluntary or accidental consumption of peanut and other than the ones taken due to a food challenge to peanut):
    - For the double-blind period;
    - For the active-treatment period (for the patients randomized to Placebo in the double-blind period: concomitant medications of the open-label period only - For the patients randomized to Viaskin® Peanut 250 µg in the double-blind period: concomitant medications of both the double-blind and the open-label periods)
3. Concomitant medications taken due to accidental/voluntary consumption of peanut on the same periods as described above
4. Concomitant medications taken as an action following an AE/SAE (including the ones taken for voluntary or accidental consumption of peanut and the ones taken due to a food challenge to peanut) on the same periods as described above
5. Concomitant medications taken due to a food challenge to peanut

Number of antihistamines and number of epinephrine intakes during food challenges will also be tabulated separately.

Non-drug therapies and surgical procedures are collected and will be coded using the latest available version of the MedDRA Dictionary. They will be also presented by SOC and preferred term and listed.

Prior and concomitant medications as well as Non-drug therapies and surgical procedures will be summarized by treatment group and overall for the safety population.

For ATP analysis, prior and concomitant medications will be determined according to rescheduled baseline date.

All prior and concomitant medications, non-drug therapies and surgical procedures will be listed.

4.7 Study duration and study treatment characteristics

4.7.1 Study duration

The overall maximum total study duration for each subject will be approximately ranges from 36 to 42 months:

- 2-week screening period,
- 6 months double-blind treatment period,
- 30 months (for patients randomized to Viaskin® Peanut 250 µg in the double-blind period) to 36 months (for patients randomized to Placebo in the double-blind period open-label treatment period,

- 2-week follow-up period.

Study duration (days) will be calculated as:

- Last visit performed date - Date of informed consent +1.

4.7.2 Treatment exposure

The exposure duration (in days, regardless of treatment interruption) will be calculated for both the DBP and the ATP. The exposure duration (in days, regardless of treatment interruption), calculated for the following periods:

- Over the whole study period as: Date of last patch application – Date of first patch application +1,
- At each study visit as: Date of last patch application before the visit – Date of first patch application at the preceding visit+1.

Exposure duration will be expressed as descriptive statistics and as number and percentage of patients according to the following classes of duration:

For the DBP:

- 1-7 days
- 8-14 days
- 15-30 days
- 31-90 days
- ≥91days

For the ATP:

- 1-7 days
- 8-14 days
- 15-30 days
- 31-90 days
- 91-185 days
- 186 days – 365 days (12 months)
- 366 days – 550 days (12-18 months)
- 551 days – 730 days (18-24 months)
- 731 days – 915 days (24-30 months)
- 916 days – 1095 days (30-36 months)
- ≥1096 days (≥36 months)

During the study, the Viaskin® patch must be applied on the skin for 24 hours every day, except during the first two weeks of the double blind period and the open label period, where the duration of application of the Viaskin® patch will be progressively increased as follows:

- During the first week, the patches will be applied for 6 hours every day,

- During the second week, the patches will be applied for 12 hours every day,
- From the third week onwards, the patches will be applied for the entire 24 hours daily.

The average daily application duration (in hours) will be summarized descriptively using the subject diary data for the following periods:

For the DBP:

 - D1 to D7,
 - D8 to D14,
 - D15 to M3,
 - M3 to M6,
 - Overall double-blind period.

For the ATP:

 - DA1 to DA7,
 - DA8 to DA14
 - DA15 to MA3,
 - MA3 to MA6,
 - MA6 to MA9,
 - MA9 to MA12,
 - MA12 to MA18,
 - MA18 to MA24,
 - MA24 to MA30,
 - MA30 to MA36 (or end of treatment),
 - Overall active treatment period (DA15 to MA36 or end of treatment)

If more than one patch is applied the same day, the daily duration considered is the cumulative application duration of these patches.
In case patches are sequentially applied on the same day: if the end date/time of first patch is not documented, the application date/time of the next patch will be used for imputation.

Since no queries are edited for diaries, inconsistent date/time of patch application can remain in the data.

A patch will be considered as "applied" if the date and time of application is documented in the diary.
The start and/or end date/time of patch application used to calculate the duration of patch application are extracted from the diary.
Inconsistencies in the diaries are not queried and only self-evident corrections according to the Data Validation specifications will be made; therefore, inconsistent date/time of patch application or patch removal may remain in the data.
In case where calculation of patch application duration leads to a negative value then:

- The duration of patch application will be considered as 'unknown' and not used in the calculation of the average duration of patch application

- For any other calculation based on the number of days with patch application, this day will be considered as a day with application

The percentage of days with no patch application, per subject diary reported data, will also be tabulated using descriptive statistics, overall and for each of the above periods.

4.7.3 Total dose of peanut protein

The total dose of protein (in mg) received via the patch for a specific period will be calculated as:
Exposure duration (in days) of the period * Actual treatment dosage.
The periods considered will be:

  - o During the DBP (for patients randomized in the active treatment group)
  - o During the ATP
  - o At each study visit

Dose of protein will be expressed using descriptive statistics.

4.7.4 Compliance

The compliance (%) will be determined as follow:

- For the double-blind and the active treatment periods as:

$$100 * \frac{\text{Number of patches dispensed} - \text{Number of patches returned}}{\text{Exposure duration (in days)}},$$

- At each study timepoint during the double-blind period (D8, M1, M3, M6) and during the active treatment period (MA1, MA3, MA6, MA9, MA18, MA24, MA30, MA36):

$$100 * \frac{\text{Nb of patches dispensed at the visit (n -1) - Nb of patches returned at visit (n)}}{\text{Date of visit (n) - Date of visit (n -1)}^{(1)}}$$

(1) Date of current visit – Date of previous visit+1 for Day 8

Compliances exceeding 100% will be set to 100%.
Global compliance of at least 80% over the treatment period is sought.
Treatment compliance will be summarized by means of descriptive statistics (n, mean, SD, median, Q1, minimum, Q3, and maximum), frequency tables (compliance < or ≥80%) and by visit using the subject diary data for the following periods:

For the DBP:

- D1 to D8,
- D8 to M1,
- M1 to M3,
- M3 to M6,

For the ATP:

- DA1 to DA8,
- DA8 to MA1
- MA1 to MA3,
- MA3 to MA6,
- MA6 to MA9,
- MA9 to MA12,
- MA12 to MA18,
- MA18 to MA24,
- MA24 to MA30,
- MA30 to MA36 (or end of treatment),

4.8 Study Drug Safety Evaluation

The safety analysis will be run on the safety population (and sub-populations), successively on the following set of subjects:

- For all subjects
- For the subjects within the age range 4 to 5 years at visit 1: [4-6[years old
- For the subjects within the age range 6 to 8 years at visit 1: [6-9[years old
- For the subjects within the age range 9 to 11 years at visit 1: [9-12[years old

Some analyses will also be run for the subjects with a mutation on the filaggrin gene vs. wild type subjects.

During the ATP, the AEs induced by a peanut-food challenge, as they are expressly provoked, will be differentiated from those AEs occurring outside of a challenge. Objective and subjective symptoms/reactions elicited during a challenge will be summarized separately:

Results will be presented by treatment group and overall.
Unless specified no missing data will be replaced/imputed.

4.8.1 Adverse Events

This section covers the analysis of adverse events apart from the ones induced by a food-challenge. The analysis of symptoms and events elicited during a peanut-food challenge is covered in section 4.9.8 Peanut-Food Challenge.

TEAEs are any AEs, regardless of relationship to study drug and are either:

- New event occurring during or after the initial patch application of the referenced period (as described in 4.2.3 Definition of Baseline)

- Event already present before the referenced period that either worsens in severity or is related to study drug following exposure to patches during or after the initial patch application of the referenced period (as described in 4.2.3 Definition of Baseline)

All AEs will be coded using the latest available version of the MedDRA dictionary and reported by System Organ Class (SOC) and Preferred Term (PT).

AEs occurring after the end of the study will be recorded only if the investigator considers that there is a causal relationship with the study drug and as such, will be considered also as TEAEs.

Pre-treatment emergent AEs will be defined as AEs that begin before the first administration of study drug and that do not worsen in severity after the first administration of study drug.

Where dates are missing or partially missing, adverse events will be assumed to be treatment-emergent, unless there is clear evidence (through comparison of partial dates) to suggest that the adverse event started prior to the first dose of study treatment.

More specifically:

- Start date of AE missing: Assumed to be date of first treatment dose.
- Start day and month of AE missing: Assumed to be date of first treatment dose (if same year for both dates), January 1st otherwise.
- Start day of AE missing: Assumed to be date of first treatment dose (if same month and year for both dates), first day of the month otherwise.
- End date for non-ongoing AE missing: Assumed to date of last treatment dose
- End day and month for non-ongoing AE missing: Assumed to be date of last treatment dose (if same year for both dates), December 31st otherwise.
- End day for non-ongoing AE missing: Assumed to be date of last treatment dose (if same month and year for both dates), last day of the month otherwise.

TEAEs with missing severity will be considered as “severe”; TEAEs with missing relationship to the study drug will be considered as “drug-related”.

TEAEs reported as related, probably related, or possibly related to the study drug will be considered as related to study drug.

Viaskin Peanut-induced Local TEAEs are defined as TEAEs considered as related to IP with a High Level Term equal to “Application and instillation site reactions” and will be flagged in the analysis datasets.

For each summary presented by SOC and PT, the SOC “General disorders and administration site conditions” will be tabulated globally as well as split in two subsections:

- General disorders (Preferred Term not specifying "Application site"),
- Administration site conditions (Preferred Term specifying "Application site").

An overall overview table of AEs will be provided showing the number of subjects, the percentage of subjects and the number of events, overall and for each treatment group, for the following categories of AEs:

- Any AE,
- Any serious AEs,
- AEs leading to study treatment discontinuation,
- AEs leading to death,
- Any mild AEs,
- Any moderate AEs,
- Any severe AEs.

The overall overview of AEs will only be presented for the DBP analysis.

An overview table of TEAEs will be provided showing the number of subjects, the percentage of subjects and the number of events for the following categories of TEAEs:

- Any TEAEs,
- Any serious TEAEs,
- Any TEAEs considered related to study drug:
    - Any TEAEs reported as Related,
    - Any TEAEs reported as Probably related,
    - Any TEAEs reported as Possibly related,
- Any TEAEs considered unrelated to study drug:
    - Any TEAEs reported as Unlikely related,
    - Any TEAEs reported as Unrelated,
- Any Serious TEAEs considered related to study drug,
- Any TEAEs leading to study permanent discontinuation
- Any TEAE leading to temporary treatment discontinuation,
- Any TEAEs leading to death,
- Any mild TEAEs,
- Any moderate TEAEs,
- Any severe TEAEs,
- Any severe TEAEs considered related to study drug,
- Any Viaskin® Peanut Induced Local TEAEs
- Any severe Viaskin® Peanut induced Local TEAEs
- Any systemic allergic TEAE considered related to IP (reported as related, probably or possibly related)
- Any TEAE leading to an epinephrine intake and relatedness to Viaskin® Peanut 250 µg

For TEAEs(#), TEAEs considered related, SAEs, serious TEAEs, serious TEAEs considered related, TEAEs leading to permanent study treatment discontinuation(#), TEAEs leading to temporary study treatment discontinuation(#), Viaskin Peanut-induced Local TEAEs(#) and TEAEs leading to death, detailed tables will be created showing the number of subjects who experienced at least one TEAE, the corresponding percentage of subjects and the number of events by SOC and PT.

The variables marked with (#) will also be presented for the subjects with a mutation on the filaggrin gene vs. wild type subjects.

Additional tables will be created to display the most frequent TEAEs (by preferred term), occurring in at least 5% of the subjects in any of the treatment group.

Additionally, tables showing the number and percentage of subjects who experienced at least one TEAE and the number of events by SOC and PT, summarizing TEAEs leading to:
- Epinephrine intake:
  - Standard medication name that contains “EPINEPHRINE”
- Systemic or inhaled corticosteroid intake,
  - ATC code starting with “H02”
  - ATC code starting with “A07EA”, “C05”, “R01” and (ROUTE in "ORAL" "RECTAL" "RESPIRATORY (INHALATION)") or (ROUTE=OTHER with other, specify that contains ("IV" "INTRAVENOUS"))
- Topical corticosteroid intake,
  - ATC code starting with “D07”
  - ATC code starting with “A07EA”, “C05”, “R01” and ROUTE = Topical

will be presented (exhaustiveness of the above selections, especially regarding the route that can be reported as “Other” with a text field specification, will be checked during the blind data review meeting).

These TEAEs will be identified according to the 2 conditions below:
- In the AE form: TEAEs for which a medication is administered (“Were there any medication administered for this adverse event? answered “Yes”)
- In the Concomitant medication form for the medication considered: corresponding AE numbers documented in the field “Number of adverse events for which the medication was taken”

For TEAEs and TEAEs considered related to IP, additional tables will be provided showing number of subjects and percentage of subjects:
- By maximum severity (severe, moderate and mild): Subjects will be counted once per SOC and once per PT at the worst severity. If the severity is missing, the worst severity will be assumed.

- By maximum duration. For these tables TEAEs will be presented by SOC, PT within each SOC and maximum duration within each PT (using the classes for duration in days: 1-7, 8-15, 16-30, 31-60, 61-90, above 91). For AEs ongoing at the end of DBP/ATP, duration will be imputed based on the date of subject end of DBP/ATP. For AEs not ongoing but with missing end date, duration will be imputed based on date of last treatment dose for DBP/ATP.

Additionally, for TEAEs and TEAEs considered related to IP, tables showing number of subjects, percentage of subjects and number of events by severity will be provided. Subjects will be counted once per SOC, per PT and per severity. For these specific tables, if the severity is missing, no hypothesis will be assumed to replace the missing severity.

Adverse event summaries will be ordered in terms of decreasing frequency for SOC, and PT within SOC, in the Viaskin® treatment group, and then similarly by decreasing frequency in the placebo group, and then alphabetically for SOC, and PT within SOC.

All AEs will be listed. A separate listing will be provided for TEAEs leading to death.

The proportions of patches that led to mild, moderate or severe TEAE considered related to study drug will be summarized on the Safety Set. For this analysis, the worst ongoing severity will be considered for each day of exposure and the following definitions will be used:

- % of patches leading to severe TEAE =

$$100\,\text{x}\left(\frac{\text{Number of days with ongoing severe TEAE}}{\text{Exposure duration}}\right)$$

- % of patches leading to moderate TEAE =

$$100\,\text{x}\left(\frac{\text{Number of days with ongoing moderate}^{(1)}\text{ TEAE}}{\text{Exposure duration}}\right)$$

(1) without concomitant event of severe intensity

- % of patches leading to mild TEAE =

$$100\,\text{x}\left(\frac{\text{Number of days with ongoing mild}^{(2)}\text{ TEAE}}{\text{Exposure duration}}\right)$$

(2) without concomitant event of moderate or severe intensity

The proportion of patches that led to Viaskin® Peanut 250 µg-induced local TEAEs will also be summarized the same way as above. The proportion of patches that led to Viaskin® Peanut 250 µg-induced local TEAEs will also be summarized for the subjects with a mutation on the filaggrin gene vs. wild type subjects.

A by-subject listing of all adverse events will be provided. This listing will be presented by treatment group and will include: treatment group, subject identifier, age, sex, adverse event (SOC, PT, and verbatim term), date/time of onset (and corresponding study day), date/time of resolution (and corresponding study day), duration, severity, treatment required, relationship to study drug, relationship to an accidental peanut consumption and relationship to Food Challenge, action taken with the study drug, outcome, and whether the event is classified as serious or not with the corresponding criteria.
Pre-treatment emergent adverse events will be listed separately the same way as the treatment emergent adverse events.
TEAE leading to an epinephrine intake will also be listed.
Finally, SAEs will also be listed the same way with an additional flag for treatment emergent SAEs.

4.8.2 Adverse event of special interest (AESI)

Treatment emergent adverse events of special interest (AESI), as defined in the protocol, are:

- Treatment emergent grade 4 local cutaneous reactions or any significant skin lesion which could potentially lead to skin barrier disruption at sites of patch application (identified with AETERM which contains "GRADE 4" " (or "GRADE IV") and High Level Term equal to "Application and instillation site reactions"),

- Any occurrence of IgE-mediated systemic-type of symptoms distant from the patch application site and considered at least possibly related to the study drug. These allergic systemic TEAEs of interest will be identified through the algorithm of the Anaphylactic Reaction Standardized MedDRA queries (SMQ) (methodology described in the Appendix 5).

All AESI data will be listed.

An overall overview table of systemic allergic TEAEs cases will also be provided showing the number of subjects, the percentage of subjects and the number of cases for the following categories of systemic AESI overall and for subjects with filaggrin gene mutations versus wild type subjects, using the definition of the protocol (related only) and in addition showing any systemic allergic reaction considered as unrelated to IP:

- Any systemic allergic TEAE cases will be considered as related to IP if they are
  - Reported as Related,
  - Reported as Probably related,
  - Reported as Possibly related,
- Any systemic allergic TEAE cases will be considered as unrelated to IP if they are:
  - Reported as Unlikely related,

- Reported as Unrelated

They will be reported in summary tables showing the number and percentage of subjects affected and the number of underlying AE events by SOC and PT, overall and for subjects with filaggrin gene mutations versus wild type subjects. Summary tables will be split for systemic allergic TEAE cases considered related, systemic allergic TEAE cases considered unrelated and treatment emergent local cutaneous reactions.

4.8.3 Subject Diaries (Local skin reactions)

Number and percentage of subjects with evaluable diary will be presented. Local skin reactions at sites of Viaskin® application as assessed by the patient using diary during the double-blind period of the study will be summarized for itching, redness, swelling as well as any local reaction together using:

- The number and percentage of subjects by maximum severity grade reported (0, 1, 2 or 3) among subjects with evaluable diary and among subjects with a reaction (grade ≥ 1),
- The number and proportion of days with a reaction reported (based on days with patches graded by type of reaction or for at least one reaction between itching, redness and swelling when reported on any local reaction) among subjects diary data,
- The proportion of days scored by severity grade (worst severity grade per day is considered).
  All proportions will be calculated as

$$\left(\frac{Number\ of\ days\ with\ a\ reaction\ reported}{Number\ of\ days\ with\ patches\ graded}\right)$$

Proportion of days with no assessment of local skin reaction out of the number of patches entered in the diary will be provided.
The number of days with a reaction reported and number of days with patches graded will be calculated based on removal dates.

The following periods of the double-blind period will be considered for the reporting:

- The overall double-blind period (D1 to D180),
- Month 1 (D1 to D30),
- Month 2 (D31 to D60),
- Month 3 (D61 to D90),
- Month 4 (D91 to D120),
- Month 5 (D121 to D150),
- Month 6 (D151 to D180 or date of Visit 6)

The local skin reactions reported by the subject will also be summarized for the subjects with a mutation on the filaggrin gene vs. wild type subjects.

All local skin reactions reported by the subject will be listed.

4.8.4 Skin reactions (as graded by the investigator)

Local skin reactions at sites of patch application are graded from Grade 0 (negative) to Grade 4 (erythema, vesicles) by the investigator at each visit.
Specifically at Visit 2 (Baseline) and at Visit 6 (Month 6), grading is to be completed before patch application and at 30 min, 1 h, 2 h and 3 h after patch application.
These graded reactions will be reported in summary tables showing the number and percentage of subjects:

- By grade (by localization and regardless of the localization) by study visit (same as above)
    - For the DBP analysis: Baseline, D8, M1, M3 and M6
    - For the ATP analyses: Rescheduled Baseline, MA6, MA7, MA9, MA12, MA15 (rescheduled ex-placebo only), MA18, MA24, MA30, MA36.
- By most severe grade (by localization and regardless of the localization) for the overall treatment period.

The local skin reactions as graded by the investigator will also be summarized for the subjects with a mutation on the filaggrin gene vs. wild type subjects.

All local skin reactions as graded by the investigator will also be listed.

4.8.5 Deaths

As detailed in the preceding section, a detailed table will be created showing the number of subjects who experienced at least one TEAE leading to Death, the corresponding percentage of subjects and the number of events group by SOC and PT.

All deaths will be listed in a summary table.

4.8.6 Clinical Laboratory Evaluation

The following laboratory evaluations will be performed at Baseline and at fixed and unscheduled post-baseline visits:

- Hematology: hemoglobin, hematocrit, platelets, red blood cells, white blood cells (neutrophils, lymphocytes, monocytes, eosinophils, basophils in value and %);
- Biochemistry: alanine aminotransferase, aspartate aminotransferase, total bilirubin, total protein, blood urea nitrogen, creatinine;

The following results will be tabulated for hematology and biochemistry tests on the safety population at all fixed scheduled visits:

- Descriptive statistics for each test value at the following visits:
  - For the DBP analysis: Baseline, M3 and M6,
  - For the ATP analyses: Rescheduled Baseline, MA3, MA6, MA12, MA18, MA24, MA30, MA36.
- Descriptive statistics for changes from Baseline at each post-baseline visit listed above.
- Common Toxicity Criteria for Adverse Events (CTCAE version 4.03) grade at each visit. CTCAE grades for hematology and biochemistry measures will be derived according to 6.3 Appendix 3: Common Terminology Criteria for Adverse Events (CTCAE) grades.
- Shift of test abnormalities between Baseline and post-baseline visits

The above descriptions will be repeated on each age range (4 to 5 years, 6 to 8 years, and 9 to 11 years)

All laboratory data will be listed.
Values that are out of normal range will be flagged in the data listings.
Flags used are:

- Low based on the following original flags from the laboratory reports:
  - PL, L2 ("Panic Low")
  - TL, L1 ("Telephone Low")
  - L ("Low")
- High based on the following original flags from the laboratory reports:
  - H ("High")
  - TH, H1 ("Telephone High")
  - PH, H2 ("Panic High")

When summarized in tables, all “High” flags will be merged together (“Panic High”, “Telephone High”, ”High”) ; the same for the low flags as well.
In listings, original flags will be reported.
Pregnancy test data will be reported only as listings.

Listings of all laboratory data will be provided by treatment group and will include subject identifier, age, sex, weight and visit. Laboratory reference ranges will also be listed.
In the event of unexplained abnormal laboratory test values of clinical significance, the tests should be repeated at a reasonable time point and followed up until they have returned to the normal range and/or an adequate explanation of the abnormality is found.
Those repeated assessments performed outside scheduled visits will appear in listings only.

4.8.7 Vital Signs

The following vital signs will be recorded at Baseline (Visit 1) and at fixed and unscheduled post-baseline visits:

- Systolic blood pressure (in mmHg),
- Diastolic blood pressure (in mmHg),
- Heart rate (beats/minute),
- Respiratory rate (breaths per minute).

Apart from the baseline visit, the following visits will be considered for reporting vital signs results in tables:

- For the DBP analysis: Screening, Baseline, D8, M1, M3 and M6
- For the ATP analyses: Rescheduled Baseline, MA3, MA6, MA9, MA10, MA12, MA15, MA18, MA24, MA30, MA36.

The following results will be tabulated for all vital signs:

- Summary statistics of values by visit
- Changes from Baseline by visit (from rescheduled baseline for ATP analyses)
- Number and percentage of subjects with vital signs abnormalities by post-baseline visit for heart rate, systolic blood pressure and diastolic blood pressure classified using the criteria defined in table 4.

Table 4: Criteria to Determine Clinically Relevant Abnormalities in Vital Signs

**Vital Sign Criteria for Abnormalities**	**Criteria for Abnormalities (any of the following situation)**
Heart rate	• value <60 beats/min,<br>• value >130 beats/min,<br>• an increase from pre-dosing of >20 beats/min, or<br>• a decrease from pre-dosing of >20 beats/min
Systolic blood pressure	• value <70 mmHg<br>• value >130 mmHg,<br>• an increase from pre-dosing of > 40 mmHg, or<br>• a decrease from pre-dosing of >30 mmHg
Diastolic blood pressure	• value <45 mmHg<br>• value >85 mmHg,<br>• an increase from pre-dosing of >30 mmHg, or<br>• a decrease from pre-dosing of >20 mmHg

Vital signs data and clinically relevant abnormalities in vital signs will also be listed.

4.8.8 Physical Examination

A physical examination will be performed at Baseline and at fixed and unscheduled post-baseline visits (the same as for vital signs).
Physical examination will include the measure of the weight (kg) and height (cm) and the examination of the following:

- General appearance,
- Head and Neck,
- Ears, nose and throat,
- Eyes,
- Complete skin examination,
- Cardiovascular system,
- Respiratory system,
- Abdominal system,
- Nervous system,
- Other system(s).

The following results will be tabulated:

- Summary statistic for weight, height BMI and BSA by visit
- Change from Baseline by visit (from rescheduled baseline for ATP analyses),
- Number and percentage of subjects with abnormal (vs. normal / not done) physical examination by visit for each of the system examined.

Physical examination abnormal findings (especially skin abnormal findings) observed during the physical examinations will be listed.

4.8.9 Spirometry and Peak Expiratory Flow Results

Spirometry test (FEV_1) (for subjects >= 6 years of age capable of performing spirometry) and PEF (for subjects >=5 years of age) will be performed at Baseline and at post-baseline and unscheduled visits.
The following results will be tabulated:

- Summary statistics for FEV1 value (L) and FEV1 percent predicted by visit:
    - For the DBP analysis: Baseline and M6,
    - For the ATP analyses: Rescheduled Baseline, MA6, MA12, MA18, MA24, MA30, MA36.
- Change of value from Baseline at each visit (from rescheduled baseline for ATP analyses),
- Summary statistics for PEF value (L) and PEF percent predicted by visit:
    - For the DBP analysis: Baseline, D8, M1, M3, M6,

  - For the ATP analyses: Rescheduled Baseline, MA1, MA3, MA6, MA12, MA18, MA24, MA30, MA36.
- Change of value from Baseline and at each fixed post-baseline visit.

Spirometry and Peak Expiratory Flow Results will also be listed.

4.9 Exploratory Analyses

Exploratory analyses will be performed on the safety population and the PP population (and sub-population), for both the DBP and ATP analyses.

4.9.1 Immunological markers

Descriptive statistics will be provided for Peanut-specific IgE and IgG4 on the following values:

- Actual values for each fixed visit
    - For the DBP analysis: Baseline, M3 and M6
    - For the ATP analyses: Rescheduled Baseline, MA3, MA6, MA9, MA12, MA18, MA24, MA30 and MA36.
- Relative changes from baseline for each fixed visit (same as above)

The same results will be provided for:

- Peanut component-specific IgE and IgG4
- IgE specific to cow's milk,
- IgE specific to hen's egg,
- IgE specific to house dust mites,
- IgE specific to grass pollen.

Mean (± SD) and median (+/- IQR) relative change from Baseline in peanut-specific IgE and IgG4 will be presented graphically, by treatment group.

The $\log_{10}$ transformation of the ratio between peanut specific IgG4 and peanut specific IgE, calculated as:

$$log_{10}\left(\frac{Peanut\ specific\ IgG4(mg/L) \times 1000}{Peanut\ specific\ IgE\ (kU/L) \times 2.4}\right)$$

The analysis will be run on the safety population and PP population (and sub-populations), successively on the following set of subjects at each timepoint:

- For all subjects
- For the subjects within the age range 4 to 5 years at visit 1: [4-6[years old
- For the subjects within the age range 6 to 8 years at visit 1: [6-9[years old
- For the subjects within the age range 9 to 11 years at visit 1: [9-12[years old

4.9.2 Skin Prick Test

Descriptive statistics will be provided for Skin Prick Test (average wheal diameter for undiluted skin prick) on the following values:

- Actual values for each fixed visit
  - For the DBP analysis: Baseline, M3 and M6
  - For the ATP analyses: Rescheduled Baseline, MA3, MA6, MA12, MA18, MA24, MA30 and MA36.
- Absolute changes from baseline for each post-baseline fixed visit (same as above)

4.9.3 Food Allergy Quality of Life Questionnaire /Food Allergy Independent Measure

Food Allergy Quality of Life Questionnaires (FAQLQ)

The FAQLQs (Child Form [CF] and Parent Form [PF]) are disease-specific health-related quality of life questionnaires for subjects with food allergy.
At screening and post baseline fixed visits, the following questionnaires will be completed (for countries where translated and validated questionnaires are available and used):
By subjects, for subjects above 8 years of age (at screening):

- FAQLQ-CF: 24 items,

By parents for all subjects:

- FAQLQ-PF: 26 items for subjects aged 4 to 6; 30 items for subjects older than 7 (at screening),

Age at screening will be used to define which questionnaires will be applicable during the study for each patient. Items not applicable will not be used for analysis.
FAQLQ questionnaires will be summarized on specific scores calculated by main domains of quality of life. FAQLQ-CF will be analyzed according to the 4 following domains [1]:

- EI: Emotional impact (item no: 19-24),
- AA: Allergen avoidance (item no: 4, 6-10, 15),
- RAE: Risk of accidental exposure (item no: 11, 13-14, 16-17),
- DR: Dietary restriction (item no: 1-3, 5, 12, 18).

FAQLQ-PF will be analyzed according to the 3 following domains [2]:

  - EI: Emotional impact (item no: 2, 6-7, 9-11, 23-28, 30),
  - FA: Food-related anxiety (item no: 1, 4-5, 16-17, 20-21, 29),
  - SDL: Social and Dietary limitations (item no: 3, 8, 12-15, 18-19, 22).

The following instructions from the reference website (www.faqlq.com) will be used for the derivations of (sub-) scales:

1) Each question of the FAQLQ is answered on a 7-point scale (0 to 6) and should be recoded 1 to 7. (Note: FAQLQ-CF items are coded on a scale of 1-7 in the CRF and in SDTM)

2) The total FAQLQ-scores (except for FAQLQ-PF for which the total score is equal to the sum of the three-scores score divided by 3) and all sub-scores are calculated by dividing the sum of completed items by the number of completed items

3) If > 20% of items in any (sub-) domain are missing then the respective (sub-) score is set to missing

The scores range from no problem/impairment to maximal problem/impairment. A negative score in the change from baseline means impairment.

Food Allergy Independent Measure (FAIM)

The FAIM questionnaires capture the subjects' expectation of something happening because of her/his food allergy.

Each question of the FAIM is answered on a 7-point scale (0 to 6) and should be recoded 1 to 7.

Total FAIM scores will be calculated by dividing the sum of completed items by the number of completed items. Total FAIM scores range from 1 “low perceived disease severity” to 7 “high perceived disease severity”.
Calculation of total FAIM scores will be done only when 80% or more of the items are completed.
Some specific items as mentioned below have to be reversed.

FAIM-CF: Child Form (8-12 years)
This questionnaire consists of 6 items. The mean score will be performed if at least 5 items are completed.

FAIM-PF: Parent Form (Children aged 0-12 years)
There are 2 sections given in the questionnaire each with the same questions :

- one from the perspective of the parents (*What chance do you think your child has of.....?*)
- one reflecting the thoughts of the child (*What chance does your child think he/she has of......?*).

Both mean scores of the parent’s form (“Parent’s thoughts” and “Child’s thoughts”) are calculated as the mean of the single items, only if none of the items is missing. In both scores question 4 needs to be reverse scored.

The quality of life scores will be summarized on the safety population by treatment arm using descriptive statistics for:

- Value at Baseline and at each post-baseline visit :

- For the DBP analysis: M6
- For the ATP analyses: MA6, MA12, MA18, MA24, MA30 and MA36.

- Change from Baseline at each visit (from rescheduled baseline for ATP analyses) (same as above).

FAQLQ and FAIM data will also be listed.

4.9.4 Accidental Consumption of Peanuts

The following results will be reported in a summary table:

- Number and percentage of subjects with any (at least one) peanut consumption and number of consumptions (in classes: 1, 2...)
- Number and percentage of subjects with any (at least one) confirmed accidental consumption of peanut and number of accidental consumptions (in classes: 1, 2...)
- Number and percentage of subjects with any (at least one) not confirmed accidental (deliberate consumption of peanut -supposed to be voluntary-) and number of voluntary consumptions (in classes: 1, 2...)
- Number and percentage of subjects with induced reaction (overall, by maximum severity and by type of symptom as classified by SOC and PT)
- Number and percentage of subjects with treatment taken following accidental peanut consumption (overall and by type of treatment as classified by ATC class (level 3) and preferred drug name)

The above descriptions will be repeated on each age range (4-5 years, 6-8 years, and 9-11 years).
All data related to accidental/voluntary peanut consumption will be listed. These data include: confirmation (or not) of accidental consumption, food consumed, estimated quantity consumed at each occurrence, and associated reactions and severity of reactions.
All SAEs (allergic reactions) caused by consumption of peanut will be listed.

4.9.5 Epigenetic analyses

Descriptive analyses of epigenetic modifications of specific genes will be performed using actual values, change from baseline and relative change from baseline using observed data. Please note that Epigenetic data will not be available at time of 6 months interim database lock.

4.9.6 Genetic Screening

Treatment-emergent AEs (0) and local skin tolerance at sites of Viaskin® patch application (**Error! Reference source not found.**) will be described among subjects with mutations in the filaggrin gene *versus* subjects carrying the wild type gene.

4.9.7 SCORAD (Scoring Atopic Dermatitis)

SCORAD total score will be imputed as 0 if the subject answer “No” to the question:
”Did subject have any areas of atopic dermatitis ?”
Summary statistics on SCORAD total score will be presented on actual values (no imputation) for:

- Value at Baseline and at fixed post baseline visits:
  - For the DBP analysis: M3 and M6
  - For the ATP analyses: MA3, MA6, MA12, MA18, MA24, MA30 and MA36.
- Change from Baseline (from rescheduled baseline for ATP analyses) at fixed post baseline visits.

4.9.8 Peanut-Food Challenge

The open peanut-food challenge(s) performed at the initiative of the investigators during the course of the study will be described, when available, such as:

- Peanut Eliciting Dose (ED),
- Peanut Cumulative Reactive Dose (CRD),
- Change in CRD from the last historical CRD obtained from a peanut-food challenge performed before entering the study when available to the CRD from the peanut-food challenges performed at any time points during the study,
- Percentage of subjects reaching a CRD ≥1,000 mg peanut protein.

The above descriptions will be presented for food challenge(s) performed at any time during the period of active treatment with Viaskin® Peanut 250 µg, keeping in mind that no peanut-food challenge can be conducted for a subject before his/her12 months of active Viaskin® Peanut 250 µg treatment.
The following periods will be used:

- From the beginning of the active treatment to 9 months of treatment (food challenges should not occur before 12 months of treatment);
- From beginning of Month 9 to end of Month 15 of the active treatment;
- From beginning of Month 16 to end of Month 27 of the active treatment;
- From beginning of Month 28 to the end of the active treatment.

The objective symptoms collected during the food challenges are the following:

- Skin:
    - Erythematous rash (and % of rash area concerned)
    - Pruritus
    - Urticaria/ angioedema
    - Rash
- Upper respiratory:
    - Sneezing/ itching
    - Nasal congestion
    - Rhinorrhea
    - Laryngeal
- Lower respiratory:
    - Wheezing
- Gastrointestinal:
    - Diarrhea
    - Vomiting
- Cardiovascular
- Eyes:
    - Conjunctivitis
- Any other objective symptoms (not reported, mild, moderate, severe)

The subjective symptoms collected are:
- Itchy mouth
- Itchy throat
- Nausea
- Abdominal pain
- Any other subjective symptoms (not reported, mild, moderate, severe)

With the exception of erythematous rash (recorded as Yes/No), each symptom is graded as: 0=”absent”, 1=”mild”, 2=”moderate” or 3=”severe”. For erythematous rash, the % area involved is collected. The grading corresponding to the area involved is captured in the grading of the “Rash” symptom. No further step is required.

A total symptom score for each subject will be calculated as the sum of all severity scores for all objective symptoms (excluding erythematous rash and subjective complaints). This will be based on each symptom being graded for severity as 0, 1, 2 or 3 (respectively absent, mild, moderate and severe).
The reactions appearing during a Food challenge (as they are expressly provoked) will be differentiated from those AEs occurring outside a food challenge. They will be summarized by group showing, for the above specified periods:
- The number and percentage of subjects with objective symptoms by severity (overall and by type of symptom)
- The number and percentage of subjects with subjective symptoms by severity (overall and by type of symptom)

All SAEs elicited during the peanut-food challenge will be listed.

4.10 Determination of Sample Size

The sample size of this study, based on an expected exposure to Viaskin® Peanut 250 µg close to 600 subject-years in the safety database at the time of registration filing, is defined at 250 subjects randomized in the active treatment group. This number of subjects enables the detection events with an annual rate ≥0.024 after 6 months of follow-up in this study and will increase the overall Viaskin® Peanut 250 µg safety database size to a number of subject-years allowing the detection of events with annual incidence rates ≥0.0055.

It is planned to screen approximately 480 subjects to reach 335 subjects randomized in the treatment period of this study (anticipated 30% screen failure rate). Assuming a drop-out rate of 15% per year, this will ensure that approximately 284 subjects will complete the first year of treatment, 242 subjects will complete the study up to the end of year 2 and 205 subjects will complete the study up to the end of year 3.

The subjects will be randomized at the start of the study with a 3:1 ratio, leading to approximately 250 subjects in the active arm and 85 subjects in the placebo arm.

Throughout the screening process and to ensure an adequate and sufficient representation of the youngest subjects from age range 4 to 6 years of age, a minimum of 90 subjects aged less than 6 years old will be enrolled in the active arm from the 120 total number of randomized subjects in this age range. This will represent a minimum of 36% of the overall number of subjects treated with active treatment at the end of the first 6 months.

4.11 Changes in the Conduct of the Study or Analysis planned in the protocol

- FAQLQ questionnaires (Child form and Parent form) will be analyzed using a global score and score by domain (as described in the protocol) and, additionally, by item.

- The proportion of patches leading to mild, moderate or severe TEAE occurring during the exposure period will be studied. This additional safety description will be performed on the Safety population.

- The randomization was not stratified by site.

5. REFERENCES

[1] Flokstra-de Blok BM, DunnGalvin A, Vlieg-Boerstra BJ et al. Development and validation of a self-administered Food Allergy Quality of Life Questionnaire for children. Clin Exp Allergy 2009; 39(1):127-137.

[2] DunnGalvin A, de BlokFlokstra BM, Burks AW, Dubois AE, Hourihane JO. Food allergy QoL questionnaire for children aged 0-12 years: content, construct, and cross-cultural validity. Clin Exp Allergy 2008;38(6):977-986.

6. APPENDICES

6.1 Appendix 1: Staging System of severity of Anaphylaxis

Anaphylaxis is a generalized allergic reaction that is rapid in onset and may progress to death (Adapted from[2]).

Staging System of Severity of Anaphylaxis	
**Stage**	**Defined by**
**1 Mild**<br>skin & subcutaneous tissues, GI, &/or mild respiratory	Flushing, urticaria, periorbital or facial angioeodema; mild dyspnea, wheeze or upper respiratory symptoms; mild abdominal pain and/or emesis
**2 Moderate**<br>mild symptoms + features suggesting moderate respiratory, cardiovascular or GI symptoms	Marked dysphagia, hoarseness, and/or stridor; shortness of breath, wheezing & retractions; crampy abdominal pain, recurrent vomiting and/or diarrhea; and/or mild dizziness
**3 Severe**<br>hypoxia,<br>hypotension (more than 20% drop in blood pressure) or<br>neurological compromise	Cyanosis or SpO2 ≤ 92% at any stage, confusion, cardiovascular collapse, loss of consciousness, incontinence, bradychardia, cardiac arrest.

[2] Sampson HA, Muñoz-Furlong A, Campbell RL, Adkinson NF Jr, Bock SA, Branum A, et al. Second symposium on the definition and management of anaphylaxis: summary report—Second National Institute of Allergy and Infectious Disease/Food Allergy and Anaphylaxis Network symposium. J Allergy Clin Immunol. 2006;117(2):391-7.

6.2 Appendix 2: Major Protocol deviations

This table provide a sample of attitute taken regarding inclusion or exclusion of subjects with protocol deviations in the analysis sets. Categories of major protocol deviations will be discussed and finalised during blind data review meetings.

Protocol Deviation	Action to be taken for analysis
Subject did not take any study medication	Exclude from all population except Randomized population
Subject took incorrect study medication	Analyze ‘As-randomized’ for Randomized Population Analysis. Analyze ‘As-treated’ for Safety Population Analysis. Exclusion from PP Population will be discussed
Subject failed to meet study inclusion criteria but was entered into the study.	Exclusion from PP Population will be discussed
Subject met study exclusion criteria but was entered into the study.	Exclusion from PP Population will be discussed
Subject met study withdrawal criteria but was not withdrawn.	Exclusion from PP Population will be discussed
Subject non-compliant with study medication regimen (<80% compliant)	Exclusion from PP Population will be discussed
Subject diary (dispense/check) not done	Exclusion from PP Population will be discussed
**Subject non-compliant with study assessment schedule:**	
Visit not within a pre-specified window after baseline visit.	Exclusion from PP Population will be discussed
Subject took prohibited concomitant medication	Exclusion from PP Population will be discussed

6.3 Appendix 3: Common Terminology Criteria for Adverse Events (CTCAE) grades version 4.03

Haematology	G1	G2	G3	G4
Red blood cells (x10e12 /L)	No grading according to CTCAE			
Hematocrit (V/V)	No grading according to CTCAE			
Hemoglobin (g/L) - low	[100;LLN[	[80;100[	<80	
Hemoglobin (g/L) - high	]ULN;ULN+20]	]ULN+20;ULN+40]	>ULN+40	
Platelets (x10e9 /L) - low	[75;LLN[	[50;75[	[25;50[	<25
Leucocytes (x10e9 /L) - low	[3;LLN[	[2;3[	[1;2[	<1
Neutrophils (x10e9 /L) - low	[1.5;LLN[	[1;1.5[	[0.5;1[	<0.5
Eosinophils (x10e9 /L)	No grading according to CTCAE			
Basophils (x10e9 /L)	No grading according to CTCAE			
Lymphocytes (x10e9 /L) - low	[0.8;LLN[	[0.5;0.8[	[0.2;0.5[	<0.2
Monocytes (x10e9 /L)	No grading according to CTCAE			

Biochemistry	G1	G2	G3	G4
BUN/urea (mmol/L)	No grading according to CTCAE			
Total protein (g/L)	No grading according to CTCAE			
Total bilirubin (µmol/L) – high	]ULN; ULN*1.5]	]ULN*1.5; ULN*3]	]ULN*3;ULN*10]	>ULN*10
AST (U/L) - high	]ULN; ULN*3]	]ULN*3; ULN*5]	]ULN*5;ULN*20]	>ULN*20
ALT (U/L) - high	]ULN; ULN*3]	]ULN*3; ULN*5]	]ULN*5;ULN*20]	>ULN*20
Creatinine (µmol/L) - high	]ULN; ULN*1.5]	]ULN*1.5; ULN*3]	]ULN*3;ULN*6]	>ULN*6

6.4 Appendix 4: List of Post-Text Tables, figures, listings, and Supportive SAS output appendices

Will be provided in a separate document.

6.5 Appendix 5: Methodology of identification of systemic allergic adverse events

This methodology will be based on the MedDRA SMQ "Anaphylactic reaction" (MedDRA version in use at the time of study results analysis) as described in the "Introductory Guide for Standardised MedDRA Queries (SMQs) Version 20.0.

The retrieval will be performed in the SMQ anaphylactic reaction using an algorithmic approach which combines a number of anaphylactic reaction symptoms in order to increase specificity. A case must include either:

- A narrow term or a term from Category A;
- A term from Category B - (Upper Airway/Respiratory) AND a term from Category C - (Angioedema/Urticaria/Pruritus/Flush);
- A term from Category D - (Cardiovascular/Hypotension) AND [a term from Category B - (Upper Airway/Respiratory) OR a term from Category C - (Angioedema/Urticaria/ Pruritus/Flush)]

The events will be considered as concomitant for the algorithm output if they appear on the same day +/- 1 day.

- Related AESI will be determined if at least on term of any association in the algorithm is "related".
- Unrelated AESI will be determined if all terms of any association in the algorithm are not "related".

Where dates are missing or partially missing, adverse events are not considered for possible association."

6.6 Appendix 6: Definition of filaggrin null mutation group

Filaggrin null mutation group: subjects with null mutation on the filaggrin gene (Heterozygous / Homozygous) *versus* wild type subjects.

- A filaggrin null mutation is considered ("heterozygous", "homozygous" or "indeterminate") if a mutation is confirmed on at least one of the following possible loci: R501X, 2282Del4, R2447X, S3247X, 3702delG.
    - Heterozygous is defined as at least 1 null mutation on a single allele locus
    - Homozygous is defined as 2 identical null mutations on the same 2 allele loci of the FLG gene
- Wild type subjects will be identified as "Negative detection" on any of the five possible mutation loci
