# Supplementary material for: Efficacy and safety of peanut epicutaneous immunotherapy in patients with atopic comorbidities
Source: J Allergy Clin Immunol Glob. 2022 Sep 22;2(1):69–75. doi: 10.1016/j.jacig.2022.07.009 (PMC10509968; doi:10.1016/j.jacig.2022.07.009)
Supplement: Supplementary Tables E1-E3 [file mmc6.docx]

**Table E1. A Comparison of Key Eligibility Criteria for PEPITES and REALISE**

| **PEPITES** | **REALISE** |
| --- | --- |
| Age 4-11 years with history of IgE-mediated reactions to peanut | Age 4-11 years with history of IgE-mediated reactions to peanut |
| Subjects with history of severe anaphylaxis were excluded (ie, hypotension requiring vasopressor support, hypoxia requiring mechanical ventilation, or neurological compromise) | Subjects with history of severe anaphylaxis were not excluded |
| >0.7 kU_A_/L peanut-specific IgE | >14 kU_A_/L peanut-specific IgE |
| ≥8 mm SPT wheal diameter to peanut (≥6 mm SPT wheal diameter for children 4-5 years of age) | ≥8 mm SPT wheal diameter to peanut |
| Positive DBPCFC to ED ≤300 mg peanut protein. ED is defined as the dose at which objective symptoms occurred leading to the end of the DBPCFC | No DBPCFC |

DBPCFC=double-blind, placebo-controlled food challenge; ED=eliciting dose; IgE=immunoglobulin E; SPT=skin prick test.

**Table E2. Respiratory TEAEs in Subjects With and Without Asthma**

|  | **Asthma** | | **Without Asthma** | |
| --- | --- | --- | --- | --- |
|  | **Viaskin Peanut 250 µg**  **(n=243)** | **Placebo**  **(n=93)** | **Viaskin Peanut 250 µg  (n=289)** | **Placebo**  **(n=124)** |
| **Respiratory TEAEs, n (%)** | 118 (48.6) | 44 (47.3) | 109 (37.7) | 33 (26.6) |
| Asthma | 50 (20.6) | 20 (21.5) | 9 (3.1) | 0 |
| Cough | 40 (16.5) | 15 (16.1) | 31 (10.7) | 13 (10.5) |
| Wheezing | 20 (8.2) | 8 (8.6) | 8 (2.8) | 3 (2.4) |
| Dyspnea | 5.1 (2.1) | 3 (3.2) | 4 (1.4) | 0 |
| Bronchospasm | 2 (0.8) | 0 | 1(0.3) | 0 |

TEAEs=treatment-emergent adverse events.

**Table E3. SCORAD Evolution Over Time (PEPITES and REALISE Pooled Population)**

| **SCORAD Total Score^a^** | **With Atopic Dermatitis** | | **Without Atopic Dermatitis** | |
| --- | --- | --- | --- | --- |
|  | **Viaskin Peanut 250 µg** | **Placebo** | **Viaskin Peanut 250 µg** | **Placebo** |
| **Baseline, n** | 239 | 103 | 289 | 114 |
| **Median (Q1, Q3)** | 4.7 (0, 13.9) | 0.0 (0.0, 11.8) | 0.0 (0.0, 0.0) | 0.0 (0.0, 0.0) |
| **Range** | 0.0–51.3 | 0.0–57.5 | 0.0–27.0 | 0.0–19.8 |
| **Month 3, Change from baseline, n** | 235 | 98 | 285 | 114 |
| **Median (Q1, Q3)** | 0.0 (-5.4, 1.7) | 0.0 (-7.1, 1.2) | 0.0 (0.0, 0.0) | 0.0 (0.0, 0.0) |
| **Range** | -51.3–29.9 | -29.0–29.7 | -27.0–47.0 | -19.8–11.0 |
| **Month 6, Change from baseline, n** | 233 | 96 | 285 | 114 |
| **Median (Q1, Q3)** | 0.0 (-7.1, 0.8) | 0.0 (-5.3, 0.0) | 0.0 (0.0, 0.0) | 0.0 (0.0, 0.0) |
| **Range** | -51.3–32.7 | -27.0–67.6 | -51.4–31.3 | -19.8–19.6 |

^a^A high SCORAD total score is correlated with severe atopic dermatitis.

Q1=first quartile; Q3=third quartile; SCORAD=SCORing Atopic Dermatitis.
