# Supplementary material for: Efficacy and safety of peanut epicutaneous immunotherapy in patients with atopic comorbidities
Source: J Allergy Clin Immunol Glob. 2022 Sep 22;2(1):69–75. doi: 10.1016/j.jacig.2022.07.009 (PMC10509968; doi:10.1016/j.jacig.2022.07.009)
Supplement: Supplementary Table E4 [file mmc7.docx]

Table E4. **TEAEs by Treatment Group and With or Without Asthma and Atopic Dermatitis, and/or Concomitant Food Allergies – P3PC Pool**

1. **Atopic Dermatitis (AD), Asthma and concomitant food allergies (CFA) other than peanut.**

|  | **Asthma + AD + CFA** | | | |
| --- | --- | --- | --- | --- |
|  | With the 3 diagnoses | | Without the 3 diagnoses | |
|  | VP250 (N=88) | Placebo (N=32) | VP250 (N=444) | Placebo (N=185) |
| Any TEAE (n,%) | 82 ( 93.2) | 30 ( 93.8) | 407 ( 91.7) | 158 ( 85.4) |
| Mild | 79 ( 89.8) | 29 ( 90.6) | 384 ( 86.5) | 145 ( 78.4) |
| Moderate | 45 ( 51.1) | 15 ( 46.9) | 194 ( 43.7) | 63 ( 34.1) |
| Severe | 5 ( 5.7) | - | 13 ( 2.9) | 3 ( 1.6) |
| Serious | 2 ( 2.3) | 2 ( 6.3) | 7 ( 1.6) | 5 ( 2.7) |
| Treatment-Related | 43 ( 48.9) | 11 ( 34.4) | 187 ( 42.1) | 42 ( 22.7) |
| Serious Treatment-Related | 1 ( 1.1) | - | 3 ( 0.7) | - |
| TEAEs leading to permanent discontinuation | 2 ( 2.3) | - | 6 ( 1.4) | - |
| TEAEs leading to temporary discontinuation | 14 ( 15.9) | 4 ( 12.5) | 60 ( 13.5) | 14 ( 7.6) |
| Treatment-related local TEAEs | 34 ( 38.6) | 8 ( 25) | 157 ( 35.4) | 33 ( 17.8) |
| Anaphylactic reaction | 4 ( 4.5) | 1 ( 3.1) | 25 ( 5.6) | 4 ( 2.2) |

1. **Asthma and Atopic Dermatitis (AD)**

|  | **Asthma + AD** | | | |
| --- | --- | --- | --- | --- |
|  | With the 2 diagnoses | | Without the 2 diagnoses | |
|  | VP250 (N=134) | Placebo (N=49) | VP250 (N=398) | Placebo (N=168) |
| Any TEAE (n,%) | 124 ( 92.5) | 44 ( 89.8) | 365 ( 91.7) | 144 ( 85.7) |
| Mild | 118 ( 88.1) | 43 ( 87.8) | 345 ( 86.7) | 131 ( 78) |
| Moderate | 64 ( 47.8) | 22 ( 44.9) | 175 ( 44) | 56 ( 33.3) |
| Severe | 5 ( 3.7) | - | 13 ( 3.3) | 3 ( 1.8) |
| Serious | 2 ( 1.5) | 2 ( 4.1) | 7 ( 1.8) | 5 ( 3) |
| Treatment-Related | 58 ( 43.3) | 18 ( 36.7) | 172 ( 43.2) | 35 ( 20.8) |
| Serious Treatment-Related | 1 ( 0.7) | - | 3 ( 0.8) | - |
| TEAEs leading to permanent discontinuation | 2 ( 1.5) | - | 6 ( 1.5) | - |
| TEAEs leading to temporary discontinuation | 19 ( 14.2) | 5 ( 10.2) | 55 ( 13.8) | 13 ( 7.7) |
| Treatment-related local TEAEs | 46 ( 34.3) | 15 ( 30.6) | 145 ( 36.4) | 26 ( 15.5) |
| Anaphylactic reaction | 5 ( 3.7) | 1 ( 2) | 24 ( 6) | 4 ( 2.4) |

1. **Atopic Dermatitis (AD) and concomitant food allergies (CFA) other than peanut.**

|  | **Asthma + CFA** | | | |
| --- | --- | --- | --- | --- |
|  | With the 2 diagnoses | | Without the 2 diagnoses | |
|  | VP250 (N=146) | Placebo (N=58) | VP250 (N=386) | Placebo (N=159) |
| Any TEAE (n, %) | 138 ( 94.5) | 53 ( 91.4) | 351 ( 90.9) | 135 ( 84.9) |
| Mild | 131 ( 89.7) | 50 ( 86.2) | 332 ( 86) | 124 ( 78) |
| Moderate | 73 ( 50) | 25 ( 43.1) | 166 ( 43) | 53 ( 33.3) |
| Severe | 8 ( 5.5) | - | 10 ( 2.6) | 3 ( 1.9) |
| Serious | 3 ( 2.1) | 3 ( 5.2) | 6 ( 1.6) | 4 ( 2.5) |
| Treatment-Related | 64 ( 43.8) | 15 ( 25.9) | 166 ( 43) | 38 ( 23.9) |
| Serious Treatment-Related | 2 ( 1.4) | - | 2 ( 0.5) | - |
| TEAEs leading to permanent discontinuation | 2 ( 1.4) | - | 6 ( 1.6) | - |
| TEAEs leading to temporary discontinuation | 22 ( 15.1) | 7 ( 12.1) | 52 ( 13.5) | 11 ( 6.9) |
| Treatment-related local TEAEs | 55 ( 37.7) | 12 ( 20.7) | 136 ( 35.2) | 29 ( 18.2) |
| Anaphylactic reaction | 7 ( 4.8) | 2 ( 3.4) | 22 ( 5.7) | 3 ( 1.9) |

1. **Atopic Dermatitis (AD) and concomitant food allergies (CFA) other than peanut.**

|  | **AD + CFA** | | | |
| --- | --- | --- | --- | --- |
|  | With the 2 diagnoses | | Without the 2 diagnoses | |
|  | VP250 (N=154) | Placebo (N=67) | VP250 (N=378) | Placebo (N=150) |
| Any TEAE (n,%) | 142 ( 92.2) | 59 ( 88.1) | 347 ( 91.8) | 129 ( 86) |
| Mild | 138 ( 89.6) | 58 ( 86.6) | 325 ( 86) | 116 ( 77.3) |
| Moderate | 79 ( 51.3) | 25 ( 37.3) | 160 ( 42.3) | 53 ( 35.3) |
| Severe | 5 ( 3.2) | - | 13 ( 3.4) | 3 ( 2) |
| Serious | 2 ( 1.3) | 3 ( 4.5) | 7 ( 1.9) | 4 ( 2.7) |
| Treatment-Related | 77 ( 50) | 22 ( 32.8) | 153 ( 40.5) | 31 ( 20.7) |
| Serious Treatment-Related | 1 ( 0.6) | - | 3 ( 0.8) | - |
| TEAEs leading to permanent discontinuation | 3 ( 1.9) | - | 5 ( 1.3) | - |
| TEAEs leading to temporary discontinuation | 23 ( 14.9) | 6 ( 9) | 51 ( 13.5) | 12 ( 8) |
| Treatment-related local TEAEs | 62 ( 40.3) | 15 ( 22.4) | 129 ( 34.1) | 26 ( 17.3) |
| Anaphylactic reaction | 9 ( 5.8) | 2 ( 3) | 20 ( 5.3) | 3 ( 2) |
