# Supplementary material for: Efficacy and safety of peanut epicutaneous immunotherapy in patients with atopic comorbidities
Source: J Allergy Clin Immunol Glob. 2022 Sep 22;2(1):69–75. doi: 10.1016/j.jacig.2022.07.009 (PMC10509968; doi:10.1016/j.jacig.2022.07.009)
Supplement: Fig E1 [file mmc8.pptx]

## Slide 1
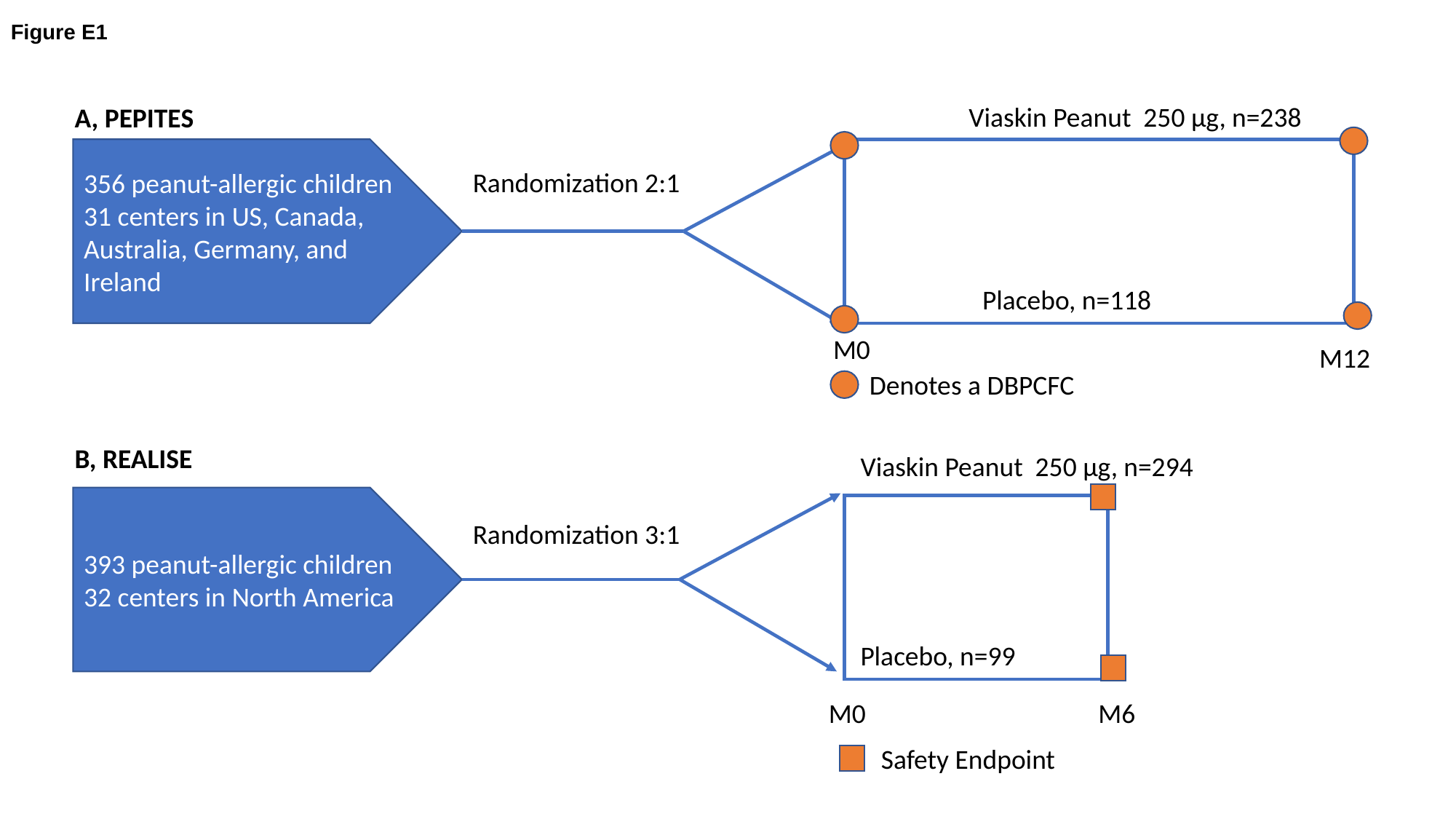

Figure E1
Viaskin Peanut 250 µg, n=238
A, PEPITES
356 peanut-allergic children
31 centers in US, Canada, Australia, Germany, and Ireland
Randomization 2:1
Placebo, n=118
M0
M12
Denotes a DBPCFC
B, REALISE
Viaskin Peanut 250 µg, n=294
393 peanut-allergic children
32 centers in North America
Randomization 3:1
Placebo, n=99
M0
M6
Safety Endpoint
