# Supplementary material for: Efficacy and safety of peanut epicutaneous immunotherapy in patients with atopic comorbidities
Source: J Allergy Clin Immunol Glob. 2022 Sep 22;2(1):69–75. doi: 10.1016/j.jacig.2022.07.009 (PMC10509968; doi:10.1016/j.jacig.2022.07.009)
Supplement: Fig E2 [file mmc9.pptx]

## Slide 1
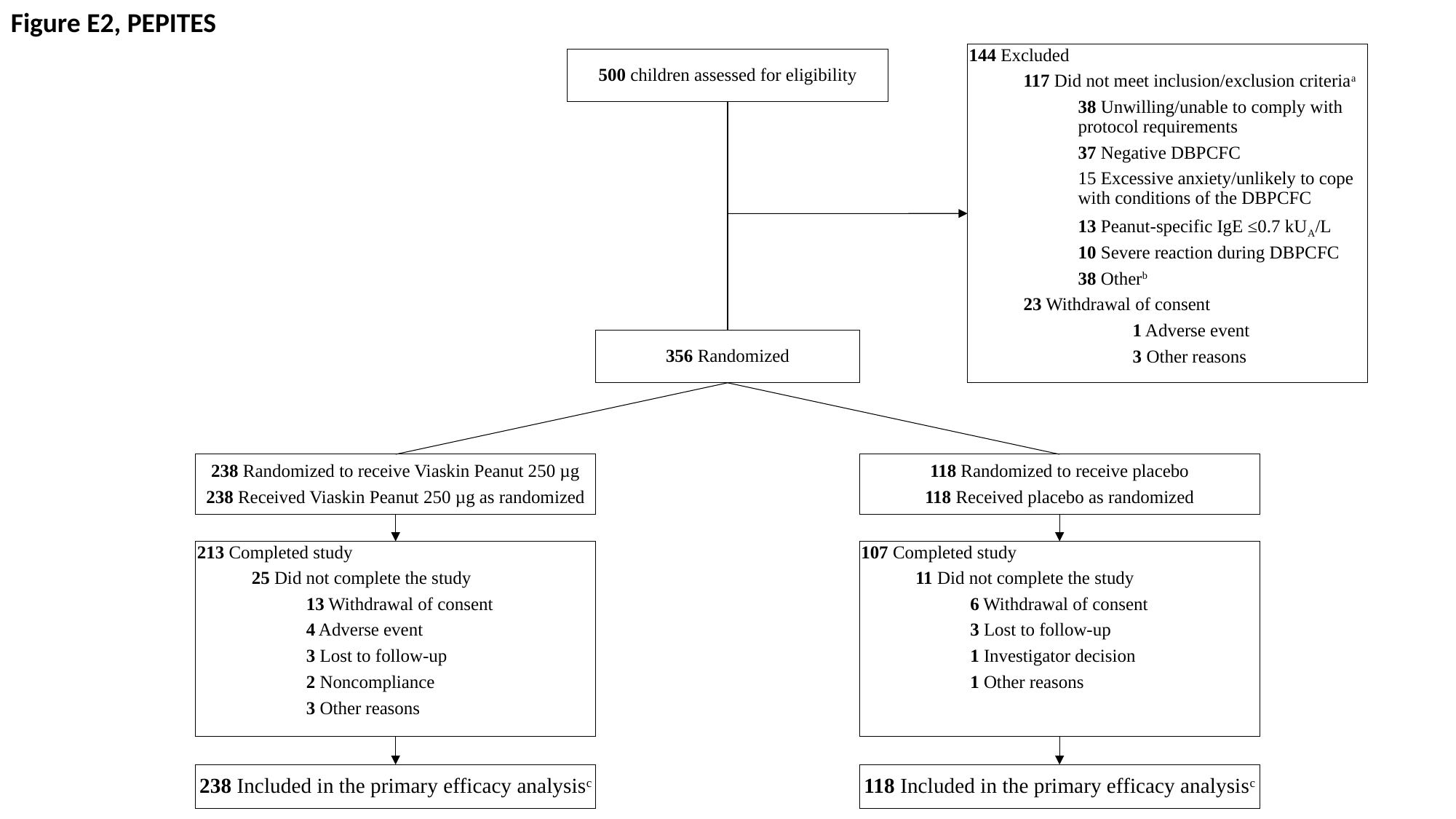

Figure E2, PEPITES
144 Excluded
117 Did not meet inclusion/exclusion criteriaa
38 Unwilling/unable to comply with protocol requirements
37 Negative DBPCFC
15 Excessive anxiety/unlikely to cope with conditions of the DBPCFC
13 Peanut-specific IgE ≤0.7 kUA/L
10 Severe reaction during DBPCFC
38 Otherb
23 Withdrawal of consent
	1 Adverse event
	3 Other reasons
500 children assessed for eligibility
356 Randomized
238 Randomized to receive Viaskin Peanut 250 µg
238 Received Viaskin Peanut 250 µg as randomized
118 Randomized to receive placebo
118 Received placebo as randomized
213 Completed study
25 Did not complete the study
13 Withdrawal of consent
4 Adverse event
3 Lost to follow-up
2 Noncompliance
3 Other reasons
107 Completed study
11 Did not complete the study
6 Withdrawal of consent
3 Lost to follow-up
1 Investigator decision
1 Other reasons
238 Included in the primary efficacy analysisc
118 Included in the primary efficacy analysisc
