# Supplementary material for: Efficacy and safety of peanut epicutaneous immunotherapy in patients with atopic comorbidities
Source: J Allergy Clin Immunol Glob. 2022 Sep 22;2(1):69–75. doi: 10.1016/j.jacig.2022.07.009 (PMC10509968; doi:10.1016/j.jacig.2022.07.009)
Supplement: Fig E3 [file mmc10.pptx]

## Slide 1
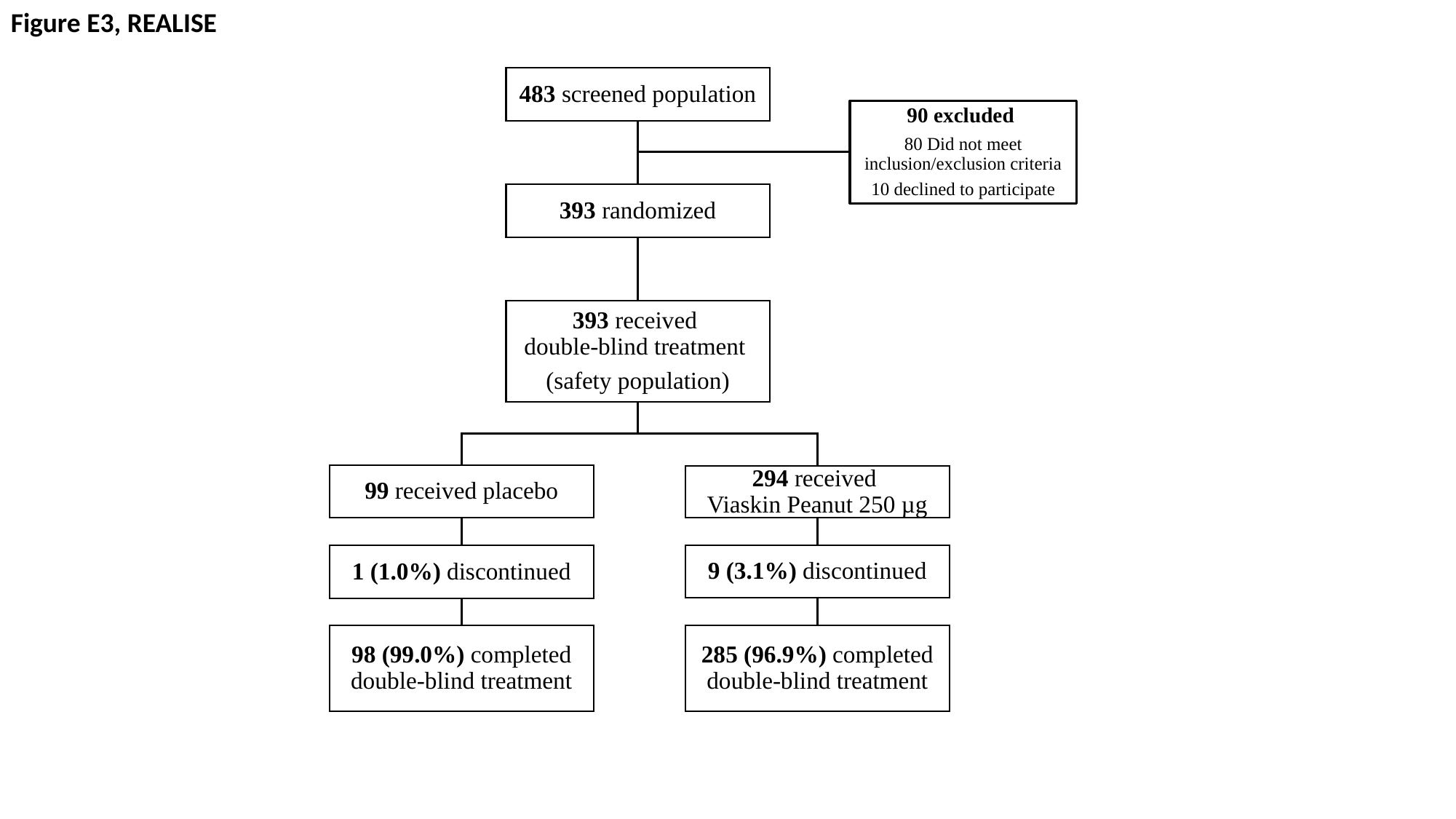

Figure E3, REALISE
483 screened population
90 excluded
80 Did not meet inclusion/exclusion criteria
10 declined to participate
393 randomized
393 received
double-blind treatment
(safety population)
99 received placebo
294 received
Viaskin Peanut 250 µg
1 (1.0%) discontinued
9 (3.1%) discontinued
98 (99.0%) completed double-blind treatment
285 (96.9%) completed double-blind treatment
