# Supplementary material for: Efficacy and safety of peanut epicutaneous immunotherapy in patients with atopic comorbidities
Source: J Allergy Clin Immunol Glob. 2022 Sep 22;2(1):69–75. doi: 10.1016/j.jacig.2022.07.009 (PMC10509968; doi:10.1016/j.jacig.2022.07.009)
Supplement: Fig E4 [file mmc11.pptx]

## Slide 1
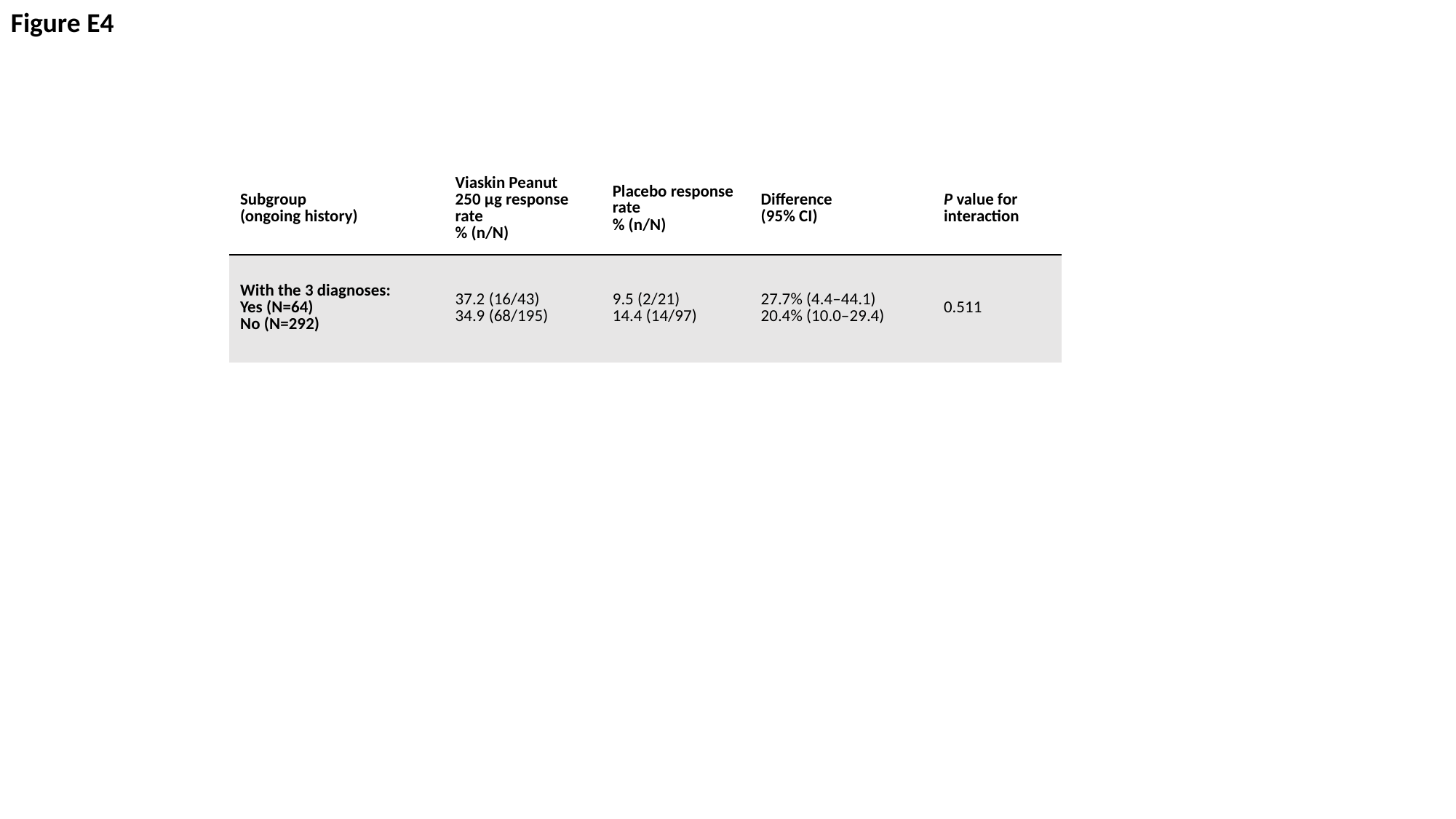

Figure E4
| Subgroup (ongoing history) | Viaskin Peanut  250 µg response rate % (n/N) | Placebo response rate % (n/N) | Difference (95% CI) | P value for interaction |
| --- | --- | --- | --- | --- |
| With the 3 diagnoses: Yes (N=64) No (N=292) | 37.2 (16/43) 34.9 (68/195) | 9.5 (2/21) 14.4 (14/97) | 27.7% (4.4–44.1) 20.4% (10.0–29.4) | 0.511 |
